# Nickel-catalyzed enantioselective reductive carbo-acylation of alkenes

**DOI:** 10.1038/s42004-020-0292-3

**Published:** 2020-04-03

**Authors:** Yun Lan, Chuan Wang

**Affiliations:** Hefei National Laboratory for Physical Science at the Microscale, Department of Chemistry, Center for Excellence in Molecular Synthesis, University of Science and Technology of China, Hefei, Anhui 230026 People’s Republic of China

**Keywords:** Synthetic chemistry methodology, Asymmetric catalysis

## Abstract

Recently, transition-metal-catalyzed asymmetric dicarbofunctionalization of tethered alkenes has emerged as a powerful method for construction of chiral cyclic carbo- and heterocycles. However, all these reactions rely on facially selective arylmetalation of the pendant olefinic unit. Here, we successfully apply acylnickelation as the enantiodetermining step in the asymmetric nickel-catalyzed reductive carbo-acylation of aryl carbamic chloride-tethered alkenes with primary and secondary alkyl iodides as well as benzyl chlorides as the coupling partners, using manganese as a reducing agent. By circumventing the use of pre-generated organometallics, this reductive strategy enables the synthesis of diverse enantioenriched oxindoles bearing a quaternary stereogenic center under mild reaction conditions with high tolerance of a broad range of functional moieties.

## Abstract (web summary)

Enantioselective dicarbofunctionalization of tethered alkenes typically relies on facially selective arylmetalation as a key step. Here, a nickel-catalyzed reductive carboacylation of alkenes and alkyl iodides which features acylnickelation as the enantiodetermining step is reported.

## Introduction

**Fig. 1:**
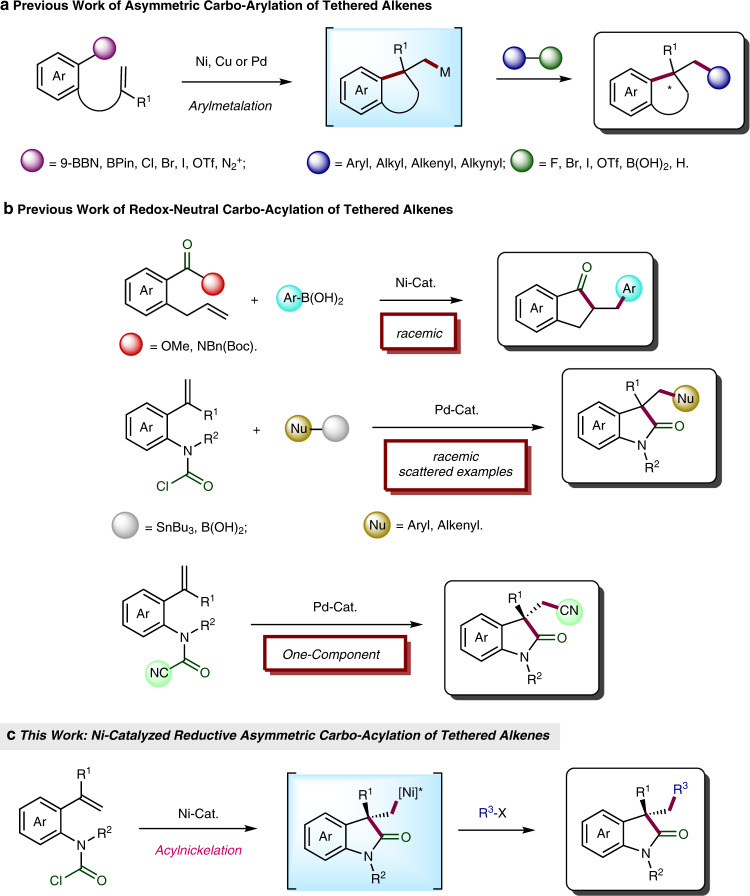
Transition-metal-catalyzed difunctionalization of alkenes. **a** Asymmetric dicarbofunctionalization of tethered alkenes involving arylmetalation. **b** Redox-neutral carbo-acylation. **c** Ni-catalyzed reductive asymmetric carbo-acylation of tethered alkenes.

Transition-metal-catalyzed dicarbofunctionalization consisting of a cyclization/cross-coupling cascade provides a powerful method to access various benzene-fused cyclic compounds starting from tethered alkenes^1–13^. Both redox-neutral^2–7^ and reductive^8–13^ strategies have been successfully applied in this reaction. Particularly, reductive dicarbofunctionalization represents a step-economical approach with high functionality tolerance through circumventing the use of organometallics as the coupling partner, and thus gains growing interest from organic chemists^14–24^. Notably, a few enantioselective two-component dicarbofunctionalizations were developed by Fu^25^, Brown^26^, Kong^27–30^, Shu^31^, Zhang^32,33^, and our group^34^ in recent years, but all these reactions rely on a facially selective intramolecular arylmetalation of the pendant olefinic unit as the enantiodetermining step (Fig. 1a). Therefore, establishing a reaction model with new retrosynthetic disconnections for asymmetric dicarbofunctionalization is highly desired for expansion of the reaction scope.

On the other side, methyl ester^35^, activated carbamate^36^, and carbamoyl chloride^37,38^ are known to undergo oxidative addition to low-valent Ni or Pd followed by intramolecular migratory insertion to an incorporated olefin in racemic fashion. The resultant cyclic alkyl metal species can be subsequently trapped by various nucleophiles in a redox-neutral pathway. Moreover, Takemoto et al. reported a one-component enantioselective carbo-acylation of alkenes with tethered carbamoyl cyanides^39^ (Fig. 1b). However, the reductive two-component carbo-acylation of appended alkenes involving termination with an electrophile remains still elusive^15^, let alone its enantioselective variant. Only very recently, Lautens et al. reported a redox-neutral asymmetric acyl-borylation of alkenes^40^.

Herein, we report a Ni-catalyzed asymmetric reductive carbo-acylation of aryl carbamic acid chloride-tethered alkenes with alkyl halides as the coupling partner, in which the intramolecular acylnickelation serves as the enantiodetermining step, to construct the oxindole motif bearing a challenging quaternary stereocenter featured in numerous biologically active compounds^41^ (Fig. 1c).

## Results

### Substrate scope of the racemic variant of Ni-catalyzed carbo-acylation

**Fig. 2:**
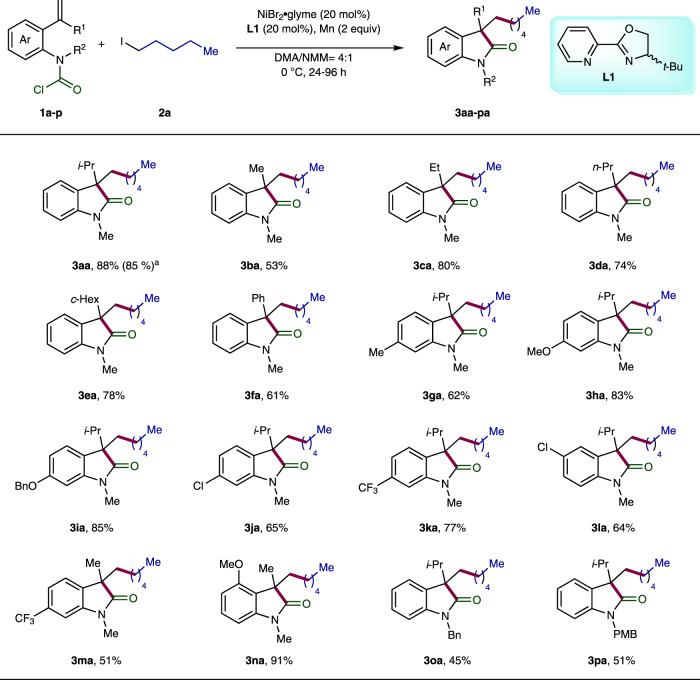
Substrate scope of carbamic chlorides. Reactions were performed on a 0.2 mmol scale of the carbamoyl chlorides **1a-p** using 2.0 equiv of *n*-pentyl iodide (**2a**), 20 mol% NiBr2·glyme, 20 mol% racemic Pyrox **L1** as ligand, and 2.0 equiv of Mn as reductant in DMA/NMM (4:1, 1.5 mL) at 0 °C. Reaction time: 24 h for **3aa-ea**, **3ga**, **3ja**, and **3la**; 48 h for **3** **ha**, **3ia**, **3ka**, **3ma**, and **3na**; and 96 h for **3fa**, **3oa**, and **3pa**. ^a^Reaction was performed on 1-mmol-scale.

**Fig. 3:**
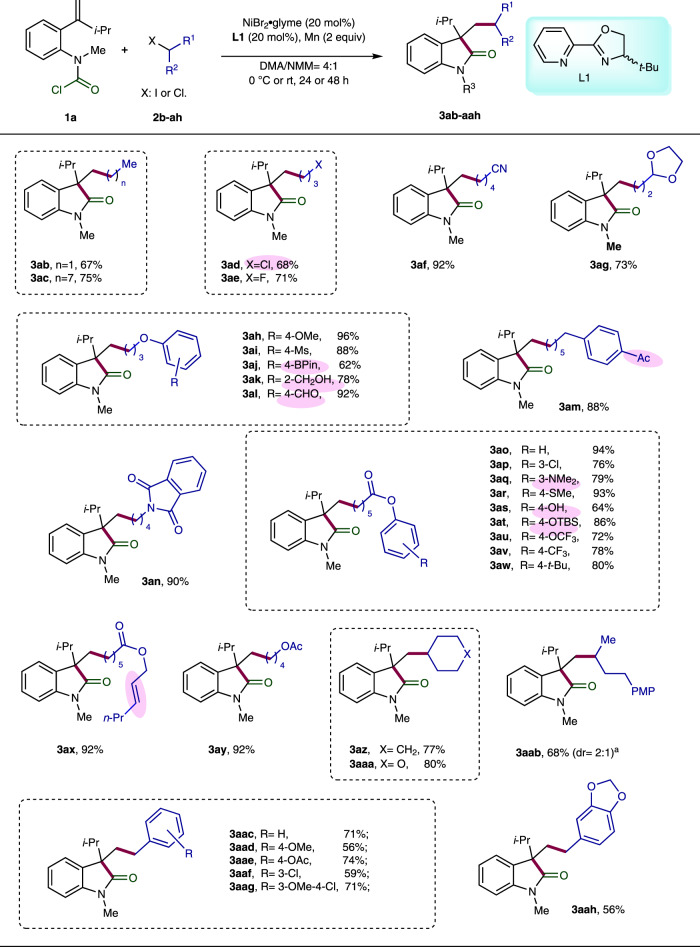
Substrate scope of alkyl halides. Unless otherwise specified, reactions were performed on a 0.2 mmol scale of the carbamoyl chloride **1a** using 2.0 equiv of alkyl iodides **2b-ab** or benzyl chlorides **2ac-ah**, 20 mol% NiBr_2_·glyme, 20 mol% racemic Pyrox **L1** as ligand, and 2.0 equiv of Mn as reductant in DMA/NMM (4:1, 1.5 mL). Reaction temperature: 0 °C for **3ab-aj**, **3al-3au**, and **3ay-aab**; room temperature for **3ak**, **3aq**, **3av-ax**, and **3aac-aah**. Reaction time: 24 h for **3ab-ah**, **3am**, and **3ay-aaa**; 48 h for **3ai-al**, **3an-ax**, and **3aab-aah**. ^a^Determined by ^1^H-spectroscopy.

Our investigation began with the racemic version of the Ni-catalyzed carbo-acylation. Systematic screening of various reaction parameters allowed us to define the optimal reaction conditions as follows: NiBr_2_·glyme as catalyst (20 mol%), racemic Pyrox **L1** as ligand (20 mol%) with Mn (2 equiv) as reductant in DMA/*N*-methyl morpholine (NMM; 4:1, 0.13 M) at 0 °C for 24 h (Supplementary Table 1). Under the optimal reaction conditions, we started to study the substrate spectrum by reacting various carbamic chloride-tethered alkenes **1a-p** with *n*-pentyl iodide (**2a**) (Fig. 2). First, permutation of the geminal substitution of the pendant olefin was carried out. Gratifyingly, the desired products were obtained in moderate to good yields for both aliphatic (**3aa-ea**) and aromatic substituent (**3fa**), wherein the latter required much longer reaction time (96 h). In the case of mono-substituted olefin (R^1^ = H), the reaction failed to deliver the desired product due to β-hydride elimination. Moreover, electron-donating or -withdrawing groups on different position of the tethered aryl ring were well tolerated, yielding the corresponding products **3ga-na** ranging from 51–91%. In the case of benzylic *N*-substitution (**3oa** and **3pa**), moderate results were achieved with extended reaction time (96 h). Subsequently, we continued to evaluate the scope of this carbo-acylation reaction by reacting diverse alkyl halides (**2b-ah**) with the carbamoyl chloride **1a** (Fig. 3). All the reactions using the primary alkyl iodides **2b-x** proceeded smoothly under standard or slightly amended conditions, furnishing the products **3ab-ax** in moderate to excellent efficiency. Of note is that good compatibility was observed for a wide range of functional moieties, including chloride (**3ad** and **3ap**), nitrile (**3af**), acetal (**3ag**), sulfone (**3ai**), boronate (**3aj**), alcohol (**3ak**), aldehyde (**3al**), ketone (**3am**), imide (**3an**), ester (**3ao-ay**), tertiary amine (**3aq**), thioether (**3ar**), phenol (**3as**), silyl ether (**3at**), and internal olefin (**3ax**). The sterically more demanding secondary alkyl iodides **2z-ab** also posed no problem, and good results were obtained for the products **3az-aab**. Remarkably, the challenging benzylic chlorides **2ac-ah** with high tendency to undergo homo-coupling also turned out to be suitable substrates, providing the products **3aac-3aah** in moderate to good yields at room temperature with prolonged reaction time (48 h). Unsuccessful alkyl sources include tertiary alkyl iodides, perfluoroalkyl iodides, α-iododifluoroacetate, and alkyl bromides.

### Optimization of the enantioselective carbo-acylation of alkenes

For optimization of the enantioselective version of the studied reaction, the aryl carbamoyl chloride **1** **f** and *n*-pentyl iodide (**2a**) were selected as the benchmark substrates (Table 1). We initially tested several Ni-precatalysts using the pyrox **L2** as ligand (entries 1–6), and the best result was achieved in the case of NiBr_2_·glyme (entry 1). Next, various chiral ligands, including BOX, PyBOX, PHOX, and BINAP were examined, but all these reactions failed to deliver the desire product (Supplementary Table 3). When the pyrox with bulkier ligand arm (**L1**) was employed, the enantiocontrol was elevated to a moderate level (entry 7). Replacing Zn by Mn as the reductant improved the efficiency significantly (entry 8). Preforming the reaction in NMP or THF afforded only inferior results (entries 9 and 10). The use of ZnI_2_ as an additive led to a higher yield (entry 11). Next, systematic tuning of the Pyrox structure was carried out. Installation of two geminal methyl groups to the oxazoline ring (**L3**) showed detrimental effect (entry 12). The use of dimethyl oxazolinol **L4** and its benzyl ether **L5** as ligands did not provide significantly improved results (entries 13 and 14). Increasing the steric hindrance of the ligand arm (**L6**) afforded only a similar outcome (entry 15). Moreover, the chiral imidazoline **L7** was also examined, giving only an inferior result (entry 16). Next, substitution on the pyridine ring of Pyrox (**L8–12**) was evaluated (entries 17–21), and it turned out that introduction of an electron-donating morpholine substituent (**L12**) could improve its performance, regarding both enantioselectivity and efficiency (entry 21). Finally, the best result (61% yield, 88% *ee*) was achieved (entry 24) through modifying the other reaction parameters, including solvent, temperature, reaction time, and catalyst loading (entries 22–24).

**Table 1:** Optimization of the reaction conditions of asymmetric carbo-acylation of alkenes.


Entry	Precatalyst	Ligand	Solvent	Reductant	Yield (%)	*ee* (%)
1	NiBr_2_·glyme	**L2**	EtOH	Zn	29	26
2	NiBr_2_	**L2**	EtOH	Zn	0	–
3	NiBr(COD)_2_	**L2**	EtOH	Zn	28	24
4	Ni(acac)_2_	**L2**	EtOH	Zn	0	–
5	NiI_2_	**L2**	EtOH	Zn	trace	–
6	NiBr_2_·glyme	**L1**	EtOH	Zn	9	71
7	NiBr_2_·glyme	**L1**	DMA	Zn	14	60
8	NiBr_2_·glyme	**L1**	DMA	Mn	50	64
9	NiBr_2_·glyme	**L1**	NMP	Mn	20	54
10	NiBr_2_·glyme	**L1**	THF	Mn	trace	–
11^a^	NiBr_2_·glyme	**L1**	DMA	Mn	62	63
12^a^	NiBr_2_·glyme	**L3**	DMA	Mn	19	33
13^a^	NiBr_2_·glyme	**L4**	DMA	Mn	50	66
14^a^	NiBr_2_·glyme	**L5**	DMA	Mn	55	63
15^a^	NiBr_2_·glyme	**L6**	DMA	Mn	44	67
16^a^	NiBr_2_·glyme	**L7**	DMA	Mn	47	57
17^a^	NiBr_2_·glyme	**L8**	DMA	Mn	trace	–
18^a^	NiBr_2_·glyme	**L9**	DMA	Mn	29	48
19^a^	NiBr_2_·glyme	**L10**	DMA	Mn	50	71
20^a^	NiBr_2_·glyme	**L11**	DMA	Mn	trace	–
21^a^	NiBr_2_·glyme	**L12**	DMA	Mn	64	85
22^b^	NiBr_2_·glyme	**L12**	DMA:NMM = 4:1	Mn	45	87
23^b^	NiBr_2_·glyme	**L12**	DMA:NMM = 2:1	Mn	trace	–
24^c^	NiBr_2_·glyme	**L12**	DMA:NMM = 4:1	Mn	65 (61)^d^	88

Reactions were performed on a 0.2 mmol scale of the carbamoyl chloride **1f** using 2.0 equiv of *n*-pentyl iodide **2a**, 10 mol% [Ni], 15 mol% ligand, and 2.0 equiv of reductant in 1.5 mL solvent at r.t. for 12 h. Yields were determined by ^1^NMR spectroscopy using CH_2_Br_2_ as an internal standard. Enantiomeric excessess were determined by HPLC analysis on chiral stationary phase.

^a^1.0 equiv of ZnI_2_ was used as an additive.

^b^10 °C, 72 h.

^c^10 °C, 96 h, NiBr_2_·glyme (20 mol%), **L12** (20 mol%).

^d^Yield of the isolated product.

### Substrate scope of the enantioselective carbo-acylation of alkenes

**Fig. 4:**
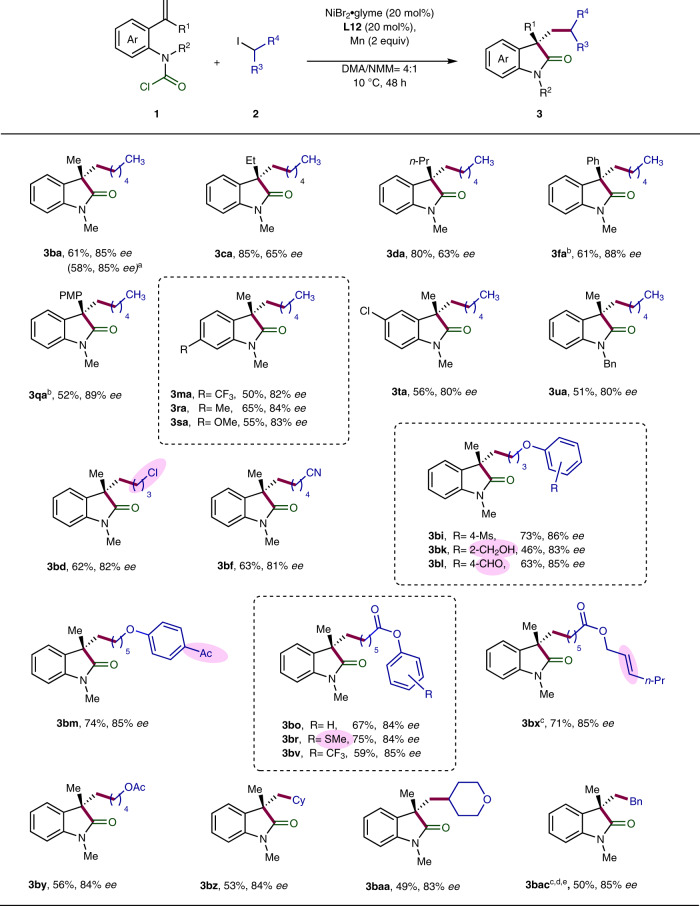
Enantioselective Ni-catalyzed carbo-acylation. Unless otherwise specified, reactions were performed on a 0.2 mmol scale of carbamoyl chlorides **1** using 2.0 equiv of alkyl iodides **2**, 20 mol% NiBr_2_·glyme, 20 mol% Pyrox **L12** as ligand, and 2.0 equiv of Mn as reductant in DMA/NMM (4:1, 1.5 mL) at 10 °C for 48 h. Enantiomeric excessess were determined by HPLC analysis on chiral stationary phase. ^a^Reaction was performed on 1-mmol-scale. ^b^Reaction time: 96 h. ^c^Reaction was performed at room temperature. ^d^Reaction time: 24 h. ^e^Benzyl chloride was used.

The scope of the asymmetric carbo-acylation was then investigated by varying the structure of both carbamic chlorides and alkyl halides (Fig. 4). It turned out that the geminal substituent of the terminal olefin has significant influence on the asymmetric induction. Bulkier alkyl group like ethyl and *n*-propyl gave rise to diminished enantioselectivities (**3ca** and **3da**). In contrast, the level of enantiocontrol remained good in the case of methyl and *p*-methoxyphenyl as substituent (**3ba** and **3qa**). Notably, the alkyl-substituted alkenes **1b-d** were found to be much more reactive than their aryl analogues **1f** and **1q**, and thus required shorter reaction time (48 h). Next, various substituted aryl carbamic chlorides were surveyed, and the corresponding products **3ma** and **3ra-ta** were obtained in good enantiomeric excesses. The benzylic *N*-substituted carbamoyl chloride **1u** was also successfully employed as precursor, giving the product **3ua** in good enantiocontrol. Furthermore, primary and secondary iodides as well as benzyl chloride all proved to be competent substrates, yielding the products in good enantioselectivities with high tolerance of various functionalities.

### Mechanistic studies

**Fig. 5:**
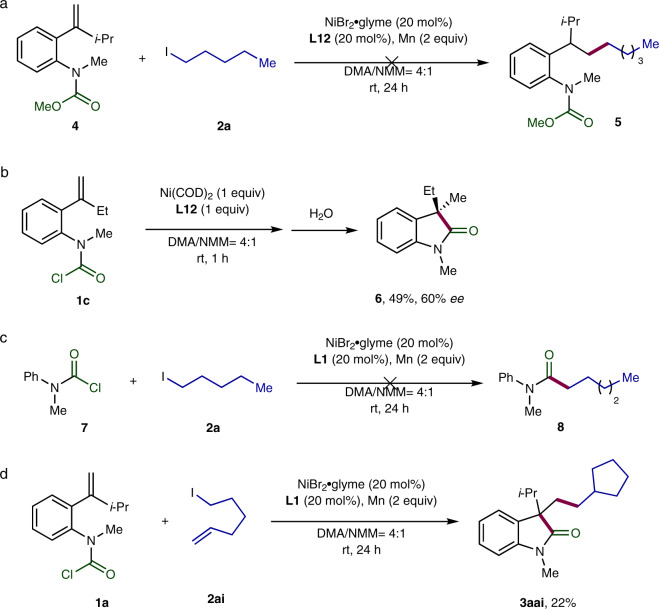
Mechanistic investigations. **a** Coupling reaction of carbamate **4** with *n*-pentyl iodide (**2a**). **b** Stoichiometric reaction of the carbamoyl chloride **1c** with Ni(COD)_2_. **c** Coupling reaction of the carbamoyl chloride **7** with *n*-pentyl iodide (**2a**). **d** Radical clock experiment of the carbamoyl chloride **1a** with 6-iodohex-1-ene (**2a**i).

A number of control experiments were conducted to disclose the mechanism of this Ni-catalyzed carbo-acylation (Fig. 5). Concerning the enantiodetermining step, two pathways are hypothesized, which are enantioselective intermolecular alkylnickelation and intramolecular acylnickelation. The first assumption turned out to be less likely, because no hydroalkylation was observed for the carbamate **4** under standard reaction conditions, which incorporates an olefinic unit with similar electronic property to the one of the carbamic chloride **1a** (Fig. 5a). In contrast, the stoichiometric reaction of the carbamoyl chloride **1c** with Ni(COD)_2_ in the presence of ligand **L12** followed by quenching with water afforded the hydroacylation product **6** in 60% *ee*, which is similar to the corresponding catalytic carbo-acylation, arguing for the intramolecular Ni(II)-mediated migratory insertion as the enantiodetermining step (Fig. 5b). Furthermore, no cross-coupling reaction occurred when treating methyl(phenyl)carbamic chloride (**7**) with *n*-pentyl iodide, suggesting that the addition of the alkyl group to the Ni center proceeds likely after the intramolecular migratory insertion step in the carbo-acylation (Fig. 5c). In the case of 6-iodohex-1-ene (**2ai**) as a radical clock, the cyclization to form the cyclopentane was found to precede the cross-coupling step to provide compound **3aai** as the product, which indicates the generation of alkyl radicals starting from the corresponding iodides in this Ni-catalyzed reaction (Fig. 5d).

**Fig. 6:**
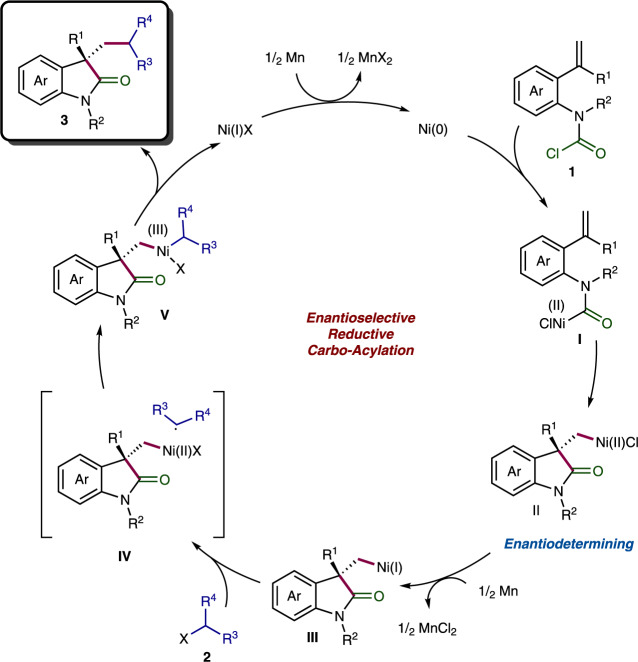
Proposed reaction mechanism for the Ni-catalyzed asymmetric reductive carbo-acylation reaction. Intramolecular acylnickelation is proposed to be the enantiodetermining step.

Relying on the aforementioned experimental evidence, we tentatively proposed the following mechanism (Fig. 6). Initially, Ni(0) is generated under reductive conditions, and then undergo oxidative addition with the carbamoyl chlorides **1** to deliver the Ni(II) complex **I**, which performs subsequently the enantiodetermining migratory insertion to the pendant olefin. Next, Mn-mediated reduction of the cyclic resultant Ni(II) species **II** enables the formation of the Ni(I) intermediate **III**. The following cage-bound (**IV**) oxidative addition with the alkyl halides **2** results in the generation of the Ni(III) species **V**. Upon facile reductive elimination from **V**, the carbo-acylation products **3** are provided. Finally, the released Ni(I)X is reduced by Mn to give the Ni(0) species for the next catalytic cycle.

Here, we developed a Ni-catalyzed carbo-acylation of tethered alkenes with both unactivated alkyl iodides and benzyl chlorides via a reductive strategy. This cyclization/cross-coupling cascade reaction furnishes diverse functional-group-rich 3,3-disubstituted oxindoles with formation of two C–C σ-bonds. The enantioselective version of this reaction was also realized by employing a chiral Ni–Pyrox complex as catalyst, enabling the construction of a quaternary stereocenter in moderate to high enantioselectivities. The preliminary mechanistic investigations indicate a Ni(II)-mediated intramolecular migratory insertion as the enantiodetermining step.

## Methods

### Synthesis and characterization

See Supplementary Methods (general information about chemicals and analytical methods, synthetic procedures, ^1^H and ^13^C NMR data, and HPLC data), Supplementary Figs. 12–36 (HPLC chromatograms), and Supplementary Figs. 37–244 (^1^H and ^13^C NMR spectra).

### General procedure for racemic variant of the Ni-catalyzed carbo-acylation

Racemic oxazoline ligand **L1** (8.2 mg, 0.04 mmol, 20 mol%), carbamoyl chlorides **1** (if solid, 0.2 mmol, 1.0 equiv), and alkyl iodides **2** (if solid, 0.4 mmol, 2.0 equiv) were added to a reaction tube equipped with a stir bar. In a nitrogen-filled glovebox, NiBr_2_·glyme (12.3 mg, 0.04 mmol, 20 mol%), and manganese dust (22 mg, 0.4 mmol, 2 equiv) were added to the mixture. The reaction tube was sealed and removed from the glovebox. Next, anhydrous DMA (1.2 mL) and NMM (0.3 mL) were added, followed by the addition of carbamoyl chlorides **1** (if liquid, 0.2 mmol, 1 equiv) and alkyl iodides **2** (if liquid, 0.4 mmol, 2.0 equiv) under the protection of nitrogen. Then the resulting mixture was stirred at corresponding temperature for 24–96 h (Supplementary Fig. 5). The reaction was quenched with sat. aq. NH_4_Cl solution (5 mL) and diluted with water (10 mL). The aqueous layer was extracted three times with EtOAc, and the combined organic layers were washed with brine (20 mL), dried over MgSO_4_, filtered, and concentrated under reduced pressure. The residue was purified through column chromatography on silica gel (petroleum ether/ethyl acetate) to afford the desired product **3**.

### General procedure for asymmetric Ni-catalyzed carbo-acylation

Chiral oxazoline **L12** (11.6 mg, 0.04 mmol, 20 mol%), carbamoyl chlorides **1** (if solid, 0.2 mmol, 1 equiv), and alkyl iodides **2** (if solid, 0.4 mmol, 2.0 equiv) were added to a reaction tube equipped with a stir bar. In a nitrogen-filled glovebox, NiBr_2_·glyme (12.3 mg, 0.04 mmol, 20 mol%), and manganese dust (22 mg, 0.4 mmol, 2 equiv) were added to the mixture. The reaction tube was sealed and removed from the glovebox. Next, anhydrous DMA (1.2 mL) and NMM (0.3 mL) were added, followed by the addition of carbamoyl chlorides **1** (if liquid, 0.2 mmol, 1 equiv) and alkyl iodides **2** (if liquid, 0.4 mmol, 2.0 equiv) under the protection of nitrogen. Then the resulting mixture was stirred at corresponding temperature for 24–96 h (Supplementary Fig. 6). The reaction was quenched with sat. aq. NH_4_Cl solution (5 mL) and diluted with water (10 mL). The aqueous layer was extracted three times with EtOAc, and the combined organic layers were washed with brine (20 mL), dried over MgSO_4_, filtered, and concentrated under reduced pressure. The residue was purified through column chromatography on silica gel (petroleum ether/ethyl acetate) to afford the desired product **3**.

### Synthesis of starting materials and chiral ligand L12

For more details, see Supplementary Figs. 1–4.

### Detailed optimization of the reaction conditions for asymmetric carbo-acylation

For more details, see Supplementary Tables 2–9.

### Procedures of control experiments for mechanistic studies

For more details, see Supplementary Figs. 7–10.

### Determination of the absolute configuration

For determination of the absolute configuration of triol product **3bac**, see Supplementary Fig. 11. The stereochemistry of all the other products was assigned by assuming a common reaction pathway.

## Supplementary information

Supplementary Information

## Data Availability

The optimization of reaction conditions, the experimental procedure, and characterization data of new compounds are available within Supplementary Information. Any further relevant data are available from the authors upon reasonable request.
