# Supplementary material for: Nickel-catalyzed enantioselective reductive carbo-acylation of alkenes
Source: Commun Chem. 2020 Apr 3;3:45. doi: 10.1038/s42004-020-0292-3 (PMC9814080; doi:10.1038/s42004-020-0292-3)
Supplement: Supplementary file 1 — Supplementary Information [file 42004_2020_292_MOESM1_ESM.pdf]

Supplementary Methods

General Information.

^{1}H NMR, ^{13}C NMR and ^{19}F NMR spectra were recorded on a Bruker Advance 400 MHz or 500 MHz NMR spectrometers at ambient temperature in $CDCl_3$. The chemical shifts are given in ppm relative to tetramethylsilane [^{1}H: δ =($SiMe_4$) = 0.00 ppm] as an internal standard or relative to the resonance of the solvent [^{1}H: δ ($CDCl_3$) = 7.26, ^{13}C: δ ($CDCl_3$) = 77.16 ppm]. Multiplicities were given as: s (singlet), d (doublet), t (triplet), q (quartet), p (pentet), dd (doublet of doublets), dt (doublet of triplets), m (multiplets), etc. Coupling constants are reported as *J* values in Hz. High resolution mass spectral analysis (HRMS) was performed on Waters XEVO G2 Q-TOF. HPLC was performed on Thermo UltiMate 3000. Flash chromatography was performed using 200-300 mesh silica gel with the indicated solvent system.

Unless otherwise stated, all reactions were set up on 10 mL reaction tube and carried out under nitrogen. $NiBr_2$•glyme was prepared according to the known procedure.[1] *N*-Methyl morpholine was purchased from Aladdin (purified by redistillation，99.5%). All other solvents were purchased from Energy Chemical and used as received. Other commercial reagents were purchased from Sigma-Aldrich, Alfa Aesar, TCI, Strem, Acros, and Adamas-beta China and were used as received.

Reactions were monitored through thin layer chromatography [Merck 60 F254 precoated silica gel plate (0.2 mm thickness)]. Subsequent to elution, spots were visualized using UV radiation (254 nm) on Spectroline Model ENF-24061/F 254 nm. Other visualization methods include staining with a basic solution of potassium permanganate or acidic solution of ceric molybdate, followed by heating.

Carbamoyl chlorides **1b**, **1f**, **1q** are known compounds in the literature.[2]

Supplementary Figure 1. General procedure (A) for the preparation of carbamoyl chlorides.

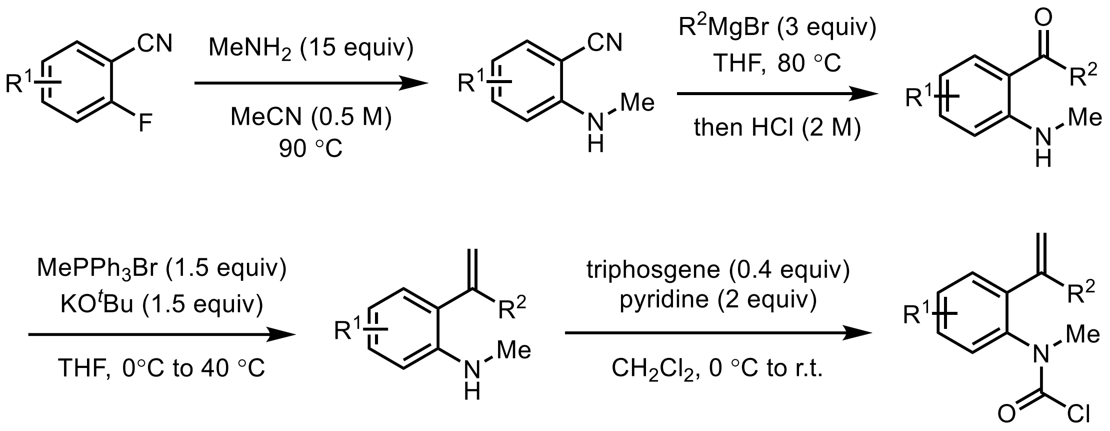

The carbamoyl chlorides were prepared according to the known procedure with slight modification.[2]

Step 1: 2-Fluorobenzonitrile (1 equiv) was added to a 100 mL screw-cap vial equipped with a magnetic stir bar, then acetonitrile (0.5 M) and methylamine (15 equiv, 40% wt. in H_2O) were added, successively. The reaction was stirred at 90 ºC for 12 h. After cooling to room temperature, the reaction was diluted with ethyl acetate and washed twice with water. The organic layer was dried over magnesium sulfate, filtered, and dried. The residue was purified by silica gel column chromatography in ethyl acetate/petroleum ether to give the desired aniline.

Step 2: The *N*-methyl aniline was stirred in THF (0.3 M) at 0 ºC. Then Grignard reagent (3.0 M, 3 equiv) was added slowly. The reaction was heated to 80 ºC for 6 h. After cooling to 0 ºC, the reaction was quenched slowly with 2M aq. HCl. The imine hydrolysis was stirred at room temperature for 1 h. In some cases the imine hydrolysis required refluxing 4M HCl in EtOH overnight to convert to the corresponding ketones. The reaction was quenched with Na_2CO_3 (aq.), then diluted with EtOAc and washed twice with water. The organic layer was dried over magnesium sulfate, filtered, and concentrated under reduced pressure. The residue was purified by silica gel column chromatography in ethyl acetate/petroleum ether to give the desired ketone.

Step 3: Potassium *tert*-butoxide (1.5 equiv) was added slowly to methyl triphenyl

phosphonium bromide (1.5 equiv) in THF (0.3 M) at 0 ºC. The suspension turned yellow upon addition of the base. The suspension was warmed to 40 ºC and stirred for 1 h. The reaction was cooled to 0 ºC and the 2-aminophenones were added in THF (10 mL). The reaction was warmed to 40 ºC again and stirred until consumption of starting material was observed by TLC (typically 2 h). Upon completion the reaction was filtered over a silica plug eluting with ethyl acetate. The crude styrene was concentrated under reduced pressure and used in next step without further purification.

Step 4: The methylated amine was dissolved in dichloromethane (0.3 M) and cooled to 0 ºC. Then pyridine (2 equiv) was added followed by triphosgene (0.4 equiv). The reaction was warmed to room temperature and stirred until completion indicated by TLC (typically 2 h). The reaction was quenched with 1 M HCl and extracted twice with dichloromethane. The organic layers were dried over magnesium sulfate, filtered, and concentrated under reduced pressure. The crude starting material was purified by silica gel column chromatography in ethyl acetate/petroleum ether to give the desired carbamoyl chloride.

Supplementary Figure 2. General procedure (B) for the preparation of carbamoyl chlorides.

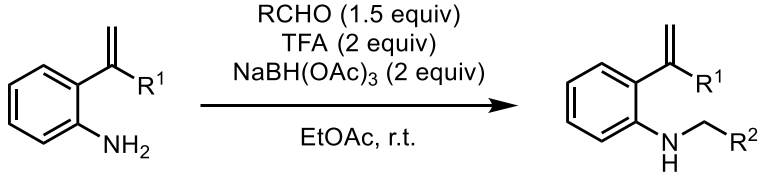

Aminostyrenes (prepared from 2-aminobenzonitriles via General Procedure (**A**)) was dissolved in ethyl acetate (0.25 M). The aldehydes (1.5 equiv) were added followed by trifluoroacetic acid (2 equiv). The reaction was stirred for 30 minutes then sodium triacetoxyborohydride (2 equiv) was added. After stirring for 2 h the reaction was then quenched with 4 M aq. NaOH, diluted with ethyl acetate and washed twice with brine. The organic layer was dried over magnesium sulfate, filtered, and concentrated under reduced pressure. The residue was purified by silica gel column chromatography.

The alkylated amines were carried forward following General Procedure (**A**) to obtain

the carbamoyl chloride.

Methyl(2-(3-methylbut-1-en-2-yl)phenyl)carbamic chloride (1a).

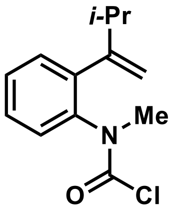

Prepared following general procedure **A**. The crude product was purified by silica gel column chromatography (4% EtOAc in petroleum ether) to provide the title compound as a yellow solid in 69% yield. The product was isolated as a mixture of two rotamers. ^{1}H NMR and ^{13}C NMR data listed are for the major rotamer.

^{1}H NMR (400 MHz, Chloroform-*d*) δ 7.46 – 7.11 (m, 4H), 5.25 (t, *J* = 1.4 Hz, 1H), 5.05 (s, 1H), 3.25 (s, 3H), 2.66 – 2.49 (m, 1H), 1.17 (d, *J* = 6.7 Hz, 3H), 1.01 (d, *J* = 6.9 Hz, 3H).

^{13}C NMR (101 MHz, Chloroform-*d*) δ 153.8, 149.7, 141.5, 140.2, 130.8, 129.0, 128.7, 128.2, 113.4, 39.9, 33.0, 22.6, 21.4.

(3-Methoxy-2-(prop-1-en-2-yl)phenyl)(methyl)carbamic chloride (1c).

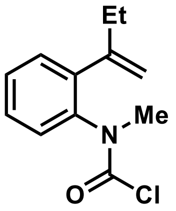

Prepared following general procedure **A**. The crude product was purified by silica gel column chromatography (4% EtOAc in petroleum ether) to provide the title compound as a colorless oil in 57% yield. The product was isolated as a mixture of two rotamers. ^{1}H NMR and ^{13}C NMR data listed are for the major rotamer.

^{1}H NMR (400 MHz, Chloroform-*d*) δ 7.42 – 7.12 (m, 4H), 5.32 – 5.13 (m, 1H), 5.06 (s, 1H), 3.25 (s, 3H), 2.51 – 2.26 (m, 2H), 1.08 (t, *J* = 7.4 Hz, 3H).

^{13}C NMR (101 MHz, Chloroform-*d*) δ 149.6, 148.4, 141.2, 140.2, 130.3, 128.9, 128.8, 128.2, 114.6, 40.0, 29.5, 12.6.

Methyl(2-(pent-1-en-2-yl)phenyl)carbamic chloride (1d).

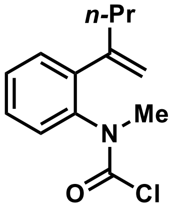

Prepared following general procedure **A**. The crude product was purified by silica gel column chromatography (4% EtOAc in petroleum ether) to provide the title compound as a pale yellow oil in 44% yield.

The product was isolated as a mixture of two rotamers. ^{1}H NMR and ^{13}C NMR data listed are for the major rotamer.

^{1}H NMR (400 MHz, Chloroform-*d*) δ 7.46 – 7.10 (m, 4H), 5.24 (s, 1H), 5.05 (s, 1H), 3.26 (s, 3H), 2.44 – 2.25 (m, 2H), 1.53 – 1.36 (m, 2H), 0.93 (t, *J* = 7.3 Hz, 3H).

^{13}C NMR (101 MHz, Chloroform-*d*) δ 149.6, 147.2, 141.0, 140.2, 130.5, 128.9, 128.8, 128.2, 115.8, 39.9, 38.7, 21.3, 13.7.

(2-(1-Cyclohexylvinyl)phenyl)(methyl)carbamic chloride (1e).

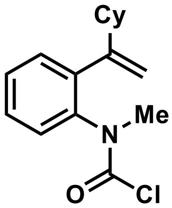

Prepared following general procedure **A**. The crude product was purified by silica gel column chromatography (4% EtOAc in petroleum ether) to provide the title compound as a colorless oil in 51% yield. The product was isolated as a mixture of two rotamers. ^{1}H NMR and ^{13}C NMR data listed are for the major rotamer.

^{1}H NMR (400 MHz, Chloroform-*d*) δ 7.42 – 7.13 (m, 4H), 5.21 (t, *J* = 1.4 Hz, 1H), 5.04 (s, 1H), 3.24 (s, 3H), 2.27 – 2.09 (m, 1H), 1.93 – 1.65 (m, 5H), 1.37 – 1.13 (m, 4H), 1.12 – 0.97 (m, 1H).

^{13}C NMR (101 MHz, Chloroform-*d*) δ 153.1, 149.7, 141.6, 140.2, 130.9, 129.0, 128.6, 128.1, 113.6, 43.0, 39.9, 33.5, 31.9, 26.7, 26.6, 26.2.

Methyl(5-methyl-2-(3-methylbut-1-en-2-yl)phenyl)carbamic chloride (1g).

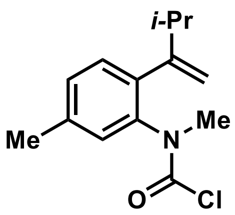

Prepared following general procedure **A**. The crude product was purified by silica gel column chromatography (4% EtOAc in petroleum ether) to provide the title compound as a pale yellow oil in 41% yield. The product was isolated as a mixture of two rotamers. ^{1}H NMR and ^{13}C NMR data listed are for the major rotamer.

^{1}H NMR (400 MHz, Chloroform-*d*) δ 7.20 – 6.93 (m, 3H), 5.22 (d, *J* = 1.4 Hz, 1H), 5.02 (d, *J* = 1.0 Hz, 1H), 3.23 (s, 3H), 2.67 – 2.48 (m, 1H), 2.37 (s, 3H), 1.16 (d, *J* = 6.7 Hz, 3H), 1.00 (d, *J* = 6.9 Hz, 3H).

^{13}C NMR (101 MHz, Chloroform-*d*) δ 153.7, 149.7, 140.0, 138.4, 138.2, 130.6,

129.4 (2C), 113.2, 39.9, 33.0, 22.6, 21.4, 20.8.

(5-Methoxy-2-(3-methylbut-1-en-2-yl)phenyl)(methyl)carbamic chloride (1h).

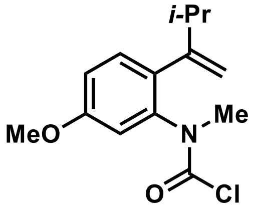

Prepared following general procedure **A**. The crude product was purified by silica gel column chromatography (8% EtOAc in petroleum ether) to provide the title compound as a yellow solid in 69% yield. The product was isolated as a mixture of two rotamers. ^{1}H NMR and ^{13}C NMR data listed are for the major rotamer.

^{1}H NMR (400 MHz, Chloroform-*d*) δ 7.15 (d, *J* = 8.5 Hz, 1H), 6.90 (dd, *J* = 8.6, 2.7 Hz, 1H), 6.72 (d, *J* = 2.6 Hz, 1H), 5.21 (t, *J* = 1.4 Hz, 1H), 5.01 (t, *J* = 1.0 Hz, 1H), 3.83 (s, 3H), 3.23 (s, 3H), 2.62 – 2.46 (m, 1H), 1.16 (d, *J* = 6.7 Hz, 3H), 1.00 (d, *J* = 6.9 Hz, 3H).

^{13}C NMR (101 MHz, Chloroform-*d*) δ 159.1, 153.3, 149.6, 140.9, 133.7, 131.5, 114.4, 114.3, 113.2, 55.5, 39.8, 33.1, 22.6, 21.4.

(5-(Benzyloxy)-2-(3-methylbut-1-en-2-yl)phenyl)(methyl)carbamic chloride (1i).

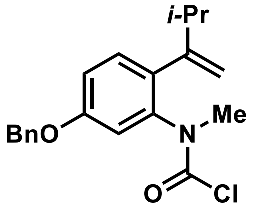

Prepared following general procedure **A**. The crude product was purified by silica gel column chromatography (8% EtOAc in petroleum ether) to provide the title compound as a pale yellow oil in 81% yield. The product was isolated as a mixture of two rotamers. ^{1}H NMR and ^{13}C NMR data listed are for the major rotamer.

^{1}H NMR (400 MHz, Chloroform-*d*) δ 7.56 – 7.28 (m, 5H), 7.14 (d, *J* = 8.5 Hz, 1H), 6.97 (dd, *J* = 8.6, 2.6 Hz, 1H), 6.82 (dd, *J* = 18.6, 2.6 Hz, 1H), 5.21 (t, *J* = 1.4 Hz, 1H), 5.13 – 4.95 (m, 3H), 3.22 (s, 3H), 2.64 – 2.40 (m, 1H), 1.15 (d, *J* = 6.7 Hz, 3H), 1.00 (d, *J* = 6.9 Hz, 3H).

^{13}C NMR (101 MHz, Chloroform-*d*) δ 158.3, 153.3, 149.6, 140.9, 136.4, 134.0, 131.6, 128.7 (2C), 128.2, 127.6 (2C), 115.29, 115.28, 113.2, 70.4, 39.8, 33.2, 22.7, 21.5.

(5-Chloro-2-(3-methylbut-1-en-2-yl)phenyl)(methyl)carbamic chloride (1j).

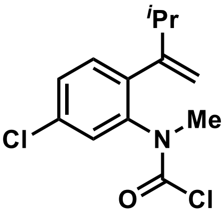

Prepared following general procedure **A**. The crude product was purified by silica gel column chromatography (4% EtOAc in petroleum ether) to provide the title compound as a yellow solid in 72% yield. The product was isolated as a mixture of two rotamers. ^{1}H NMR and ^{13}C NMR data listed are for the major rotamer.

^{1}H NMR (400 MHz, Chloroform-*d*) δ 7.40 – 7.05 (m, 3H), 5.27 (t, *J* = 1.2 Hz, 1H), 5.04 (s, 1H), 3.23 (s, 3H), 2.64 – 2.47 (m, 1H), 1.16 (d, *J* = 6.7 Hz, 3H), 1.01 (d, *J* = 6.9 Hz, 3H).

^{13}C NMR (101 MHz, Chloroform-*d*) δ 152.8, 149.3, 141.0, 140.1, 133.2, 131.9, 129.2, 129.0, 114.1, 39.8, 33.0, 22.5, 21.4.

Methyl(2-(3-methylbut-1-en-2-yl)-5-(trifluoromethyl)phenyl)carbamic chloride (1k).

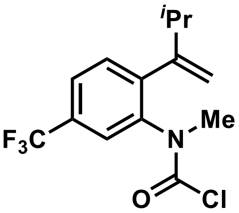

Prepared following general procedure **A**. The crude product was purified by silica gel column chromatography (3% EtOAc in petroleum ether) to provide the title compound as a yellow oil in 51% yield. The product was isolated as a mixture of two rotamers. ^{1}H NMR and ^{13}C NMR data listed are for the major rotamer.

^{1}H NMR (400 MHz, Chloroform-*d*) δ 7.70 – 7.32 (m, 3H), 5.33 (dd, *J* = 1.6, 0.8 Hz, 1H), 5.09 (s, 1H), 3.27 (s, 3H), 2.70 – 2.52 (m, 1H), 1.19 (d, *J* = 6.7 Hz, 3H), 1.02 (d, *J* = 6.9 Hz, 3H).

^{13}C NMR (101 MHz, Chloroform-*d*) δ 152.8, 149.2, 145.4, 140.5, 131.6, 130.7 (q, *J* = 33.3 Hz), 126.4 (q, *J* = 3.8 Hz), 125.5 (q, *J* = 3.6 Hz), 123.4 (q, *J* = 272.4 Hz), 114.6, 39.8, 32.8, 22.5, 21.3.

^{19}F NMR (376 MHz, Chloroform-*d*) δ −62.55 (s, 3F).

(4-Chloro-2-(3-methylbut-1-en-2-yl)phenyl)(methyl)carbamic chloride (1l).

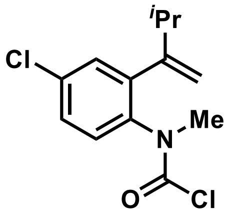

Prepared following general procedure **A**. The crude product was purified by silica gel column chromatography (4% EtOAc in petroleum ether) to provide the title compound as a yellow oil in 67% yield. The product was isolated as a mixture of two rotamers. ^{1}H NMR and ^{13}C NMR data listed are for the major rotamer.

^{1}H NMR (400 MHz, Chloroform-*d*) δ 7.31 (dd, *J* = 8.4, 2.5 Hz, 1H), 7.23 (d, *J* = 2.4 Hz, 1H), 7.13 (d, *J* = 8.4 Hz, 1H), 5.28 (dd, *J* = 1.6, 0.9 Hz, 1H), 5.07 (d, *J* = 0.9 Hz, 1H), 3.22 (s, 3H), 2.67 – 2.47 (m, 1H), 1.17 (d, *J* = 6.7 Hz, 3H), 1.02 (d, *J* = 6.9 Hz, 3H).

^{13}C NMR (101 MHz, Chloroform-*d*) δ 152.8, 149.5, 143.2, 138.7, 134.4, 130.7, 130.4, 128.3, 114.3, 39.9, 32.8, 22.6, 21.3.

Methyl(2-(prop-1-en-2-yl)-5-(trifluoromethyl)phenyl)carbamic chloride (1m).

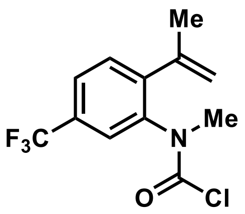

Prepared following general procedure **A**. The crude product was purified by silica gel column chromatography (4% EtOAc in petroleum ether) to provide the title compound as a yellow solid in 83% yield. The product was isolated as a mixture of two rotamers. ^{1}H NMR and ^{13}C NMR data listed are for the major rotamer.

^{1}H NMR (400 MHz, Chloroform-*d*) δ 7.66 – 7.56 (m, 1H), 7.53 – 7.48 (m, 1H), 7.45 (d, *J* = 8.2 Hz, 1H), 5.34 (t, *J* = 1.5 Hz, 1H), 5.10 (s, 1H), 3.29 (s, 3H), 2.12 (s, 3H).

^{13}C NMR (126 MHz, Chloroform-*d*) δ 149.1, 145.2, 141.3, 140.5, 130.8 (q, *J* = 33.2 Hz), 130.6, 126.2 (q, *J* = 3.6 Hz), 125.7 (q, *J* = 3.5 Hz), 123.3 (q, *J* = 272.3 Hz), 118.2, 39.9, 23.1.

^{19}F NMR (376 MHz, Chloroform-*d*) δ −62.59 (s, 3F).

(3-Methoxy-2-(prop-1-en-2-yl)phenyl)(methyl)carbamic chloride (1n).

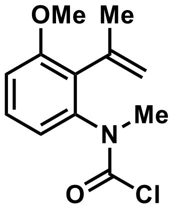

Prepared following general procedure **A**. The crude product was

purified by silica gel column chromatography (5% EtOAc in petroleum ether) to provide the title compound as a yellow solid in 55% yield. The product was isolated as a mixture of two rotamers. ^{1}H NMR and ^{13}C NMR data listed are for the major rotamer.

^{1}H NMR (400 MHz, Chloroform-*d*) δ 7.29 (t, *J* = 8.1 Hz, 1H), 7.00 – 6.76 (m, 2H), 5.45 – 5.26 (m, 1H), 5.00 (s, 1H), 3.86 (s, 3H), 3.24 (s, 3H), 2.07 (s, 3H).

^{13}C NMR (101 MHz, Chloroform-*d*) δ 157.6, 149.5, 141.4, 139.2, 131.0, 128.6, 120.7, 116.7, 111.0, 56.0, 40.4, 23.1.

Benzyl(2-(3-methylbut-1-en-2-yl)phenyl)carbamic chloride (1o).

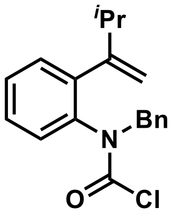

Prepared following general procedure **B**. The crude product was purified by silica gel column chromatography (4% EtOAc in petroleum ether) to provide the title compound as a white solid in 24% yield. The product was isolated as a mixture of two rotamers. ^{1}H NMR and ^{13}C NMR data listed are for the major rotamer.

^{1}H NMR (400 MHz, Chloroform-*d*) δ 7.37 – 7.05 (m, 8H), 6.68 (d, *J* = 8.9 Hz, 1H), 5.43 – 5.24 (m, 2H), 5.12 (s, 1H), 4.15 (d, *J* = 14.3 Hz, 1H), 2.79 – 2.65 (m, 1H), 1.25 (d, *J* = 6.7 Hz, 3H), 1.03 (d, *J* = 6.9 Hz, 3H).

^{13}C NMR (101 MHz, Chloroform-*d*) δ 154.4, 150.2, 141.4, 137.9, 135.5, 131.2, 130.9, 129.3 (2C), 128.7, 128.5 (2C), 128.1, 127.5, 113.6, 55.3, 32.9, 23.0, 21.3.

(4-Methoxybenzyl)(2-(3-methylbut-1-en-2-yl)phenyl)carbamic chloride (1p).

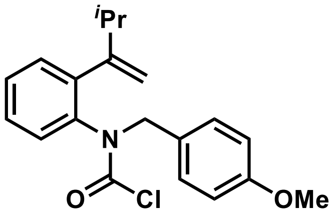

Prepared following general procedure **B**. The crude product was purified by silica gel column chromatography (10% EtOAc in petroleum ether) to provide the title compound as a yellow oil in 47% yield. The product was isolated as a mixture of two rotamers. ^{1}H NMR and ^{13}C NMR data listed are for the major rotamer.

^{1}H NMR (400 MHz, Chloroform-*d*) δ 7.31 (td, *J* = 7.5, 1.1 Hz, 1H), 7.24 (dd, *J* =

7.7, 1.7 Hz, 1H), 7.13 (td, *J* = 7.7, 1.7 Hz, 1H), 7.06 (d, *J* = 8.6 Hz, 2H), 6.78 (d, *J* = 8.7 Hz, 2H), 6.65 (dd, *J* = 7.9, 1.3 Hz, 1H), 5.31 (s, 1H), 5.29 (d, *J* = 14.2 Hz, 1H), 5.11 (s, 1H), 4.08 (d, *J* = 14.2 Hz, 1H), 3.78 (s, 3H), 2.78 – 2.65 (m, 1H), 1.24 (d, *J* = 6.7 Hz, 3H), 1.02 (d, *J* = 6.9 Hz, 3H).

^{13}C NMR (101 MHz, Chloroform-*d*) δ 159.4, 154.4, 150.0, 141.4, 137.9, 131.3, 130.8, 130.7 (2C), 128.6, 127.7, 127.5, 113.8 (2C), 113.6, 55.2, 54.8, 32.9, 23.0, 21.3.

Methyl(5-methyl-2-(prop-1-en-2-yl)phenyl)carbamic chloride (1r).

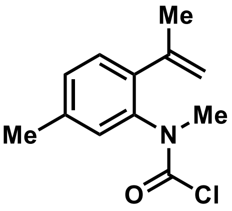

Prepared following general procedure **A**. The crude product was purified by silica gel column chromatography (4% EtOAc in petroleum ether) to provide the title compound as a yellow oil in 48% yield. The product was isolated as a mixture of two rotamers. ^{1}H NMR and ^{13}C NMR data listed are for the major rotamer.

^{1}H NMR (400 MHz, Chloroform-*d*) δ 7.22 – 7.10 (m, 2H), 7.02 – 6.99 (m, 1H), 5.25 – 5.21 (m, 1H), 5.04 – 4.99 (m, 1H), 3.25 (s, 3H), 2.37 (s, 3H), 2.08 (s, 3H).

^{13}C NMR (101 MHz, Chloroform-*d*) δ 149.6, 142.2, 139.9, 138.4, 138.3, 129.6, 129.5, 129.2, 116.5, 40.0, 23.5, 20.9.

(5-Methoxy-2-(prop-1-en-2-yl)phenyl)(methyl)carbamic chloride (1s).

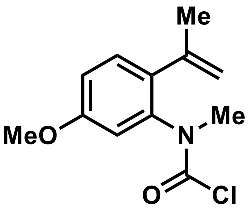

Prepared following general procedure **A**. The crude product was purified by silica gel column chromatography (8% EtOAc in petroleum ether) to provide the title compound as a yellow solid in 79% yield. The product was isolated as a mixture of two rotamers. ^{1}H NMR and ^{13}C NMR data listed are for the major rotamer.

^{1}H NMR (400 MHz, Chloroform-*d*) δ 7.23 (d, *J* = 8.5 Hz, 1H), 6.91 (dd, *J* = 8.6, 2.6 Hz, 1H), 6.72 (d, *J* = 2.6 Hz, 1H), 5.21 (t, *J* = 1.6 Hz, 1H), 5.00 (dd, *J* = 1.9, 1.0 Hz, 1H), 3.83 (s, 3H), 3.26 (s, 3H), 2.07 (s, 3H).

^{13}C NMR (101 MHz, Chloroform-*d*) δ 159.2, 149.5, 141.8, 140.8, 133.6, 130.5, 116.4, 114.5, 114.1, 55.6, 39.8, 23.6.

(4-Chloro-2-(prop-1-en-2-yl)phenyl)(methyl)carbamic chloride (1t).

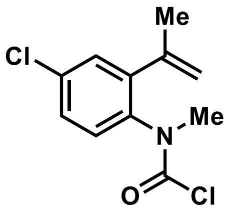

Prepared following general procedure **A**. The crude product was purified by silica gel column chromatography (4% EtOAc in petroleum ether) to provide the title compound as a yellow oil in 67% yield. The product was isolated as a mixture of two rotamers. ^{1}H NMR and ^{13}C NMR data listed are for the major rotamer.

^{1}H NMR (400 MHz, Chloroform-*d*) δ 7.33 – 7.25 (m, 2H), 7.19 – 7.11 (m, 1H), 5.31 – 5.28 (m, 1H), 5.09 – 5.04 (m, 1H), 3.24 (s, 3H), 2.09 (s, 3H).

^{13}C NMR (101 MHz, Chloroform-*d*) δ 149.3, 143.0, 141.3, 138.7, 134.6, 130.2, 129.8, 128.5, 117.8, 39.9, 23.1.

Benzyl(2-(prop-1-en-2-yl)phenyl)carbamic chloride (1u).

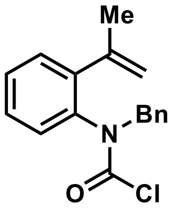

Prepared following general procedure **B**. The crude product was purified by silica gel column chromatography (4% EtOAc in petroleum ether) to provide the title compound as a yellow oil in 52% yield. The product was isolated as a mixture of two rotamers. ^{1}H NMR and ^{13}C NMR data listed are for the major rotamer.

^{1}H NMR (400 MHz, Chloroform-*d*) δ 7.36 – 7.24 (m, 5H), 7.23 – 7.07 (m, 3H), 6.71 (d, *J* = 7.9 Hz, 1H), 5.39 (d, *J* = 14.3 Hz, 1H), 5.34 – 5.25 (m, 1H), 5.14 (s, 1H), 4.10 (d, *J* = 14.3 Hz, 1H), 2.16 (s, 3H).

^{13}C NMR (101 MHz, Chloroform-*d*) δ 150.2, 142.6, 141.1, 138.1, 135.4, 130.9, 129.8, 129.3 (2C), 128.9, 128.6 (2C), 128.2, 127.7, 117.2, 55.6, 23.4.

Alkyl halides **2f**,[3] **2g**,[4] **2h**,[5] **2i**,[6] **2j**,[7] 2l,[7] **2m**,[7] **2n**,[3] **2ab**[8] and **2ad-ah**[9] are known compounds in the literature.

Supplementary Figure 3. General procedure for the preparation of alkyl iodides.

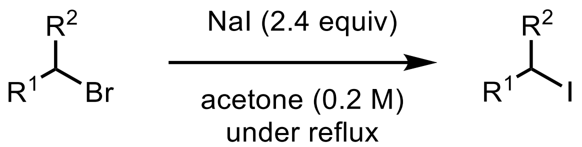

The alkyl bromides (5 mmol，1 equiv) and acetone (25 mL) were added to a 100 mL flask equipped with a magnetic stir bar, successively. After addition of NaI (1.80 g, 12 mmol, 2.4 equiv), the colorless solution turned yellow and a colorless precipitate was formed. The reaction mixture was then refluxed for 12 h. After cooling to room temperature, the mixture was filtered over a silica plug eluting with EtOAc. The solvent was concentrated under reduced pressure and the residue was purified through column chromatography on silica gel (ethyl acetate/petroleum ether) to give the corresponding iodides.

(2-(3-Iodopropoxy)phenyl)methanol (2k).

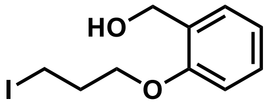

The title compound was isolated as a colorless oil in 78% yield (1.14 g) through column chromatography on silica gel (40% EtOAc in petroleum ether).

^{1}H NMR (400 MHz, Chloroform-*d*) δ 7.34 – 7.26 (m, 2H), 6.97 (td, *J* = 7.5, 1.1 Hz, 1H), 6.91 (dd, *J* = 8.2, 1.0 Hz, 1H), 4.70 (s, 2H), 4.12 (t, *J* = 5.8 Hz, 2H), 3.37 (t, *J* = 6.7 Hz, 2H), 2.37 – 2.26 (m, 2H), 2.13 (s, 1H).

^{13}C NMR (101 MHz, Chloroform-*d*) 156.3, 129.2, 129.0, 128.8, 121.0, 111.2, 67.3, 61.8, 32.7, 2.2.

Phenyl 6-iodohexanoate (2o).

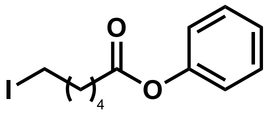

The title compound was isolated as a colorless oil in 68% yield (1.08 g) through column chromatography on silica gel (10% EtOAc in petroleum ether).

^{1}H NMR (400 MHz, Chloroform-*d*) δ 7.38 (t, *J* = 7.9 Hz, 2H), 7.23 (t, *J* = 7.4 Hz, 1H), 7.08 (d, *J* = 7.7 Hz, 2H), 3.22 (t, *J* = 6.9 Hz, 2H), 2.58 (t, *J* = 7.4 Hz, 2H), 1.89 (p, *J* = 7.1 Hz, 2H), 1.78 (p, *J* = 7.5 Hz, 2H), 1.59 – 1.48 (m, 2H).

^{13}C NMR (101 MHz, Chloroform-*d*) δ 172.0, 150.7, 129.5 (2C), 125.8, 121.6 (2C), 34.2, 33.1, 29.9, 23.9, 6.6.

3-Chlorophenyl 6-iodohexanoate (2p).

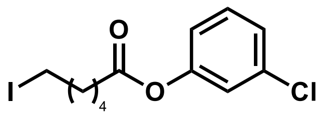

The title compound was isolated as a colorless oil in 65% yield (1.15 g) through column chromatography on silica gel (10% EtOAc in petroleum ether).

^{1}H NMR (500 MHz, Chloroform-*d*) δ 7.31 (t, *J* = 8.1 Hz, 1H), 7.22 (ddd, *J* = 8.1, 2.0, 1.1 Hz, 1H), 7.13 (t, *J* = 2.1 Hz, 1H), 7.00 (ddd, *J* = 8.1, 2.2, 1.0 Hz, 1H), 3.22 (t, *J* = 6.9 Hz, 2H), 2.58 (t, *J* = 7.4 Hz, 2H), 1.95 – 1.83 (m, 2H), 1.83 – 1.72 (m, 2H), 1.61 – 1.49 (m, 2H).

^{13}C NMR (101 MHz, Chloroform-*d*) δ 171.5, 151.1, 134.7, 130.2, 126.1, 122.3, 120.0, 34.0, 33.0, 29.9, 23.9, 6.5.

3-(Dimethylamino)phenyl 6-iodohexanoate (2q).

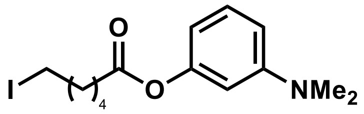

The title compound was isolated as a colorless oil in 88% yield (1.59 g) through column chromatography on silica gel (10% EtOAc in petroleum ether).

^{1}H NMR (400 MHz, Chloroform-*d*) δ 7.23 (t, *J* = 8.2 Hz, 1H), 6.60 (ddd, *J* = 8.4, 2.5, 0.8 Hz, 1H), 6.47 – 6.37 (m, 2H), 3.24 (t, *J* = 6.9 Hz, 2H), 2.97 (s, 6H), 2.59 (t, *J* = 7.5 Hz, 2H), 1.97 – 1.87 (m, 2H), 1.85 – 1.74 (m, 2H), 1.62 – 1.54 (m, 2H).

^{13}C NMR (101 MHz, Chloroform-*d*) δ 172.1, 151.8, 151.7, 129.6, 109.9, 109.2, 105.4, 40.5 (2C), 34.2, 33.1, 30.0, 23.9, 6.6.

4-(Methylthio)phenyl 6-iodohexanoate (2r).

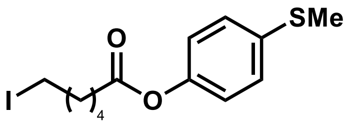

The title compound was isolated as a colorless oil in 84% yield (1.53 g) through column chromatography on silica gel (10% EtOAc in petroleum ether).

^{1}H NMR (400 MHz, Chloroform-*d*) δ 7.30 – 7.24 (m, 2H), 7.05 – 6.98 (m, 2H), 3.21 (t, *J* = 6.9 Hz, 2H), 2.57 (t, *J* = 7.4 Hz, 2H), 2.47 (s, 3H), 1.94 – 1.83 (m, 2H), 1.82 – 1.72 (m, 2H), 1.60 – 1.47 (m, 2H).

^{13}C NMR (101 MHz, Chloroform-*d*) δ 171.9, 148.4, 135.6, 128.0 (2C), 122.0 (2C), 34.1, 33.1, 29.9, 23.8, 16.5, 6.6.

4-Hydroxyphenyl 6-iodohexanoate (2s).

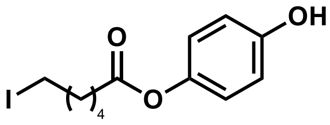

The title compound was isolated as a colorless oil in 57% yield (0.95 g) through column chromatography on silica gel (30% EtOAc in petroleum ether).

^{1}H NMR (500 MHz, Chloroform-*d*) δ 6.97 – 6.86 (m, 2H), 6.80 – 6.68 (m, 2H), 5.21 (s, 1H), 3.21 (t, *J* = 6.9 Hz, 2H), 2.56 (t, *J* = 7.4 Hz, 2H), 1.95 – 1.84 (m, 2H), 1.83 – 1.72 (m, 2H), 1.59 – 1.47 (m, 2H).

^{13}C NMR (126 MHz, Chloroform-*d*) δ 172.8, 153.4, 144.0, 122.4 (2C), 116.0 (2C), 34.1, 33.0, 29.9, 23.8, 6.5.

4-((*tert*-Butyldimethylsilyl)oxy)phenyl 6-iodohexanoate (2t).

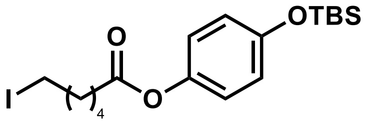

The title compound was isolated as a colorless oil in 72% yield (1.61 g) through column chromatography on silica gel (10% EtOAc in petroleum ether).

^{1}H NMR (500 MHz, Chloroform-*d*) δ 6.82 – 6.72 (m, 2H), 6.70 – 6.61 (m, 2H), 3.06 (t, *J* = 6.9 Hz, 2H), 2.40 (t, *J* = 7.4 Hz, 2H), 1.78 – 1.68 (m, 2H), 1.66 – 1.56 (m, 2H), 1.44 – 1.33 (m, 2H), 0.82 (s, 9H), 0.03 (s, 6H).

^{13}C NMR (126 MHz, Chloroform-*d*) δ 172.2, 153.2, 144.6, 122.2 (2C), 120.5 (2C),

34.1, 33.1, 29.9, 25.7 (3C), 23.9, 18.2, 6.6, −4.5 (2C).

4-(Trifluoromethoxy)phenyl 6-iodohexanoate (2u).

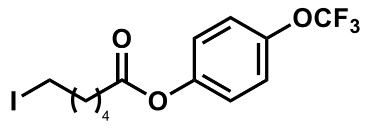

The title compound was isolated as a colorless oil in 82% yield (1.65 g) through column chromatography on silica gel (10% EtOAc in petroleum ether).

^{1}H NMR (500 MHz, Chloroform-*d*) δ 7.23 (d, *J* = 8.5 Hz, 2H), 7.15 – 7.08 (m, 2H), 3.22 (t, *J* = 6.9 Hz, 2H), 2.59 (t, *J* = 7.4 Hz, 2H), 1.94 – 1.84 (m, 2H), 1.83 – 1.74 (m, 2H), 1.59 – 1.48 (m, 2H).

^{13}C NMR (126 MHz, Chloroform-*d*) δ 171.7, 148.9, 146.5, 122.9 (2C), 122.1 (2C), 120.4 (q, *J* = 256.8 Hz), 34.0, 33.0, 29.9, 23.8, 6.5.

^{19}F NMR (376 MHz, Chloroform-*d*) δ −58.13 (s, 3F).

4-(Trifluoromethyl)phenyl 6-iodohexanoate (2v).

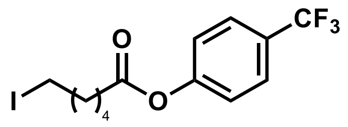

The title compound was isolated as a colorless oil in 68% yield (1.31 g) through column chromatography on silica gel (10% EtOAc in petroleum ether).

^{1}H NMR (500 MHz, Chloroform-*d*) δ 7.69 – 7.60 (m, 2H), 7.25 – 7.18 (m, 2H), 3.22 (t, *J* = 6.9 Hz, 2H), 2.61 (t, *J* = 7.4 Hz, 2H), 1.95 – 1.84 (m, 2H), 1.84 – 1.73 (m, 2H), 1.60 – 1.49 (m, 2H).

^{13}C NMR (101 MHz, Chloroform-*d*) δ 171.4, 153.1, 128.1 (q, *J* = 32.9 Hz), 126.8 (q, *J* = 3.7 Hz, 2C), 123.9 (q, *J* = 271.9 Hz), 122.1 (2C), 34.1, 33.0, 29.9, 23.7, 6.5.

^{19}F NMR (376 MHz, Chloroform-*d*) δ −62.22 (s, 3F).

4-(*tert*-Butyl)phenyl 6-iodohexanoate (2w).

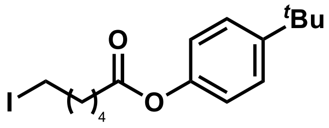

The title compound was isolated as a colorless oil in 78% yield (1.46 g) through column chromatography on silica gel (10% EtOAc in petroleum ether).

^{1}H NMR (400 MHz, Chloroform-*d*) δ 7.42 – 7.34 (m, 2H), 7.04 – 6.97 (m, 2H), 3.22 (t, *J* = 7.0 Hz, 2H), 2.57 (t, *J* = 7.4 Hz, 2H), 1.93 – 1.83 (m, 2H), 1.82 – 1.71 (m, 2H), 1.60 – 1.49 (m, 2H), 1.31 (s, 9H).

^{13}C NMR (101 MHz, Chloroform-*d*) δ 172.1, 148.6, 148.3, 126.3 (2C), 120.8 (2C), 34.5, 34.2, 33.1, 31.4 (3C), 29.9, 23.9, 6.6.

(*E*)-Hex-2-en-1-yl 6-iodohexanoate (2x).

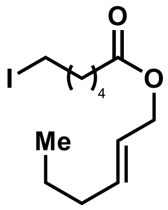

The title compound was isolated as a colorless oil in 64% yield (1.04 g) through column chromatography on silica gel (10% EtOAc in petroleum ether).

^{1}H NMR (500 MHz, Chloroform-*d*) δ 5.77 (dtt, *J* = 14.9, 6.7, 1.3 Hz, 1H), 5.56 (dtt, *J* = 15.6, 6.6, 1.5 Hz, 1H), 4.52 (dd, *J* = 6.6, 0.7 Hz, 2H), 3.19 (t, *J* = 7.0 Hz, 2H), 2.33 (t, *J* = 7.5 Hz, 2H), 2.07 – 1.98 (m, 2H), 1.88 – 1.79 (m, 2H), 1.70 – 1.61 (m, 2H), 1.49 – 1.37 (m, 4H), 0.90 (t, *J* = 7.4 Hz, 3H).

^{13}C NMR (126 MHz, Chloroform-*d*) δ 173.3, 136.5, 123.9, 65.3, 34.3, 34.1, 33.1, 30.0, 23.9, 22.1, 13.7, 6.6.

Supplementary Figure 4. Procedure for the preparation of ligand L12.

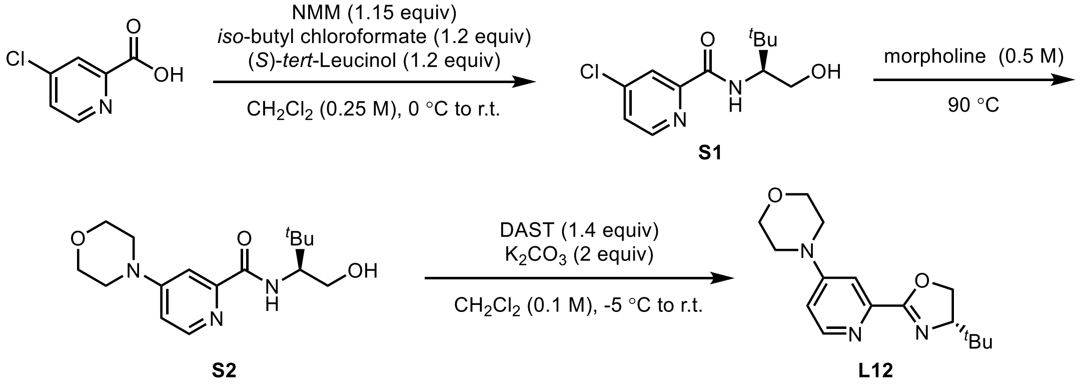

Step 1: The chiral ligand was prepared according to the known procedure with slight modification.[10] To a dry 250 mL round bottom flask containing a stir bar was added 4-chloropicolinic acid (3.94g, 25 mmol, 1 equiv). Under a N_2 atmosphere, CH_2Cl_2 (100 mL) was added via syringe, followed by *N*-methylmorpholine (2.91 g, 28.75 mmol, 1.15 equiv). The reaction mixture was cooled to 0 °C, before *iso*-butyl chloroformate (4.10 g, 30 mmol, 1.2 equiv) was added. The mixture was stirred for 20 min, and then (*S*)-*tert*-Leucinol (3.52 g, 30 mmol, 1.2 equiv) was added. The mixture was allowed to warm to r.t. and stirred overnight. Subsequently, the mixture was quenched with H_2O. The aqueous layer was extracted twice with CH_2Cl_2 and the combined organic phases were dried over Na_2SO_4, filtered and concentrated under reduced pressure. The residue was purified through column chromatography on silica gel (50% EtOAc in petroleum ether) to provide **S1** as a colorless oil in 63% yield (4.04 g).

Step 2: To a 100 mL screw-cap vial equipped with a magnetic stir bar was added **S1** (3.85 g, 15 mmol, 1equiv) and morpholine (15 mL), successively. The reaction mixture was stirred at 90 °C for 12 h. After cooling to room temperature, the reaction was diluted with EtOAc and washed twice with H_2O. The organic phase was then dried over Na_2SO_4, filtered, and concentrated under reduced pressure. The residue was purified through column chromatography on silica gel (60% EtOAc in petroleum ether) to give **S2** as a colorless oil in 82% yield (3.78 g).

Step 3: To a dry 250 mL round-bottom flask containing a stir bar was added **S2** (3.69 g, 12 mmol, 1equiv). Under a N_2 atmosphere, CH_2Cl_2 (120 mL) was added via

syringe. The reaction mixture was cooled to –5 °C, and diethylaminosulfur trifluoride (2.71 g, 16.8 mmol, 1.4 equiv) was added. The reaction mixture was stirred for 2 h at this temperature, before K_2CO_3 (3.32 g, 24 mmol, 2.0 equiv) was added. The mixture was allowed to warm to r.t. and stirred for 3 h. Next, the reaction was quenched with H_2O carefully. The organic layer was washed with saturated aq. $NaHCO_3$ and brine, dried over Na_2SO_4, filtered and concentrated under reduced pressure. The residue was purified through column chromatography on silica gel (70% EtOAc in petroleum ether + 1% NEt_3) to provide **L12** as a colorless syrup in 69% yield (2.40 g).

^{1}H NMR (400 MHz, Chloroform-*d*) δ 8.37 (d, *J* = 5.9 Hz, 1H), 7.50 (d, *J* = 2.7 Hz, 1H), 6.72 (dd, *J* = 5.9, 2.7 Hz, 1H), 4.42 (dd, *J* = 10.3, 8.7 Hz, 1H), 4.29 (t, *J* = 8.5 Hz, 1H), 4.09 (dd, *J* = 10.3, 8.3 Hz, 1H), 3.92 – 3.79 (m, 4H), 3.45 – 3.31 (m, 4H), 0.97 (s, 9H).

^{13}C NMR (101 MHz, Chloroform-*d*) δ 163.1, 155.5, 150.3, 147.6, 109.4, 108.2, 76.4, 69.2, 66.4 (2C), 46.1 (2C), 34.0, 26.0 (3C).

HRMS (ESI) calcd for $C_{16}H_{24}N_3O_2^+$ [$(M+H)^+$] 290.1863, found 290.1859.

Supplementary Table 1. Optimization of reaction conditions for racemic variant of Ni-catalyzed carbo-acylation

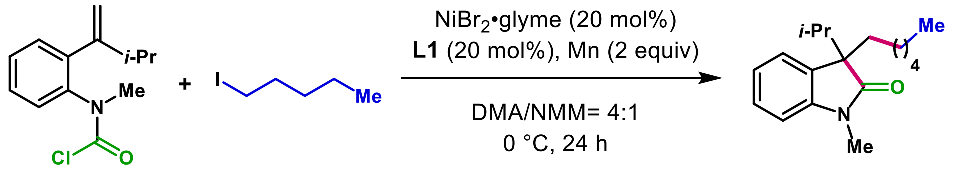

1a 2a 3aa

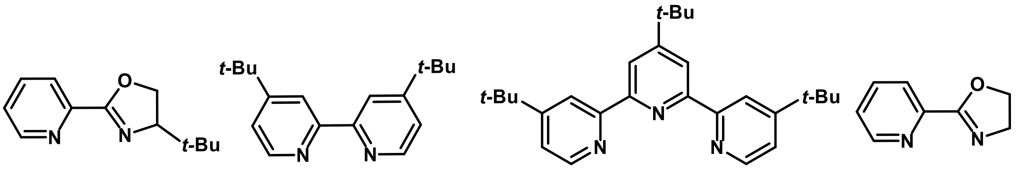

L1 L13 L14 L15

entry	precatalyst	ligand	solvent	reductant	yield (%)
1	$NiBr_2$·glyme	**L1**	DMA/NMM= 4:1	Mn	90 (88[c])
2	$NiBr_2$·glyme	**L13**	DMA/NMM= 4:1	Mn	trace
3	$NiBr_2$·glyme	**L14**	DMA/NMM= 4:1	Mn	0
4	$NiBr_2$·glyme	**L15**	DMA/NMM= 4:1	Mn	42
5	$NiBr_2$	**L1**	DMA/NMM= 4:1	Mn	0
6	$Ni(COD)_2$	**L1**	DMA/NMM= 4:1	Mn	77
7	$Ni(acac)_2$	**L1**	DMA/NMM= 4:1	Mn	0
8	$NiBr_2$·glyme	**L1**	DMA	Mn	65
9	$NiBr_2$·glyme	**L1**	NMM	Mn	0
10	$NiBr_2$·glyme	**L1**	THF	Mn	trace
11	$NiBr_2$·glyme	**L1**	DMF	Mn	0
12	$NiBr_2$·glyme	**L1**	DMSO	Mn	0
13	$NiBr_2$·glyme	**L1**	acetone	Mn	0
14	$NiBr_2$·glyme	-	DMA/NMM= 4:1	Mn	trace
15	$NiBr_2$·glyme	**L1**	DMA/NMM= 4:1	Mn	70
16	$NiBr_2$·glyme	**L1**	DMA/NMM= 4:1	Mn	0

Supplementary Figure 5. General procedure for racemic variant of the Ni-catalyzed carbo-acylation

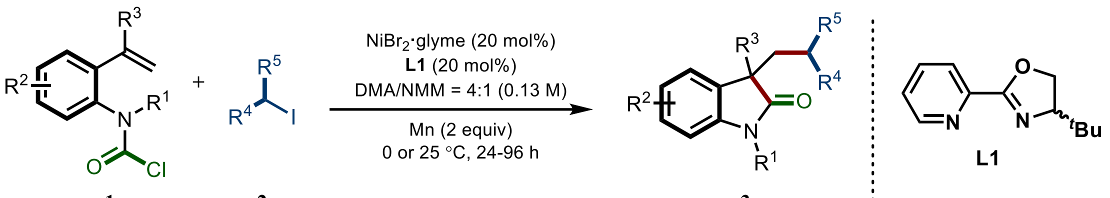

Racemic 4-(*tert*-Butyl)-2-(pyridin-2-yl)-4,5-dihydrooxazole (**L1**) (8.2 mg, 0.04 mmol, 20 mol%), carbamoyl chlorides **1** (if solid, 0.2 mmol, 1.0 equiv) and alkyl iodides **2**[a] (if solid, 0.4 mmol, 2.0 equiv) were added to a reaction tube equipped with a stir bar. In a nitrogen-filled glovebox, $NiBr_2$·glyme (12.3 mg, 0.04 mmol, 20 mol%) and manganese dust (22 mg, 0.4 mmol, 2 equiv) were added to the mixture. The reaction tube was sealed and removed from the glovebox. Next, anhydrous DMA (1.2 mL) and *N*-methyl morpholine (0.3 mL) were added, followed by the addition of carbamoyl chlorides **1** (if liquid, 0.2 mmol, 1 equiv) and alkyl iodies **2**[a] (if liquid, 0.4 mmol, 2.0 equiv) under the protection of nitrogen. Then the resulting mixture was stirred at the temperature specified below[b] for 24-96 h[c]. The reaction was quenched with sat. aq. NH_4Cl solution (5 mL) and diluted with water (10 mL). The aqueous layer was extracted three times with EtOAc, and the combined organic layers were washed with brine (20 mL), dried over $MgSO_4$, filtered, and concentrated under reduced pressure. The residue was purified through column chromatography on silica gel (petroleum ether/ethyl acetate) to afford the desired product **3**.

[a] Benzyl chlorides were used for **3aac-3aah**,

[b] 25 °C for **3ak**, **3ap**, **3aq**, **3as**, **3av-ax** and **3aac-aah**, 0 °C for **3aa-pa**, **3ab-aj, 3al-ao, 3ar, 3at, 3au** and **3ay-aab**,

[c] Reaction time: 24 h for **3aa-ea**, **3ga**, **3ja**, **3la**, **3ab-ah**, **3am**, **3ay-aaa**, 48 h for **3ha**, **3ia**, **3ka**, **3ma**, **3na**, **3ai-al**, **3an-ax** and **3aab-aah**, 96 h for **3fa**, **3oa** and **3pa.**

3-Hexyl-3-isopropyl-1-methylindolin-2-one (3aa).

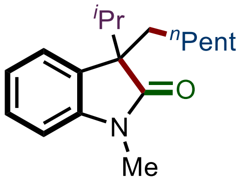

The title compound was isolated as a pale yellow oil in 88% yield (48.0 mg) starting from the carbamoyl chloride **1a** (47.5 mg) through column chromatography on silica gel (10% EtOAc in petroleum ether). For 1-mmol-scale reaction, the product was obtained in 85% yield (232.2 mg) starting the carbamoyl chloride **1a** (237.7 mg).

^{1}H NMR (400 MHz, Chloroform-*d*) δ 7.31 – 7.23 (m, 1H), 7.16 (ddd, *J* = 7.4, 1.3, 0.6 Hz, 1H), 7.05 (td, *J* = 7.5, 1.0 Hz, 1H), 6.85 – 6.79 (m, 1H), 3.20 (s, 3H), 2.23 – 2.07 (m, 1H), 1.93 – 1.79 (m, 2H), 1.22 – 1.06 (m, 6H), 0.97 (d, *J* = 7.0 Hz, 3H), 0.94 – 0.85 (m, 1H), 0.80 (t, *J* = 7.0 Hz, 3H), 0.73 – 0.62 (m, 1H), 0.67 (d, *J* = 6.8 Hz, 3H).

^{13}C NMR (101 MHz, Chloroform-*d*) δ 180.3, 144.4, 131.5, 127.5, 123.5, 122.1, 107.5, 56.5, 35.5, 35.3, 31.6, 29.6, 25.8, 24.4, 22.6, 17.4, 17.3, 14.0.

HRMS (ESI) calcd for $C_{18}H_{28}NO^+$ [$(M+H)^+$] 274.2165, found 274.2166.

3-Hexyl-1,3-dimethylindolin-2-one (3ba).

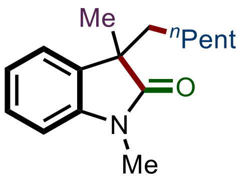

The title compound was isolated as a pale yellow oil in 53% yield (26.1 mg) starting from the carbamoyl chloride **1b** (41.9 mg) through column chromatography on silica gel (10% EtOAc in petroleum ether).

^{1}H NMR (400 MHz, Chloroform-*d*) δ 7.26 (t, *J* = 7.7 Hz, 1H), 7.17 (d, *J* = 7.3 Hz, 1H), 7.06 (t, *J* = 7.5 Hz, 1H), 6.84 (d, *J* = 7.7 Hz, 1H), 3.21 (s, 3H), 1.95 – 1.85 (m, 1H), 1.76 – 1.68 (m, 1H), 1.35 (s, 3H), 1.24 – 1.08 (m, 6H), 1.03 – 0.93 (m, 1H), 0.87 – 0.73 (m, 1H), 0.80 (t, *J* = 6.9 Hz, 3H).

^{13}C NMR (101 MHz, Chloroform-*d*) δ 180.9, 143.3, 134.3, 127.6, 122.5, 122.4, 107.9, 48.5, 38.6, 31.5, 29.4, 26.1, 24.4, 23.8, 22.6, 14.0.

HRMS (ESI) calcd for $C_{16}H_{24}NO^+$ [$(M+H)^+$] 246.1852, found 246.1852.

3-Hexyl-3-isopropyl-1-methylindolin-2-one (3ca).

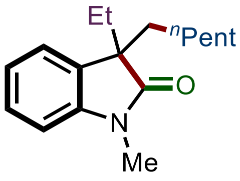

The title compound was isolated as a pale yellow oil in 80% yield

(41.6 mg) starting from the carbamoyl chloride **1c** (44.7 mg) through column chromatography on silica gel (10% EtOAc in petroleum ether).

^{1}H NMR (400 MHz, Chloroform-*d*) δ 7.30 – 7.23 (m, 1H), 7.13 (d, *J* = 7.2 Hz, 1H), 7.07 (t, *J* = 7.4 Hz, 1H), 6.83 (d, *J* = 7.8 Hz, 1H), 3.21 (s, 3H), 1.96 – 1.83 (m, 2H), 1.82 – 1.66 (m, 2H), 1.22 – 1.07 (m, 6H), 1.00 – 0.90 (m, 1H), 0.85 – 0.72 (m, 1H), 0.80 (t, *J* = 6.9 Hz, 3H), 0.55 (t, *J* = 7.3 Hz, 3H).

^{13}C NMR (101 MHz, Chloroform-*d*) δ 180.2, 144.2, 132.4, 127.5, 122.6, 122.4, 107.7, 53.8, 37.8, 31.5, 31.0, 29.5, 26.0, 24.2, 22.6, 14.0, 8.6.

HRMS (ESI) calcd for $C_{17}H_{26}NO^+$ [$(M+H)^+$] 260.2009, found 260.2009.

3-Hexyl-1-methyl-3-propylindolin-2-one (3da).

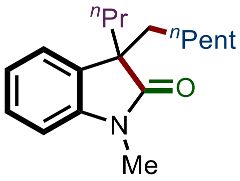

The title compound was isolated as a pale yellow oil in 74% yield (40.5 mg) starting from the carbamoyl chloride **1d** (47.5 mg) through column chromatography on silica gel (10% EtOAc in petroleum ether).

^{1}H NMR (400 MHz, Chloroform-*d*) δ 7.26 (t, *J* = 7.6 Hz, 1H), 7.14 (d, *J* = 7.2 Hz, 1H), 7.07 (t, *J* = 7.4 Hz, 1H), 6.83 (d, *J* = 7.7 Hz, 1H), 3.20 (s, 3H), 1.91 – 1.81 (m, 2H), 1.77 – 1.66 (m, 2H), 1.23 – 1.08 (m, 6H), 1.03 – 0.91 (m, 2H), 0.87 – 0.74 (m, 2H), 0.80 (t, *J* = 6.8 Hz, 3H), 0.76 (t, *J* = 6.4 Hz, 3H).

^{13}C NMR (101 MHz, Chloroform-*d*) δ 180.4, 144.0, 132.8, 127.5, 122.6, 122.3, 107.7, 53.3, 40.4, 38.1, 31.5, 29.5, 26.0, 24.1, 22.6, 17.5, 14.2, 14.0.

HRMS (ESI) calcd for $C_{18}H_{28}NO^+$ [$(M+H)^+$] 274.2165, found 274.2165.

3-Cyclohexyl-3-hexyl-1-methylindolin-2-one (3ea).

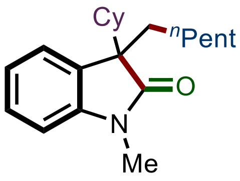

The title compound was isolated as a pale yellow oil in 78% yield (49.0 mg) starting from the carbamoyl chloride **1e** (55.4 mg) through column chromatography on silica gel (10% EtOAc in petroleum ether).

^{1}H NMR (400 MHz, Chloroform-*d*) δ 7.28 – 7.23 (m, 1H), 7.16 (dd, *J* = 7.4, 1.2 Hz, 1H), 7.05 (td, *J* = 7.5, 1.0 Hz, 1H), 6.81 (dd, *J* = 7.8, 0.9 Hz, 1H), 3.19 (s, 3H), 1.89 – 1.69 (m, 5H), 1.63 – 1.53 (m, 2H), 1.48 – 1.41 (m, 1H), 1.25 – 1.06 (m, 9H), 1.05 – 0.86 (m, 2H), 0.79 (t, J = 7.0 Hz, 3H), 0.77 – 0.62 (m, 2H).

^{13}C NMR (101 MHz, Chloroform-*d*) δ 180.4, 144.3, 132.1, 127.3, 123.5, 122.0, 107.5, 56.6, 45.4, 34.8, 31.5, 29.6, 27.4, 27.2, 26.7, 26.4, 26.3, 25.8, 24.1, 22.6, 14.0.

HRMS (ESI) calcd for $C_{21}H_{32}NO^+$ $[(M+H)^+]$ 314.2478, found 314.2479.

3-Hexyl-1-methyl-3-phenylindolin-2-one (3fa).

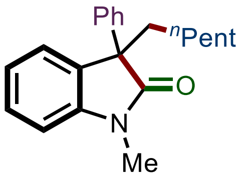

The title compound was isolated as a pale yellow oil in 61% yield (37.5 mg) starting from the carbamoyl chloride **1f** (54.3 mg) through column chromatography on silica gel (10% EtOAc in petroleum ether).

^{1}H NMR (400 MHz, Chloroform-*d*) δ 7.40 – 7.18 (m, 7H), 7.11 (td, *J* = 7.5, 1.1 Hz, 1H), 6.90 (d, *J* = 7.8 Hz, 1H), 3.22 (s, 3 H), 2.36 (td, *J* = 12.7, 4.4 Hz, 1H), 2.18 (td, *J* = 12.8, 4.2 Hz, 1H), 1.29 – 1.07 (m, 7H), 0.92 – 0.79 (m, 1H), 0.81 (t, *J* = 6.8 Hz, 3H).

^{13}C NMR (101 MHz, Chloroform-*d*) δ 178.7, 144.0, 140.4, 132.4, 128.5 (2C), 128.1, 127.2, 126.9 (2C), 124.8, 122.6, 108.2, 56.8, 38.0, 31.5, 29.5, 26.4, 24.5, 22.6, 14.1.

HRMS (ESI) calcd for $C_{21}H_{26}NO^+$ $[(M+H)^+]$ 308.2009, found 308.2008.

3-Hexyl-3-isopropyl-1,6-dimethylindolin-2-one (3ga).

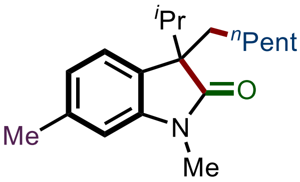

The title compound was isolated as a pale yellow oil in 62% yield (35.6 mg) starting from the carbamoyl chloride **1g** (50.3 mg) through column chromatography on silica gel (10% EtOAc in petroleum ether).

^{1}H NMR (400 MHz, Chloroform-*d*) δ 7.03 (d, *J* = 7.5 Hz, 1H), 6.85 (dd, *J* = 7.5, 1.5 Hz, 1H), 6.65 (d, *J* = 1.4 Hz, 1H), 3.18 (s, 3H), 2.39 (s, 3H), 2.12 (hept, *J* = 6.9 Hz,

1H), 1.89 – 1.78 (m, 2H), 1.23 – 1.06 (m, 6H), 0.96 (d, *J* = 7.0 Hz, 3H), 0.94 – 0.86 (m, 1H), 0.80 (t, *J* = 7.0 Hz, 3H), 0.75 – 0.68 (m, 1H), 0.66 (d, *J* = 6.7 Hz, 3H).

^{13}C NMR (101 MHz, Chloroform-*d*) δ 180.6, 144.4, 137.4, 128.3, 123.2, 122.6, 108.5, 56.2, 35.5, 35.3, 31.6, 29.6, 25.8, 24.4, 22.6, 21.8, 17.5, 17.3, 14.0.

HRMS (ESI) calcd for $C_{19}H_{30}NO^+$ [$(M+H)^+$] 288.2322, found 288.2322.

3-Hexyl-3-isopropyl-6-methoxy-1-methylindolin-2-one (3ha).

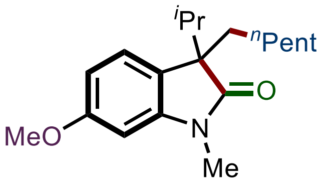

The title compound was isolated as a pale yellow oil in 83% yield (50.3 mg) starting from the carbamoyl chloride **1h** (53.6 mg) through column chromatography on silica gel (10% EtOAc in petroleum ether).

^{1}H NMR (400 MHz, Chloroform-*d*) δ 7.04 (d, *J* = 8.1 Hz, 1H), 6.55 (dd, *J* = 8.1, 2.3 Hz, 1H), 6.42 (d, *J* = 2.4 Hz, 1H), 3.84 (s, 3H), 3.17 (s, 3H), 2.15 – 2.05 (m, 1H), 1.89 – 1.76 (m, 2H), 1.23 – 1.06 (m, 6H), 0.96 (d, *J* = 6.9 Hz, 3H), 0.93 – 0.85 (m, 1H), 0.80 (t, *J* = 7.0 Hz, 3H), 0.76 – 0.68 (m, 1H), 0.65 (d, *J* = 6.7 Hz, 3H).

^{13}C NMR (101 MHz, Chloroform-*d*) δ 180.9, 159.7, 145.6, 124.0, 123.2, 105.7, 95.6, 56.0, 55.4, 35.6, 35.3, 31.6, 29.6, 25.8, 24.4, 22.6, 17.5, 17.3, 14.0.

HRMS (ESI) calcd for $C_{19}H_{30}NO_2^+$ [$(M+H)^+$] 304.2271, found 304.2270.

6-(Benzyloxy)-3-hexyl-3-isopropyl-1-methylindolin-2-one (3ia).

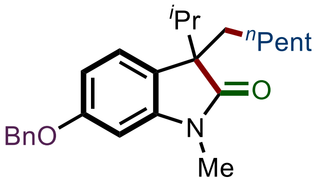

The title compound was isolated as a pale yellow oil in 85% yield (64.4 mg) starting from the carbamoyl chloride **1i** (68.8 mg) through column chromatography on silica gel (10% EtOAc in petroleum ether).

^{1}H NMR (400 MHz, Chloroform-*d*) δ 7.48 – 7.32 (m, 5H), 7.04 (d, *J* = 8.1 Hz, 1H), 6.63 (dd, *J* = 8.2, 2.3 Hz, 1H), 6.51 (d, *J* = 2.3 Hz, 1H), 5.07 (s, 2H), 3.16 (s, 3H), 2.16 – 2.05 (m, 1H), 1.90 – 1.76 (m, 2H), 1.23 – 1.08 (m, 6H), 0.97 (d, *J* = 6.9 Hz, 3H), 0.93 – 0.86 (m, 1H), 0.80 (t, *J* = 7.0 Hz, 3H), 0.76 – 0.68 (m, 1H), 0.66 (d, *J* = 6.7 Hz, 3H).

^{13}C NMR (101 MHz, Chloroform-*d*) δ 180.9, 159.0, 145.6, 136.9, 128.6 (2C), 128.1, 127.6 (2C), 124.0, 123.6, 106.6, 96.5, 70.3, 56.0, 35.6, 35.3, 31.6, 29.6, 25.8, 24.4, 22.6, 17.5, 17.3, 14.0.

HRMS (ESI) calcd for $C_{25}H_{34}NO_2^+$ [$(M+H)^+$] 380.2584, found 380.2584.

6-Chloro-3-hexyl-3-isopropyl-1-methylindolin-2-one (3ja).

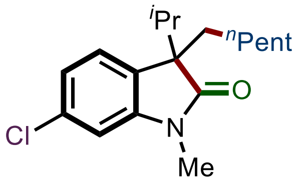

The title compound was isolated as a pale yellow oil in 65% yield (40.1 mg) starting from the carbamoyl chloride **1j** (54.4 mg) through column chromatography on silica gel (10% EtOAc in petroleum ether).

^{1}H NMR (400 MHz, Chloroform-*d*) δ 7.07 (d, *J* = 7.9 Hz, 1H), 7.02 (dd, *J* = 7.9, 1.8 Hz, 1H), 6.82 (d, *J* = 1.8 Hz, 1H), 3.18 (s, 3H), 2.18 – 2.07 (m, 1H), 1.94 – 1.76 (m, 2H), 1.22 – 1.07 (m, 6H), 0.95 (d, *J* = 6.9 Hz, 3H), 0.92 – 0.85 (m, 1H), 0.80 (t, *J* = 7.0 Hz, 3H), 0.74 – 0.62 (m, 1H), 0.67 (d, *J* = 6.7 Hz, 3H).

^{13}C NMR (101 MHz, Chloroform-*d*) δ 180.2, 145.6, 133.3, 129.8, 124.3, 121.9, 108.3, 56.3, 35.4, 35.3, 31.5, 29.5, 25.9, 24.4, 22.6, 17.4, 17.3, 14.0.

HRMS (ESI) calcd for $C_{18}H_{27}ClNO^+$ [$(M+H)^+$] 308.1776, found 308.1776.

3-Hexyl-3-isopropyl-1-methyl-6-(trifluoromethyl)indolin-2-one (3ka).

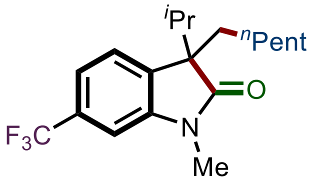

The title compound was isolated as a pale yellow oil in 77% yield (52.5 mg) starting from the carbamoyl chloride **1k** (61.1 mg) through column chromatography on silica gel (10% EtOAc in petroleum ether).

^{1}H NMR (400 MHz, Chloroform-*d*) δ 7.29 – 7.24 (m, 1H), 7.21 – 7.16 (m, 1H), 6.95 (d, *J* = 1.5 Hz, 1H), 3.16 (s, 3H), 2.14 – 2.05 (m, 1H), 1.89 – 1.74 (m, 2H), 1.14 – 1.00 (m, 6H), 0.89 (d, *J* = 6.9 Hz, 3H), 0.86 – 0.78 (m, 1H), 0.73 (t, *J* = 7.0 Hz, 3H), 0.62 (d, *J* = 6.8 Hz, 3H), 0.60 – 0.51 (m, 1H).

^{13}C NMR (101 MHz, Chloroform-*d*) δ 178.8, 143.9, 134.6, 129.0 (q, *J* = 32.4 Hz),

123.1 (q, *J* = 272.1 Hz), 122.5, 118.1 (q, *J* = 4.1 Hz), 103.2 (q, *J* = 3.7 Hz), 55.6, 34.4, 34.3, 30.5, 28.5, 24.9, 23.3, 21.5, 16.3, 16.2, 12.9.

^{19}F NMR (471 MHz, Chloroform-*d*) δ −62.28 (s, 3F)**.**

HRMS (ESI) calcd for $C_{19}H_{26}F_3NONa^+$ [$(M+Na)^+$] 364.1859, found 364.1860.

5-Chloro-3-hexyl-3-isopropyl-1-methylindolin-2-one (3la).

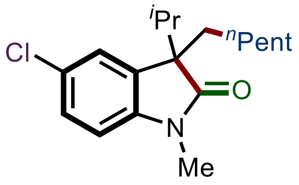

The title compound was isolated as a pale yellow oil in 64% yield (39.5 mg) starting from the carbamoyl chloride **1l** (54.4 mg) through column chromatography on silica gel (10% EtOAc in petroleum ether).

^{1}H NMR (400 MHz, Chloroform-*d*) δ 7.25 (dd, *J* = 8.2, 2.1 Hz, 1H), 7.14 (d, *J* = 2.1 Hz, 1H), 6.75 (d, *J* = 8.2 Hz, 1H), 3.18 (s, 3H), 2.13 (hept, *J* = 6.9 Hz, 1H), 1.93 – 1.76 (m, 2H), 1.22 – 1.09 (m, 6H), 0.96 (d, *J* = 6.9 Hz, 3H), 0.93 – 0.86 (m, 1H), 0.81 (t, *J* = 7.0 Hz, 3H), 0.75 – 0.64 (m, 1H), 0.69 (d, *J* = 6.8 Hz, 3H).

^{13}C NMR (101 MHz, Chloroform-*d*) δ 179.7, 142.9, 133.4, 127.5, 127.4, 123.9, 108.4, 56.8, 35.4, 35.4, 31.5, 29.6, 25.9, 24.4, 22.6, 17.4, 17.2, 14.0.

HRMS (ESI) calcd for $C_{18}H_{27}ClNO^+$ [$(M+H)^+$] 308.1776, found 308.1776.

3-Hexyl-1,3-dimethyl-6-(trifluoromethyl)indolin-2-one (3ma).

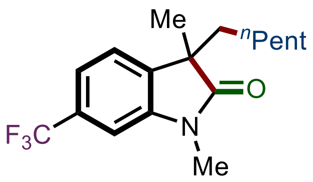

The title compound was isolated as a pale yellow oil in 51% yield (31.7 mg) starting from the carbamoyl chloride **1m** (55.4 mg) through column chromatography on silica gel (10% EtOAc in petroleum ether).

^{1}H NMR (400 MHz, Chloroform-*d*) δ 7.35 (d, *J* = 7.7 Hz, 1H), 7.26 (d, *J* = 7.5 Hz, 1H), 7.04 (s, 1H), 3.25 (s, 3H), 1.92 (td, *J* = 12.8, 4.6 Hz, 1H), 1.74 (td, *J* = 12.9, 4.4 Hz, 1H), 1.37 (s, 3H), 1.24 – 1.09 (m, 6H), 1.02 – 0.91 (m, 1H), 0.86 – 0.75 (m, 1H), 0.81 (t, *J* = 6.9 Hz, 3H).

^{13}C NMR (101 MHz, Chloroform-*d*) δ 180.5, 143.9, 138.2, 130.2 (q, *J* = 32.3 Hz),

124.1 (q, J = 272.6 Hz), 122.6, 119.5 (q, J = 4.1 Hz), 104.6 (q, J = 3.9 Hz), 48.6, 38.4, 31.5, 29.3, 26.3, 24.4, 23.6, 22.5, 14.0.

^{19}F NMR (471 MHz, Chloroform-*d*) δ −62.26 (s, 3F)**.**

HRMS (ESI) calcd for $C_{17}H_{23}F_3NO^+$ $[(M+H)^+]$ 314.1726, found 314.1732.

3-Hexyl-4-methoxy-1,3-dimethylindolin-2-one (3na).

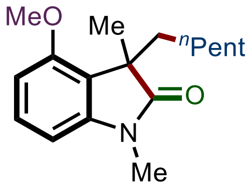

The title compound was isolated as a pale yellow oil in 91% yield (49.8 mg) starting from the carbamoyl chloride **1n** (47.9 mg) through column chromatography on silica gel (10% EtOAc in petroleum ether).

^{1}H NMR (400 MHz, Chloroform-*d*) δ 7.22 (t, J = 8.1 Hz, 1H), 6.62 (d, J = 8.5 Hz, 1H), 6.50 (d, J = 7.8 Hz, 1H), 3.84 (s, 3H), 3.19 (s, 3H), 2.13 – 2.03 (m, 1H), 1.88 – 1.78 (m, 1H), 1.40 (s, 3H), 1.21 – 1.09 (m, 6H), 0.92 – 0.84 (m, 1H), 0.80 (t, J = 7.0 Hz, 3H), 0.77 – 0.67 (m, 1H).

^{13}C NMR (101 MHz, Chloroform-*d*) δ 181.3, 156.0, 144.5, 128.7, 119.4, 105.7, 101.3, 55.3, 49.2, 36.1, 31.5, 29.3, 26.3, 24.9, 22.5, 22.0, 14.0.

HRMS (ESI) calcd for $C_{17}H_{26}NO_2^+$ $[(M+H)^+]$ 276.1958, found 276.1959.

1-Benzyl-3-hexyl-3-isopropylindolin-2-one (3oa).

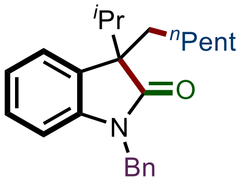

The title compound was isolated as a pale yellow oil in 45% yield (31.5 mg) starting from the carbamoyl chloride **1o** (62.8 mg) through column chromatography on silica gel (10% EtOAc in petroleum ether).

^{1}H NMR (400 MHz, Chloroform-*d*) δ 7.35 – 7.22 (m, 5H), 7.20 – 7.11 (m, 2H), 7.01 (t, J = 7.6 Hz, 1H), 6.72 (d, J = 7.7 Hz, 1H), 4.99 – 4.83 (m, 2H), 2.25 – 2.14 (m, 1H), 1.98 – 1.82 (m, 2H), 1.23 – 1.06 (m, 6H), 1.04 – 0.93 (m, 1H), 0.99 (d, J = 7.0 Hz, 3H), 0.81 (t, J = 6.9 Hz, 3H), 0.77 – 0.64 (m, 1H), 0.74 (d, J = 6.7 Hz, 3H).

^{13}C NMR (101 MHz, Chloroform-*d*) δ 180.3, 143.5, 136.3, 131.6, 128.6 (2C),

127.49 (2C), 127.47, 127.38, 123.5, 122.1, 108.6, 56.3, 43.6, 35.9, 35.5, 31.6, 29.6, 24.5, 22.5, 17.6, 17.5, 14.1.

HRMS (ESI) calcd for $C_{24}H_{32}NO^+$ [(M+H)$^+$] 350.2478, found 350.2478.

3-Hexyl-3-isopropyl-1-(4-methoxybenzyl)indolin-2-one (3pa).

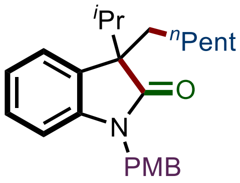

The title compound was isolated as a pale yellow oil in 51% yield (38.8 mg) starting from the carbamoyl chloride **1p** (68.8 mg) through column chromatography on silica gel (10% EtOAc in petroleum ether).

^{1}H NMR (500 MHz, Chloroform-*d*) δ 7.25 (d, *J* = 8.0 Hz, 2H), 7.18 – 7.12 (m, 2H), 7.00 (t, *J* = 7.5 Hz, 1H), 6.82 (d, *J* = 8.2 Hz, 2H), 6.75 (d, *J* = 7.9 Hz, 1H), 4.91 – 4.79 (m, 2H), 3.77 (s, 3H), 2.24 – 2.13 (m, 1H), 1.97 – 1.81 (m, 2H), 1.22 – 1.06 (m, 6H), 1.02 – 0.92 (m, 1H), 0.98 (d, *J* = 7.0 Hz, 3H), 0.80 (t, *J* = 7.0 Hz, 3H), 0.75 – 0.59 (m, 1H), 0.72 (d, *J* = 6.8 Hz, 3H),.

^{13}C NMR (126 MHz, Chloroform-*d*) δ 180.3, 159.0, 143.6, 131.6, 128.9 (2C), 128.5, 127.4, 123.5, 122.0, 114.0 (2C), 108.6, 56.2, 55.2, 43.1, 35.8, 35.5, 31.6, 29.6, 24.5, 22.5, 17.6, 17.4, 14.0.

HRMS (ESI) calcd for $C_{25}H_{34}NO_2^+$ [(M+H)$^+$] 380.2584, found 380.2584.

3-Isopropyl-1-methyl-3-propylindolin-2-one (3ab).

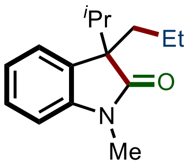

The title compound was isolated as a pale yellow oil in 67% yield (31.1 mg) starting from the carbamoyl chloride **1a** (47.5 mg) through column chromatography on silica gel (10% EtOAc in petroleum ether).

^{1}H NMR (400 MHz, Chloroform-*d*) δ 7.26 (td, *J* = 7.7, 1.3 Hz, 1H), 7.17 (ddd, *J* = 7.4, 1.3, 0.5 Hz, 1H), 7.05 (td, *J* = 7.5, 1.1 Hz, 1H), 6.82 (dt, *J* = 7.8, 0.7 Hz, 1H), 3.20 (s, 3H), 2.20 – 2.08 (m, 1H), 1.93 – 1.78 (m, 2H), 1.02 – 0.89 (m, 1H), 0.97 (d, *J* = 6.9 Hz, 3H), 0.77 (t, *J* = 6.9 Hz, 3H), 0.75 – 0.64 (m, 1H), 0.68 (d, *J* = 6.8 Hz, 3H).

^{13}C NMR (101 MHz, Chloroform-*d*) δ 180.3, 144.3, 131.5, 127.5, 123.5, 122.0, 107.5, 56.5, 37.7, 35.3, 25.8, 17.8, 17.4, 17.3, 14.3.

HRMS (ESI) calcd for $C_{15}H_{22}NO^+$ [(M+H)$^+$] 232.1696, found 232.1696.

3-Isopropyl-1-methyl-3-nonylindolin-2-one (3ac).

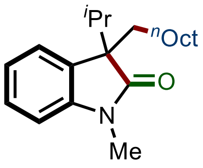

The title compound was isolated as a pale yellow oil in 75% yield (47.2 mg) starting from the carbamoyl chloride **1a** (47.5 mg) through column chromatography on silica gel (10% EtOAc in petroleum ether).

^{1}H NMR (400 MHz, Chloroform-*d*) δ 7.26 (td, *J* = 7.6, 1.3 Hz, 1H), 7.16 (dd, *J* = 7.4, 1.2 Hz, 1H), 7.04 (td, *J* = 7.5, 1.0 Hz, 1H), 6.82 (d, *J* = 7.7 Hz, 1H), 3.20 (s, 3H), 2.14 (hept, *J* = 6.9 Hz, 1H), 1.93 – 1.79 (m, 2H), 1.28 – 1.06 (m, 12H), 0.99 – 0.87 (m, 1H), 0.97 (d, *J* = 6.9 Hz, 3H), 0.85 (t, *J* = 7.0 Hz, 3H), 0.75 – 0.62 (m, 1H), 0.68 (d, *J* = 6.7 Hz, 3H).

^{13}C NMR (101 MHz, Chloroform-*d*) δ 180.3, 144.3, 131.5, 127.5, 123.5, 122.1, 107.5, 56.4, 35.5, 35.3, 31.8, 30.0, 29.5, 29.4, 29.2, 25.8, 24.5, 22.7, 17.4, 17.3, 14.1.

HRMS (ESI) calcd for $C_{21}H_{34}NO^+$ [(M+H)$^+$] 316.2635, found 316.2635.

3-(4-Chlorobutyl)-3-isopropyl-1-methylindolin-2-one (3ad).

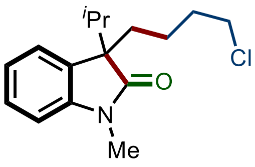

The title compound was isolated as a pale yellow oil in 68% yield (38.1 mg) starting from the carbamoyl chloride **1a** (47.5 mg) through column chromatography on silica gel (10% EtOAc in petroleum ether).

^{1}H NMR (400 MHz, Chloroform-*d*) δ 7.28 (td, *J* = 7.7, 1.3 Hz, 1H), 7.17 (ddd, *J* = 7.4, 1.4, 0.6 Hz, 1H), 7.06 (td, *J* = 7.5, 1.0 Hz, 1H), 6.84 (d, *J* = 7.7 Hz, 1H), 3.44 – 3.32 (m, 2H), 3.21 (s, 3H), 2.15 (hept, *J* = 6.9 Hz, 1H), 1.97 – 1.82 (m, 2H), 1.73 – 1.55 (m, 2H), 1.09 – 1.00 (m, 1H), 0.97 (d, *J* = 7.0 Hz, 3H), 0.93 – 0.83 (m, 1H), 0.68 (d, *J* = 6.7 Hz, 3H).

^{13}C NMR (101 MHz, Chloroform-*d*) δ 179.9, 144.3, 130.9, 127.7, 123.5, 122.2, 107.7, 56.2, 44.5, 35.3, 34.6, 32.8, 25.8, 21.9, 17.4, 17.2.

HRMS (ESI) calcd for $C_{16}H_{23}ClNO^+$ [$(M+H)^+$] 280.1463, found 280.1472.

3-(4-Fluorobutyl)-3-isopropyl-1-methylindolin-2-one (3ae).

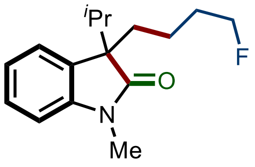

The title compound was isolated as a pale yellow oil in 71% yield (37.3 mg) starting from the carbamoyl chloride **1a** (47.5 mg) through column chromatography on silica gel (10% EtOAc in petroleum ether).

^{1}H NMR (400 MHz, Chloroform-*d*) δ 7.28 (td, *J* = 7.6, 1.3 Hz, 1H), 7.17 (dd, *J* = 7.5, 1.2 Hz, 1H), 7.06 (td, *J* = 7.5, 1.0 Hz, 1H), 6.83 (d, *J* = 7.7 Hz, 1H), 4.41 – 4.17 (m, 2H), 3.20 (s, 3H), 2.15 (hept, *J* = 7.0 Hz, 1H), 1.98 – 1.85 (m, 2H), 1.70 – 1.47 (m, 2H), 1.09 – 0.93 (m, 1H), 0.97 (d, *J* = 7.0 Hz, 3H) 0.90 – 0.79 (m, 1H), 0.68 (d, *J* = 6.8 Hz, 3H).

^{13}C NMR (101 MHz, Chloroform-*d*) δ 180.0, 144.3, 131.0, 127.7, 123.5, 122.2, 107.7, 83.8 (d, *J* = 164.6 Hz), 56.3, 35.4, 35.1, 30.6 (d, *J* = 19.6 Hz), 25.9, 20.4 (d, *J* = 5.5 Hz), 17.4, 17.3.

^{19}F NMR (471 MHz, Chloroform-*d*) δ −223.09 (s, 1F)**.**

HRMS (ESI) calcd for $C_{16}H_{23}FNO^+$ [$(M+H)^+$] 264.1758, found 264.1766.

6-(3-Isopropyl-1-methyl-2-oxoindolin-3-yl)hexanenitrile (3af).

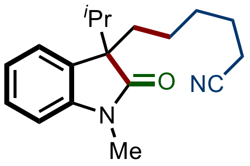

The title compound was isolated as a pale yellow oil in 92% yield (52.3 mg) starting from the carbamoyl chloride **1a** (47.5 mg) through column chromatography on silica gel (20% EtOAc in petroleum ether).

^{1}H NMR (400 MHz, Chloroform-*d*) δ 7.25 – 7.17 (m, 1H), 7.09 (dd, *J* = 7.4, 1.2 Hz, 1H), 6.99 (td, *J* = 7.5, 1.0 Hz, 1H), 6.77 (dd, *J* = 7.8, 0.9 Hz, 1H), 3.13 (s, 3H), 2.14 (t, *J* = 7.1 Hz, 2H), 2.11 – 2.00 (m, 1H), 1.88 – 1.74 (m, 2H), 1.50 – 1.39 (m, 2H), 1.31 – 1.20 (m, 2H), 0.93 – 0.82 (m, 1H), 0.90 (d, *J* = 7.0 Hz, 3H), 0.73 – 0.62 (m, 1H), 0.60

(d, J = 6.8 Hz, 3H).

^{13}C NMR (101 MHz, Chloroform-*d*) δ 180.0, 144.3, 131.0, 127.7, 123.4, 122.2, 119.6, 107.7, 56.3, 35.4, 34.9, 28.8, 25.8, 25.0, 23.6, 17.4, 17.2, 17.0.

HRMS (ESI) calcd for $C_{18}H_{25}N_2O^+$ [(M+H)$^+$] 285.1961, found 285.1966.

3-(3-(1,3-Dioxolan-2-yl)propyl)-3-isopropyl-1-methylindolin-2-one (3ag).

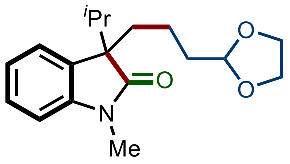

The title compound was isolated as a pale yellow oil in 73% yield (44.4 mg) starting from the carbamoyl chloride **1a** (47.5 mg) through column chromatography on silica gel (15% EtOAc in petroleum ether).

^{1}H NMR (500 MHz, Chloroform-*d*) δ 7.26 (td, J = 7.7, 1.3 Hz, 1H), 7.16 (dd, J = 7.5, 1.2 Hz, 1H), 7.04 (td, J = 7.5, 1.0 Hz, 1H), 6.84 – 6.79 (m, 1H), 4.68 (t, J = 4.9 Hz, 1H), 3.93 – 3.82 (m, 2H), 3.80 – 3.71 (m, 2H), 3.19 (s, 3H), 2.20 – 2.10 (m, 1H), 1.98 – 1.87 (m, 2H), 1.63 – 1.53 (m, 1H), 1.53 – 1.44 (m, 1H), 1.12 – 1.01 (m, 1H), 0.96 (d, J = 6.9 Hz, 3H), 0.93 – 0.80 (m, 1H), 0.68 (d, J = 6.7 Hz, 3H).

^{13}C NMR (126 MHz, Chloroform-*d*) δ 178.0, 144.3, 131.1, 127.6, 123.5, 122.1, 107.6, 104.4, 64.8, 64.7, 56.3, 35.4 (2C), 34.1, 25.9, 19.3, 17.4, 17.3.

HRMS (ESI) calcd for $C_{18}H_{26}NO_3^+$ [(M+H)$^+$] 304.1907, found 304.1909.

3-Isopropyl-3-(4-(4-methoxyphenoxy)butyl)-1-methylindolin-2-one (3ah).

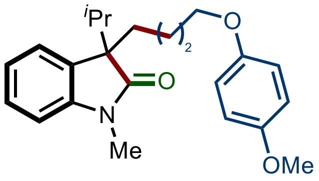

The title compound was isolated as a pale yellow oil in 96% yield (71.0 mg) starting from the carbamoyl chloride **1a** (47.5 mg) through column chromatography on silica gel (10% EtOAc in petroleum ether).

^{1}H NMR (400 MHz, Chloroform-*d*) δ 7.27 (t, J = 7.8 Hz, 1H), 7.16 (d, J = 7.3 Hz, 1H), 7.05 (t, J = 7.5 Hz, 1H), 6.82 (d, J = 7.8 Hz, 1H), 6.81 – 6.69 (m, 4H), 3.82 – 3.68 (m, 2H), 3.74 (s, 3H), 3.20 (s, 3H), 2.20 – 2.10 (m, 1H), 2.00 – 1.86 (m, 2H), 1.73 – 1.54 (m, 2H), 1.15 – 1.03 (m, 1H), 0.97 (d, J = 6.9 Hz, 3H), 0.94 – 0.81 (m,

1H), 0.68 (d, J = 6.7 Hz, 3H).

^{13}C NMR (101 MHz, Chloroform-*d*) δ 180.1, 153.6, 153.1, 144.3, 131.1, 127.6, 123.5, 122.2, 115.4 (2C), 114.5 (2C), 107.7, 68.2, 56.4, 55.7, 35.4, 35.2, 29.6, 25.9, 21.1, 17.4, 17.3.

HRMS (ESI) calcd for $C_{23}H_{29}NO_3Na^+$ [(M+Na)$^+$] 390.2040, found 390.2045.

3-Isopropyl-1-methyl-3-(4-(4-(methylsulfonyl)phenoxy)butyl)indolin-2-one (3ai).

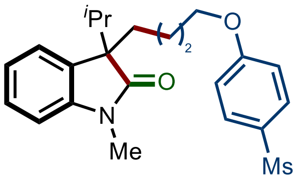

The title compound was isolated as a pale yellow oil in 88% yield (73.3 mg) starting from the carbamoyl chloride **1a** (47.5 mg) through column chromatography on silica gel (50% EtOAc in petroleum ether).

^{1}H NMR (400 MHz, Chloroform-*d*) δ 7.81 (dd, J = 8.8, 1.9 Hz, 2H), 7.28 (ddd, J = 8.0, 5.8, 1.7 Hz, 1H), 7.17 (d, J = 7.4 Hz, 1H), 7.06 (t, J = 7.5 Hz, 1H), 6.91 (dd, J = 8.8, 1.9 Hz, 2H), 6.84 (d, J = 7.8 Hz, 1H), 3.88 (t, J = 6.7 Hz, 2H), 3.21 (s, 3H), 3.01 (s, 3H), 2.23 – 2.07 (m, 1H), 2.05 – 1.87 (m, 2H), 1.79 – 1.61 (m, 2H), 1.16 – 1.04 (m, 1H), 0.98 (d, J = 6.9 Hz, 3H), 0.94 – 0.80 (m, 1H), 0.68 (d, J = 6.7 Hz, 3H).

^{13}C NMR (101 MHz, Chloroform-*d*) δ 180.0, 163.1, 144.3, 131.9, 131.0, 129.5 (2C), 127.7, 123.5, 122.2, 114.9 (2C), 107.7, 68.1, 56.3, 44.9, 35.4, 35.0, 29.2, 25.9, 21.0, 17.4, 17.3.

HRMS (ESI) calcd for $C_{23}H_{30}NO_4S^+$ [(M+H)$^+$] 416.1890, found 416.1892.

3-Isopropyl-1-methyl-3-(4-(4-(4,4,5,5-tetramethyl-1,3,2-dioxaborolan-2-yl)phenoxy)butyl)indolin-2-one (3aj).

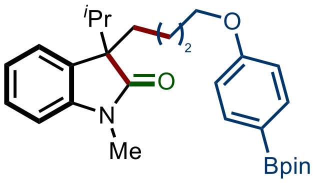

The title compound was isolated as a pale yellow oil in 62% yield (57.1 mg) starting from the carbamoyl chloride **1a** (47.5 mg) through column chromatography on silica gel (20% EtOAc in petroleum ether).

^{1}H NMR (400 MHz, Chloroform-*d*) δ 7.73 – 7.66 (m, 2H), 7.27 (td, J = 7.7, 1.3 Hz,

1H), 7.16 (ddd, J = 7.4, 1.3, 0.6 Hz, 1H), 7.05 (td, J = 7.5, 1.0 Hz, 1H), 6.82 (dt, J = 7.7, 0.7 Hz, 1H), 6.80 – 6.74 (m, 2H), 3.90 – 3.76 (m, 2H), 3.20 (s, 3H), 2.21 – 2.10 (m, 1H), 2.01 – 1.88 (m, 2H), 1.74 – 1.57 (m, 2H), 1.32 (s, 12H), 1.13 – 1.03 (m, 1H), 0.97 (d, J = 6.9 Hz, 3H), 0.92 – 0.83 (m, 1H), 0.68 (d, J = 6.7 Hz, 3H).

^{13}C NMR (101 MHz, Chloroform-*d*) δ 180.0, 161.5, 144.3, 136.4 (2C), 131.1, 127.6, 123.5, 122.2, 113.8 (2C), 107.6, 83.5 (2C), 67.3, 56.4, 35.4, 35.1, 29.4, 25.8, 24.9 (4C), 21.1, 17.4, 17.3.

HRMS (ESI) calcd for $C_{28}H_{38}BNO_4Na^+$ [$(M+Na)^+$] 486.2786, found 486.2793.

3-(4-(2-(Hydroxymethyl)phenoxy)butyl)-3-isopropyl-1-methylindolin-2-one (3ak).

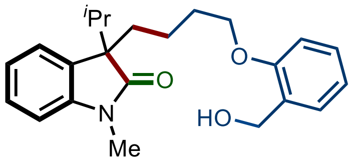

The title compound was isolated as a pale yellow oil in 78% yield (57.4 mg) starting from the carbamoyl chloride **1a** (47.5 mg) through column chromatography on silica gel (60% EtOAc in petroleum ether).

^{1}H NMR (400 MHz, Chloroform-*d*) δ 7.31 – 7.13 (m, 4H), 7.05 (t, J = 7.5 Hz, 1H), 6.89 (t, J = 7.4 Hz, 1H), 6.83 (d, J = 7.7 Hz, 1H), 6.76 (d, J = 8.2 Hz, 1H), 4.55 (s, 2H), 3.93 – 3.80 (m, 2H), 3.19 (s, 3H), 2.61 – 2.32 (brs, 1H), 2.20 – 2.08 (m, 1H), 2.05 – 1.85 (m, 2H), 1.75 – 1.59 (m, 2H), 1.16 – 1.03 (m, 1H), 0.98 (d, J = 6.9 Hz, 3H), 0.96 – 0.83 (m, 1H), 0.68 (d, J = 6.7 Hz, 3H).

^{13}C NMR (101 MHz, Chloroform-*d*) δ 180.2, 156.7, 144.3, 131.1, 129.3, 128.8 (2C), 127.7, 123.5, 122.3 120.5, 111.1, 107.8, 67.3, 61.8, 56.5, 35.4, 35.0, 29.3, 25.9, 21.1, 17.4, 17.3.

HRMS (ESI) calcd for $C_{23}H_{29}NO_3Na^+$ [$(M+Na)^+$] 390.2040, found 390.2046.

4-(4-(3-Isopropyl-1-methyl-2-oxoindolin-3-yl)butoxy)benzaldehyde (3al).

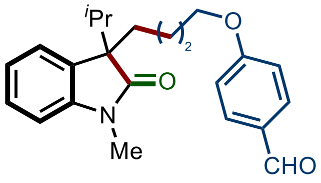

The title compound was isolated as a pale yellow oil in 92% yield (67.4 mg) starting from the carbamoyl chloride **1a**

(47.5 mg) through column chromatography on silica gel (30% EtOAc in petroleum ether).

^{1}H NMR (400 MHz, Chloroform-*d*) δ 9.86 (s, 1H), 7.79 (d, *J* = 8.4 Hz, 2H), 7.33 – 7.24 (m, 1H), 7.17 (d, *J* = 7.3 Hz, 1H), 7.09 – 7.02 (m, 1H), 6.89 (d, *J* = 8.3 Hz, 2H), 6.84 (d, *J* = 7.7 Hz, 1H), 3.96 – 3.83 (m, 2H), 3.20 (s, 3H), 2.20 – 2.10 (m, 1H), 2.04 – 1.88 (m, 2H), 1.76 – 1.60 (m, 2H), 1.18 – 1.03 (m, 1H), 0.98 (d, *J* = 6.9 Hz, 3H), 0.94 – 0.85 (m, 1H), 0.69 (d, *J* = 6.7 Hz, 3H).

^{13}C NMR (101 MHz, Chloroform-*d*) δ 190.8, 180.0, 164.1, 144.3, 132.0 (2C), 131.0, 129.7, 127.7, 123.5, 122.2, 114.7 (2C), 107.7, 68.0, 56.4, 35.4, 35.1, 29.3, 25.9, 21.1, 17.4, 17.3.

HRMS (ESI) calcd for $C_{23}H_{28}NO_3^+$ [$(M+H)^+$] 366.2064, found 366.2081.

3-(6-(4-Acetylphenoxy)hexyl)-3-isopropyl-1-methylindolin-2-one (3am).

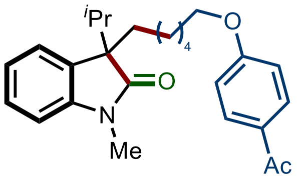

The title compound was isolated as a pale yellow oil in 88% yield (71.5 mg) starting from the carbamoyl chloride **1a** (47.5 mg) through column chromatography on silica gel (20% EtOAc in petroleum ether).

^{1}H NMR (400 MHz, Chloroform-*d*) δ 7.96 – 7.86 (m, 2H), 7.31 – 7.23 (m, 1H), 7.16 (dd, *J* = 7.4, 1.2 Hz, 1H), 7.05 (td, *J* = 7.5, 1.0 Hz, 1H), 6.90 – 6.80 (m, 3H), 3.93 (t, *J* = 6.5 Hz, 2H), 3.20 (s, 3H), 2.55 (s, 3H), 2.17 – 2.09 (m, 1H), 1.95 – 1.80 (m, 2H), 1.71 – 1.64 (m, 2H), 1.33 – 1.23 (m, 4H), 1.02 – 0.90 (m, 1H), 0.97 (d, *J* = 7.0 Hz, 3H), 0.80 – 0.70 (m, 1H), 0.67 (d, *J* = 6.7 Hz, 3H).

^{13}C NMR (101 MHz, Chloroform-*d*) δ 196.8, 180.2, 163.0, 144.3, 131.3, 130.6 (2C), 130.1, 127.5, 123.4, 122.1, 114.1 (2C), 107.6, 68.1, 56.4, 35.4, 35.3, 29.6, 29.0, 26.3, 25.8, 25.7, 24.3, 17.4, 17.3.

HRMS (ESI) calcd for $C_{26}H_{33}NO_3Na^+$ [$(M+Na)^+$] 430.2353, found 430.2359.

2-(5-(3-Isopropyl-1-methyl-2-oxoindolin-3-yl)pentyl)isoindoline-1,3-dione (3an).

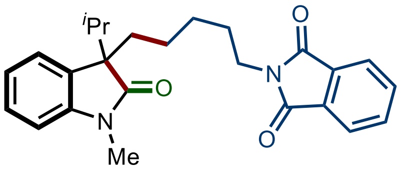

The title compound was isolated as a pale yellow oil in 90% yield (72.6 mg) starting from the carbamoyl chloride **1a** (47.5 mg) through column chromatography on silica gel (40% EtOAc in petroleum ether).

^{1}H NMR (500 MHz, Chloroform-*d*) δ 7.82 (dd, *J* = 5.4, 3.0 Hz, 2H), 7.70 (dd, *J* = 5.5, 3.0 Hz, 2H), 7.25 (td, *J* = 7.7, 1.2 Hz, 1H), 7.15 (dd, *J* = 7.3, 1.2 Hz, 1H), 7.03 (td, *J* = 7.5, 1.0 Hz, 1H), 6.81 (d, *J* = 7.8 Hz, 1H), 3.56 (t, *J* = 7.1 Hz, 2H), 3.19 (s, 3H), 2.17 – 2.07 (m, 1H), 1.92 – 1.80 (m, 2H), 1.57 – 1.47 (m, 2H), 1.29 – 1.17 (m, 2H), 1.00 – 0.90 (m, 1H), 0.95 (d, *J* = 7.0 Hz, 3H), 0.78 – 0.69 (m, 1H), 0.66 (d, *J* = 6.8 Hz, 3H).

^{13}C NMR (126 MHz, Chloroform-*d*) δ 180.1, 168.4 (2C), 144.3, 133.8 (2C), 132.1 (2C), 131.2, 127.6, 123.5, 123.1 (2C), 122.1, 107.6, 56.3, 38.0, 35.3, 35.2, 28.3, 27.1, 25.8, 24.1, 17.4, 17.3.

HRMS (ESI) calcd for $C_{25}H_{29}N_2O_3^+$ [$(M+H)^+$] 405.2173, found 405.2184.

Phenyl 7-(3-isopropyl-1-methyl-2-oxoindolin-3-yl)heptanoate (3ao).

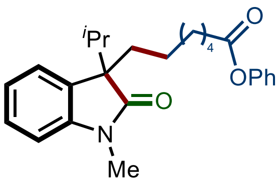

The title compound was isolated as a pale yellow oil in 94% yield (73.8 mg) starting from the carbamoyl chloride **1a** (47.5 mg) through column chromatography on silica gel (15% EtOAc in petroleum ether).

^{1}H NMR (400 MHz, Chloroform-*d*) δ 7.39 – 7.31 (m, 2H), 7.29 – 7.24 (m, 1H), 7.23 – 7.14 (m, 2H), 7.08 – 7.01 (m, 3H), 6.82 (dt, *J* = 7.7, 0.7 Hz, 1H), 3.19 (s, 3H), 2.46 (t, *J* = 7.5 Hz, 2H), 2.14 (hept, *J* = 6.8 Hz, 1H), 1.95 – 1.81 (m, 2H), 1.67 – 1.57 (m, 2H), 1.32 – 1.16 (m, 4H), 1.03 – 0.90 (m, 1H), 0.97 (d, *J* = 6.9 Hz, 3H), 0.79 – 0.65 (m, 1H), 0.68 (d, *J* = 6.8 Hz, 3H).

^{13}C NMR (101 MHz, Chloroform-*d*) δ 180.2, 172.2, 150.7, 144.4, 131.3, 129.4 (2C), 127.6, 125.7, 123.5, 122.1, 121.6 (2C), 107.6, 56.4, 35.4, 35.3, 34.3, 29.6, 28.8, 25.8, 24.8, 24.3, 17.4, 17.3.

HRMS (ESI) calcd for $C_{25}H_{32}NO_3^+$ [(M+H)$^+$] 394.2377, found 394.2383.

3-Chlorophenyl 7-(3-isopropyl-1-methyl-2-oxoindolin-3-yl)heptanoate (3ap).

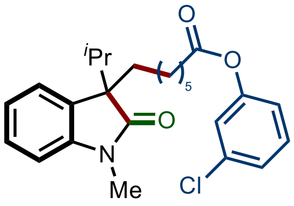

The title compound was isolated as a pale yellow oil in 76% yield (65.1 mg) starting from the carbamoyl chloride **1a** (47.5 mg) through column chromatography on silica gel (15% EtOAc in petroleum ether).

^{1}H NMR (500 MHz, Chloroform-*d*) δ 7.32 – 7.25 (m, 2H), 7.22 – 7.15 (m, 2H), 7.09 (t, J = 2.1 Hz, 1H), 7.05 (td, J = 7.5, 1.0 Hz, 1H), 6.96 (ddd, J = 8.1, 2.2, 1.0 Hz, 1H), 6.83 (d, J = 7.7 Hz, 1H), 3.20 (s, 3H), 2.46 (t, J = 7.5 Hz, 2H), 2.18 – 2.10 (m, 1H), 1.95 – 1.82 (m, 2H), 1.65 – 1.58 (m, 2H), 1.30 – 1.15 (m, 4H), 1.01 – 0.91 (m, 1H), 0.97 (d, J = 6.9 Hz, 3H), 0.78 – 0.69 (m, 1H), 0.68 (d, J = 6.7 Hz, 3H).

^{13}C NMR (126 MHz, Chloroform-*d*) δ 180.2, 171.8, 151.2, 144.4, 134.6, 131.3, 130.1, 127.6, 126.0, 123.5, 122.3, 122.1, 120.0, 107.6, 56.4, 35.4, 35.3, 34.2, 29.5, 28.8, 25.8, 24.7, 24.3, 17.4, 17.3.

HRMS (ESI) calcd for $C_{25}H_{31}ClNO_3^+$ [(M+H)$^+$] 428.1987, found 428.1996.

3-(Dimethylamino)phenyl 7-(3-isopropyl-1-methyl-2-oxoindolin-3-yl)heptanoate (3aq).

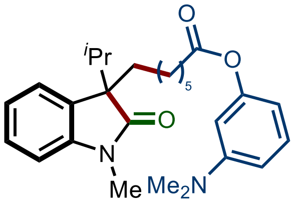

The title compound was isolated as a pale yellow oil in 79% yield (69.0 mg) starting from the carbamoyl chloride **1a** (47.5 mg) through column chromatography on silica gel (15% EtOAc in petroleum ether).

^{1}H NMR (400 MHz, Chloroform-*d*) δ 7.26 (t, J = 4.2 Hz, 1H), 7.22 – 7.14 (m, 2H), 7.05 (t, J = 7.5 Hz, 1H), 6.82 (d, J = 7.7 Hz, 1H), 6.56 (dd, J = 8.4, 2.3 Hz, 1H), 6.42 – 6.31 (m, 2H), 3.20 (s, 3H), 2.93 (s, 6H), 2.45 (t, J = 7.5 Hz, 2H), 2.18 – 2.10 (m, 1H), 1.95 – 1.81 (m, 2H), 1.65 – 1.56 (m, 2H), 1.30 – 1.16 (m, 4H), 0.96 – 0.89 (m, 1H), 0.97 (d, J = 6.9 Hz, 3H), 0.78 – 0.64 (m, 1H), 0.67 (d, J = 6.7 Hz, 3H).

^{13}C NMR (101 MHz, Chloroform-*d*) δ 180.2, 172.4, 151.8, 151.6, 144.3, 131.3,

129.6, 127.5, 123.5, 122.1, 109.8, 109.3, 107.6, 105.4, 56.4, 40.5 (2C), 35.36, 35.35, 34.4, 29.6, 28.9, 25.8, 24.9, 24.3, 17.4, 17.3.

HRMS (ESI) calcd for $C_{27}H_{37}N_2O_3^+$ [$(M+H)^+$] 437.2799, found 437.2798.

4-(Methylthio)phenyl 7-(3-isopropyl-1-methyl-2-oxoindolin-3-yl)heptanoate (3ar).

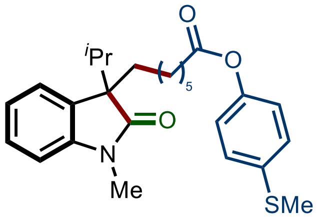

The title compound was isolated as a pale yellow oil in 93% yield (81.9 mg) starting from the carbamoyl chloride **1a** (47.5 mg) through column chromatography on silica gel (15% EtOAc in petroleum ether).

^{1}H NMR (400 MHz, Chloroform-*d*) δ 7.26 (dd, *J* = 8.7, 2.8 Hz, 3H), 7.16 (d, *J* = 7.3 Hz, 1H), 7.05 (t, *J* = 7.4 Hz, 1H), 6.98 (d, *J* = 8.4 Hz, 2H), 6.83 (d, *J* = 7.7 Hz, 1H), 3.20 (s, 3H), 2.46 (d, *J* = 6.3 Hz, 5H), 2.19 – 2.10 (m, 1H), 1.94 – 1.80 (m, 2H), 1.66 – 1.56 (m, 2H), 1.29 – 1.18 (m, 4H), 1.01 – 0.88 (m, 1H), 0.97 (d, *J* = 6.9 Hz, 3H), 0.78 – 0.64 (m, 1H), 0.67 (d, *J* = 6.7 Hz, 3H).

^{13}C NMR (101 MHz, Chloroform-*d*) δ 180.2, 172.2, 148.4, 144.3, 135.5, 131.3, 128.0 (2C), 127.6, 123.5, 122.11, 122.06 (2C), 107.6, 56.4, 35.4, 35.3, 34.2, 29.5, 28.8, 25.8, 24.8, 24.3, 17.4, 17.3, 16.5.

HRMS (ESI) calcd for $C_{26}H_{34}NO_3S^+$ [$(M+H)^+$] 440.2254, found 440.2261.

4-Hydroxyphenyl 7-(3-isopropyl-1-methyl-2-oxoindolin-3-yl)heptanoate (3as).

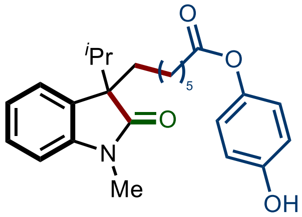

The title compound was isolated as a pale yellow oil in 64% yield (52.4 mg) starting from the carbamoyl chloride **1a** (47.5 mg) through column chromatography on silica gel (40% EtOAc in petroleum ether).

^{1}H NMR (400 MHz, Chloroform-*d*) δ 7.32 – 7.23 (m, 1H), 7.17 (d, *J* = 7.4 Hz, 1H), 7.07 (t, *J* = 7.6 Hz, 1H), 6.92 – 6.73 (m, 5H), 6.55 – 6.24 (brs, 1H), 3.22 (s, 3H), 2.42 (t, *J* = 7.6 Hz, 2H), 2.20 – 2.10 (m, 1H), 1.96 – 1.81 (m, 2H), 1.65 – 1.52 (m, 2H), 1.27 – 1.14 (m, 4H), 1.02 – 0.86 (m, 1H), 0.98 (d, *J* = 6.9 Hz, 3H), 0.77 – 0.60 (m,

1H), 0.66 (d, J = 6.7 Hz, 3H).

^{13}C NMR (101 MHz, Chloroform-*d*) δ 180.8, 172.9, 153.8, 144.2, 143.8, 131.3, 127.6, 123.5, 122.4, 122.3 (2C), 116.0 (2C), 107.8, 56.7, 35.4, 35.3, 34.2, 29.5, 28.8, 26.0, 24.8, 24.3, 17.4, 17.3.

HRMS (ESI) calcd for $C_{25}H_{31}NO_4Na^+$ [$(M+Na)^+$] 432.2145, found 432.2147.

4-((*tert*-Butyldimethylsilyl)oxy)phenyl 7-(3-isopropyl-1-methyl-2-oxoindolin-3-yl)heptanoate (3at).

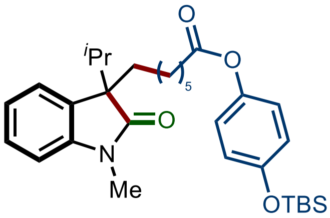

The title compound was isolated as a pale yellow oil in 86% yield (90.1 mg) starting from the carbamoyl chloride **1a** (47.5 mg) through column chromatography on silica gel (15% EtOAc in petroleum ether).

^{1}H NMR (500 MHz, Chloroform-*d*) δ 7.31 – 7.23 (m, 1H), 7.16 (d, J = 7.3 Hz, 1H), 7.05 (t, J = 7.5 Hz, 1H), 6.89 (d, J = 8.4 Hz, 2H), 6.82 (d, J = 7.8 Hz, 1H), 6.79 (d, J = 8.4 Hz, 2H), 3.20 (s, 3H), 2.43 (t, J = 7.5 Hz, 2H), 2.20 – 2.08 (m, 1H), 1.96 – 1.79 (m, 2H), 1.66 – 1.55 (m, 2H), 1.29 – 1.15 (m, 4H), 1.02 – 0.81 (m, 1H), 0.97 (s, 9H), 0.96 (d, J = 6.9 Hz, 3H), 0.78 – 0.65 (m, 1H), 0.67 (d, J = 6.7 Hz, 3H), 0.18 (s, 6H).

^{13}C NMR (126 MHz, Chloroform-*d*) δ 180.2, 172.5, 153.1, 144.7, 144.3, 131.3, 127.5, 123.5, 122.2 (2C), 122.1, 120.5 (2C), 107.6, 56.4, 35.4, 35.3, 34.2, 29.6, 28.8, 25.8, 25.7 (3C), 24.9, 24.3, 18.2, 17.4, 17.3, −4.5 (2C).

HRMS (ESI) calcd for $C_{31}H_{46}NO_4Si^+$ [$(M+H)^+$] 524.3191, found 524.3200.

4-(Trifluoromethoxy)phenyl 7-(3-isopropyl-1-methyl-2-oxoindolin-3-yl)heptanoate (3au).

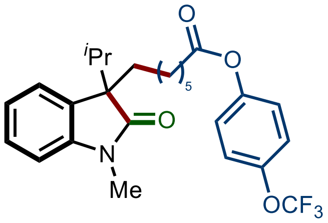

The title compound was isolated as a colorless oil in 72% yield (69.1 mg) starting from the carbamoyl chloride **1a** (47.5 mg) through column chromatography on silica gel (15% EtOAc in petroleum ether).

^{1}H NMR (500 MHz, Chloroform-*d*) δ 7.27 (td, *J* = 7.7, 1.3 Hz, 1H), 7.24 – 7.18 (m, 2H), 7.16 (dd, *J* = 7.4, 1.3 Hz, 1H), 7.11 – 7.01 (m, 3H), 6.85 – 6.80 (m, 1H), 3.20 (s, 3H), 2.47 (t, *J* = 7.5 Hz, 2H), 2.19 – 2.10 (m, 1H), 1.95 – 1.81 (m, 2H), 1.67 – 1.57 (m, 2H), 1.30 – 1.16 (m, 4H), 1.02 – 0.90 (m, 1H), 0.97 (d, *J* = 6.9 Hz, 3H), 0.79 – 0.70 (m, 1H), 0.68 (d, *J* = 6.8 Hz, 3H).

^{13}C NMR (126 MHz, Chloroform-*d*) δ 180.2, 171.9, 148.9, 146.4 (q, *J* = 1.6 Hz), 144.4, 131.3, 127.6, 123.5, 122.9 (2C), 122.12, 122.07 (2C), 120.4 (q, *J* = 258.3 Hz), 107.6, 56.4, 35.4, 35.3, 34.2, 29.5, 28.8, 25.8, 24.7, 24.3, 17.4, 17.3.

HRMS (ESI) calcd for $C_{26}H_{31}F_3NO_4^+$ [(M+H)$^+$] 478.2200, found 478.2208.

^{19}F NMR (376 MHz, Chloroform-*d*) δ −58.12 (s, 3F).

4-(Trifluoromethyl)phenyl 7-(3-isopropyl-1-methyl-2-oxoindolin-3-yl)heptanoate (3av).

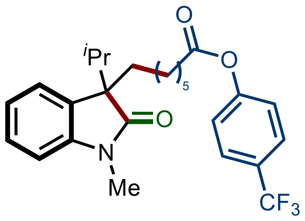

The title compound was isolated as a pale yellow oil in 78% yield (72.0 mg) starting from the carbamoyl chloride **1a** (47.5 mg) through column chromatography on silica gel (15% EtOAc in petroleum ether).

^{1}H NMR (500 MHz, Chloroform-*d*) δ 7.63 (d, *J* = 8.6 Hz, 2H), 7.27 (td, *J* = 7.7, 1.2 Hz, 1H), 7.22 – 7.13 (m, 3H), 7.05 (td, *J* = 7.5, 1.0 Hz, 1H), 6.83 (d, *J* = 7.8 Hz, 1H), 3.20 (s, 3H), 2.49 (t, *J* = 7.5 Hz, 2H), 2.14 (hept, *J* = 6.8 Hz, 1H), 1.96 – 1.80 (m, 2H), 1.67 – 1.59 (m, 2H), 1.28 – 1.19 (m, 4H), 1.01 – 0.90 (m, 1H), 0.97 (d, *J* = 6.9 Hz, 3H), 0.79 – 0.69 (m, 1H), 0.68 (d, *J* = 6.7 Hz, 3H).

^{13}C NMR (126 MHz, Chloroform-*d*) δ 180.2, 171.7, 153.2, 144.4, 131.3, 128.0 (q, *J* = 33.1 Hz), 127.6, 126.8 (q, *J* = 3.6 Hz, 2C), 123.9 (q, *J* = 272.0 Hz), 123.5, 122.12, 122.09 (2C), 107.6, 56.4, 35.4, 35.3, 34.2, 29.5, 28.8, 25.8, 24.7, 24.3, 17.4, 17.3.

HRMS (ESI) calcd for $C_{26}H_{31}F_3NO_3^+$ [(M+H)$^+$] 462.2251, found 462.2261.

^{19}F NMR (376 MHz, Chloroform-*d*) δ −62.21 (s, 3F).

4-(*tert*-Butyl)phenyl 7-(3-isopropyl-1-methyl-2-oxoindolin-3-yl)heptanoate (3aw).

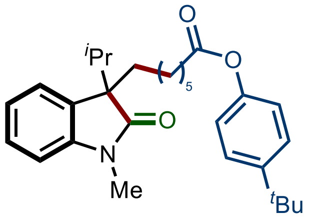

The title compound was isolated as a pale yellow oil in 80% yield (71.7 mg) starting from the carbamoyl chloride **1a** (47.5 mg) through column chromatography on silica gel (10% EtOAc in petroleum ether).

^{1}H NMR (400 MHz, Chloroform-*d*) δ 7.36 (d, *J* = 8.5 Hz, 2H), 7.27 (td, *J* = 7.7, 1.2 Hz, 1H), 7.16 (d, *J* = 7.3 Hz, 1H), 7.09 – 7.01 (m, 1H), 6.96 (d, *J* = 8.5 Hz, 2H), 6.82 (d, *J* = 7.7 Hz, 1H), 3.20 (s, 3H), 2.45 (t, *J* = 7.5 Hz, 2H), 2.20 – 2.09 (m, 1H), 1.95 – 1.80 (m, 2H), 1.65 – 1.56 (m, 2H), 1.31 (s, 9H), 1.27 – 1.15 (m, 4H), 1.00 – 0.86 (m, 1H), 0.97 (d, *J* = 6.9 Hz, 3H), 0.77 – 0.62 (m, 1H), 0.68 (d, *J* = 6.7 Hz, 3H).

^{13}C NMR (101 MHz, Chloroform-*d*) δ 180.2, 172.4, 148.5, 148.3, 144.3, 131.3, 127.6, 126.3 (2C), 123.5, 122.1, 120.9 (2C), 107.6, 56.4, 35.4, 35.3, 34.5, 34.3, 31.4 (3C), 29.6, 28.8, 25.9, 24.9, 24.3, 17.4, 17.3.

HRMS (ESI) calcd for $C_{29}H_{40}NO_3^+$ [(M+H)$^+$] 450.3003, found 450.3012.

(*E*)-Hex-2-en-1-yl 7-(3-isopropyl-1-methyl-2-oxoindolin-3-yl)heptanoate (3ax).

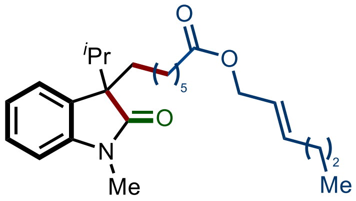

The title compound was isolated as a pale yellow oil in 92% yield (73.5 mg) starting from the carbamoyl chloride **1a** (47.5 mg) through column chromatography on silica gel (15% EtOAc in petroleum ether).

^{1}H NMR (500 MHz, Chloroform-*d*) δ 7.31 – 7.23 (m, 1H), 7.15 (dd, *J* = 7.3, 1.2 Hz, 1H), 7.05 (td, *J* = 7.5, 1.0 Hz, 1H), 6.85 – 6.78 (m, 1H), 5.74 (dtt, *J* = 14.8, 6.7, 1.2 Hz, 1H), 5.54 (dtt, *J* = 15.6, 6.5, 1.5 Hz, 1H), 4.48 (dd, *J* = 6.5, 1.1 Hz, 2H), 3.20 (s, 3H), 2.22 (t, *J* = 7.5 Hz, 2H), 2.18 – 2.09 (m, 1H), 2.06 – 1.98 (m, 2H), 1.92 – 1.79 (m, 2H), 1.53 – 1.45 (m, 2H), 1.45 – 1.35 (m, 2H), 1.25 – 1.10 (m, 4H), 0.96 (d, *J* = 6.9 Hz, 3H), 0.94 – 0.85 (m, 1H), 0.89 (t, *J* = 7.4 Hz, 3H), 0.74 – 0.64 (m, 1H), 0.67 (d, *J* = 6.8 Hz, 3H).

^{13}C NMR (126 MHz, Chloroform-*d*) δ 180.2, 173.6, 144.3, 136.3, 131.4, 127.5, 124.0, 123.5, 122.1, 107.6, 65.1, 56.4, 35.4 (2C), 34.31, 34.25, 29.6, 28.9, 25.8, 24.9, 24.3, 22.1, 17.4, 17.3, 13.7.

HRMS (ESI) calcd for $C_{25}H_{38}NO_3^+$ [(M+H)$^+$] 400.2846, found 400.2851.

5-(3-Isopropyl-1-methyl-2-oxoindolin-3-yl)pentyl acetate (3ay).

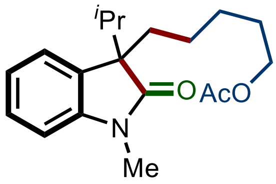

The title compound was isolated as a pale yellow oil in 92% yield (58.3 mg) starting from the carbamoyl chloride **1a** (47.5 mg) through column chromatography on silica gel (15% EtOAc in petroleum ether).

^{1}H NMR (400 MHz, Chloroform-*d*) δ 7.31 – 7.24 (m, 1H), 7.16 (d, *J* = 7.3 Hz, 1H), 7.05 (t, *J* = 7.5 Hz, 1H), 6.83 (d, *J* = 7.7 Hz, 1H), 3.93 (t, *J* = 6.7 Hz, 2H), 3.20 (s, 3H), 2.14 (hept, *J* = 6.6 Hz, 1H), 1.99 (s, 3H), 1.94 – 1.76 (m, 2H), 1.52 – 1.42 (m, 2H), 1.31 – 1.15 (m, 2H), 1.04 – 0.86 (m, 1H), 0.97 (d, *J* = 6.9 Hz, 3H), 0.79 – 0.59 (m, 1H), 0.67 (d, *J* = 6.8 Hz, 3H).

^{13}C NMR (101 MHz, Chloroform-*d*) δ 180.1, 171.2, 144.3, 131.2, 127.6, 123.4, 122.1, 107.6, 64.5, 56.3, 35.4, 35.3, 28.3, 26.3, 25.8, 24.2, 21.0, 17.4, 17.3.

HRMS (ESI) calcd for $C_{19}H_{28}NO_3^+$ [(M+H)$^+$] 318.2064, found 318.2073.

3-(Cyclohexylmethyl)-3-isopropyl-1-methylindolin-2-one (3az).

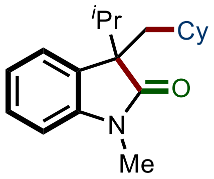

The title compound was isolated as a pale yellow oil in 77% yield (43.7 mg) starting from the carbamoyl chloride **1a** (47.5 mg) through column chromatography on silica gel (10% EtOAc in petroleum ether).

^{1}H NMR (400 MHz, Chloroform-*d*) δ 7.31 – 7.21 (m, 1H), 7.14 (ddd, *J* = 7.4, 1.3, 0.6 Hz, 1H), 7.03 (td, *J* = 7.5, 1.0 Hz, 1H), 6.82 (dt, *J* = 7.8, 0.8 Hz, 1H), 3.20 (s, 3H), 2.12 – 2.01 (m, 1H), 1.93 – 1.81 (m, 2H), 1.54 – 1.33 (m, 4H), 1.14 – 1.06 (m, 1H), 1.03 – 0.78 (m, 5H), 0.92 (d, *J* = 6.9 Hz, 3H), 0.74 – 0.61 (m, 1H), 0.65 (d, *J* = 6.8, 0.8 Hz, 3H).

^{13}C NMR (101 MHz, Chloroform-*d*) δ 180.4, 144.2, 131.5, 127.4, 123.9, 121.9, 107.6, 55.5, 42.5, 36.9, 34.8, 34.6, 33.8, 26.2, 26.1, 26.1, 25.9, 17.2, 17.1.

HRMS (ESI) calcd for $C_{19}H_{28}NO^+$ [$(M+H)^+$] 286.2165, found 286.2173.

3-Isopropyl-1-methyl-3-((tetrahydro-2*H*-pyran-4-yl)methyl)indolin-2-one (3aaa).

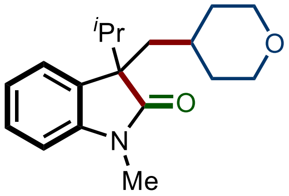

The title compound was isolated as a pale yellow oil in 80% yield (46.1 mg) starting from the carbamoyl chloride **1a** (47.5 mg) through column chromatography on silica gel (15% EtOAc in petroleum ether).

^{1}H NMR (400 MHz, Chloroform-*d*) δ 7.28 (t, *J* = 6.5 Hz, 1H), 7.16 (d, *J* = 7.3 Hz, 1H), 7.05 (t, *J* = 7.5 Hz, 1H), 6.83 (d, *J* = 7.7 Hz, 1H), 3.76 (dd, *J* = 9.0, 5.9 Hz, 1H), 3.71 – 3.62 (m, 1H), 3.21 (s, 3H), 3.15 – 2.99 (m, 2H), 2.13 – 2.03 (m, 1H), 1.99 – 1.86 (m, 2H), 1.32 – 1.21 (m, 2H), 1.20 – 1.09 (m, 1H), 1.09 – 0.97 (m, 1H), 0.95 (d, *J* = 6.9 Hz, 3H), 0.92 – 0.85 (m, 1H), 0.64 (d, *J* = 6.7 Hz, 3H).

^{13}C NMR (101 MHz, Chloroform-*d*) δ 180.1, 144.1, 131.1, 127.7, 123.9, 122.1, 107.7, 67.8, 67.7, 55.3, 42.1, 36.8, 34.2, 33.7, 32.3, 25.9, 17.2, 17.1.

HRMS (ESI) calcd for $C_{18}H_{26}NO_2^+$ [$(M+H)^+$] 288.1958, found 288.1958.

3-Isopropyl-3-(4-(4-methoxyphenyl)-2-methylbutyl)-1-methylindolin-2-one (3aab).

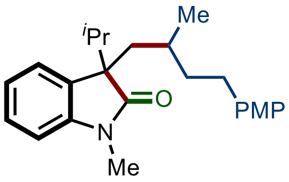

The title compound was isolated as a pale yellow oil in 68% yield (49.8 mg, dr = 2:1) starting from the carbamoyl chloride **1a** (47.5 mg) through column chromatography on silica gel (15% EtOAc in petroleum ether).

^{1}H NMR (400 MHz, Chloroform-*d*) δ (mixture of two diastereomers) 7.32 – 7.21 (m, 1H), 7.17 – 6.70 (m, 7H), 3.77 (s, 3H), 3.15 (s, 3H), 2.52 – 2.24 (m, 2H), 2.14 – 1.98 (m, 2H), 1.91 – 1.79 (m, 1H), 1.44 – 1.30 (m, 0.67H), 1.21 – 1.09 (m, 1.33H), 1.06 – 0.82 (m, 4H), 0.76 – 0.58 (m, 5H), 0.53 (d, *J* = 6.3 Hz, 1H).

^{13}C NMR (101 MHz, Chloroform-*d*) δ (major diastereomer) 180.4, 157.5, 144.3, 134.6, 131.1, 129.1 (2C), 127.5, 124.0, 121.9, 113.6 (2C), 107.7, 55.7, 55.3, 41.7, 40.1, 36.8, 32.1, 29.6, 25.9, 20.2, 17.3, 17.1.

^{13}C NMR (101 MHz, Chloroform-*d*) δ (minor diastereomer) 180.2, 157.4, 144.2, 134.9, 131.3, 129.2 (2C), 127.5, 124.1, 121.9, 113.6 (2C), 107.6, 55.7, 55.3, 42.2, 39.4, 36.9, 31.8, 29.9, 25.9, 20.9, 17.3, 17.1.

HRMS (ESI) calcd for $C_{24}H_{32}NO_2^+$ [(M+H)$^+$] 366.2428, found 366.2436.

3-Isopropyl-1-methyl-3-phenethylindolin-2-one (3aac).

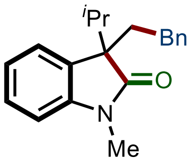

The title compound was isolated as a pale yellow oil in 71% yield (41.7 mg) starting from the carbamoyl chloride **1a** (47.5 mg) through column chromatography on silica gel (10% EtOAc in petroleum ether).

^{1}H NMR (400 MHz, Chloroform-*d*) δ 7.31 (t, *J* = 7.7 Hz, 1H), 7.26 – 7.17 (m, 3H), 7.16 – 7.06 (m, 2H), 7.03 (d, *J* = 7.5 Hz, 2H), 6.86 (d, *J* = 7.8 Hz, 1H), 3.21 (s, 3H), 2.33 – 2.07 (m, 4H), 2.06 – 1.92 (m, 1H), 0.98 (d, *J* = 6.9 Hz, 3H), 0.70 (d, *J* = 6.7 Hz, 3H).

^{13}C NMR (101 MHz, Chloroform-*d*) δ 179.8, 144.5, 141.8, 130.9, 128.34 (2C), 128.25 (2C), 127.8, 125.8, 123.5, 122.3, 107.7, 56.4, 37.6, 35.4, 31.0, 25.9, 17.4, 17.3.

HRMS (ESI) calcd for $C_{20}H_{23}NONa^+$ [(M+Na)$^+$] 316.1672, found 316.1678.

3-Isopropyl-3-(4-methoxyphenethyl)-1-methylindolin-2-one (3aad).

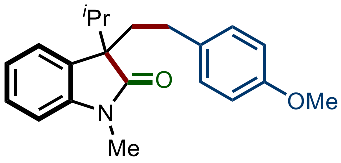

The title compound was isolated as a pale yellow oil in 56% yield (36.1 mg) starting from the carbamoyl chloride **1a** (47.5 mg) through column chromatography on silica gel (15% EtOAc in petroleum ether).

^{1}H NMR (400 MHz, Chloroform-*d*) δ 7.30 (td, *J* = 7.7, 1.3 Hz, 1H), 7.22 (dd, *J* = 7.3, 1.2 Hz, 1H), 7.09 (td, *J* = 7.5, 1.0 Hz, 1H), 6.94 (dt, *J* = 8.6, 2.3 Hz, 2H), 6.85 (d, *J* = 7.7 Hz, 1H), 6.75 (dt, *J* = 8.7, 2.4 Hz, 2H), 3.75 (s, 3H), 3.21 (s, 3H), 2.28 – 2.14 (m, 3H), 2.13 – 2.02 (m, 1H), 1.99 – 1.88 (m, 1H), 0.98 (d, *J* = 6.9 Hz, 3H), 0.69 (d, *J* = 6.8 Hz, 3H).

^{13}C NMR (101 MHz, Chloroform-*d*) δ 179.8, 157.7, 144.5, 133.9, 130.9, 129.2 (2C), 127.7, 123.5, 122.3, 113.7 (2C), 107.7, 56.4, 55.2, 37.8, 35.5, 30.0, 25.9, 17.4, 17.3.

HRMS (ESI) calcd for $C_{21}H_{25}NO_2Na^+$ [(M+Na)$^+$] 324.1958, found 324.1966.

4-(2-(3-Isopropyl-1-methyl-2-oxoindolin-3-yl)ethyl)phenyl acetate (3aae).

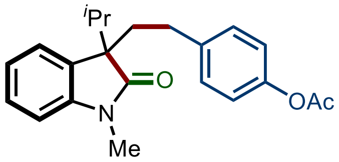

The title compound was isolated as a white solid in 74% yield (52.1 mg) starting from the carbamoyl chloride **1a** (47.5 mg) through column chromatography on silica gel (15% EtOAc in petroleum ether).

^{1}H NMR (400 MHz, Chloroform-*d*) δ 7.30 (t, *J* = 7.7 Hz, 1H), 7.21 (d, *J* = 7.4 Hz, 1H), 7.09 (t, *J* = 7.5 Hz, 1H), 7.01 (d, *J* = 8.0 Hz, 2H), 6.91 (d, *J* = 8.0 Hz, 2H), 6.85 (d, *J* = 7.8 Hz, 1H), 3.20 (s, 3H), 2.33 – 2.05 (m, 4H), 2.26 (s, 3H), 2.05 – 1.93 (m, 1H), 0.98 (d, *J* = 6.8 Hz, 3H), 0.69 (d, *J* = 6.7 Hz, 3H).

^{13}C NMR (101 MHz, Chloroform-*d*) δ 179.7, 169.7, 148.7, 144.4, 139.3, 130.8, 129.3 (2C), 127.8, 123.5, 122.3, 121.2 (2C), 107.8, 56.3, 37.4, 35.5, 30.4, 25.9, 21.2, 17.4, 17.3.

HRMS (ESI) calcd for $C_{22}H_{26}NO_3^+$ [(M+H)$^+$] 352.1907, found 352.1911.

3-(3-Chlorophenethyl)-3-isopropyl-1-methylindolin-2-one (3aaf).

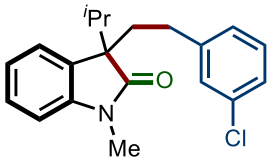

The title compound was isolated as a pale yellow oil in 59% yield (38.5 mg) starting from the carbamoyl chloride **1a** (47.5 mg) through column chromatography on silica gel (10% EtOAc in petroleum ether).

^{1}H NMR (400 MHz, Chloroform-*d*) δ 7.31 (t, *J* = 7.7 Hz, 1H), 7.21 (d, *J* = 7.4 Hz, 1H), 7.16 – 7.05 (m, 3H), 6.98 (s, 1H), 6.92 (d, *J* = 7.1 Hz, 1H), 6.86 (d, *J* = 7.8 Hz, 1H), 3.21 (s, 3H), 2.30 – 1.91 (m, 5H), 0.98 (d, *J* = 6.9 Hz, 3H), 0.69 (d, *J* = 6.6 Hz, 3H).

^{13}C NMR (101 MHz, Chloroform-*d*) δ 179.6, 144.4, 143.7, 133.9, 130.6, 129.5, 128.5, 127.9, 126.6, 126.0, 123.5, 122.4, 107.8, 56.2, 37.1, 35.5, 30.8, 25.9, 17.4, 17.2.

HRMS (ESI) calcd for $C_{20}H_{23}ClNO^+$ [(M+H)$^+$] 328.1463, found 328.1469.

3-(4-Chloro-3-methoxyphenethyl)-3-isopropyl-1-methylindolin-2-one (3aag).

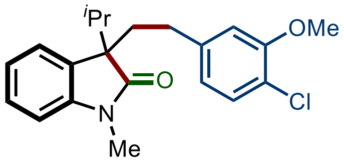

The title compound was isolated as a white solid in 71% yield (50.5 mg) starting from the carbamoyl chloride **1a** (47.5 mg) through column chromatography on silica gel (15% EtOAc in petroleum ether).

^{1}H NMR (400 MHz, Chloroform-*d*) δ 7.31 (td, *J* = 7.7, 1.3 Hz, 1H), 7.23 (ddd, *J* = 7.4, 1.3, 0.6 Hz, 1H), 7.17 (d, *J* = 8.0 Hz, 1H), 7.10 (td, *J* = 7.5, 1.0 Hz, 1H), 6.86 (dt, *J* = 7.9, 0.8 Hz, 1H), 6.60 – 6.51 (m, 2H), 3.84 (s, 3H), 3.20 (s, 3H), 2.29 – 2.07 (m, 4H), 2.05 – 1.95 (m, 1H), 0.98 (d, *J* = 6.9 Hz, 3H), 0.69 (d, *J* = 6.7 Hz, 3H).

^{13}C NMR (126 MHz, Chloroform-*d*) δ 179.7, 154.6, 144.5, 141.8, 130.7, 129.8, 127.9, 123.6, 122.3, 121.1, 119.7, 112.4, 107.8, 56.3, 56.1, 37.3, 35.5, 31.0, 25.9, 17.4, 17.3.

HRMS (ESI) calcd for $C_{21}H_{24}ClNO_2Na^+$ [(M+Na)$^+$] 380.1388, found 380.1395.

3-(2-(Benzo[*d*][1,3]dioxol-5-yl)ethyl)-3-isopropyl-1-methylindolin-2-one (3aah).

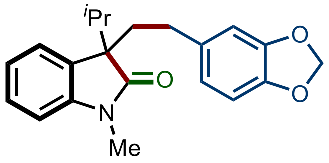

The title compound was isolated as a pale yellow oil in 56% yield (38.0 mg) starting from the carbamoyl chloride **1a** (47.5 mg) through column chromatography on silica gel (15% EtOAc in petroleum ether).

^{1}H NMR (400 MHz, Chloroform-*d*) δ 7.30 (td, *J* = 7.7, 1.3 Hz, 1H), 7.21 (d, *J* = 7.3 Hz, 1H), 7.09 (t, *J* = 7.5 Hz, 1H), 6.85 (d, *J* = 7.7 Hz, 1H), 6.65 (d, *J* = 7.9 Hz, 1H), 6.52 (d, *J* = 1.5 Hz, 1H), 6.46 (dd, *J* = 8.0, 1.6 Hz, 1H), 5.88 (s, 2H), 3.22 (s, 3H), 2.27 – 2.12 (m, 3H), 2.10 – 2.02 (m, 1H), 1.95 – 1.87 (m, 1H), 0.98 (d, *J* = 6.9 Hz, 3H), 0.68 (d, *J* = 6.7 Hz, 3H).

^{13}C NMR (101 MHz, Chloroform-*d*) δ 179.8, 147.4, 145.6, 144.4, 135.7, 130.9, 127.8, 123.5, 122.3, 121.0, 108.9, 108.0, 107.7, 100.7, 56.3, 37.9, 35.5, 30.7, 25.9, 17.4, 17.2.

HRMS (ESI) calcd for $C_{21}H_{24}NO_3^+$ [(M+H)$^+$] 338.1751, found 338.1758.

Supplementary Table 2. Ni-Precatalyst Screening for asymmetric carbo-acylation[a-c]

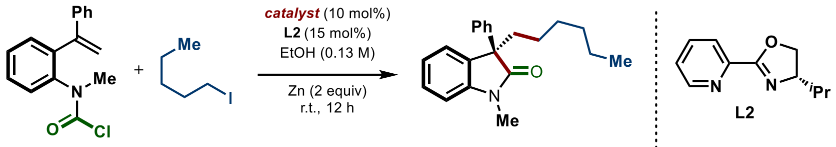

1f 2a 3fa

entry	precatalyst	yield (%)	*ee* (%)
1	$NiBr_2$·glyme	29	26
2	$NiBr_2$·diglyme	28	25
3	$NiBr_2$	0	-
4	NiI_2	trace	-
5	$NiCl_2$·glyme	28	26
6	$Ni(OTf)_2$	0	-
7	$Ni(acac)_2$	0	-
8	$NiCl_2$	0	-
9	$Ni(cod)_2$	28	24

[a] Reactions were performed on a 0.2 mmol scale of the carbamoyl chloride **1f** using 2.0 equiv of *n*-pentyl iodide (**2a**), 10 mol% Ni-precatalyst, 15 mol% ligand **L15**, 2.0 equiv of Zn as reductant in 1.5 mL EtOH at room temperature for 12 h, [b] NMR-yields using CH_2Br_2 as an internal standard, [c] Enantiomeric Excessess were determined by HPLC analysis on chiral stationary phase.

Supplementary Table 3. Ligand Screening for asymmetric carbo-acylation [a-c]

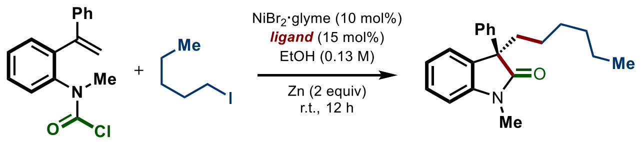

1f 2a 3fa

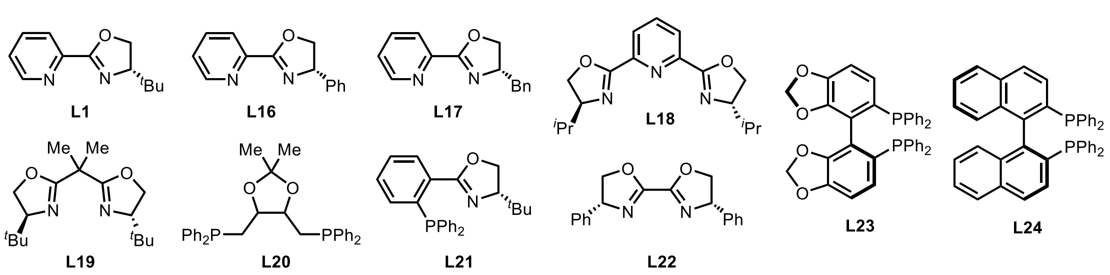

entry	ligand	yield (%)	*ee* (%)
1	**L1**	9	71
2	**L16**	0	-

3	**L17**	34	25
4	**L18**	0	-
5	**L19**	trace	-
6	**L20**	0	-
7	**L21**	0	-
8	**L22**	0	-
9	**L23**	0	-
10	**L24**	0	-

[a] Reactions were performed on a 0.2 mmol scale of the carbamoyl chloride **1f** using 2.0 equiv of *n*-pentyl iodide (**2a**), 10 mol% $NiBr_2$•glyme, 15 mol% ligand, 2.0 equiv of Zn as reductant in 1.5 mL EtOH at room temperature for 12 h, [b] NMR-yields using CH_2Br_2 as an internal standard, [c] Enantiomeric Excessess were determined by HPLC analysis on chiral stationary phase.

Supplementary Table 4. Solvent screening with Zn as reductant for asymmetric carbo-acylation [a-c]

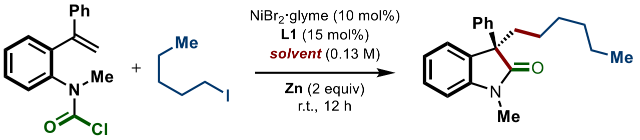

1f **2a** **3fa**

entry	solvent	yield (%)	*ee* (%)
1	THF	trace	-
2	PhOMe	0	-
3	MeCN	trace	-
4	DMAc	14	60
5	DMF	trace	-
6	NMP	20	53
7	MeOH	trace	-

[a] Reactions were performed on a 0.2 mmol scale of the carbamoyl chloride **1f** using 2.0 equiv of *n*-pentyl iodide (**2a**), 10 mol% $NiBr_2$•glyme, 15 mol% ligand **L1**, 2.0 equiv of Zn as reductant in 1.5 mL solvent at room temperature for 12 h, [b] NMR-yields using CH_2Br_2 as an internal standard, [c] Enantiomeric Excessess were determined by HPLC analysis on chiral stationary phase.

Supplementary Table 5. Solvent screening with Mn as reductant for asymmetric carbo-acylation [a-c]

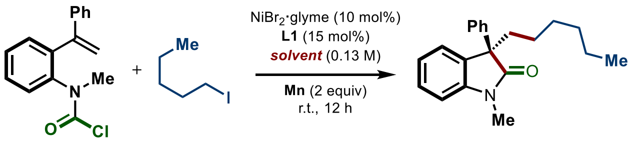

1f 2a 3af

entry	solvent[a]	yield (%)	*ee* (%)
1	THF	trace	-
2	Et_2O	0	-
3	PhOMe	0	-
4	MTBE[c]	0	-
5	CPME[d]	0	-
6	MeCN	trace	-
7	DMF	trace	-
8	DMA	50	64
9	DMPU	0	-
10	NMP	20	54
11	MeOH	trace	-
12	EtOH	11	73

[a] Reactions were performed on a 0.2 mmol scale of the carbamoyl chloride **1f** using 2.0 equiv of *n*-pentyl iodide (**2a**), 10 mol% $NiBr_2$•glyme, 15 mol% ligand **L1**, 2.0 equiv of Mn as reductant in 1.5 mL solvent at room temperature for 12 h, [b] NMR-yields using CH_2Br_2 as an internal standard, [c] Enantiomeric Excessess were determined by HPLC analysis on chiral stationary phase.

Supplementary Table 6. Additive screening for asymmetric carbo-acylation [a-c]

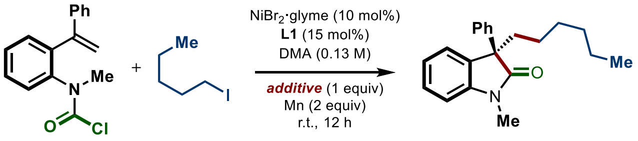

1f 2a 3af

entry	additive	yield (%)	*ee* (%)
1	KI	37	64
2	KBr	19	65
3	KF	28	63
4	CsF	trace	-
5	DMBA[d]	trace	-
6	4 Å MS	58	64
7	$MgCl_2$	trace	-

8	ZnI_2	62	63

[a] Reactions were performed on a 0.2 mmol scale of the carbamoyl chloride **1f** using 2.0 equiv of *n*-pentyl iodide (**2a**), 10 mol% $NiBr_2$•glyme, 15 mol% ligand **L1**, 2.0 equiv of Mn as reductant and 1.0 equiv of additive in 1.5 mL DMA at room temperature for 12 h, [b] NMR-yields using CH_2Br_2 as an internal standard, [c] Enantiomeric Excessess were determined by HPLC analysis on chiral stationary phase**.** [d] DMBA = 2,6-dimethylbenzoic acid.

Supplementary Table 7. Further ligand screening for asymmetric carbo-acylation [a-c]

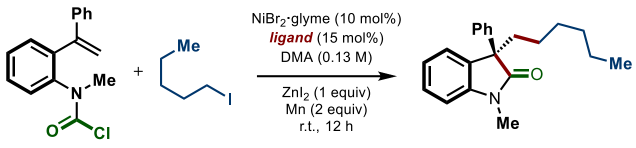

1f **2a** **3af**

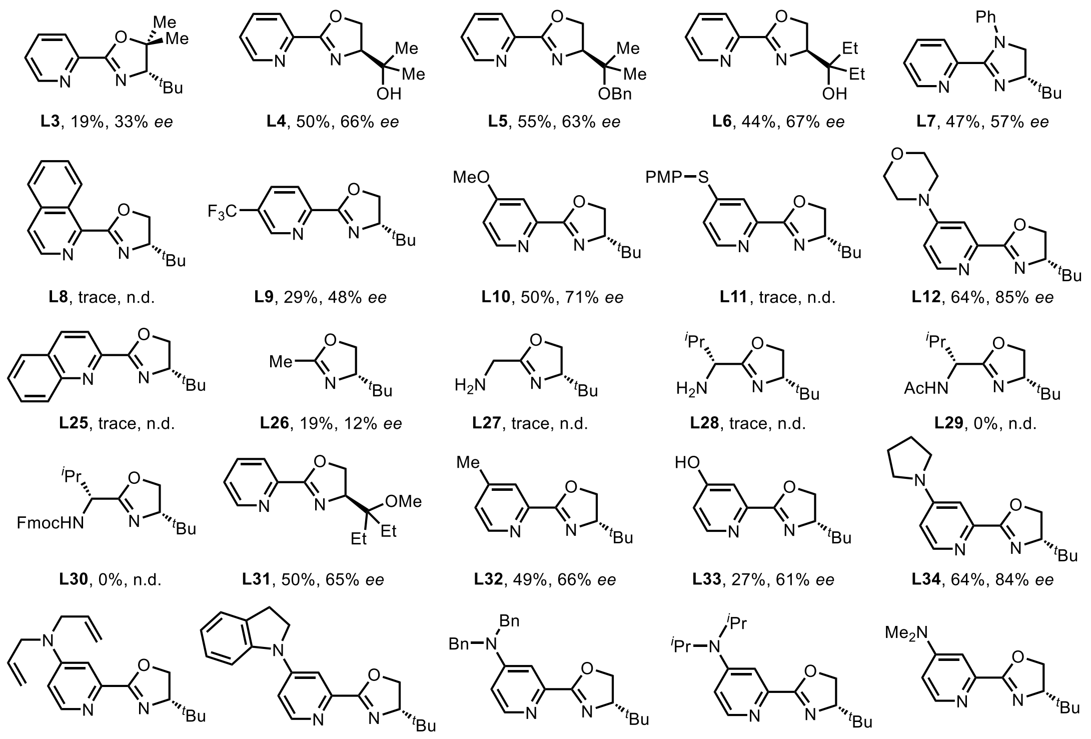

L3, 19%, 33% *ee*; **L4**, 50%, 66% *ee*; **L5**, 55%, 63% *ee*; **L6**, 44%, 67% *ee*; **L7**, 47%, 57% *ee*

L8, trace, n.d.; **L9**, 29%, 48% *ee*; **L10**, 50%, 71% *ee*; **L11**, trace, n.d.; **L12**, 64%, 85% *ee*

L25, trace, n.d.; **L26**, 19%, 12% *ee*; **L27**, trace, n.d.; **L28**, trace, n.d.; **L29**, 0%, n.d.

L30, 0%, n.d.; **L31**, 50%, 65% *ee*; **L32**, 49%, 66% *ee*; **L33**, 27%, 61% *ee*; **L34**, 64%, 84% *ee*

L35, 58%, 83% *ee*; **L36**, 60%, 79% *ee*; **L37**, 61%, 84% *ee*; **L38**, 60%, 83% *ee*; **L39**, 62%, 82% *ee*

[a] Reactions were performed on a 0.2 mmol scale of the carbamoyl chloride **1f** using 2.0 equiv of *n*-pentyl iodide (**2a**), 10 mol% $NiBr_2$•glyme, 15 mol% ligand, 2.0 equiv of Mn as reductant and 1.0 equiv of ZnI_2 as additive in 1.5 mL DMA at room temperature for 12 h, [b] NMR-yields using CH_2Br_2 as an internal standard, [c] Enantiomeric Excessess were determined by HPLC analysis on chiral stationary phase**.**

Supplementary Table 8. Co-solvent and concentration screening for asymmetric carbo-acylation [a-d]

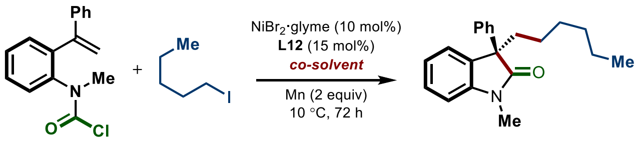

1f 2a 3af

entry	Solvent	yield (%)	ee (%)
1	DMA/NMM = 9:1 (0.13 M)	20	-
2	DMA/NMM = 4:1 (0.13 M)	45	87
3	DMA/NMM = 3:1 (0.13 M)	32	87
4	DMA/NMM = 4:1 (0.2 M)	33	87
5	DMA/NMM = 4:1 (0.4 M)	29	87
6[e]	DMA/NMM = 4:1 (0.13 M)	trace	-

[a] Reactions were performed on a 0.2 mmol scale of the carbamoyl chloride **1f** using 2.0 equiv of *n*-pentyl iodide (**2a**), 10 mol% $NiBr_2$•glyme, 15 mol% ligand **L14**, 2.0 equiv of Mn as reductant in 1.5 mL DMA/NMM at 10 °C for 72 h, [b] NMR-yields using CH_2Br_2 as an internal standard, [c] Enantiomeric Excessess were determined by HPLC analysis on chiral stationary phase, [d] NMM = *N*-methyl morpholine. [e] Reaction performed with 1.0 equiv of ZnI_2.

Supplementary Table 9. Further optimization of the catalyst loading for asymmetric carbo-acylation [a-c]

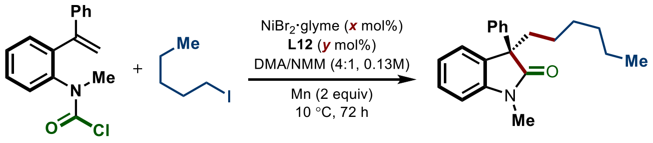

3f 2a 3af

entry	catalyst (*x* mol%)	ligand (*y* mol%)	yield (%)	*ee* (%)
1	15	20	55	88
2	10	20	21	87
3	20	20	61	88
4[d]	20	20	65 (61[e])	88

[a] Reactions were performed on a 0.2 mmol scale of the carbamoyl chloride **1f** using 2.0 equiv of *n*-pentyl iodide (**2a**), *x* mol% $NiBr_2$•glyme, *y* mol% ligand **L14**, 2.0 equiv of Mn as reductant and 1.0 equiv of additive in 1.5 mL DMA/NMM at 10 °C for 72 h, [b] NMR-yields using CH_2Br_2 as an internal standard, [c] Enantiomeric Excessess were determined by HPLC analysis on chiral stationary phase, [d] Reaction time: 96 h, [e] Yield of the isolated product.

Supplementary Figure 6. General procedure for asymmetric Ni-Catalyzed carbo-acylation

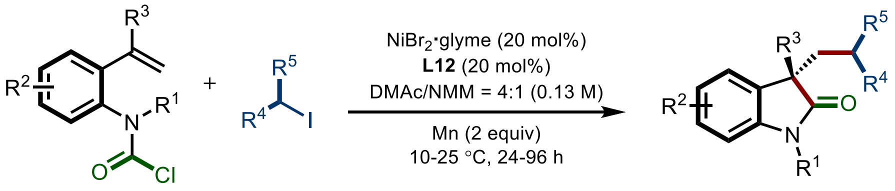

1 2 3

(*S*)-4-(2-(4-(*tert*-Butyl)-4,5-dihydrooxazol-2-yl)pyridin-4-yl)morpholine (**L12**) (11.6 mg, 0.04 mmol, 20 mol%), carbamoyl chlorides **1** (if solid, 0.2 mmol, 1 equiv) and alkyl iodides **2**[a] (if solid, 0.4 mmol, 2.0 equiv) were added to a reaction tube equipped with a stir bar. In a nitrogen-filled glovebox, $NiBr_2$·glyme (12.3 mg, 0.04 mmol, 20 mol%) and manganese dust (22 mg, 0.4 mmol, 2 equiv) were added to the mixture. The reaction tube was sealed and removed from the glovebox. Next, anhydrous DMAc (1.2 mL) and *N*-methyl morpholine (0.3 mL) were added, followed by the addition of carbamoyl chlorides **1** (if liquid, 0.2 mmol, 1 equiv) and alkyl iodides **2**[a] (if liquid, 0.4 mmol, 2.0 equiv) under the protection of nitrogen. Then the resulting mixture was stirred at the temperature specified below[b] for 24-96 h[c]. The reaction was quenched with sat. aq. NH_4Cl solution (5 mL) and diluted with water (10 mL). The aqueous layer was extracted three times with EtOAc, and the combined organic layers were washed with brine (20 mL), dried over $MgSO_4$, filtered, and concentrated under reduced pressure. The residue was purified through column chromatography on silica gel (petroleum ether/ethyl acetate) to afford the desired product **3**.

[a] Benzyl chloride was used for **3bac**,

[b] 25 °C for **3bx** and **3bac**, 10 °C for **3ba-da**, **3fa**, **3ma**, **3qa-ua**, **3bd**, **3bf**, **3bi, 3bk-bm**, **3bo**, **3br**, **3bv** and **3by-baa**,

[c] 24 h for **3bac**, 48 h for **3ba-da**, **3ma**, **3ra-ua**, **3bd**, **3bf**, **3bi, 3bk-bm**, **3bo**, **3br**, **3bv** and **3bx-baa**, 96 h for **3fa** and **3qa**.

(*S*)-3-Hexyl-1,3-dimethylindolin-2-one (3ba).

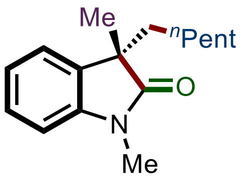

The title compound was isolated as a pale yellow oil in 61% yield (30.1 mg, 85% *ee*) starting from the carbamoyl chloride **1b** (41.9 mg) through column chromatography on silica gel (10% EtOAc in petroleum ether). For 1-mmol-scale reaction, the product was obtained in 58% yield (142.2 mg, 85% *ee*) starting the carbamoyl chloride **1b** (209.7 mg).

^{1}H NMR (400 MHz, Chloroform-*d*) δ 7.26 (t, *J* = 7.7 Hz, 1H), 7.17 (d, *J* = 7.3 Hz, 1H), 7.06 (t, *J* = 7.5 Hz, 1H), 6.84 (d, *J* = 7.7 Hz, 1H), 3.21 (s, 3H), 1.95 – 1.85 (m, 1H), 1.76 – 1.68 (m, 1H), 1.35 (s, 3H), 1.24 – 1.08 (m, 6H), 1.03 – 0.93 (m, 1H), 0.87 – 0.73 (m, 1H), 0.80 (t, *J* = 6.9 Hz, 3H).

^{13}C NMR (101 MHz, Chloroform-*d*) δ 180.9, 143.3, 134.3, 127.6, 122.5, 122.4, 107.9, 48.5, 38.6, 31.5, 29.4, 26.1, 24.4, 23.8, 22.6, 14.0.

HRMS (ESI) calcd for $C_{16}H_{24}NO^+$ [$(M+H)^+$] 246.1852, found 246.1852.

HPLC-Data: CHIRALPAK AD-H, 25 °C, ^{i}PrOH-hexanes 1.5/98.5, 1 mL/min, 254 nm, t_R(major) = 8.3 min, t_R(minor) = 9.1 min.

(*S*)-3-Ethyl-3-hexyl-1-methylindolin-2-one (3ca).

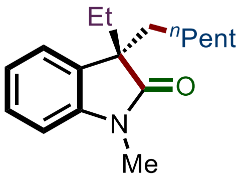

The title compound was isolated as a pale yellow oil in 85% yield (44.4 mg, 65% *ee*) starting from the carbamoyl chloride **1c** (44.7 mg) through column chromatography on silica gel (10% EtOAc in petroleum ether).

^{1}H NMR (400 MHz, Chloroform-*d*) δ 7.30 – 7.23 (m, 1H), 7.13 (d, *J* = 7.2 Hz, 1H), 7.07 (t, *J* = 7.4 Hz, 1H), 6.83 (d, *J* = 7.8 Hz, 1H), 3.21 (s, 3H), 1.96 – 1.83 (m, 2H), 1.82 – 1.66 (m, 2H), 1.22 – 1.07 (m, 6H), 1.00 – 0.90 (m, 1H), 0.85 – 0.72 (m, 1H), 0.80 (t, *J* = 6.9 Hz, 3H), 0.55 (t, *J* = 7.3 Hz, 3H).

^{13}C NMR (101 MHz, Chloroform-*d*) δ 180.2, 144.2, 132.4, 127.5, 122.6, 122.4, 107.7, 53.8, 37.8, 31.5, 31.0, 29.5, 26.0, 24.2, 22.6, 14.0, 8.6.

HRMS (ESI) calcd for $C_{17}H_{26}NO^+$ [$(M+H)^+$] 260.2009, found 260.2009.

HPLC-Data: CHIRALPAK IC, 25 °C, ^{i}PrOH-hexanes 20/80, 1 mL/min, 254 nm, t_R(major) = 5.0 min, t_R(minor) = 6.2 min.

(*S*)-3-Hexyl-1-methyl-3-propylindolin-2-one (3da).

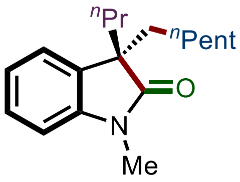

The title compound was isolated as a pale yellow oil in 80% yield (43.6 mg, 63% *ee*) starting from the carbamoyl chloride **1d** (47.5 mg) through column chromatography on silica gel (10% EtOAc in petroleum ether).

^{1}H NMR (400 MHz, Chloroform-*d*) δ 7.26 (t, *J* = 7.6 Hz, 1H), 7.14 (d, *J* = 7.2 Hz, 1H), 7.07 (t, *J* = 7.4 Hz, 1H), 6.83 (d, *J* = 7.7 Hz, 1H), 3.20 (s, 3H), 1.91 – 1.81 (m, 2H), 1.77 – 1.66 (m, 2H), 1.23 – 1.08 (m, 6H), 1.03 – 0.91 (m, 2H), 0.87 – 0.74 (m, 2H), 0.80 (t, *J* = 6.8 Hz, 3H), 0.76 (t, *J* = 6.4 Hz, 3H).

^{13}C NMR (101 MHz, Chloroform-*d*) δ 180.4, 144.0, 132.8, 127.5, 122.6, 122.3, 107.7, 53.3, 40.4, 38.1, 31.5, 29.5, 26.0, 24.1, 22.6, 17.5, 14.2, 14.0.

HRMS (ESI) calcd for $C_{18}H_{28}NO^+$ [$(M+H)^+$] 274.2165, found 274.2165.

HPLC-Data: CHIRALPAK IC, 25 °C, ^{i}PrOH-hexanes 20/80, 1 mL/min, 254 nm, t_R(major) = 4.6 min, t_R(minor) = 5.3 min.

(*R*)-3-Hexyl-1-methyl-3-phenylindolin-2-one (3fa).

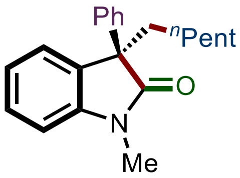

The title compound was isolated as a pale yellow oil in 61% yield (37.6 mg, 88% *ee*) starting from the carbamoyl chloride **1f** (54.3 mg) through column chromatography on silica gel (10% EtOAc in petroleum ether).

^{1}H NMR (400 MHz, Chloroform-*d*) δ 7.40 – 7.18 (m, 7H), 7.11 (td, *J* = 7.5, 1.1 Hz, 1H), 6.90 (d, *J* = 7.8 Hz, 1H), 3.22 (s, 3 H), 2.36 (td, *J* = 12.7, 4.4 Hz, 1H), 2.18 (td, *J* = 12.8, 4.2 Hz, 1H), 1.29 – 1.07 (m, 7H), 0.92 – 0.79 (m, 1H), 0.81 (t, *J* = 6.8 Hz, 3H).

^{13}C NMR (101 MHz, Chloroform-*d*) δ 178.7, 144.0, 140.4, 132.4, 128.5 (2C), 128.1, 127.2, 126.9 (2C), 124.8, 122.6, 108.2, 56.8, 38.0, 31.5, 29.5, 26.4, 24.5, 22.6, 14.1.

HRMS (ESI) calcd for $C_{21}H_{26}NO^+$ [(M+H)$^+$] 308.2009, found 308.2008.

HPLC-Data: CHIRALPAK AD-H, 25 °C, ^{i}PrOH-hexanes 15/85, 1 mL/min, 254 nm, t_R(major) = 5.6 min, t_R(minor) = 6.2 min.

(*R*)-3-Hexyl-3-(4-methoxyphenyl)-1-methylindolin-2-one (3qa).

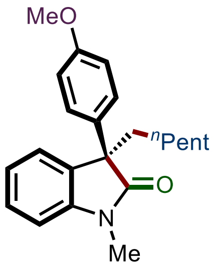

The title compound was isolated as a pale yellow oil in 52% yield (35.2 mg, 89% *ee*) starting from the carbamoyl chloride **1q** (60.4 mg) through column chromatography on silica gel (10% EtOAc in petroleum ether).

^{1}H NMR (400 MHz, Chloroform-*d*) δ 7.33 (td, J = 7.7, 1.3 Hz, 1H), 7.28 (d, J = 2.2 Hz, 1H), 7.26 (d, J = 1.7 Hz, 1H), 7.22 (dd, J = 7.5, 0.6 Hz, 1H), 7.11 (td, J = 7.5, 1.1 Hz, 1H), 6.90 (d, J = 7.8 Hz, 1H), 6.84 – 6.78 (m, 2H), 3.76 (s, 3H), 3.21 (s, 3H), 2.32 (td, J = 12.7, 4.4 Hz, 1H), 2.14 (td, J = 12.8, 4.2 Hz, 1H), 1.28 – 1.06 (m, 7H), 0.93 – 0.77 (m, 1H), 0.81 (t, J = 6.9 Hz, 3H).

^{13}C NMR (101 MHz, Chloroform-*d*) δ 179.0, 158.7, 144.0, 132.6, 132.5, 128.0 (2C), 124.7, 122.5, 113.8 (3C), 108.2, 56.1, 55.3, 38.1, 31.5, 29.5, 26.3, 24.5, 22.6, 14.0.

HRMS (ESI) calcd for $C_{22}H_{28}NO_2^+$ [(M+H)$^+$] 338.2115, found 338.2123.

HPLC-Data: CHIRALPAK IC, 25 °C, ^{i}PrOH-hexanes 30/70, 1 mL/min, 254 nm, t_R(major) = 13.1 min, t_R(minor) = 8.5 min.

(*S*)-3-Hexyl-1,3-dimethyl-6-(trifluoromethyl)indolin-2-one (3ma).

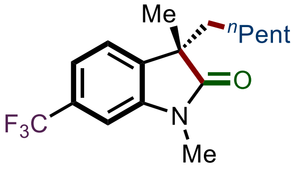

The title compound was isolated as a pale yellow oil in 50% yield (31.3 mg, 82% *ee*) starting from the carbamoyl chloride **1m** (55.4 mg) through column chromatography on silica gel (10% EtOAc in petroleum ether).

^{1}H NMR (400 MHz, Chloroform-*d*) δ 7.35 (d, J = 7.7 Hz, 1H), 7.26 (d, J = 7.5 Hz, 1H), 7.04 (s, 1H), 3.25 (s, 3H), 1.92 (td, J = 12.8, 4.6 Hz, 1H), 1.74 (td, J = 12.9, 4.4 Hz, 1H), 1.37 (s, 3H), 1.24 – 1.09 (m, 6H), 1.02 – 0.91 (m, 1H), 0.86 – 0.75 (m, 1H),

0.81 (t, J = 6.9 Hz, 3H).

^{13}C NMR (101 MHz, Chloroform-*d*) δ 180.5, 143.9, 138.2, 130.2 (q, J = 32.3 Hz), 124.1 (q, J = 272.6 Hz), 122.6, 119.5 (q, J = 4.1 Hz), 104.6 (q, J = 3.9 Hz), 48.6, 38.4, 31.5, 29.3, 26.3, 24.4, 23.6, 22.5, 14.0.

^{19}F NMR (471 MHz, Chloroform-*d*) δ −62.26 (s, 3F).

HRMS (ESI) calcd for $C_{17}H_{23}F_3NO^+$ [$(M+H)^+$] 314.1726, found 314.1732.

HPLC-Data: CHIRALPAK AD-H, 25 °C, ^{i}PrOH-hexanes 1/99, 1 mL/min, 254 nm, t_R(major) = 5.6 min, t_R(minor) = 6.3 min.

(*S*)-3-Hexyl-1,3,6-trimethylindolin-2-one (3ra).

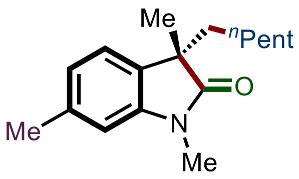

The title compound was isolated as a pale yellow oil in 65% yield (33.8 mg, 84% *ee*) starting from the carbamoyl chloride **1r** (44.7 mg) through column chromatography on silica gel (10% EtOAc in petroleum ether).

^{1}H NMR (400 MHz, Chloroform-*d*) δ 7.04 (dd, J = 7.5, 1.4 Hz, 1H), 6.88 (d, J = 7.4 Hz, 1H), 6.67 (s, 1H), 3.19 (s, 3H), 2.39 (s, 3H), 1.86 (tdd, J = 12.2, 4.7, 1.4 Hz, 1H), 1.70 (tdd, J = 13.2, 4.5, 1.4 Hz, 1H), 1.32 (s, 3H), 1.22 – 1.08 (m, 6H), 1.04 – 0.93 (m, 1H), 0.89 – 0.77 (m, 1H), 0.81 (td, J = 6.9, 1.5 Hz, 3H).

^{13}C NMR (101 MHz, Chloroform-*d*) δ 181.2, 143.4, 137.6, 131.4, 122.9, 122.2, 108.8, 48.3, 38.6, 31.6, 29.4, 26.1, 24.4, 23.9, 22.6, 21.8, 14.0.

HRMS (ESI) calcd for $C_{17}H_{26}NO^+$ [$(M+H)^+$] 260.2009, found 260.2008.

HPLC-Data: CHIRALPAK AD-H, 25 °C, ^{i}PrOH-hexanes 2/98, 1 mL/min, 254 nm, t_R(major) = 5.8 min, t_R(minor) = 6.3 min.

(*S*)-3-Hexyl-6-methoxy-1,3-dimethylindolin-2-one (3sa).

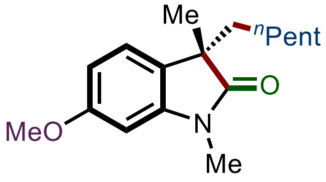

The title compound was isolated as a pale yellow oil in 55% yield (30.3 mg, 83% *ee*) starting from the carbamoyl chloride **1s** (47.9 mg) through column chromatography on

silica gel (15% EtOAc in petroleum ether).

^{1}H NMR (400 MHz, Chloroform-*d*) δ 7.05 (d, J = 8.1 Hz, 1H), 6.56 (dd, *J* = 8.1, 2.3 Hz, 1H), 6.43 (d, *J* = 2.3 Hz, 1H), 3.83 (s, 3H), 3.19 (s, 3H), 1.85 (ddd, *J* = 13.3, 12.1, 4.8 Hz, 1H), 1.68 (ddd, *J* = 13.3, 12.2, 4.5 Hz, 1H), 1.32 (s, 3H), 1.24 – 1.07 (m, 6H), 1.00 – 0.90 (m, 1H), 0.89 – 0.77 (m, 1H), 0.81 (t, *J* = 16.9 Hz, 3H),.

^{13}C NMR (101 MHz, Chloroform-*d*) δ 181.5, 159.8, 144.5, 126.3, 122.9, 106.1, 95.9, 55.5, 48.0, 38.7, 31.5, 29.4, 26.1, 24.4, 23.9, 22.6, 14.0.

HRMS (ESI) calcd for $C_{17}H_{26}NO_2^+$ [(M+H)$^+$] 276.1958, found 276.1961.

HPLC-Data: CHIRALPAK IB, 25 °C, ^{i}PrOH-hexanes 3/97, 1 mL/min, 254 nm, t_R(major) = 5.1 min, t_R(minor) = 6.6 min.

(*S*)-5-Chloro-3-hexyl-1,3-dimethylindolin-2-one (3ta).

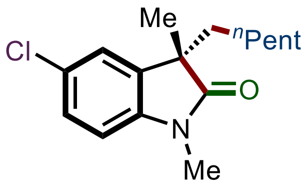

The title compound was isolated as a pale yellow oil in 56% yield (31.2 mg, 80% *ee*) starting from the carbamoyl chloride **1t** (48.8 mg) through column chromatography on silica gel (10% EtOAc in petroleum ether).

^{1}H NMR (400 MHz, Chloroform-*d*) δ 7.24 (dd, *J* = 8.2, 2.1 Hz, 1H), 7.14 (d, *J* = 2.1 Hz, 1H), 6.76 (d, *J* = 8.3 Hz, 1H), 3.20 (s, 3H), 1.89 (td, *J* = 12.9, 4.7 Hz, 1H), 1.69 (td, *J* = 12.9, 4.4 Hz, 1H), 1.34 (s, 3H), 1.23 – 1.08 (m, 6H), 1.02 – 0.88 (m, 1H), 0.87 – 0.74 (m, 1H), 0.82 (t, *J* = 6.9 Hz, 3H).

^{13}C NMR (101 MHz, Chloroform-*d*) δ 180.3, 141.9, 136.1, 127.8, 127.5, 123.0, 108.8, 48.8, 38.5, 31.5, 29.4, 26.2, 24.4, 23.7, 22.6, 14.0.

HRMS (ESI) calcd for $C_{16}H_{23}ClNO^+$ [(M+H)$^+$] 280.1463, found 280.1473.

HPLC-Data: CHIRALPAK AD-H, 25 °C, ^{i}PrOH-hexanes 2/98, 1 mL/min, 254 nm, t_R(major) = 5.8 min, t_R(minor) = 6.4 min.

(*S*)-1-Benzyl-3-hexyl-3-methylindolin-2-one (3ua).

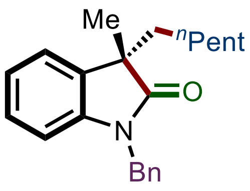

The title compound was isolated as a pale yellow oil in 51%

yield (32.9 mg, 80% *ee*) starting from the carbamoyl chloride **1u** (57.0 mg) through column chromatography on silica gel (10% EtOAc in petroleum ether).

^{1}H NMR (500 MHz, Chloroform-*d*) δ 7.33 – 7.21 (m, 5H), 7.19 – 7.11 (m, 2H), 7.02 (t, *J* = 7.4 Hz, 1H), 6.71 (d, *J* = 7.8 Hz, 1H), 4.99 (d, *J* = 15.7 Hz, 1H), 4.84 (d, *J* = 15.6 Hz, 1H), 1.96 (td, *J* = 12.9, 4.7 Hz, 1H), 1.77 (td, *J* = 12.8, 4.3 Hz, 1H), 1.40 (s, 3H), 1.22 – 1.10 (m, 6H), 1.09 – 1.00 (m, 1H), 0.89 – 0.77 (m, 1H), 0.81 (t, *J* = 6.9 Hz, 3H).

^{13}C NMR (126 MHz, Chloroform-*d*) δ 181.0, 142.4, 136.2, 134.3, 128.7 (2C), 127.52, 127.49, 127.3 (2C), 122.6, 122.4, 109.0, 48.5, 43.6, 38.7, 31.5, 29.4, 24.6, 24.2, 22.5, 14.0.

HRMS (ESI) calcd for $C_{22}H_{27}NONa^+$ [$(M+Na)^+$] 344.1985, found 344.1985.

HPLC-Data: CHIRALPAK AD-H, 25 °C, ^{i}PrOH-hexanes 10/90, 1 mL/min, 254 nm, t_R(major) = 5.8 min, t_R(minor) = 7.3 min.

(*S*)-3-(4-Chlorobutyl)-1,3-dimethylindolin-2-one (3bd).

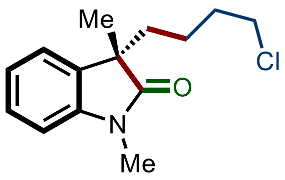

The title compound was isolated as a pale yellow oil in 62% yield (31.1 mg, 82% *ee*) starting from the carbamoyl chloride **1b** (41.9 mg) through column chromatography on silica gel (10% EtOAc in petroleum ether).

^{1}H NMR (400 MHz, Chloroform-*d*) δ 7.28 (td, *J* = 7.6, 1.3 Hz, 1H), 7.18 (dd, *J* = 7.4, 1.3 Hz, 1H), 7.08 (td, *J* = 7.5, 1.0 Hz, 1H), 6.85 (dt, *J* = 7.7, 0.7 Hz, 1H), 3.40 (td, *J* = 6.8, 1.7 Hz, 2H), 3.22 (s, 3H), 1.91 (td, *J* = 12.8, 5.0 Hz, 1H), 1.76 (td, *J* = 12.5, 4.6 Hz, 1H), 1.71 – 1.58 (m, 2H), 1.36 (s, 3H), 1.17 – 0.95 (m, 2H).

^{13}C NMR (101 MHz, Chloroform-*d*) δ 180.6, 143.3, 133.8, 127.8, 122.6, 122.5, 108.0, 48.3, 44.5, 37.6, 32.6, 26.2, 23.8, 22.0.

HRMS (ESI) calcd for $C_{14}H_{19}ClNO^+$ [$(M+H)^+$] 252.1150, found 252.1157.

HPLC-Data: CHIRALPAK AD-H, 25 °C, ^{i}PrOH-hexanes 2/98, 1 mL/min, 254 nm, t_R(major) = 11.3 min, t_R(minor) = 12.5 min.

(*S*)-6-(1,3-Dimethyl-2-oxoindolin-3-yl)hexanenitrile (3bf).

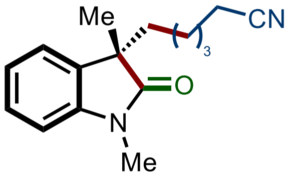

The title compound was isolated as a pale yellow oil in 63% yield (32.3 mg, 81% *ee*) starting from the carbamoyl chloride **1b** (41.9 mg) through column chromatography on silica gel (20% EtOAc in petroleum ether).

^{1}H NMR (400 MHz, Chloroform-*d*) δ 7.28 (td, J = 7.7, 1.3 Hz, 1H), 7.16 (ddd, J = 7.3, 1.4, 0.6 Hz, 1H), 7.08 (td, J = 7.5, 1.0 Hz, 1H), 6.85 (dt, J = 7.8, 0.8 Hz, 1H), 3.22 (s, 3H), 2.23 (t, J = 7.1 Hz, 2H), 1.92 (ddd, J = 13.3, 12.1, 4.8 Hz, 1H), 1.73 (ddd, J = 13.3, 12.2, 4.5 Hz, 1H), 1.58 – 1.47 (m, 2H), 1.40 – 1.28 (m, 2H), 1.35 (s, 3H), 1.07 – 0.95 (m, 1H), 0.92 – 0.82 (m, 1H).

^{13}C NMR (101 MHz, Chloroform-*d*) δ 180.6, 143.3, 133.9, 127.8, 122.6, 122.4, 119.6, 108.0, 48.3, 38.0, 28.6, 26.2, 25.0, 23.9, 23.7, 17.0.

HPLC-Data: CHIRALPAK AD-H, 25 °C, ^{i}PrOH-hexanes 10/90, 1 mL/min, 254 nm, t_R(major) = 9.2 min, t_R(minor) = 9.8 min.

HRMS (ESI) calcd for $C_{16}H_{21}N_2O^+$ [(M+H)$^+$] 257.1648, found 257.1653.

(*S*)-1,3-Dimethyl-3-(4-(4-(methylsulfonyl)phenoxy)butyl)indolin-2-one (3bi).

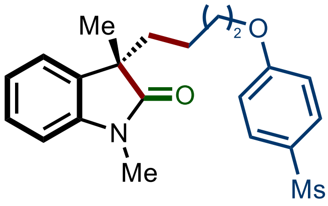

The title compound was isolated as a pale yellow oil in 73% yield (56.5 mg, 86% ee) starting from the carbamoyl chloride **1b** (41.9 mg) through column chromatography on silica gel (50% EtOAc in petroleum ether).

^{1}H NMR (500 MHz, Chloroform-*d*) δ 7.85 – 7.79 (m, 2H), 7.28 (td, J = 7.7, 1.1 Hz, 1H), 7.18 (d, J = 7.1 Hz, 1H), 7.08 (t, J = 7.5 Hz, 1H), 6.94 – 6.90 (m, 2H), 6.86 (d, J = 7.8 Hz, 1H), 3.89 (t, J = 6.5 Hz, 2H), 3.22 (s, 3H), 3.02 (s, 3H), 1.98 (td, J = 12.7, 4.7 Hz, 1H), 1.81 (td, J = 12.8, 4.4 Hz, 1H), 1.75 – 1.60 (m, 2H), 1.37 (s, 3H), 1.20 – 1.11 (m, 1H), 1.09 – 0.98 (m, 1H).

^{13}C NMR (126 MHz, Chloroform-*d*) δ 180.6, 163.1, 143.3, 133.9, 132.0, 129.5 (2C), 127.8, 122.6, 122.5, 114.9 (2C), 108.1, 68.0, 48.4, 44.9, 38.1, 29.0, 26.2, 23.9, 21.1.

HRMS (ESI) calcd for $C_{21}H_{25}NO_4SNa^+$ [(M+Na)$^+$] 410.1397, found 410.1403.

HPLC-Data: CHIRALPAK AD-H, 25 °C, ^{i}PrOH-hexanes 40/60, 1 mL/min, 254 nm, t_R(major) = 9.8 min, t_R(minor) = 8.6 min.

(*S*)-3-(4-(2-(Hydroxymethyl)phenoxy)butyl)-1,3-dimethylindolin-2-one (3bk).

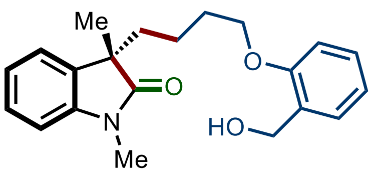

The title compound was isolated as a pale yellow oil in 46% yield (31.2 mg, 83% *ee*) starting from the carbamoyl chloride **1b** (41.9 mg) through column chromatography on silica gel (60% EtOAc in petroleum ether).

^{1}H NMR (400 MHz, Chloroform-*d*) δ 7.30 – 7.15 (m, 4H), 7.07 (td, *J* = 7.5, 1.0 Hz, 1H), 6.91 (td, *J* = 7.4, 1.1 Hz, 1H), 6.85 (dt, *J* = 7.7, 0.7 Hz, 1H), 6.78 (dd, *J* = 8.2, 1.0 Hz, 1H), 4.56 (s, 2H), 3.96 – 3.85 (m, 2H), 3.21 (s, 3H), 2.39 – 2.09 (brs, 1H), 2.08 – 1.94 (m, 1H), 1.85 – 1.76 (m, 1H), 1.76 – 1.60 (m, 2H), 1.37 (s, 3H), 1.21 – 1.11 (m, 1H), 1.10 – 0.98 (m, 1H).

^{13}C NMR (101 MHz, Chloroform-*d*) δ 180.7, 156.7, 143.3, 133.9, 129.2, 128.9 (2C), 127.8, 122.6, 122.4, 120.6, 111.1, 108.1, 67.3, 62.0, 48.5, 38.0, 29.2, 26.2, 23.8, 21.1.

HRMS (ESI) calcd for $C_{21}H_{25}NO_3Na^+$ [$(M+Na)^+$] 362.1727, found 362.1732.

HPLC-Data: CHIRALPAK IC, 25 °C, ^{i}PrOH-hexanes 50/50, 1 mL/min, 254 nm, t_R(major) = 7.4 min, t_R(minor) = 8.5 min.

(*S*)-4-(4-(1,3-Dimethyl-2-oxoindolin-3-yl)butoxy)benzaldehyde (3bl).

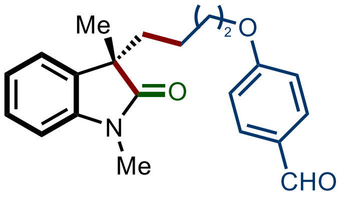

The title compound was isolated as a pale yellow oil in 63% yield (42.5 mg, 85% *ee*) starting from the carbamoyl chloride **1b** (41.9 mg) through column chromatography on silica gel (30% EtOAc in petroleum ether).

^{1}H NMR (500 MHz, Chloroform-*d*) δ 9.86 (s, 1H), 7.82 – 7.75 (m, 2H), 7.32 – 7.24 (m, 1H), 7.18 (d, *J* = 7.1 Hz, 1H), 7.07 (t, *J* = 7.5 Hz, 1H), 6.90 (d, *J* = 8.5 Hz, 2H), 6.85 (d, *J* = 7.7 Hz, 1H), 3.91 (td, *J* = 6.5, 2.7 Hz, 2H), 3.22 (s, 3H), 1.98 (td, *J* = 12.7, 4.8 Hz, 1H), 1.81 (td, *J* = 12.8, 4.4 Hz, 1H), 1.75 – 1.62 (m, 2H), 1.37 (s, 3H), 1.21 –

1.11 (m, 1H), 1.10 – 0.97 (m, 1H),.

^{13}C NMR (126 MHz, Chloroform-*d*) δ 190.8, 180.6, 164.1, 143.3, 133.9, 132.0 (2C), 129.8, 127.8, 122.6, 122.5, 114.7 (2C), 108.0, 67.9, 48.4, 38.1, 29.1, 26.2, 23.9, 21.1.

HRMS (ESI) calcd for $C_{21}H_{24}NO_3^+$ [(M+H)$^+$] 338.1751, found 338.1752.

HPLC-Data: CHIRALPAK AD-H, 25 °C, ^{i}PrOH-hexanes 20/80, 1 mL/min, 254 nm, t_R(major) = 9.8 min, t_R(minor) = 11.2 min.

(*S*)-3-(6-(4-Acetylphenoxy)hexyl)-1,3-dimethylindolin-2-one (3bm).

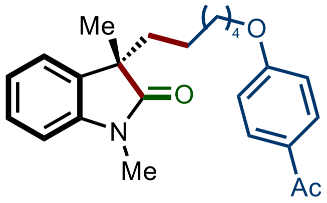

The title compound was isolated as a pale yellow oil in 74% yield (55.9 mg, 85% *ee*) starting from the carbamoyl chloride **1b** (41.9 mg) through column chromatography on silica gel (20% EtOAc in petroleum ether).

^{1}H NMR (400 MHz, Chloroform-*d*) δ 7.94 – 7.88 (m, 2H), 7.27 (td, *J* = 7.7, 1.3 Hz, 1H), 7.17 (ddd, *J* = 7.3, 1.4, 0.6 Hz, 1H), 7.07 (td, *J* = 7.5, 1.0 Hz, 1H), 6.89 – 6.82 (m, 3H), 3.93 (t, *J* = 6.5 Hz, 2H), 3.21 (s, 3H), 2.55 (s, 3H), 1.99 – 1.84 (m, 1H), 1.79 – 1.62 (m, 3H), 1.42 – 1.30 (m, 2H), 1.35 (s, 3H), 1.27 – 1.18 (m, 2H), 1.07 – 0.94 (m, 1H), 0.93 – 0.81 (m, 1H).

^{13}C NMR (101 MHz, Chloroform-*d*) δ 196.8, 180.8, 163.1, 143.3, 134.2, 130.6 (2C), 130.1, 127.7, 122.45, 122.46, 114.1 (2C), 107.9, 68.1, 48.4, 38.4, 29.4, 29.0, 26.4, 26.1, 25.7, 24.4, 23.9.

HRMS (ESI) calcd for $C_{24}H_{30}NO_3^+$ [(M+H)$^+$] 380.2220, found 380.2225.

HPLC-Data: CHIRALPAK AD-H, 25 °C, ^{i}PrOH-hexanes 20/80, 1 mL/min, 254 nm, t_R(major) = 11.0 min, t_R(minor) = 11.9 min.

Phenyl (*S*)-7-(1,3-dimethyl-2-oxoindolin-3-yl)heptanoate (3bo).

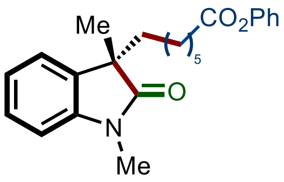

The title compound was isolated as a pale yellow oil in 67% yield (48.8 mg, 84% *ee*) starting from the carbamoyl chloride **1b** (41.9 mg) through column chromatography on silica gel (15% EtOAc in petroleum ether).

^{1}H NMR (400 MHz, Chloroform-*d*) δ 7.36 (t, *J* = 7.7 Hz, 2H), 7.29 – 7.24 (m, 1H), 7.21 (t, *J* = 7.4 Hz, 1H), 7.17 (d, *J* = 7.3 Hz, 1H), 7.09 – 7.01 (m, 3H), 6.84 (d, *J* = 7.8 Hz, 1H), 3.21 (s, 3H), 2.47 (t, *J* = 7.5 Hz, 2H), 1.91 (td, *J* = 12.7, 4.7 Hz, 1H), 1.73 (td, *J* = 12.8, 4.4 Hz, 1H), 1.67 – 1.57 (m, 2H), 1.35 (s, 3H), 1.32 – 1.15 (m, 4H), 1.07 – 0.94 (m, 1H), 0.92 – 0.77 (m, 1H).

^{13}C NMR (101 MHz, Chloroform-*d*) δ 180.8, 172.2, 150.7, 143.3, 134.2, 129.4 (2C), 127.7, 125.7, 122.48, 122.46, 121.6 (2C), 107.9, 48.4, 38.4, 34.3, 29.4, 28.8, 26.1, 24.8, 24.3, 23.9.

HRMS (ESI) calcd for $C_{23}H_{28}NO_3^+$ [(M+H)$^+$] 366.2064, found 366.2069.

HPLC-Data: CHIRALPAK AD-H, 25 °C, ^{i}PrOH-hexanes 10/90, 1 mL/min, 254 nm, t_R(major) = 9.6 min, t_R(minor) = 10.4 min.

4-(Methylthio)phenyl (*S*)-7-(1,3-dimethyl-2-oxoindolin-3-yl)heptanoate (3br).

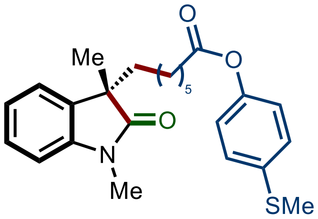

The title compound was isolated as a pale yellow oil in 75% yield (61.6 mg, 84% *ee*) starting from the carbamoyl chloride **1b** (41.9 mg) through column chromatography on silica gel (20% EtOAc in petroleum ether).

^{1}H NMR (400 MHz, Chloroform-*d*) δ 7.29 – 7.22 (m, 3H), 7.17 (dd, *J* = 7.3, 1.2 Hz, 1H), 7.06 (td, *J* = 7.5, 1.1 Hz, 1H), 7.01 – 6.93 (m, 2H), 6.84 (dt, *J* = 7.7, 0.7 Hz, 1H), 3.21 (s, 3H), 2.46 (s, 3H), 2.46 (t, *J* = 7.5 Hz, 2H), 1.90 (td, *J* = 12.8, 4.7 Hz, 1H), 1.73 (td, *J* = 12.8, 4.4 Hz, 1H), 1.67 – 1.57 (m, 2H), 1.35 (s, 3H), 1.31 – 1.16 (m, 4H), 1.07 – 0.94 (m, 1H), 0.91 – 0.78 (m, 1H).

^{13}C NMR (101 MHz, Chloroform-*d*) δ 180.8, 172.2, 148.5, 143.3, 135.5, 134.2, 128.0 (2C), 127.7, 122.48, 122.46, 122.1 (2C), 107.9, 48.4, 38.4, 34.2, 29.3, 28.8, 26.1, 24.8, 24.3, 23.9, 16.5.

HRMS (ESI) calcd for $C_{24}H_{30}NO_3S^+$ [$(M+H)^+$] 412.1941, found 412.1948.

HPLC-Data: CHIRALPAK AD-H, 25 °C, ^{i}PrOH-hexanes 20/80, 1 mL/min, 254 nm, t_R(major) = 9.2 min, t_R(minor) = 9.9 min.

4-(Trifluoromethyl)phenyl (*S*)-7-(1,3-dimethyl-2-oxoindolin-3-yl)heptanoate (3bv).

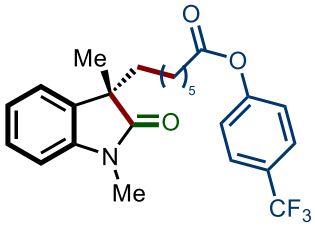

The title compound was isolated as a pale yellow oil in 59% yield (51.1 mg, 85% *ee*) starting from the carbamoyl chloride **1b** (41.9 mg) through column chromatography on silica gel (15% EtOAc in petroleum ether).

^{1}H NMR (500 MHz, Chloroform-*d*) δ 7.56 (d, *J* = 8.4 Hz, 2H), 7.23 – 7.16 (m, 1H), 7.13 – 7.07 (m, 3H), 6.99 (t, *J* = 7.5 Hz, 1H), 6.77 (d, *J* = 7.7 Hz, 1H), 3.14 (s, 3H), 2.42 (t, *J* = 7.5 Hz, 2H), 1.84 (td, *J* = 12.8, 4.6 Hz, 1H), 1.66 (td, *J* = 12.8, 4.4 Hz, 1H), 1.60 – 1.52 (m, 2H), 1.28 (s, 3H), 1.24 – 1.11 (m, 4H), 0.98 – 0.87 (m, 1H), 0.84 – 0.73 (m, 1H).

^{13}C NMR (126 MHz, Chloroform-*d*) δ 180.8, 171.7, 153.2, 143.3, 134.2, 128.0 (q, *J* = 32.7 Hz), 127.7, 126.8 (q, *J* = 4.0 Hz, 2C), 123.9 (q, *J* = 272.3 Hz), 122.48, 122.45, 122.1 (2C), 107.9, 48.4, 38.4, 34.2, 29.3, 28.7, 26.1, 24.7, 24.3, 23.9.

^{19}F NMR (376 MHz, Chloroform-*d*) δ −62.21 (s, 3F).

HRMS (ESI) calcd for $C_{24}H_{27}F_3NO_3^+$ [$(M+H)^+$] 434.1938, found 434.1942.

HPLC-Data: CHIRALPAK AD-H, 25 °C, ^{i}PrOH-hexanes 10/90, 1 mL/min, 254 nm, t_R(major) = 8.2 min, t_R(minor) = 8.9 min.

(*E*)-Hex-2-en-1-yl (*S*)-7-(1,3-dimethyl-2-oxoindolin-3-yl)heptanoate (3bx).

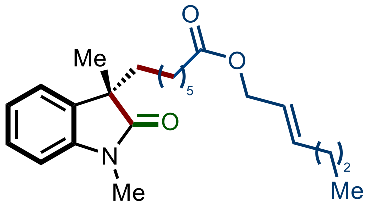

The title compound was isolated as a pale yellow oil in 71% yield (52.6 mg, 85% *ee*) starting from the carbamoyl chloride **1b** (41.9 mg) through column chromatography on silica gel (15% EtOAc in

petroleum ether).

^{1}H NMR (500 MHz, Chloroform-*d*) δ 7.30 – 7.23 (m, 1H), 7.16 (d, J = 7.3 Hz, 1H), 7.06 (t, *J* = 7.4 Hz, 1H), 6.84 (d, *J* = 7.7 Hz, 1H), 5.74 (dt, *J* = 14.7, 6.7 Hz, 1H), 5.54 (dt, *J* = 14.3, 6.6, Hz, 1H), 4.48 (d, *J* = 6.5 Hz, 2H), 3.21 (s, 3H), 2.22 (t, *J* = 7.5 Hz, 2H), 2.02 (q, *J* = 7.2 Hz, 2H), 1.88 (td, *J* = 12.8, 4.6 Hz, 1H), 1.71 (td, *J* = 12.9, 4.4 Hz, 1H), 1.55 – 1.45 (m, 2H), 1.45 – 1.36 (m, 2H), 1.34 (s, 3H), 1.23 – 1.11 (m, 4H), 1.01 – 0.93 (m, 1H), 0.89 (t, *J* = 7.4 Hz, 3H), 0.85 – 0.75 (m, 1H).

^{13}C NMR (126 MHz, Chloroform-*d*) δ 180.8, 173.6, 143.3, 136.3, 134.2, 127.6, 124.0, 122.5 (2C), 107.9, 65.1, 48.4, 38.5, 34.31, 34.25, 29.4, 28.9, 26.1, 24.9, 24.3, 23.8, 22.1, 13.7.

HRMS (ESI) calcd for $C_{23}H_{34}NO_3^+$ [$(M+H)^+$] 372.2533, found 372.2536.

HPLC-Data: CHIRALPAK AD-H, 25 °C, ^{i}PrOH-hexanes 10/90, 1 mL/min, 254 nm, t_R(major) = 5.4 min, t_R(minor) = 5.8 min.

(*S*)-5-(1,3-dimethyl-2-oxoindolin-3-yl)pentyl acetate (3by).

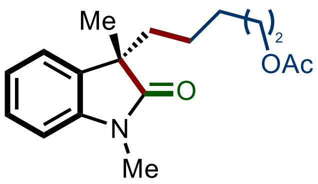

The title compound was isolated as a pale yellow oil in 56% yield (32.2 mg, 84% *ee*) starting from the carbamoyl chloride **1b** (41.9 mg) through column chromatography on silica gel (20% EtOAc in petroleum ether).

^{1}H NMR (400 MHz, Chloroform-*d*) δ 7.31 – 7.24 (m, 1H), 7.16 (ddd, *J* = 7.4, 1.3, 0.6 Hz, 1H), 7.07 (td, *J* = 7.5, 1.0 Hz, 1H), 6.85 (dt, *J* = 7.7, 0.8 Hz, 1H), 3.94 (t, *J* = 6.7 Hz, 2H), 3.22 (s, 3H), 2.00 (s, 3H), 1.90 (td, *J* = 12.5, 4.7 Hz, 1H), 1.73 (td, *J* = 12.5, 4.6 Hz, 1H), 1.52 – 1.43 (m, 2H), 1.35 (s, 3H), 1.29 – 1.13 (m, 2H), 1.08 – 0.95 (m, 1H), 0.90 – 0.79 (m, 1H).

^{13}C NMR (101 MHz, Chloroform-*d*) δ 180.7, 171.1, 143.3, 134.1, 127.7, 122.5, 122.4, 107.9, 64.4, 48.4, 38.3, 28.3, 26.11, 26.05, 24.2, 23.8, 21.0.

HRMS (ESI) calcd for $C_{17}H_{24}NO_3^+$ [$(M+H)^+$] 290.1751, found 290.1756.

HPLC-Data: CHIRALPAK IC, 25 °C, ^{i}PrOH-hexanes 40/60, 1 mL/min, 254 nm, t_R(major) = 11.1 min, t_R(minor) = 12.6 min.

(*S*)-3-(Cyclohexylmethyl)-1,3-dimethylindolin-2-one (3bz).

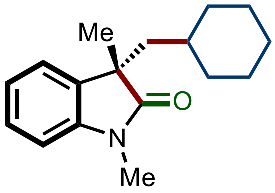

The title compound was isolated as a pale yellow oil in 53% yield (27.3 mg, 84% *ee*) starting from the carbamoyl chloride **1b** (41.9 mg) through column chromatography on silica gel (10% EtOAc in petroleum ether).

^{1}H NMR (500 MHz, Chloroform-*d*) δ 7.30 – 7.23 (m, 1H), 7.16 (d, *J* = 7.3 Hz, 1H), 7.06 (t, *J* = 7.5 Hz, 1H), 6.85 (d, *J* = 7.7 Hz, 1H), 3.22 (s, 3H), 1.93 (dd, *J* = 14.0, 7.0 Hz, 1H), 1.73 (dd, *J* = 14.0, 5.2 Hz, 1H), 1.56 – 1.43 (m, 3H), 1.39 – 1.30 (m, 1H), 1.31 (s, 3H), 1.24 – 1.17 (m, 1H), 1.02 – 0.89 (m, 4H), 0.87 – 0.80 (m, 1H), 0.79 – 0.70 (m, 1H).

^{13}C NMR (126 MHz, Chloroform-*d*) δ 181.2, 143.1, 134.4, 127.5, 122.7, 122.3, 108.0, 47.9, 45.4, 34.8, 34.5, 33.5, 26.22, 26.18, 26.1 (2C), 26.0.

HRMS (ESI) calcd for $C_{17}H_{24}NO^+$ [(M+H)$^+$] 258.1852, found 258.1856.

HPLC-Data: CHIRALPAK AD-H, 25 °C, ^{i}PrOH-hexanes 2/98, 1 mL/min, 254 nm, t_R(major) = 8.5 min, t_R(minor) = 9.3 min.

(*S*)-1,3-Dimethyl-3-((tetrahydro-2*H*-pyran-4-yl)methyl)indolin-2-one (3baa).

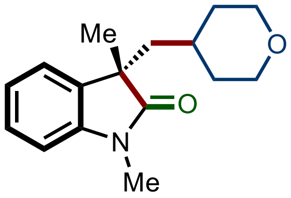

The title compound was isolated as a pale yellow oil in 49% yield (25.5 mg, 83% *ee*) starting from the carbamoyl chloride **1b** (41.9 mg) through column chromatography on silica gel (15% EtOAc in petroleum ether).

^{1}H NMR (400 MHz, Chloroform-*d*) δ 7.28 (t, *J* = 7.7 Hz, 1H), 7.17 (d, *J* = 7.3 Hz, 1H), 7.07 (t, *J* = 7.5 Hz, 1H), 6.86 (d, *J* = 7.7 Hz, 1H), 3.81 – 3.65 (m, 2H), 3.23 (s, 3H), 3.17 – 3.02 (m, 2H), 2.00 (dd, *J* = 14.1, 5.6 Hz, 1H), 1.78 (dd, *J* = 14.1, 4.9 Hz, 1H), 1.34 (s, 3H), 1.28 – 0.96 (m, 5H).

^{13}C NMR (101 MHz, Chloroform-*d*) δ 180.8, 143.0, 134.1, 127.8, 122.7, 122.5, 108.1, 67.72, 67.69, 47.7, 44.9, 34.0, 33.4, 32.2, 26.3, 26.1.

HRMS (ESI) calcd for $C_{16}H_{22}NO_2^+$ [(M+H)$^+$] 260.1645, found 260.1651.

HPLC-Data: CHIRALPAK AD-H, 25 °C, ^{i}PrOH-hexanes 10/90, 1 mL/min, 254 nm,

t_R(major) = 6.7 min, t_R(minor) = 7.2 min.

(*S*)-1,3-Dimethyl-3-phenethylindolin-2-one (3bac).

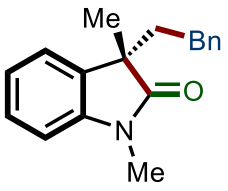

The title compound was isolated as a pale yellow oil in 50% yield (26.6 mg, 85% *ee*) starting from the carbamoyl chloride **1b** (41.9 mg) through column chromatography on silica gel (15% EtOAc in petroleum ether).

^{1}H NMR (500 MHz, Chloroform-*d*) δ 7.30 (t, *J* = 7.7 Hz, 1H), 7.24 – 7.17 (m, 3H), 7.15 – 7.08 (m, 2H), 7.02 (d, *J* = 7.4 Hz, 2H), 6.87 (d, *J* = 7.7 Hz, 1H), 3.21 (s, 3H), 2.34 – 2.22 (m, 2H), 2.17 – 2.08 (m, 1H), 2.06 – 1.97 (m, 1H), 1.39 (s, 3H).

^{13}C NMR (126 MHz, Chloroform-*d*) δ 180.4, 143.5, 141.4, 133.8, 128.31 (2C), 128.26 (2C), 127.9, 125.9, 122.6, 122.5, 108.0, 48.4, 40.3, 31.0, 26.2, 24.0.

HRMS (ESI) calcd for $C_{18}H_{20}NO^+$ [(M+H)$^+$] 266.1539, found 266.1542.

HPLC-Data: CHIRALCEL OD-H, 25 °C, ^{i}PrOH-hexanes 10/90, 1 mL/min, 254 nm, t_R(major) = 7.6 min, t_R(minor) = 6.1 min.

Supplementary Figure 7. Coupling reaction of carbamate 4 with *n*-pentyl iodide (2a)

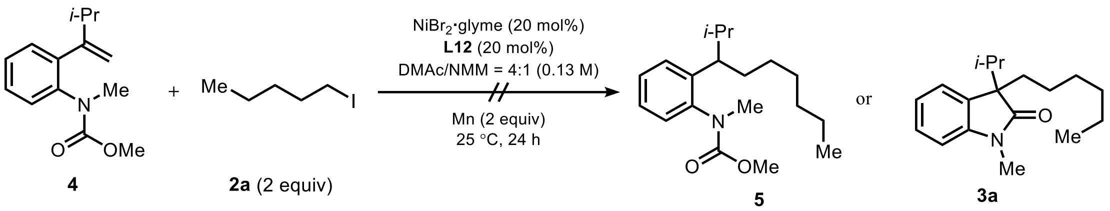

L12 (8.2 mg, 0.04 mmol, 20 mol%) and methyl methyl(2-(3-methylbut-1-en-2-yl)phenyl)carbamate (**4**) (46.7 mg, 0.2 mmol, 1 equiv) were added to a reaction tube equipped with a stir bar. In a nitrogen-filled glovebox, $NiBr_2$ • glyme (12.3 mg, 0.04 mmol, 20 mol%) and manganese dust (22 mg, 0.4 mmol, 2 equiv) were added to the mixture. The reaction tube was sealed and removed from the glovebox. Next, anhydrous DMAc (1.2 mL) and *N*-methyl morpholine (0.3 mL) were added, followed by the addition of 1-iodopentane **2a** (79.2 mg, 0.4 mmol, 2.0 equiv) under the protection of nitrogen. Then the resulting mixture was stirred at 25 °C for 24 h. The reaction was quenched with sat. aq. NH_4Cl solution (5 mL) and diluted with water (10 mL). The aqueous layer was extracted three times with EtOAc, and the combined organic layers were washed with brine (20 mL), dried over $MgSO_4$, filtered, and concentrated under reduced pressure. No reaction occurred to the carbamate **4** according to TLC analysis.

Supplementary Figure 8. Stoichiometric reaction of the carbamoyl chloride 1c with Ni(COD)$_2$

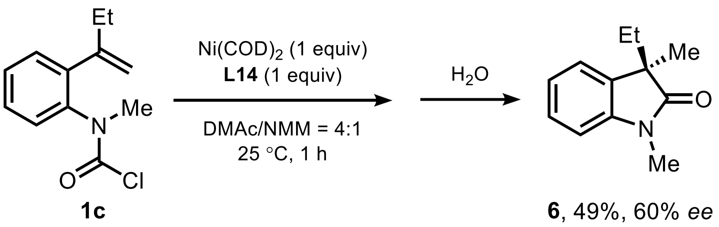

L12 (57.9 mg, 0.2 mmol, 1 equiv) and the carbamic chloride **1c** (44.7 mg, 0.2 mmol, 1 equiv) were added to a reaction tube equipped with a stir bar. Next, anhydrous DMAc (1.2 mL) and *N*-methyl morpholine (0.3 mL) were added under the protection of nitrogen. Subsequently, the reaction mixture was brought into a nitrogen-filled glovebox, and Ni(COD)$_2$ (55 mg, 0.2 mmol, 1 equiv) was added to the mixture. The

reaction tube was sealed and removed from the glovebox. After the resulting mixture was stirred at 25 °C for 1 h, the reaction was quenched with sat. aq. NH_4Cl solution (5 mL) and diluted with water (10 mL). The aqueous layer was extracted three times with EtOAc, and the combined organic layers were washed with brine (20 mL), dried over $MgSO_4$, filtered, and concentrated under reduced pressure. The residue was purified by silica gel column chromatography (petroleum ether/ethyl acetate) to afford the desired product *(R)-3-ethyl-1,3-dimethylindolin-2-on* (**6**) in 49% yield (18.5 mg, 60% *ee*).

(*R*)-3-ethyl-1,3-dimethylindolin-2-one (6)

^{1}H NMR (500 MHz, Chloroform-*d*) δ 7.27 (t, *J* = 7.7 Hz, 1H), 7.17 (d, *J* = 7.3 Hz, 1H), 7.07 (t, *J* = 7.4 Hz, 1H), 6.84 (d, *J* = 7.8 Hz, 1H), 3.22 (s, 3H), 1.93 (dq, *J* = 14.5, 7.4 Hz, 1H), 1.77 (dq, *J* = 14.5, 7.4 Hz, 1H), 1.35 (s, 3H), 0.59 (t, *J* = 7.4 Hz, 3H).

^{13}C NMR (126 MHz, Chloroform-*d*) δ 180.8, 143.5, 134.0, 127.6, 122.5, 122.4, 107.8, 49.0, 31.5, 26.1, 23.3, 8.9.

HRMS (ESI) calcd for $C_{12}H_{16}NO^+$ $[(M+H)^+]$ 190.1226, found 190.1226.

HPLC-Data: CHIRALCEL OJ-H, 25 °C, ^{i}PrOH-hexanes 2/98, 1 mL/min, 254 nm, t_R(major) = 7.2 min, t_R(minor) = 7.7 min.

Supplementary Figure 9. Coupling reaction of the carbamoyl chloride 7 with *n*-pentyl iodide (2a)

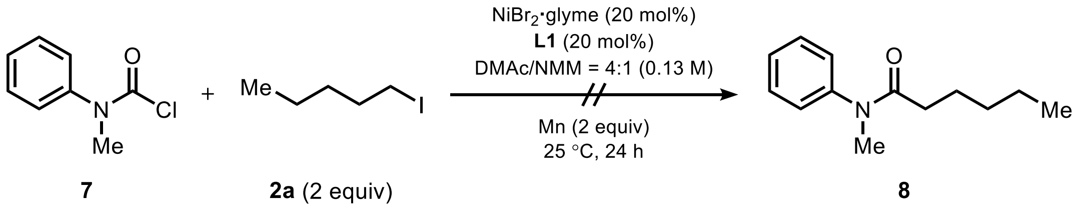

Racemic **L1** (8.2 mg, 0.04 mmol, 20 mol%) and methyl(phenyl)carbamic chloride (**7**) (33.9 mg, 0.2 mmol, 1 equiv) were added to a reaction tube equipped with a stir bar and brought into a nitrogen-filled glovebox. $NiBr_2$ • glyme (12.3 mg, 0.04 mmol, 20 mol%) and manganese dust (22 mg, 0.4 mmol, 2 equiv) were added to the mixture. The reaction tube was sealed and removed from the glovebox. Next, anhydrous DMAc (1.2 mL) and *N*-methyl morpholine (0.3 mL) were added, followed by the

addition of 1-iodopentane **2a** (79.2 mg, 0.4 mmol, 2.0 equiv) under the protection of nitrogen. After the resulting mixture was stirred at 25 °C for 24 h, the reaction was quenched with sat. aq. NH_4Cl solution (5 mL) and further diluted with water (10 mL). The aqueous layer was extracted three times with EtOAc, and the combined organic layers were washed with brine (20 mL), dried over $MgSO_4$, filtered, and concentrated under reduced pressure. The formation of the cross-coupling product **8** was not observed.

Supplementary Figure 10. Radical clock experiment of the carbamoyl chloride 1a with 6-iodohex-1-ene (2ai)

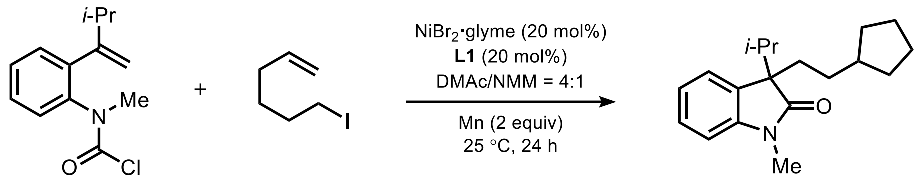

1a **2ai** (2 equiv) **3aai** (22%)

Racemic **L1** (20 mol%) and the carbamic chloride **1a** (47.5 mg, 0.2 mmol, 1 equiv) were added to a reaction tube equipped with a stir bar and brought into a nitrogen-filled glovebox. $NiBr_2$ • glyme (12.3 mg, 0.04 mmol, 20 mol%) and manganese dust (22 mg, 0.4 mmol, 2 equiv) were added to the mixture. The reaction tube was sealed and removed from the glovebox. Next, anhydrous DMAc (1.2 mL) and *N*-methyl morpholine (0.3 mL) were added, followed by the addition of 6-iodohex-1-ene **2ai** (84.0 mg, 0.4 mmol, 2.0 equiv) under the protection of nitrogen. After the resulting mixture was stirred at 25 °C for 24 h, the reaction was quenched with sat. aq. NH_4Cl solution (5 mL) and further diluted with water (10 mL). The aqueous layer was extracted three times with EtOAc, and the combined organic layers were washed with brine (20 mL), dried over $MgSO_4$, filtered, and concentrated under reduced pressure. The residue was purified by silica gel column chromatography (petroleum ether/ethyl acetate) to afford *3-(2-cyclopentylethyl)-3-isopropyl-1-methyl indolin-2-one* (**3aai**) as a pale yellow oil in 22% yield (12.6 mg).

^{1}H NMR (500 MHz, Chloroform-*d*) δ 7.30 – 7.24 (m, 1H), 7.16 (d, *J* = 7.4 Hz, 1H), 7.05 (t, *J* = 7.5 Hz, 1H), 6.82 (d, *J* = 7.7 Hz, 1H), 3.20 (s, 3H), 2.20 – 2.06 (m, 1H), 1.95 – 1.79 (m, 3H), 1.71 – 1.55 (m, 2H), 1.53 – 1.38 (m, 2H), 1.25 – 1.08 (m, 3H),

1.04 – 0.81 (m, 2H), 0.96 (d, J = 6.8 Hz, 3H), 0.77 – 0.62 (m, 1H), 0.67 (d, J = 6.6 Hz, 3H).

HRMS (ESI) calcd for $C_{19}H_{27}NONa^+$ [$(M+Na)^+$] 308.1985, found 308.1992.

Supplementary Figure 11. Determination of the Absolute Configuration of the Carbo-Acylation Products

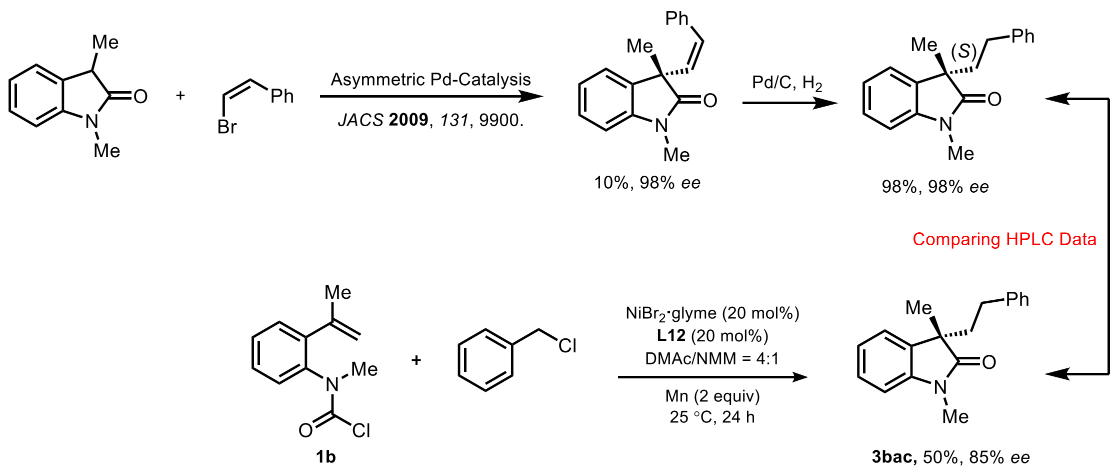

The carbamoyl chloride **1b** was subjected to the alkyl-carbamoylation with benzyl chloride under the standard reaction conditions, affording the oxindole **3bac** as the product. It is known in the literature that the compound **3bac** with *S*-configuration can be synthesized through Pd-catalyzed asymmetric alkenylation of an oxindole followed by hydrogenation.[11] The absolute configuration of compound **3bac** prepared using our method was determined to be *S* through comparison of the HPLC data with these reported in the literature.[a] The absolute stereochemistry of all the other alkyl-carbamoylation products were assigned assuming a common reaction pathway.

[a] **Our HPLC-Data**: CHIRALCEL OD-H, 25 °C, ^{i}PrOH-hexanes 10/90, 1 mL/min, 254 nm, t_R(major) = 7.6 min, t_R(minor) = 6.1 min.

Reported HPLC-Data: CHIRALCEL OD-H, 25 °C, ^{i}PrOH-hexanes 10/90, 1 mL/min, 254 nm, t_R(major) = 7.4 min, t_R(minor) = 6.1 min.

Supplementary Figure 12

Chromatogram

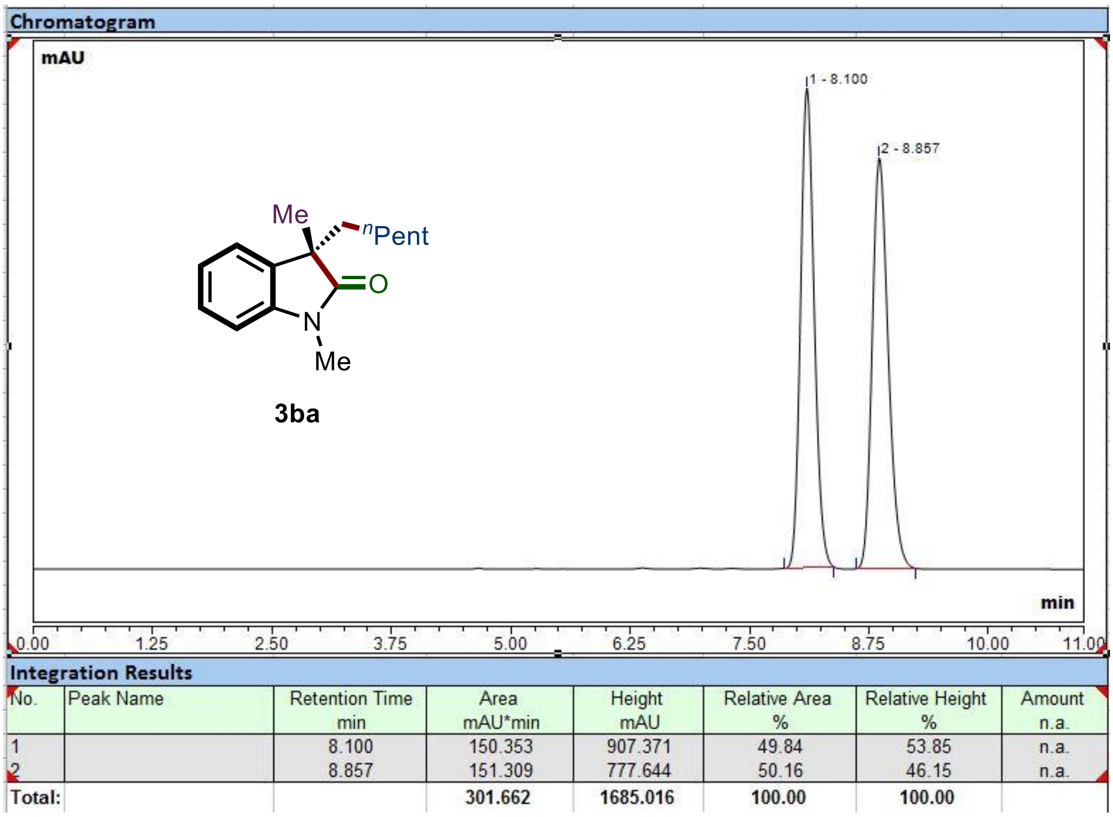

Integration Results

No.	Peak Name	Retention Time<br>min	Area<br>mAU*min	Height<br>mAU	Relative Area<br>%	Relative Height<br>%	Amount<br>n.a.
1		8.100	150.353	907.371	49.84	53.85	n.a.
2		8.857	151.309	777.644	50.16	46.15	n.a.
Total:			**301.662**	**1685.016**	**100.00**	**100.00**	

Chromatogram

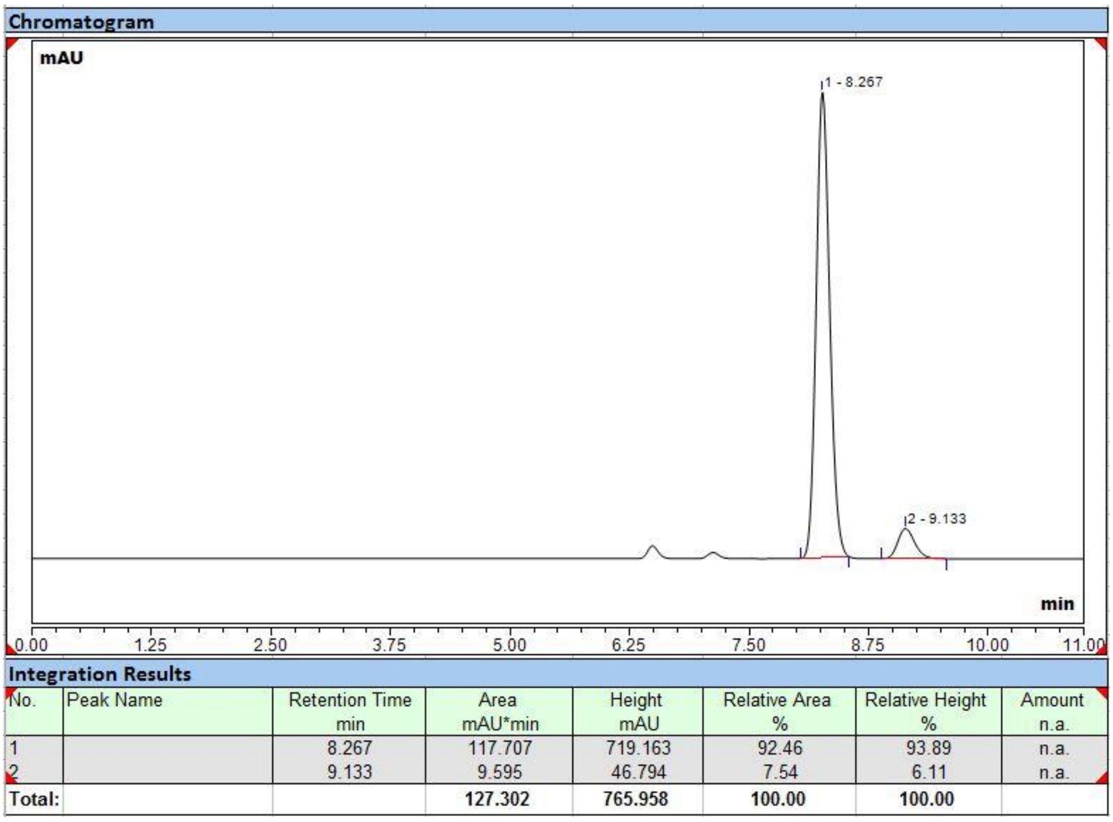

Integration Results

No.	Peak Name	Retention Time<br>min	Area<br>mAU*min	Height<br>mAU	Relative Area<br>%	Relative Height<br>%	Amount<br>n.a.
1		8.267	117.707	719.163	92.46	93.89	n.a.
2		9.133	9.595	46.794	7.54	6.11	n.a.
Total:			**127.302**	**765.958**	**100.00**	**100.00**	

Supplementary Figure 13

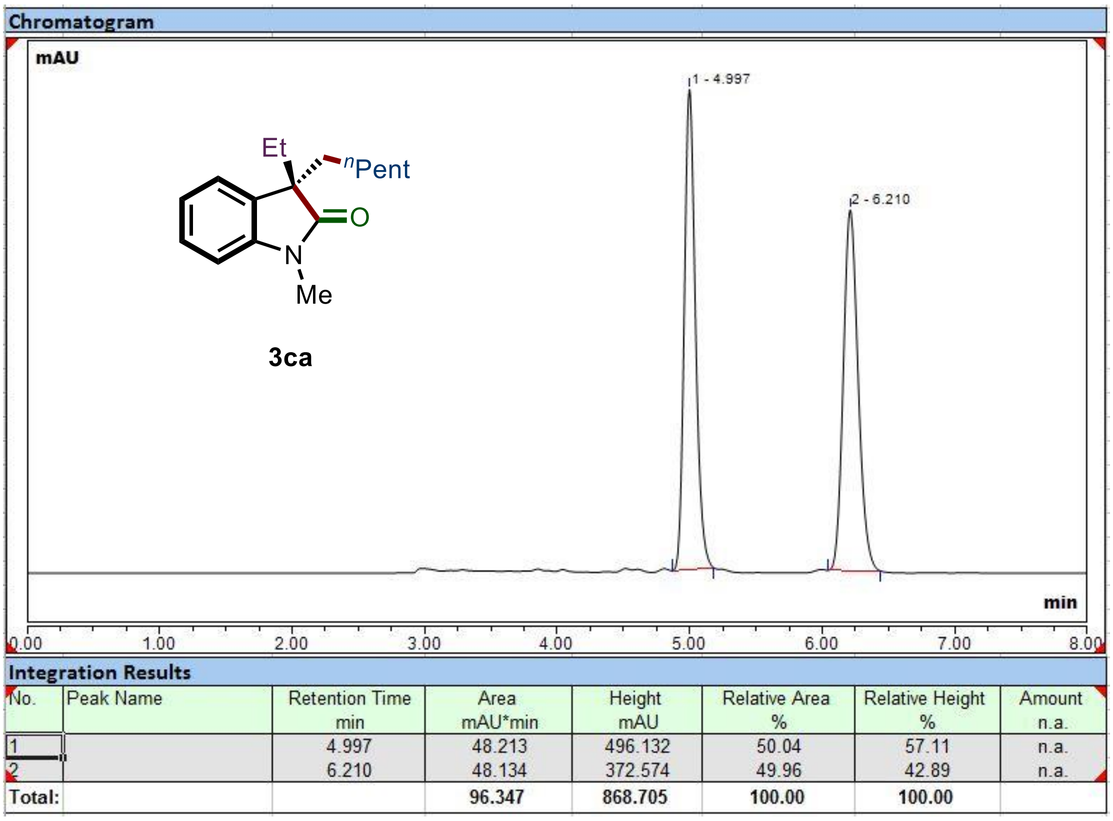

Integration Results

No.	Peak Name	Retention Time min	Area mAU*min	Height mAU	Relative Area %	Relative Height %	Amount n.a.
1		4.997	48.213	496.132	50.04	57.11	n.a.
2		6.210	48.134	372.574	49.96	42.89	n.a.
**Total:**			**96.347**	**868.705**	**100.00**	**100.00**	

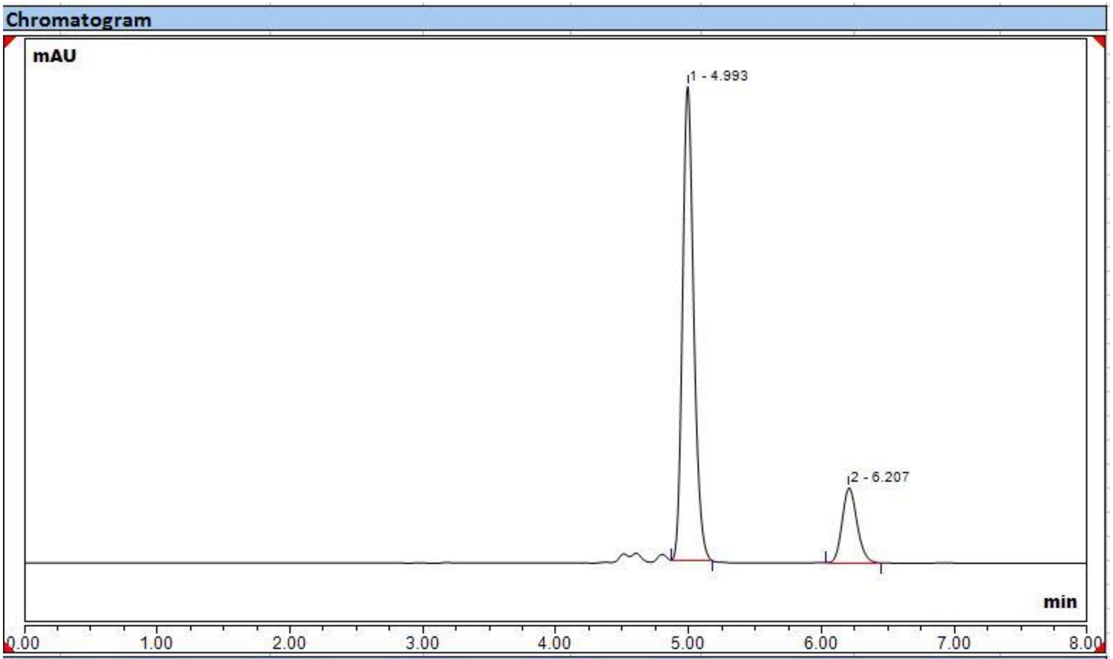

Integration Results

No.	Peak Name	Retention Time min	Area mAU*min	Height mAU	Relative Area %	Relative Height %	Amount n.a.
1		4.993	15.869	162.845	82.60	86.32	n.a.
2		6.207	3.342	25.813	17.40	13.68	n.a.
**Total:**			**19.211**	**188.657**	**100.00**	**100.00**	

Supplementary Figure 14.

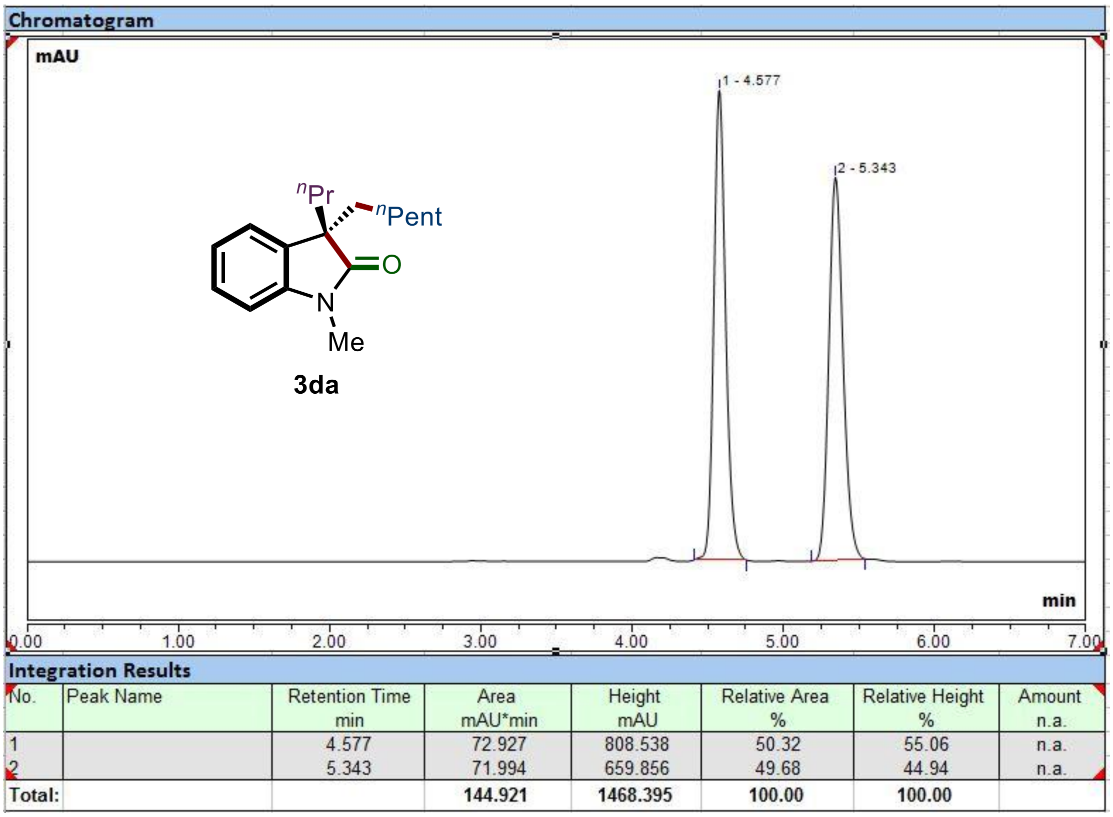

Integration Results

No.	Peak Name	Retention Time<br>min	Area<br>mAU*min	Height<br>mAU	Relative Area<br>%	Relative Height<br>%	Amount<br>n.a.
1		4.577	72.927	808.538	50.32	55.06	n.a.
2		5.343	71.994	659.856	49.68	44.94	n.a.
**Total:**			**144.921**	**1468.395**	**100.00**	**100.00**	

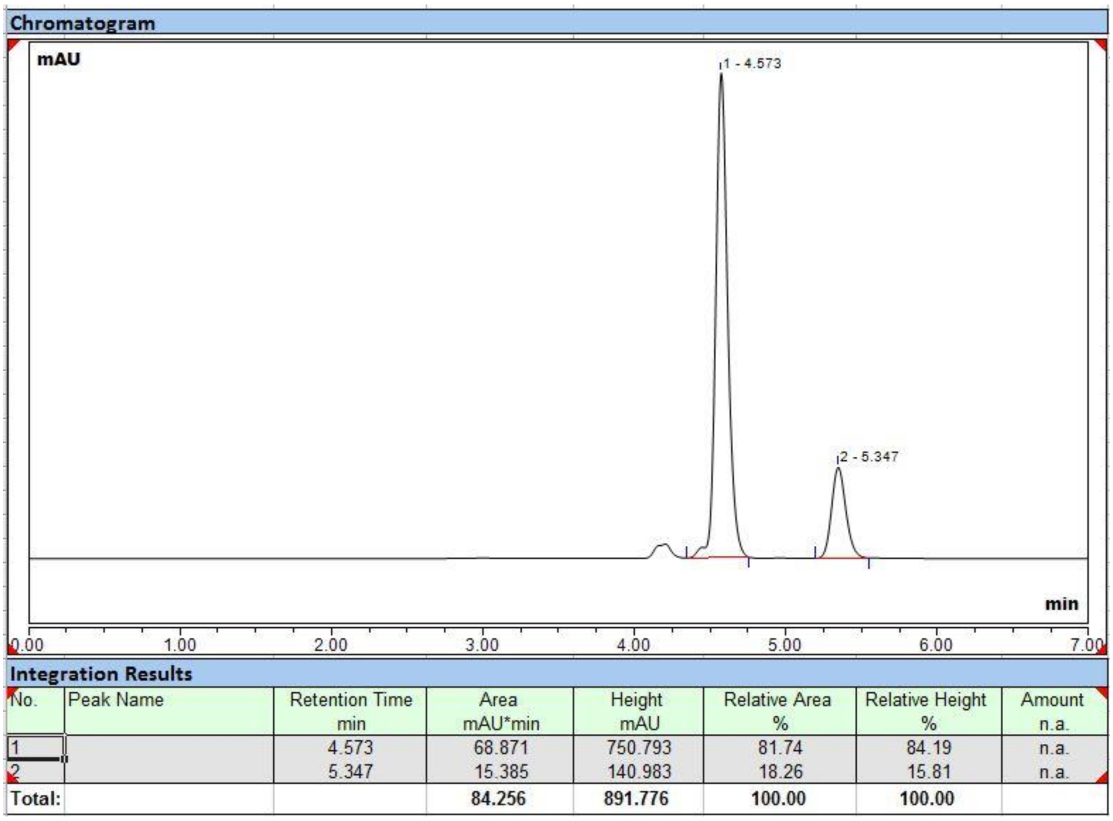

Integration Results

No.	Peak Name	Retention Time<br>min	Area<br>mAU*min	Height<br>mAU	Relative Area<br>%	Relative Height<br>%	Amount<br>n.a.
1		4.573	68.871	750.793	81.74	84.19	n.a.
2		5.347	15.385	140.983	18.26	15.81	n.a.
**Total:**			**84.256**	**891.776**	**100.00**	**100.00**	

Supplementary Figure 15.

Chromatogram

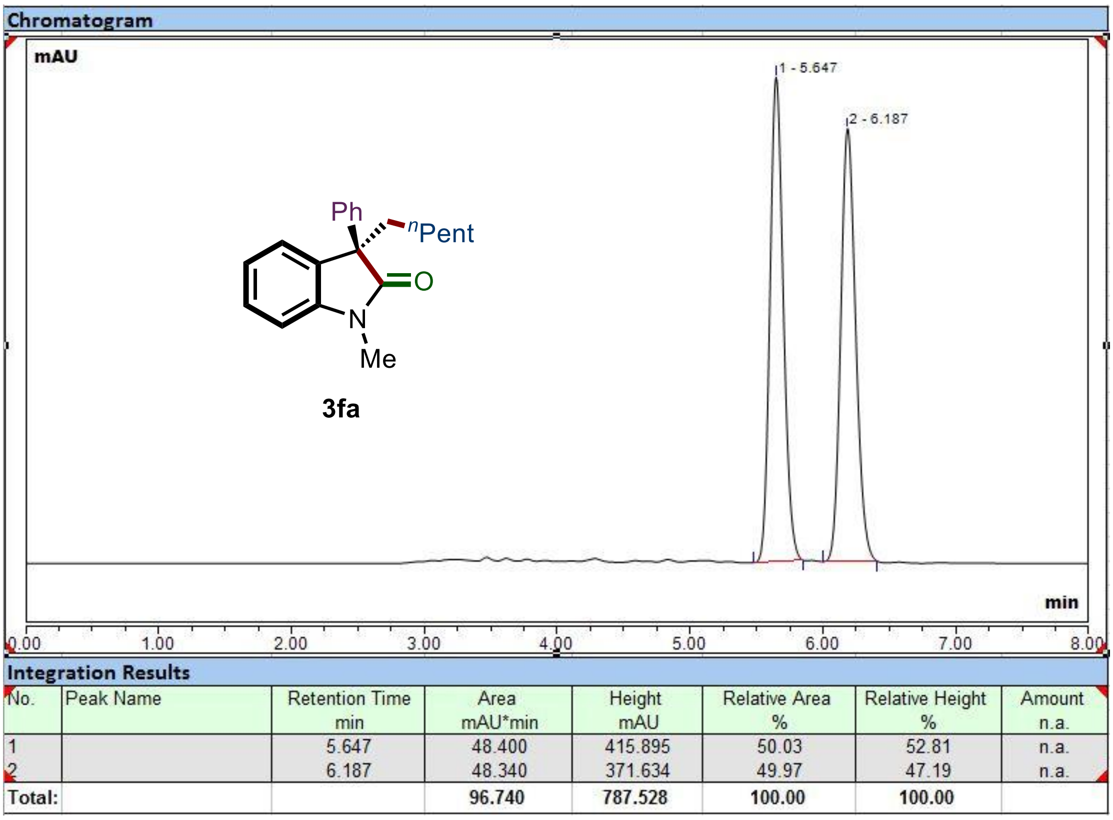

Integration Results

No.	Peak Name	Retention Time min	Area mAU*min	Height mAU	Relative Area %	Relative Height %	Amount n.a.
1		5.647	48.400	415.895	50.03	52.81	n.a.
2		6.187	48.340	371.634	49.97	47.19	n.a.
Total:			**96.740**	**787.528**	**100.00**	**100.00**	

Chromatogram

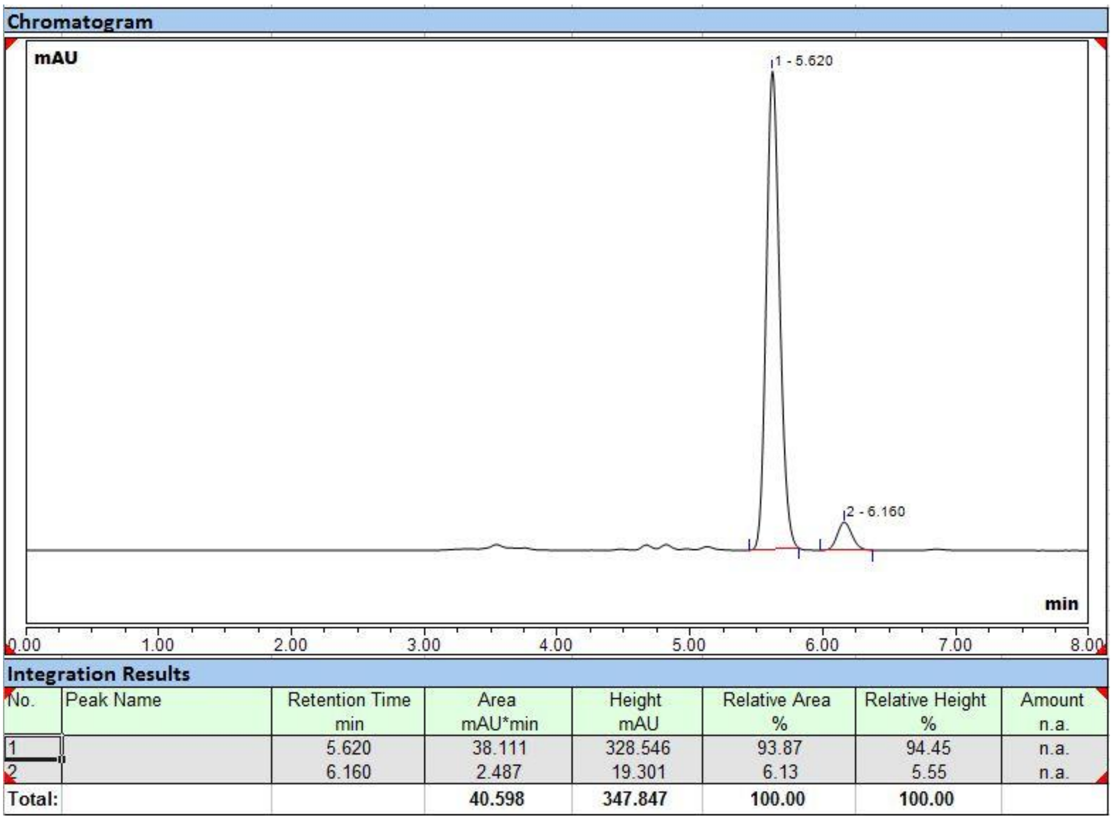

Integration Results

No.	Peak Name	Retention Time min	Area mAU*min	Height mAU	Relative Area %	Relative Height %	Amount n.a.
1		5.620	38.111	328.546	93.87	94.45	n.a.
2		6.160	2.487	19.301	6.13	5.55	n.a.
Total:			**40.598**	**347.847**	**100.00**	**100.00**	

Supplementary Figure 16.

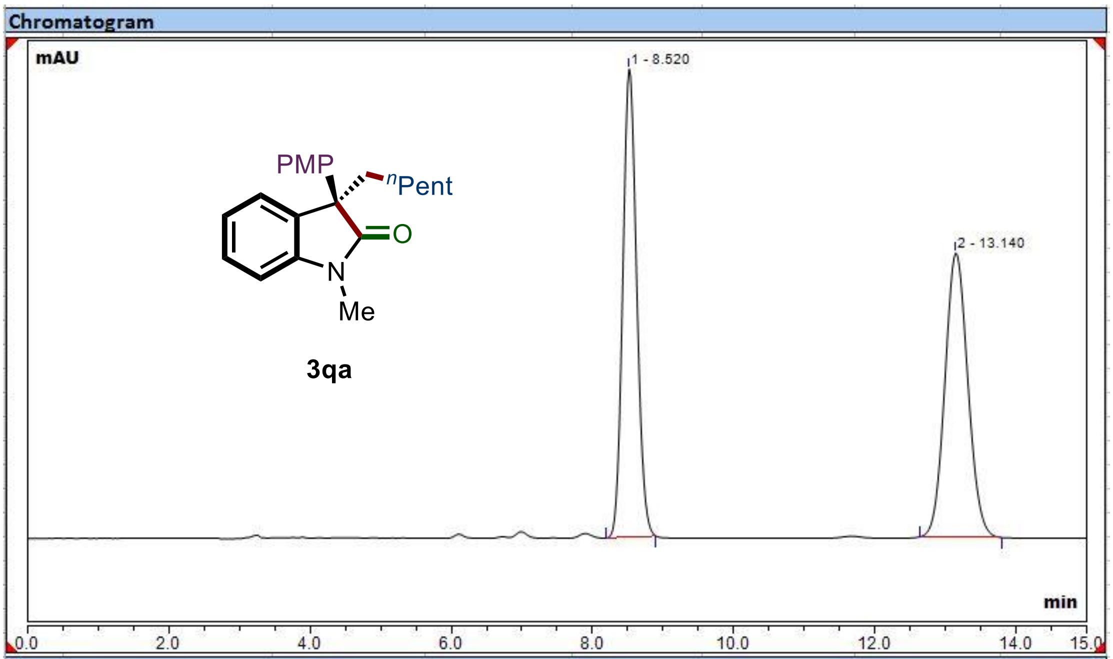

Integration Results

No.	Peak Name	Retention Time min	Area mAU*min	Height mAU	Relative Area %	Relative Height %	Amount n.a.
1		8.520	25.627	112.896	49.89	62.21	n.a.
2		13.140	25.740	68.570	50.11	37.79	n.a.
Total:			**51.367**	**181.466**	**100.00**	**100.00**	

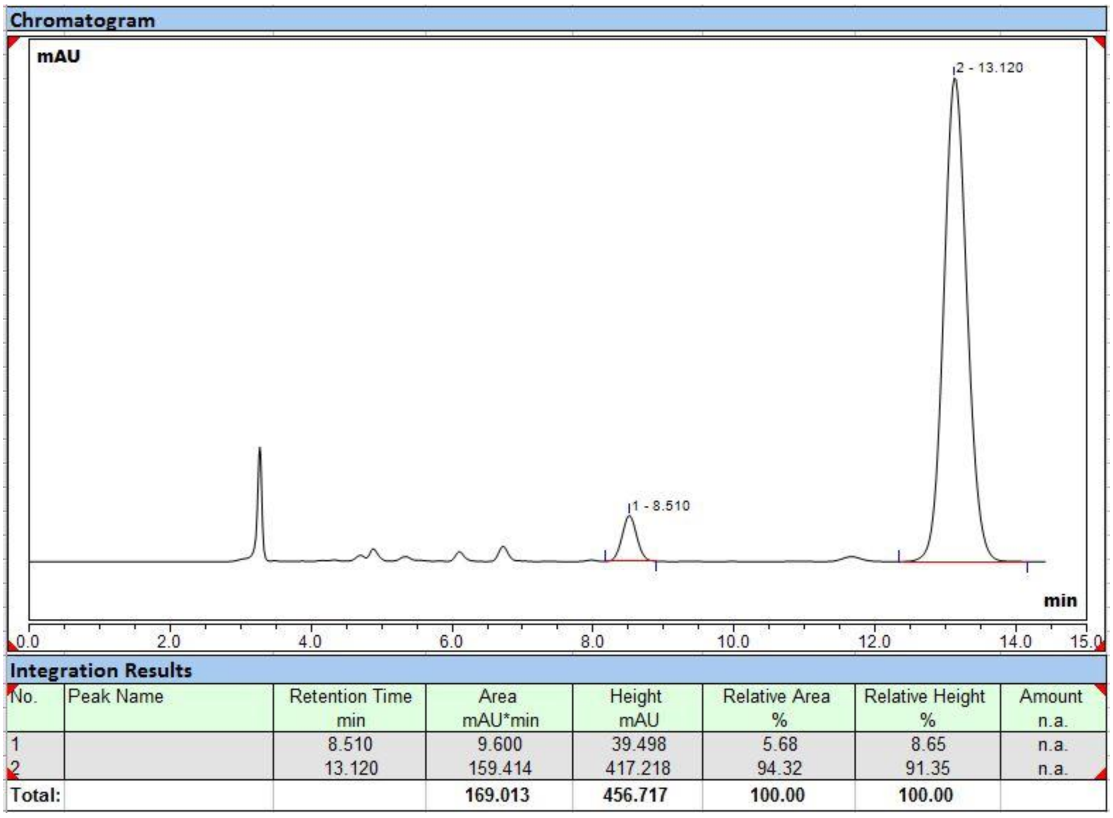

Integration Results

No.	Peak Name	Retention Time min	Area mAU*min	Height mAU	Relative Area %	Relative Height %	Amount n.a.
1		8.510	9.600	39.498	5.68	8.65	n.a.
2		13.120	159.414	417.218	94.32	91.35	n.a.
Total:			**169.013**	**456.717**	**100.00**	**100.00**	

Supplementary Figure 17.

Chromatogram

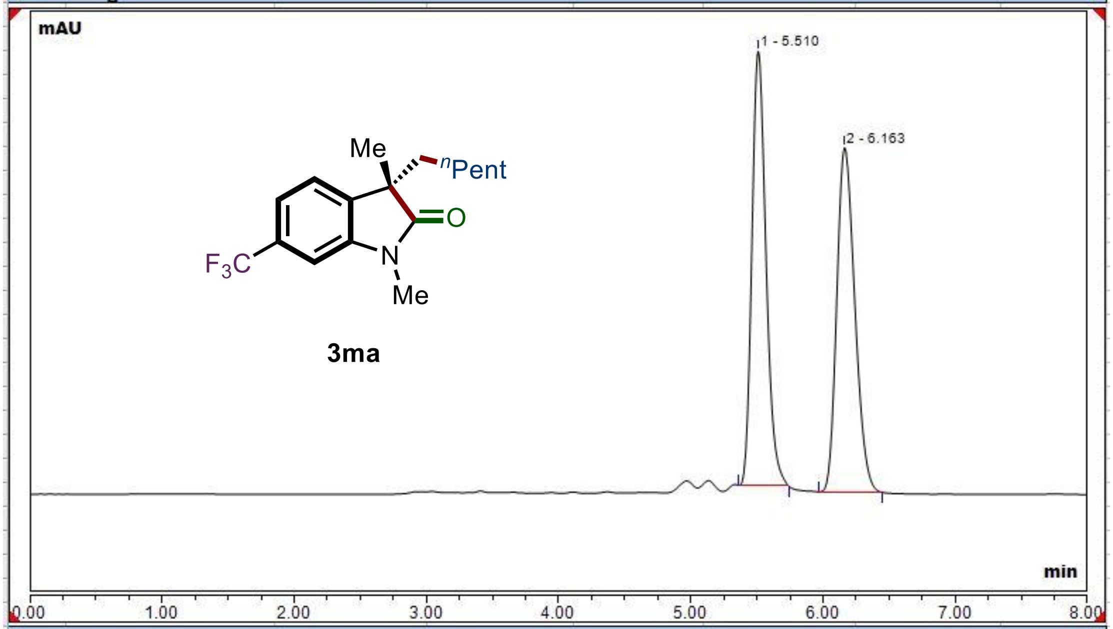

Integration Results

No.	Peak Name	Retention Time<br>min	Area<br>mAU*min	Height<br>mAU	Relative Area<br>%	Relative Height<br>%	Amount<br>n.a.
1		5.510	27.716	224.925	49.57	55.77	n.a.
2		6.163	28.199	178.386	50.43	44.23	n.a.
Total:			55.915	403.311	100.00	100.00	

Chromatogram

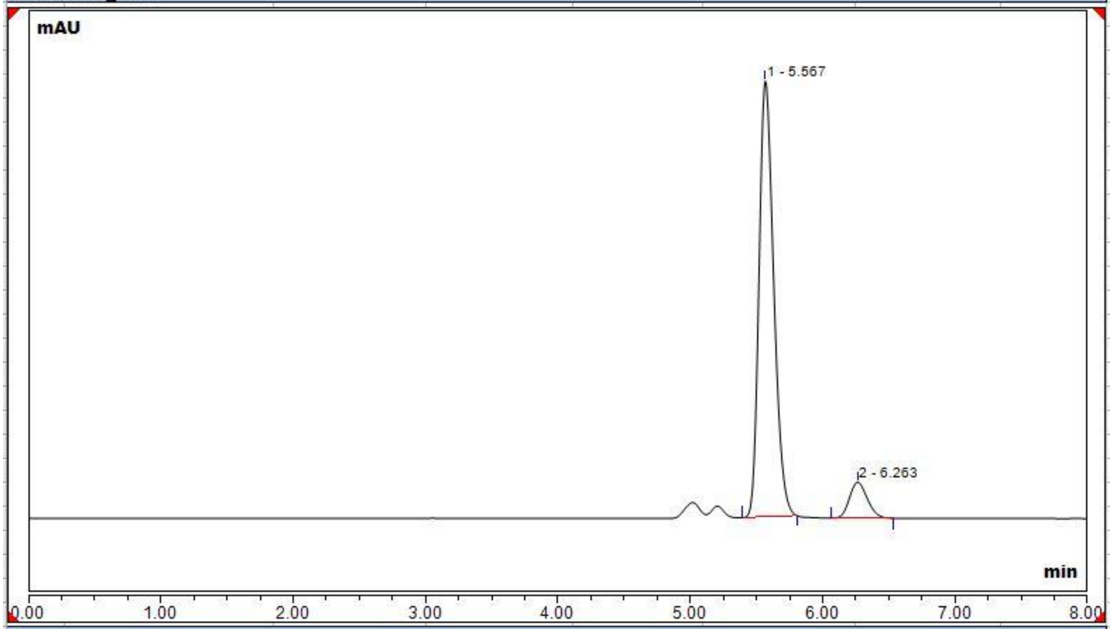

Integration Results

No.	Peak Name	Retention Time<br>min	Area<br>mAU*min	Height<br>mAU	Relative Area<br>%	Relative Height<br>%	Amount<br>n.a.
1		5.567	77.144	600.773	90.83	92.36	n.a.
2		6.263	7.784	49.693	9.17	7.64	n.a.
Total:			84.928	650.466	100.00	100.00	

Supplementary Figure 18.

Chromatogram

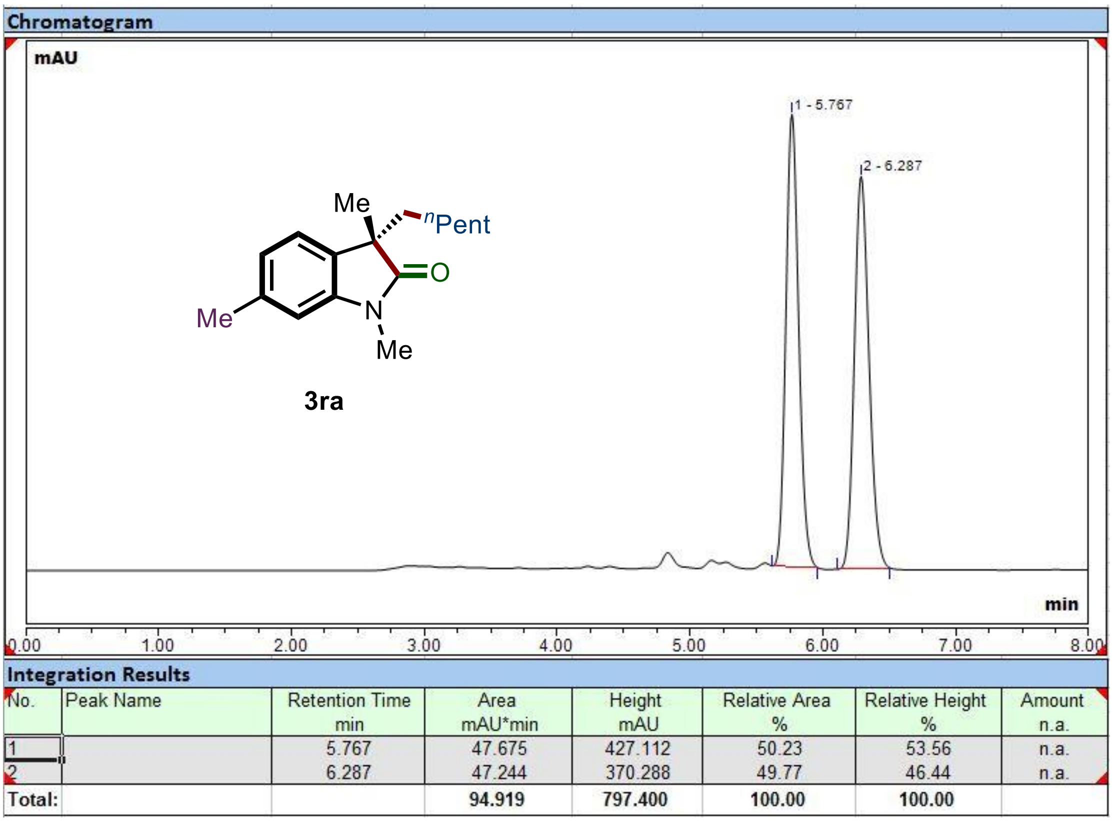

Integration Results

No.	Peak Name	Retention Time<br>min	Area<br>mAU*min	Height<br>mAU	Relative Area<br>%	Relative Height<br>%	Amount<br>n.a.
1		5.767	47.675	427.112	50.23	53.56	n.a.
2		6.287	47.244	370.288	49.77	46.44	n.a.
Total:			**94.919**	**797.400**	**100.00**	**100.00**	

Chromatogram

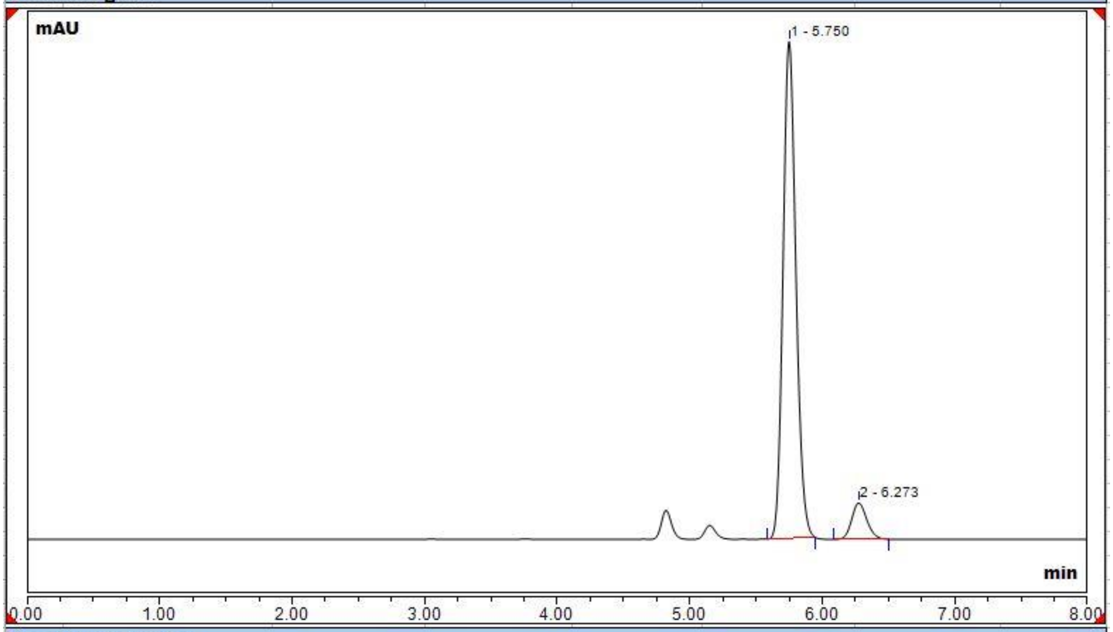

Integration Results

No.	Peak Name	Retention Time<br>min	Area<br>mAU*min	Height<br>mAU	Relative Area<br>%	Relative Height<br>%	Amount<br>n.a.
1		5.750	52.796	470.435	92.07	93.17	n.a.
2		6.273	4.545	34.498	7.93	6.83	n.a.
Total:			**57.341**	**504.933**	**100.00**	**100.00**	

Supplementary Figure 19.

Chromatogram

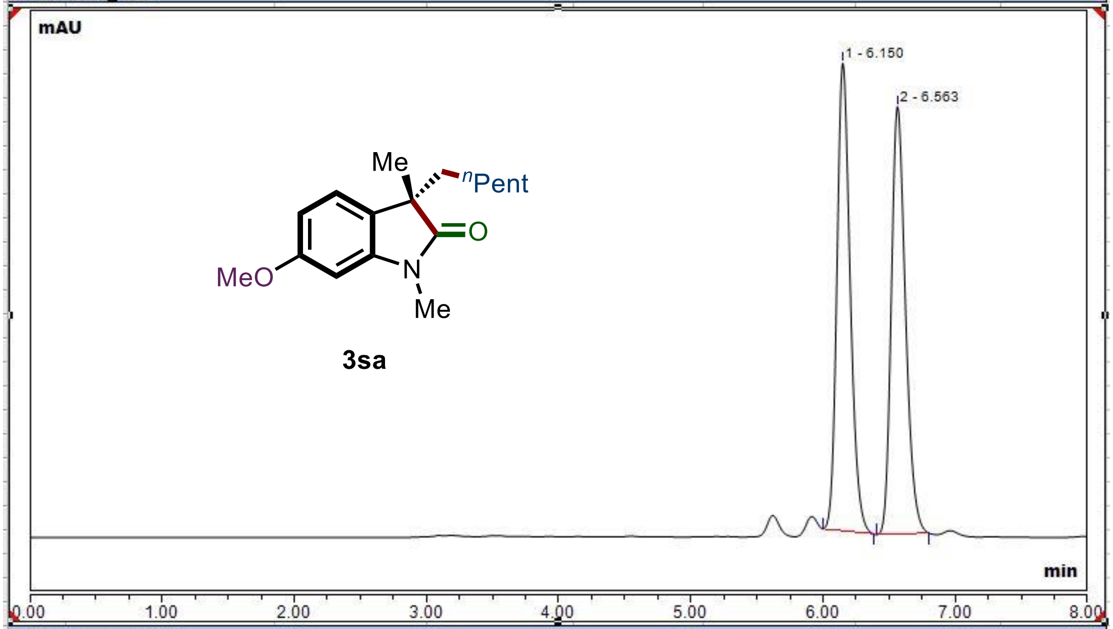

Integration Results

No.	Peak Name	Retention Time min	Area mAU*min	Height mAU	Relative Area %	Relative Height %	Amount n.a.
1		6.150	20.643	177.556	50.60	52.25	n.a.
2		6.563	20.152	162.252	49.40	47.75	n.a.
**Total:**			**40.796**	**339.807**	**100.00**	**100.00**	

Chromatogram

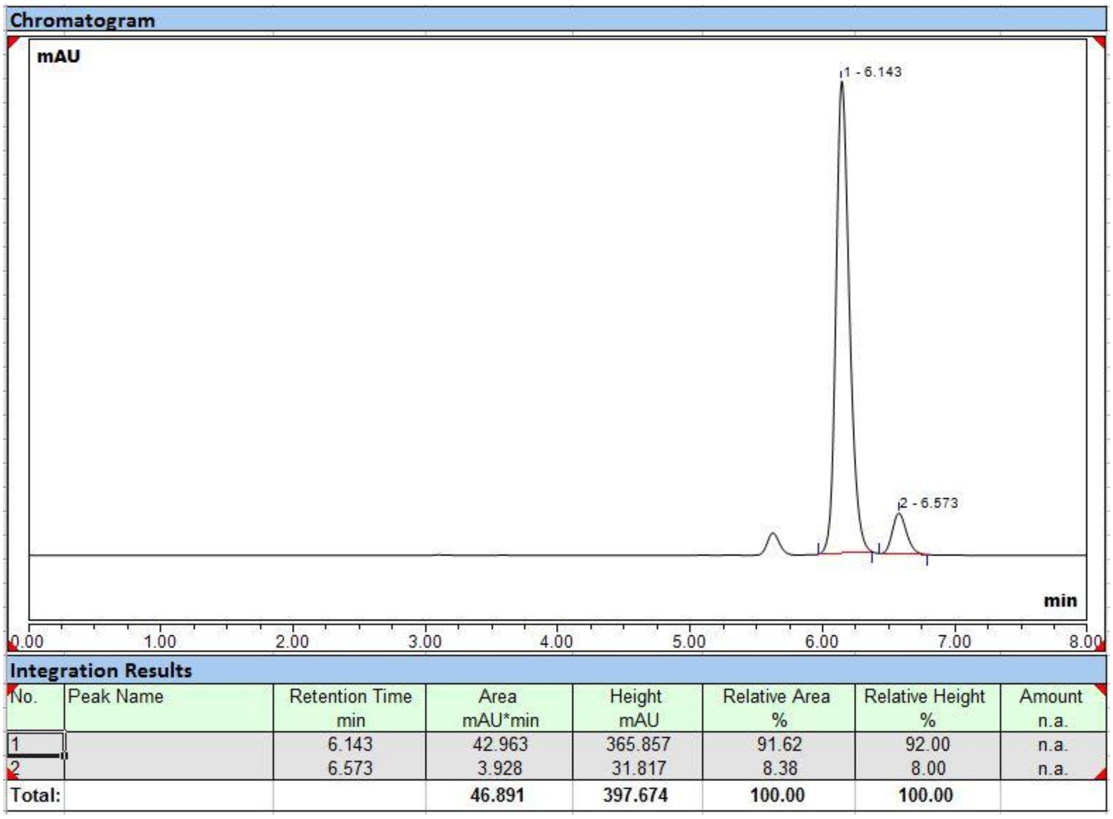

Integration Results

No.	Peak Name	Retention Time min	Area mAU*min	Height mAU	Relative Area %	Relative Height %	Amount n.a.
1		6.143	42.963	365.857	91.62	92.00	n.a.
2		6.573	3.928	31.817	8.38	8.00	n.a.
**Total:**			**46.891**	**397.674**	**100.00**	**100.00**	

Supplementary Figure 20.

Chromatogram

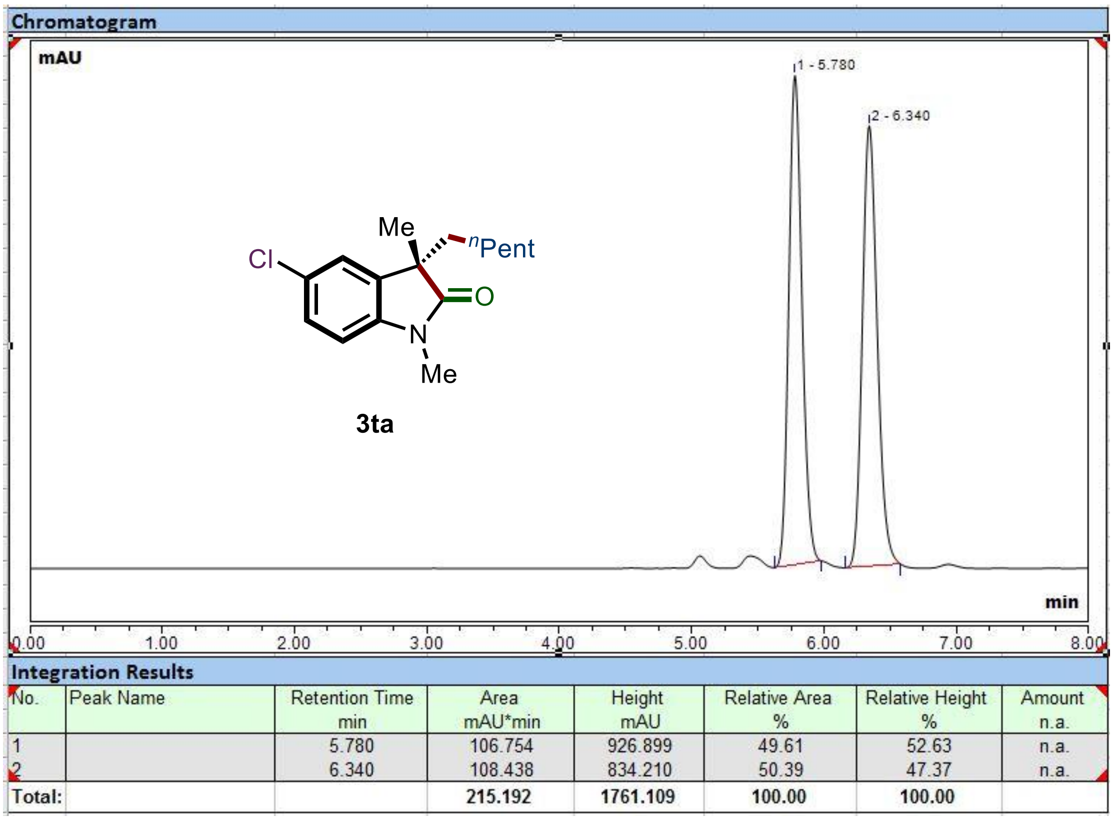

Integration Results

No.	Peak Name	Retention Time min	Area mAU*min	Height mAU	Relative Area %	Relative Height %	Amount n.a.
1		5.780	106.754	926.899	49.61	52.63	n.a.
2		6.340	108.438	834.210	50.39	47.37	n.a.
Total:			**215.192**	**1761.109**	**100.00**	**100.00**	

Chromatogram

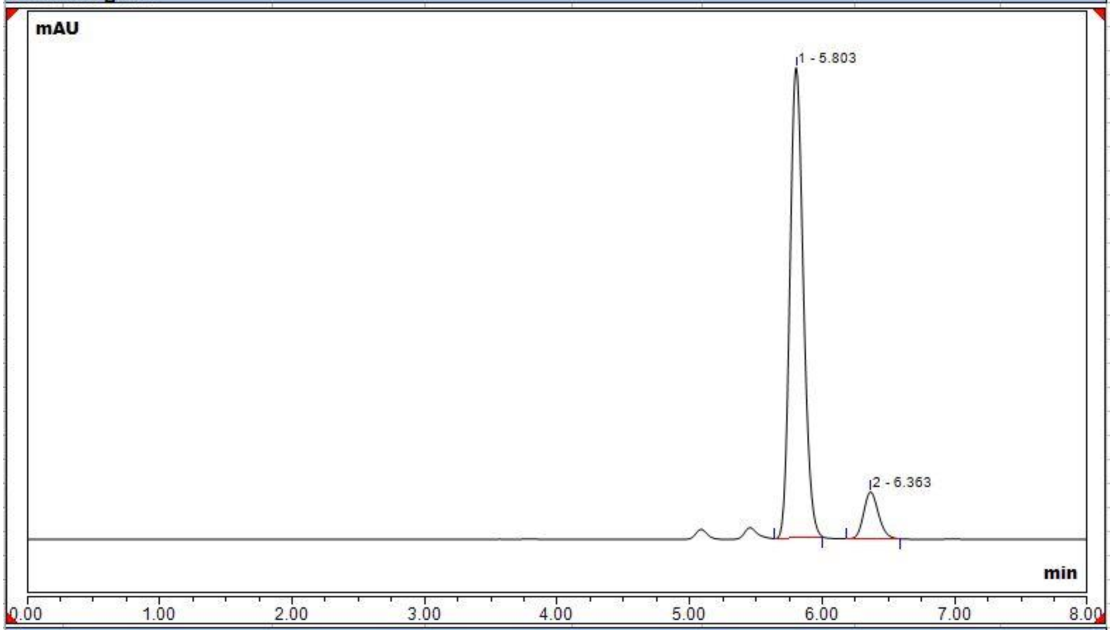

Integration Results

No.	Peak Name	Retention Time min	Area mAU*min	Height mAU	Relative Area %	Relative Height %	Amount n.a.
1		5.803	208.674	1780.801	90.11	90.93	n.a.
2		6.363	22.914	177.659	9.89	9.07	n.a.
Total:			**231.588**	**1958.460**	**100.00**	**100.00**	

Supplementary Figure 21.

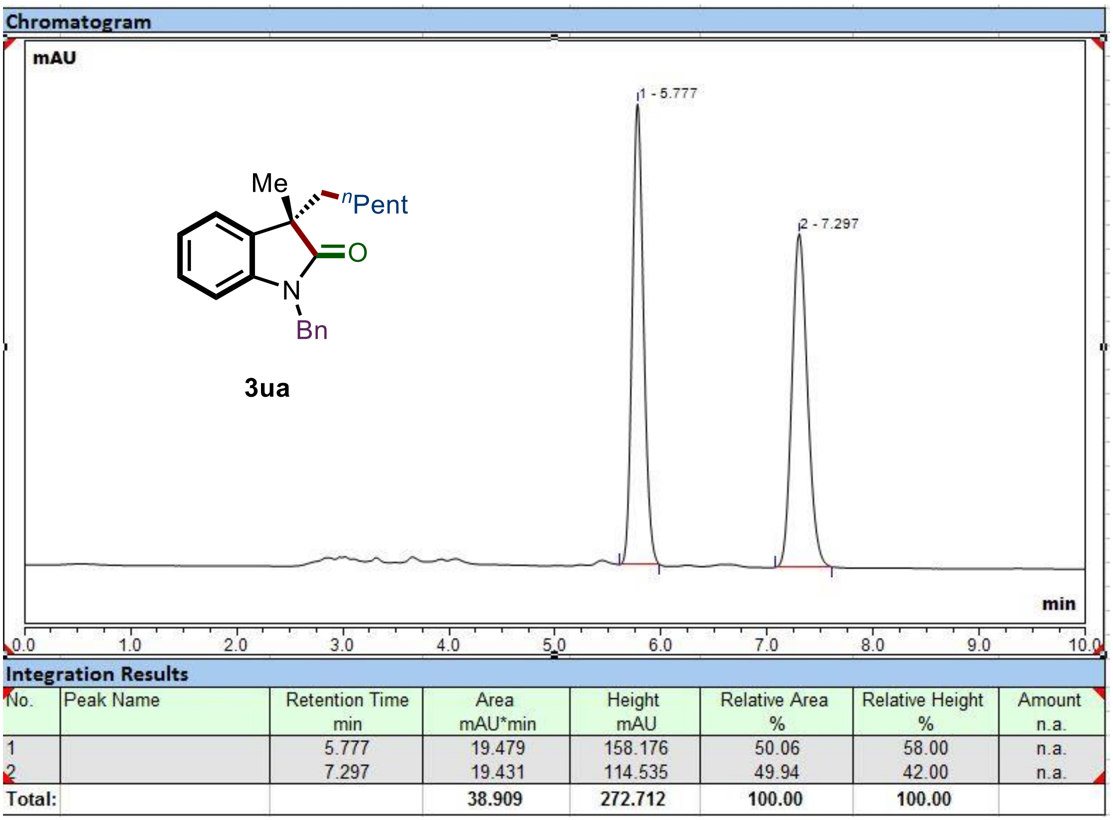

Integration Results

No.	Peak Name	Retention Time min	Area mAU*min	Height mAU	Relative Area %	Relative Height %	Amount n.a.
1		5.777	19.479	158.176	50.06	58.00	n.a.
2		7.297	19.431	114.535	49.94	42.00	n.a.
Total:			38.909	272.712	100.00	100.00	

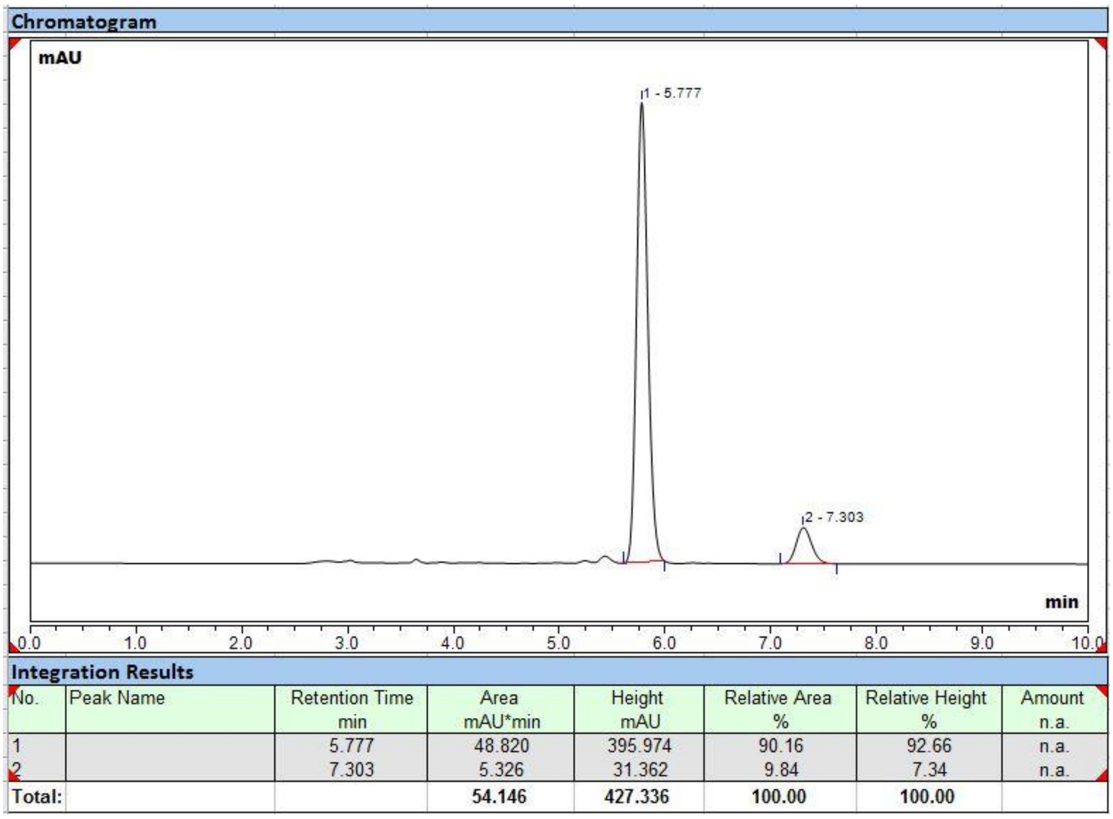

Integration Results

No.	Peak Name	Retention Time min	Area mAU*min	Height mAU	Relative Area %	Relative Height %	Amount n.a.
1		5.777	48.820	395.974	90.16	92.66	n.a.
2		7.303	5.326	31.362	9.84	7.34	n.a.
Total:			54.146	427.336	100.00	100.00	

Supplementary Figure 22.

Chromatogram

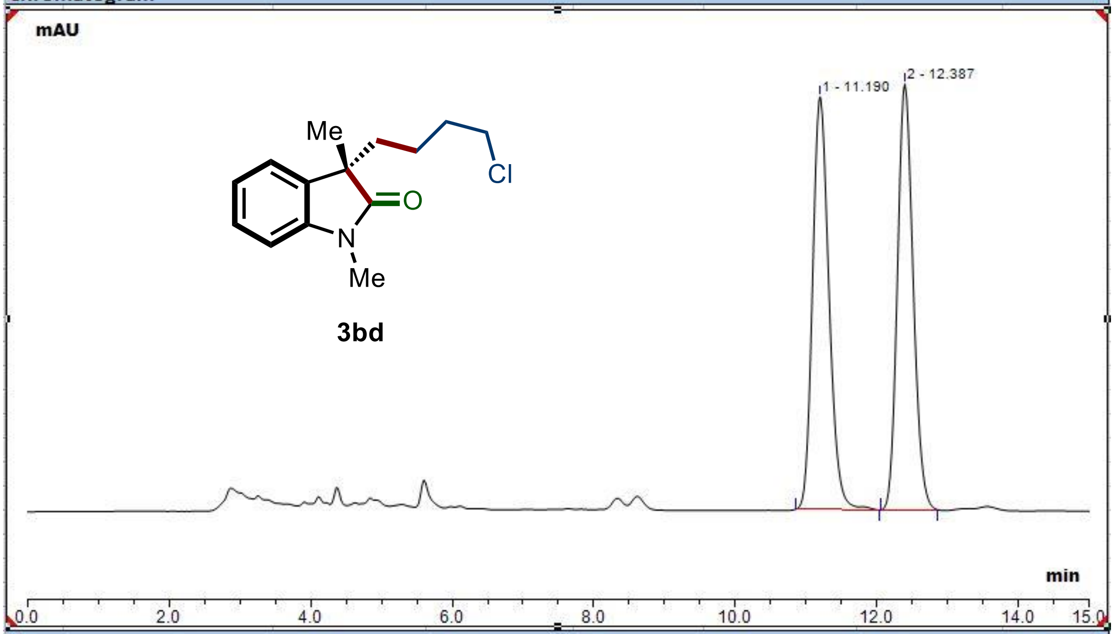

Integration Results

No.	Peak Name	Retention Time<br>min	Area<br>mAU*min	Height<br>mAU	Relative Area<br>%	Relative Height<br>%	Amount<br>n.a.
1		11.190	26.684	99.264	50.35	49.20	n.a.
2		12.387	26.309	102.474	49.65	50.80	n.a.
Total:			**52.994**	**201.738**	**100.00**	**100.00**	

Chromatogram

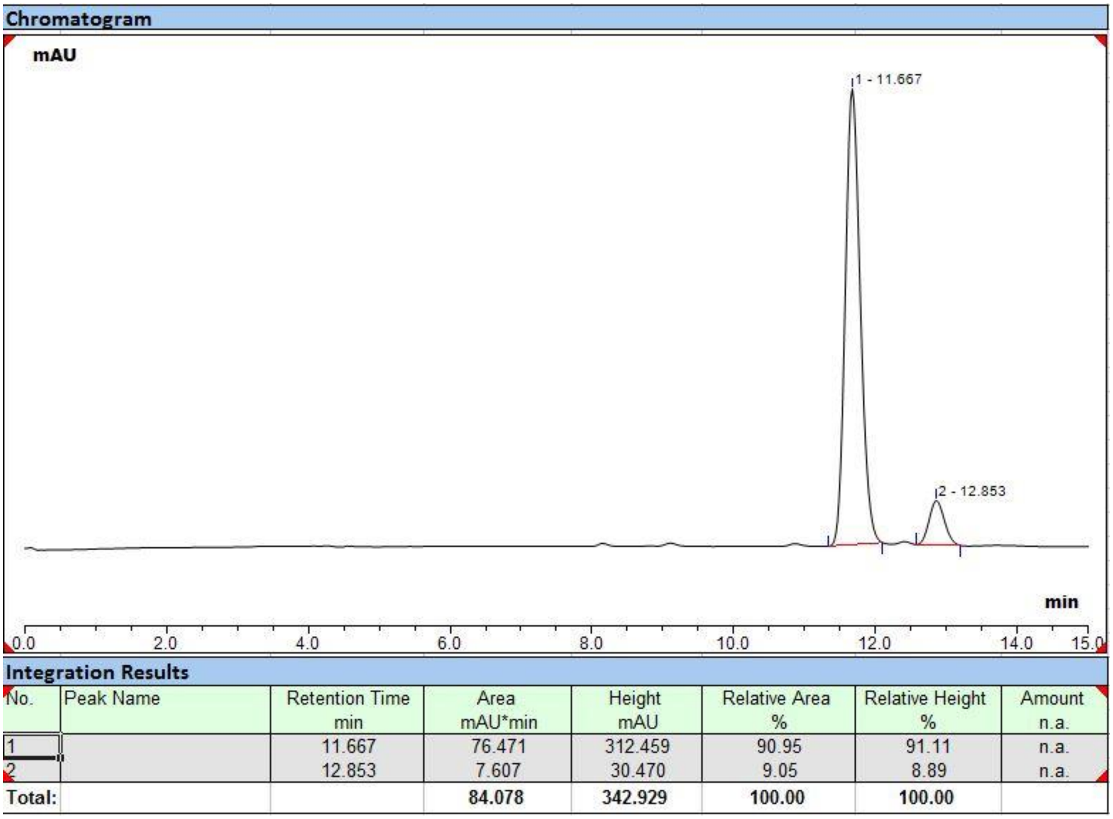

Integration Results

No.	Peak Name	Retention Time<br>min	Area<br>mAU*min	Height<br>mAU	Relative Area<br>%	Relative Height<br>%	Amount<br>n.a.
1		11.667	76.471	312.459	90.95	91.11	n.a.
2		12.853	7.607	30.470	9.05	8.89	n.a.
Total:			**84.078**	**342.929**	**100.00**	**100.00**	

Supplementary Figure 23.

Chromatogram

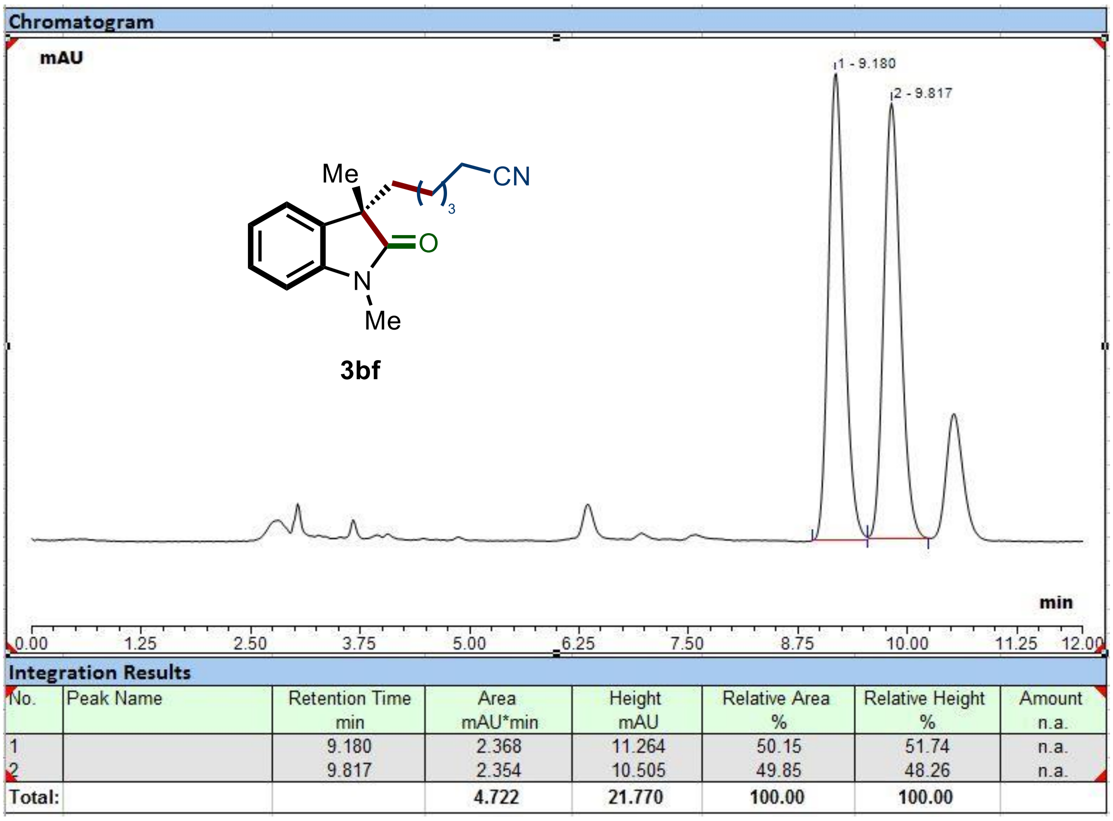

Integration Results

No.	Peak Name	Retention Time min	Area mAU*min	Height mAU	Relative Area %	Relative Height %	Amount n.a.
1		9.180	2.368	11.264	50.15	51.74	n.a.
2		9.817	2.354	10.505	49.85	48.26	n.a.
Total:			**4.722**	**21.770**	**100.00**	**100.00**	

Chromatogram

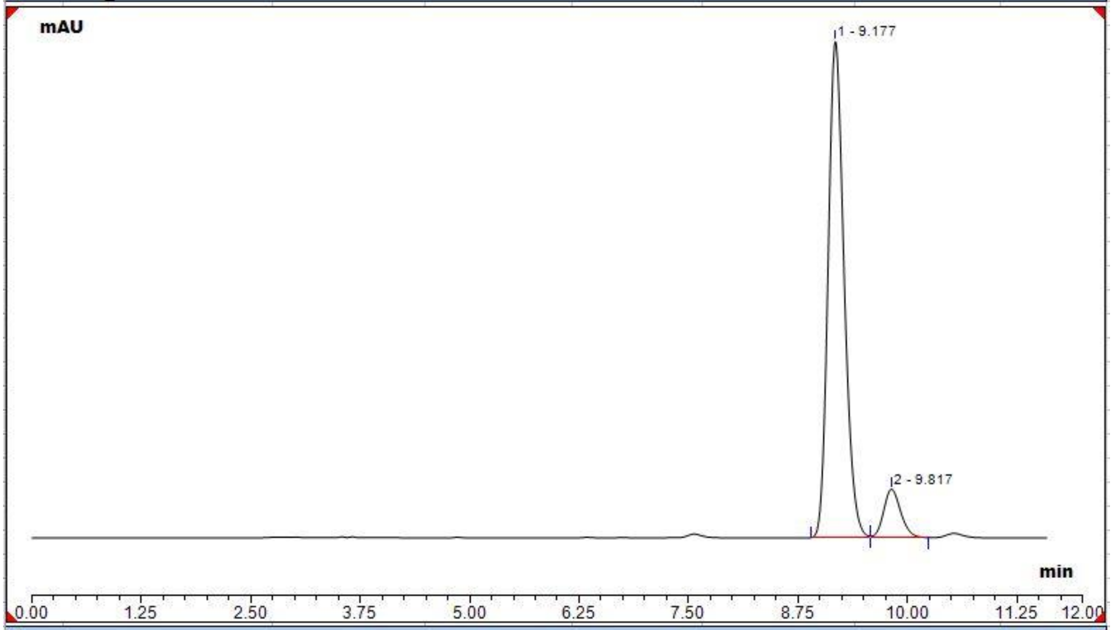

Integration Results

No.	Peak Name	Retention Time min	Area mAU*min	Height mAU	Relative Area %	Relative Height %	Amount n.a.
1		9.177	99.282	469.865	90.57	91.10	n.a.
2		9.817	10.340	45.908	9.43	8.90	n.a.
Total:			**109.621**	**515.774**	**100.00**	**100.00**	

Supplementary Figure 24.

Chromatogram

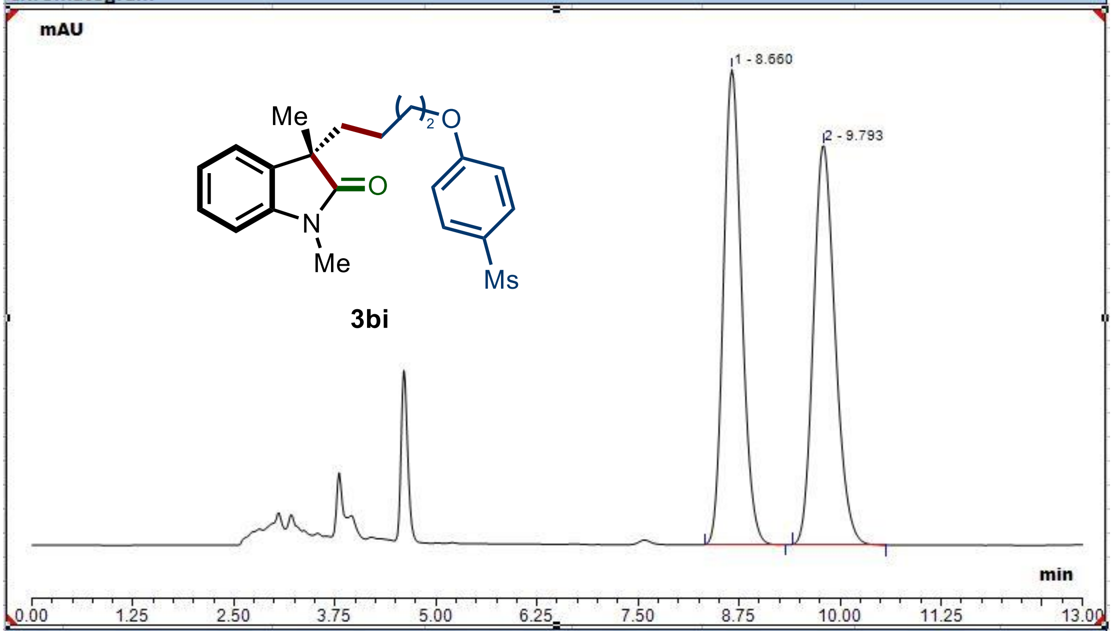

Integration Results

No.	Peak Name	Retention Time min	Area mAU*min	Height mAU	Relative Area %	Relative Height %	Amount n.a.
1		8.660	51.147	196.104	49.97	54.31	n.a.
2		9.793	51.205	164.983	50.03	45.69	n.a.
Total:			**102.351**	**361.087**	**100.00**	**100.00**	

Chromatogram

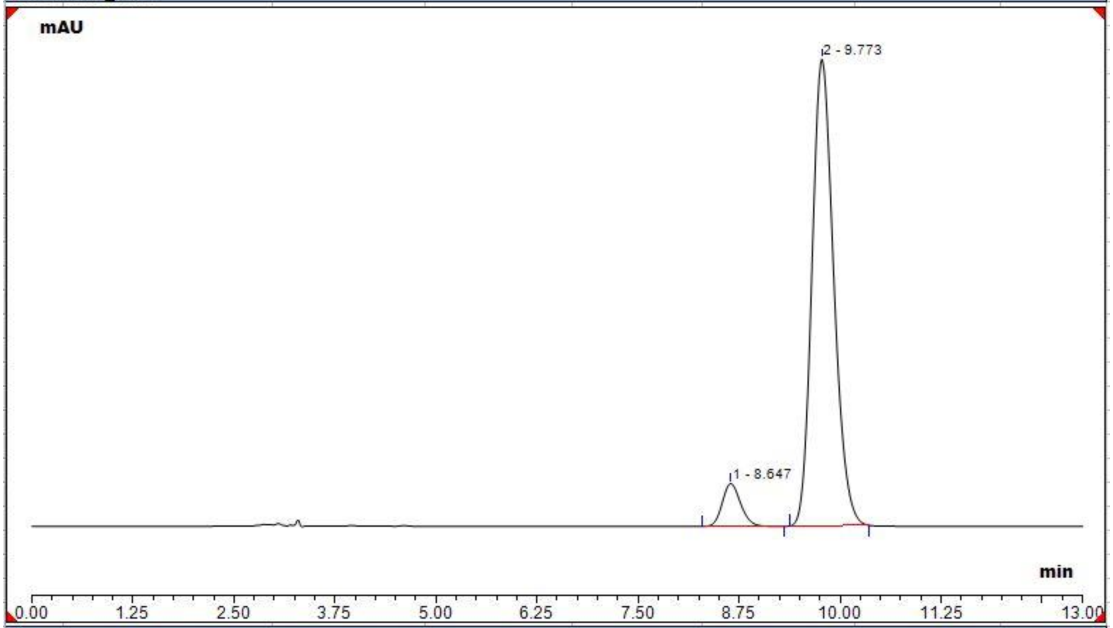

Integration Results

No.	Peak Name	Retention Time min	Area mAU*min	Height mAU	Relative Area %	Relative Height %	Amount n.a.
1		8.647	3.490	13.286	7.20	8.41	n.a.
2		9.773	45.008	144.630	92.80	91.59	n.a.
Total:			**48.498**	**157.915**	**100.00**	**100.00**	

Supplementary Figure 25.

Chromatogram

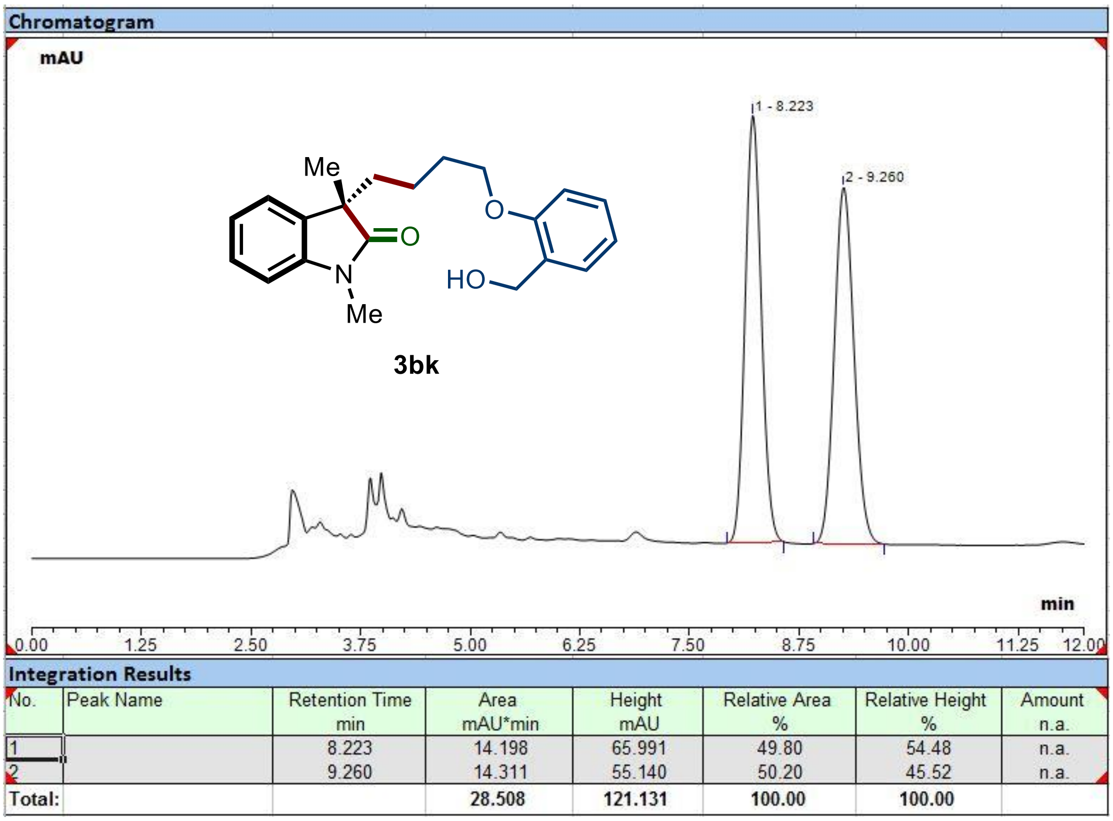

Integration Results

No.	Peak Name	Retention Time min	Area mAU*min	Height mAU	Relative Area %	Relative Height %	Amount n.a.
1		8.223	14.198	65.991	49.80	54.48	n.a.
2		9.260	14.311	55.140	50.20	45.52	n.a.
Total:			**28.508**	**121.131**	**100.00**	**100.00**	

Chromatogram

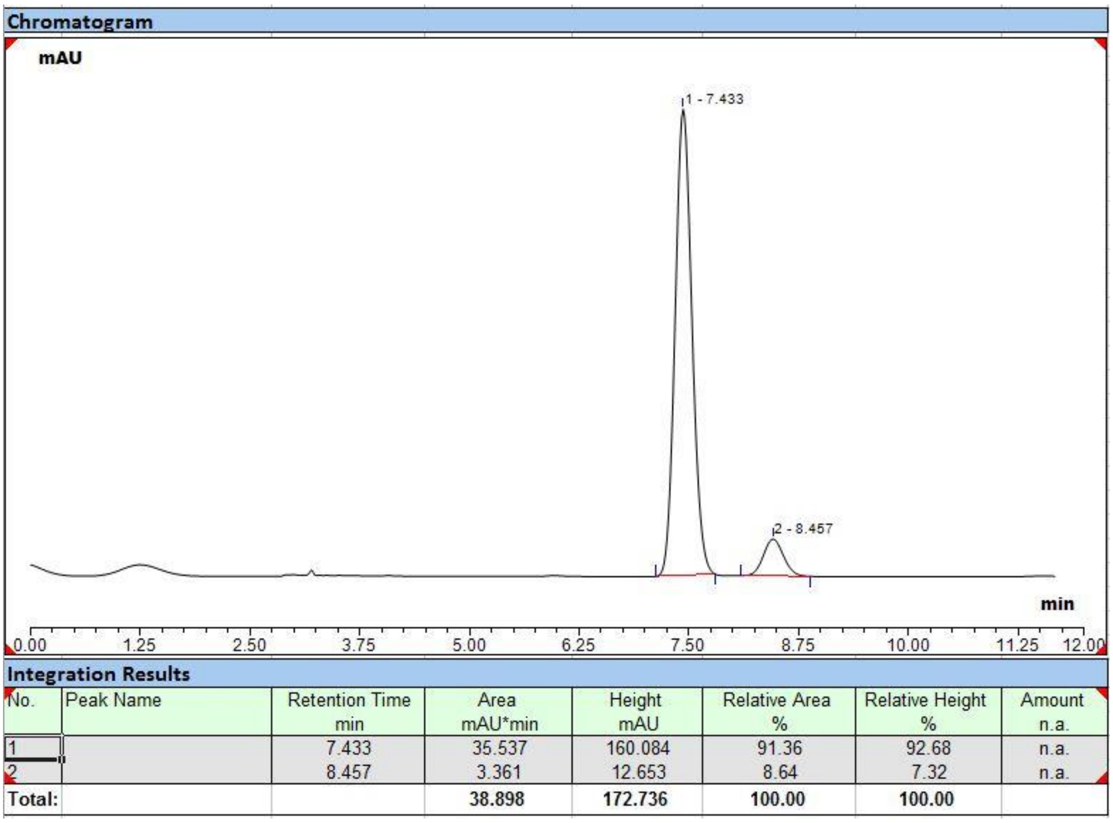

Integration Results

No.	Peak Name	Retention Time min	Area mAU*min	Height mAU	Relative Area %	Relative Height %	Amount n.a.
1		7.433	35.537	160.084	91.36	92.68	n.a.
2		8.457	3.361	12.653	8.64	7.32	n.a.
Total:			**38.898**	**172.736**	**100.00**	**100.00**	

Supplementary Figure 26.

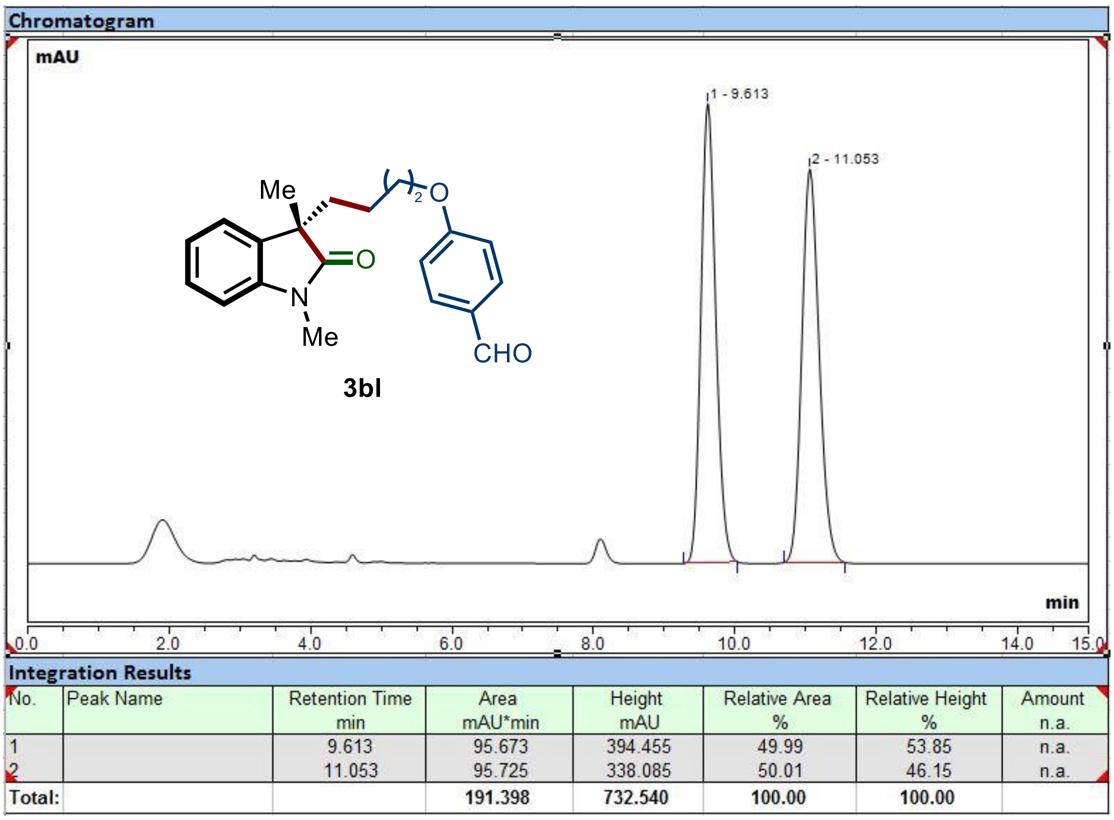

Integration Results

No.	Peak Name	Retention Time min	Area mAU*min	Height mAU	Relative Area %	Relative Height %	Amount n.a.
1		9.613	95.673	394.455	49.99	53.85	n.a.
2		11.053	95.725	338.085	50.01	46.15	n.a.
Total:			**191.398**	**732.540**	**100.00**	**100.00**	

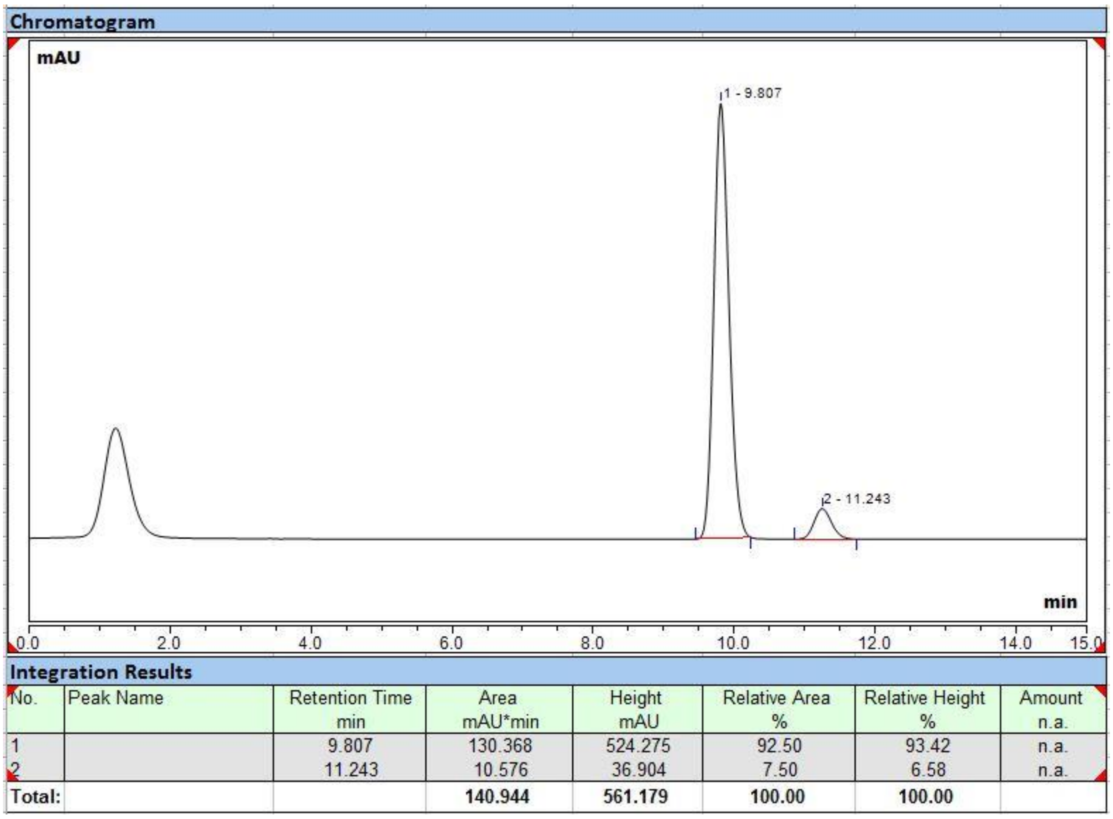

Integration Results

No.	Peak Name	Retention Time min	Area mAU*min	Height mAU	Relative Area %	Relative Height %	Amount n.a.
1		9.807	130.368	524.275	92.50	93.42	n.a.
2		11.243	10.576	36.904	7.50	6.58	n.a.
Total:			**140.944**	**561.179**	**100.00**	**100.00**	

Supplementary Figure 27.

Chromatogram

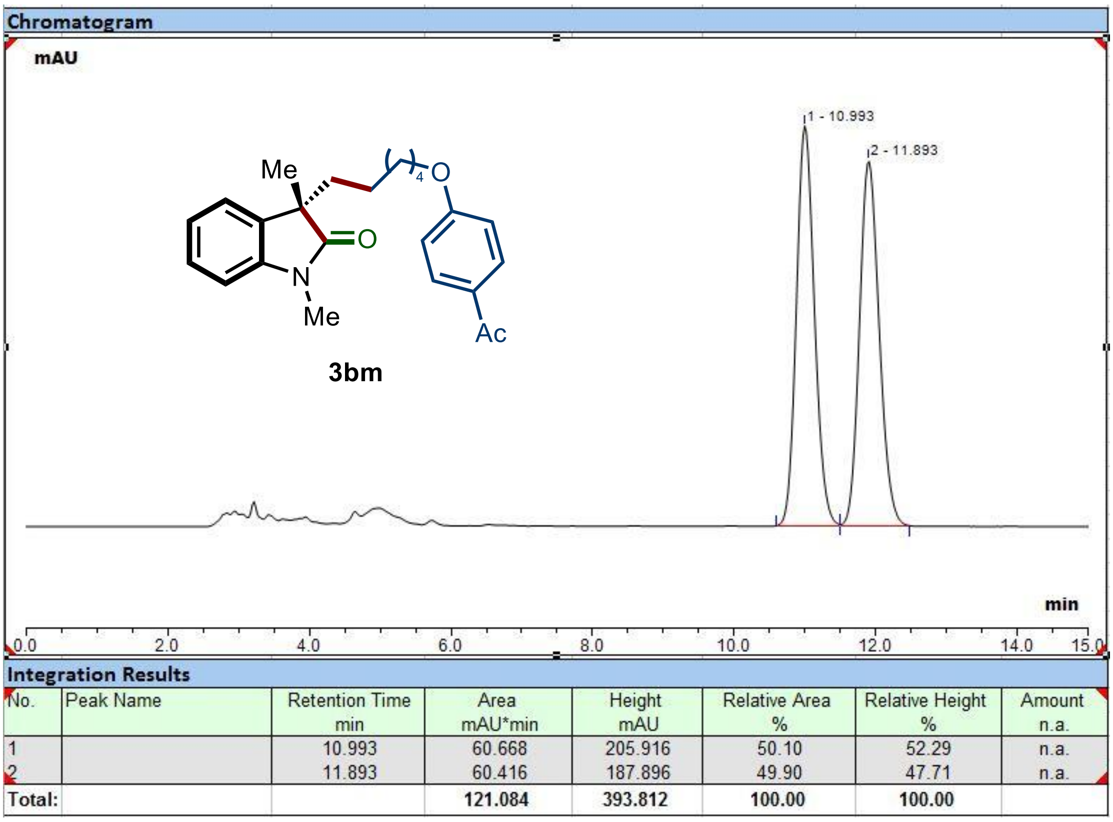

Integration Results

No.	Peak Name	Retention Time min	Area mAU*min	Height mAU	Relative Area %	Relative Height %	Amount n.a.
1		10.993	60.668	205.916	50.10	52.29	n.a.
2		11.893	60.416	187.896	49.90	47.71	n.a.
Total:			121.084	393.812	100.00	100.00	

Chromatogram

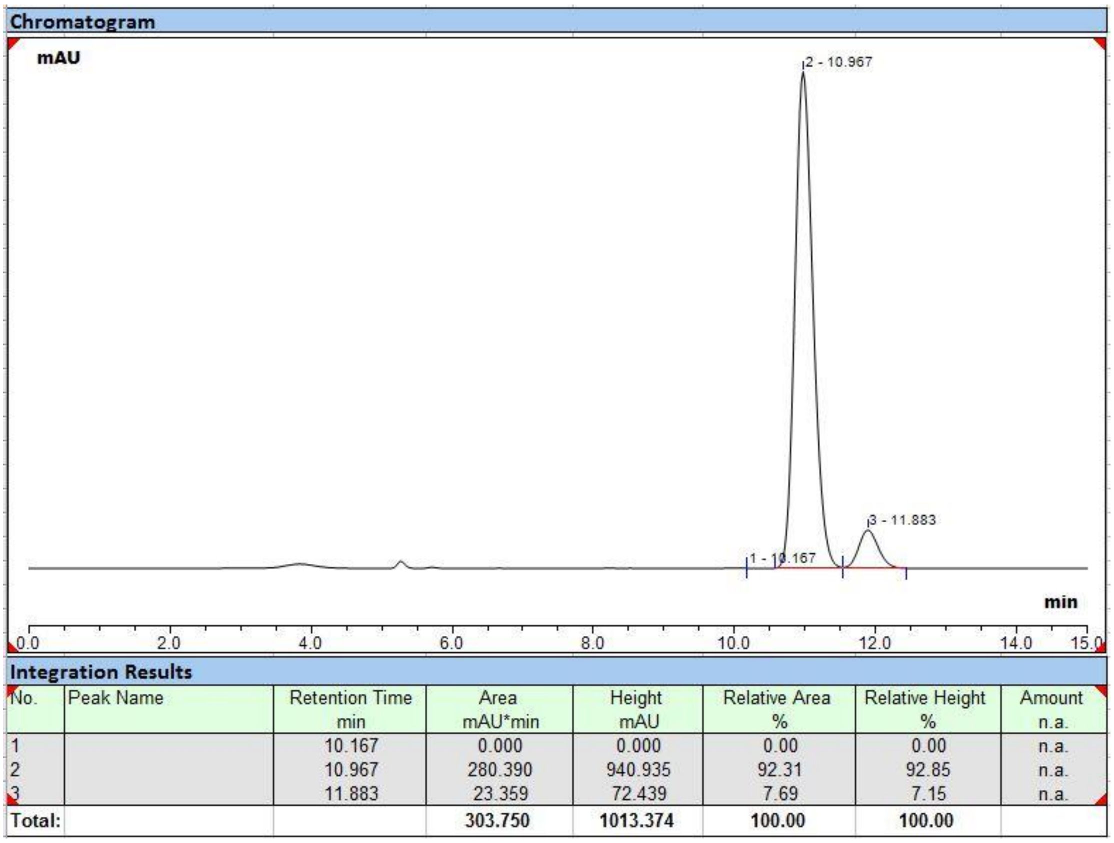

Integration Results

No.	Peak Name	Retention Time min	Area mAU*min	Height mAU	Relative Area %	Relative Height %	Amount n.a.
1		10.167	0.000	0.000	0.00	0.00	n.a.
2		10.967	280.390	940.935	92.31	92.85	n.a.
3		11.883	23.359	72.439	7.69	7.15	n.a.
Total:			303.750	1013.374	100.00	100.00	

Supplementary Figure 28.

Chromatogram

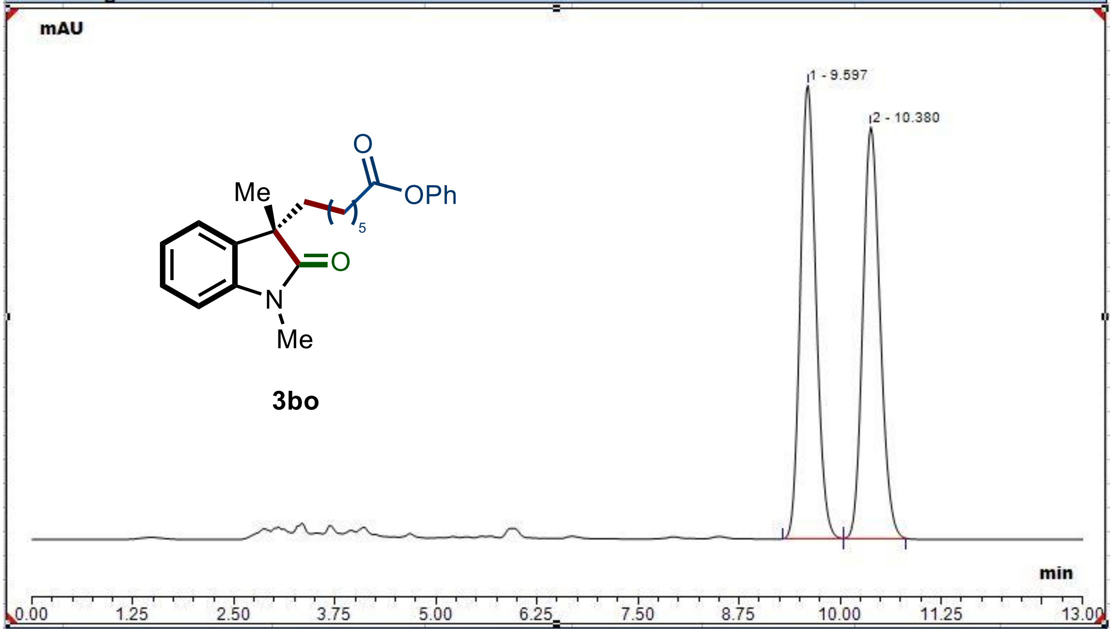

Integration Results

No.	Peak Name	Retention Time<br>min	Area<br>mAU*min	Height<br>mAU	Relative Area<br>%	Relative Height<br>%	Amount<br>n.a.
1		9.597	38.602	171.779	50.10	52.42	n.a.
2		10.380	38.450	155.939	49.90	47.58	n.a.
**Total:**			**77.051**	**327.718**	**100.00**	**100.00**	

Chromatogram

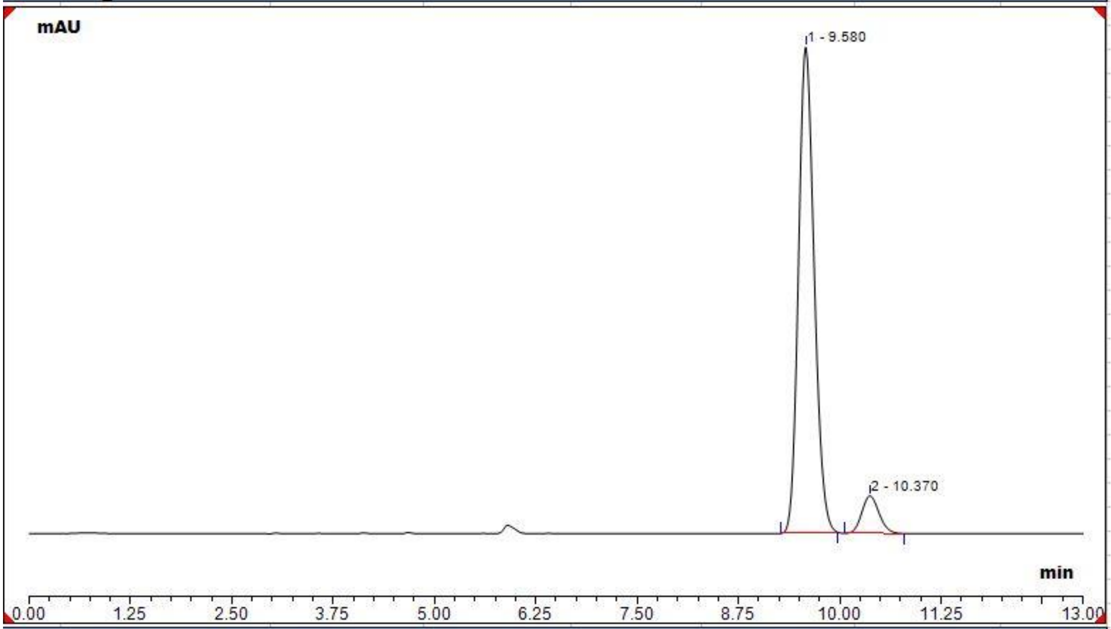

Integration Results

No.	Peak Name	Retention Time<br>min	Area<br>mAU*min	Height<br>mAU	Relative Area<br>%	Relative Height<br>%	Amount<br>n.a.
1		9.580	93.703	417.319	92.19	92.81	n.a.
2		10.370	7.943	32.332	7.81	7.19	n.a.
**Total:**			**101.646**	**449.651**	**100.00**	**100.00**	

Supplementary Figure 29.

Chromatogram

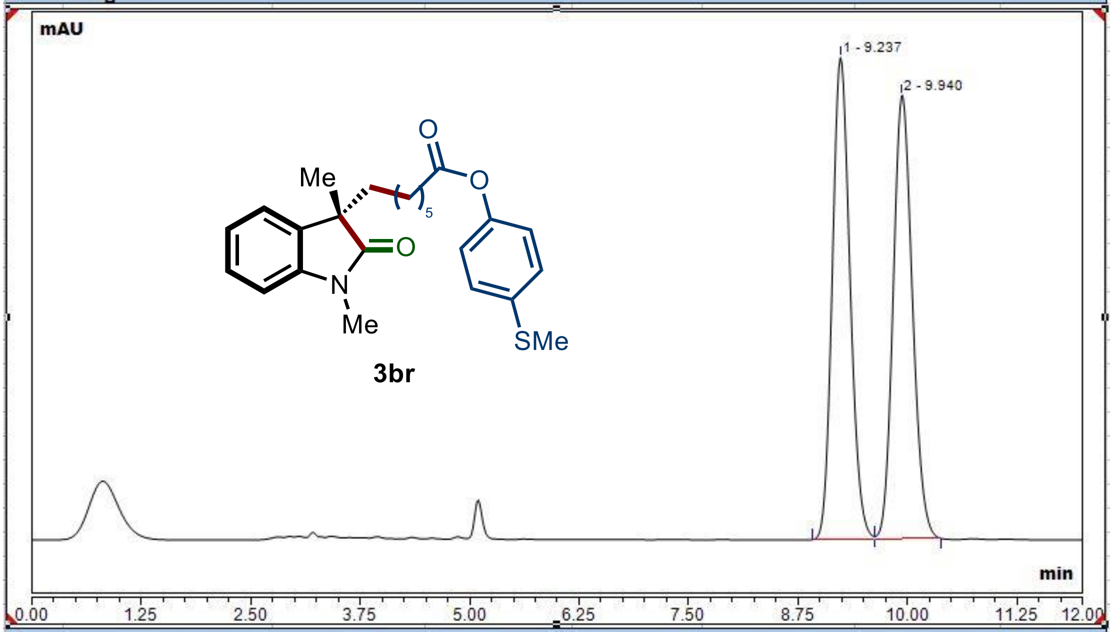

Integration Results

No.	Peak Name	Retention Time min	Area mAU*min	Height mAU	Relative Area %	Relative Height %	Amount n.a.
1		9.237	107.035	456.395	50.07	52.08	n.a.
2		9.940	106.716	419.917	49.93	47.92	n.a.
Total:			**213.751**	**876.312**	**100.00**	**100.00**	

Chromatogram

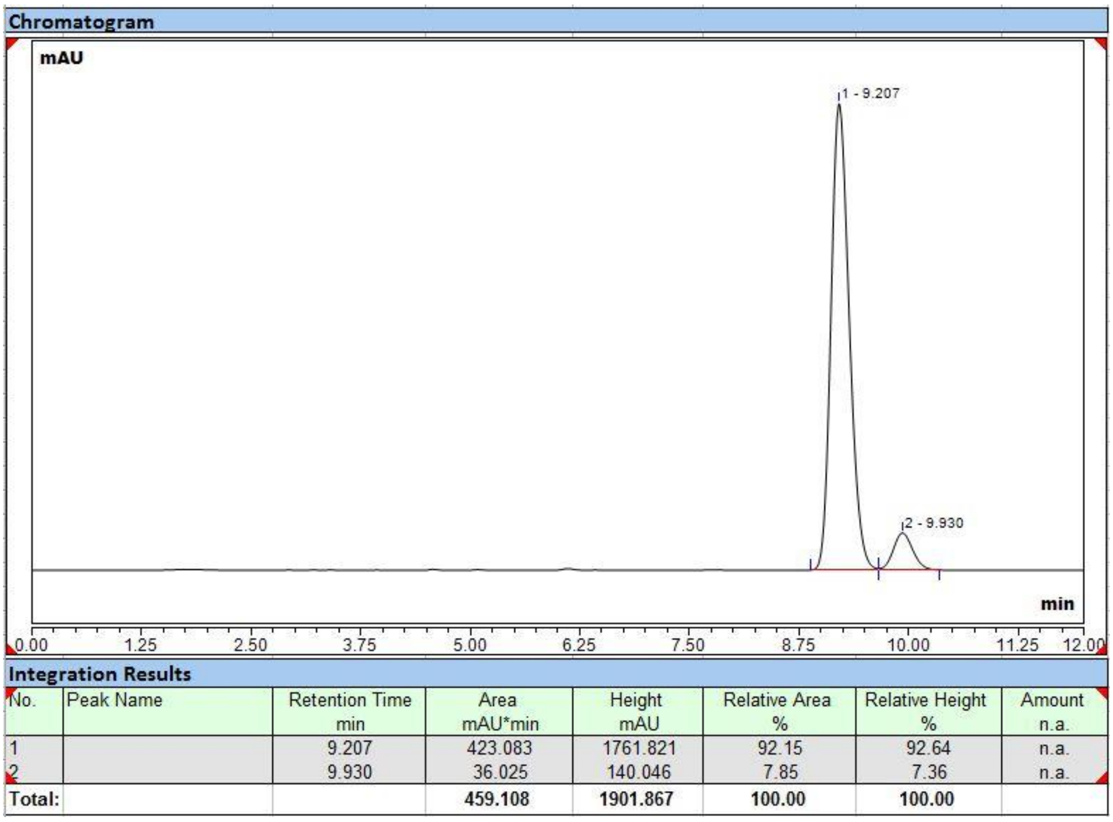

Integration Results

No.	Peak Name	Retention Time min	Area mAU*min	Height mAU	Relative Area %	Relative Height %	Amount n.a.
1		9.207	423.083	1761.821	92.15	92.64	n.a.
2		9.930	36.025	140.046	7.85	7.36	n.a.
Total:			**459.108**	**1901.867**	**100.00**	**100.00**	

Supplementary Figure 30.

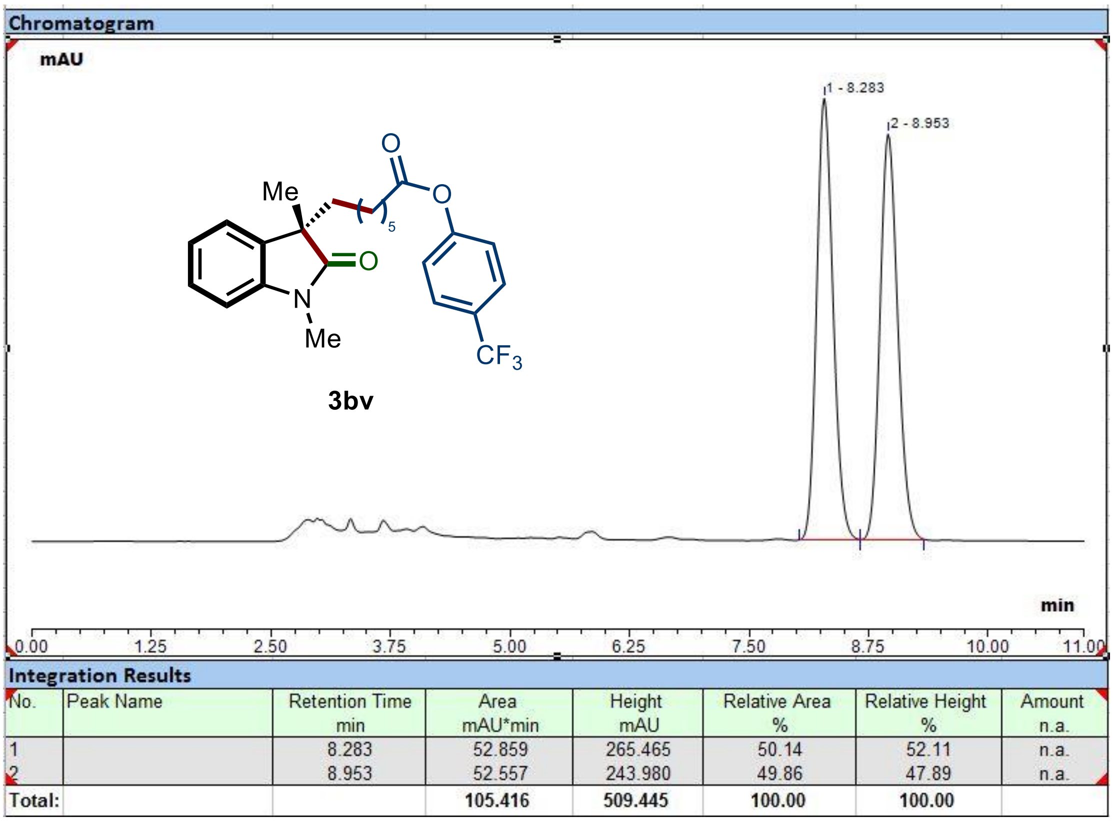

Integration Results

No.	Peak Name	Retention Time min	Area mAU*min	Height mAU	Relative Area %	Relative Height %	Amount n.a.
1		8.283	52.859	265.465	50.14	52.11	n.a.
2		8.953	52.557	243.980	49.86	47.89	n.a.
Total:			105.416	509.445	100.00	100.00	

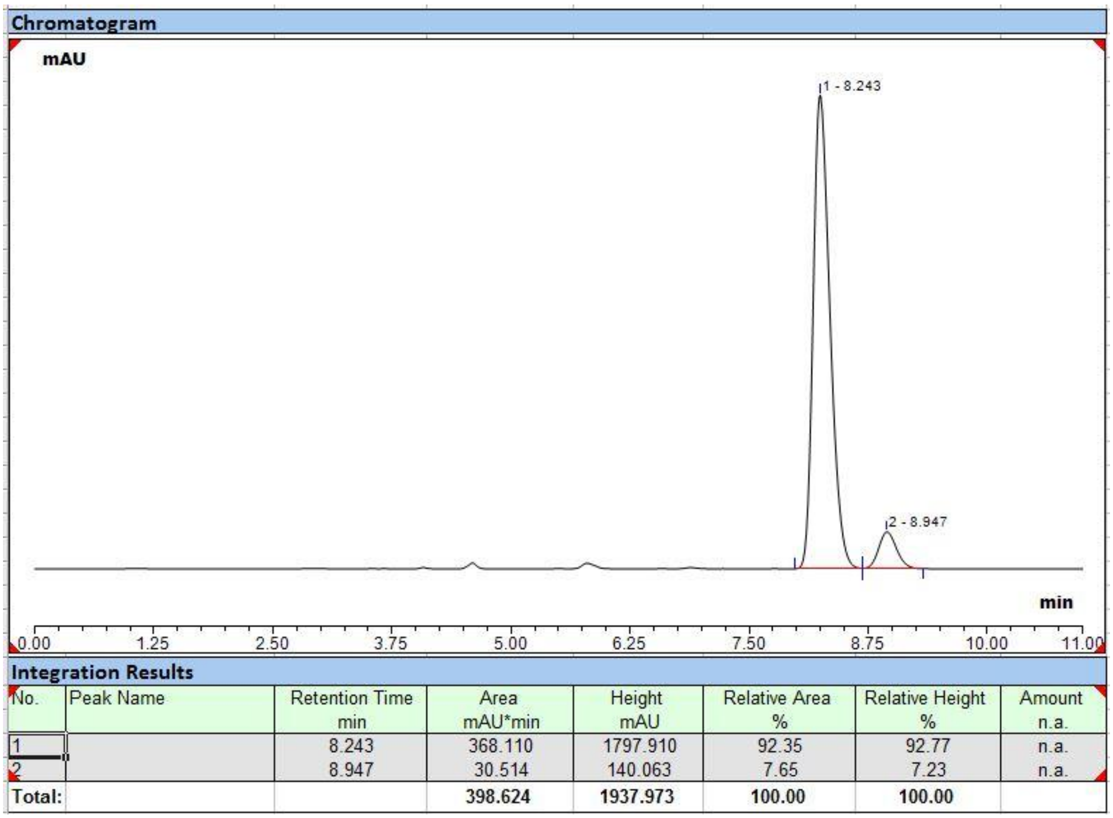

Integration Results

No.	Peak Name	Retention Time min	Area mAU*min	Height mAU	Relative Area %	Relative Height %	Amount n.a.
1		8.243	368.110	1797.910	92.35	92.77	n.a.
2		8.947	30.514	140.063	7.65	7.23	n.a.
Total:			398.624	1937.973	100.00	100.00	

Supplementary Figure 31.

Chromatogram

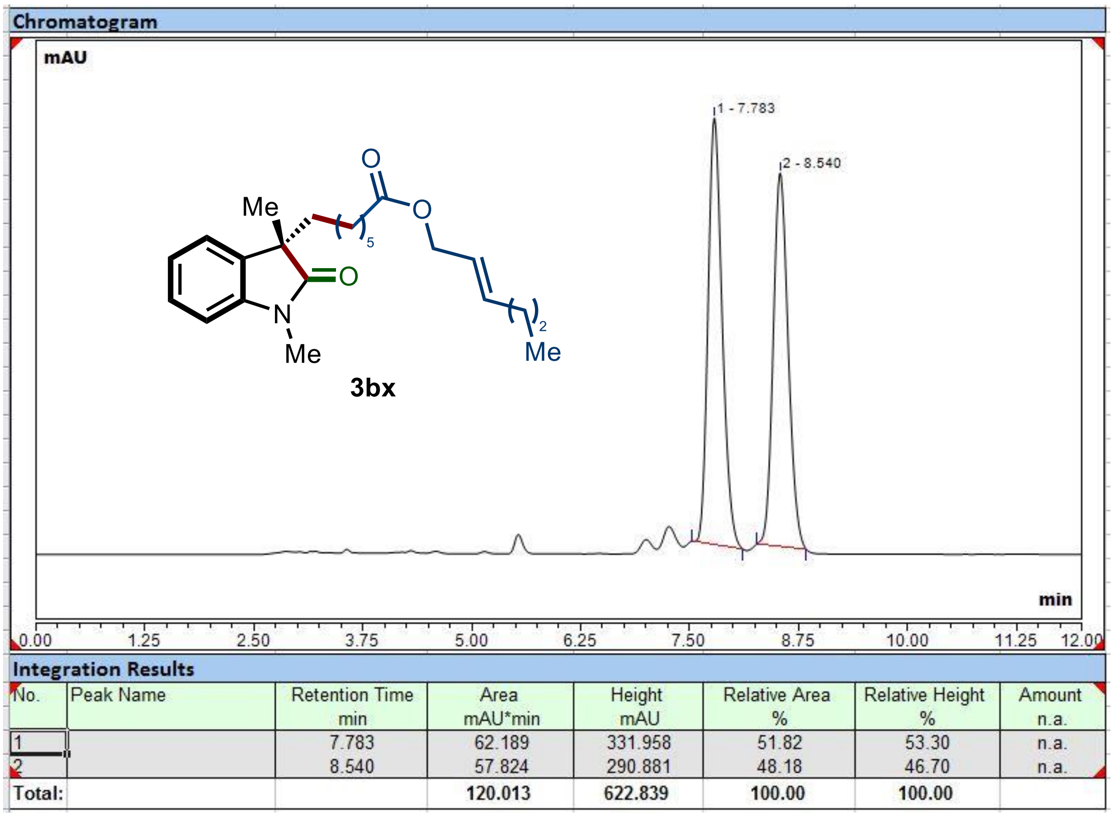

Integration Results

No.	Peak Name	Retention Time<br>min	Area<br>mAU*min	Height<br>mAU	Relative Area<br>%	Relative Height<br>%	Amount<br>n.a.
1		7.783	62.189	331.958	51.82	53.30	n.a.
2		8.540	57.824	290.881	48.18	46.70	n.a.
**Total:**			**120.013**	**622.839**	**100.00**	**100.00**	

Chromatogram

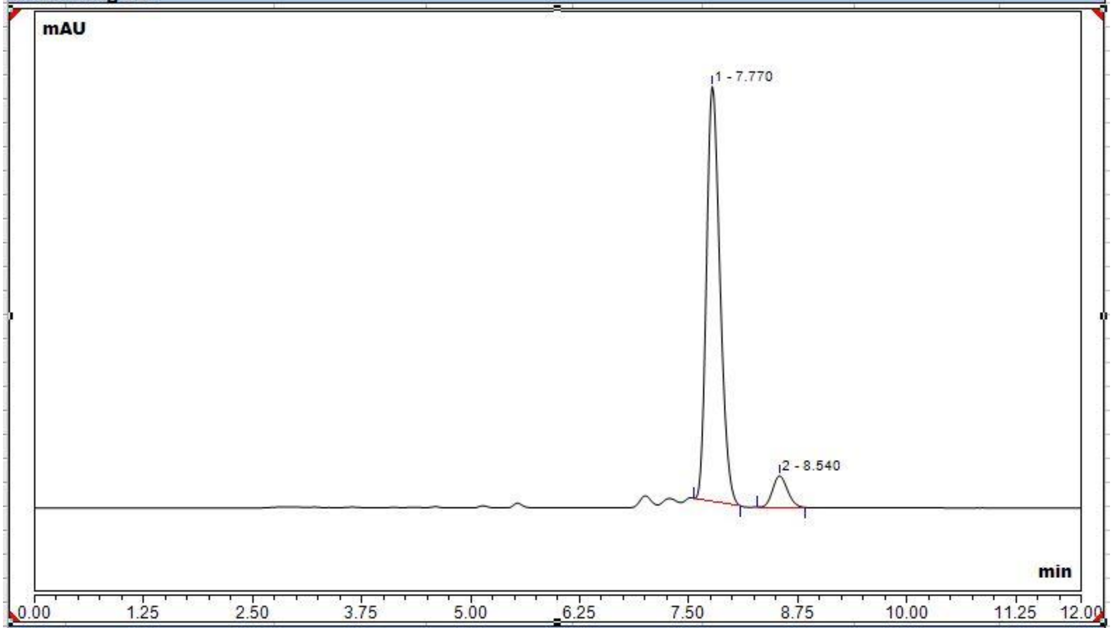

Integration Results

No.	Peak Name	Retention Time<br>min	Area<br>mAU*min	Height<br>mAU	Relative Area<br>%	Relative Height<br>%	Amount<br>n.a.
1		7.770	179.972	1003.380	92.28	92.95	n.a.
2		8.540	15.050	76.100	7.72	7.05	n.a.
**Total:**			**195.022**	**1079.480**	**100.00**	**100.00**	

Supplementary Figure 32.

Chromatogram

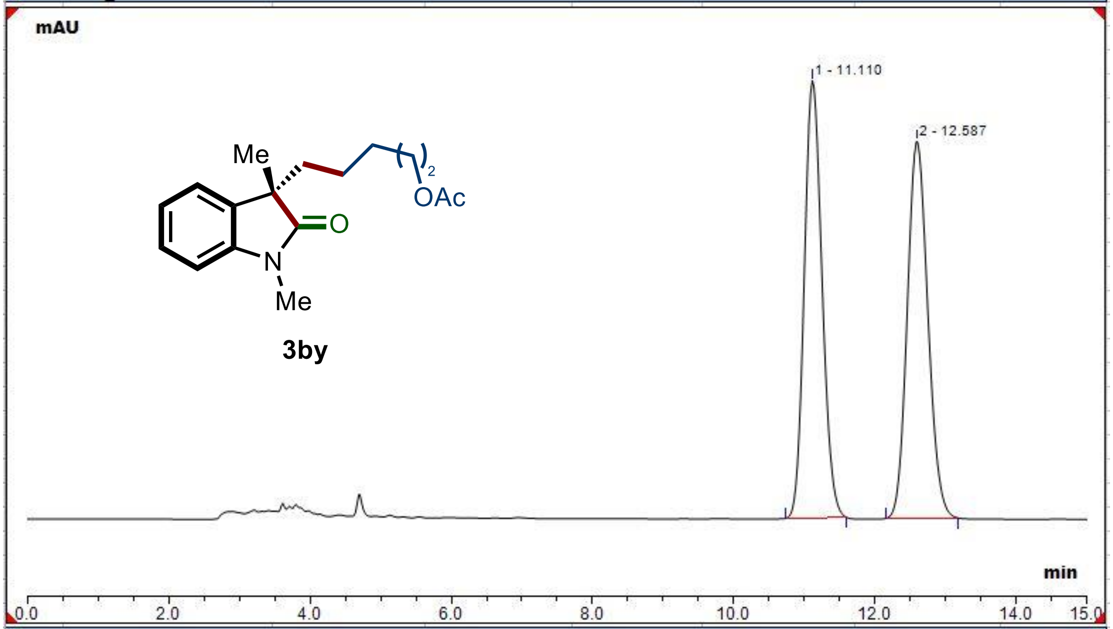

Integration Results

No.	Peak Name	Retention Time min	Area mAU*min	Height mAU	Relative Area %	Relative Height %	Amount n.a.
1		11.110	87.429	301.211	49.95	53.73	n.a.
2		12.587	87.592	259.367	50.05	46.27	n.a.
Total:			**175.022**	**560.578**	**100.00**	**100.00**	

Chromatogram

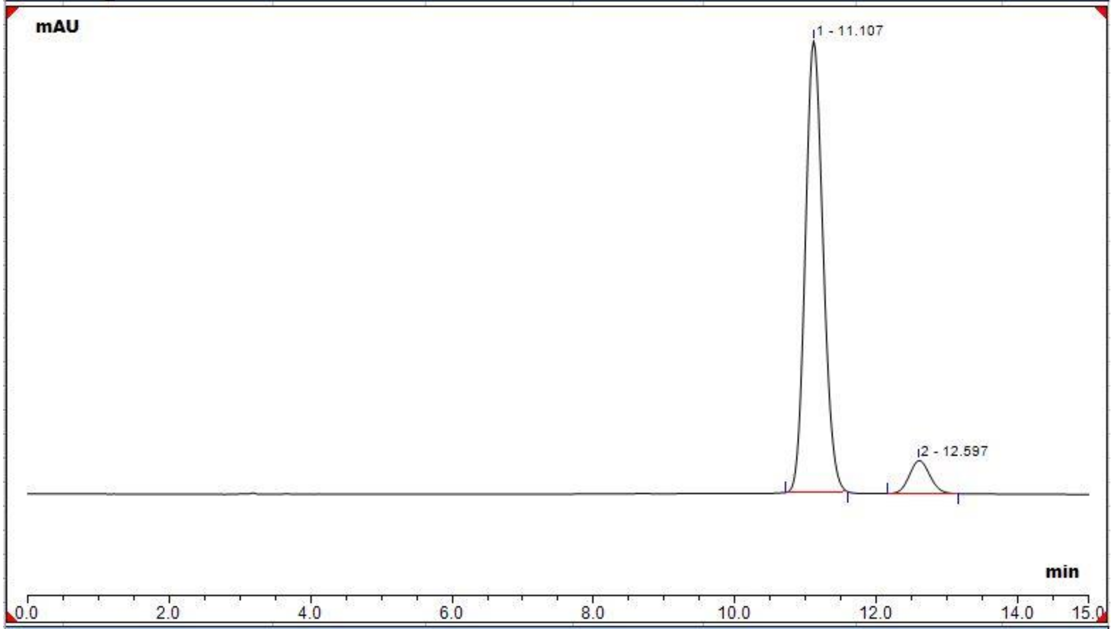

Integration Results

No.	Peak Name	Retention Time min	Area mAU*min	Height mAU	Relative Area %	Relative Height %	Amount n.a.
1		11.107	67.522	232.921	92.18	93.17	n.a.
2		12.597	5.730	17.067	7.82	6.83	n.a.
Total:			**73.252**	**249.988**	**100.00**	**100.00**	

Supplementary Figure 33.

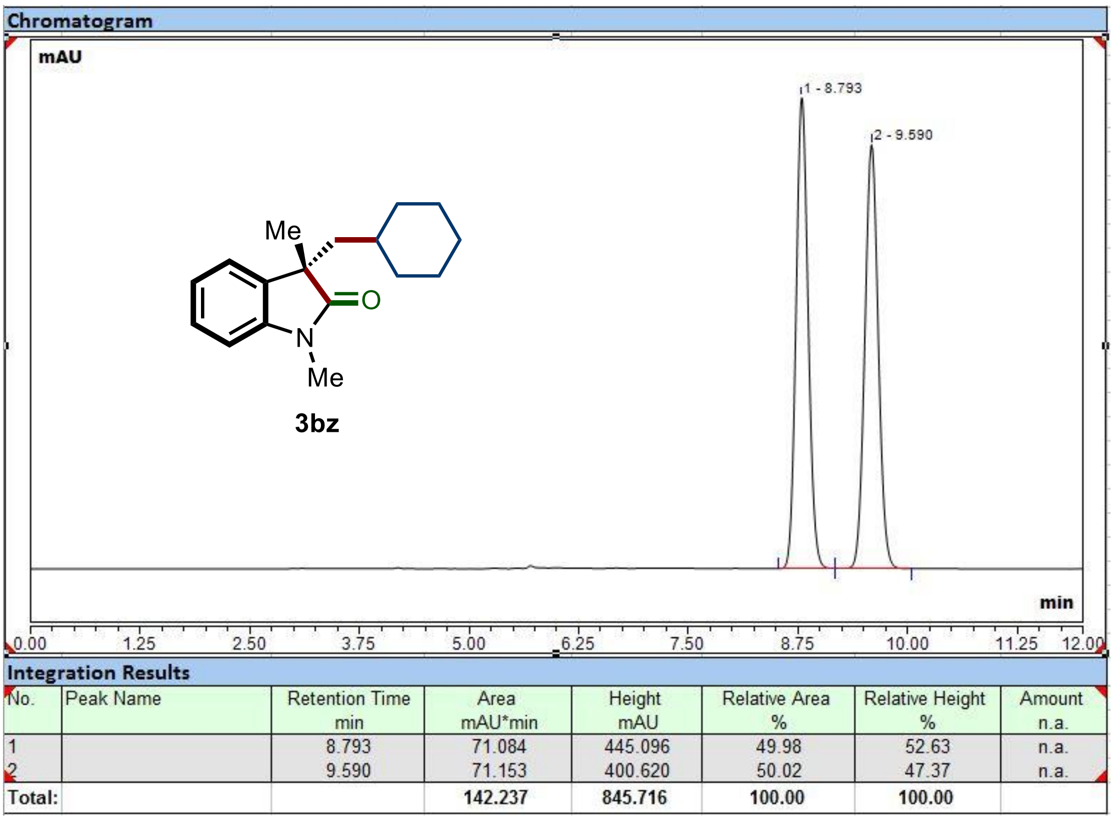

Integration Results

No.	Peak Name	Retention Time min	Area mAU*min	Height mAU	Relative Area %	Relative Height %	Amount n.a.
1		8.793	71.084	445.096	49.98	52.63	n.a.
2		9.590	71.153	400.620	50.02	47.37	n.a.
Total:			**142.237**	**845.716**	**100.00**	**100.00**	

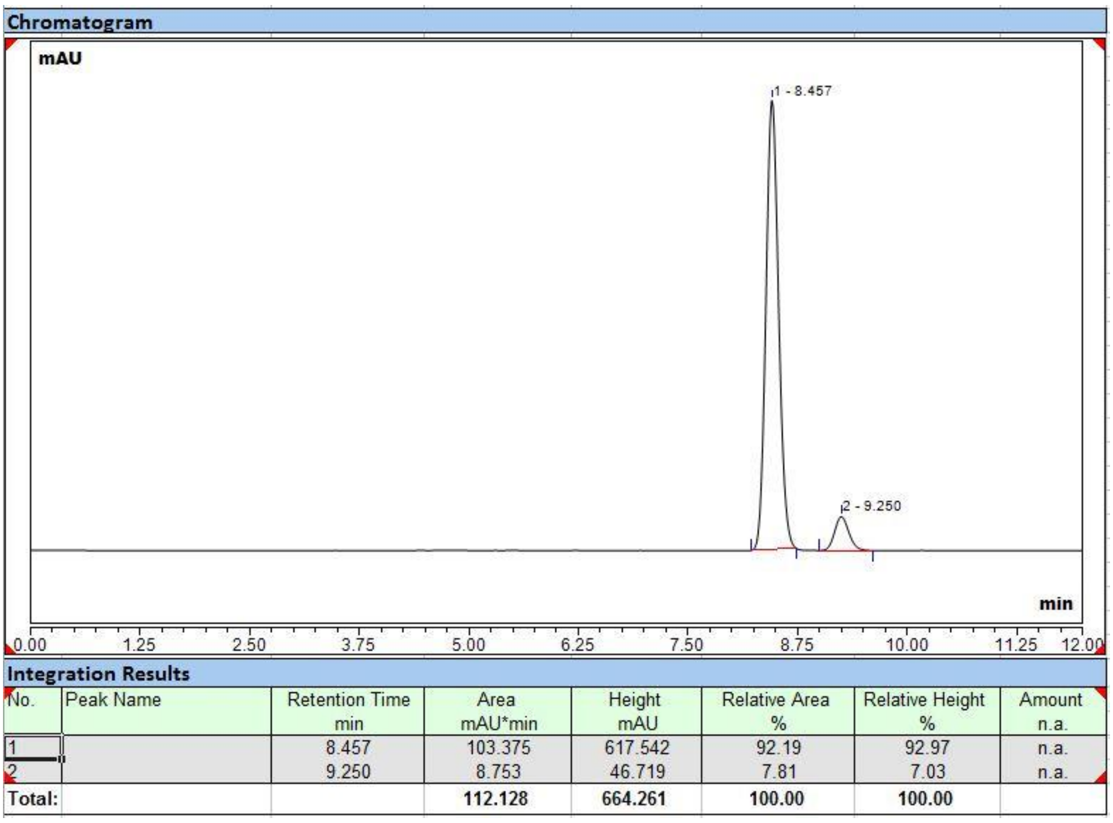

Integration Results

No.	Peak Name	Retention Time min	Area mAU*min	Height mAU	Relative Area %	Relative Height %	Amount n.a.
1		8.457	103.375	617.542	92.19	92.97	n.a.
2		9.250	8.753	46.719	7.81	7.03	n.a.
Total:			**112.128**	**664.261**	**100.00**	**100.00**	

Supplementary Figure 34.

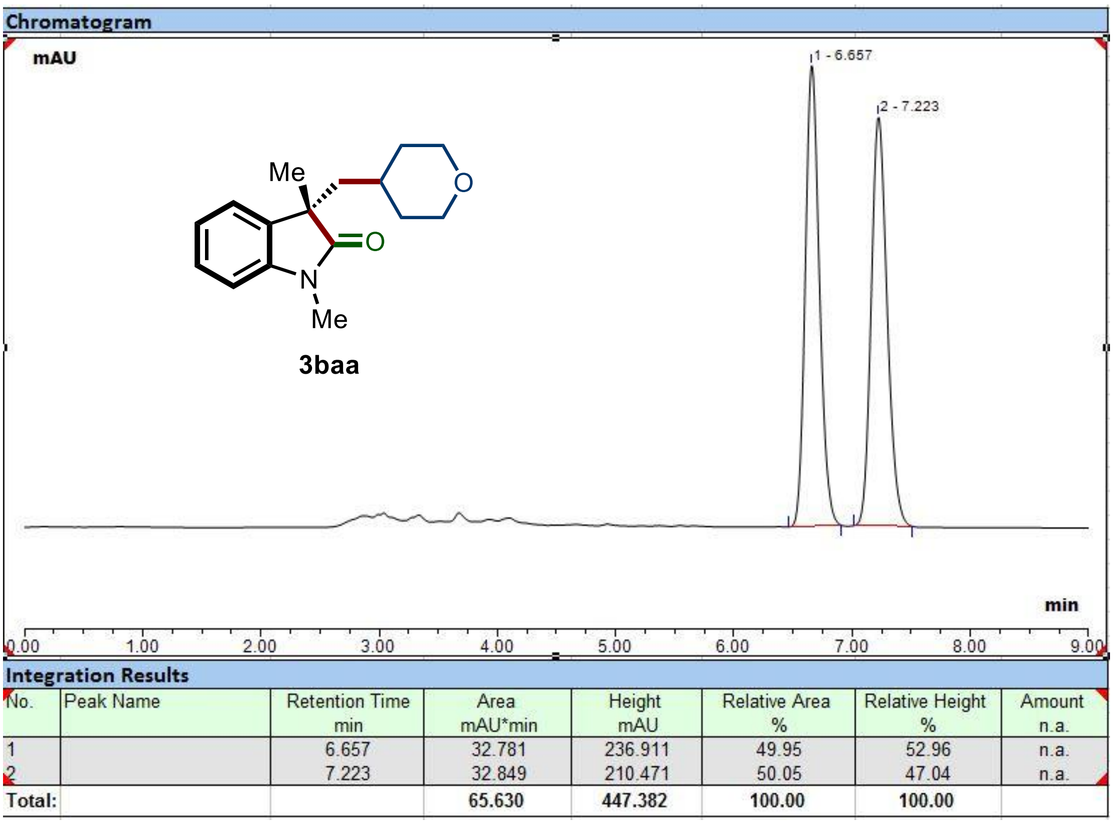

Integration Results

No.	Peak Name	Retention Time min	Area mAU*min	Height mAU	Relative Area %	Relative Height %	Amount n.a.
1		6.657	32.781	236.911	49.95	52.96	n.a.
2		7.223	32.849	210.471	50.05	47.04	n.a.
Total:			**65.630**	**447.382**	**100.00**	**100.00**	

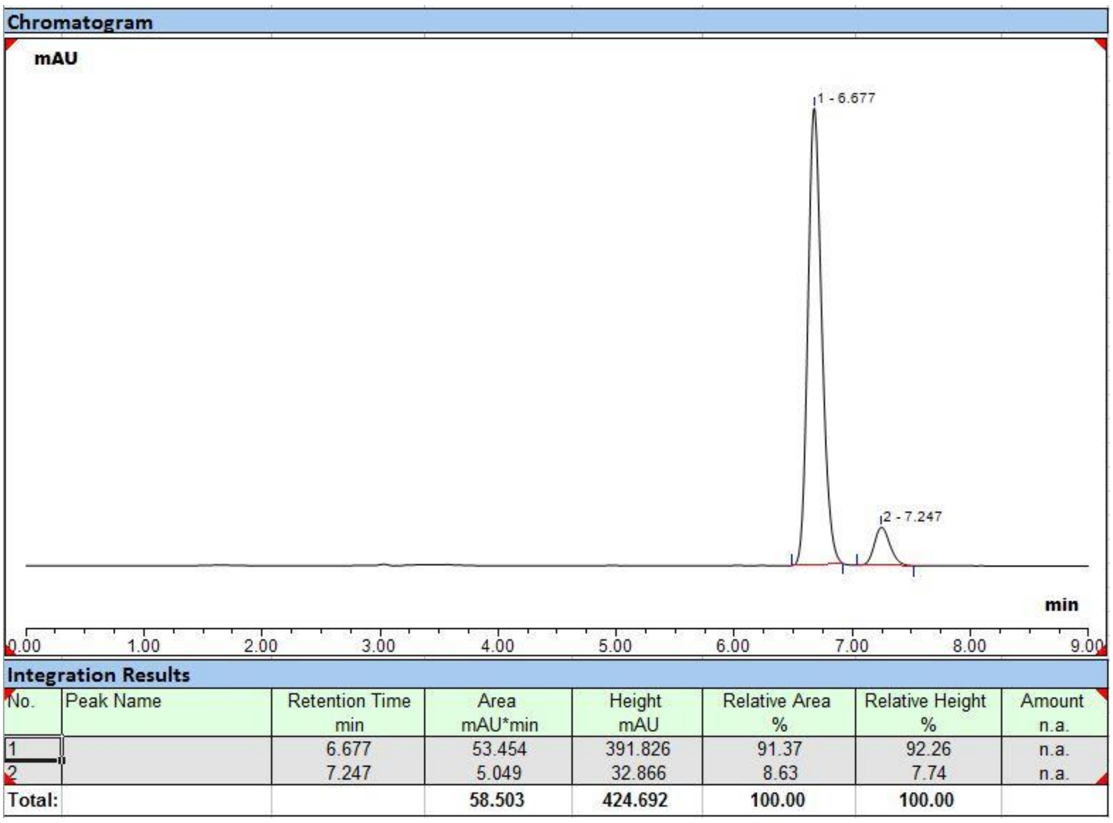

Integration Results

No.	Peak Name	Retention Time min	Area mAU*min	Height mAU	Relative Area %	Relative Height %	Amount n.a.
1		6.677	53.454	391.826	91.37	92.26	n.a.
2		7.247	5.049	32.866	8.63	7.74	n.a.
Total:			**58.503**	**424.692**	**100.00**	**100.00**	

Supplementary Figure 35.

Chromatogram

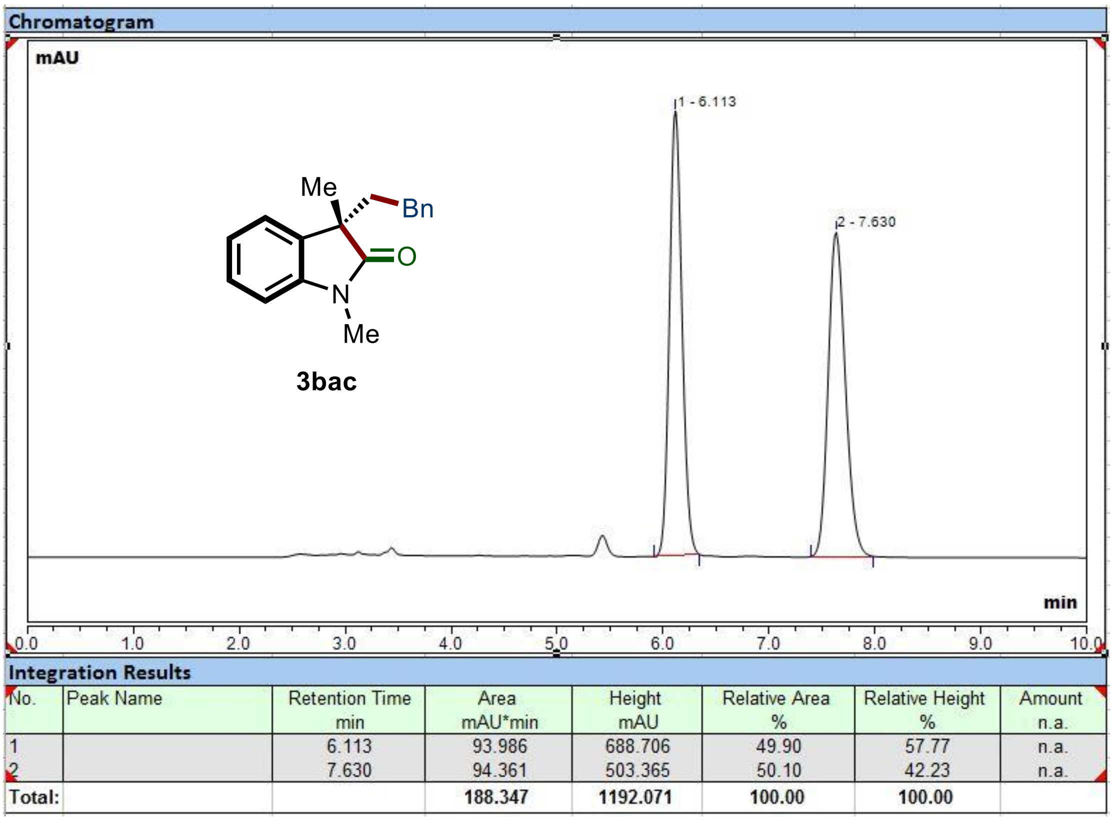

Integration Results

No.	Peak Name	Retention Time min	Area mAU*min	Height mAU	Relative Area %	Relative Height %	Amount n.a.
1		6.113	93.986	688.706	49.90	57.77	n.a.
2		7.630	94.361	503.365	50.10	42.23	n.a.
Total:			**188.347**	**1192.071**	**100.00**	**100.00**	

Chromatogram

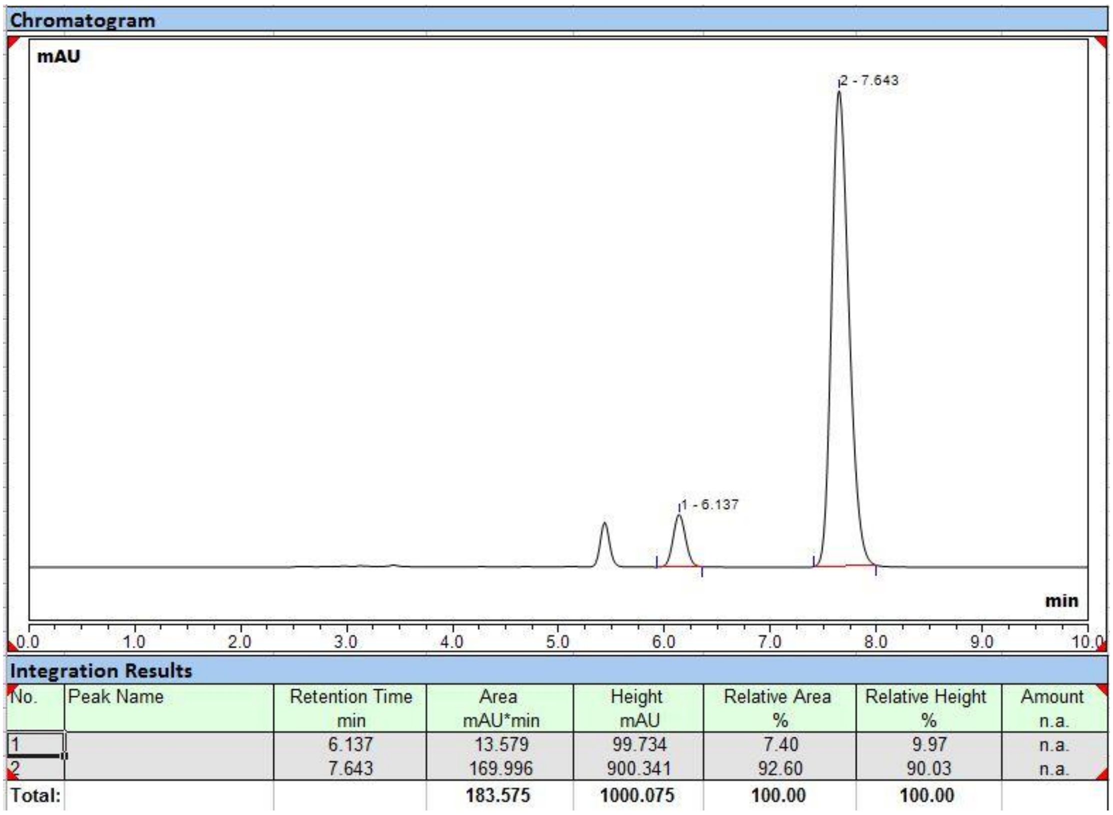

Integration Results

No.	Peak Name	Retention Time min	Area mAU*min	Height mAU	Relative Area %	Relative Height %	Amount n.a.
1		6.137	13.579	99.734	7.40	9.97	n.a.
2		7.643	169.996	900.341	92.60	90.03	n.a.
Total:			**183.575**	**1000.075**	**100.00**	**100.00**	

Supplementary Figure 36.

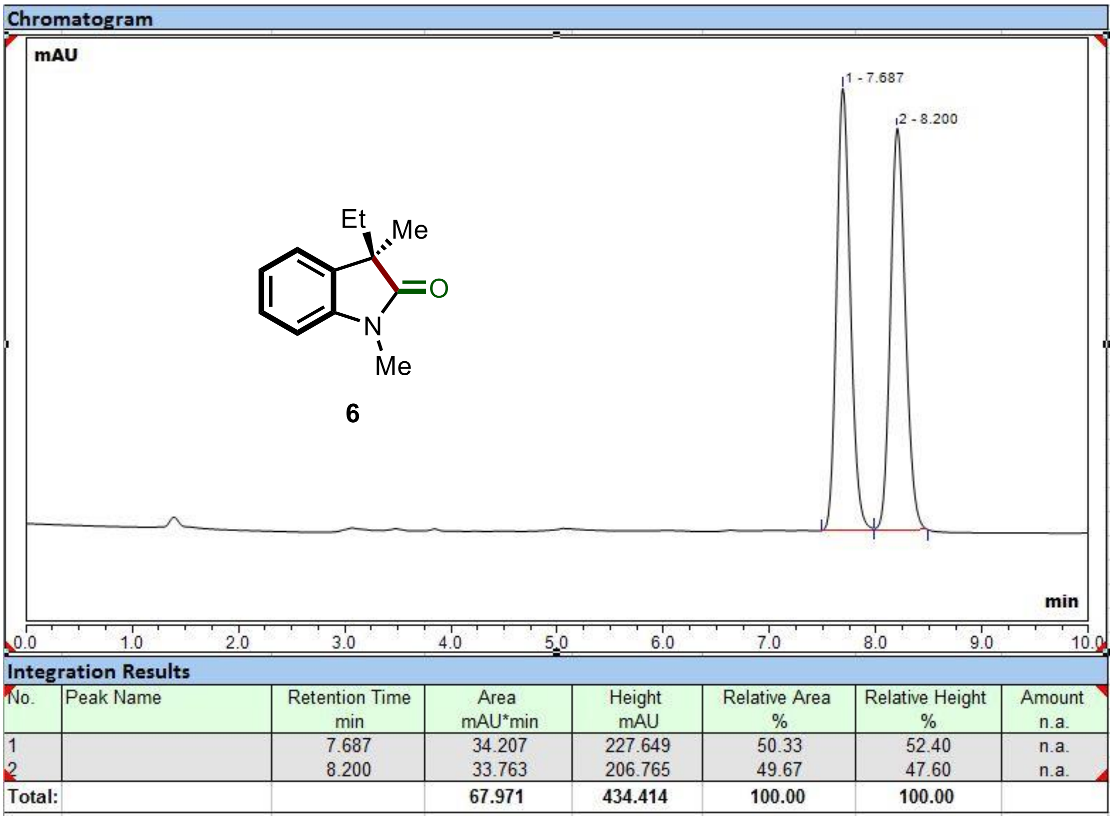

Integration Results

No.	Peak Name	Retention Time<br>min	Area<br>mAU*min	Height<br>mAU	Relative Area<br>%	Relative Height<br>%	Amount<br>n.a.
1		7.687	34.207	227.649	50.33	52.40	n.a.
2		8.200	33.763	206.765	49.67	47.60	n.a.
Total:			**67.971**	**434.414**	**100.00**	**100.00**	

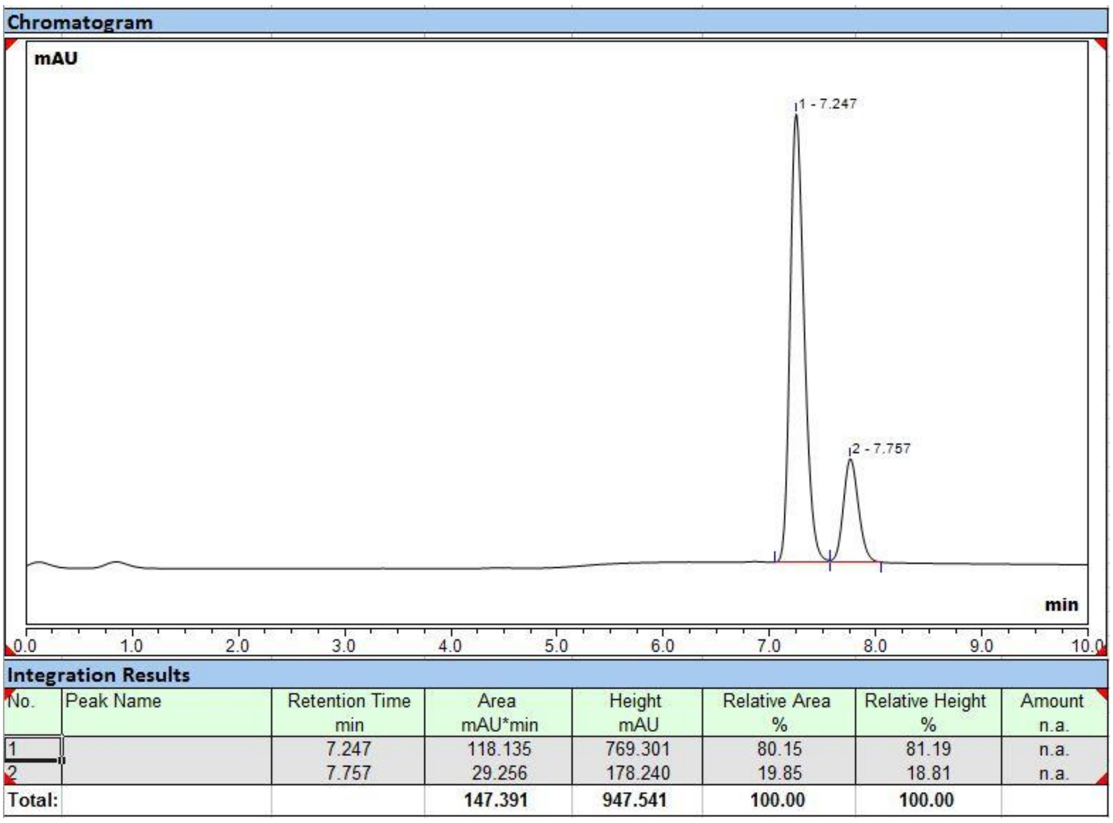

Integration Results

No.	Peak Name	Retention Time<br>min	Area<br>mAU*min	Height<br>mAU	Relative Area<br>%	Relative Height<br>%	Amount<br>n.a.
1		7.247	118.135	769.301	80.15	81.19	n.a.
2		7.757	29.256	178.240	19.85	18.81	n.a.
Total:			**147.391**	**947.541**	**100.00**	**100.00**	

Supplementary Figure 37

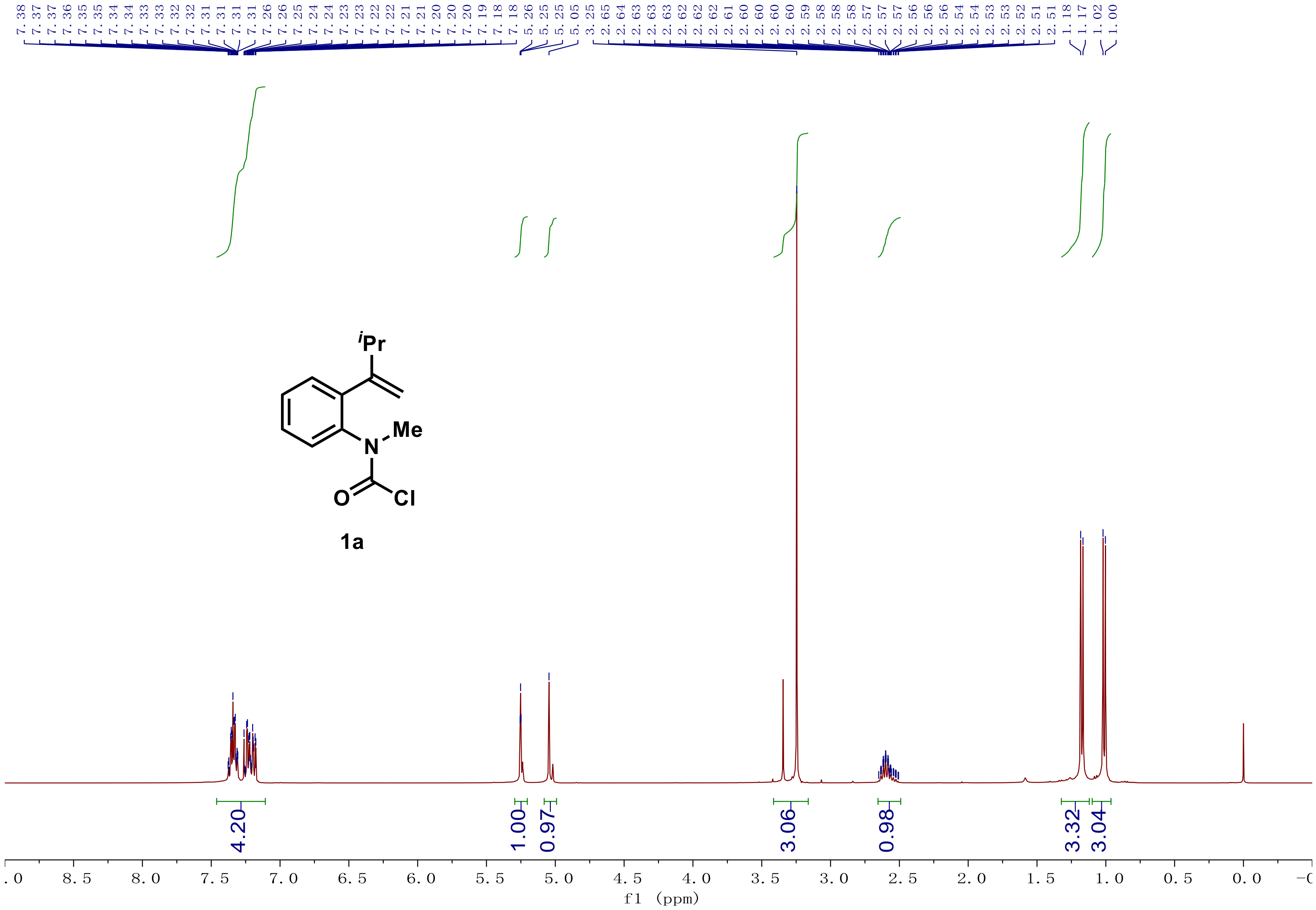

Supplementary Figure 38

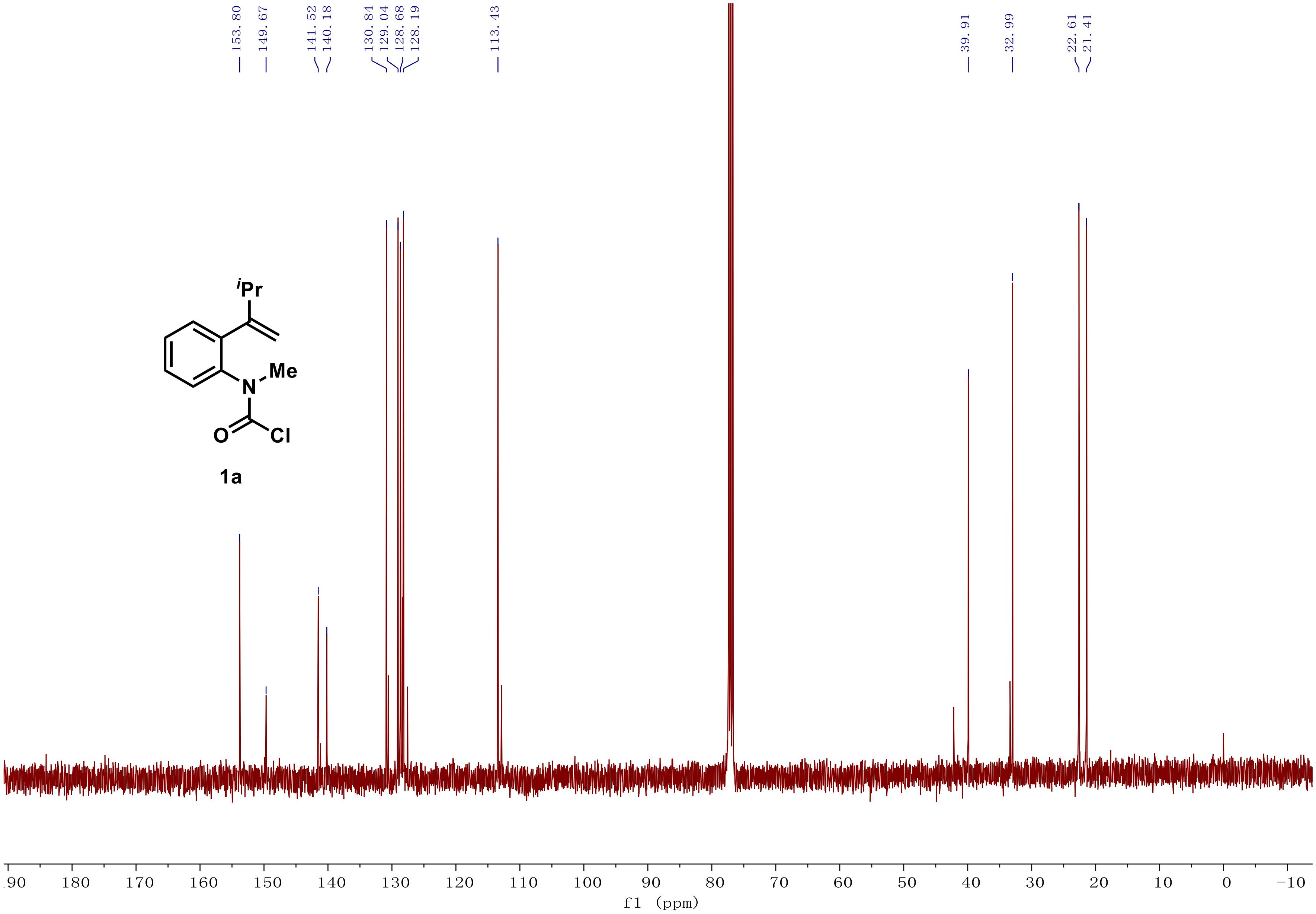

1a

Supplementary Figure 39

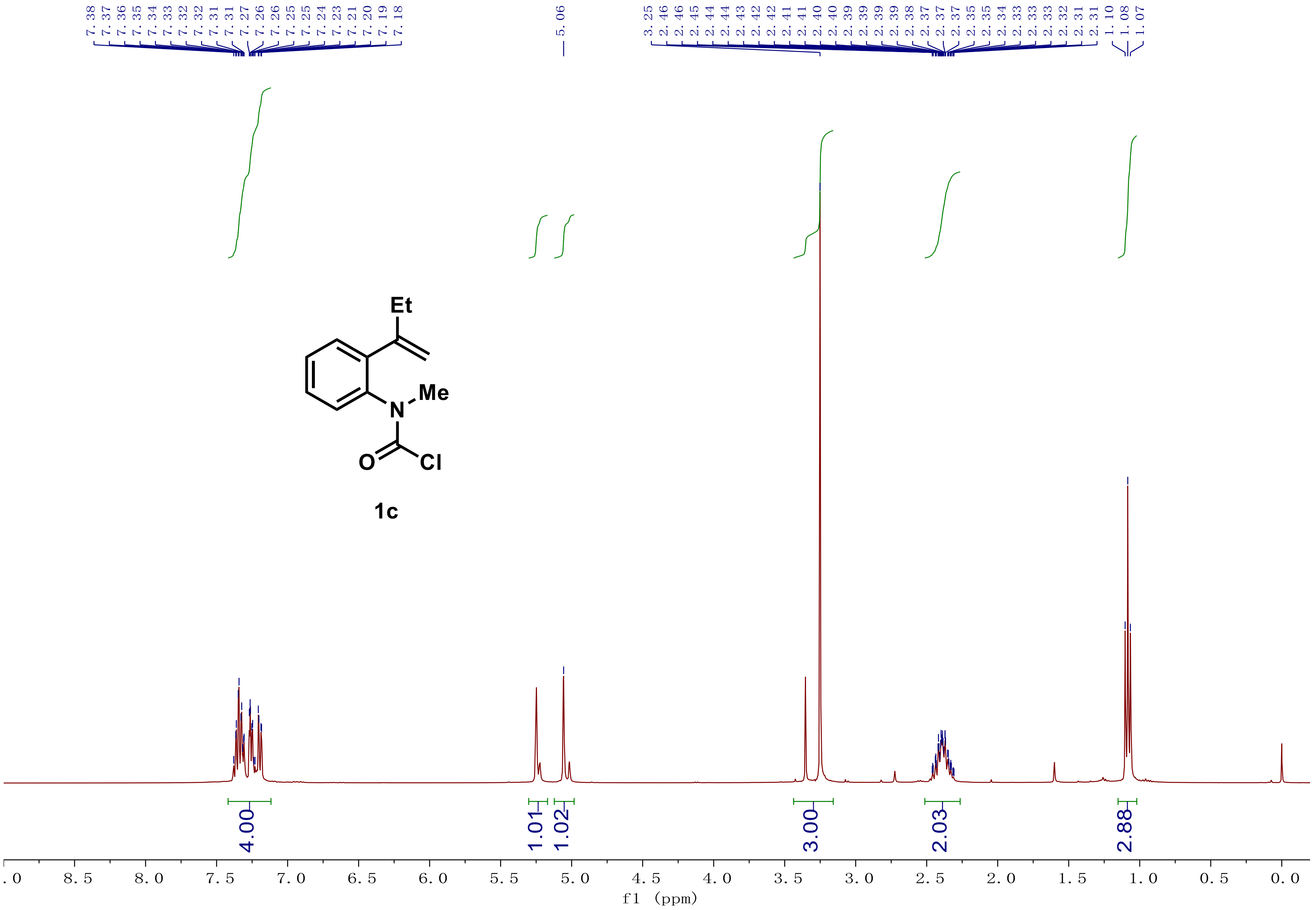

Supplementary Figure 40

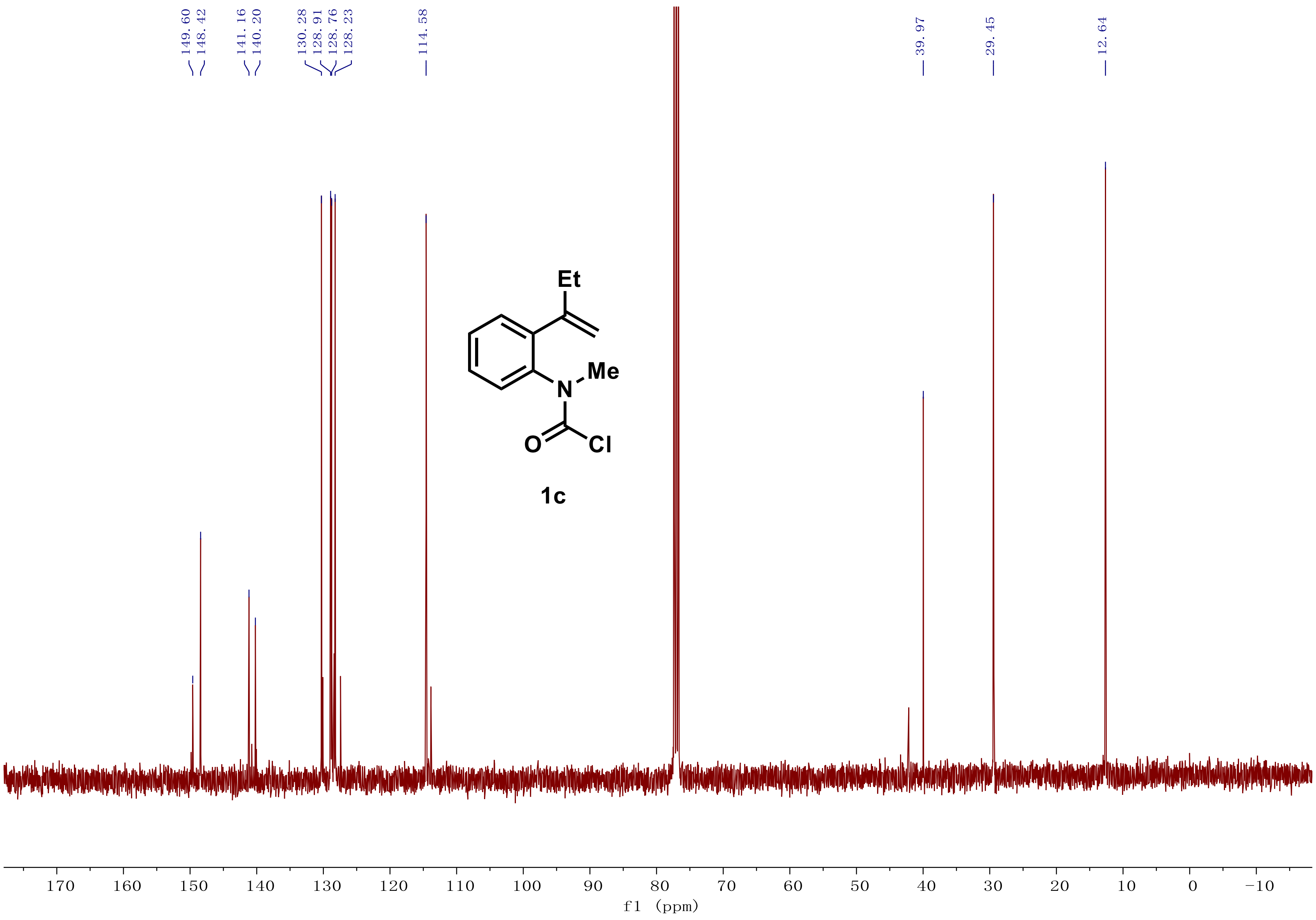

Supplementary Figure 41

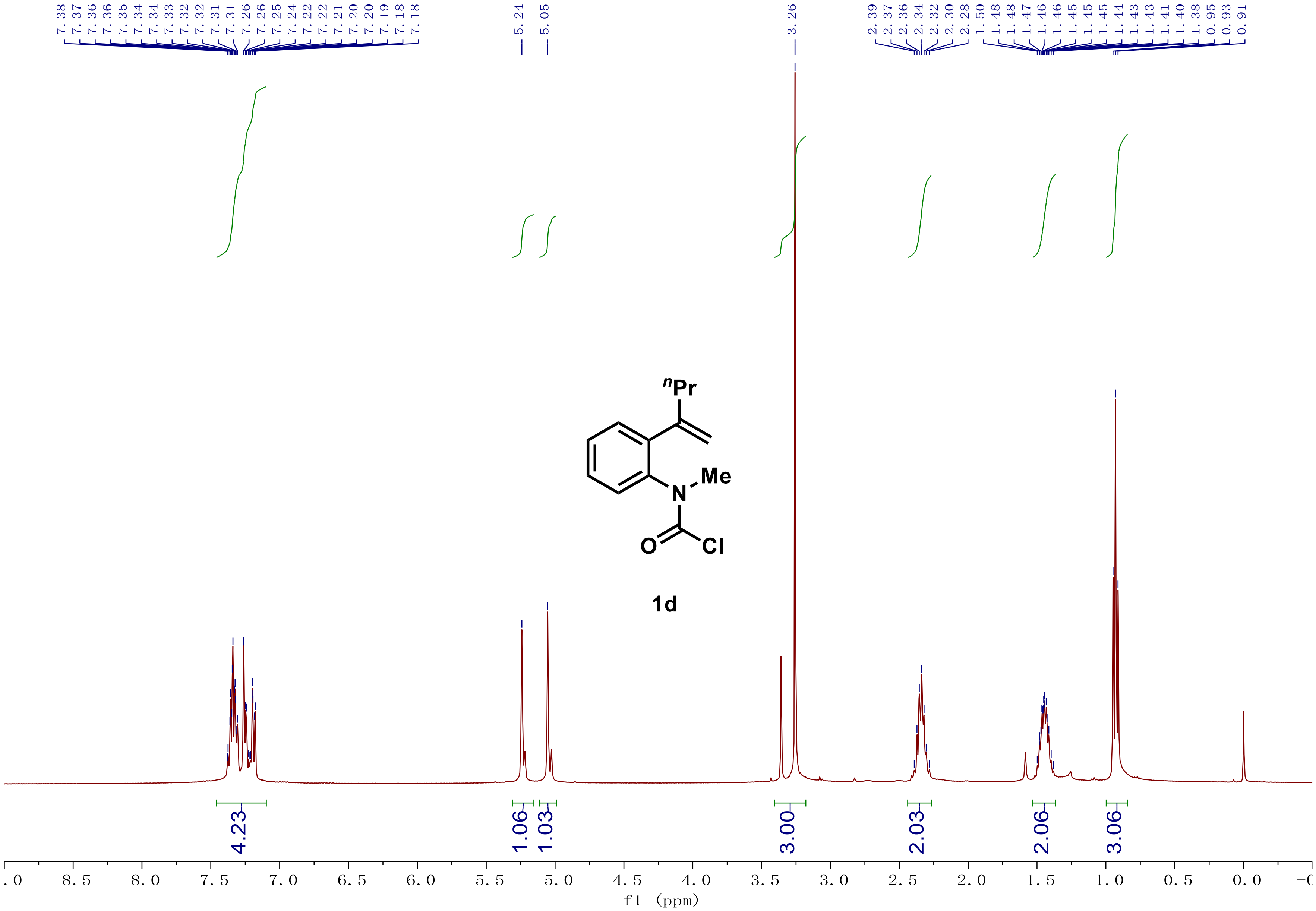

Supplementary Figure 42

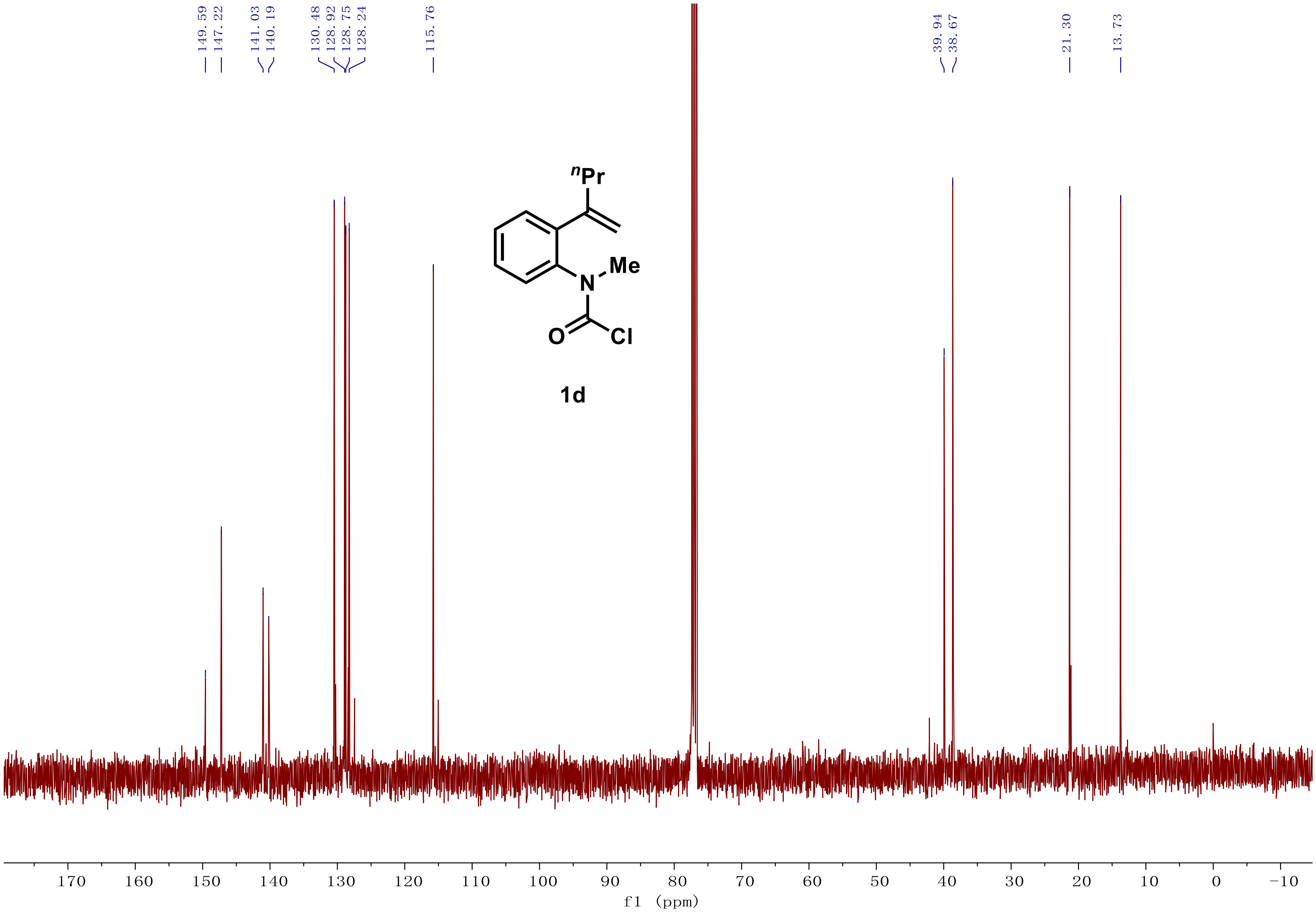

Supplementary Figure 43

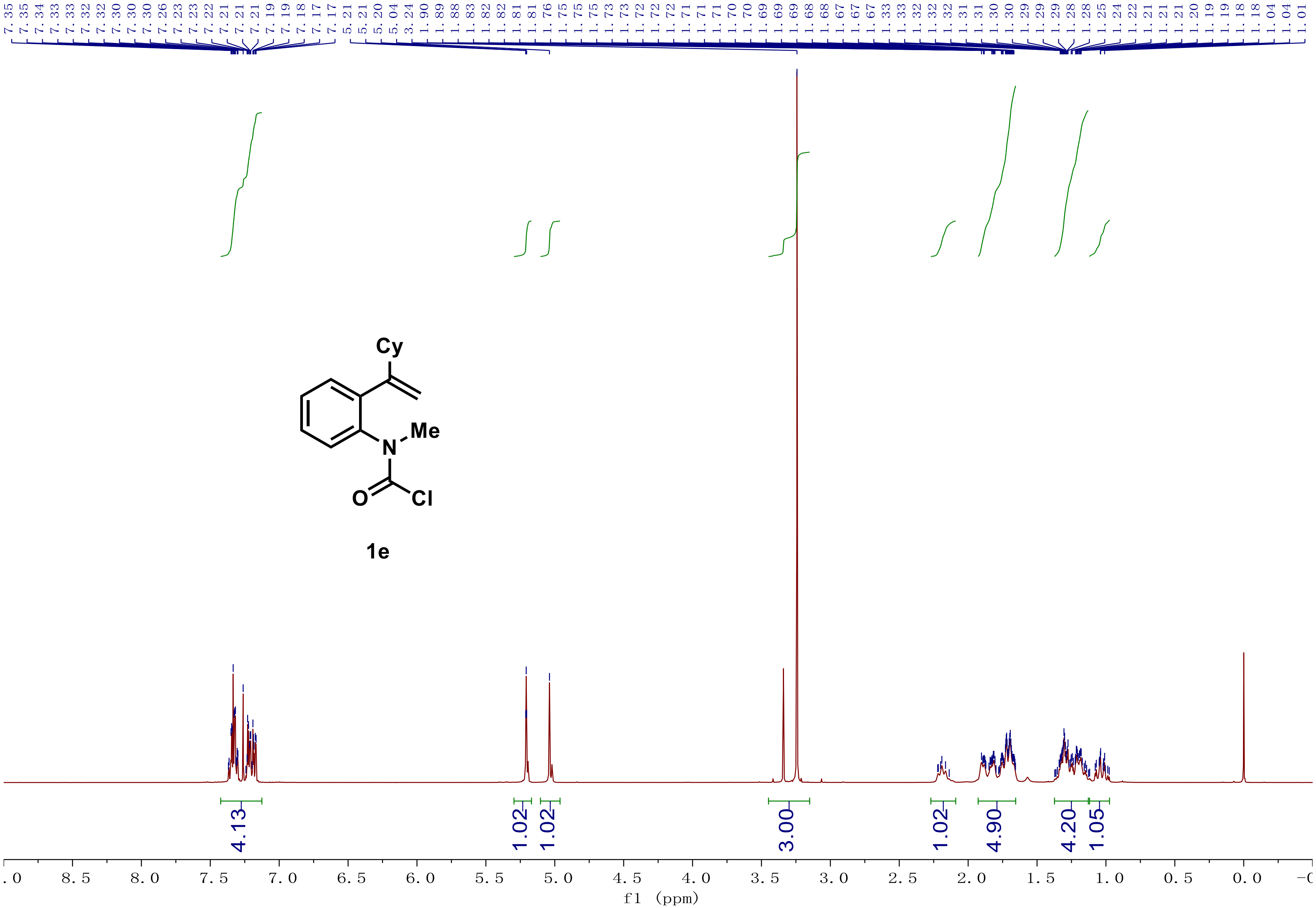

Supplementary Figure 44

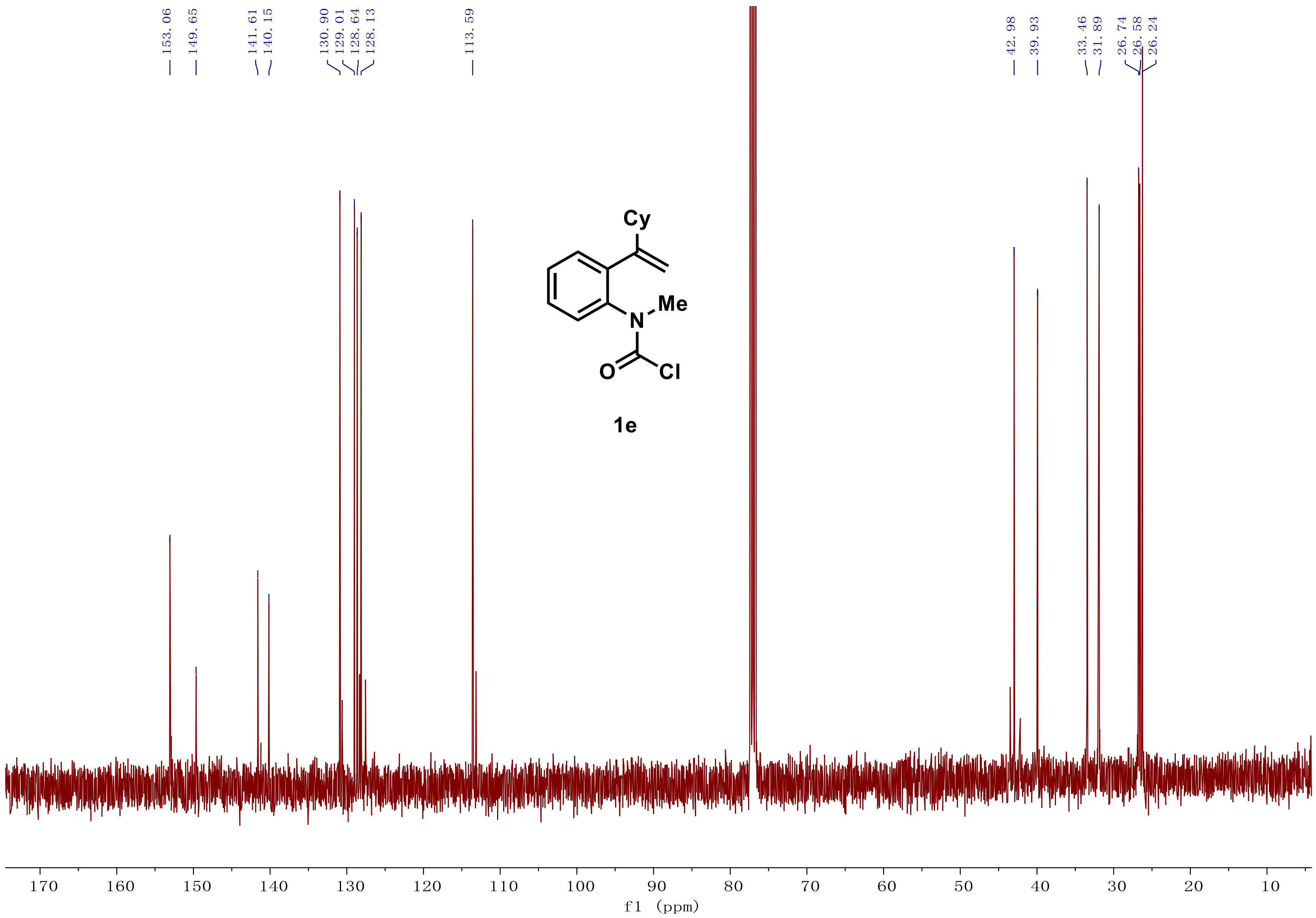

Supplementary Figure 45

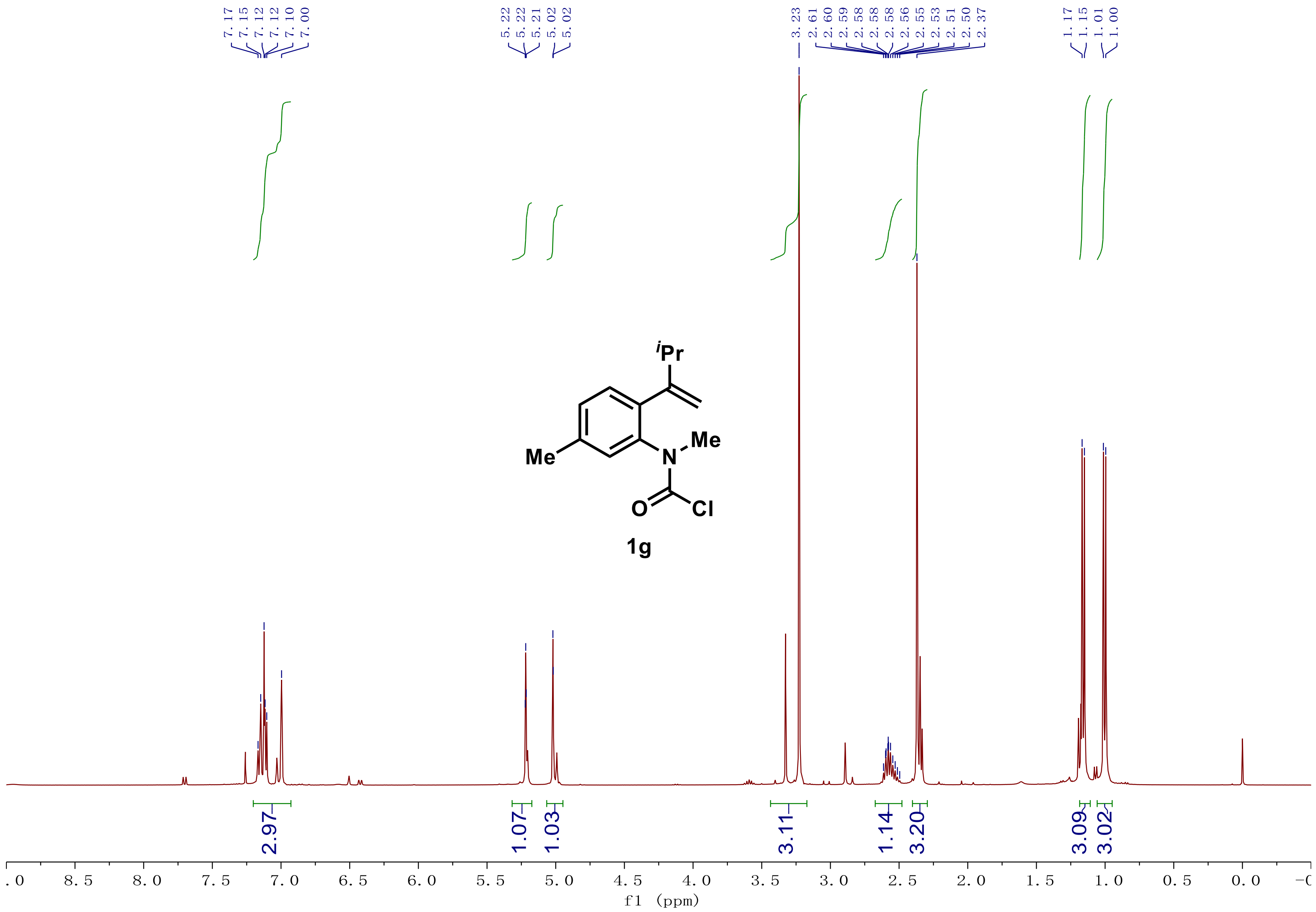

Supplementary Figure 46

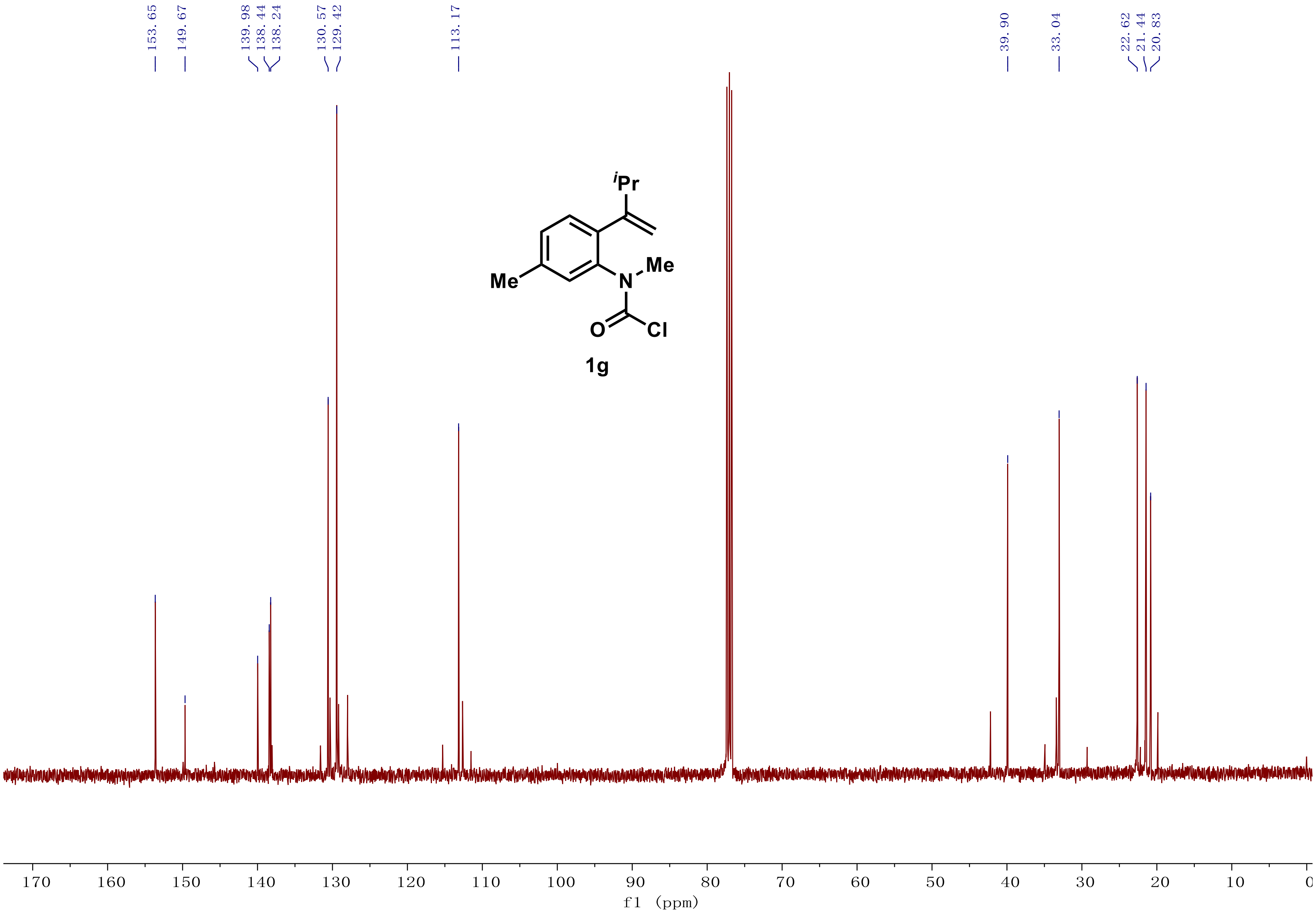

Supplementary Figure 47

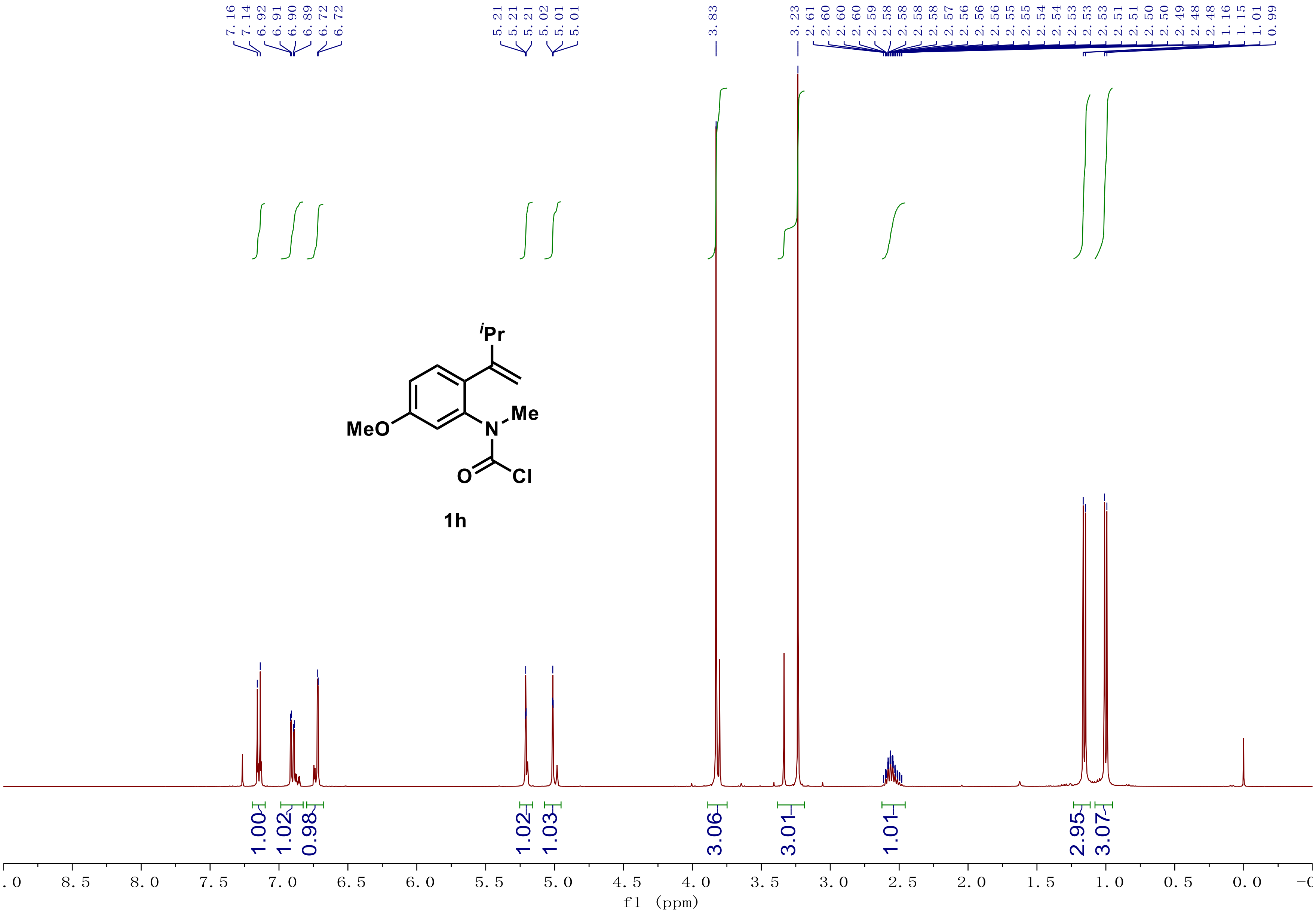

Supplementary Figure 48

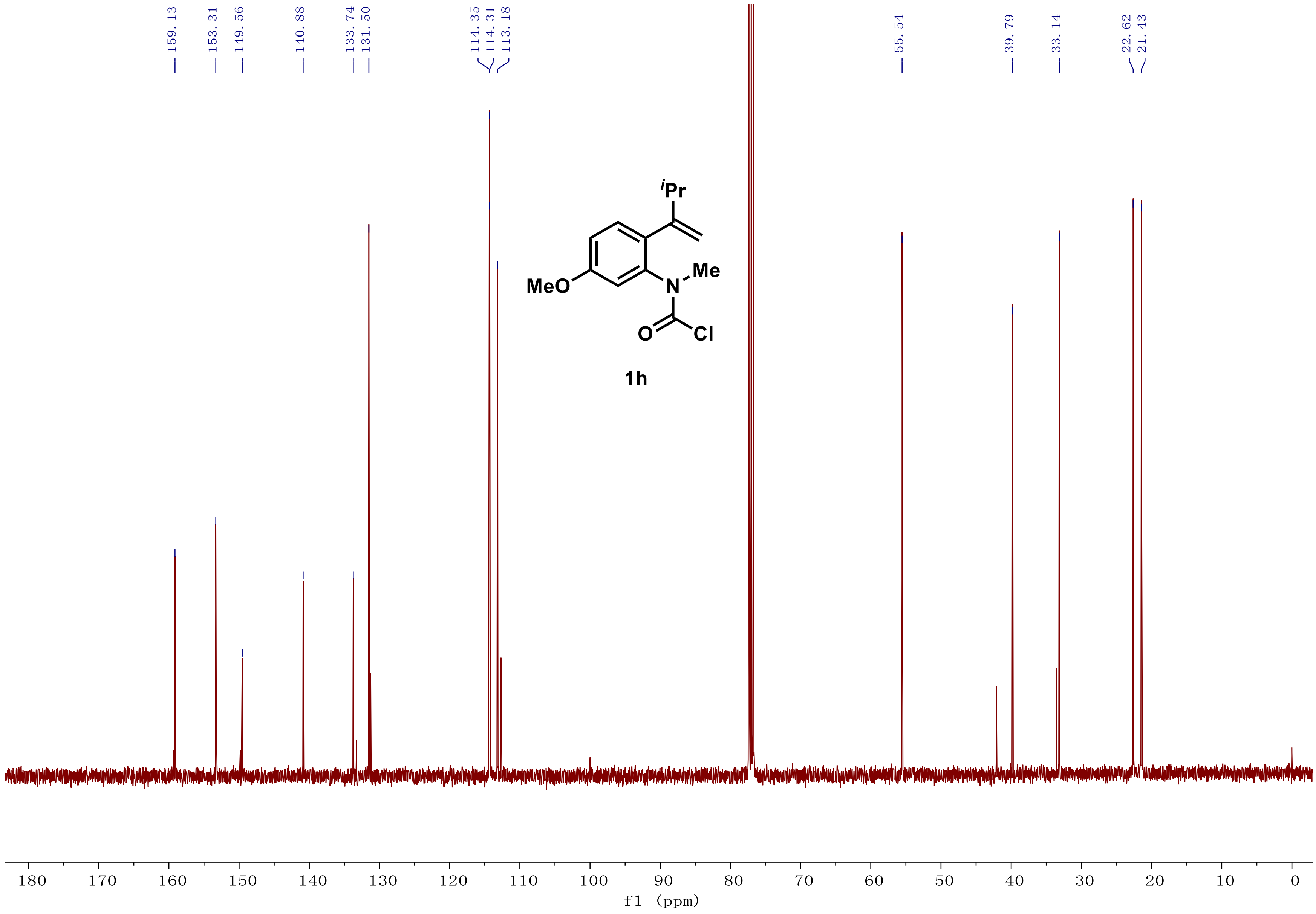

Supplementary Figure 49

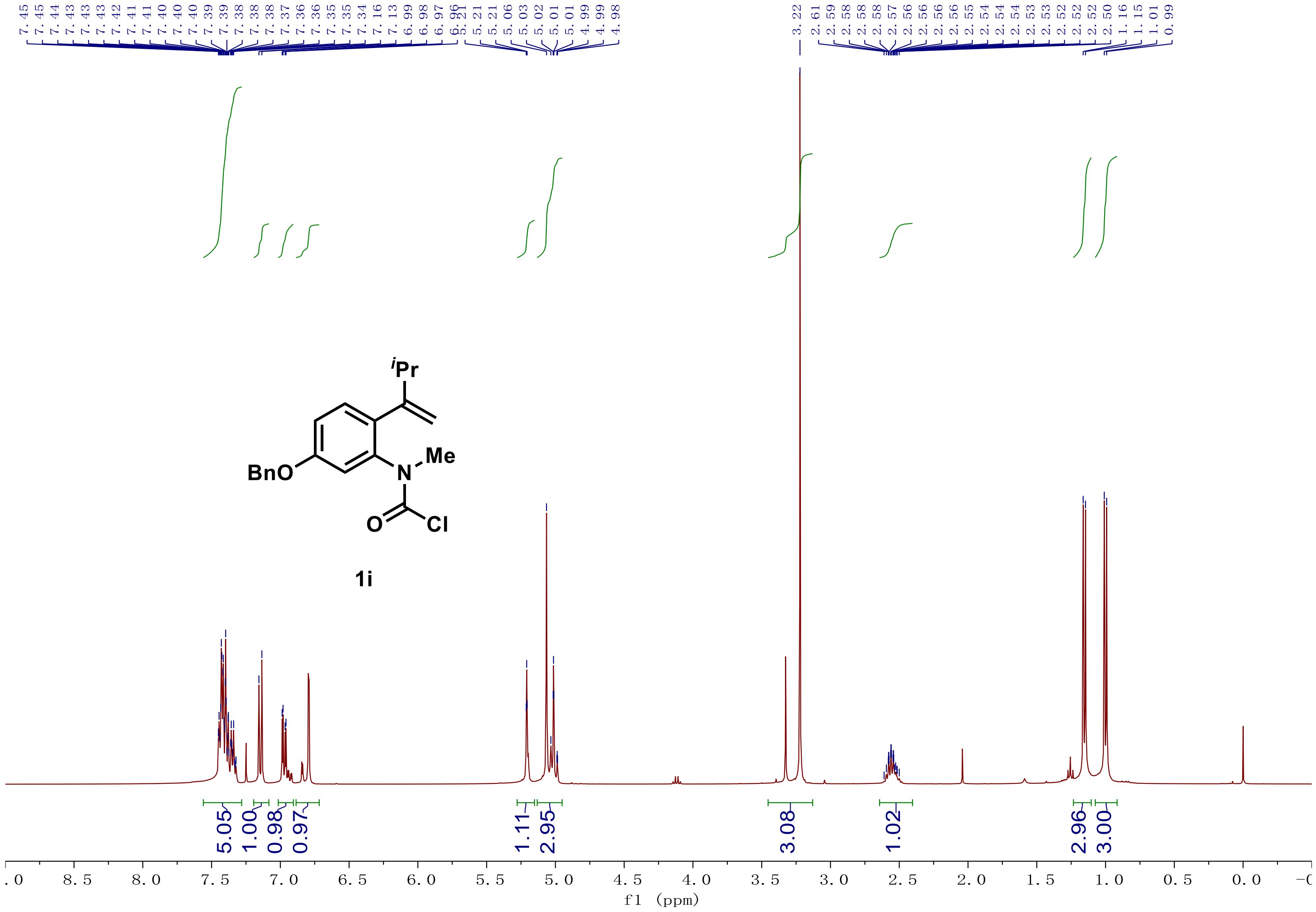

Supplementary Figure 50

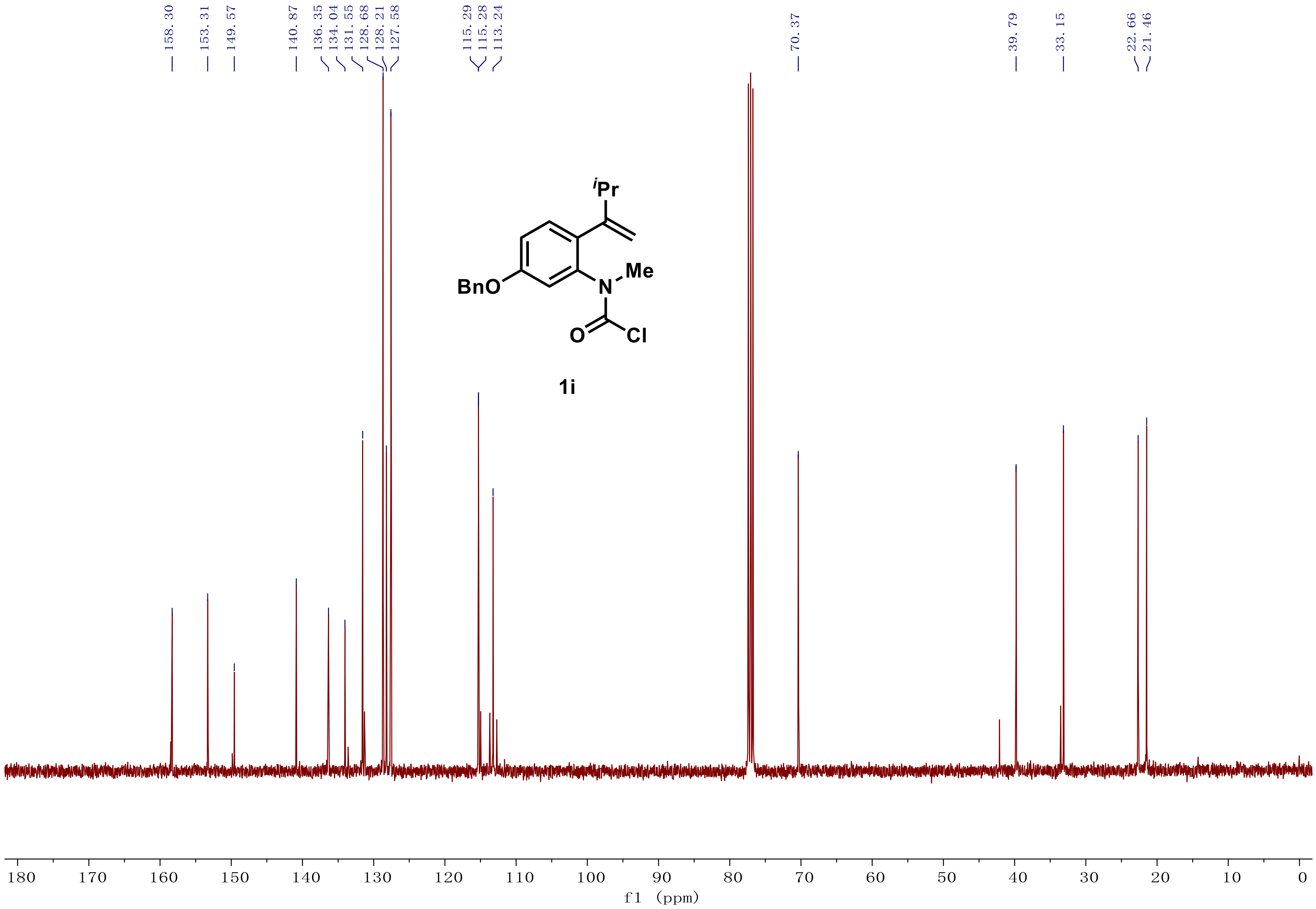

1i

Supplementary Figure 51

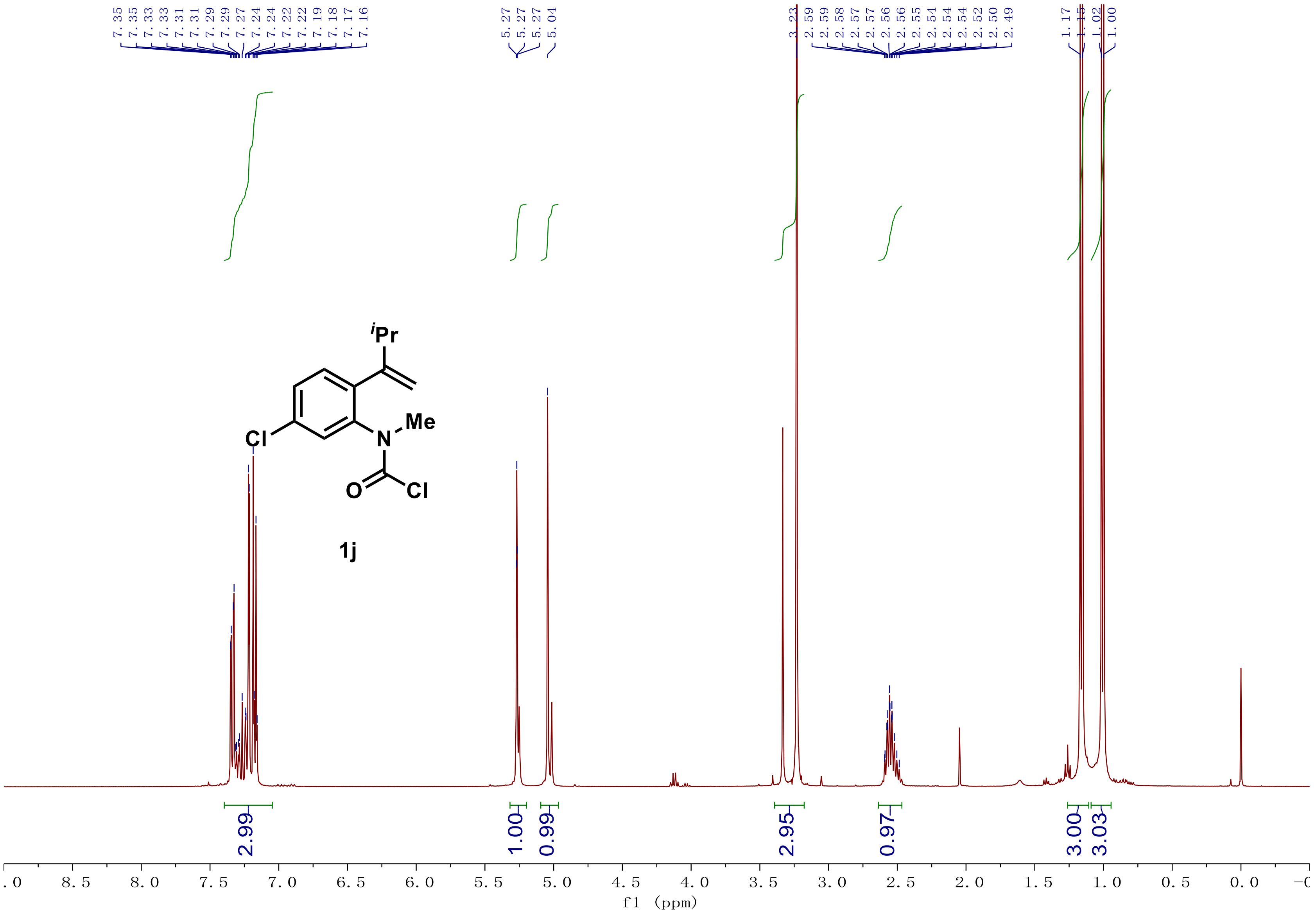

Supplementary Figure 52

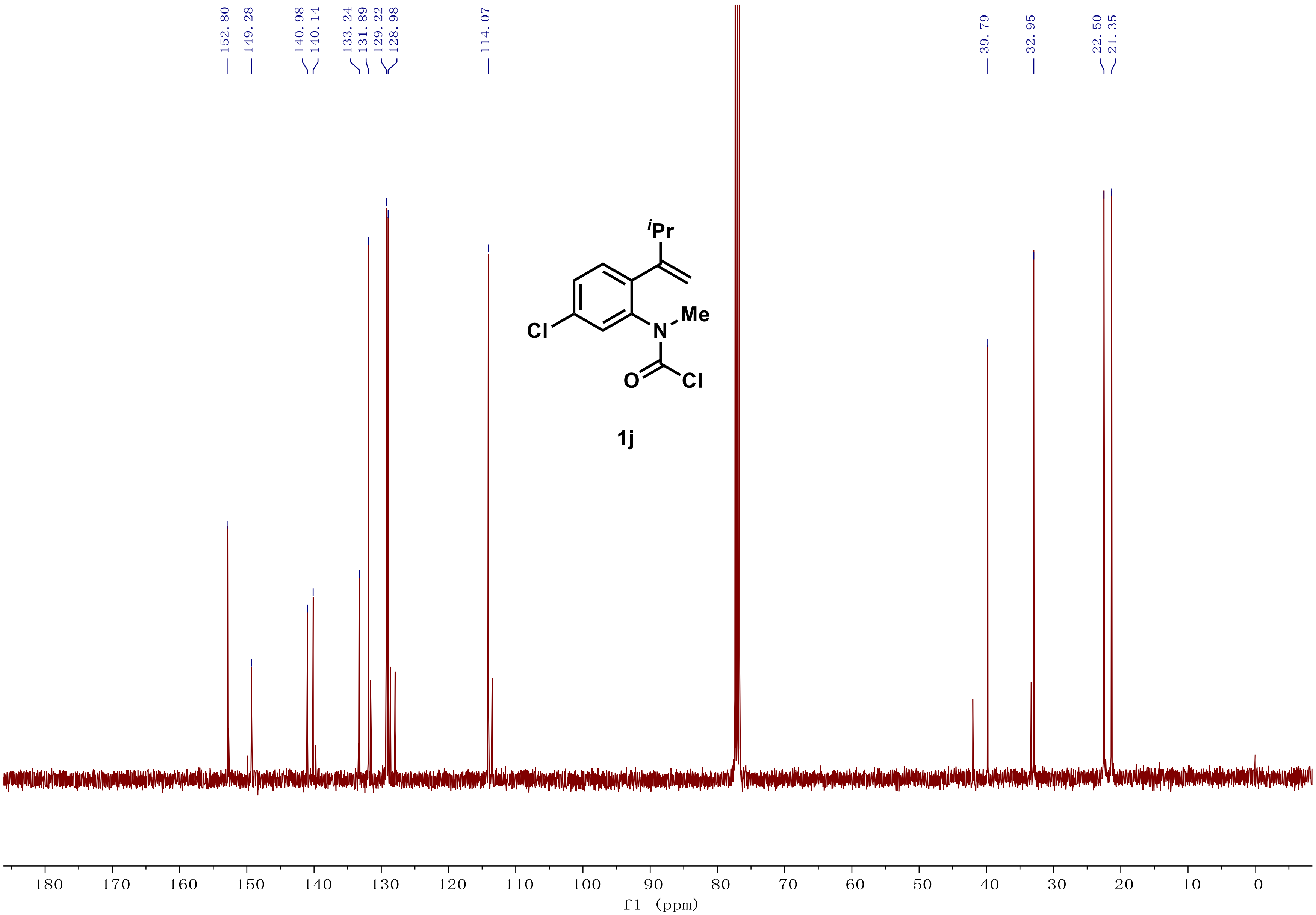

1j

Supplementary Figure 53

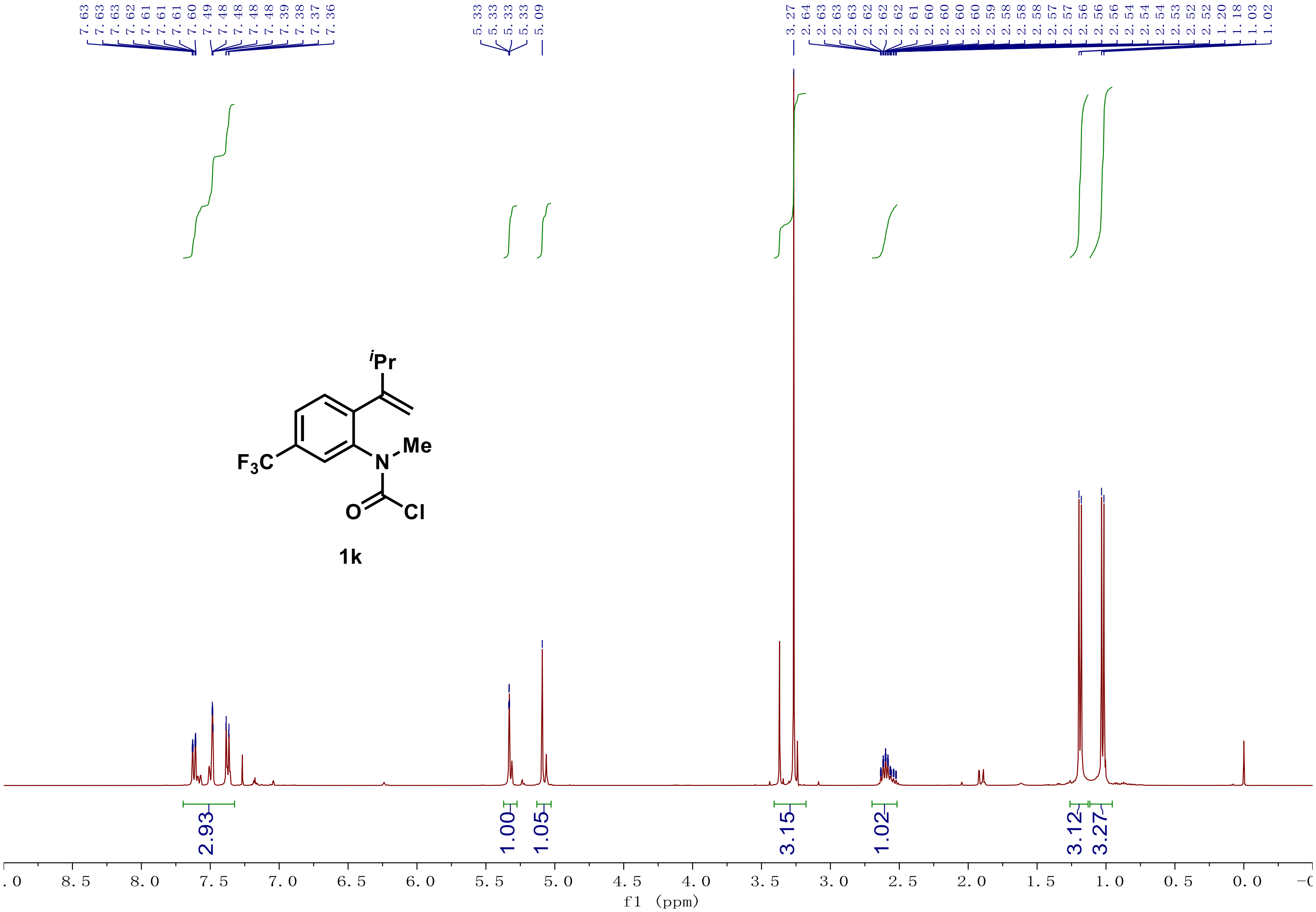

Supplementary Figure 54

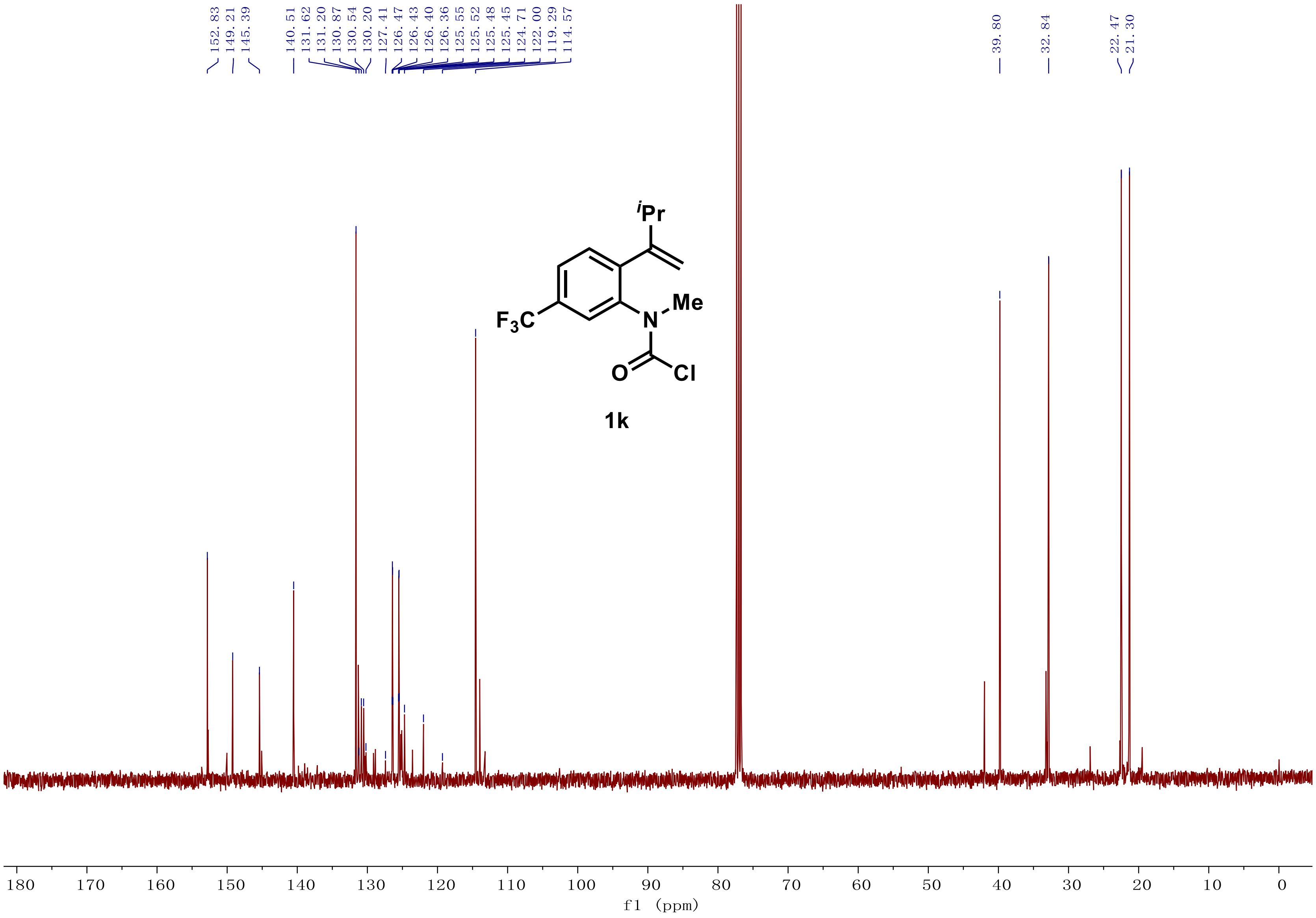

Supplementary Figure 55

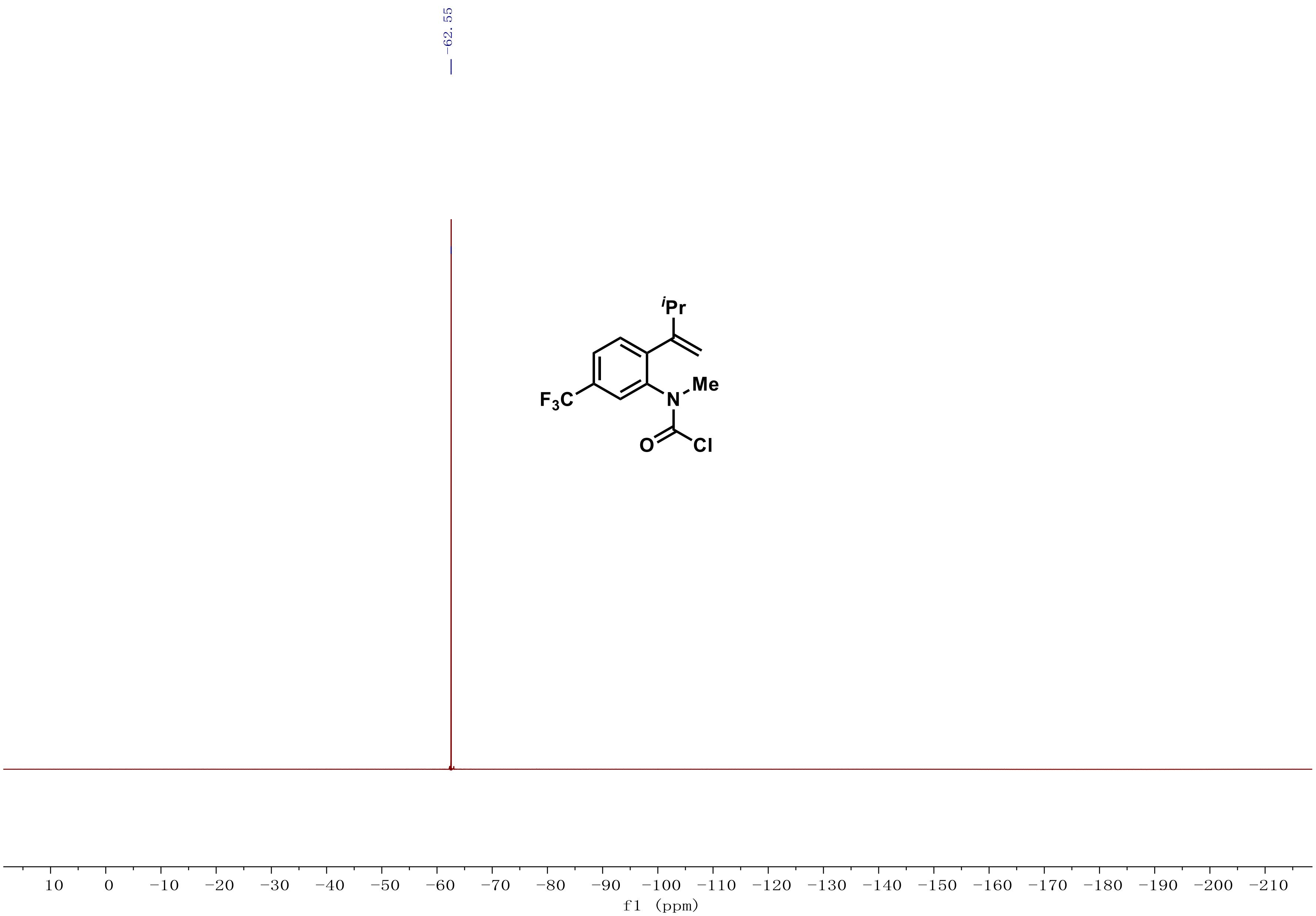

Supplementary Figure 56

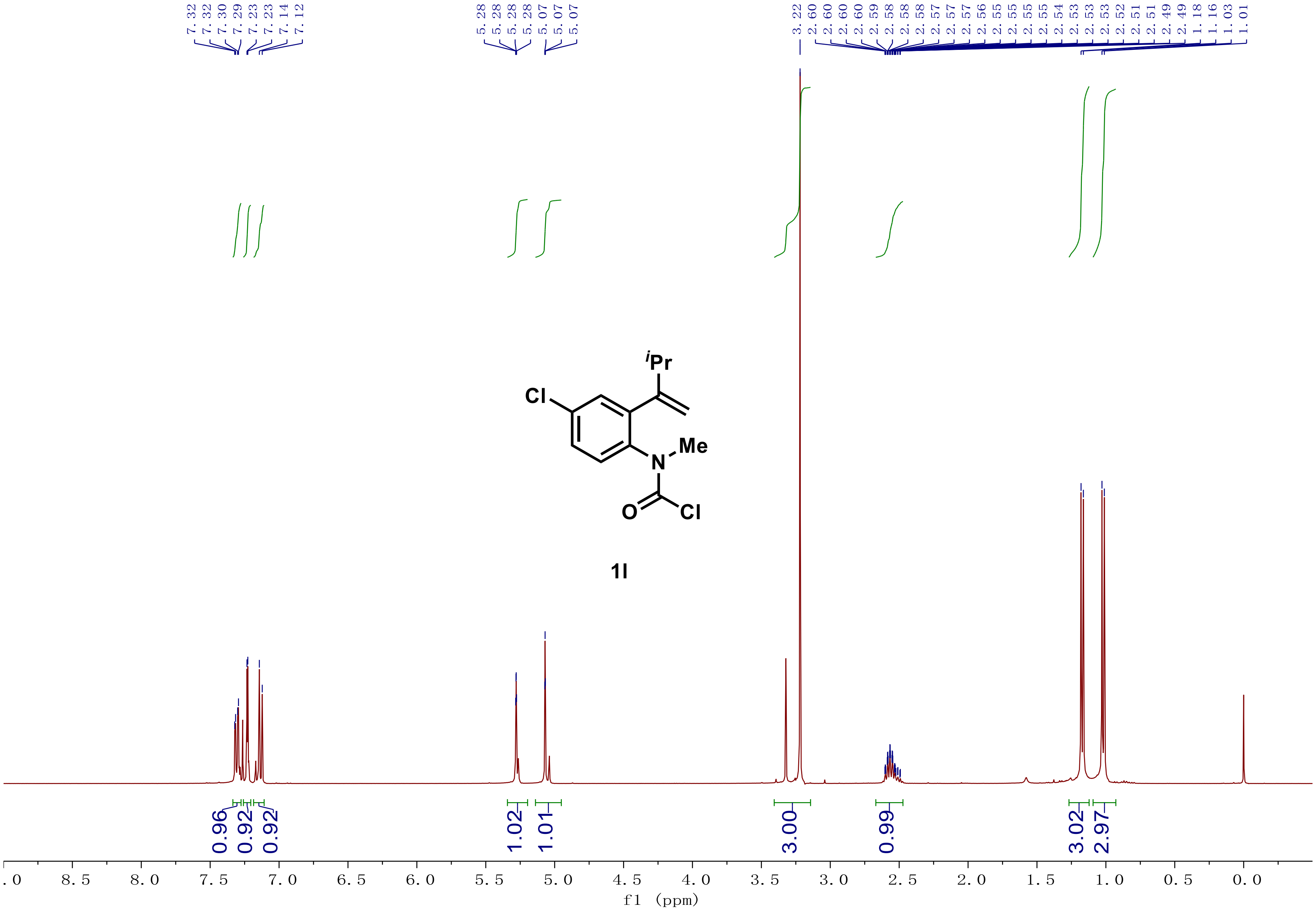

Supplementary Figure 57

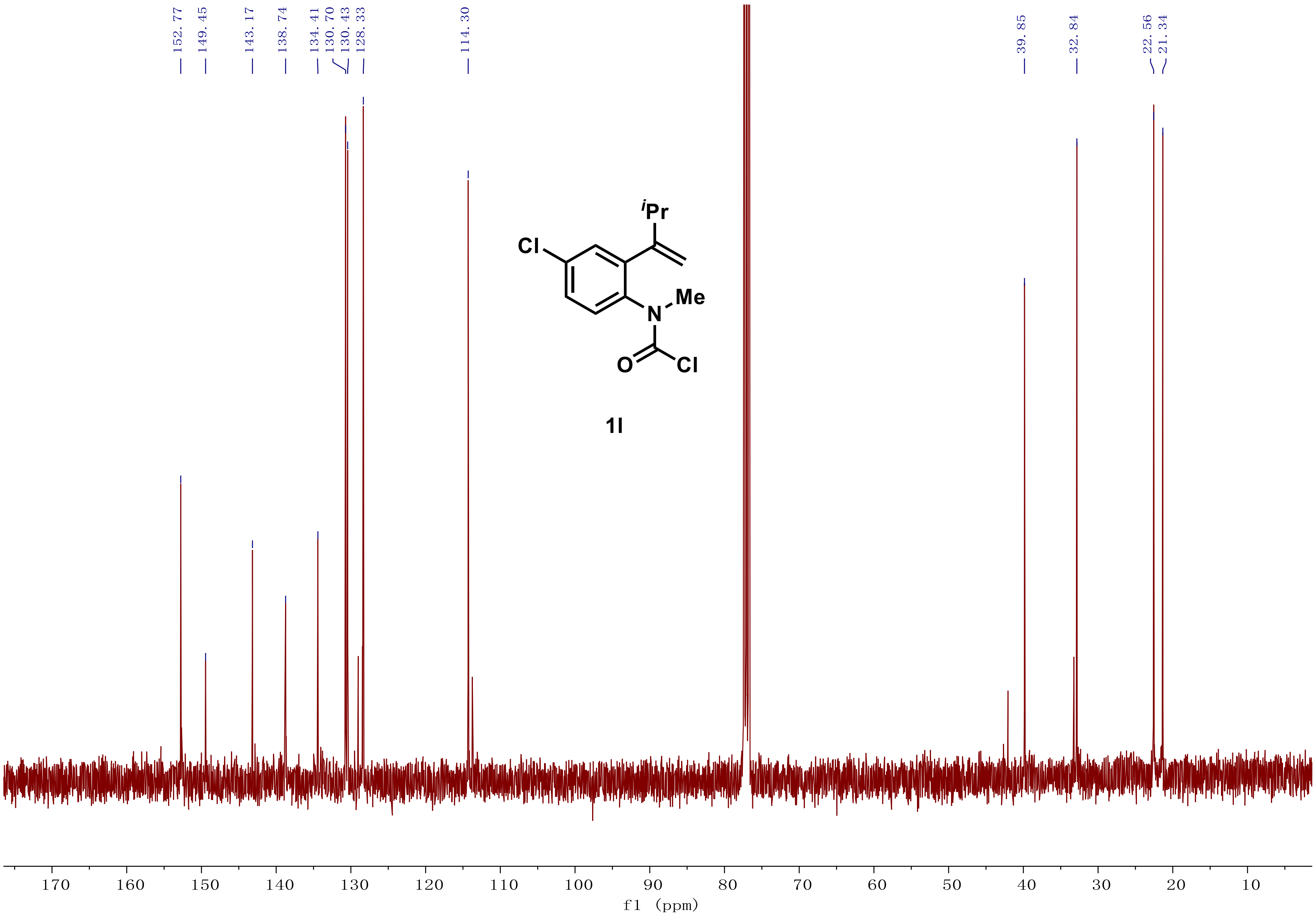

Supplementary Figure 58

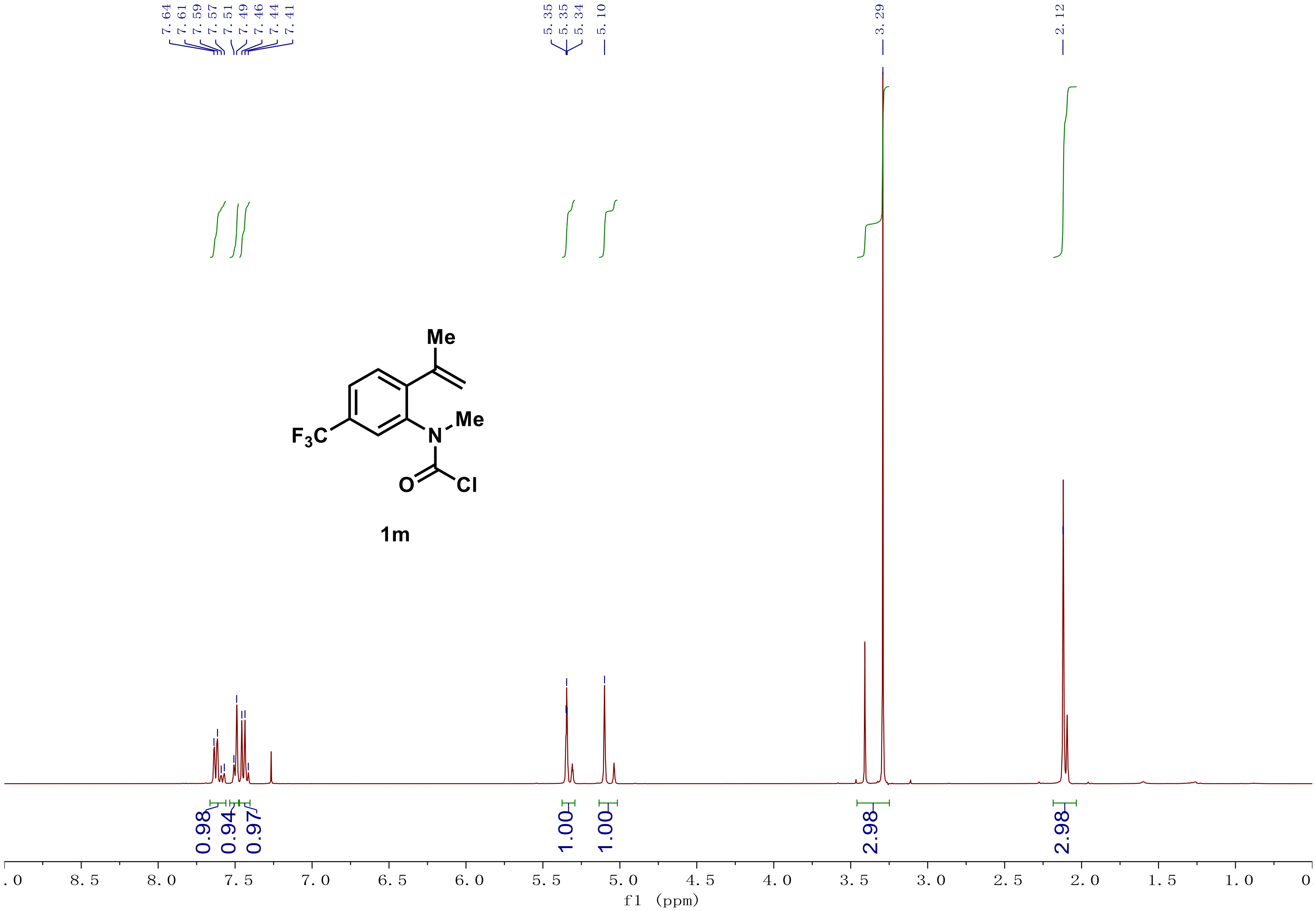

Supplementary Figure 59

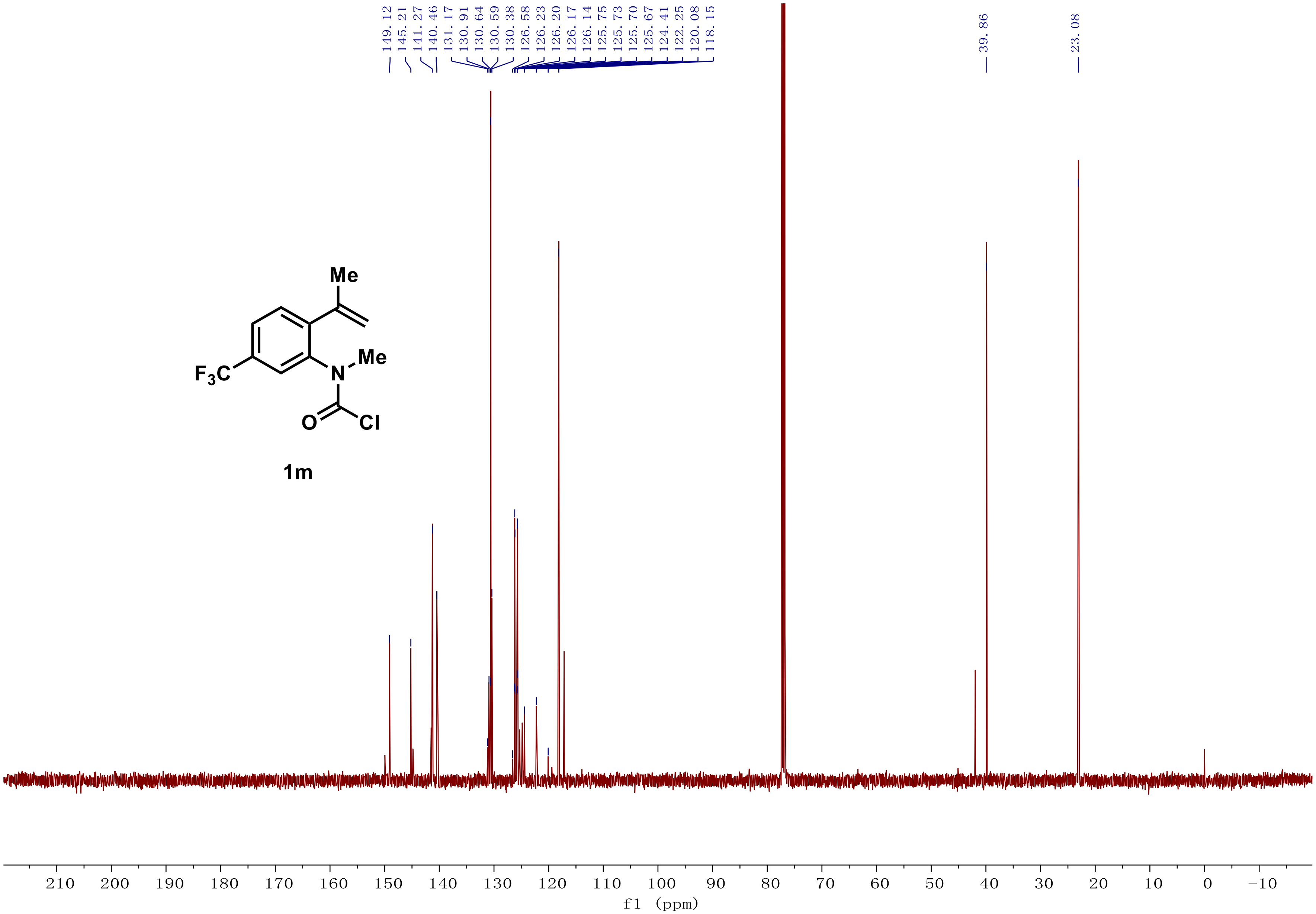

Supplementary Figure 60

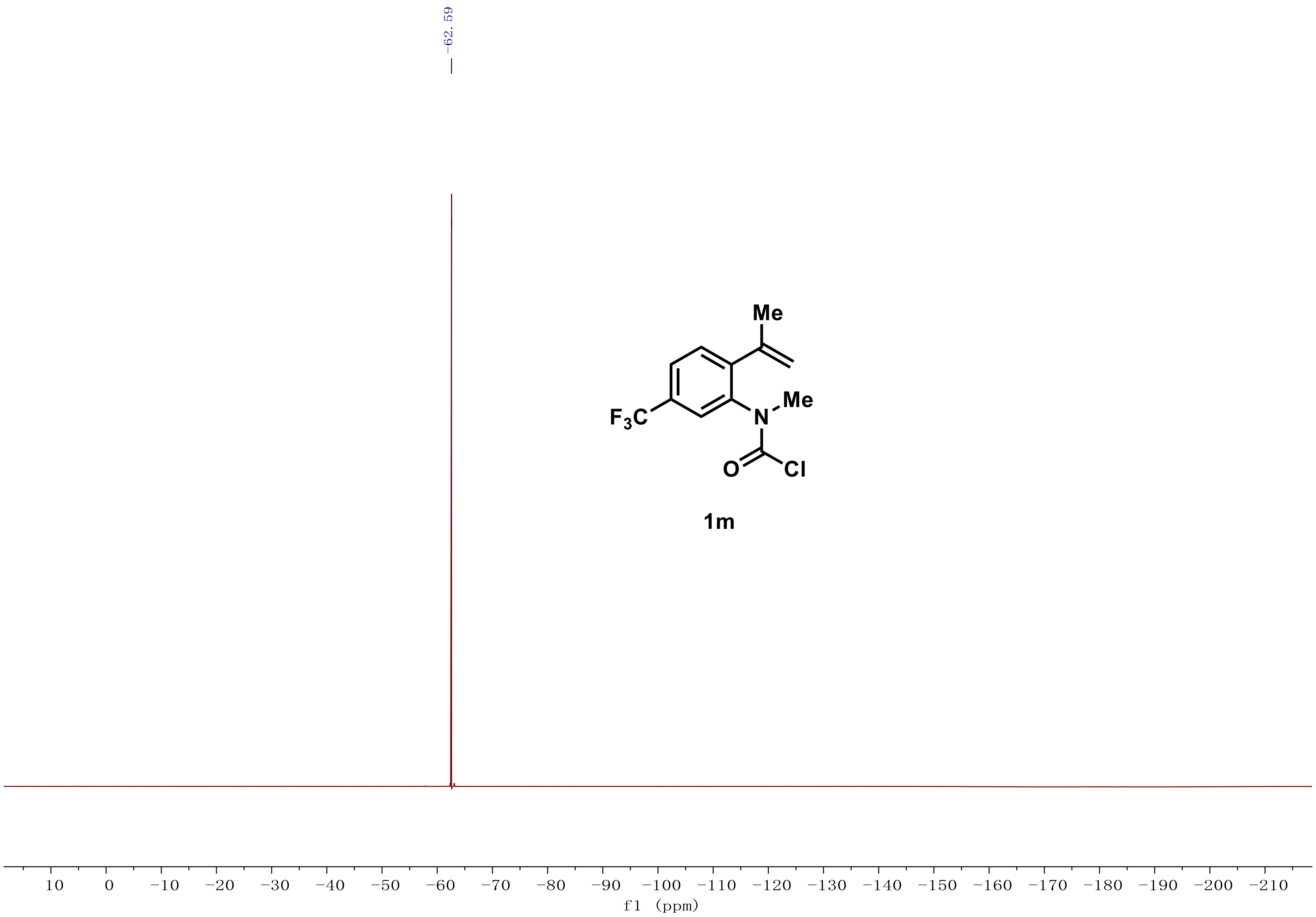

Supplementary Figure 61

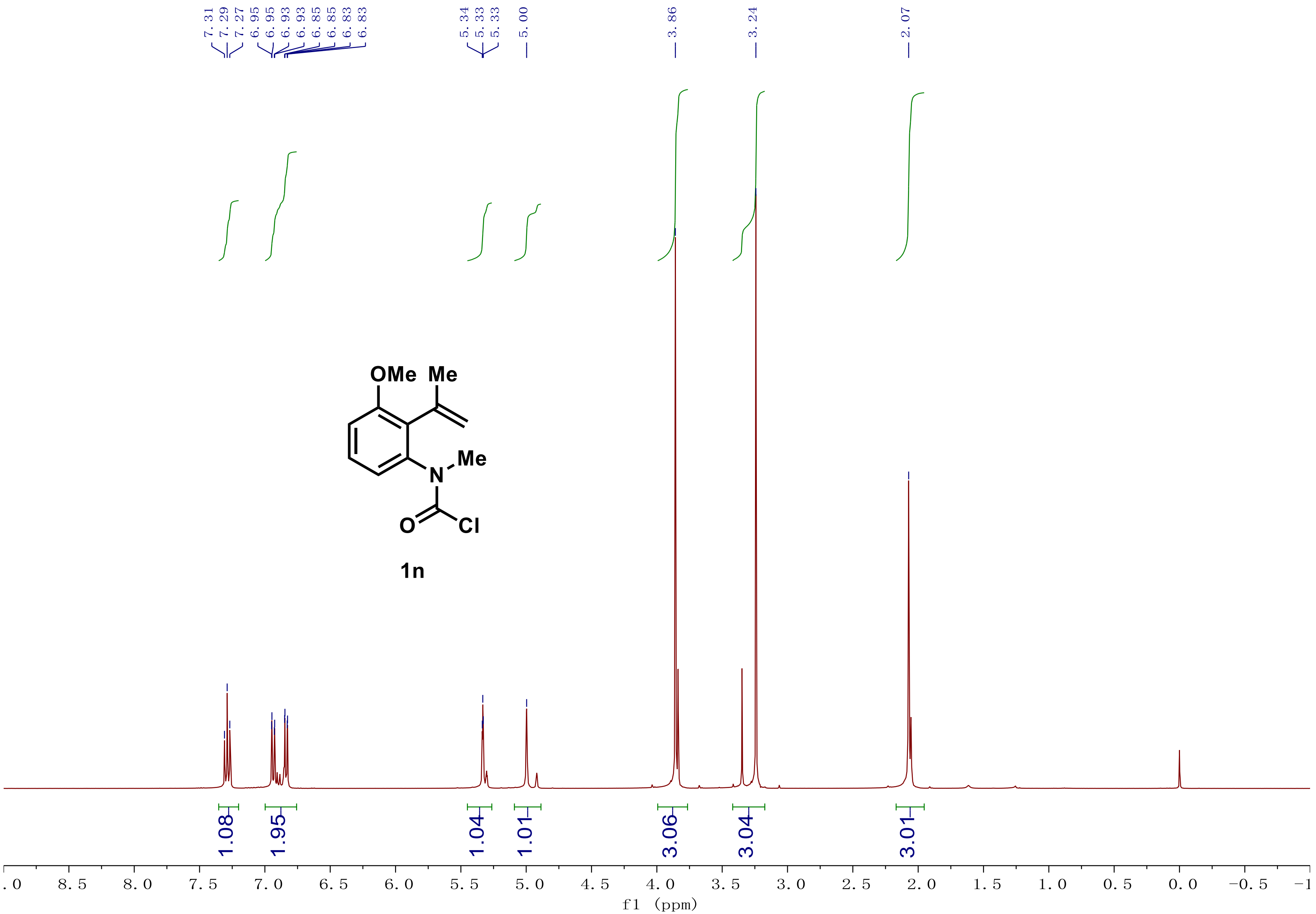

Supplementary Figure 62

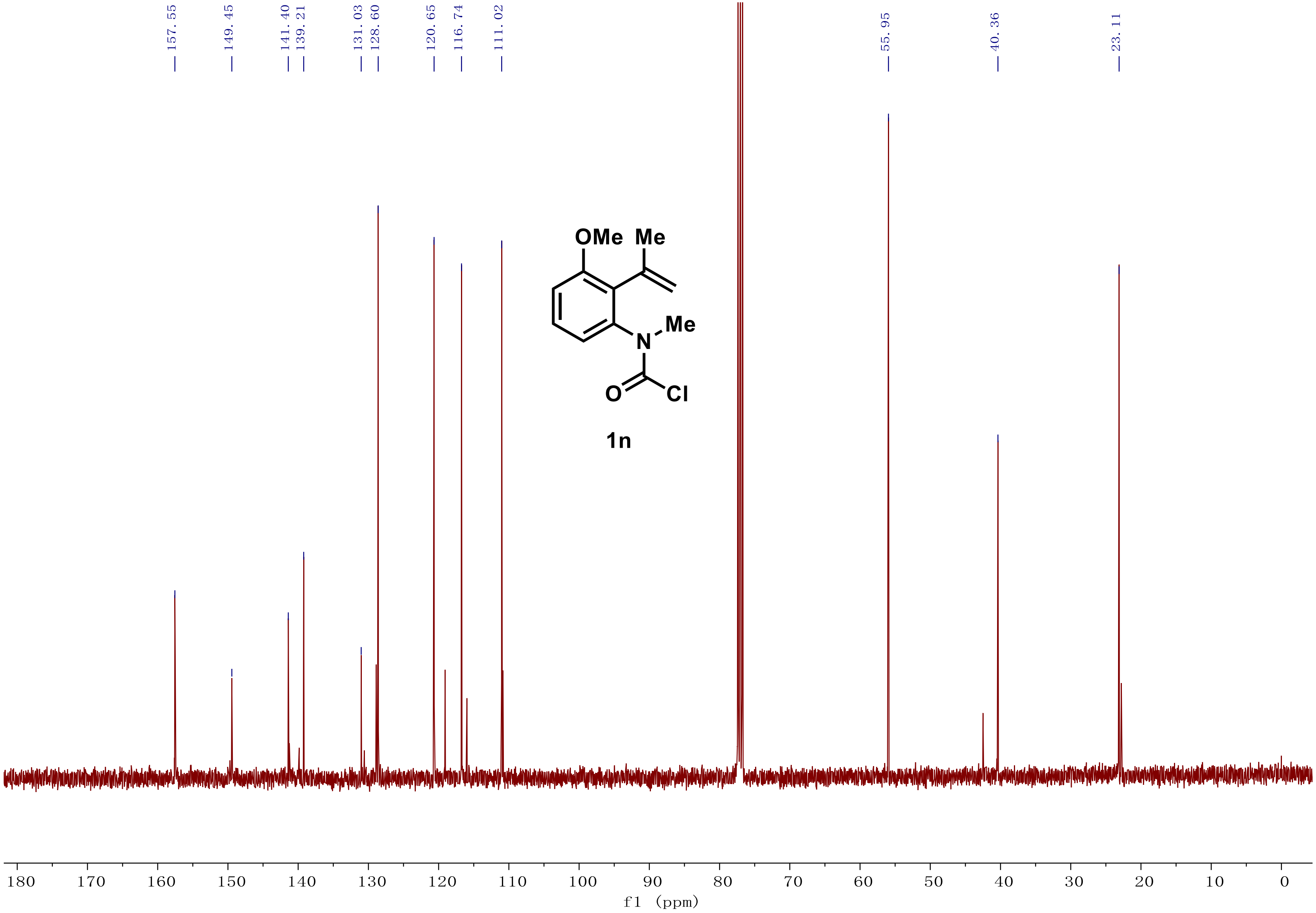

Supplementary Figure 63

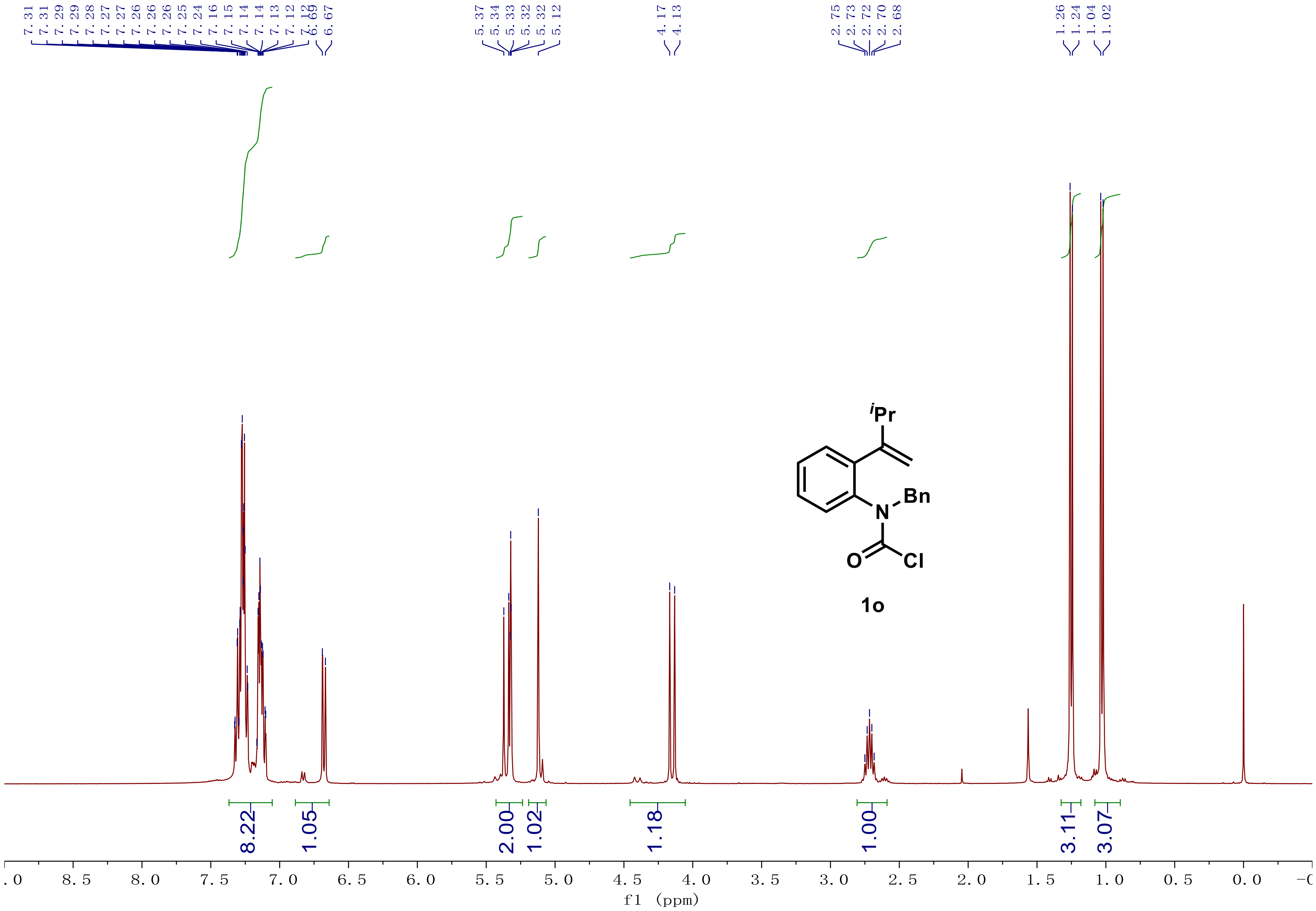

Supplementary Figure 64

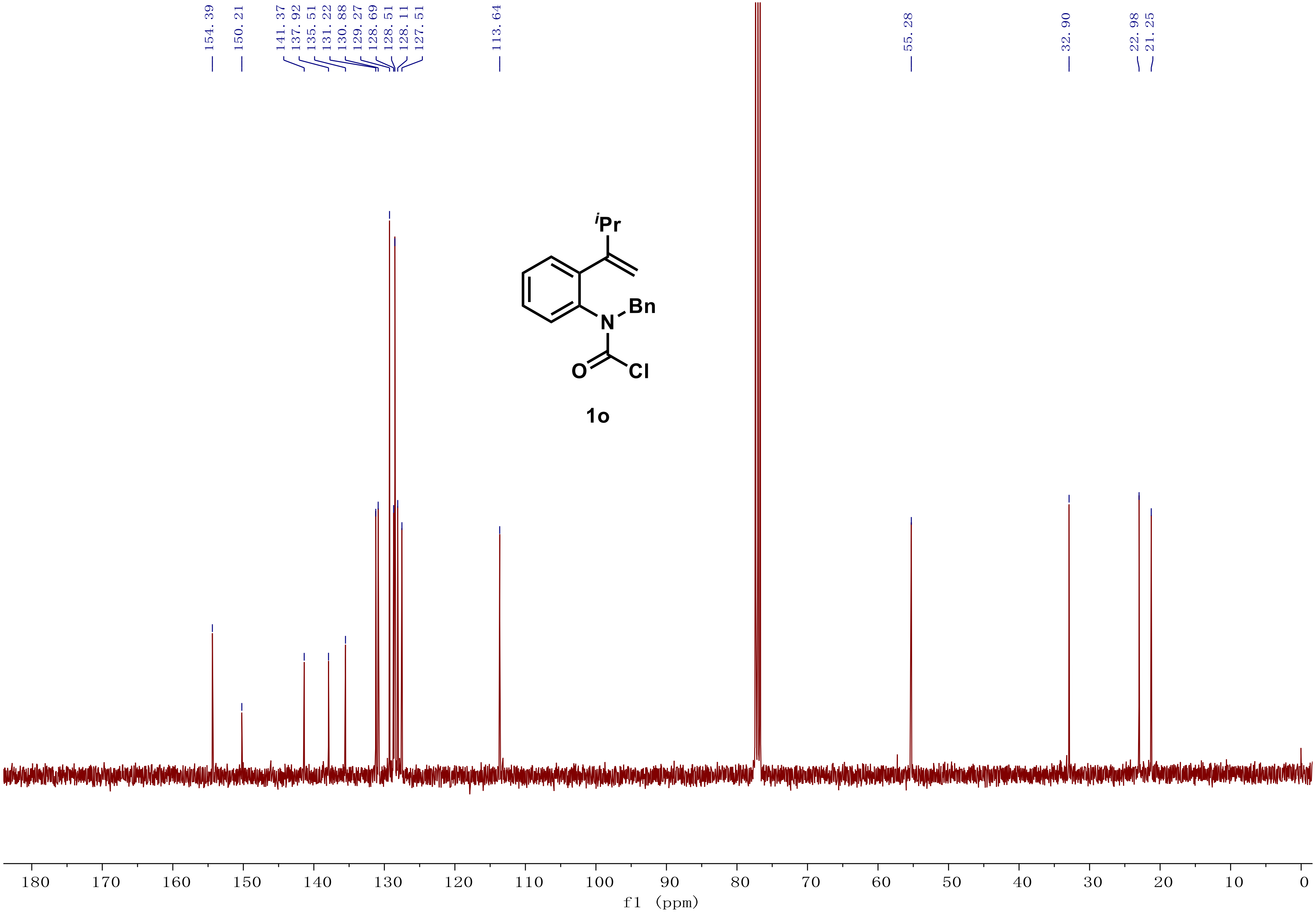

Supplementary Figure 65

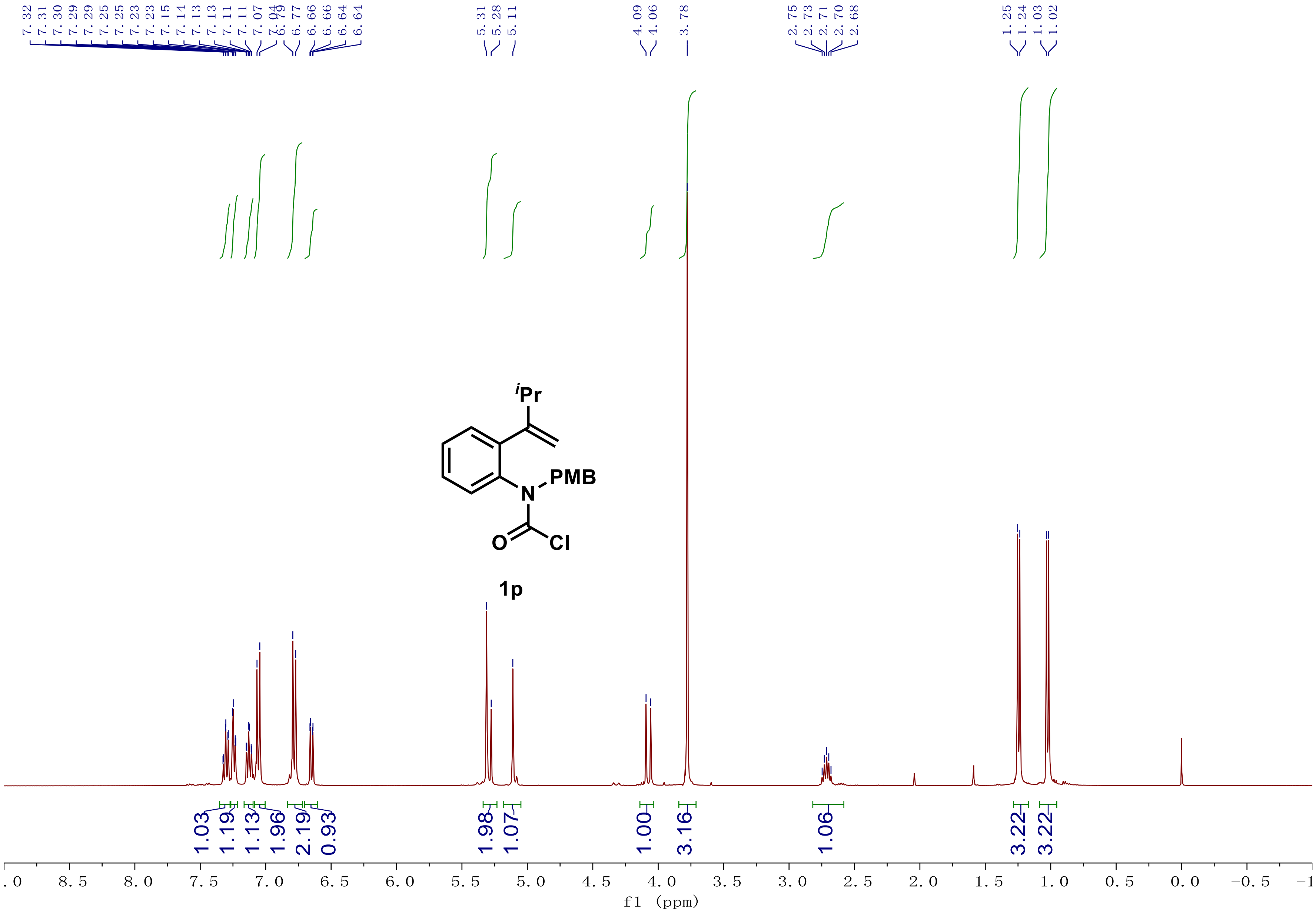

Supplementary Figure 66

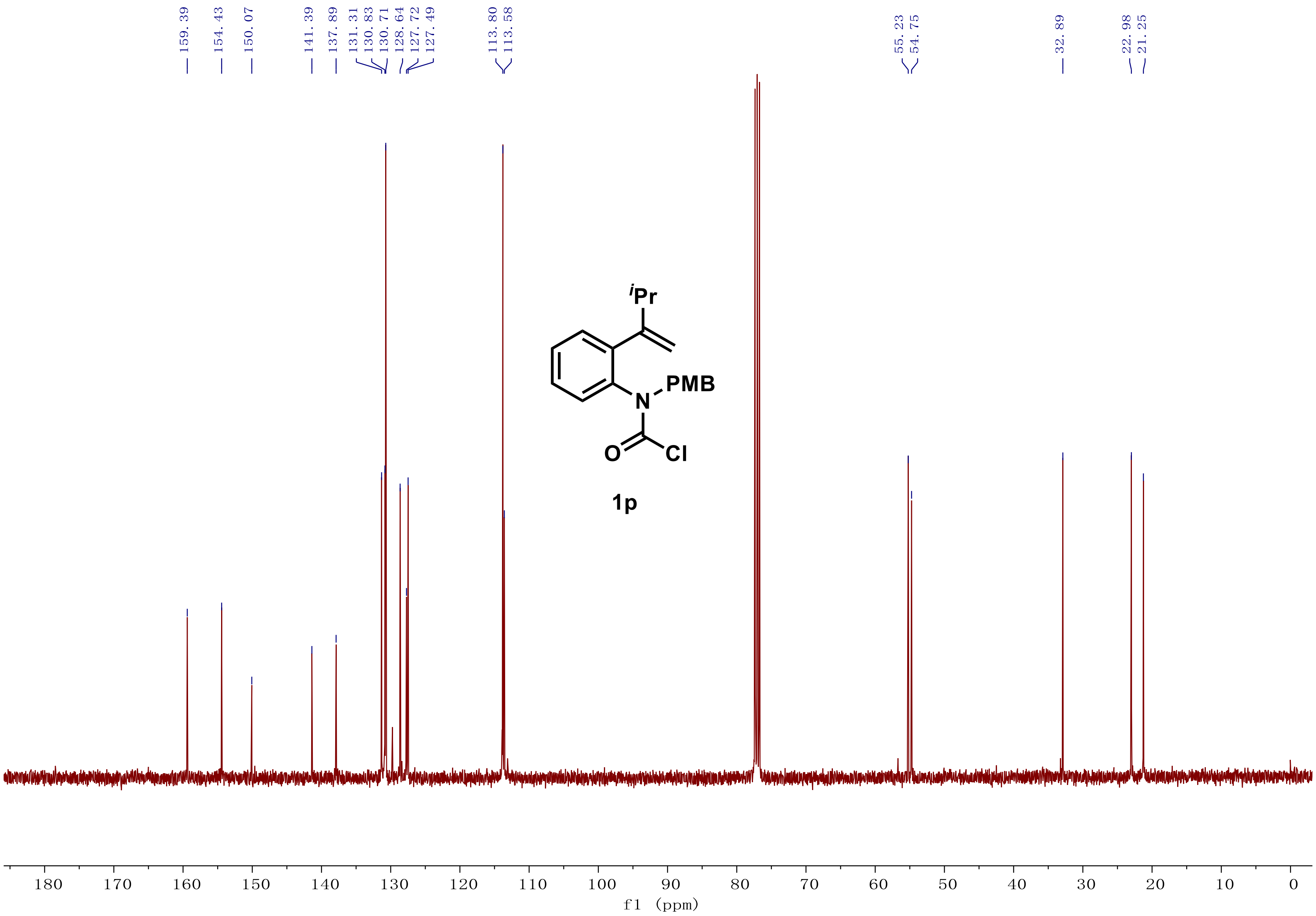

1p

Supplementary Figure 67

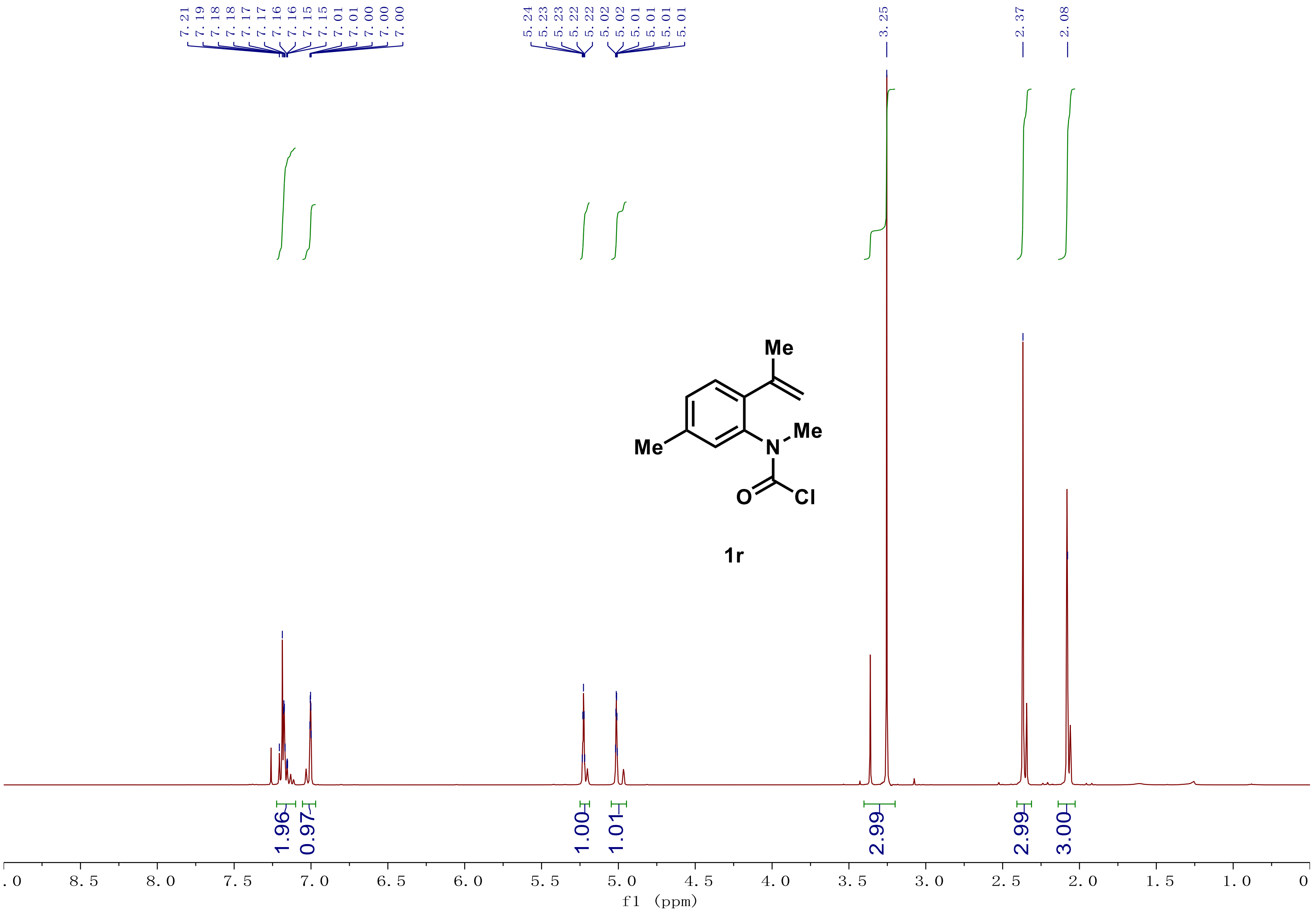

Supplementary Figure 68

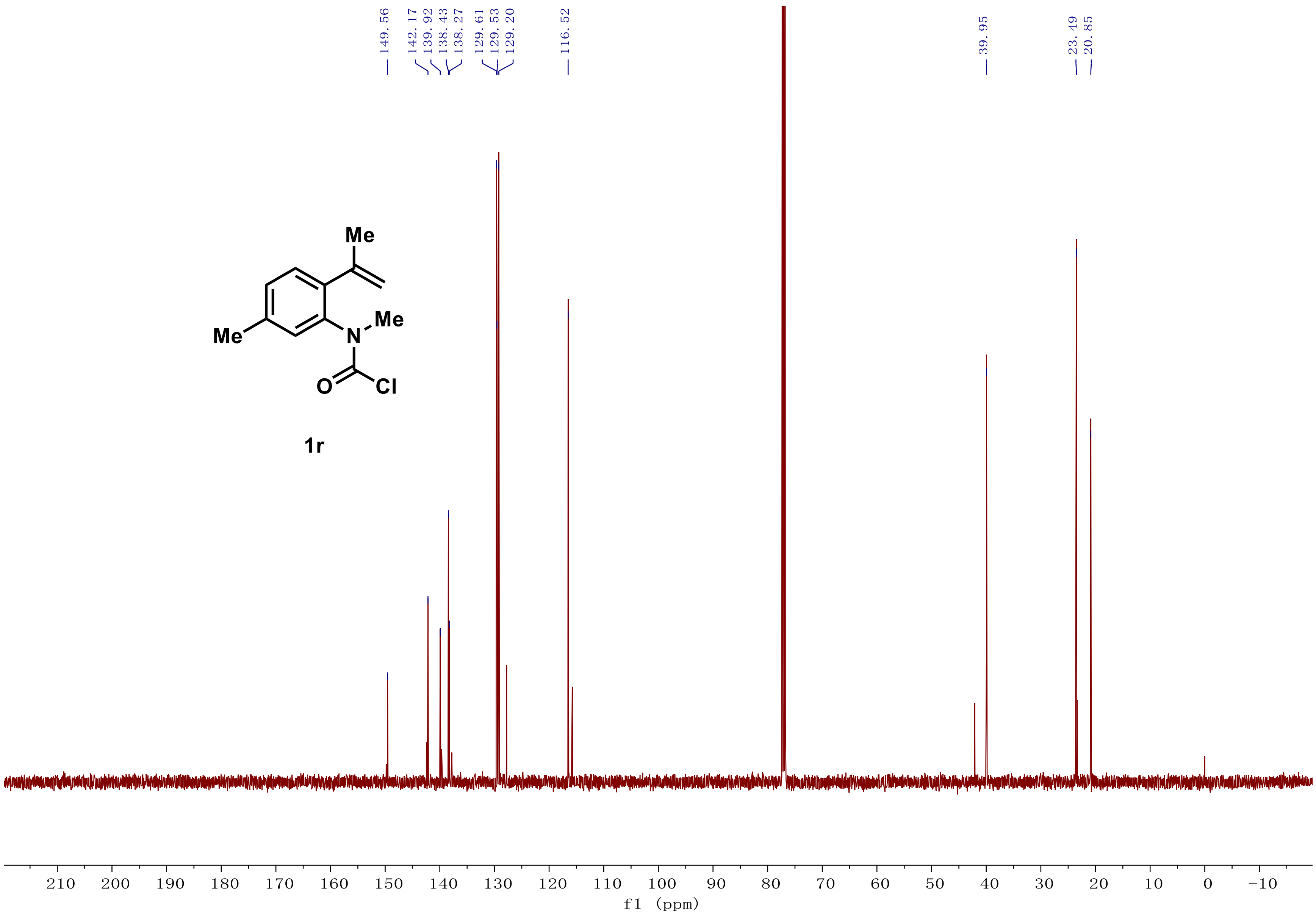

Supplementary Figure 69

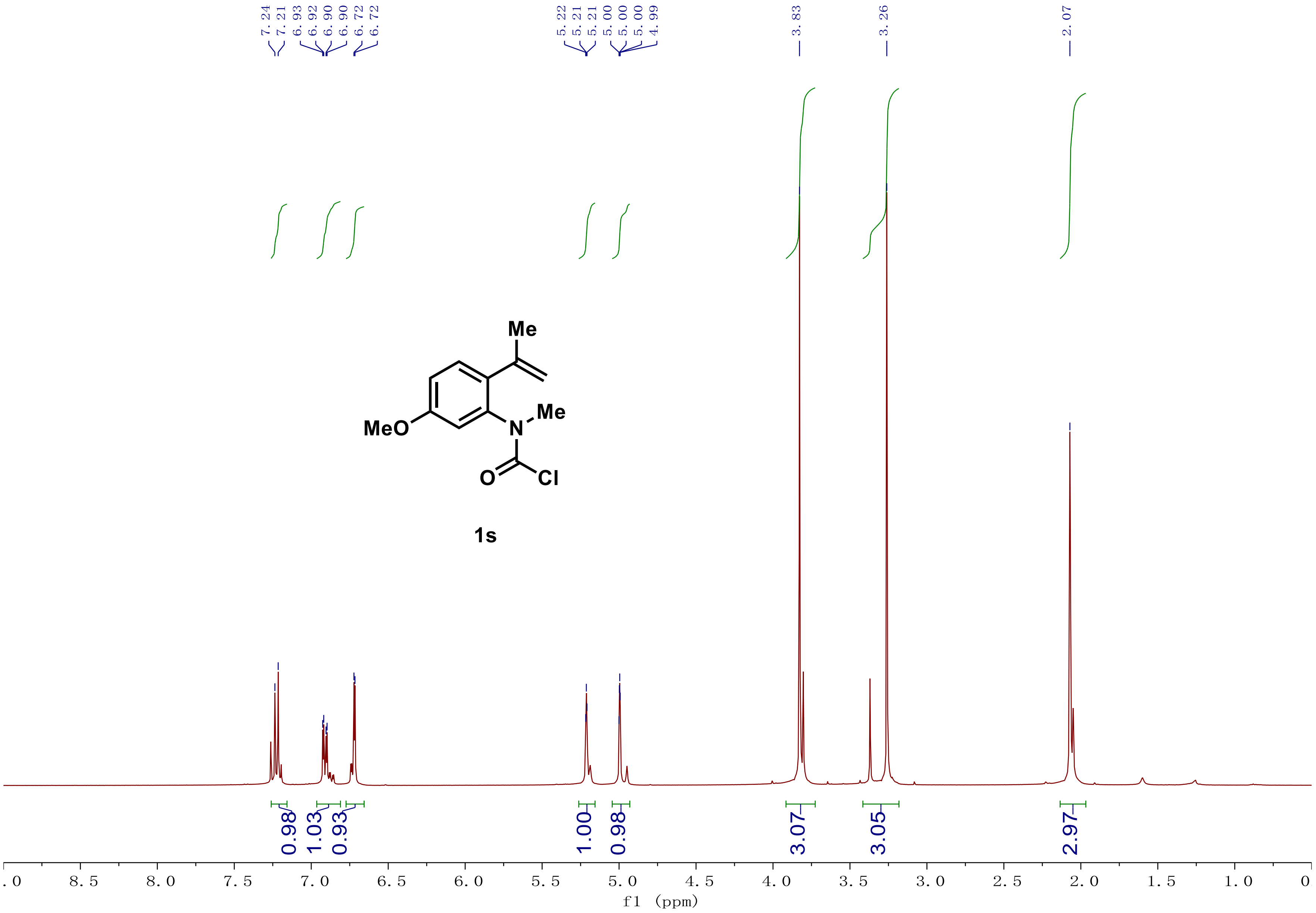

Supplementary Figure 70

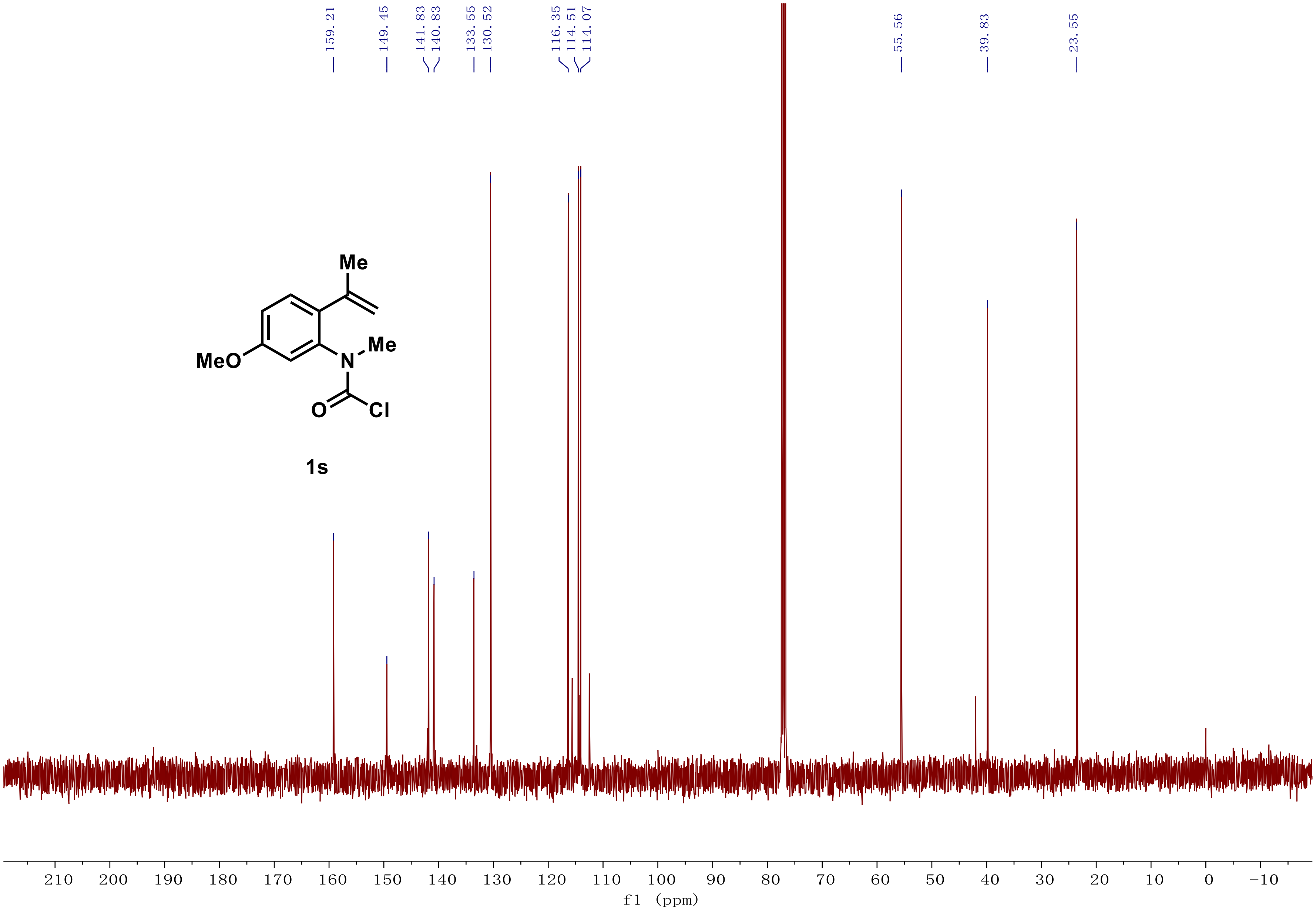

Supplementary Figure 71

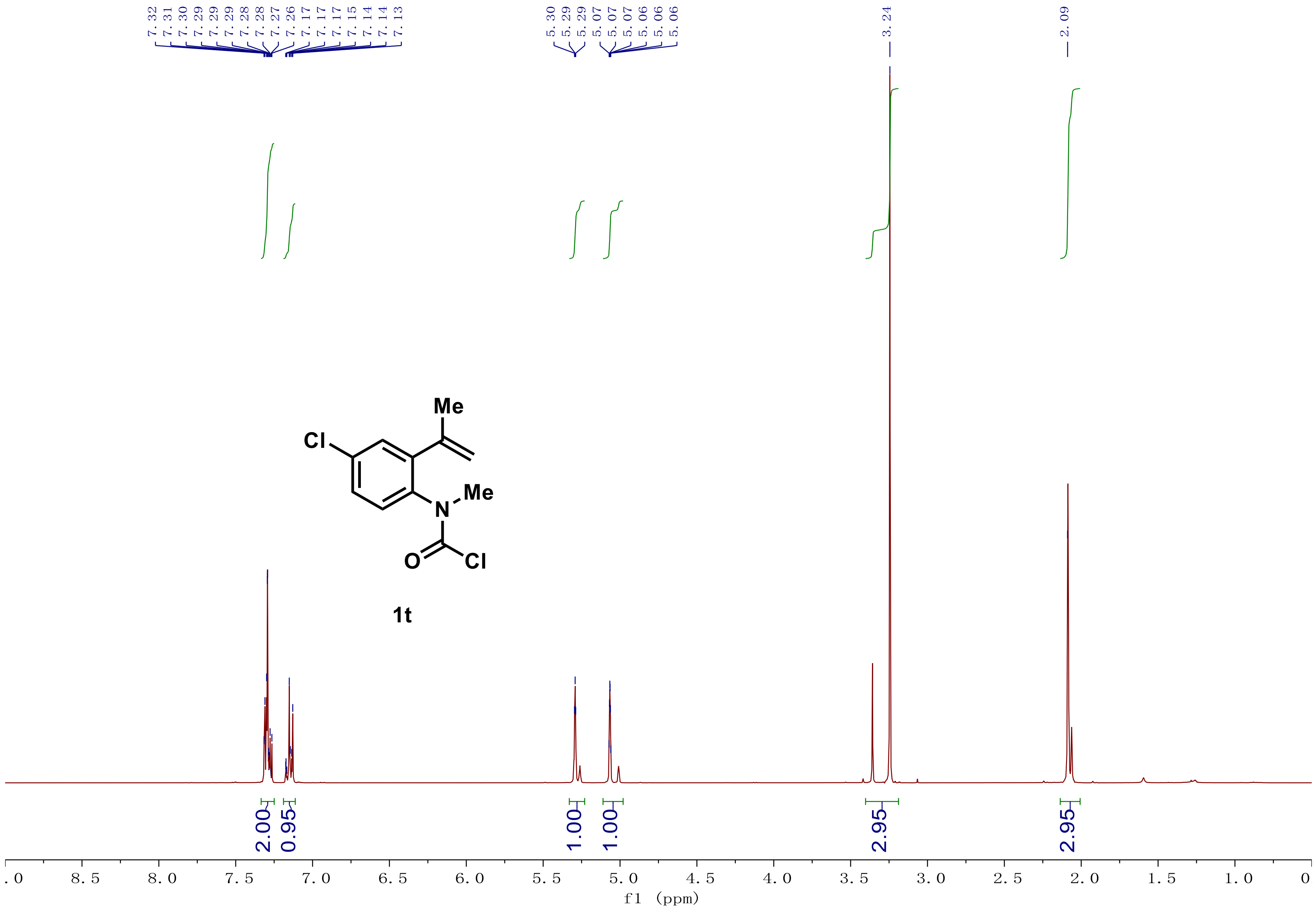

Supplementary Figure 72

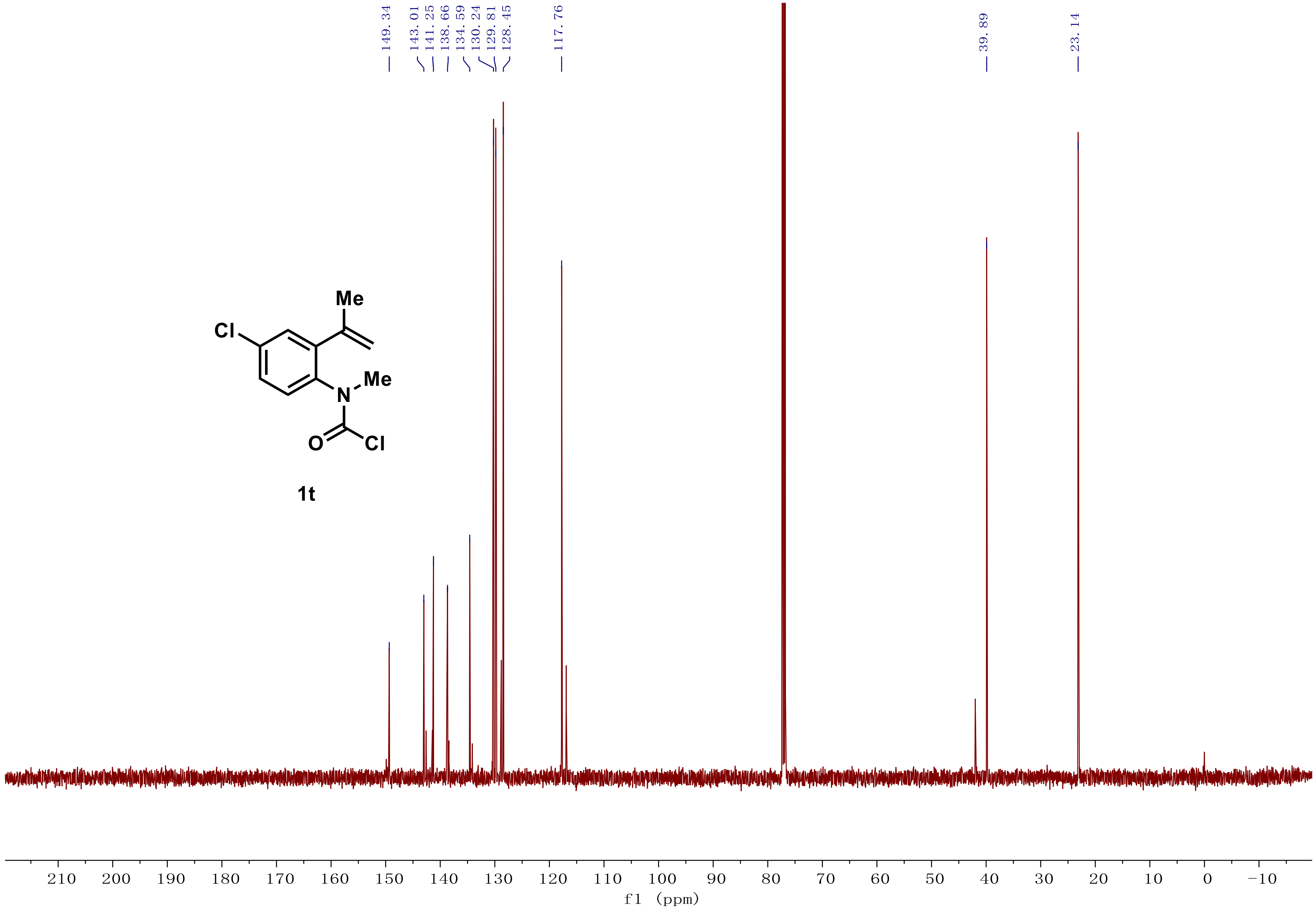

Supplementary Figure 73

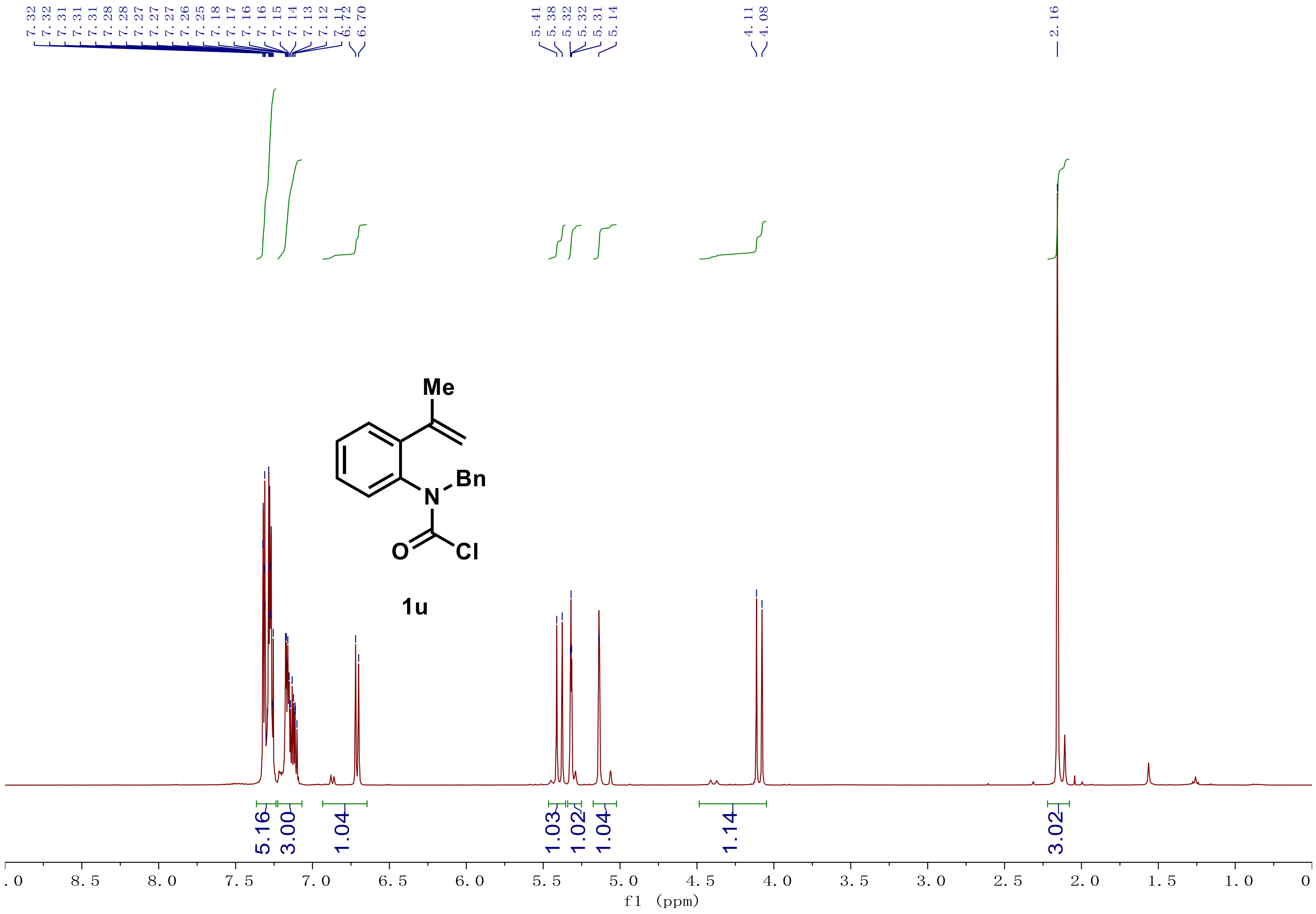

Supplementary Figure 74

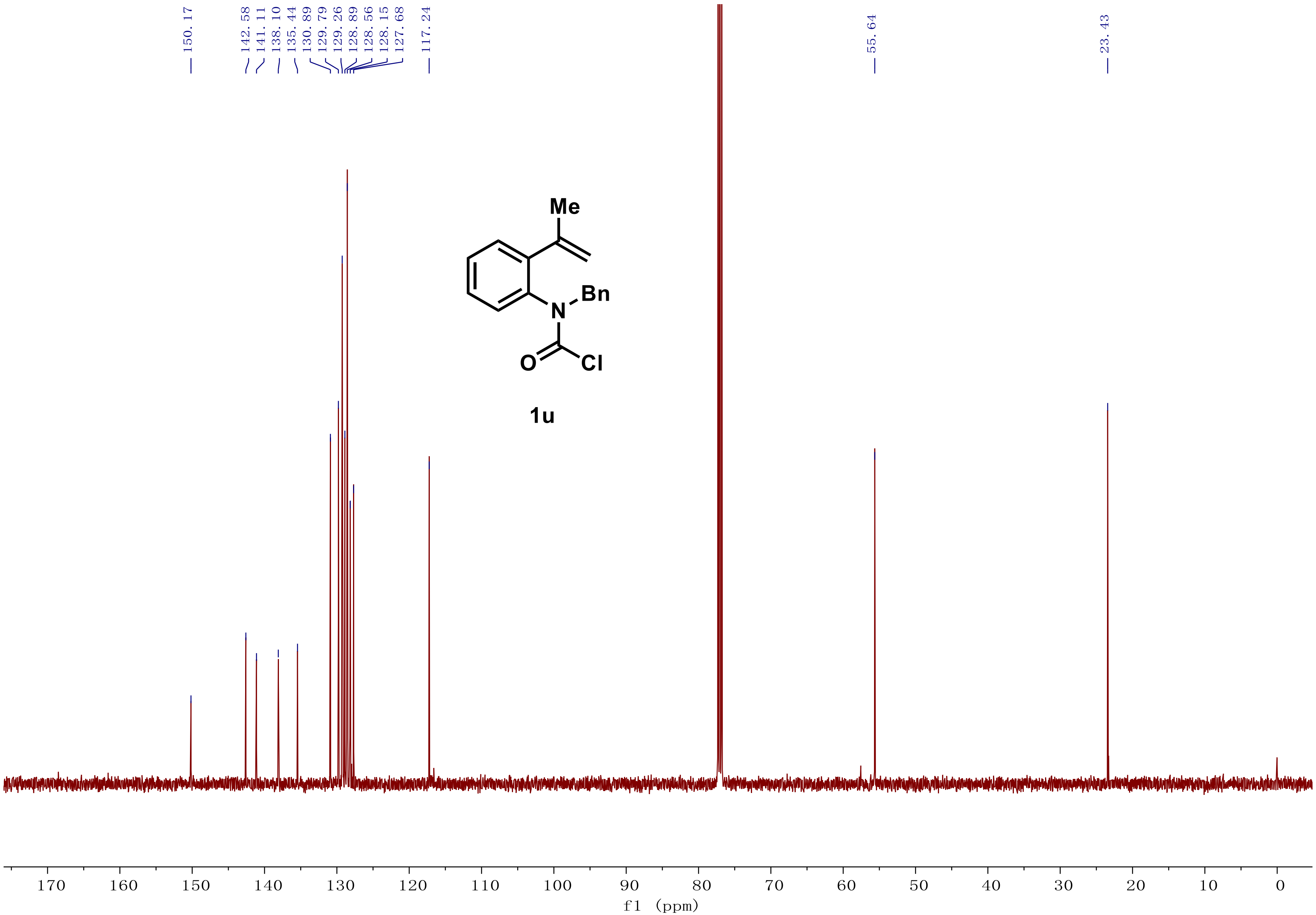

1u

Supplementary Figure 75

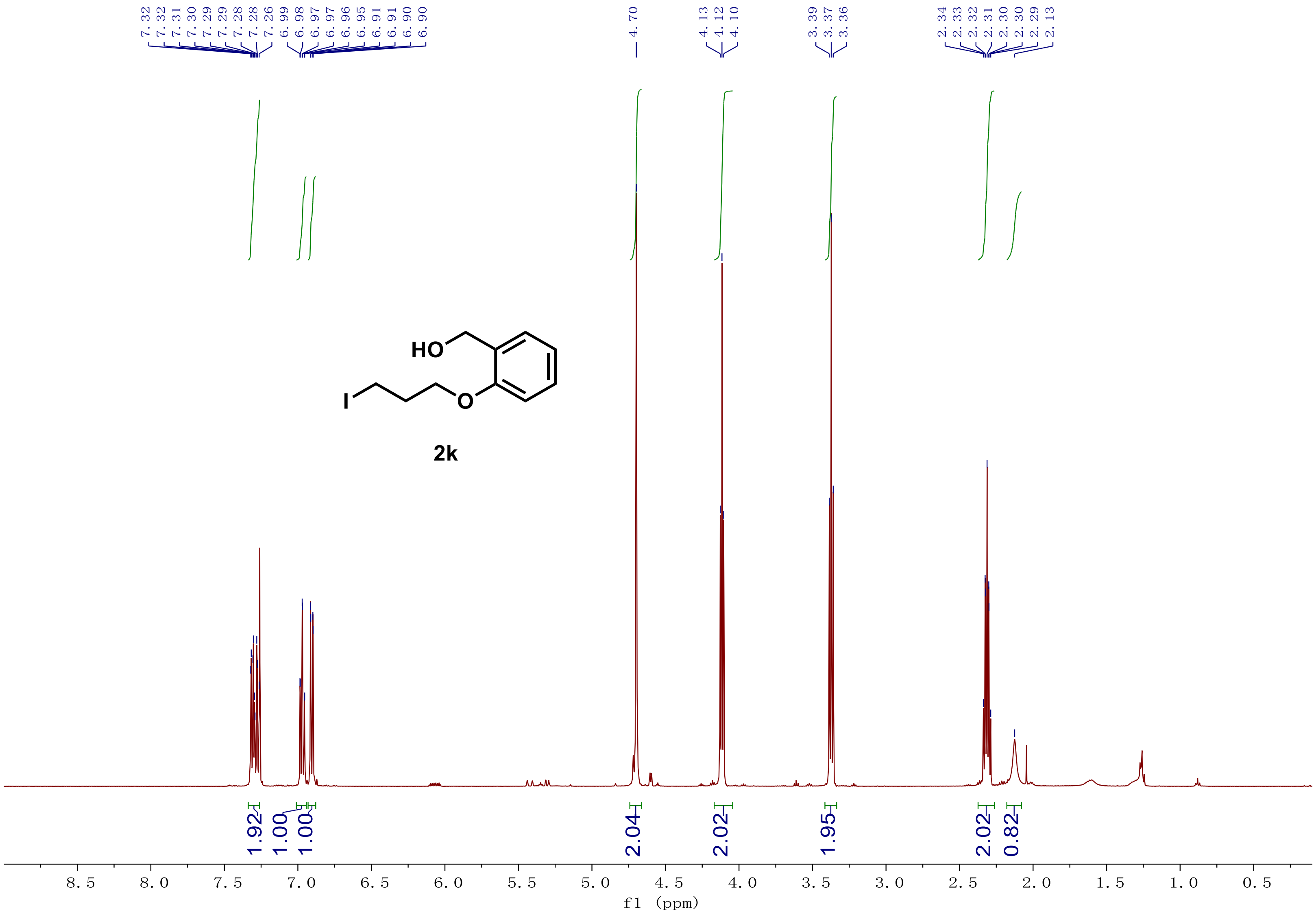

Supplementary Figure 76

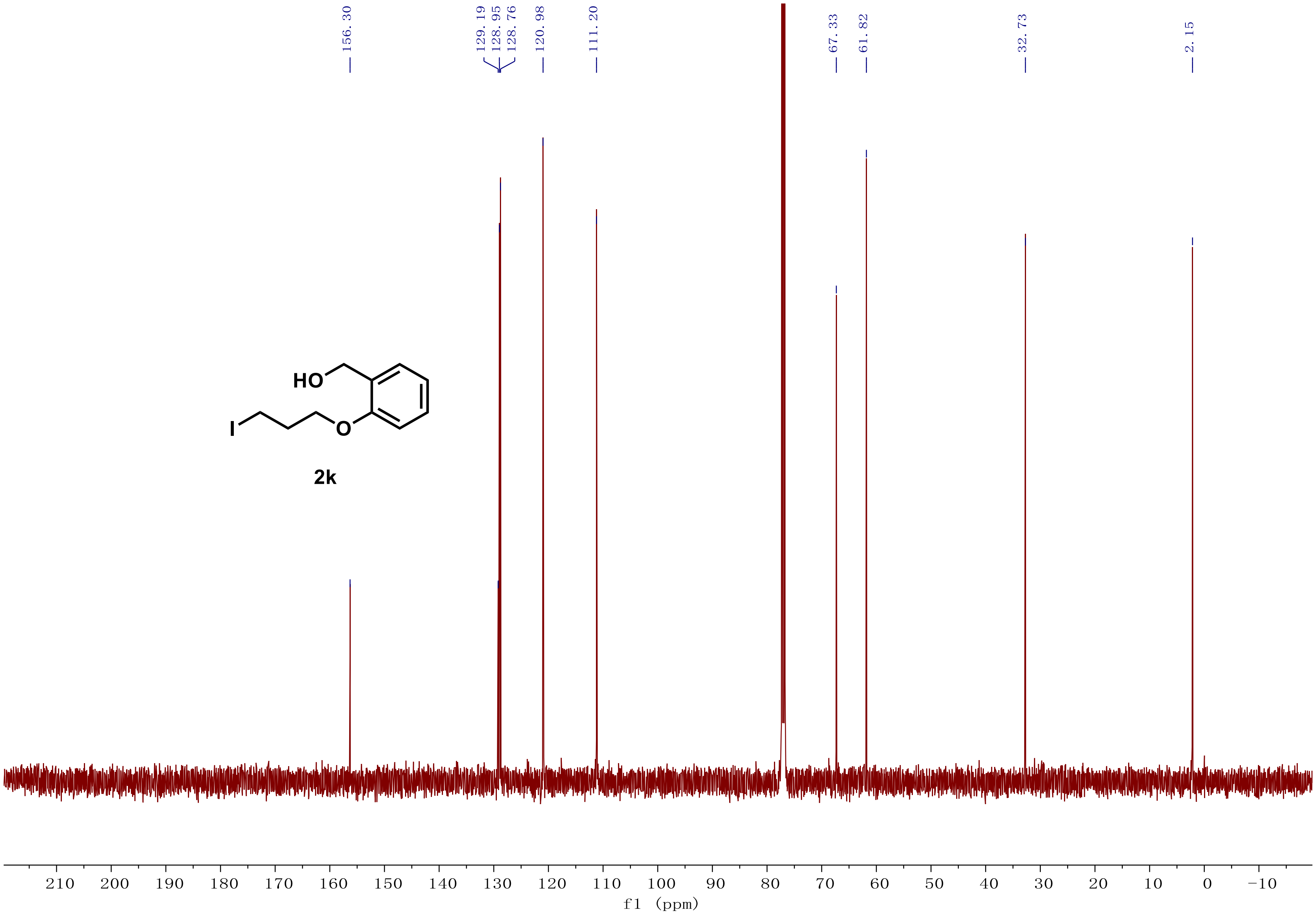

Supplementary Figure 77

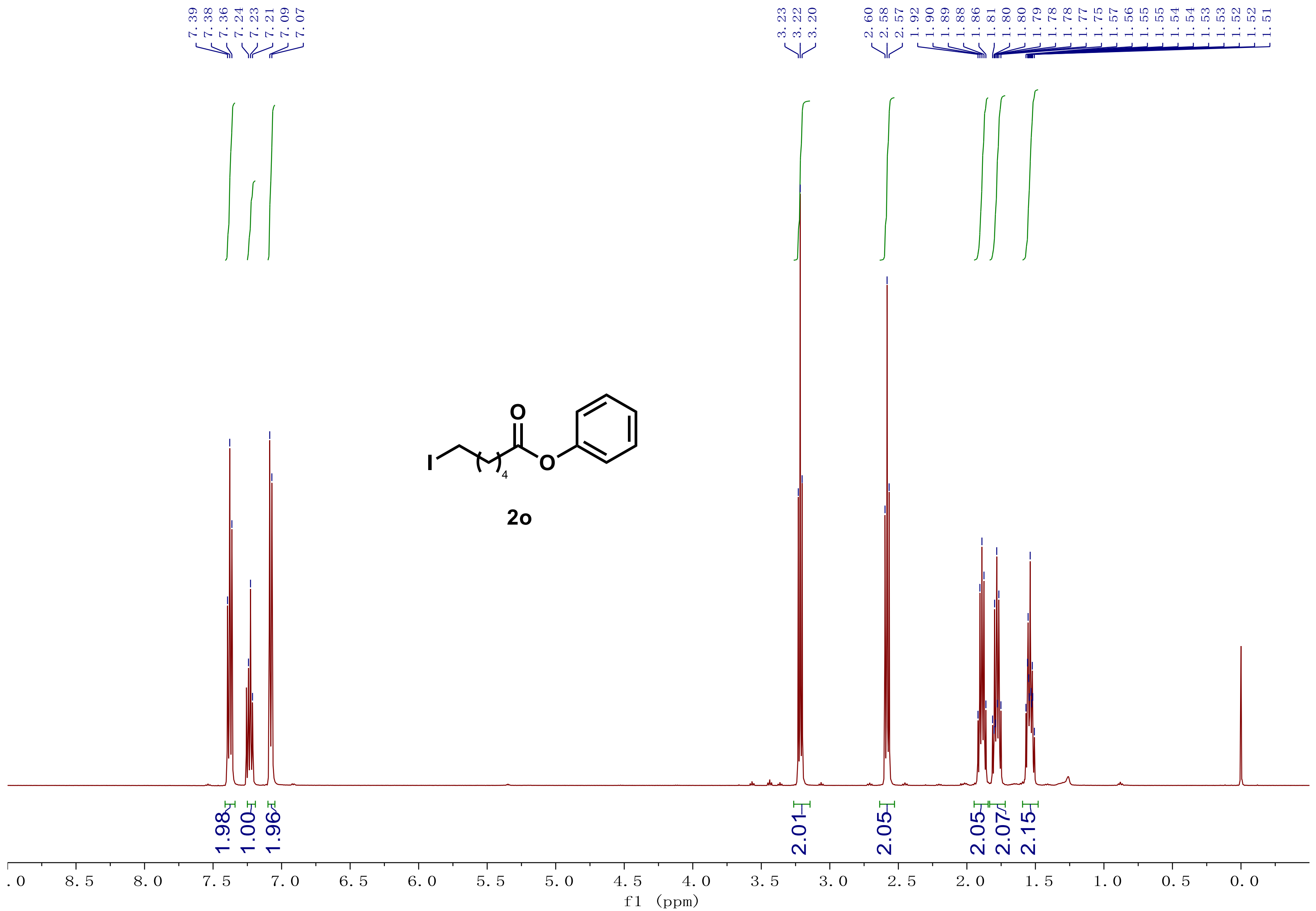

Supplementary Figure 78

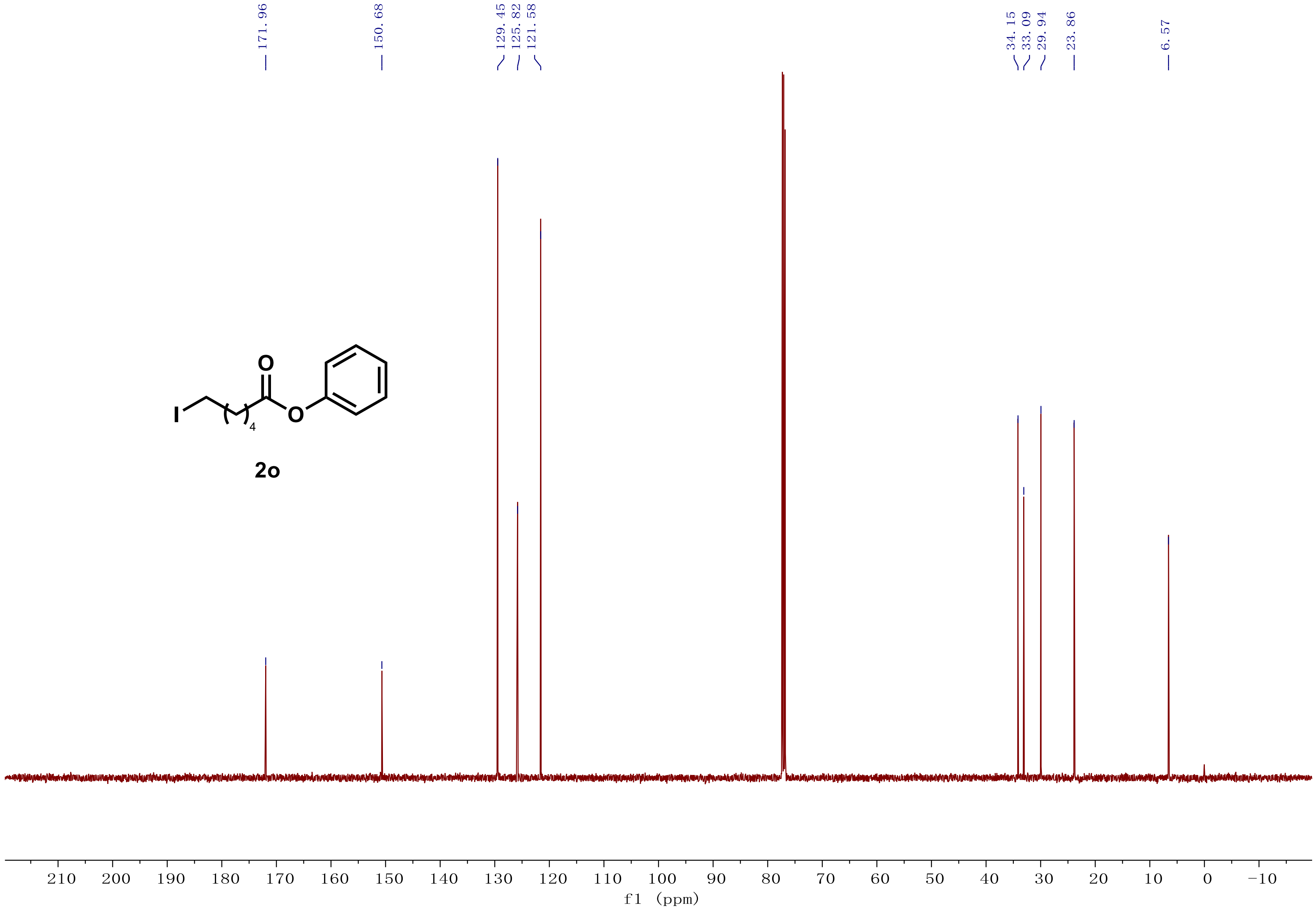

Supplementary Figure 79

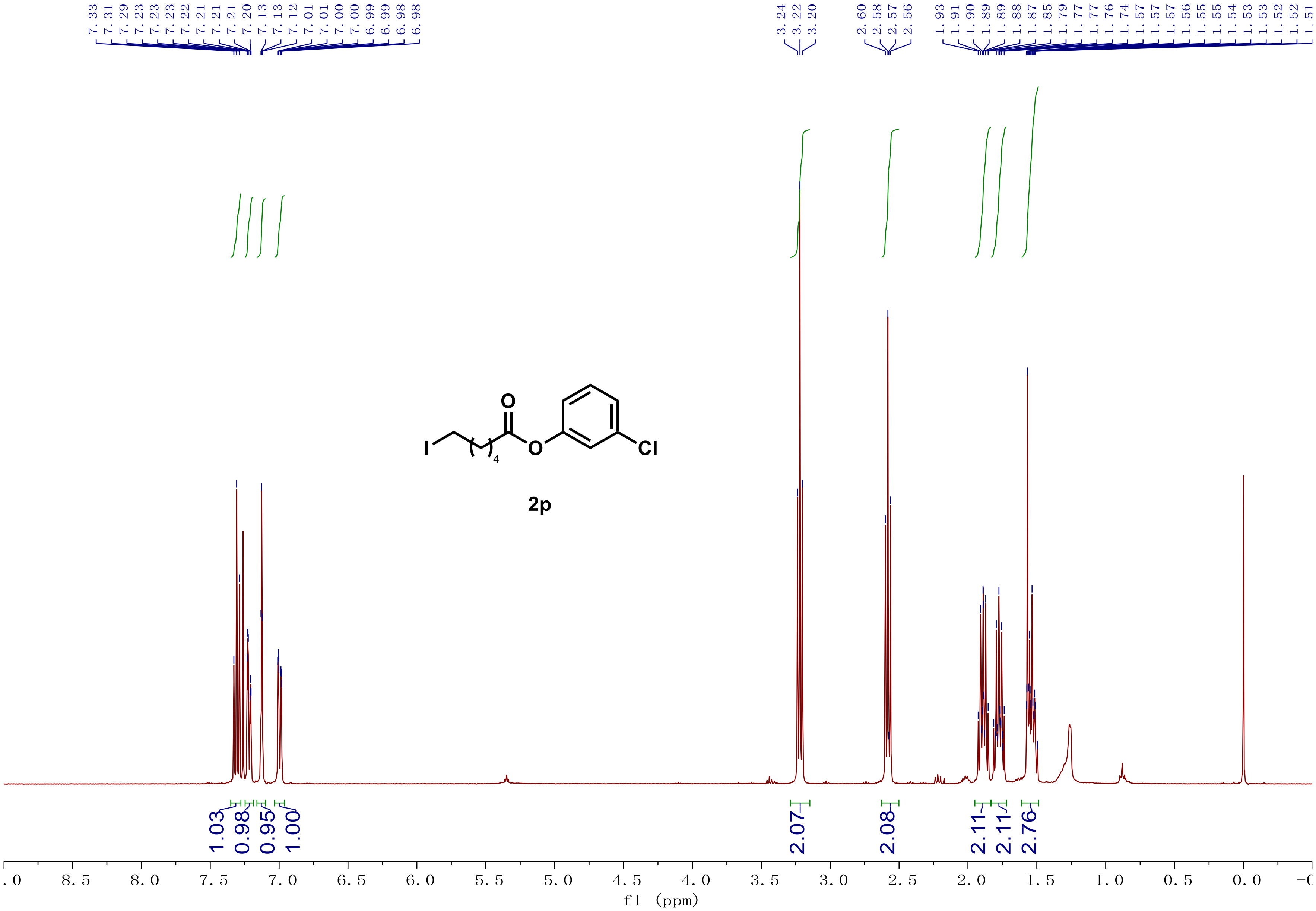

Supplementary Figure 80

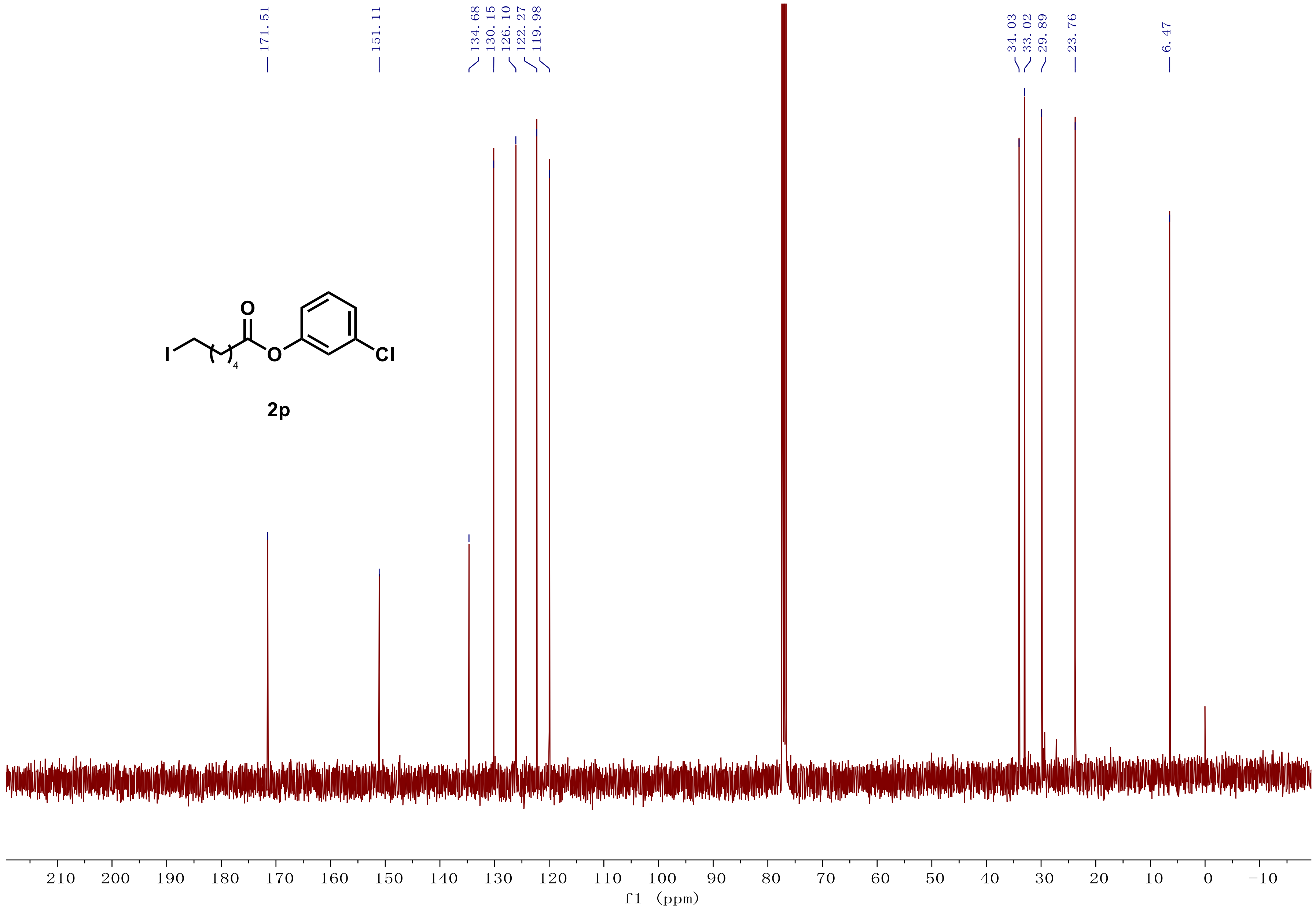

Supplementary Figure 81

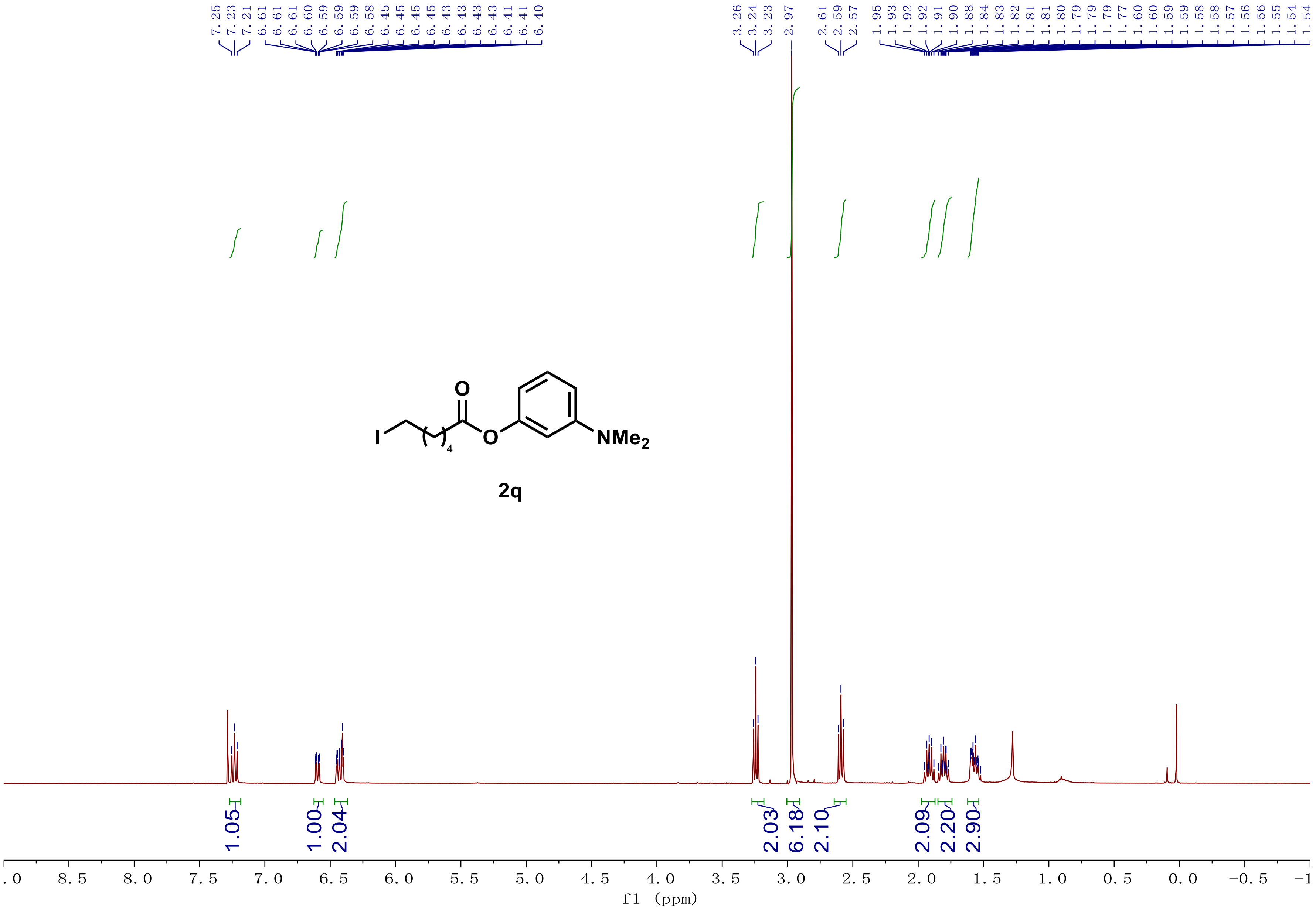

2q

Supplementary Figure 82

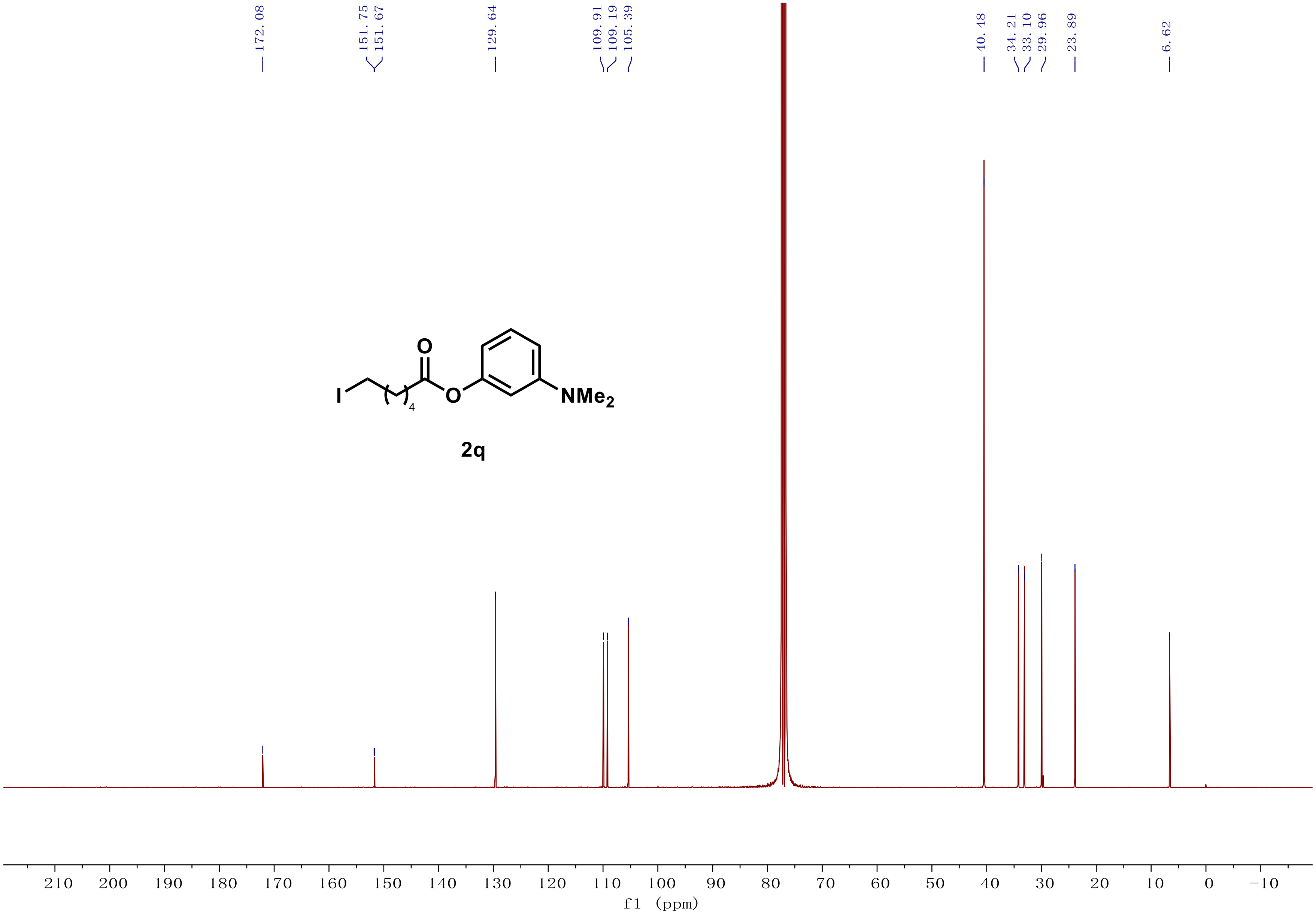

Supplementary Figure 83

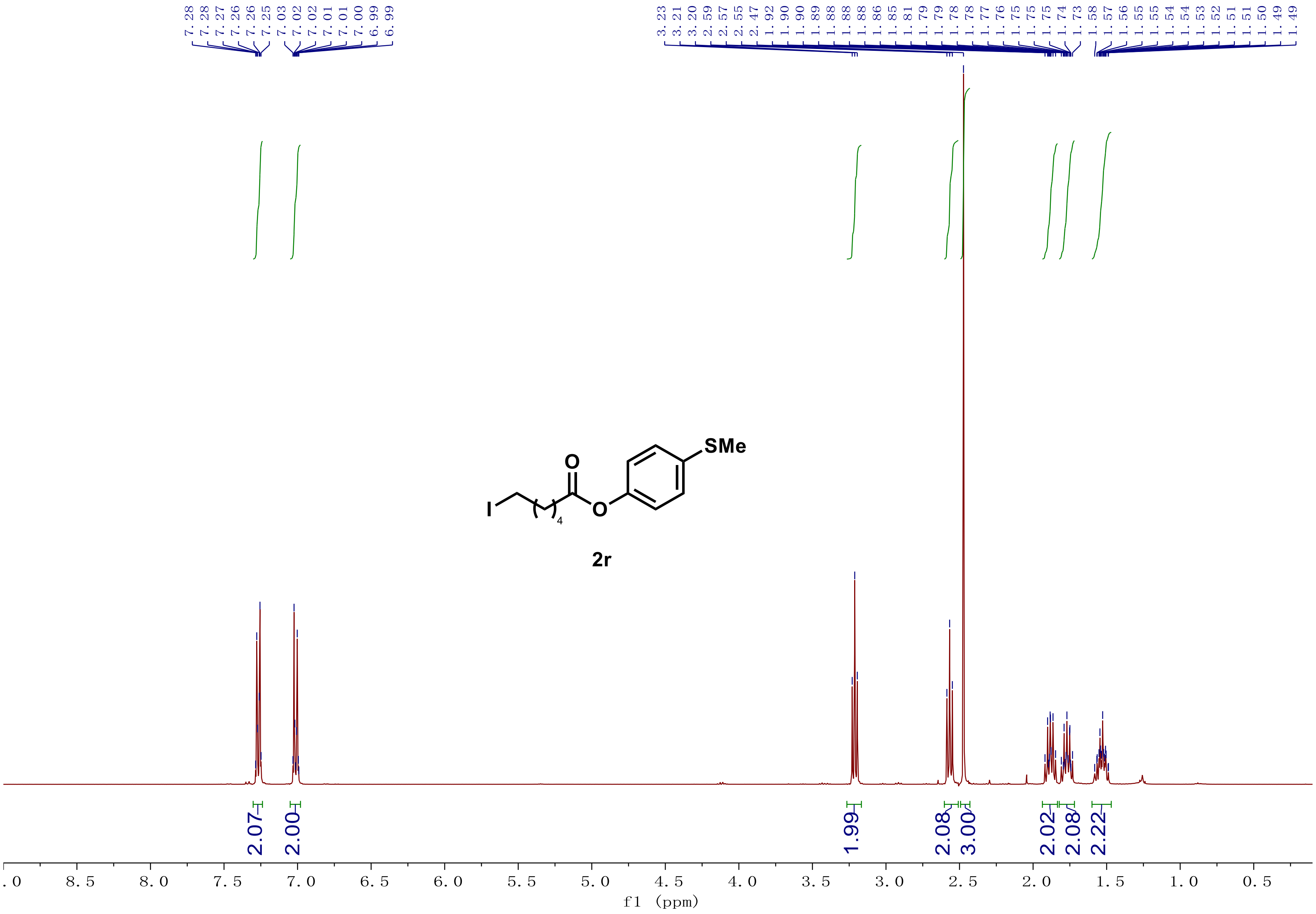

Supplementary Figure 84

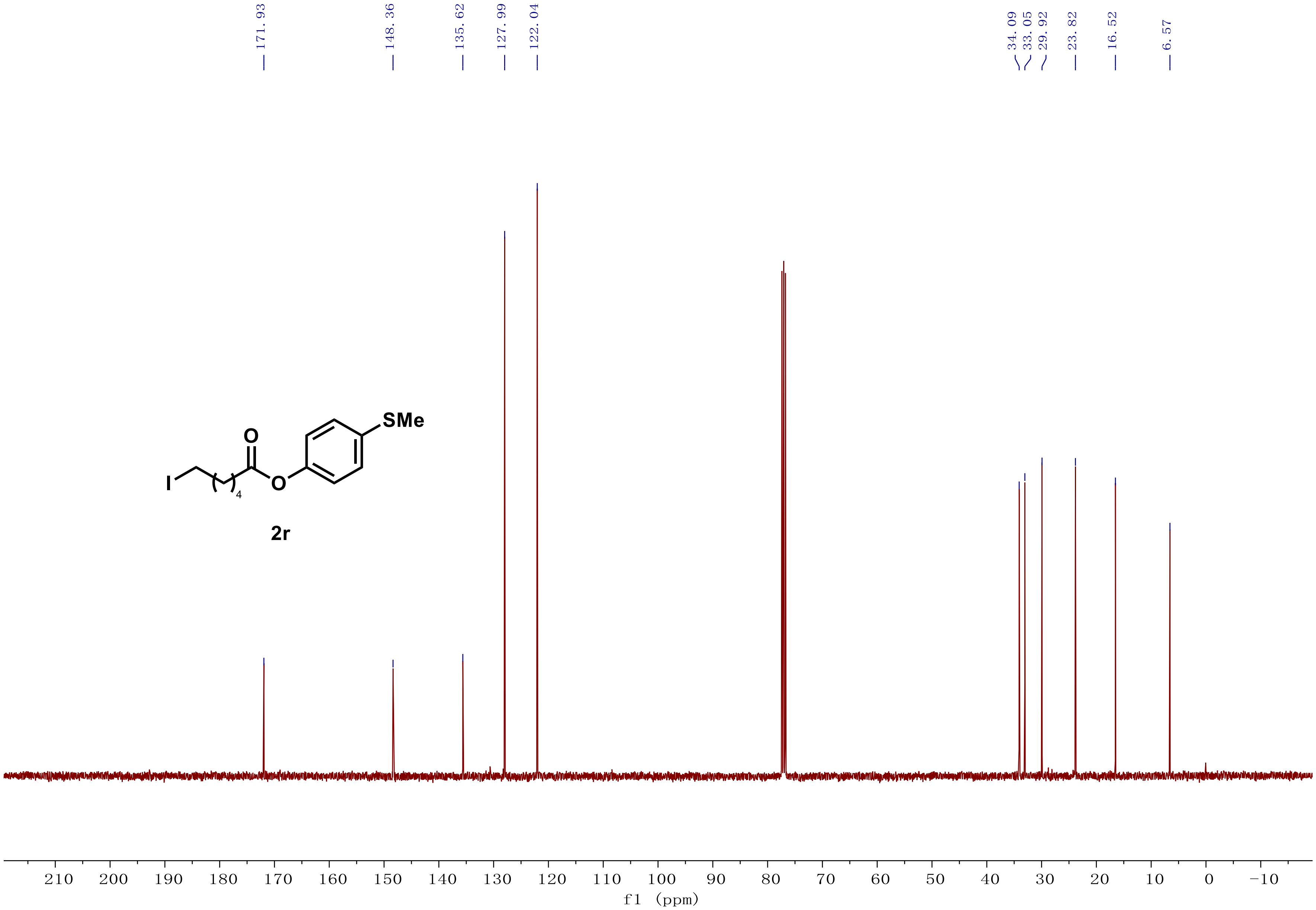

Supplementary Figure 85

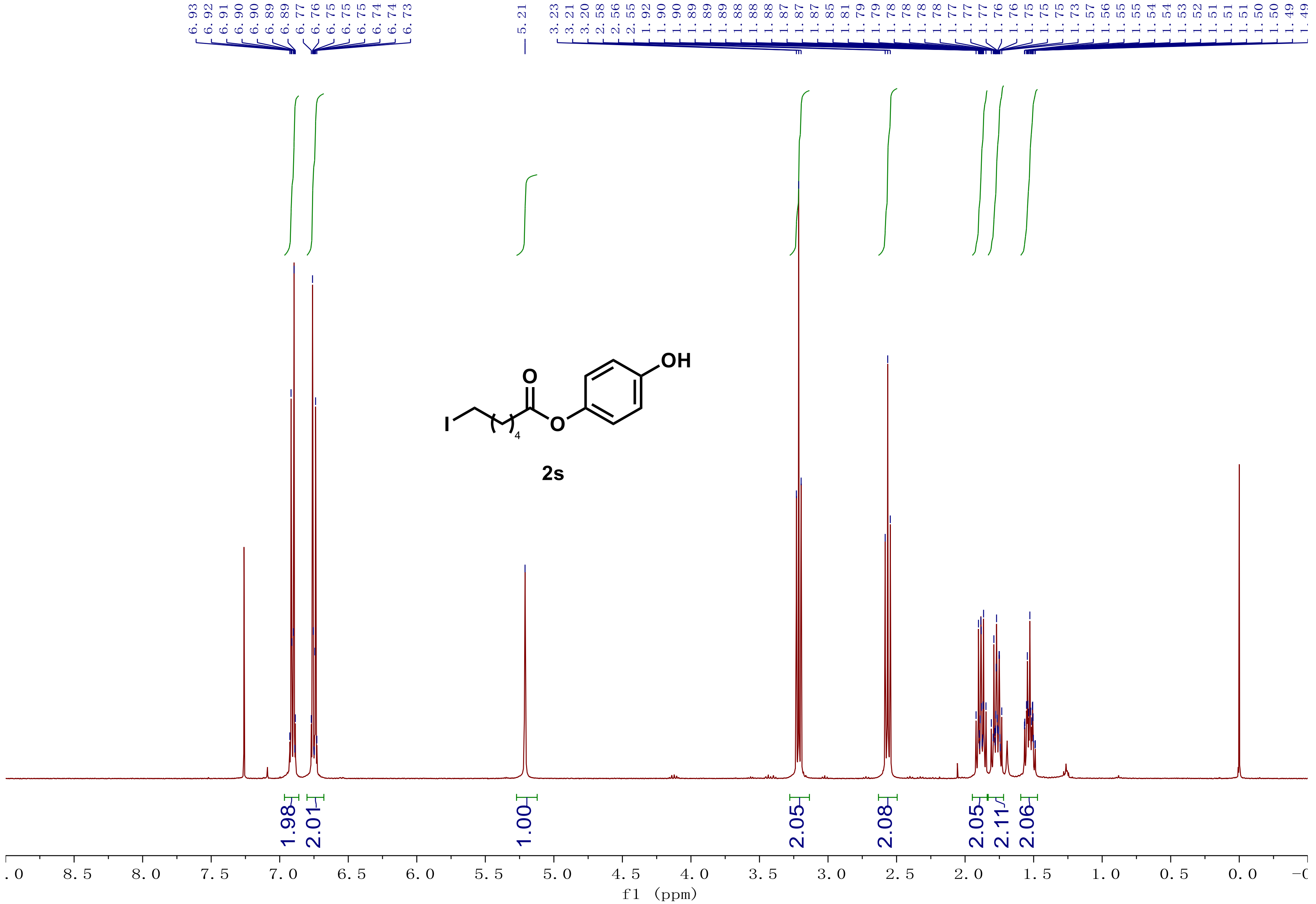

Supplementary Figure 86

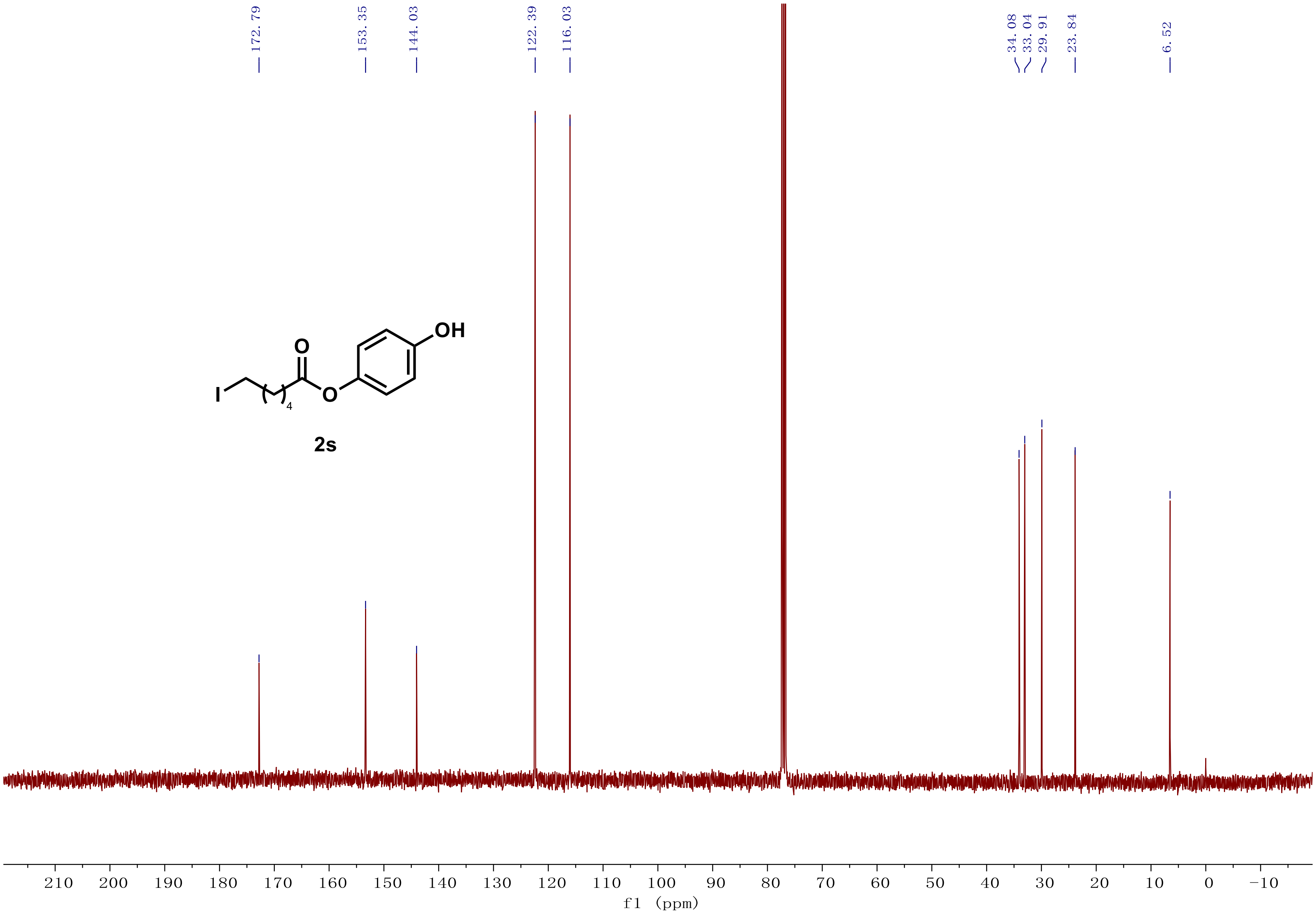

Supplementary Figure 87

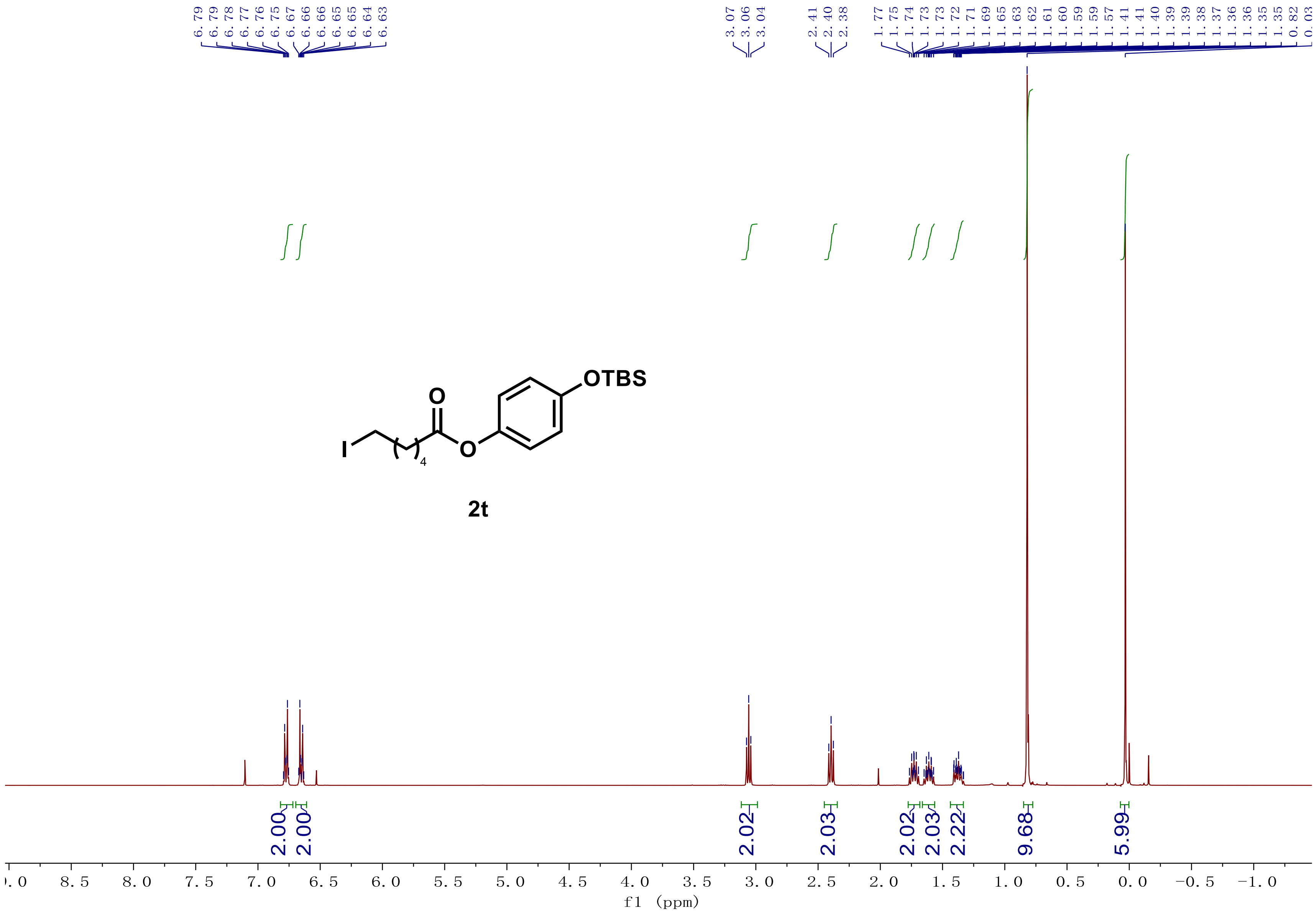

Supplementary Figure 88

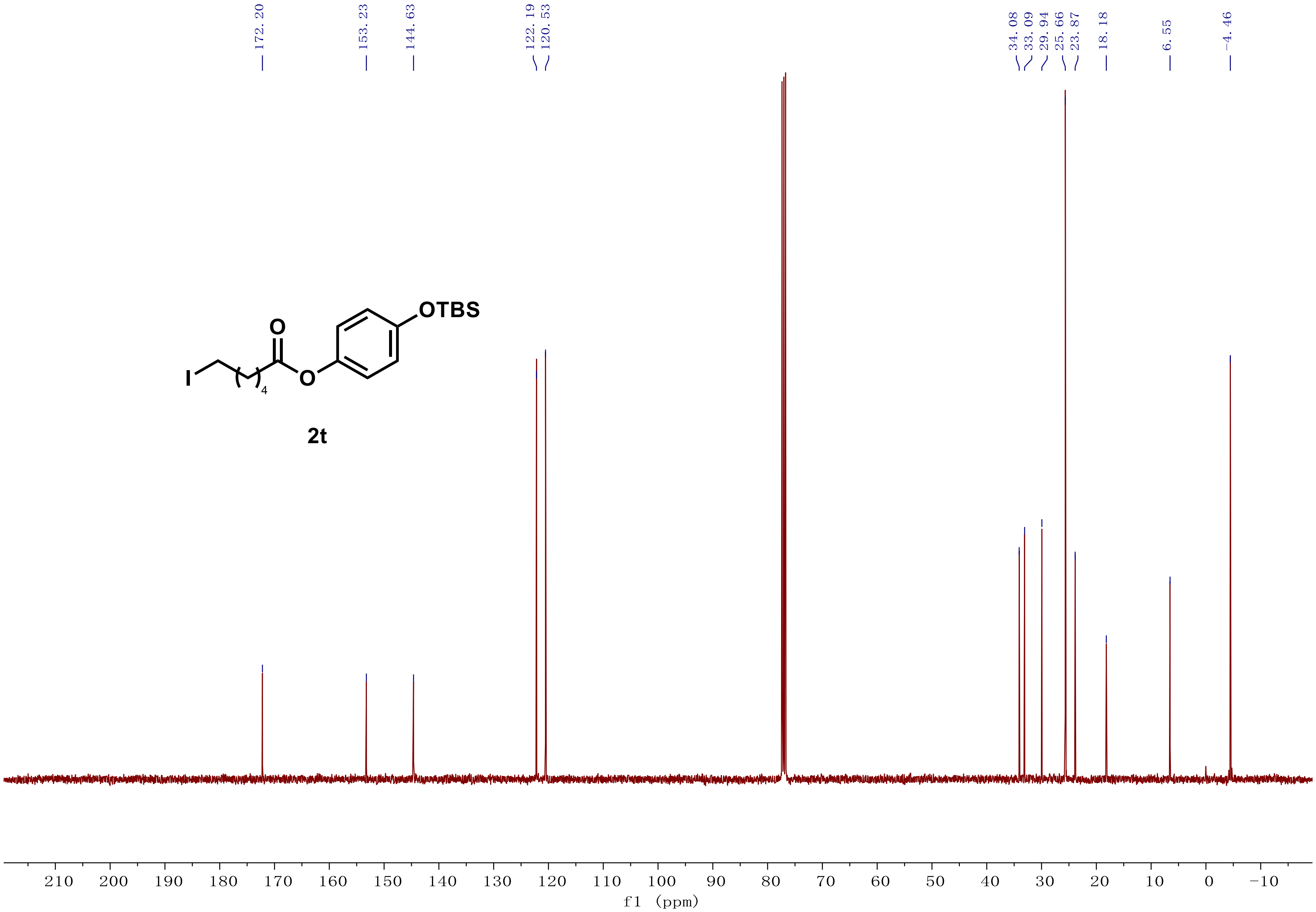

Supplementary Figure 89

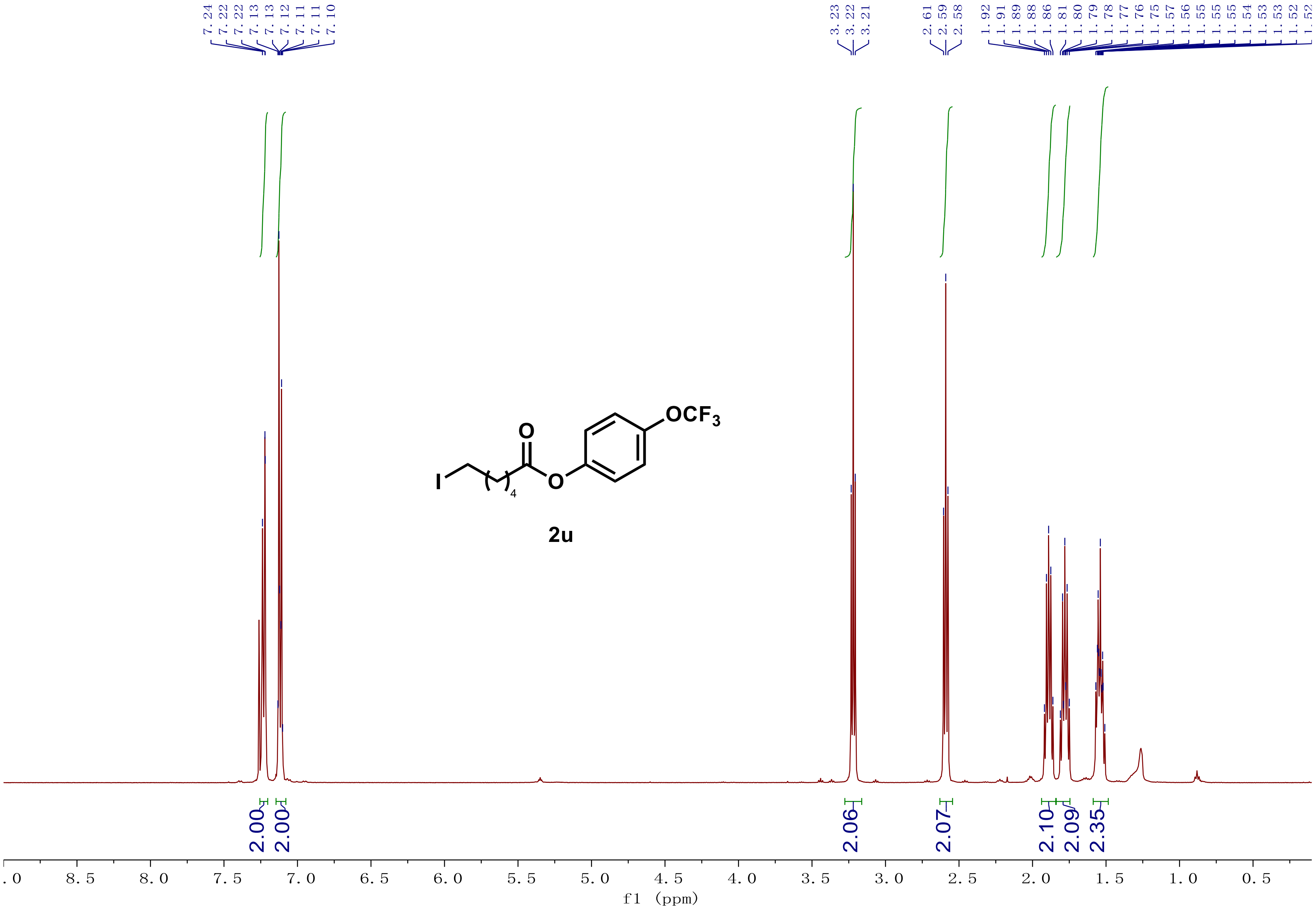

Supplementary Figure 90

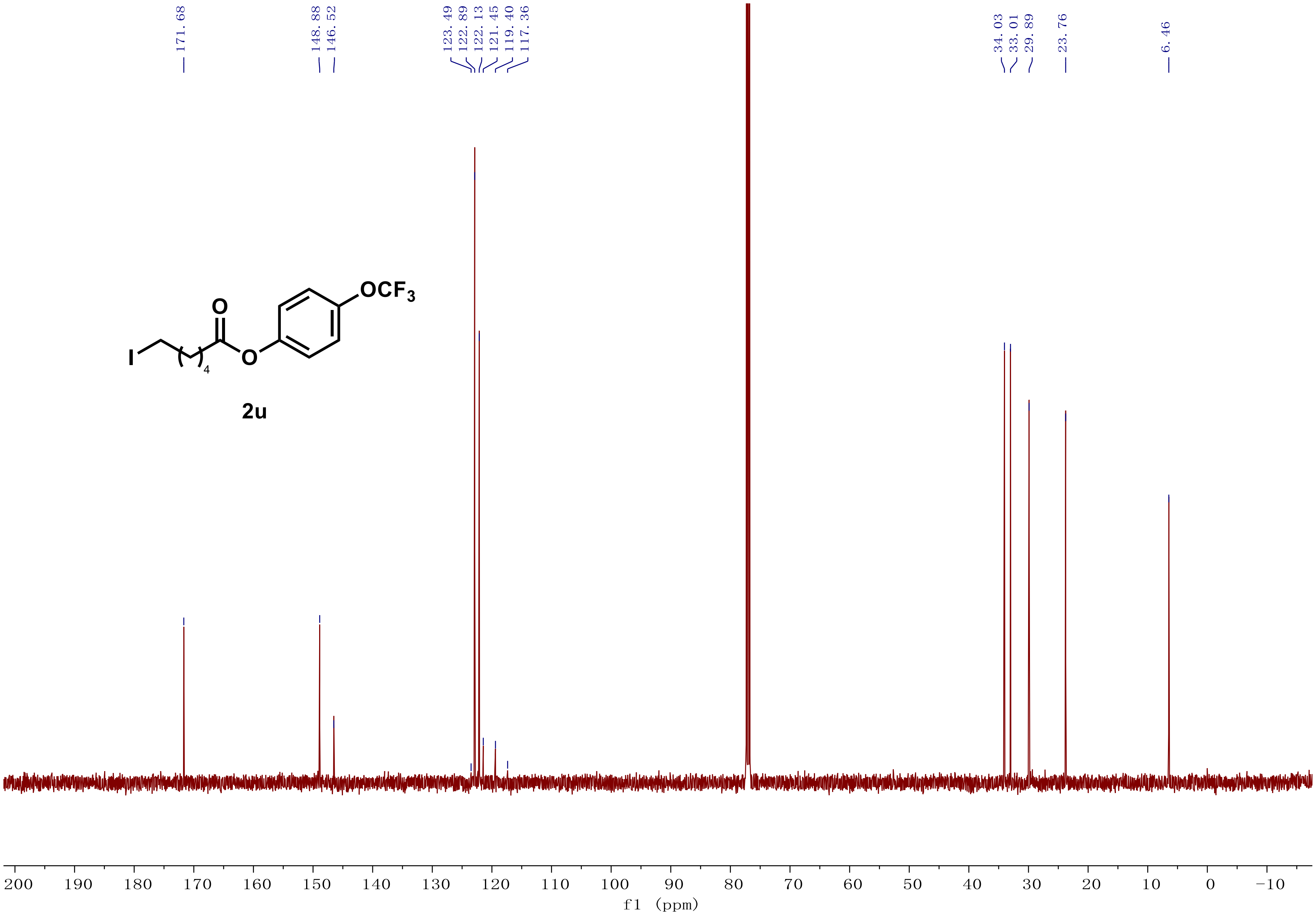

Supplementary Figure 91

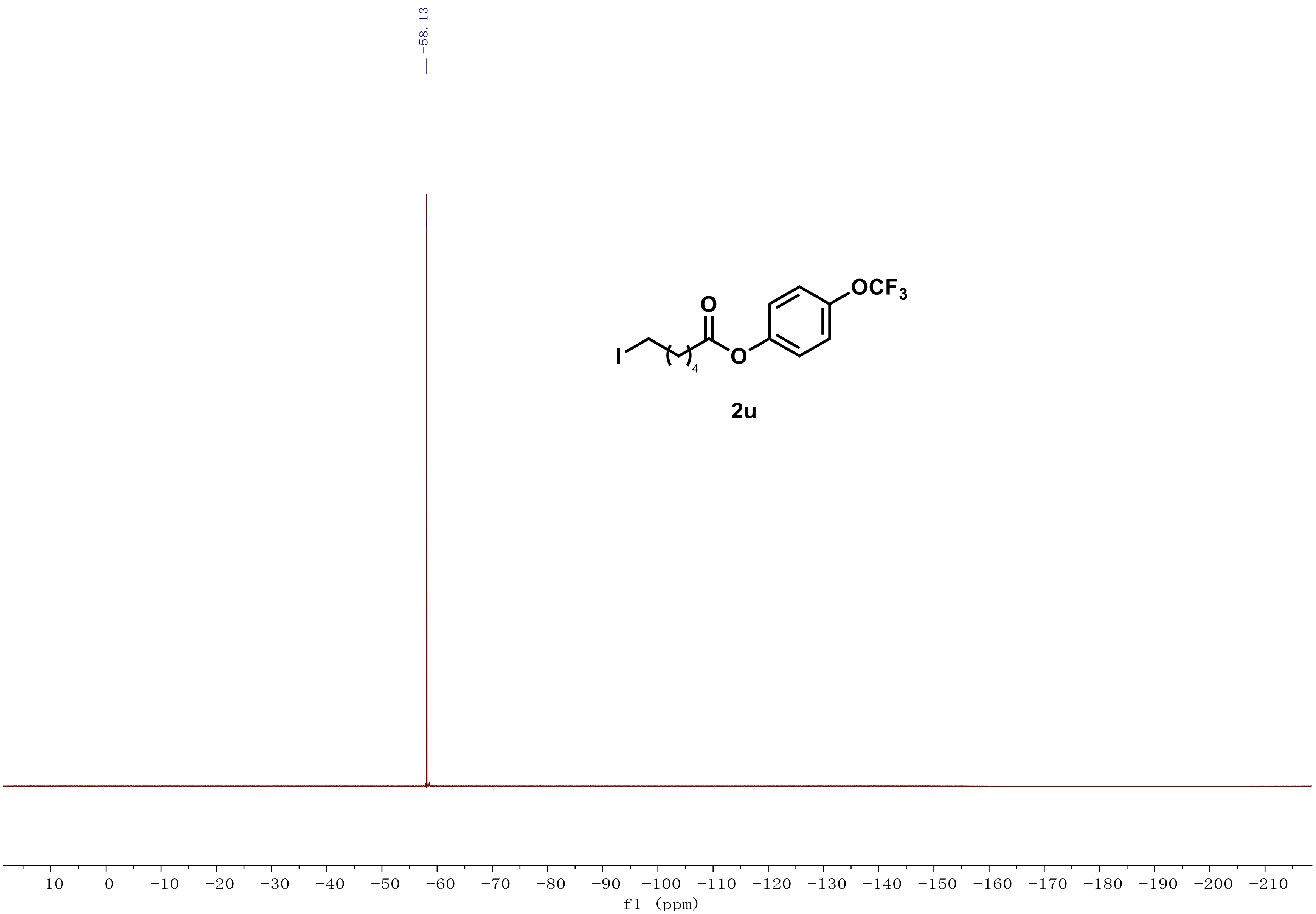

Supplementary Figure 92

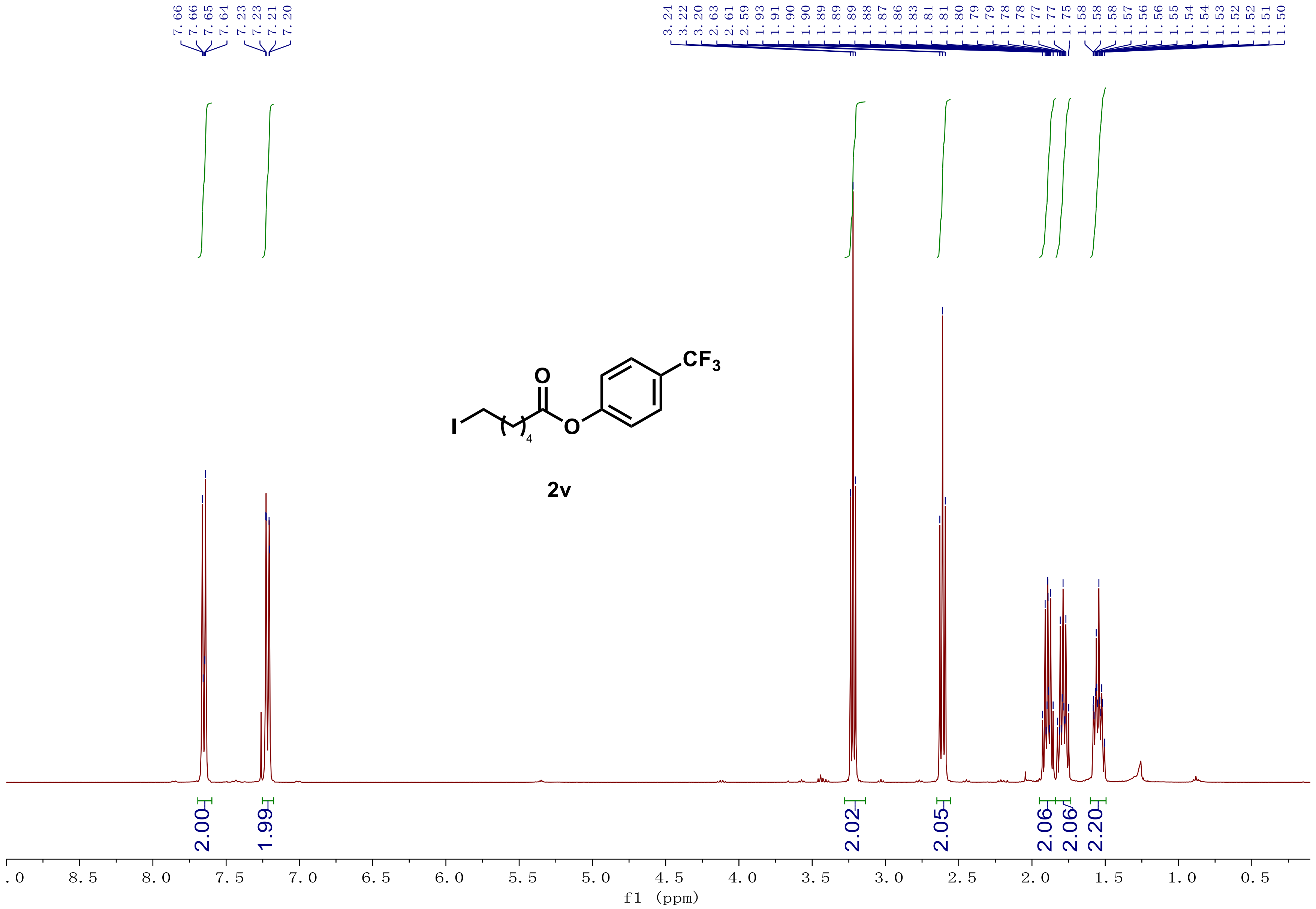

Supplementary Figure 93

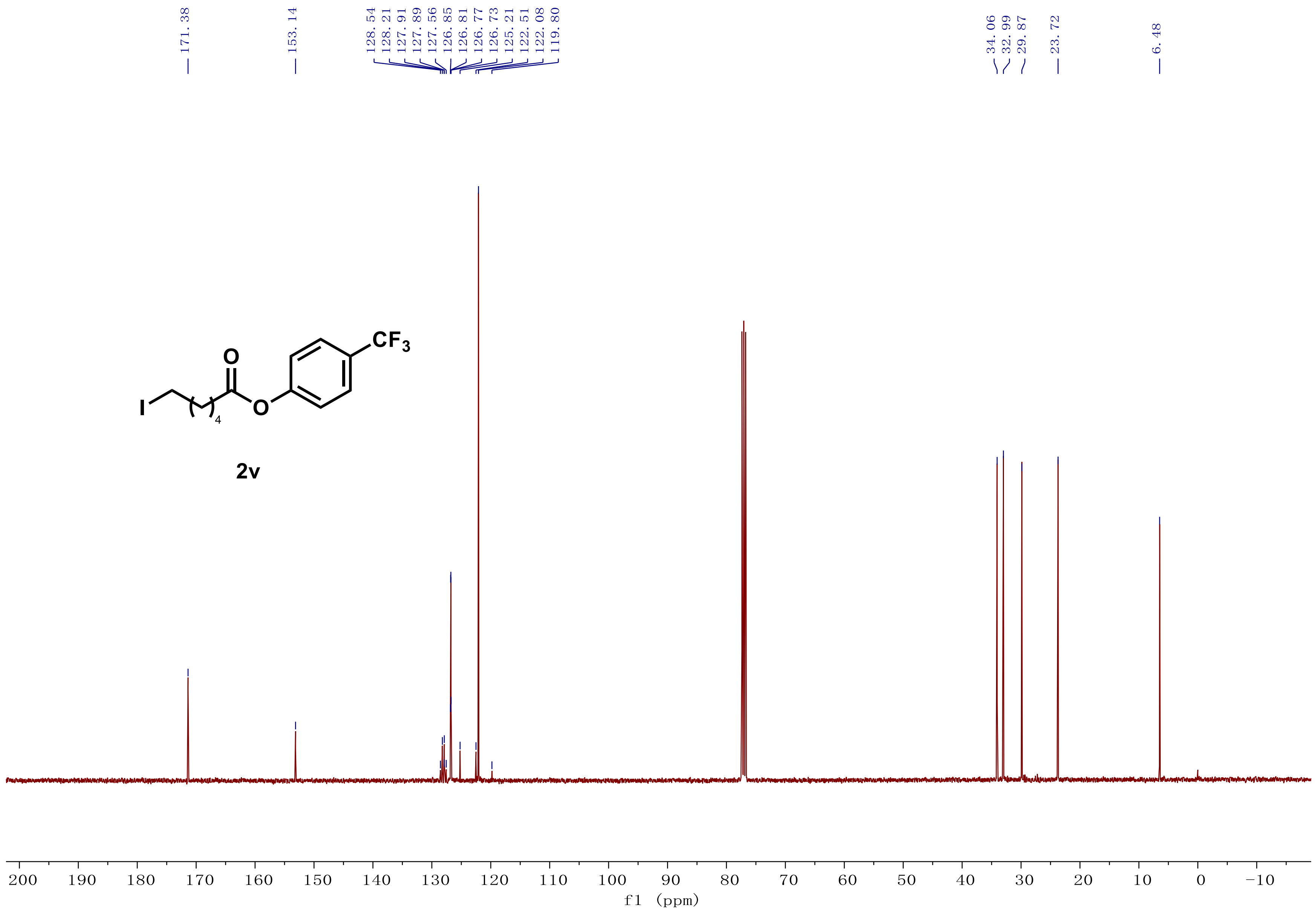

Supplementary Figure 94

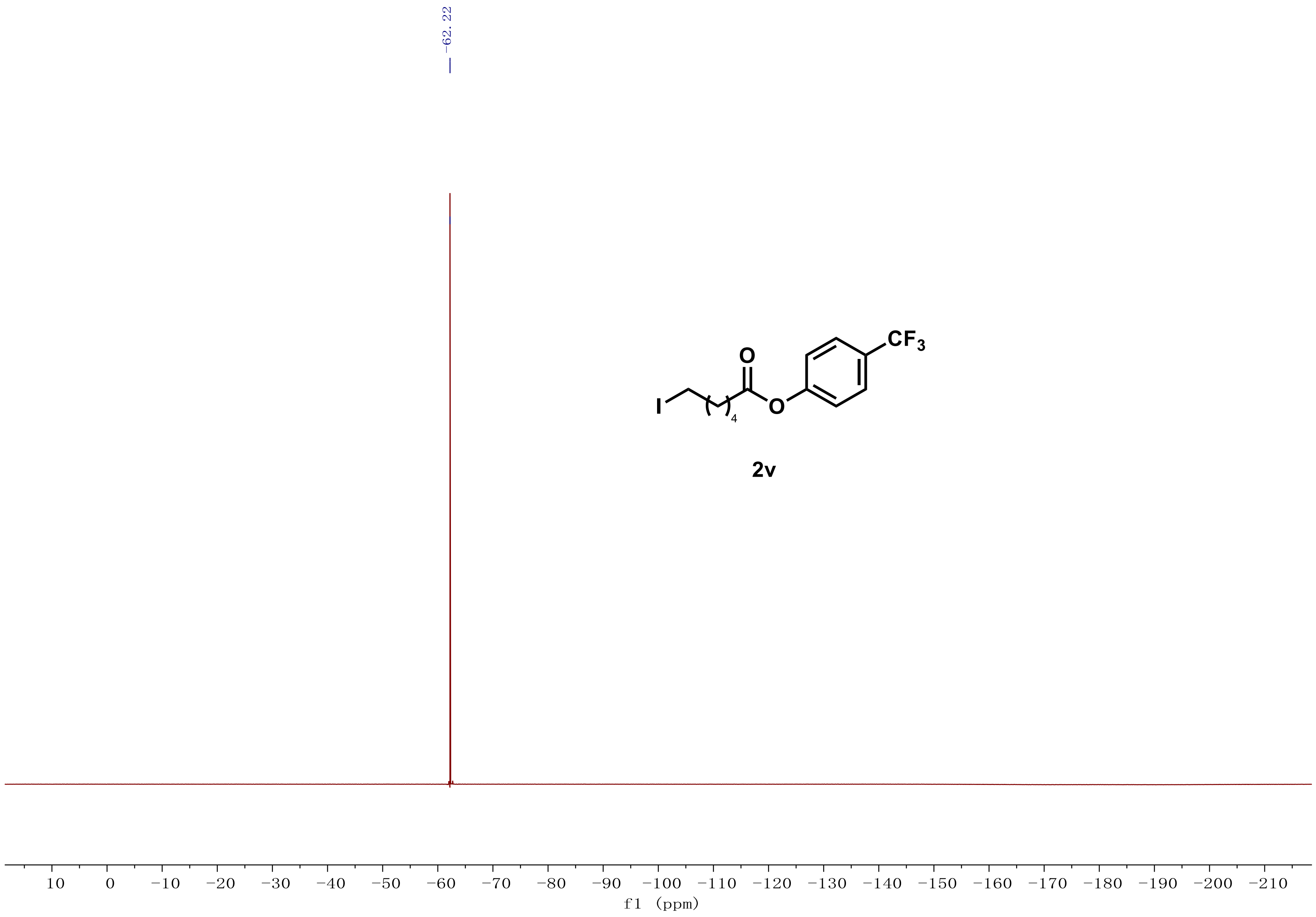

Supplementary Figure 95

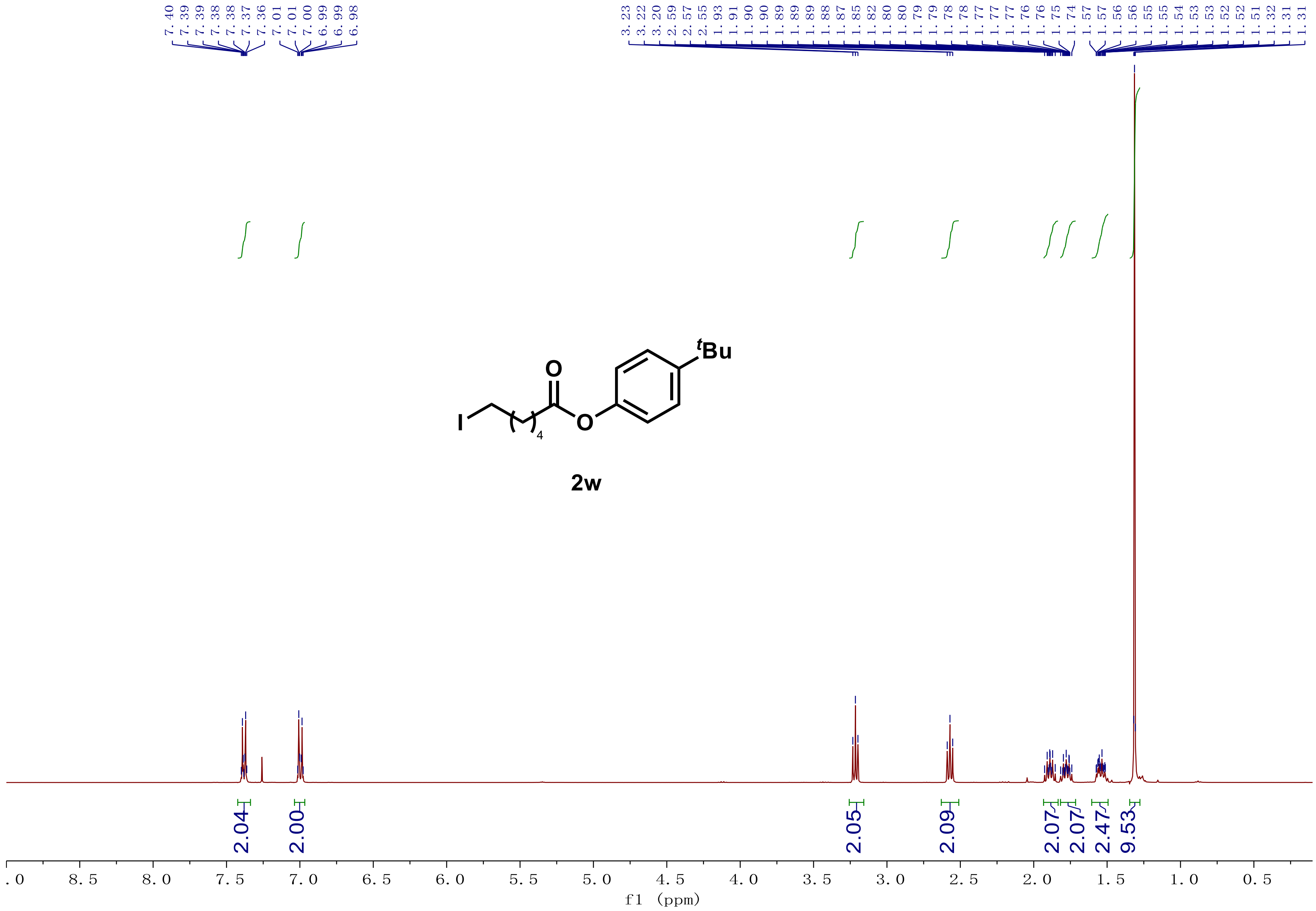

Supplementary Figure 96

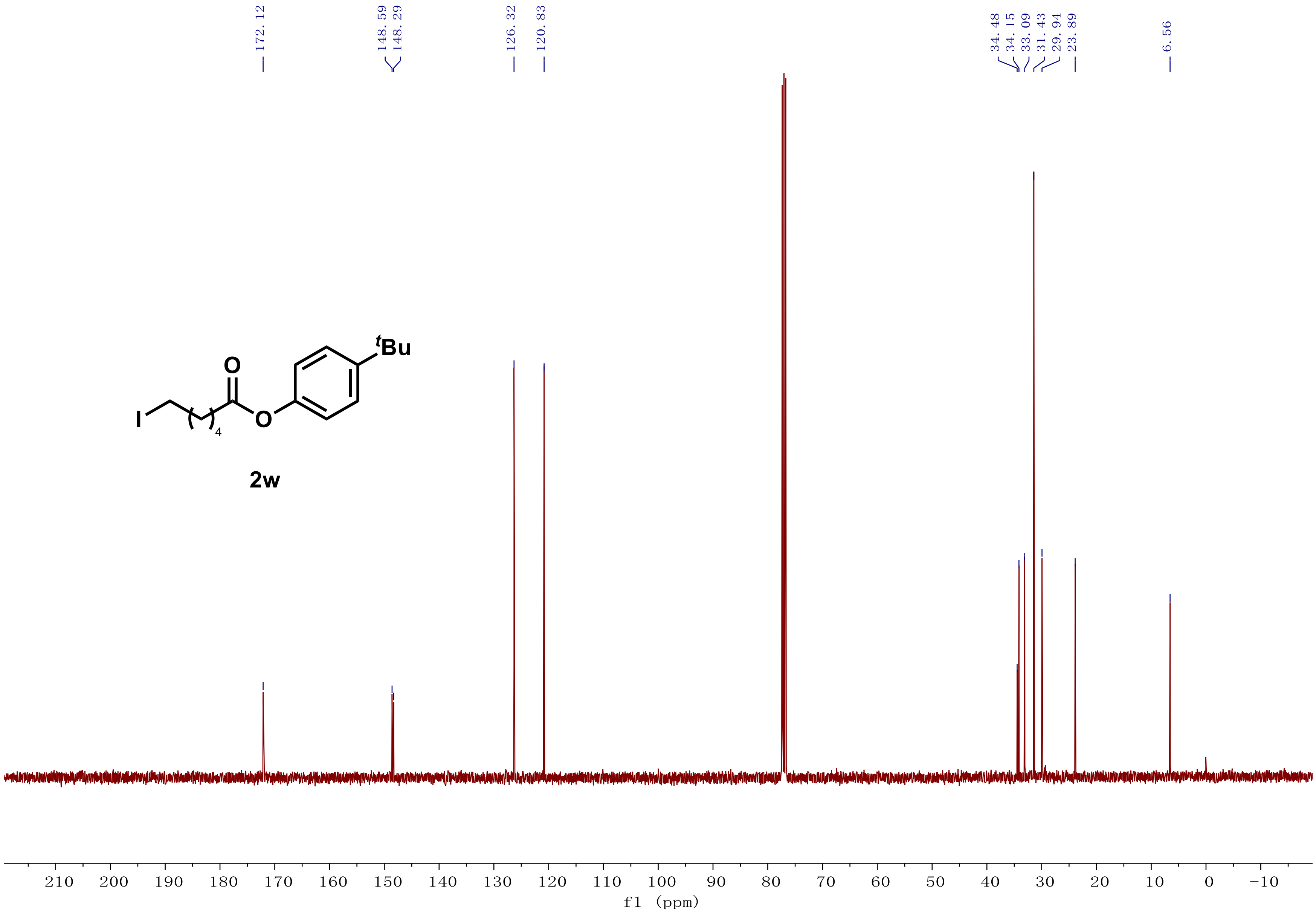

Supplementary Figure 97

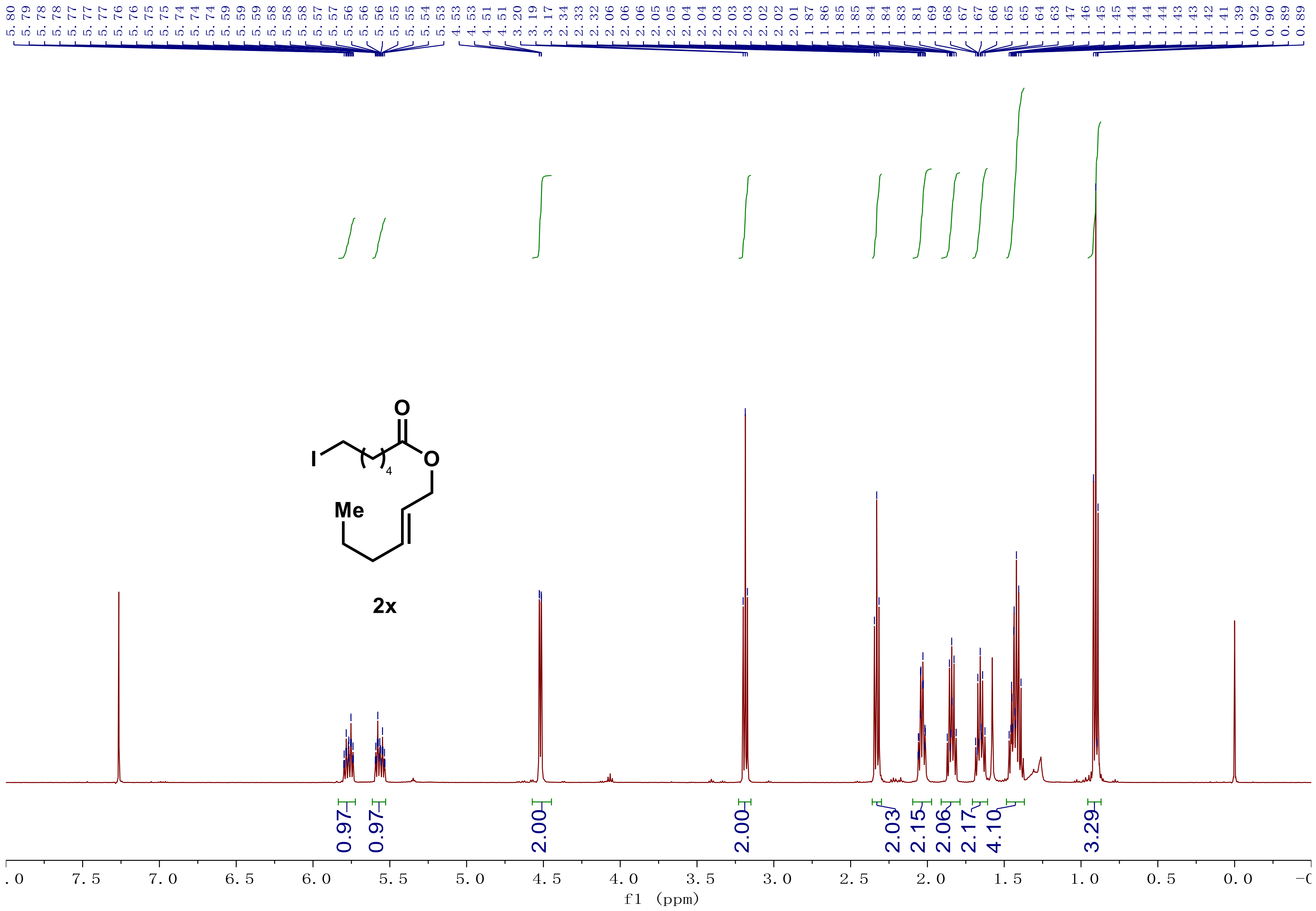

Supplementary Figure 98

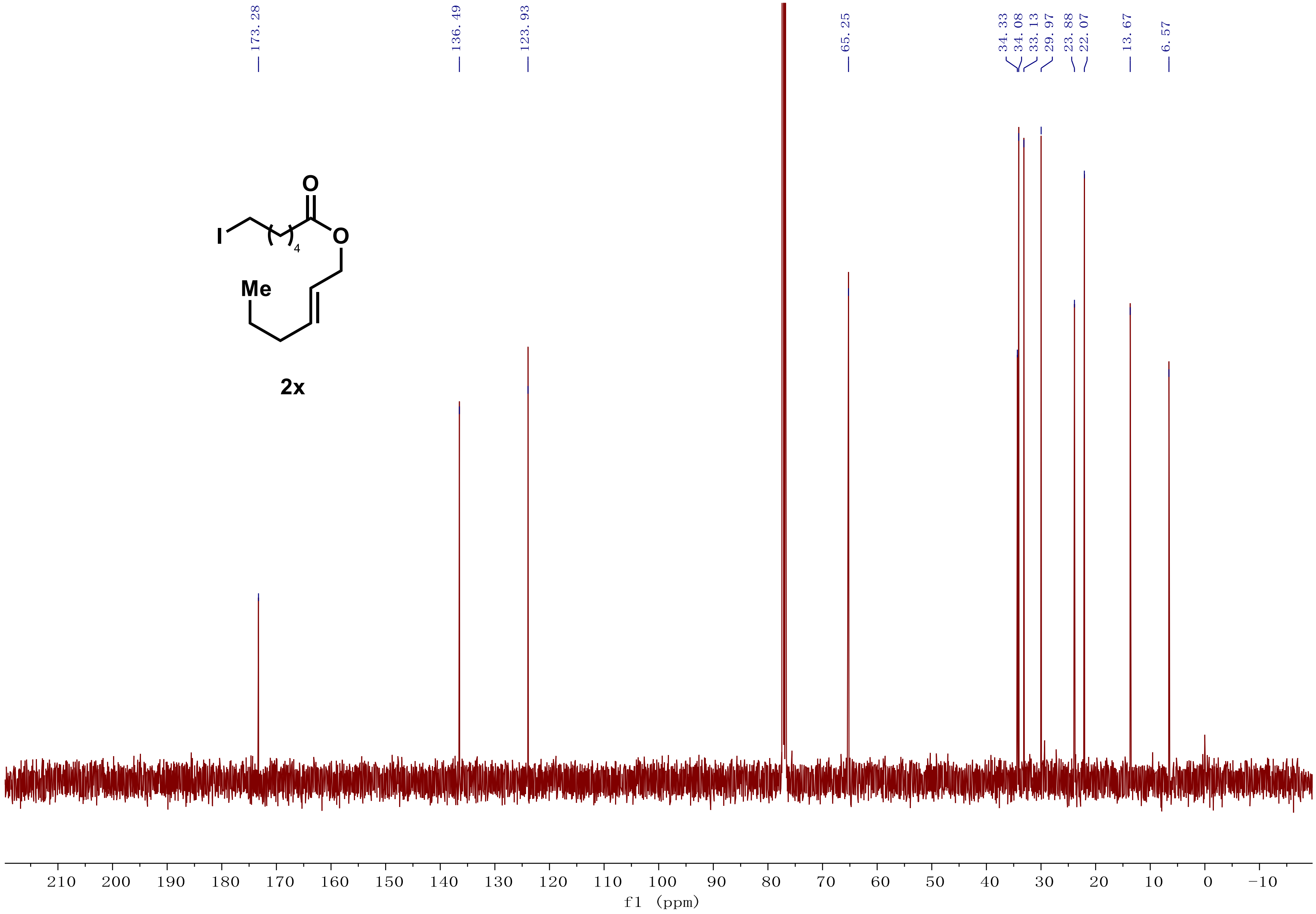

Supplementary Figure 99

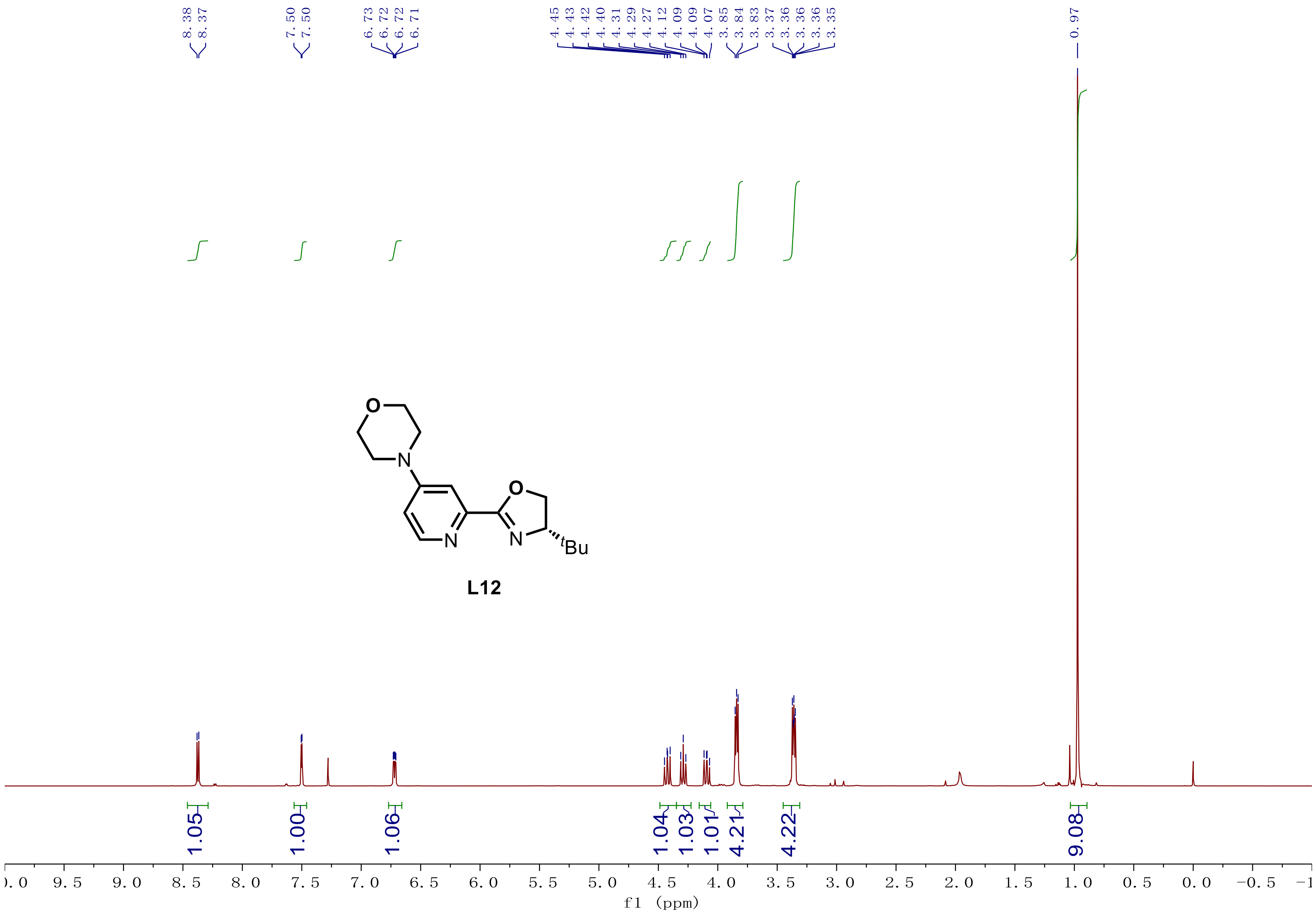

Supplementary Figure 100

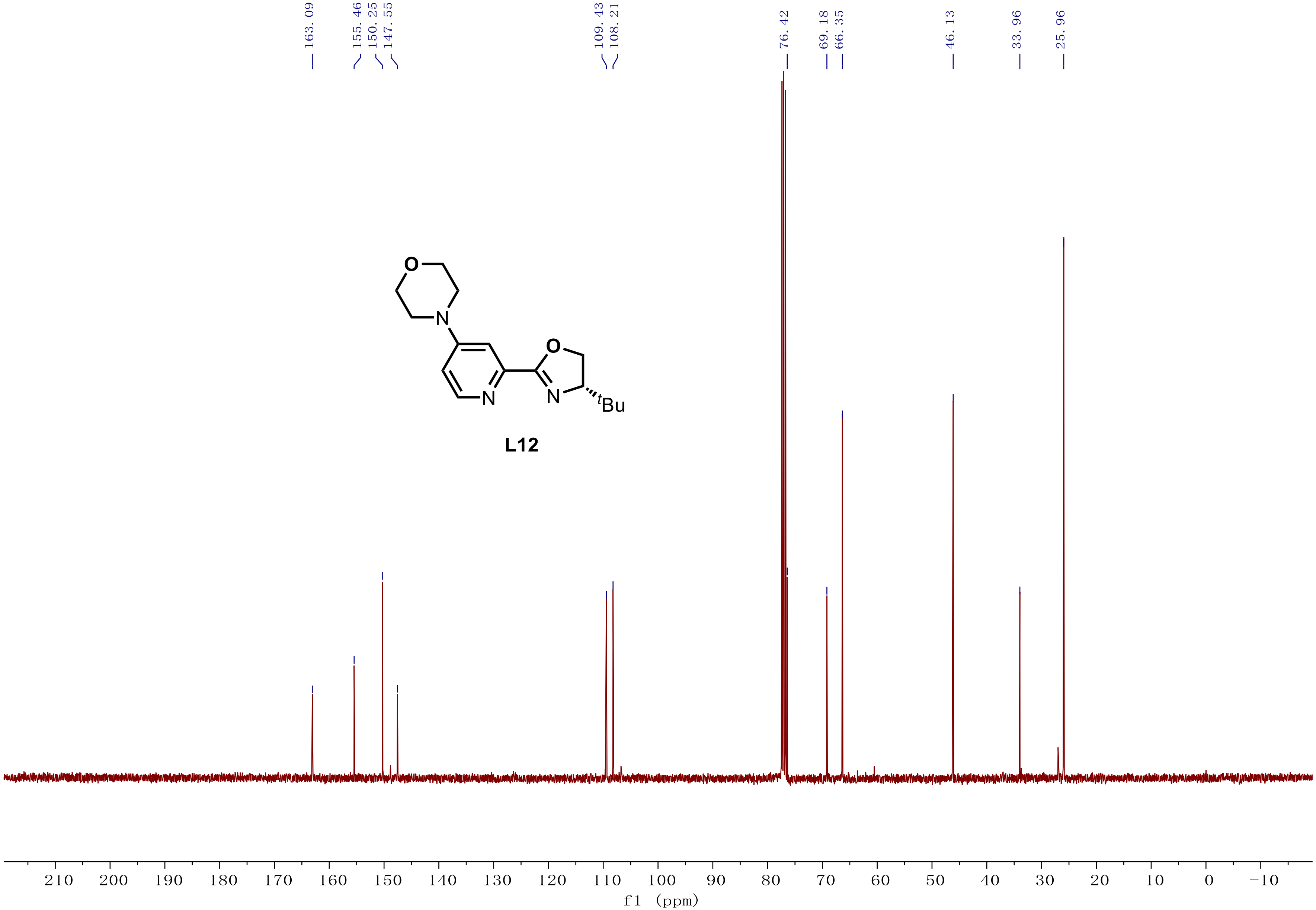

Supplementary Figure 101

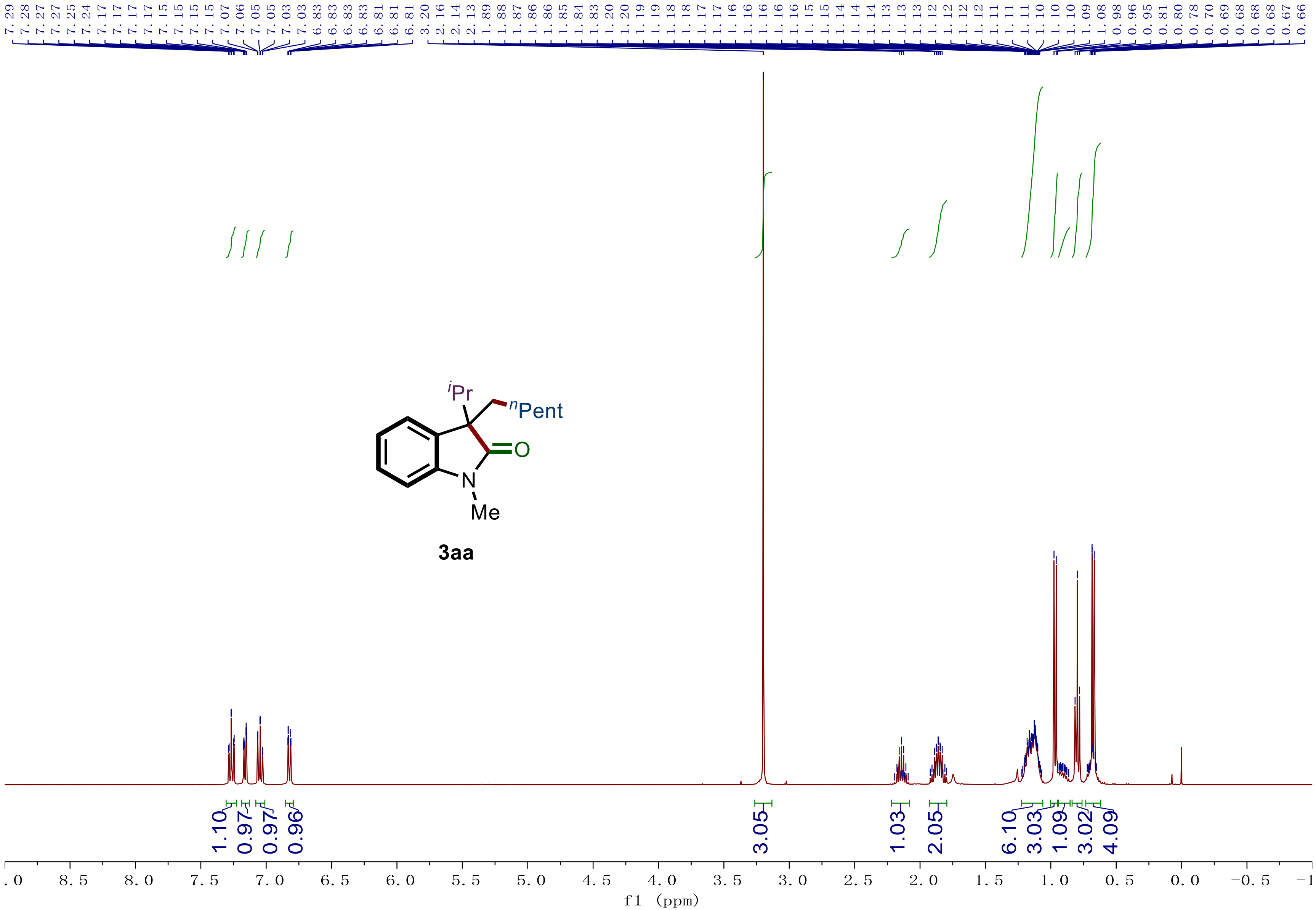

3aa

Supplementary Figure 102

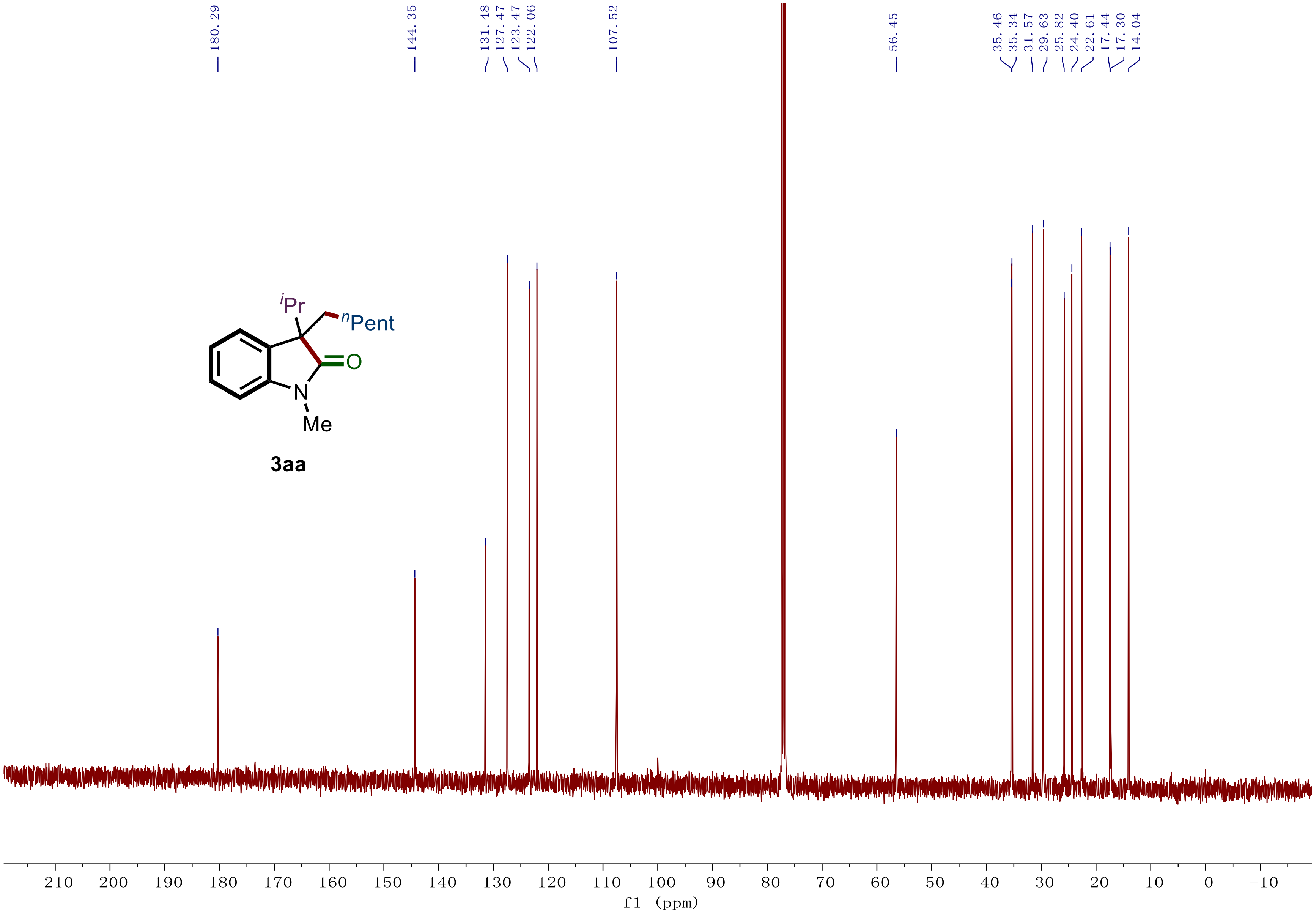

Supplementary Figure 103

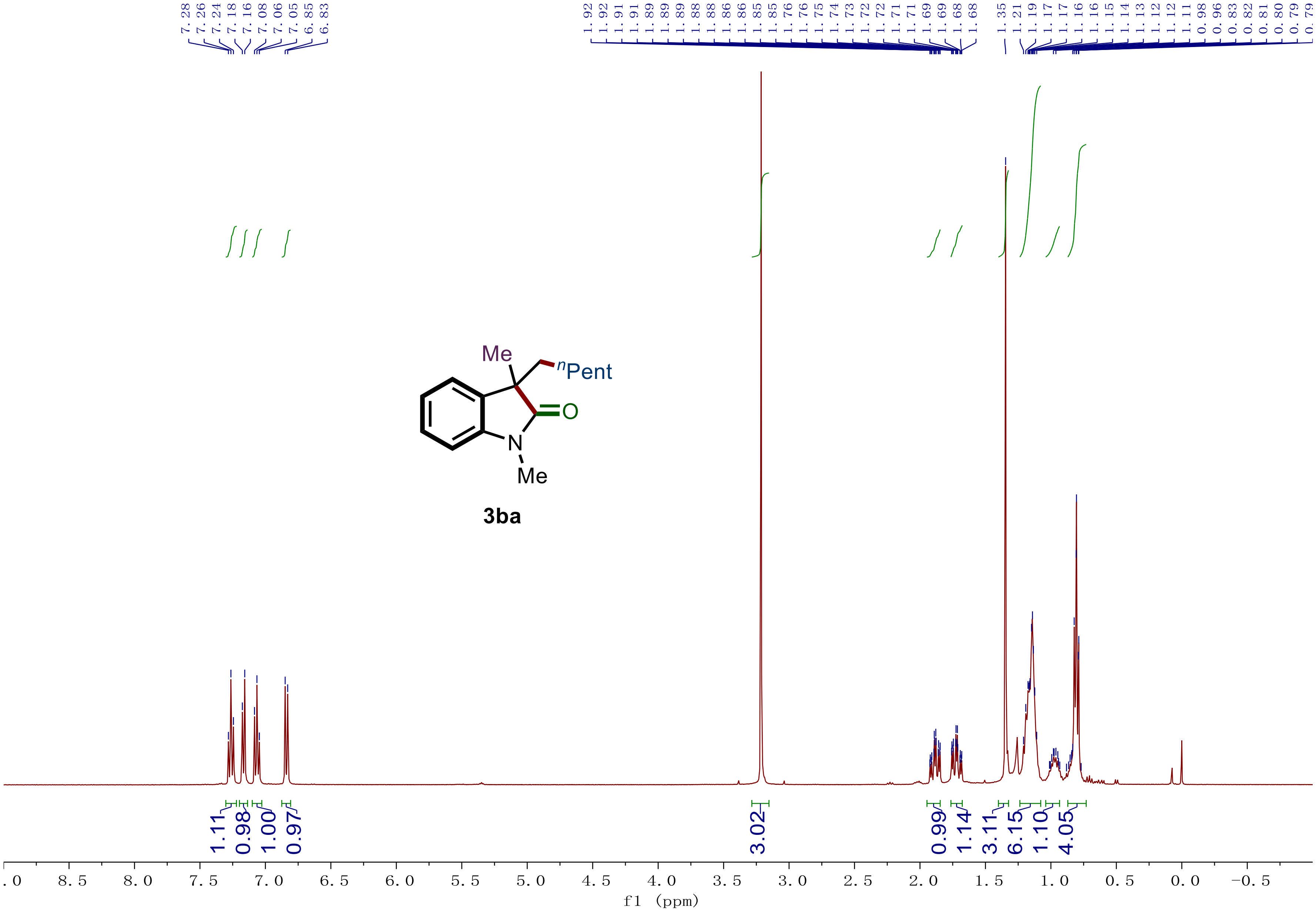

Supplementary Figure 104

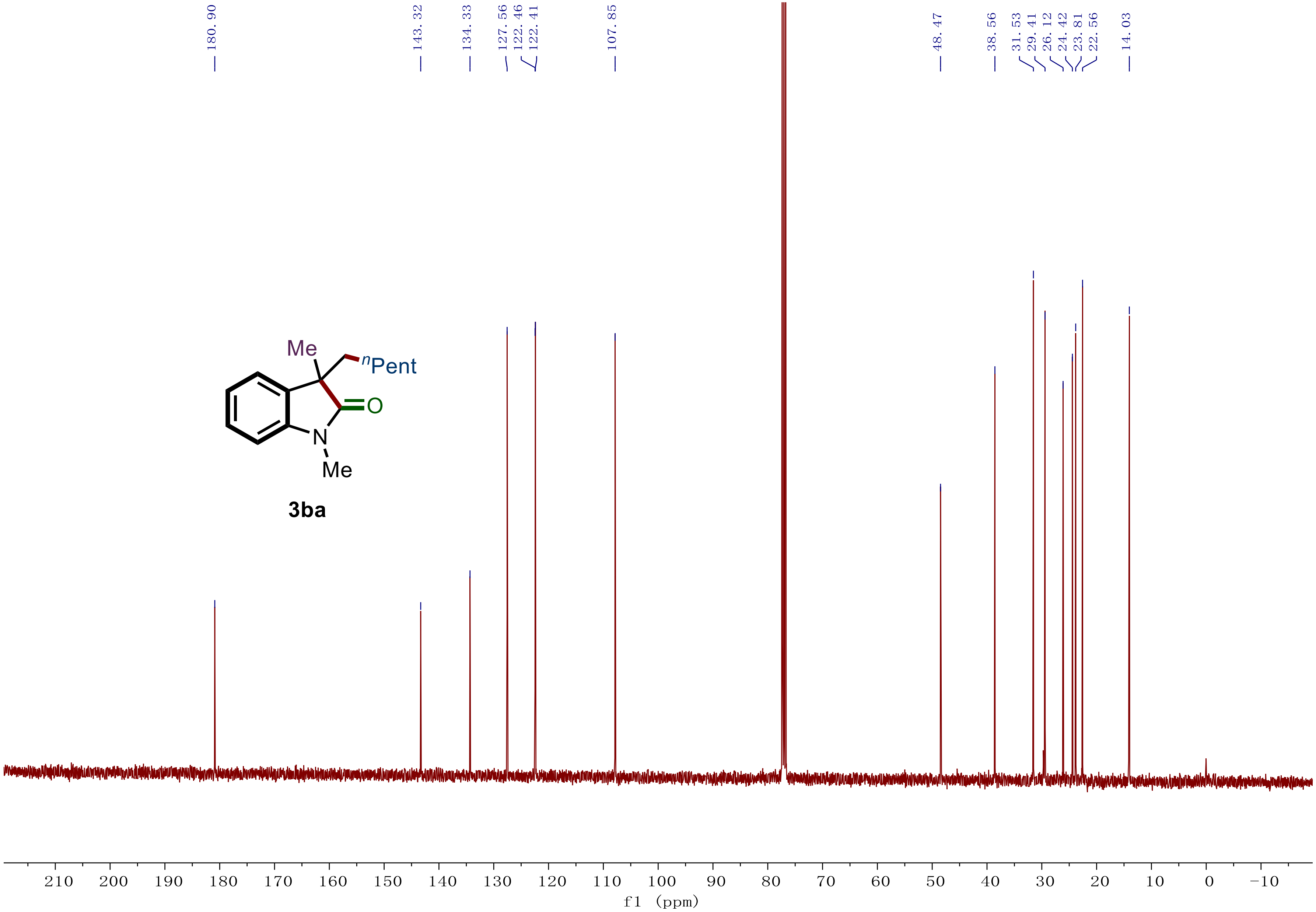

Supplementary Figure 105

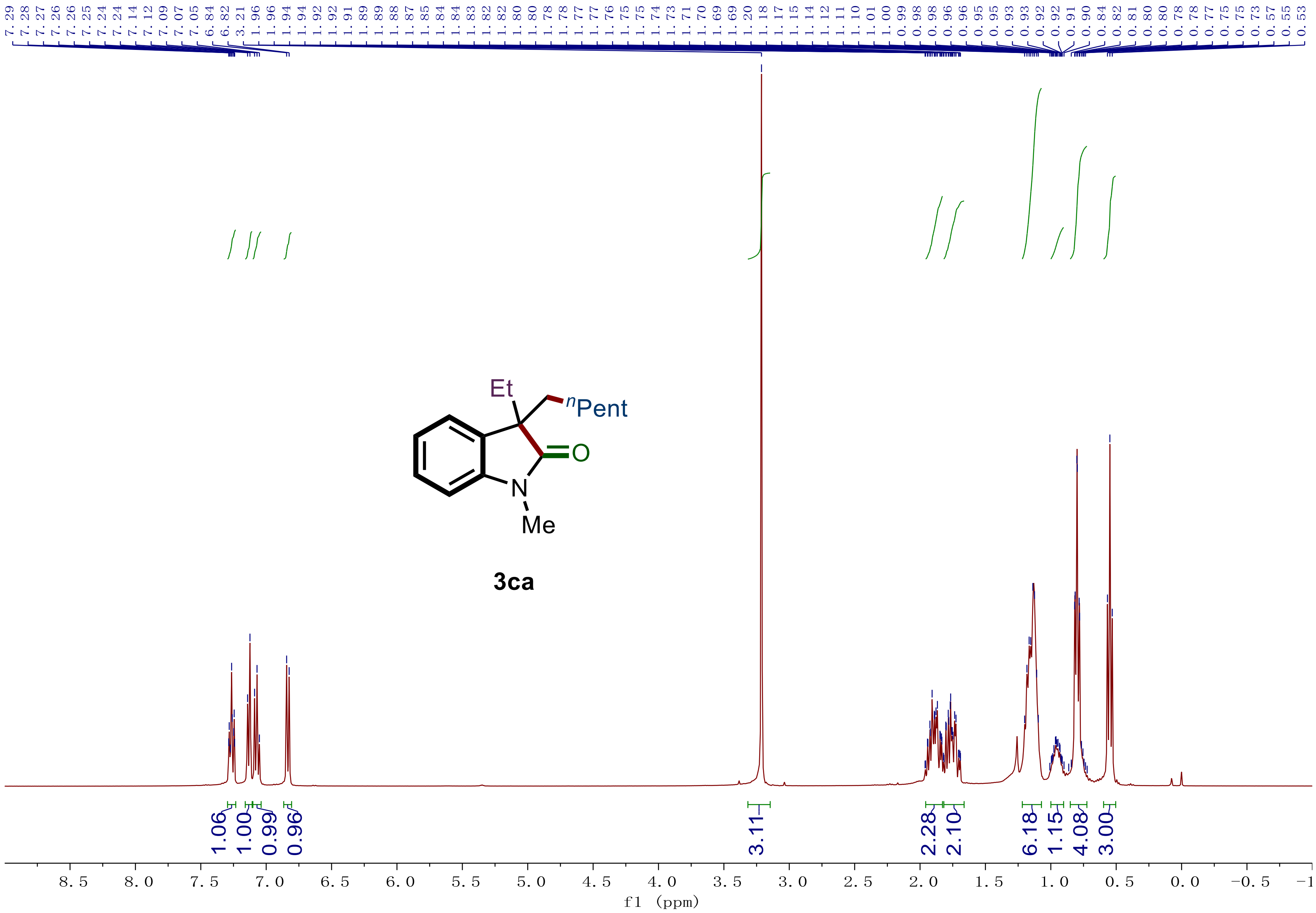

Supplementary Figure 106

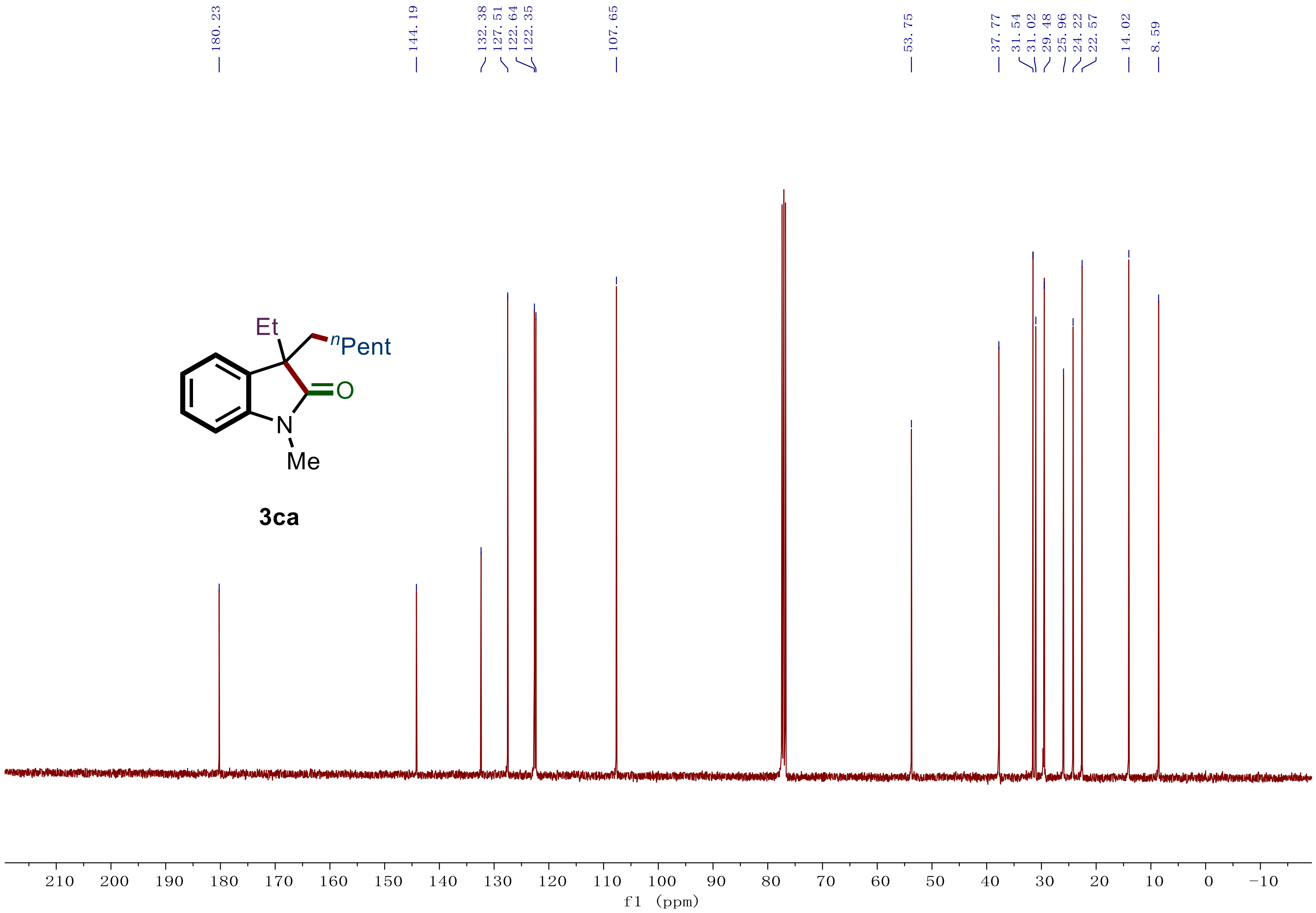

3ca

Supplementary Figure 107

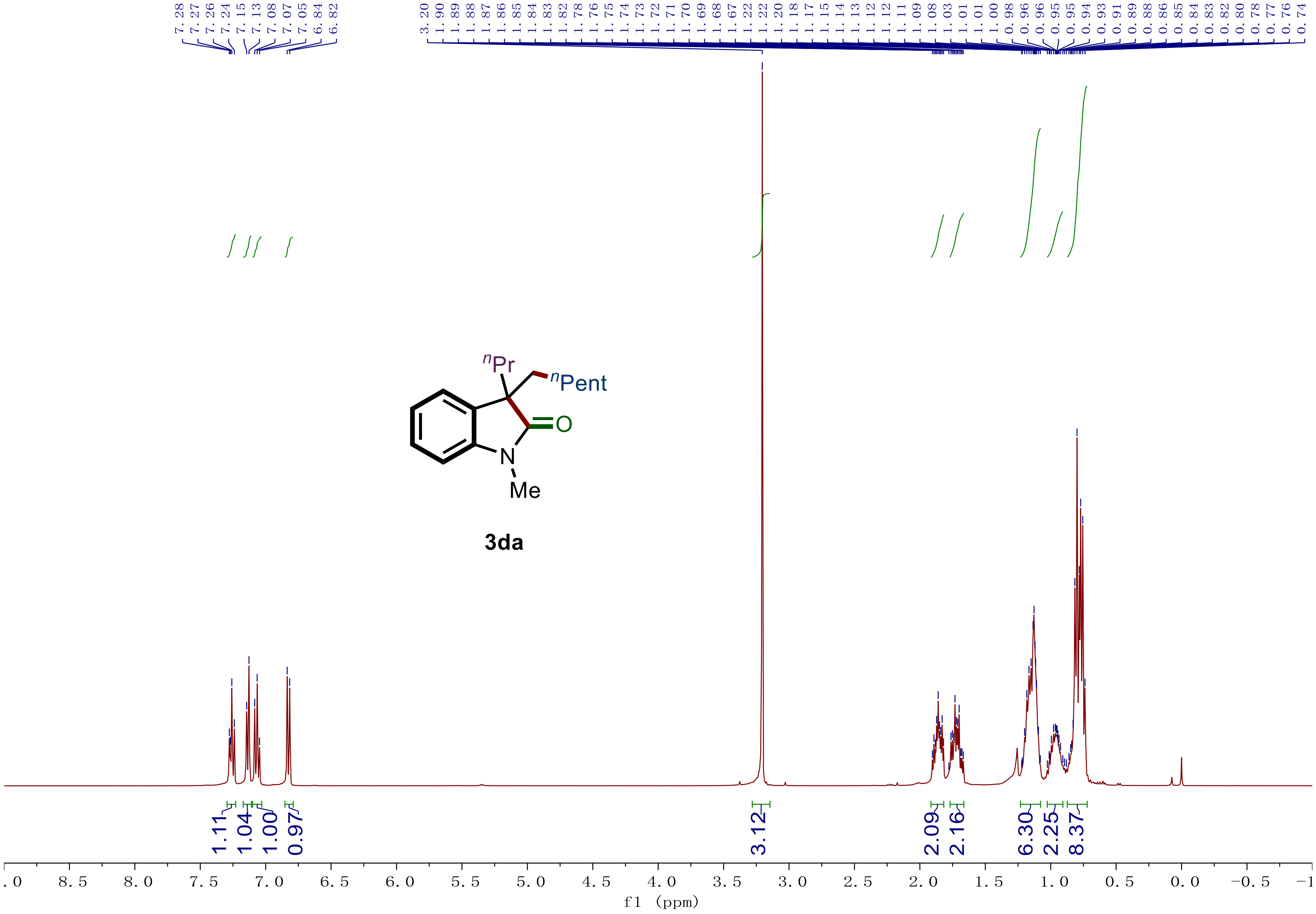

Supplementary Figure 108

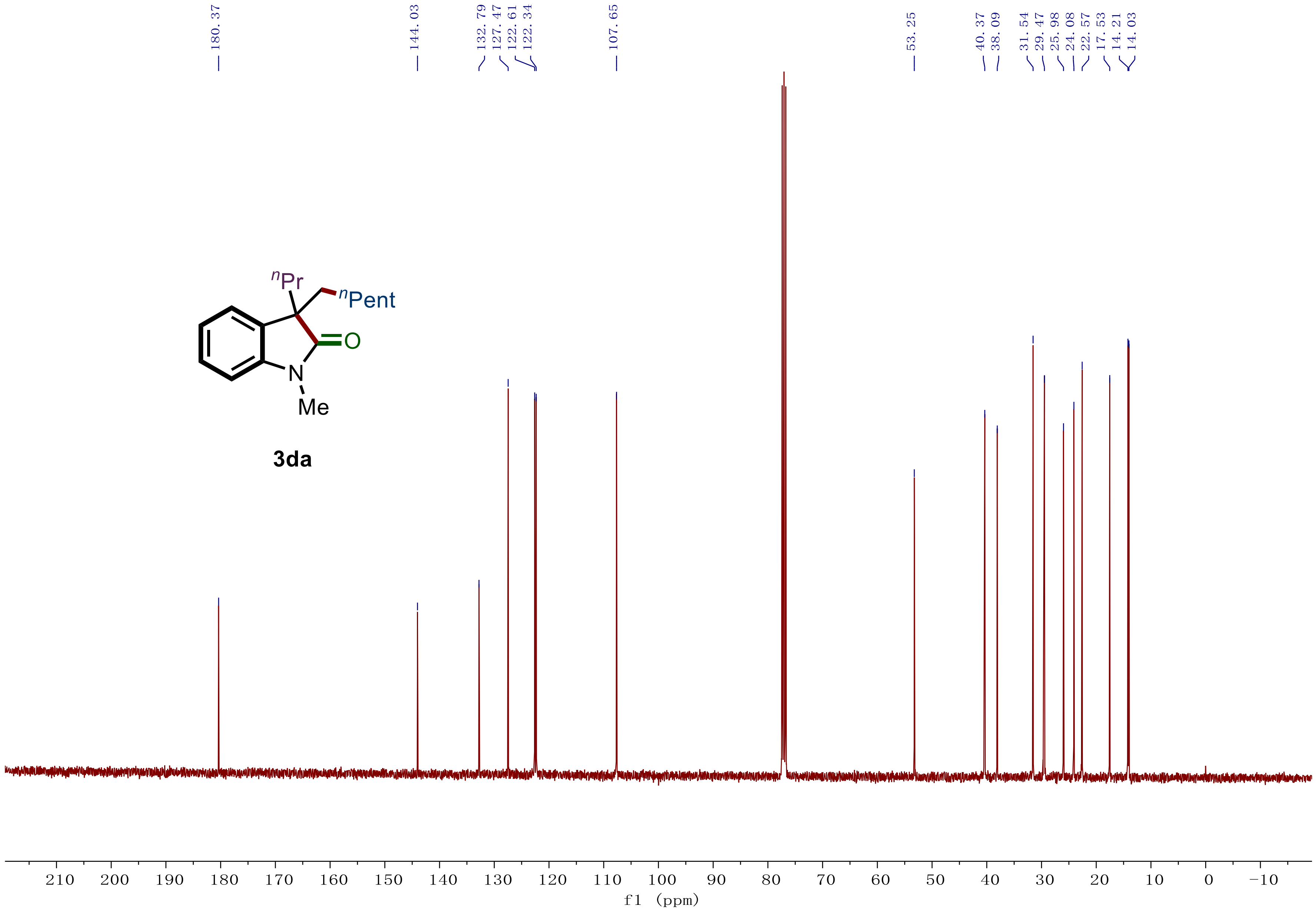

Supplementary Figure 109

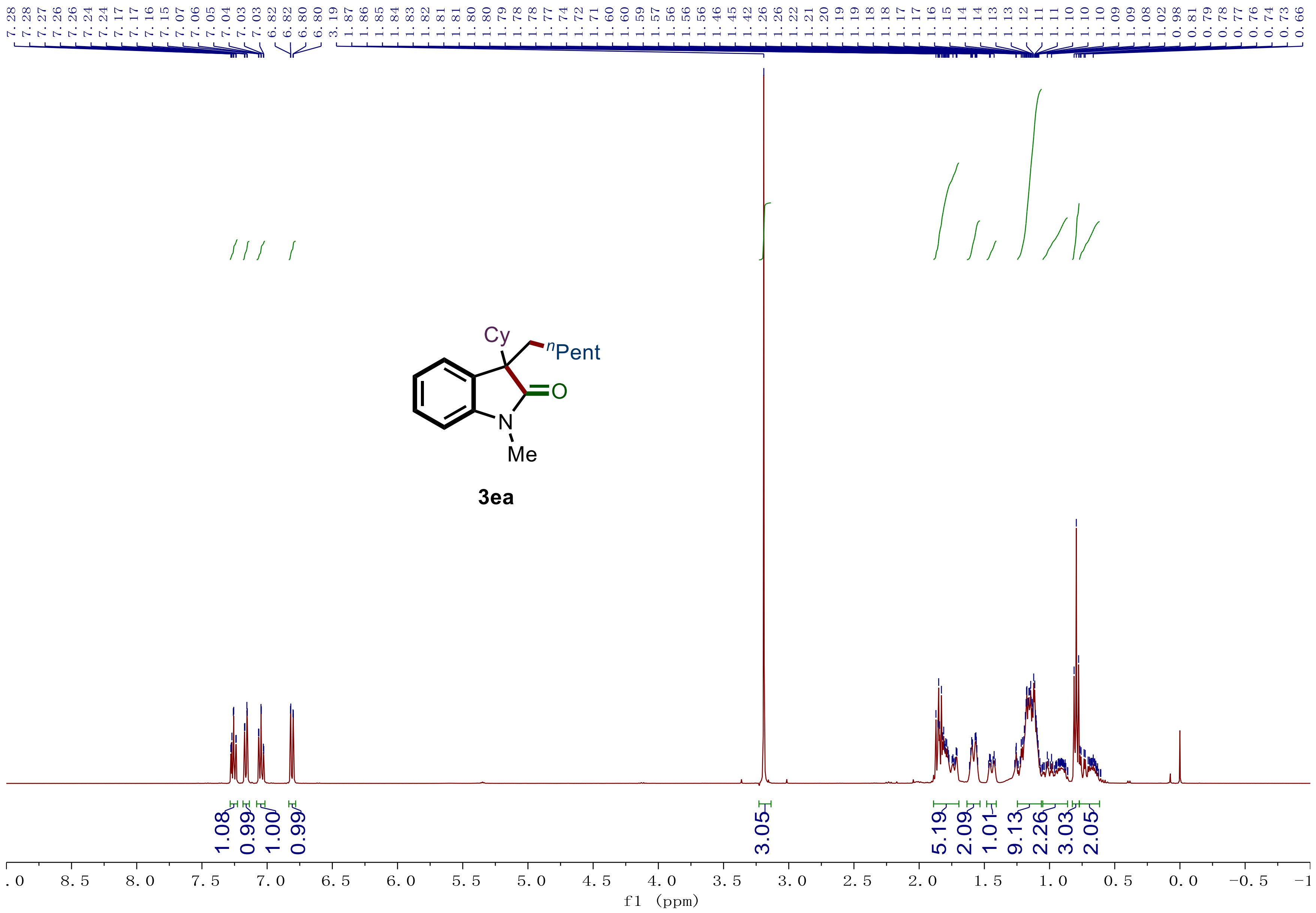

Supplementary Figure 110

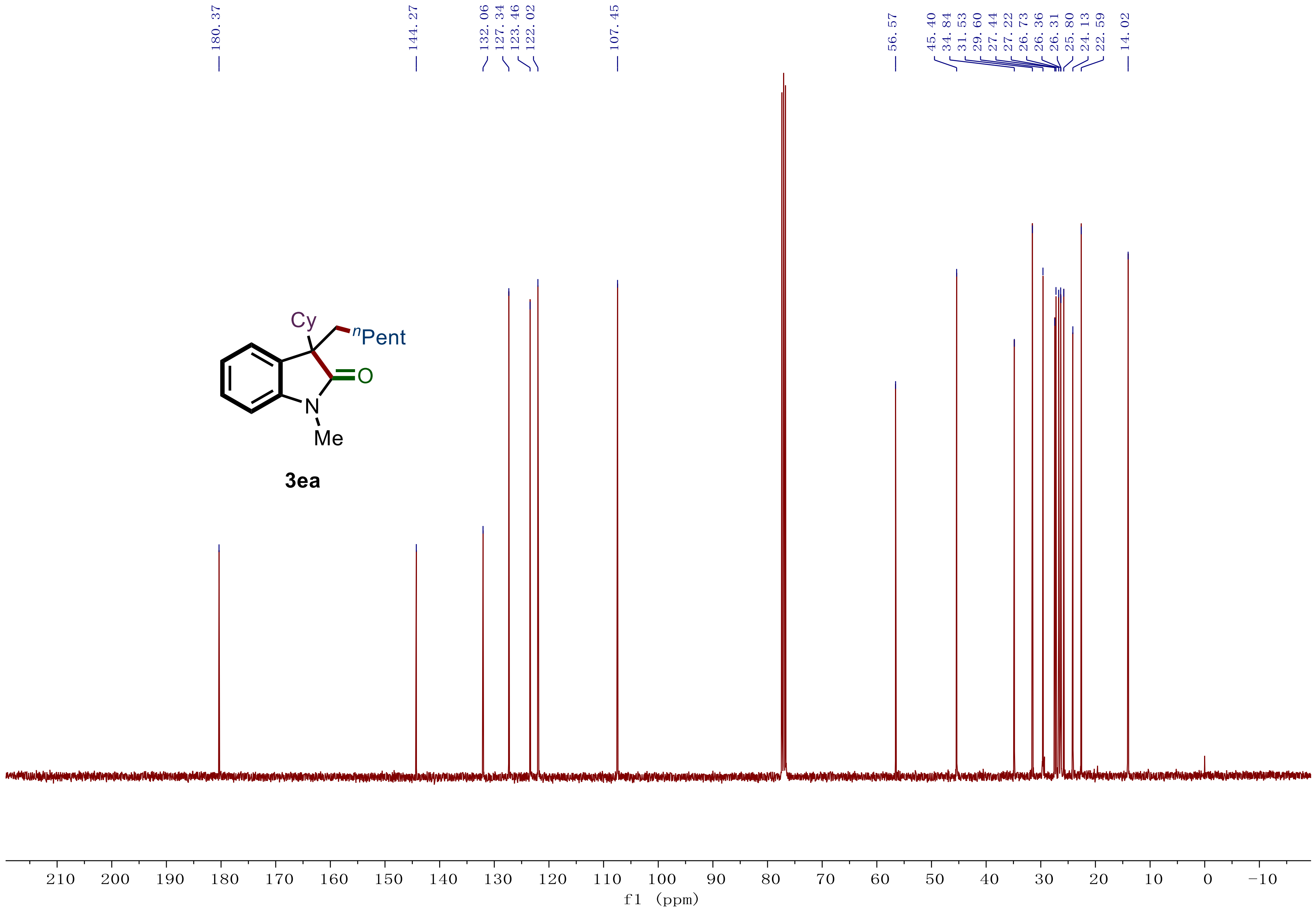

Supplementary Figure 111

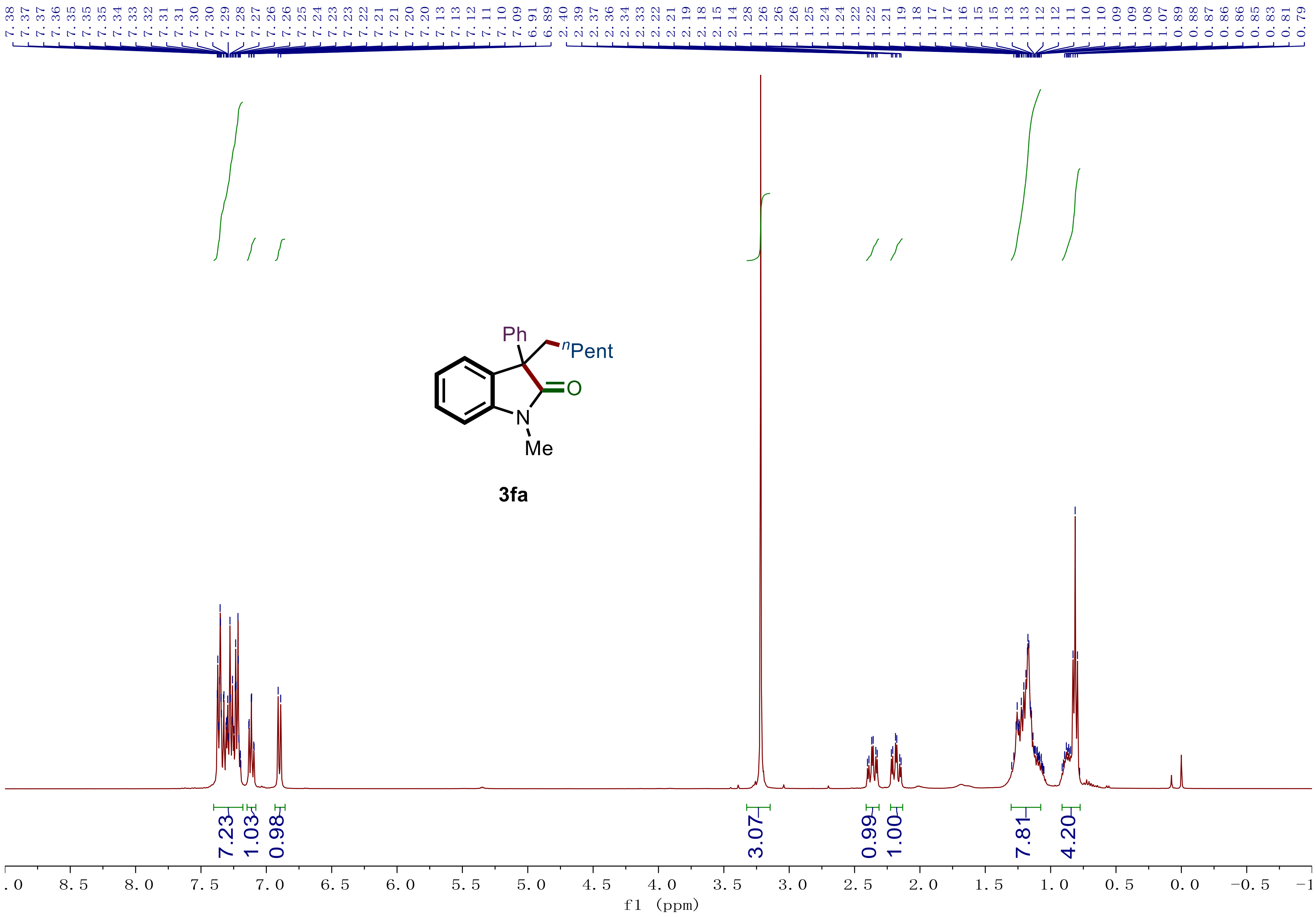

Supplementary Figure 112

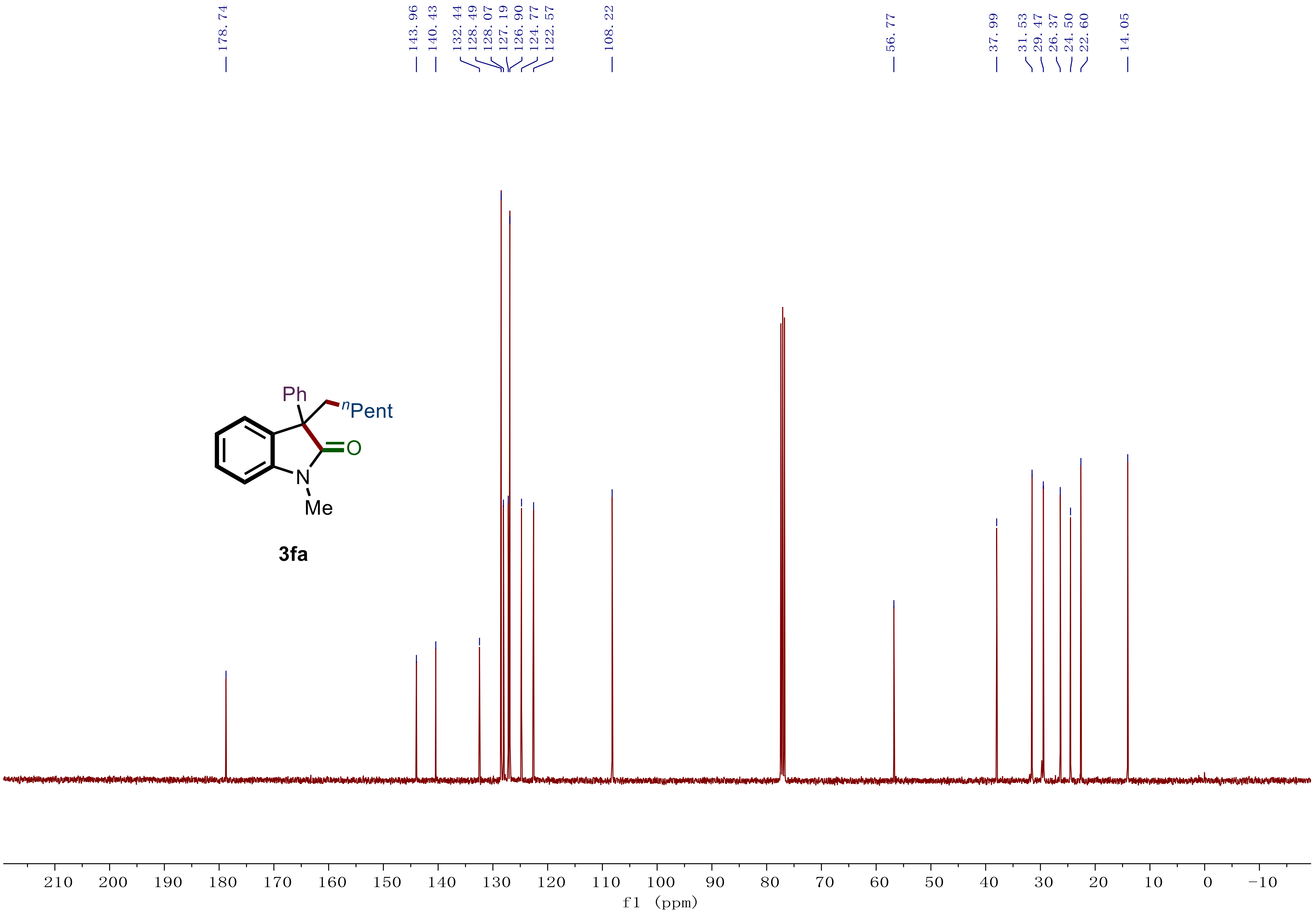

3fa

Supplementary Figure 113

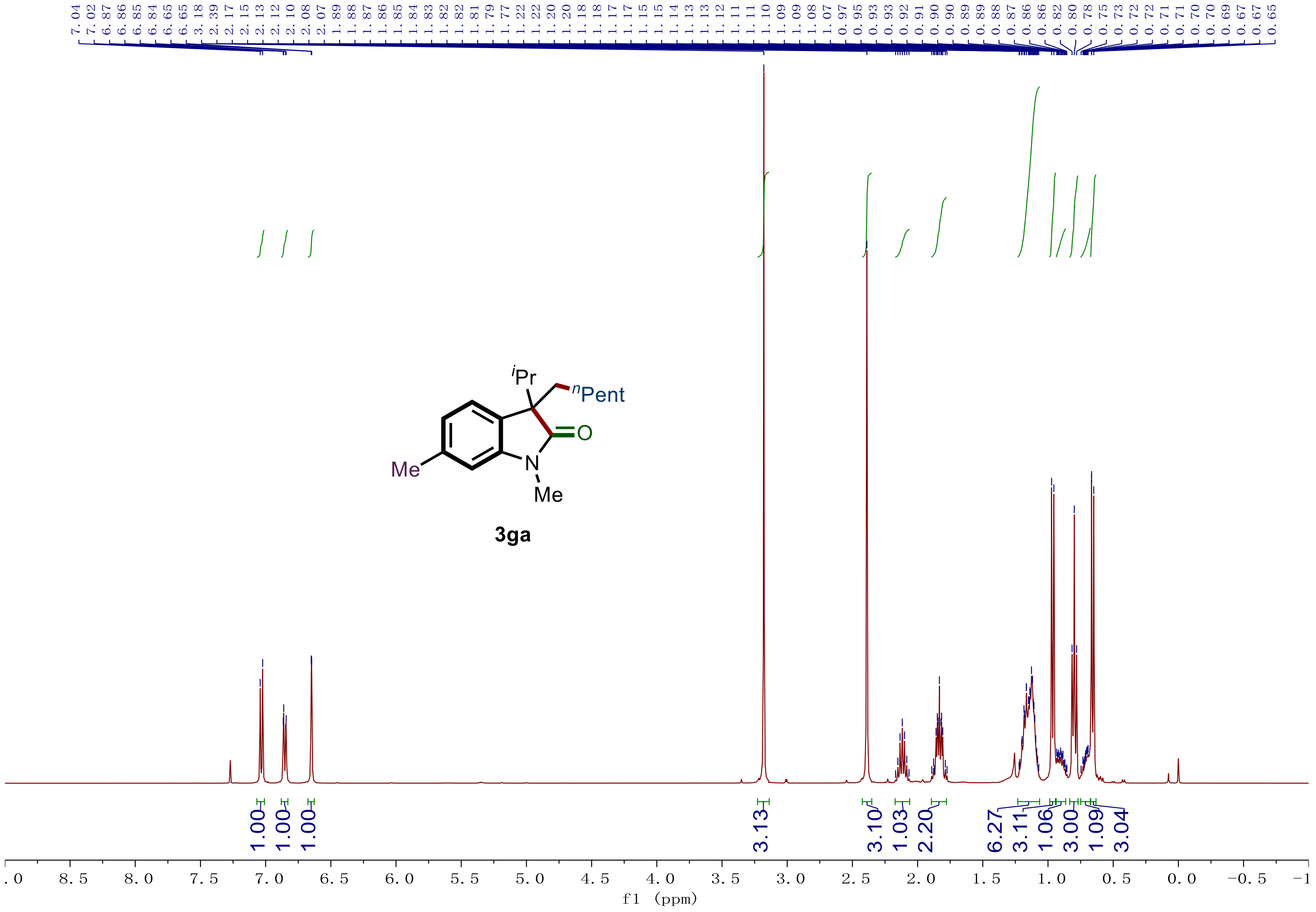

Supplementary Figure 114

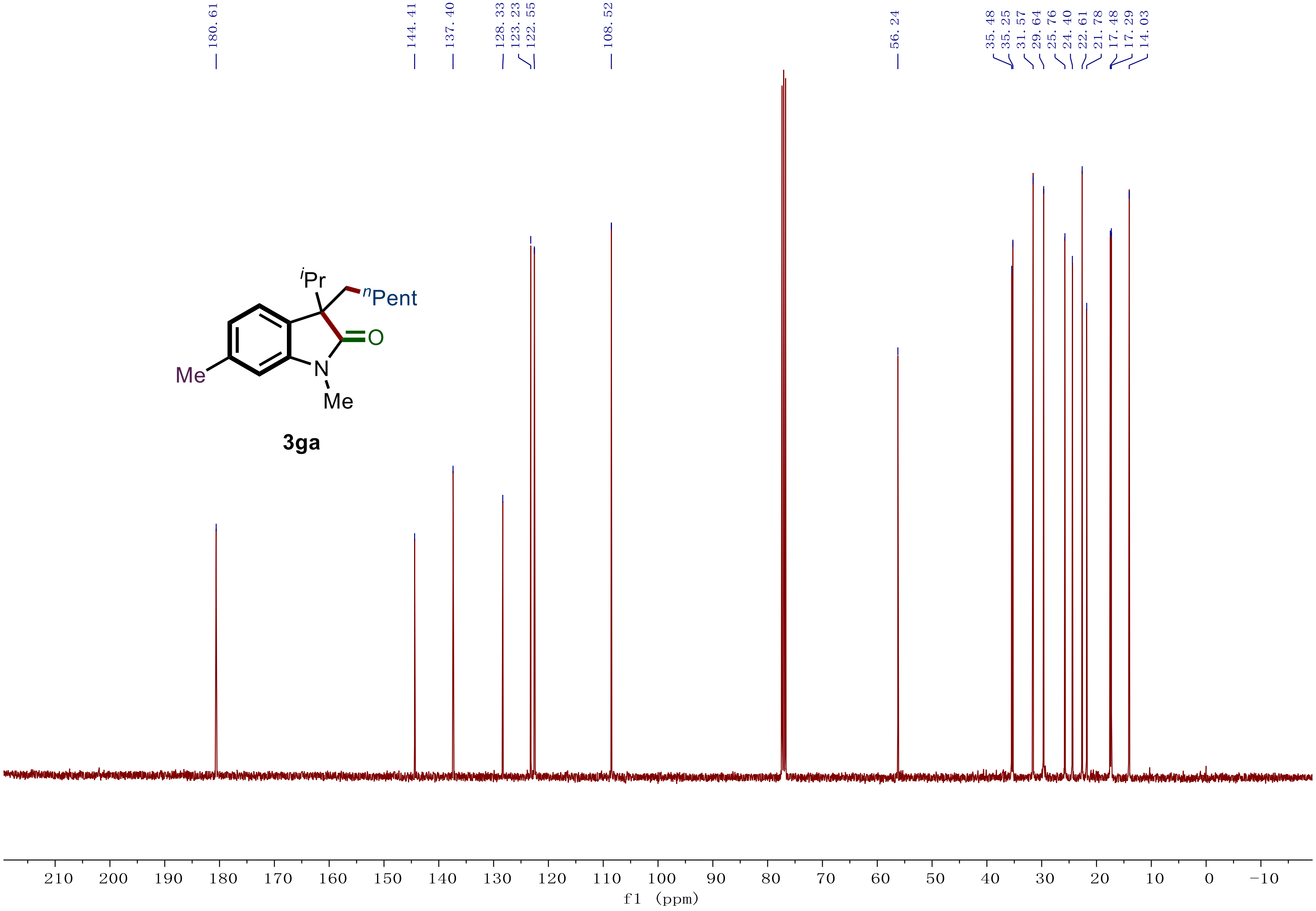

Supplementary Figure 115

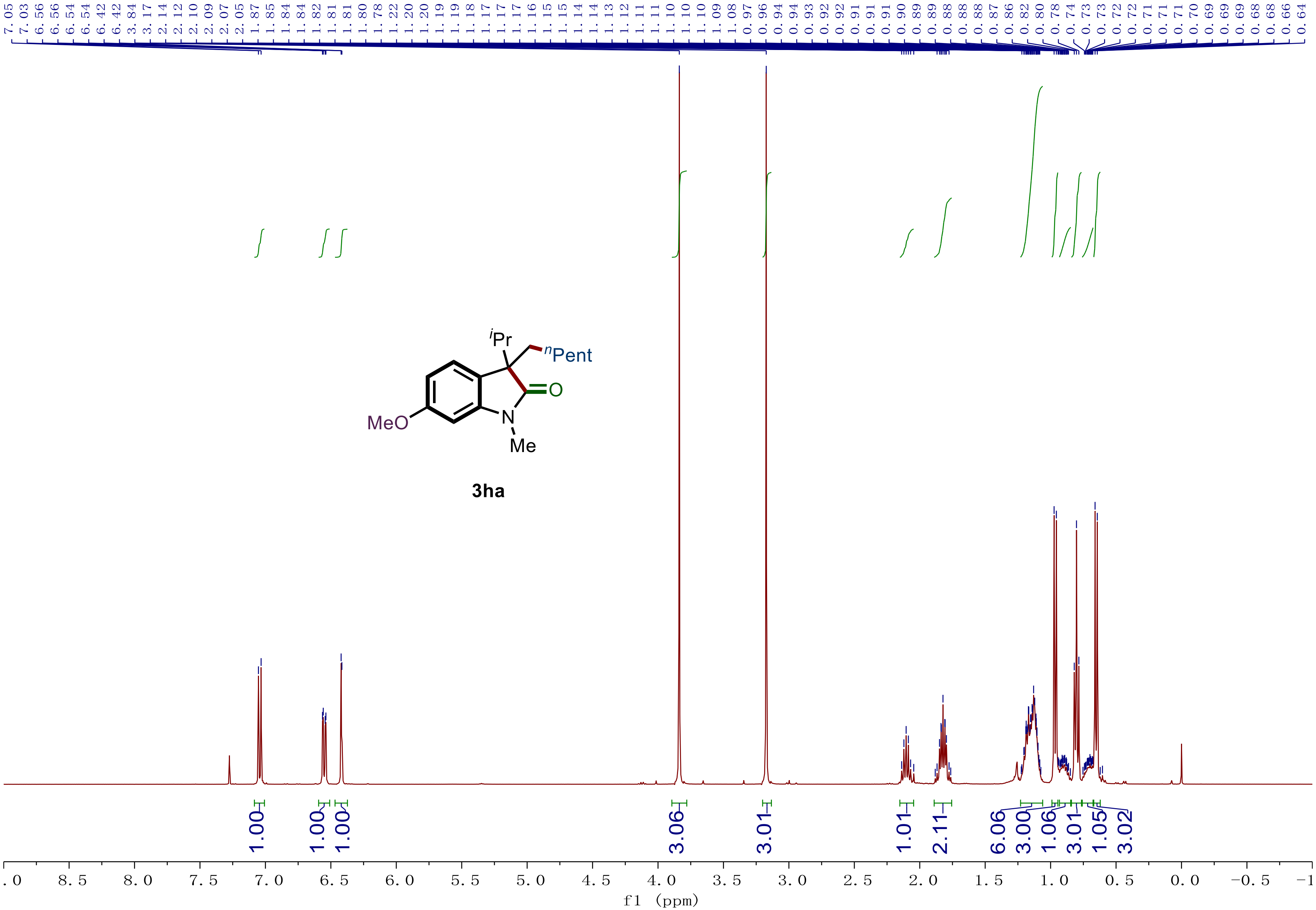

Supplementary Figure 116

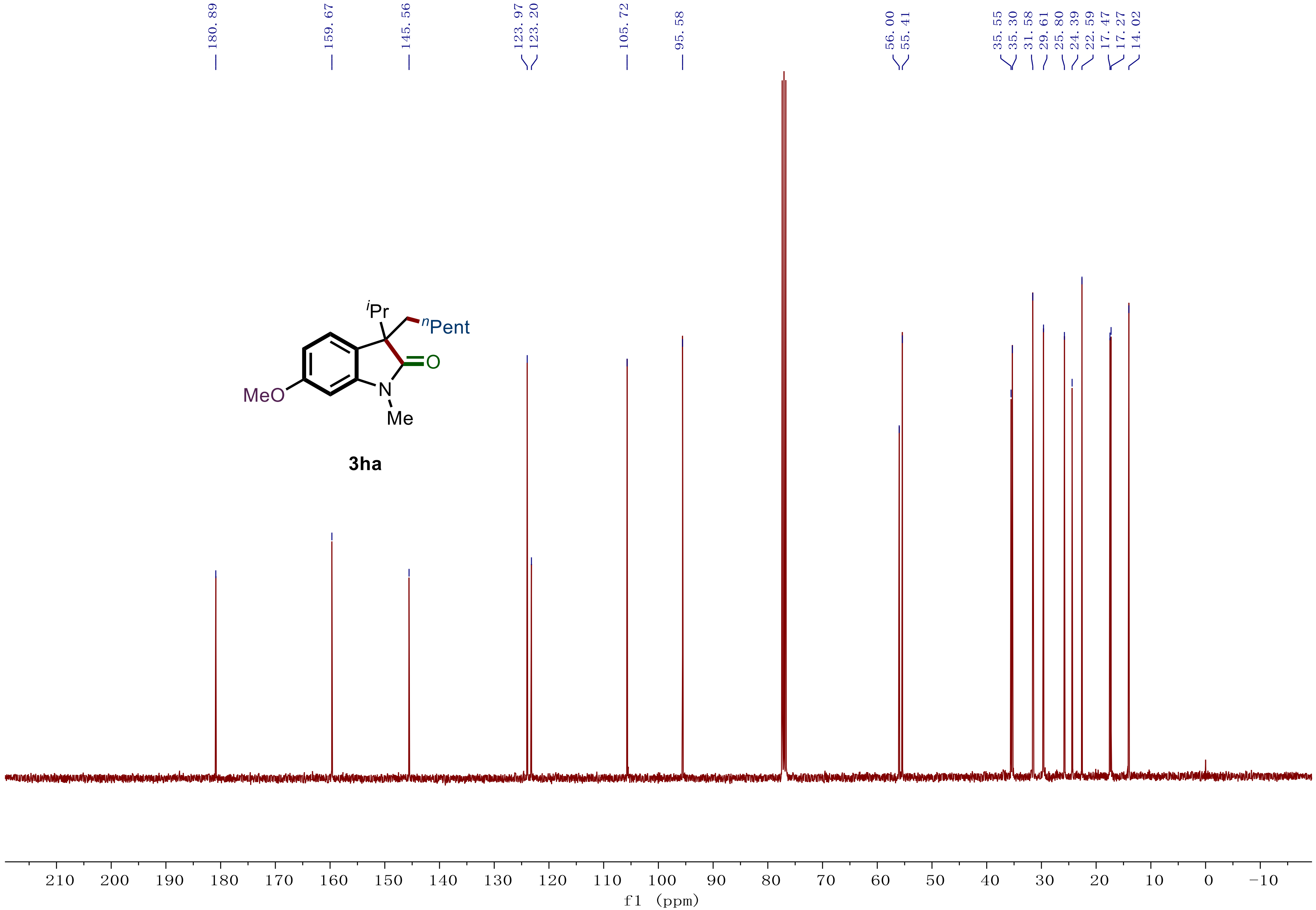

3ha

Supplementary Figure 117

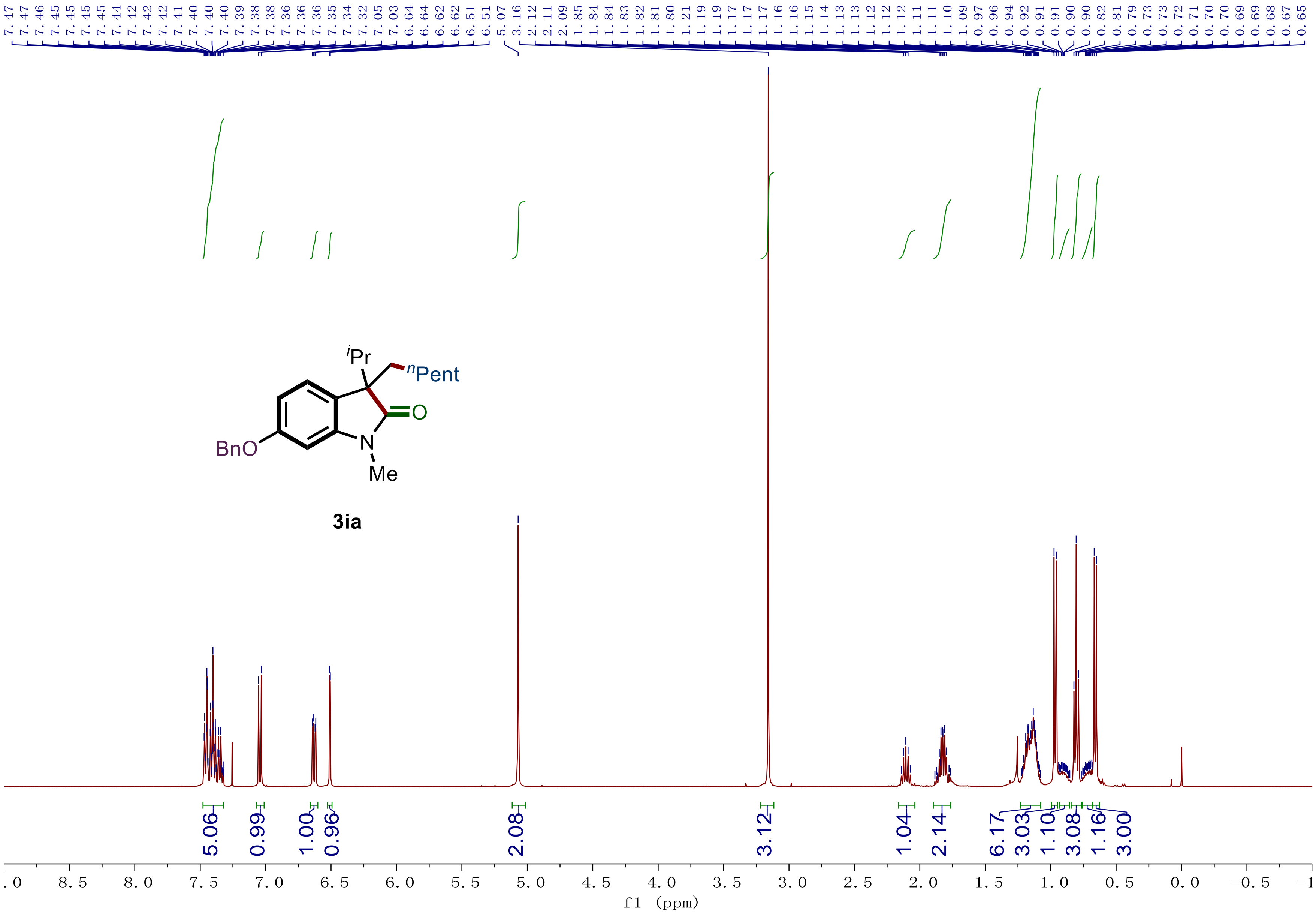

Supplementary Figure 118

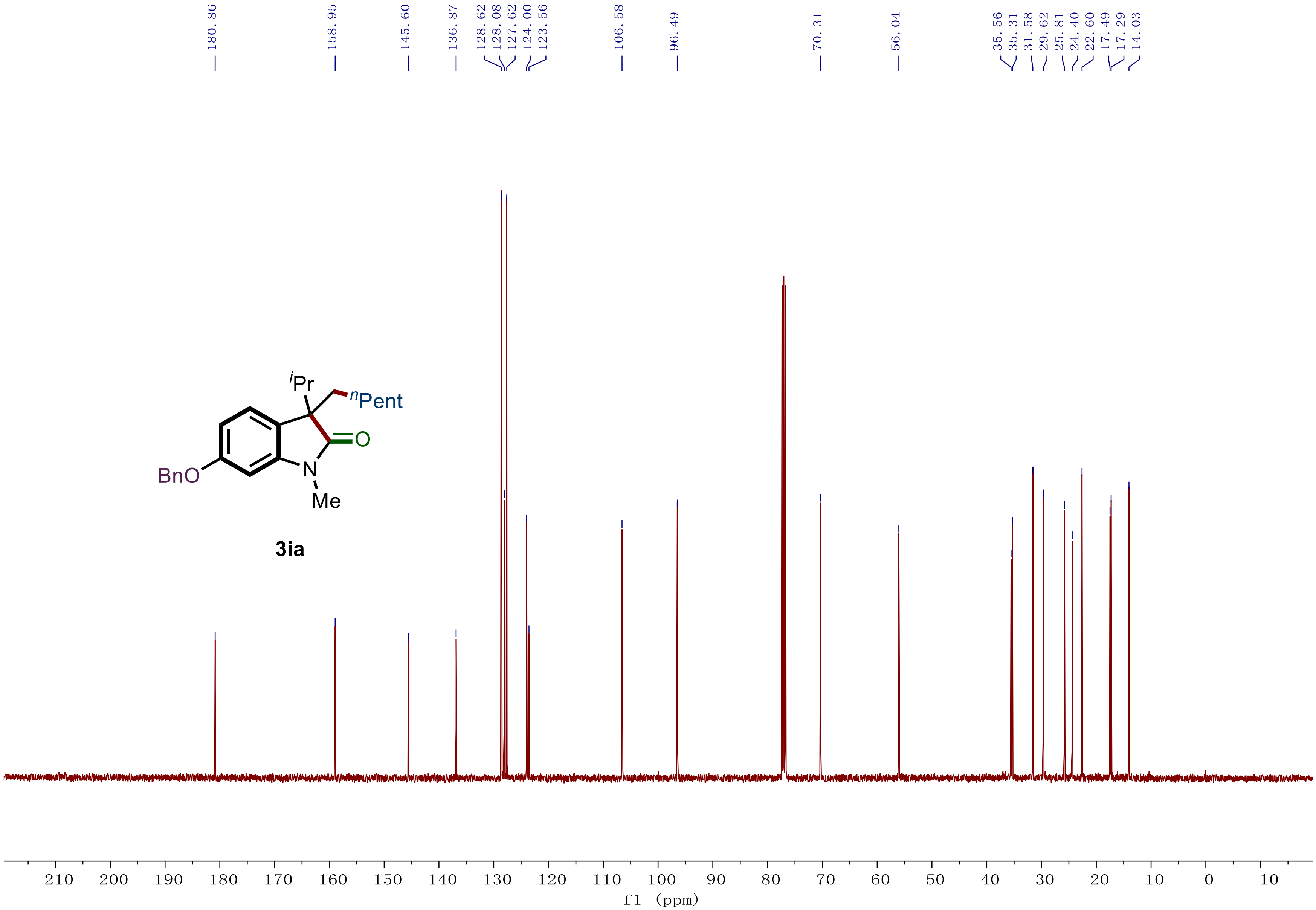

Supplementary Figure 119

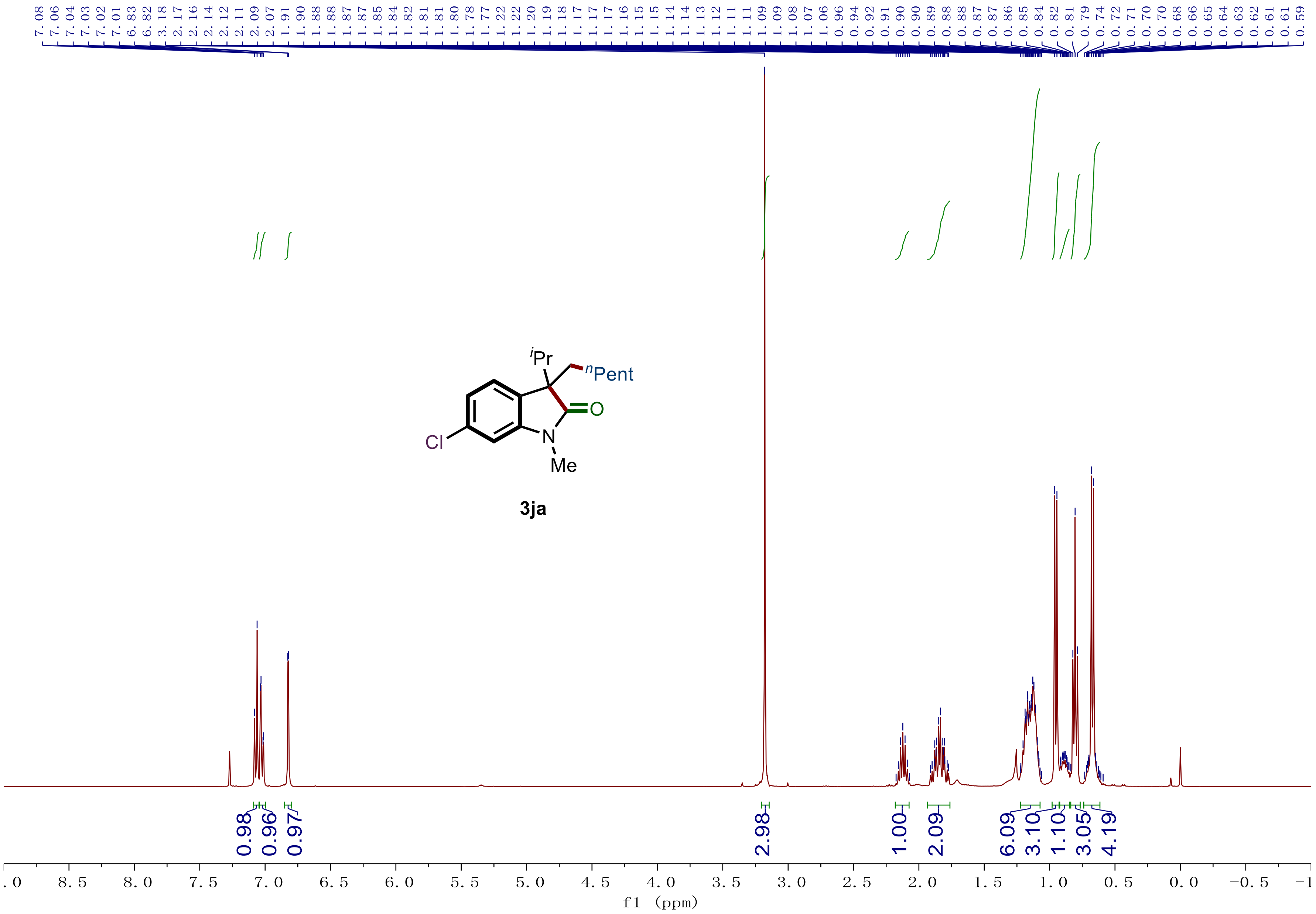

3ja

Supplementary Figure 120

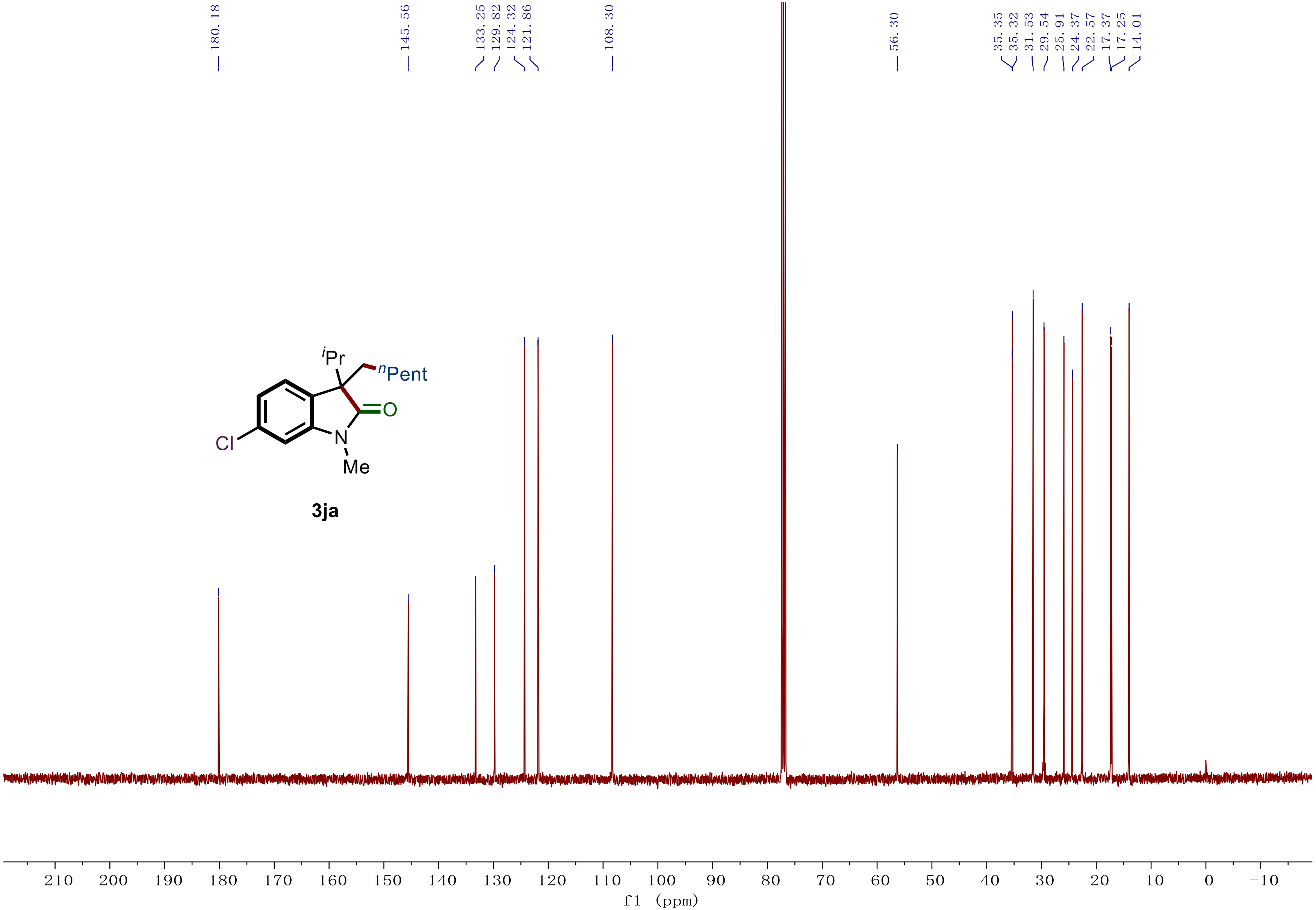

3ja

Supplementary Figure 121

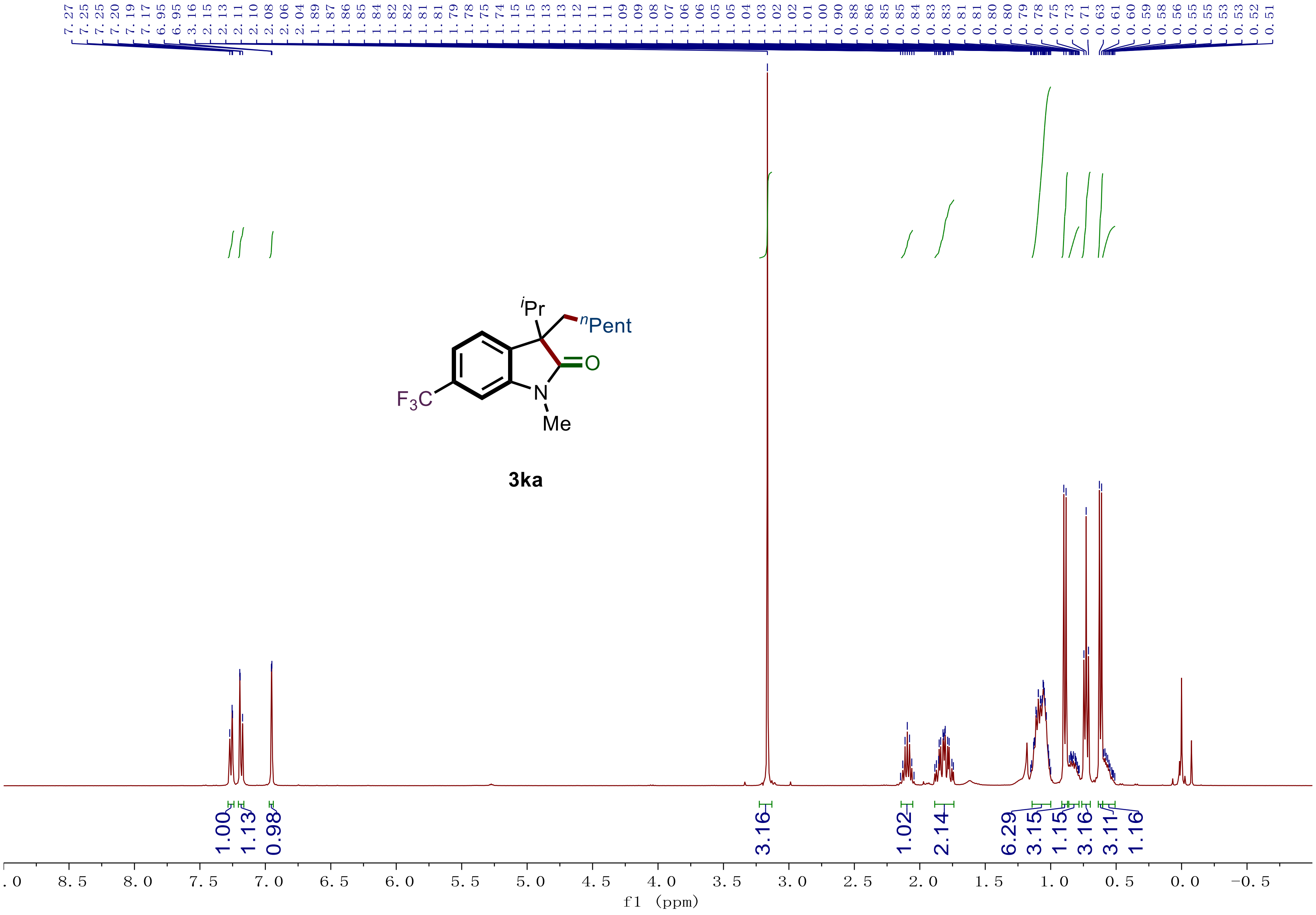

Supplementary Figure 122

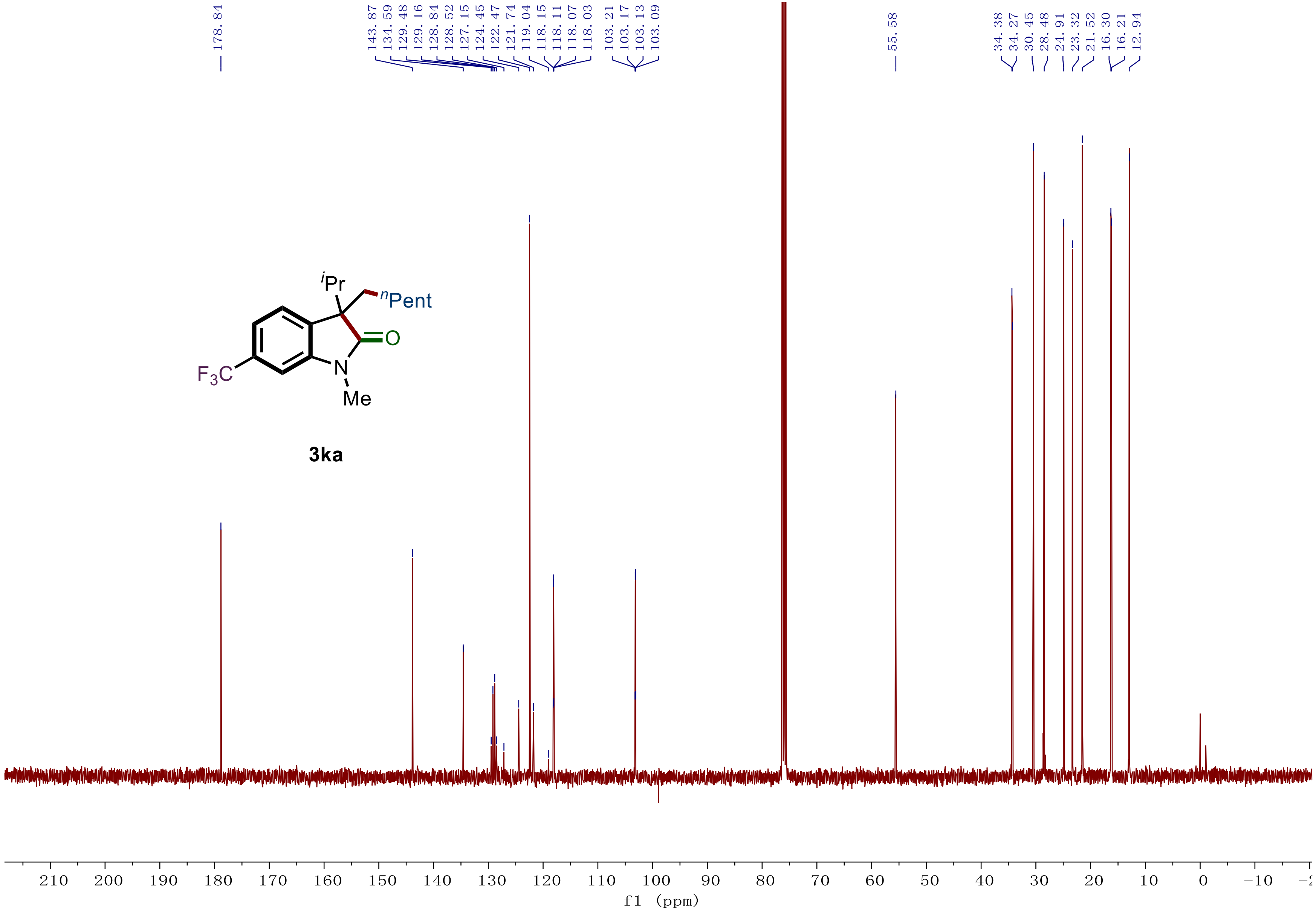

Supplementary Figure 123

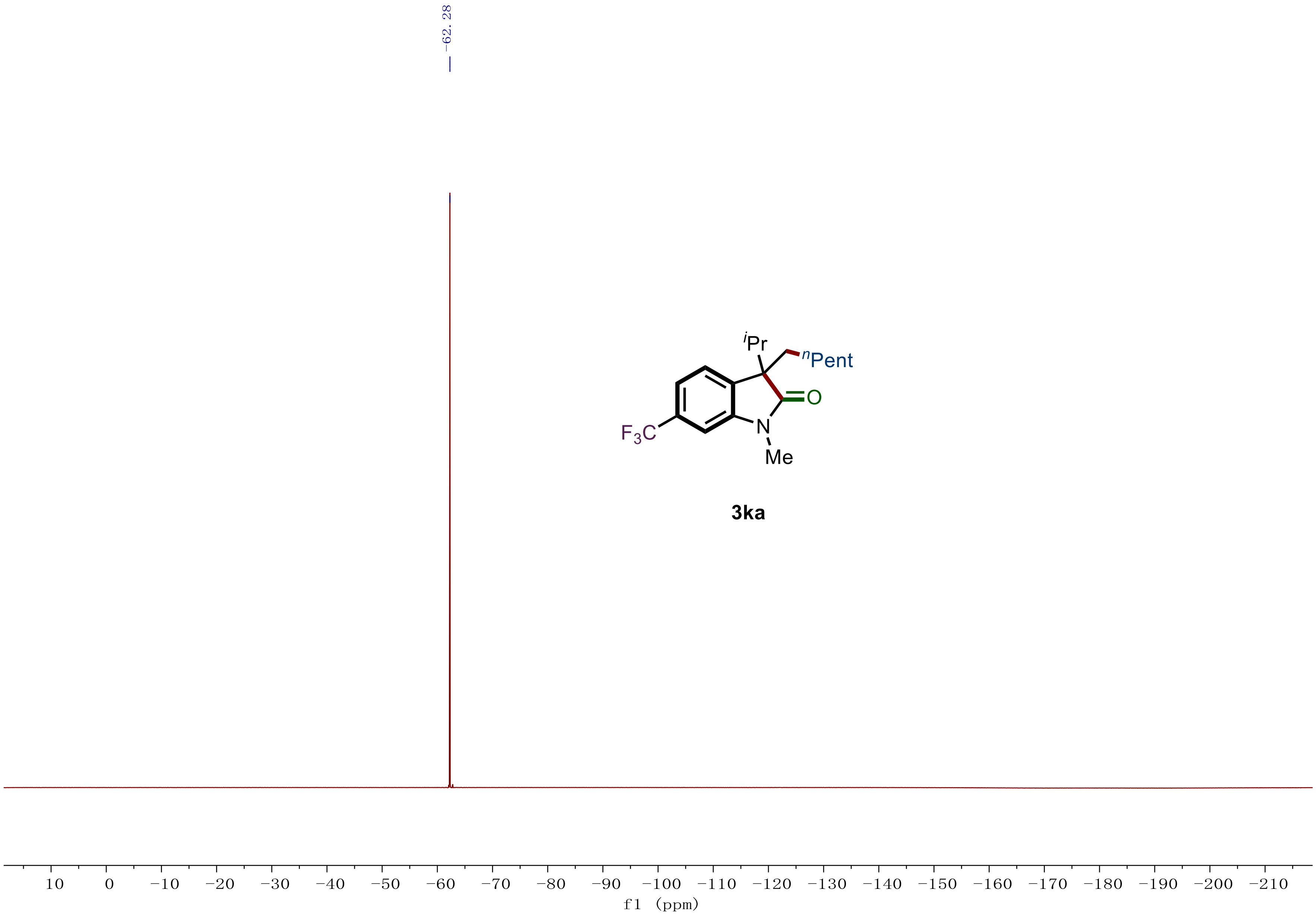

3ka

Supplementary Figure 124

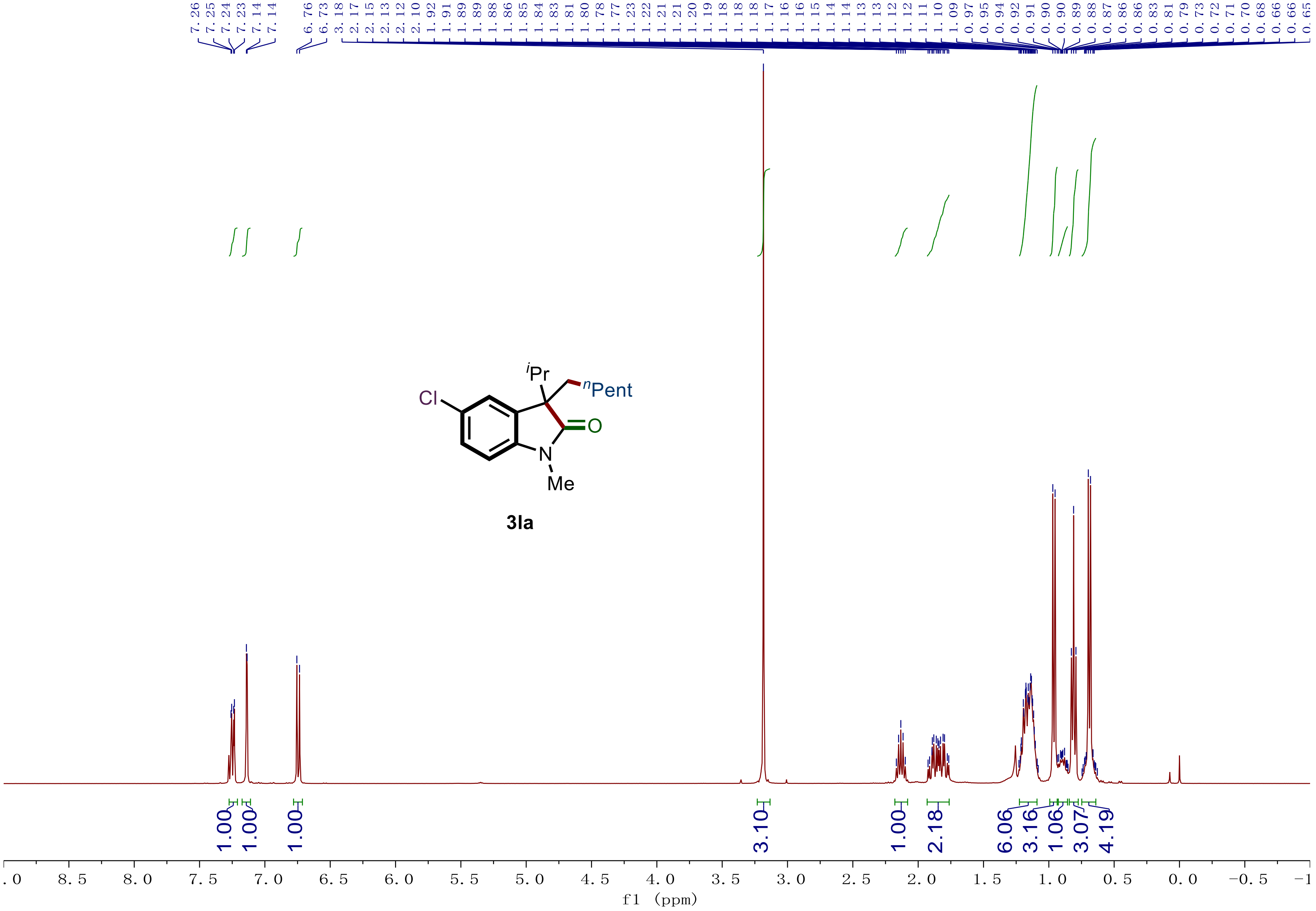

3la

Supplementary Figure 125

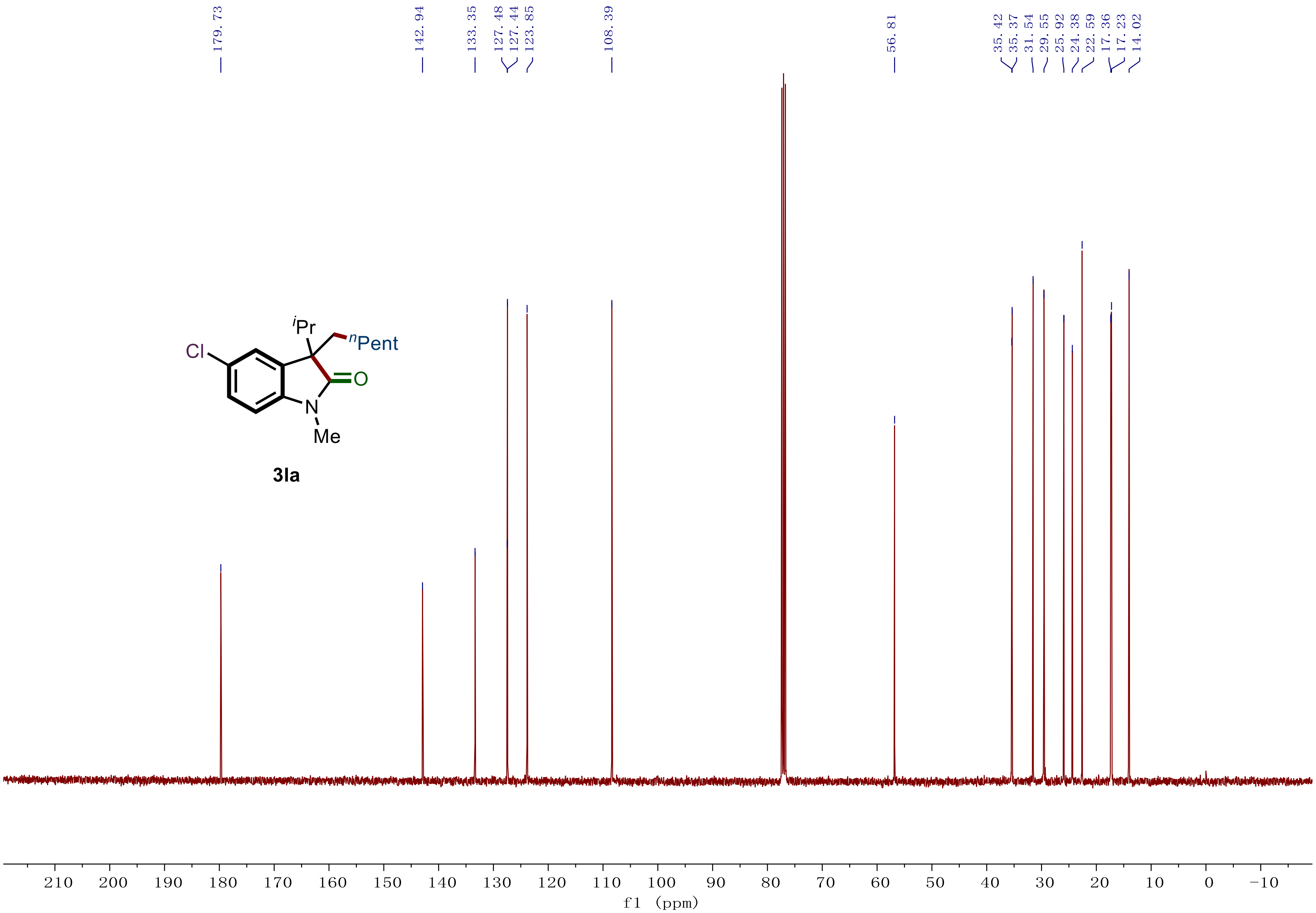

Supplementary Figure 126

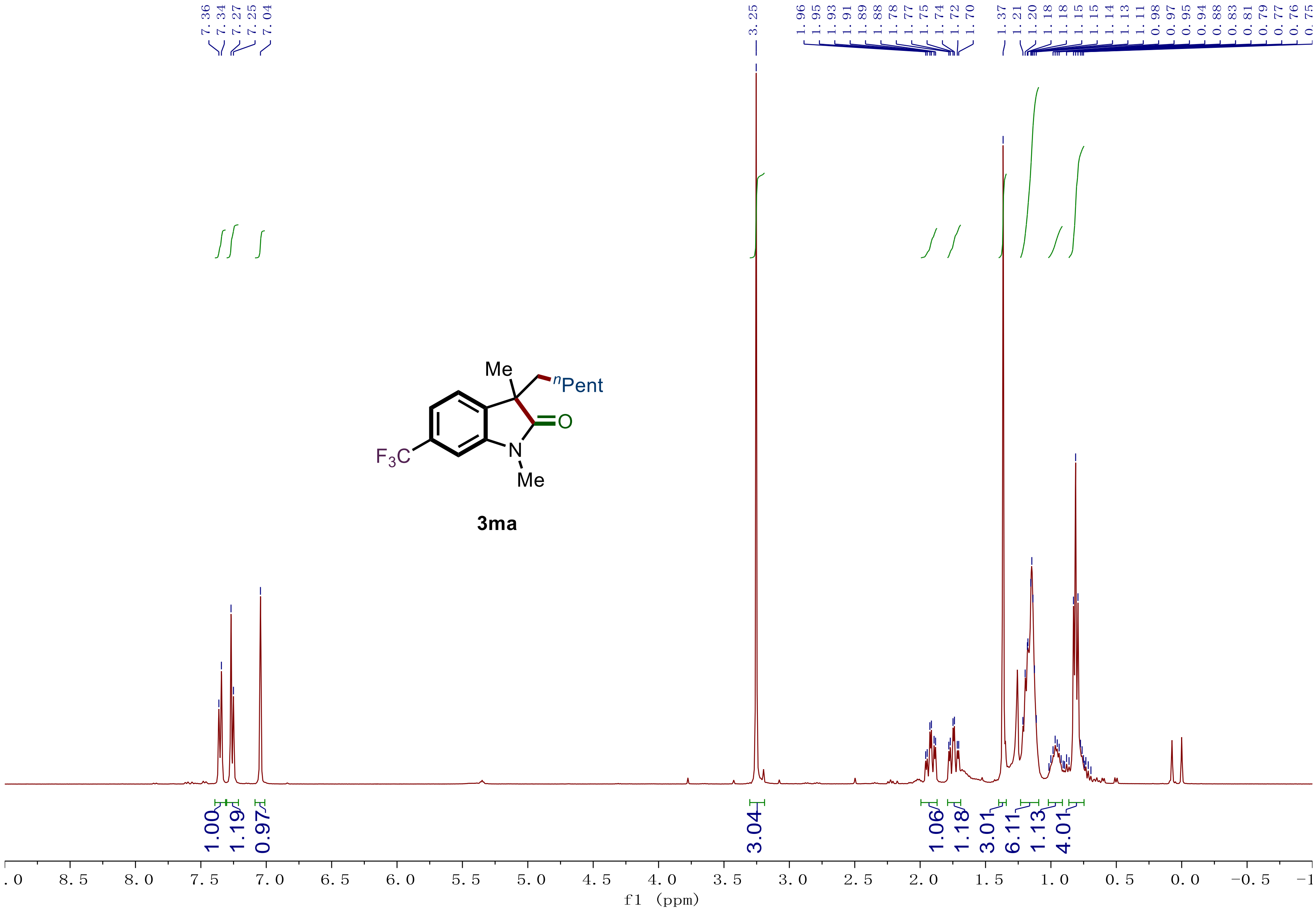

3ma

Supplementary Figure 127

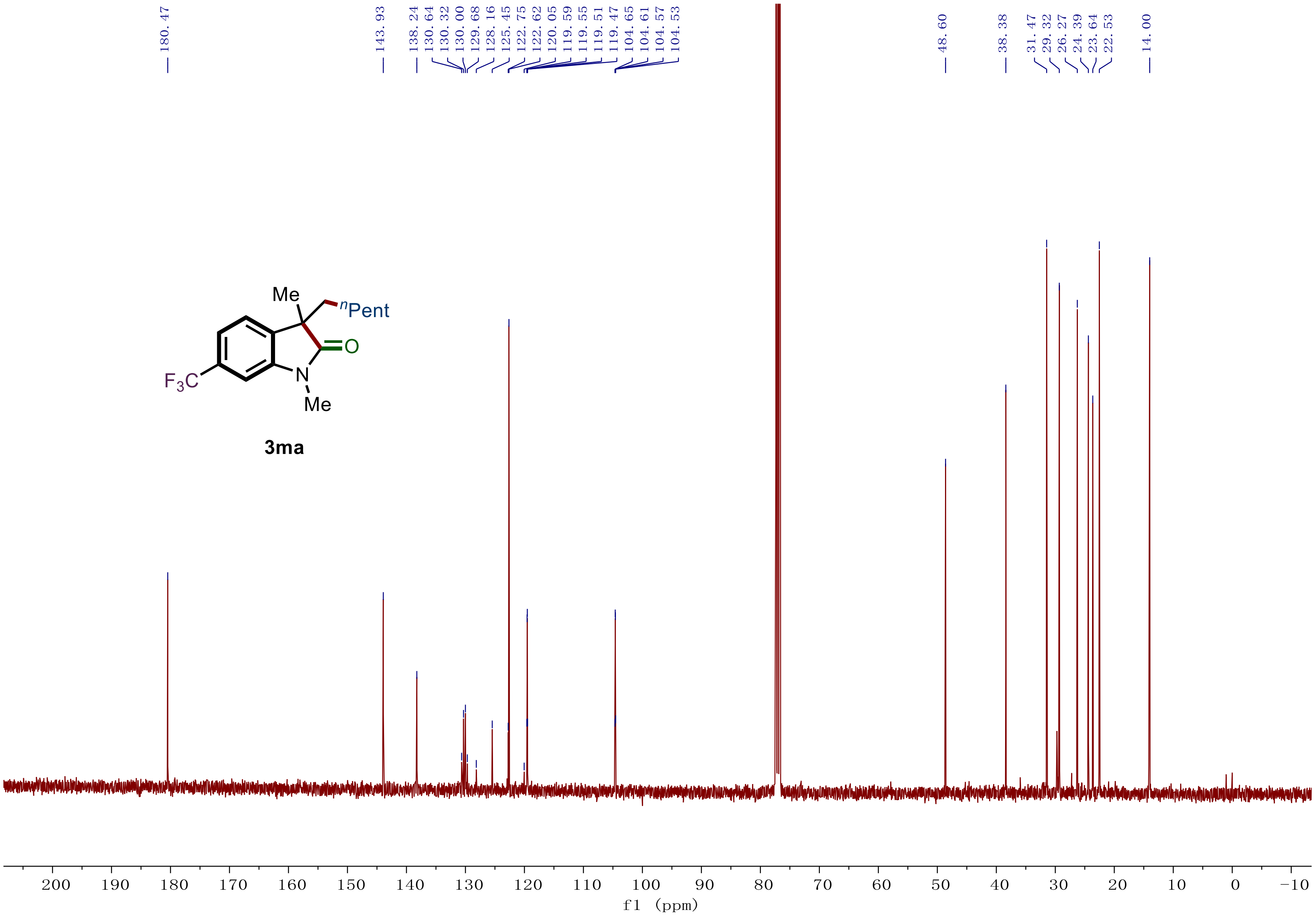

Supplementary Figure 128

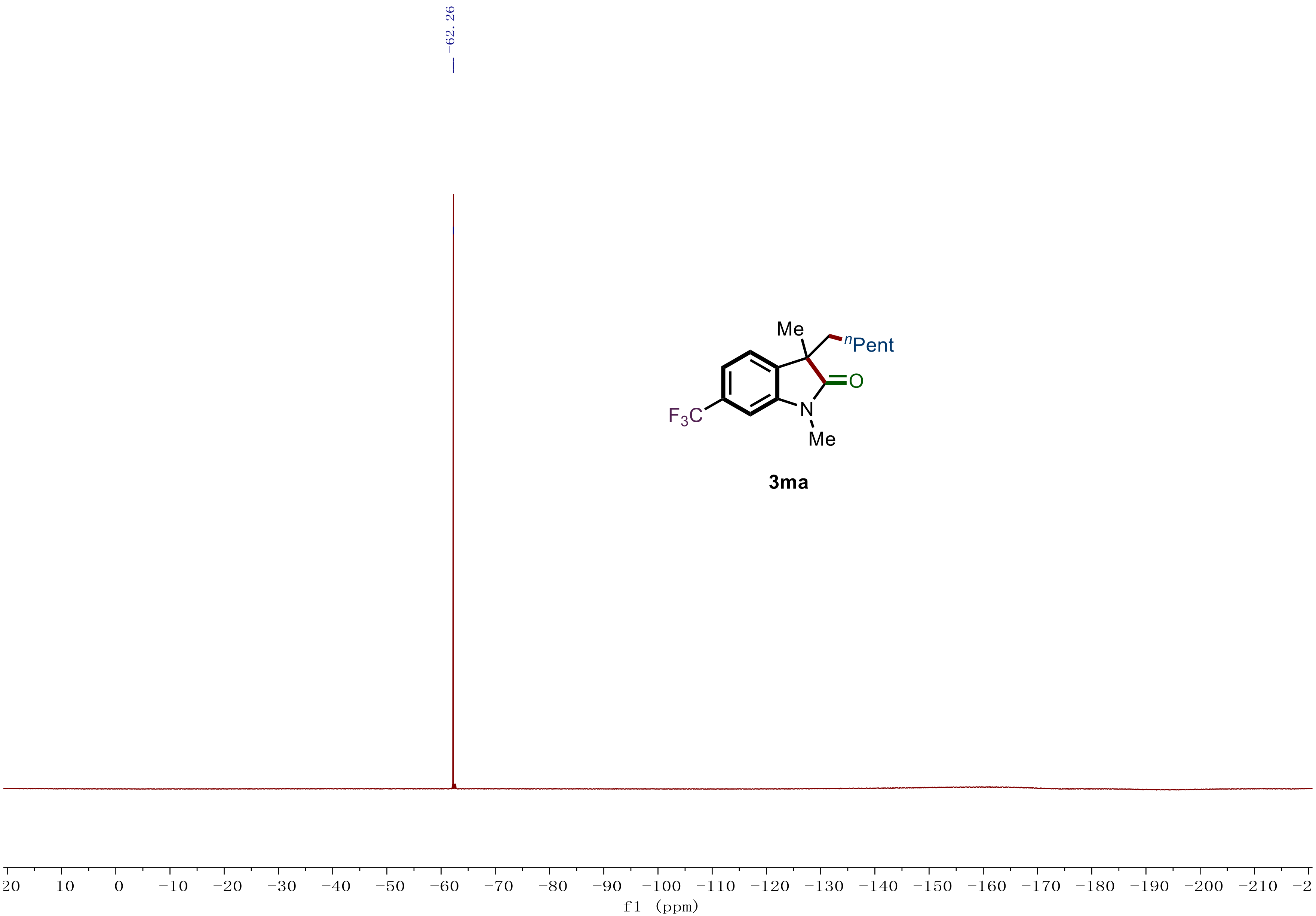

Supplementary Figure 129

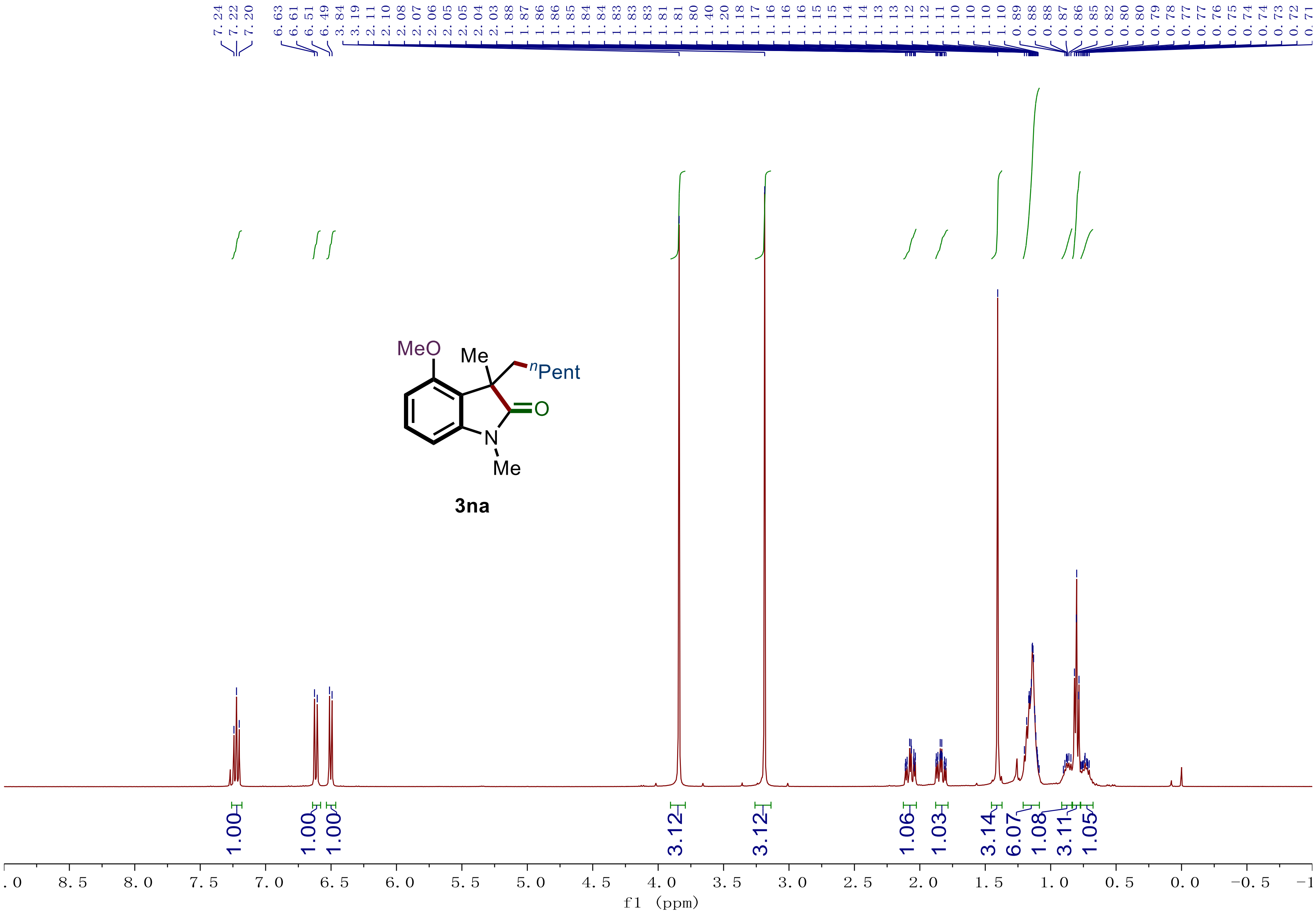

Supplementary Figure 130

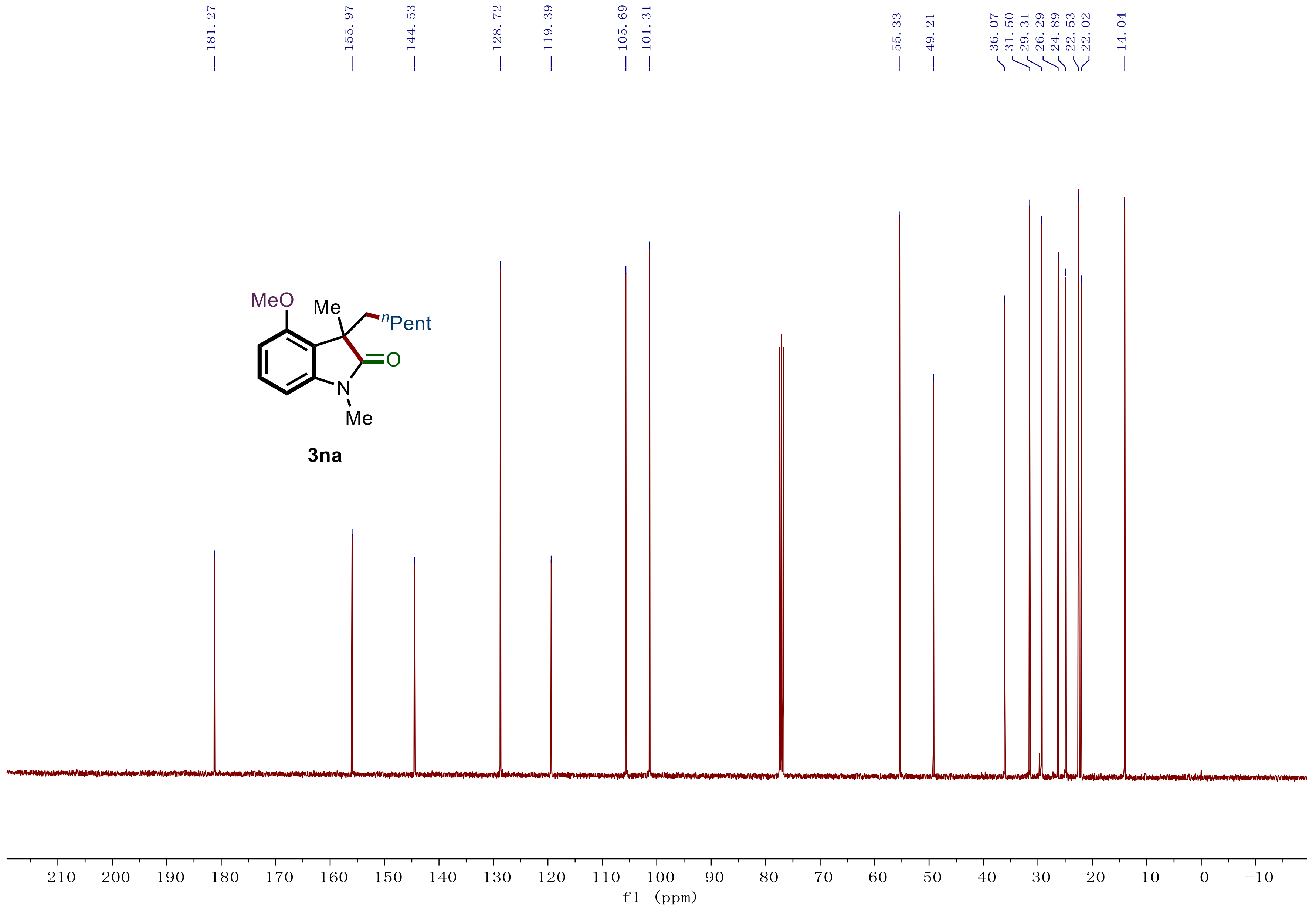

Supplementary Figure 131

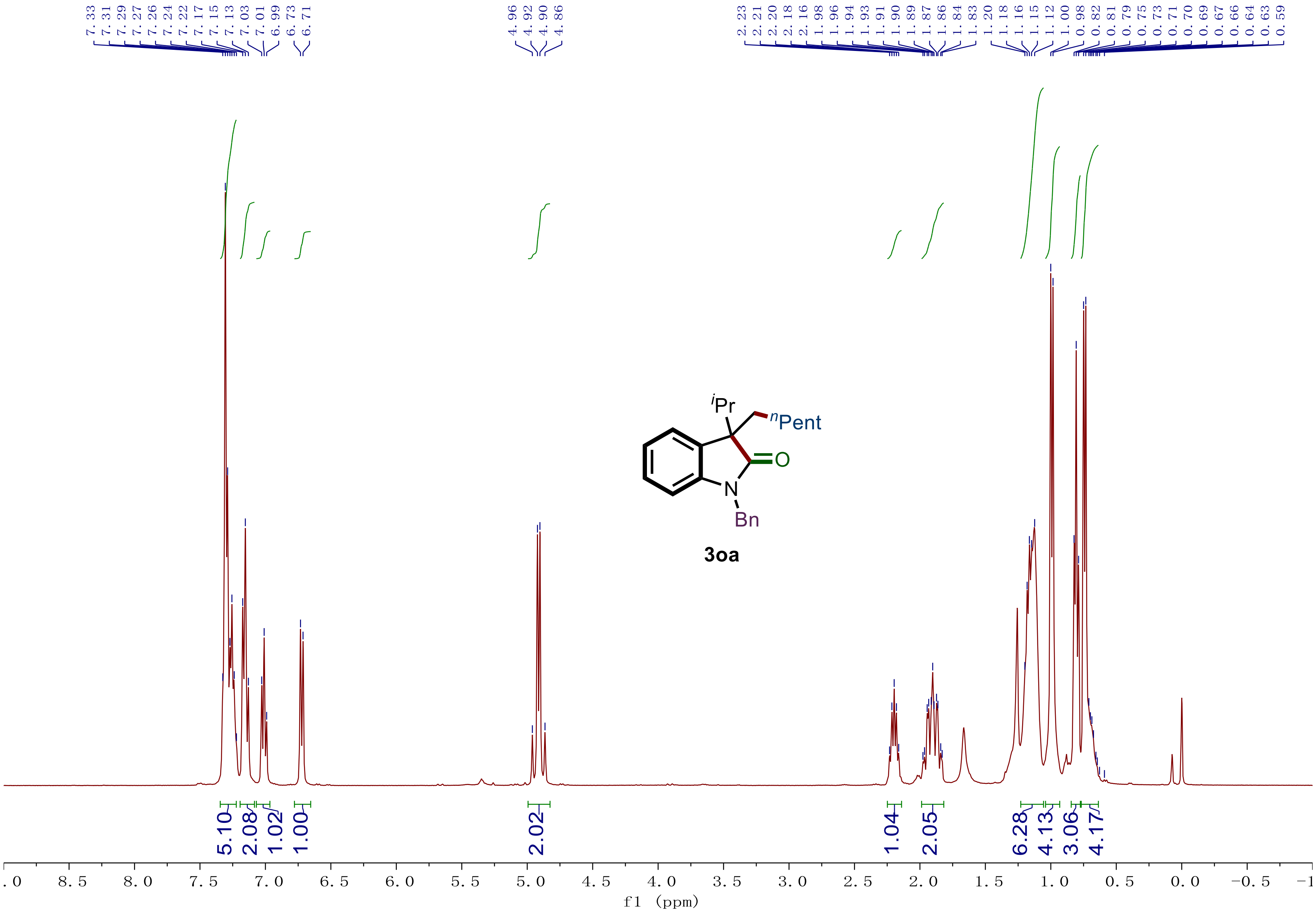

3oa

Supplementary Figure 132

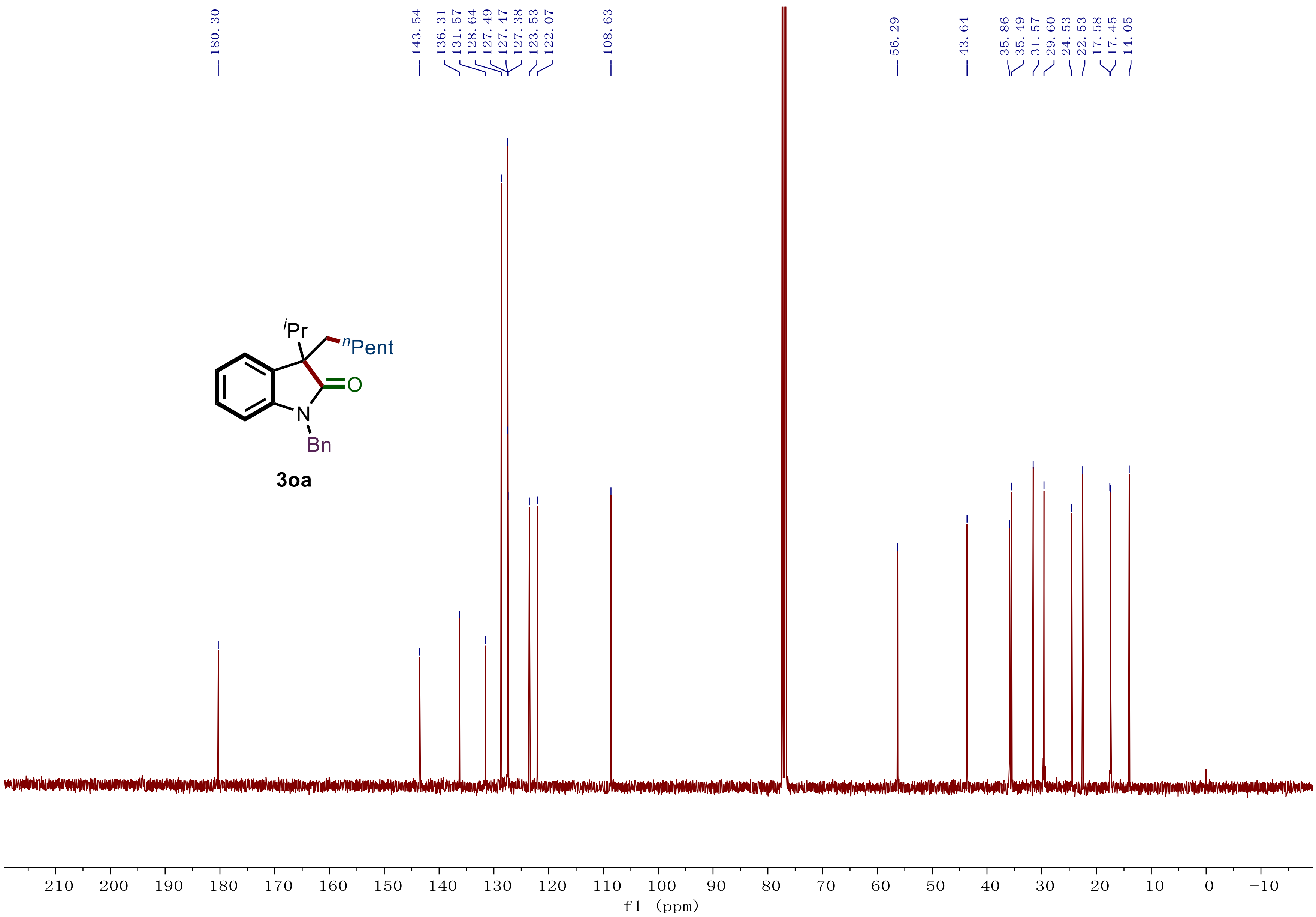

Supplementary Figure 133

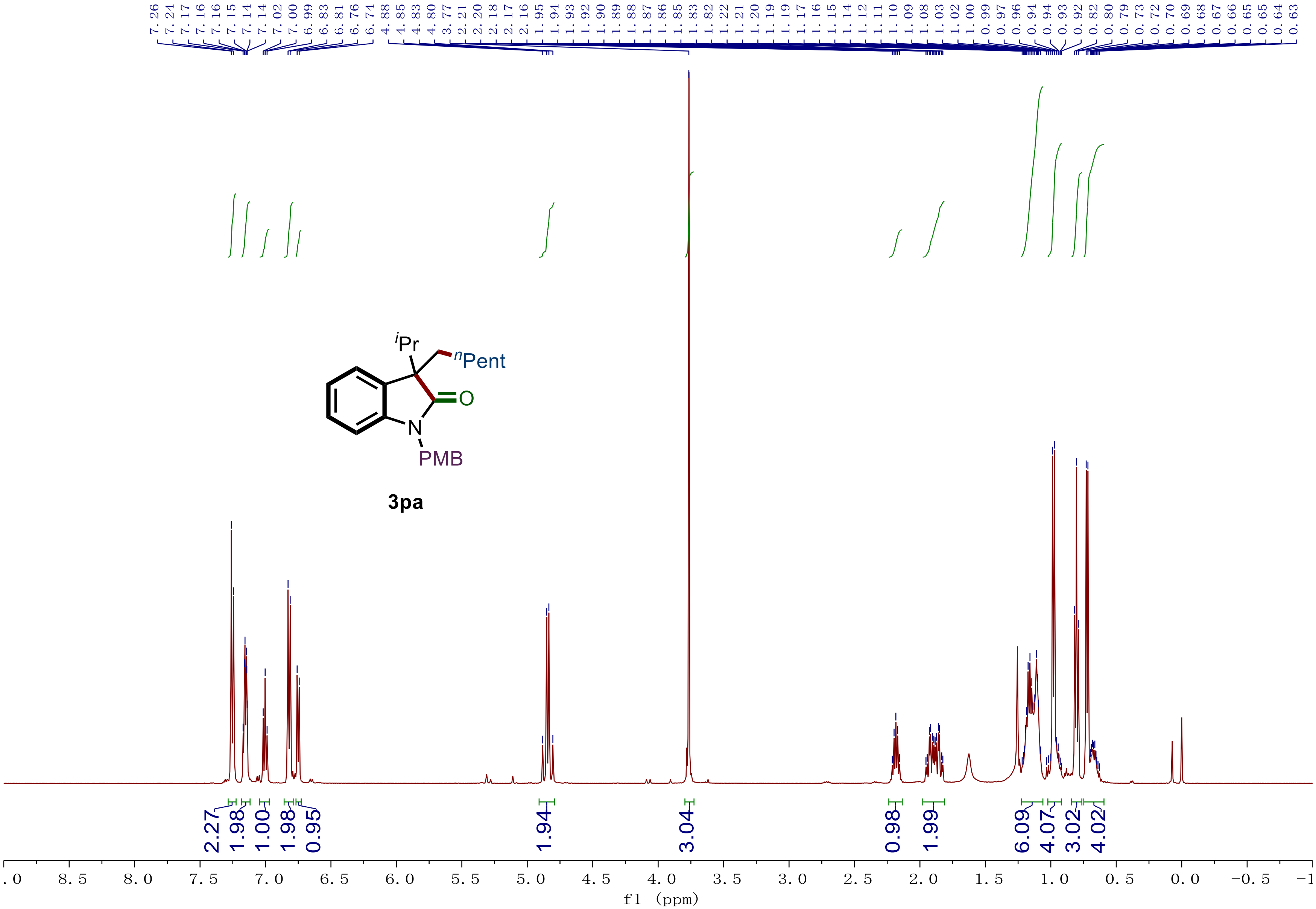

Supplementary Figure 134

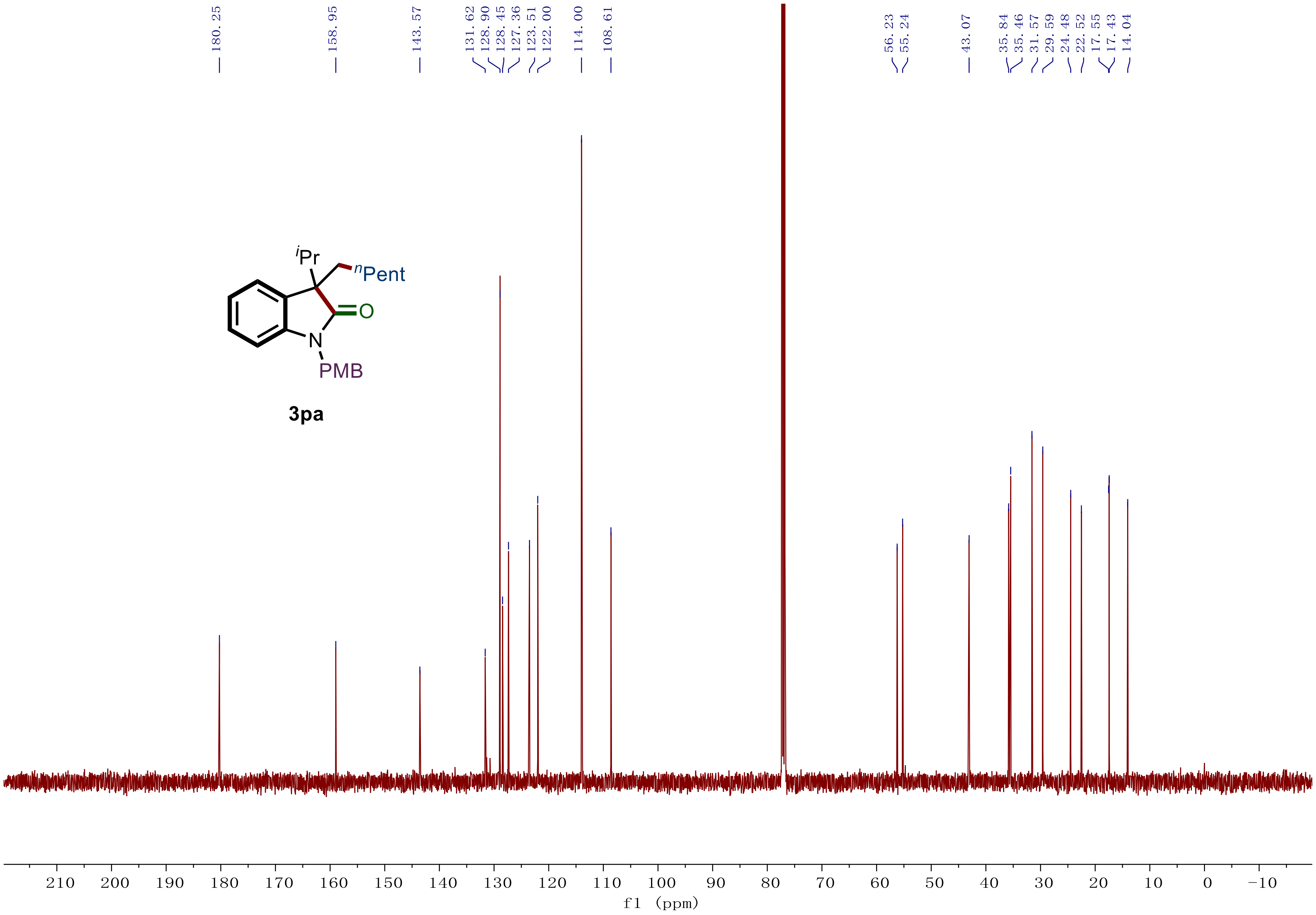

Supplementary Figure 135

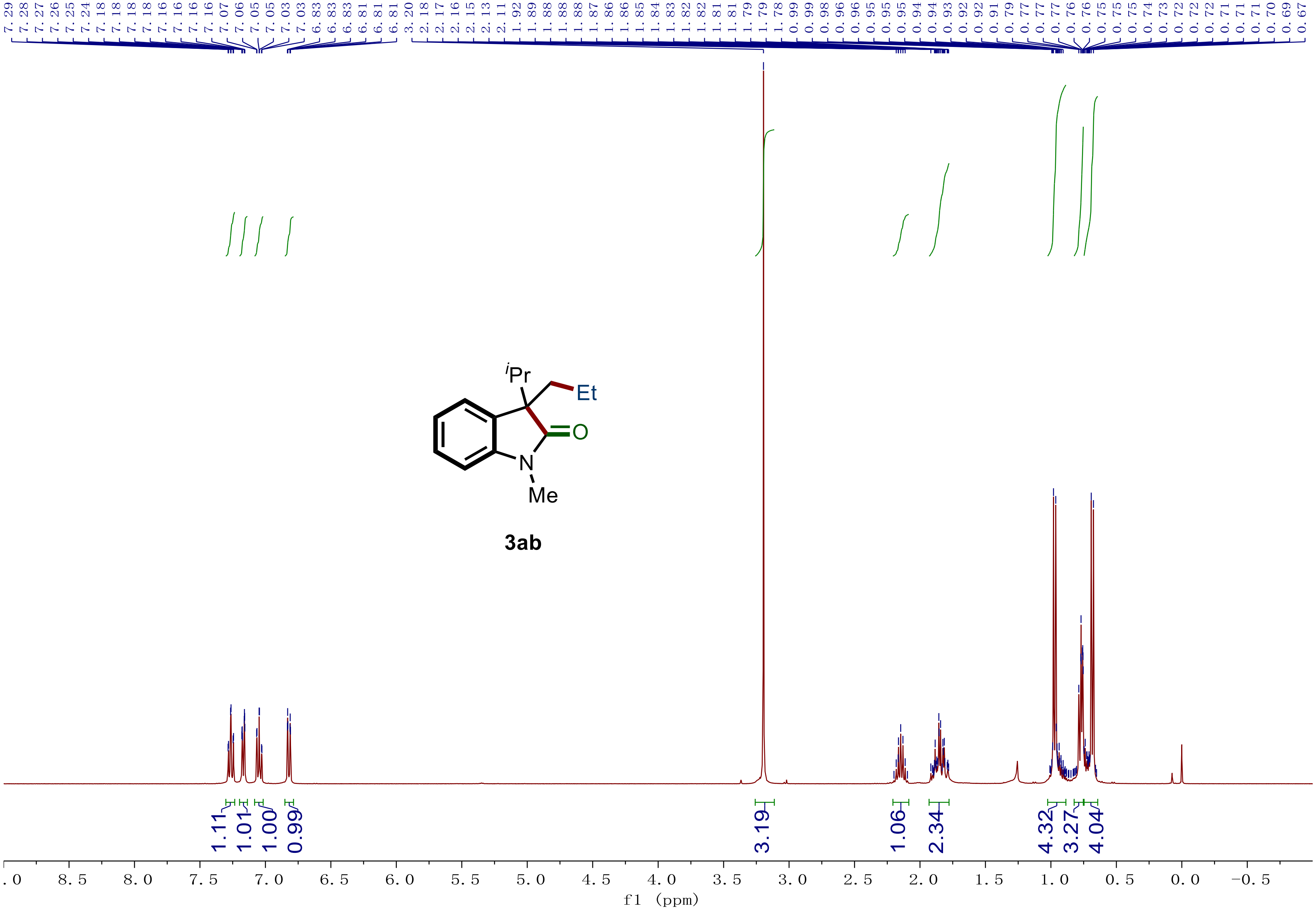

Supplementary Figure 135

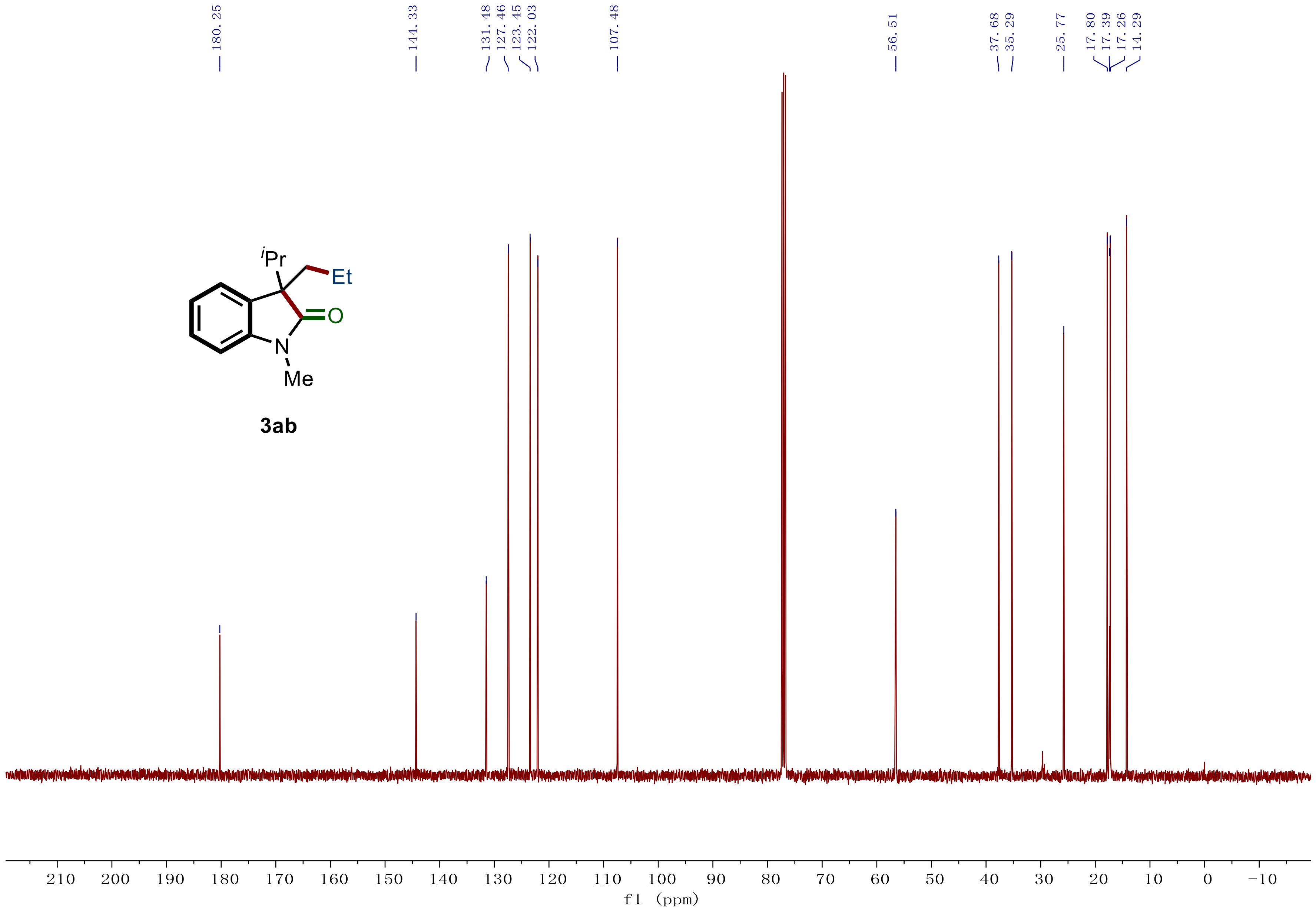

Supplementary Figure 136

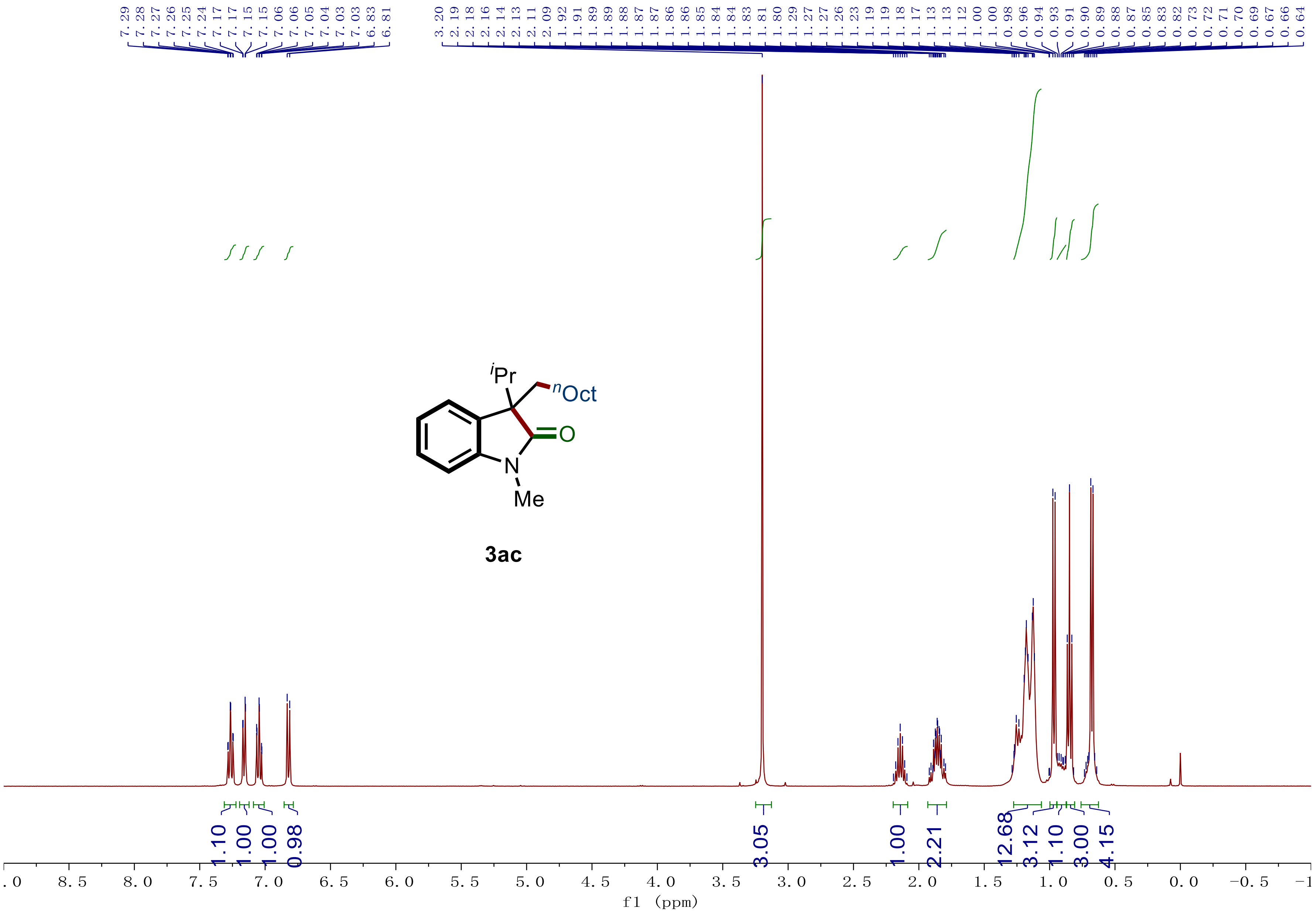

Supplementary Figure 137

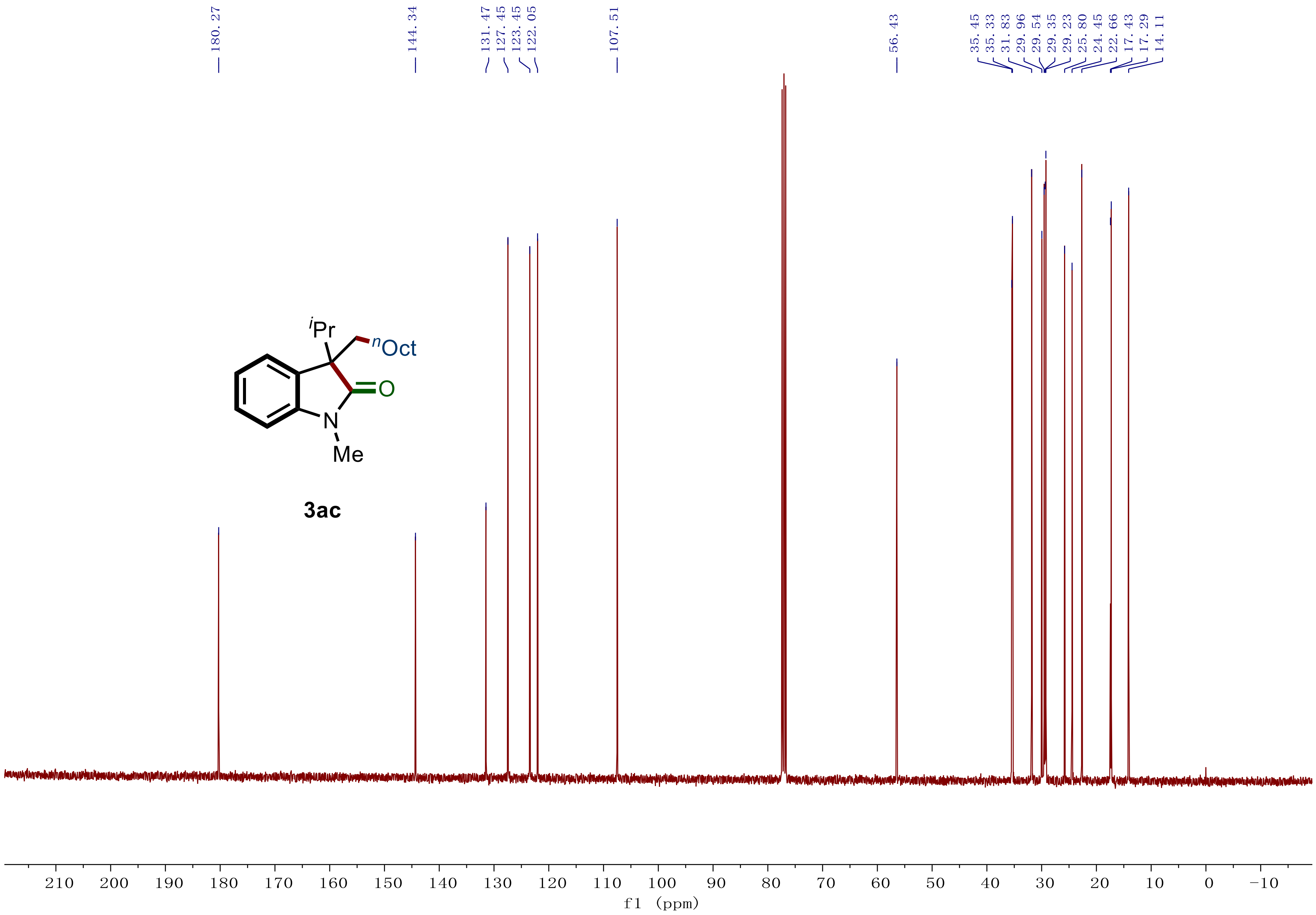

Supplementary Figure 138

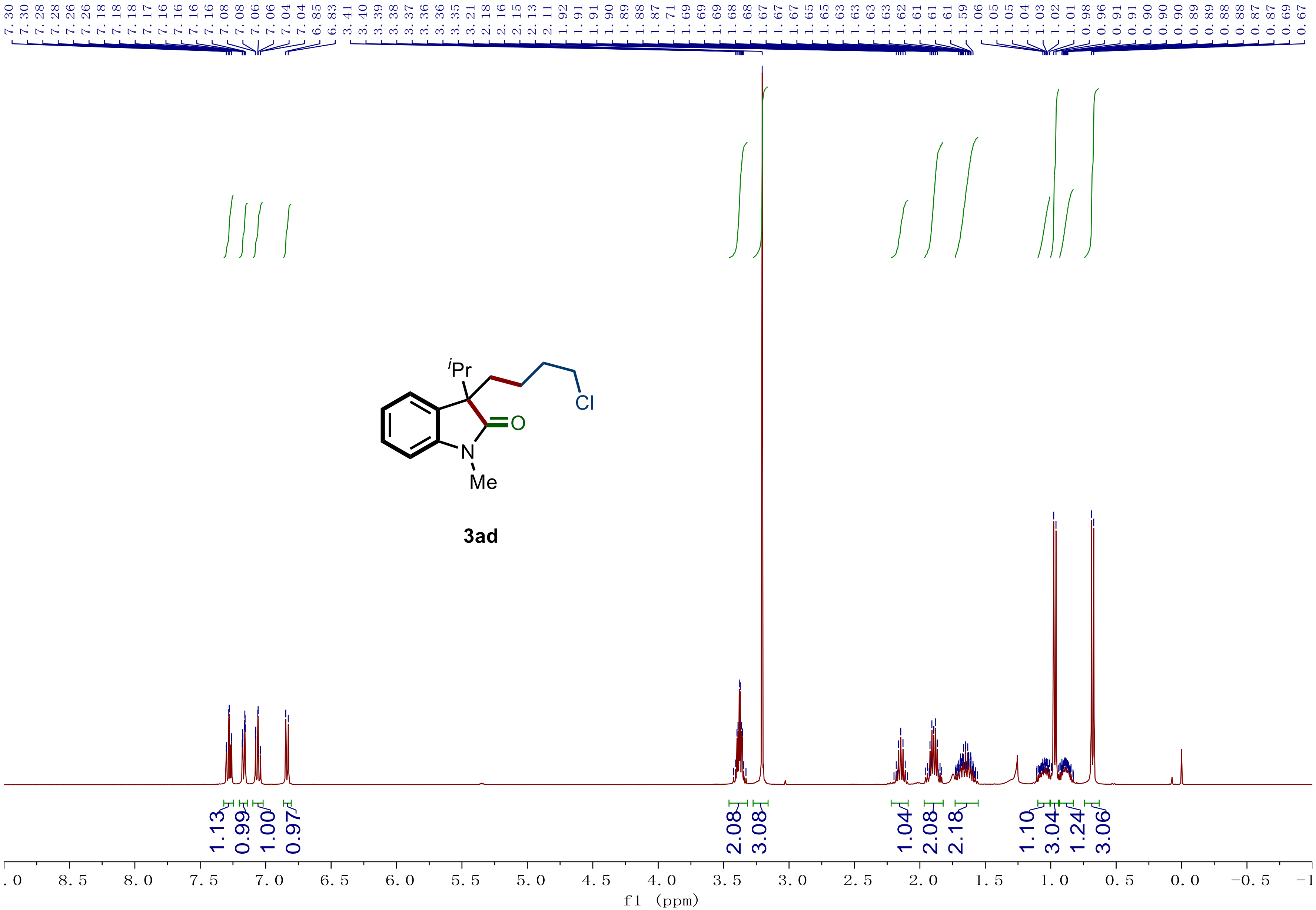

Supplementary Figure 139

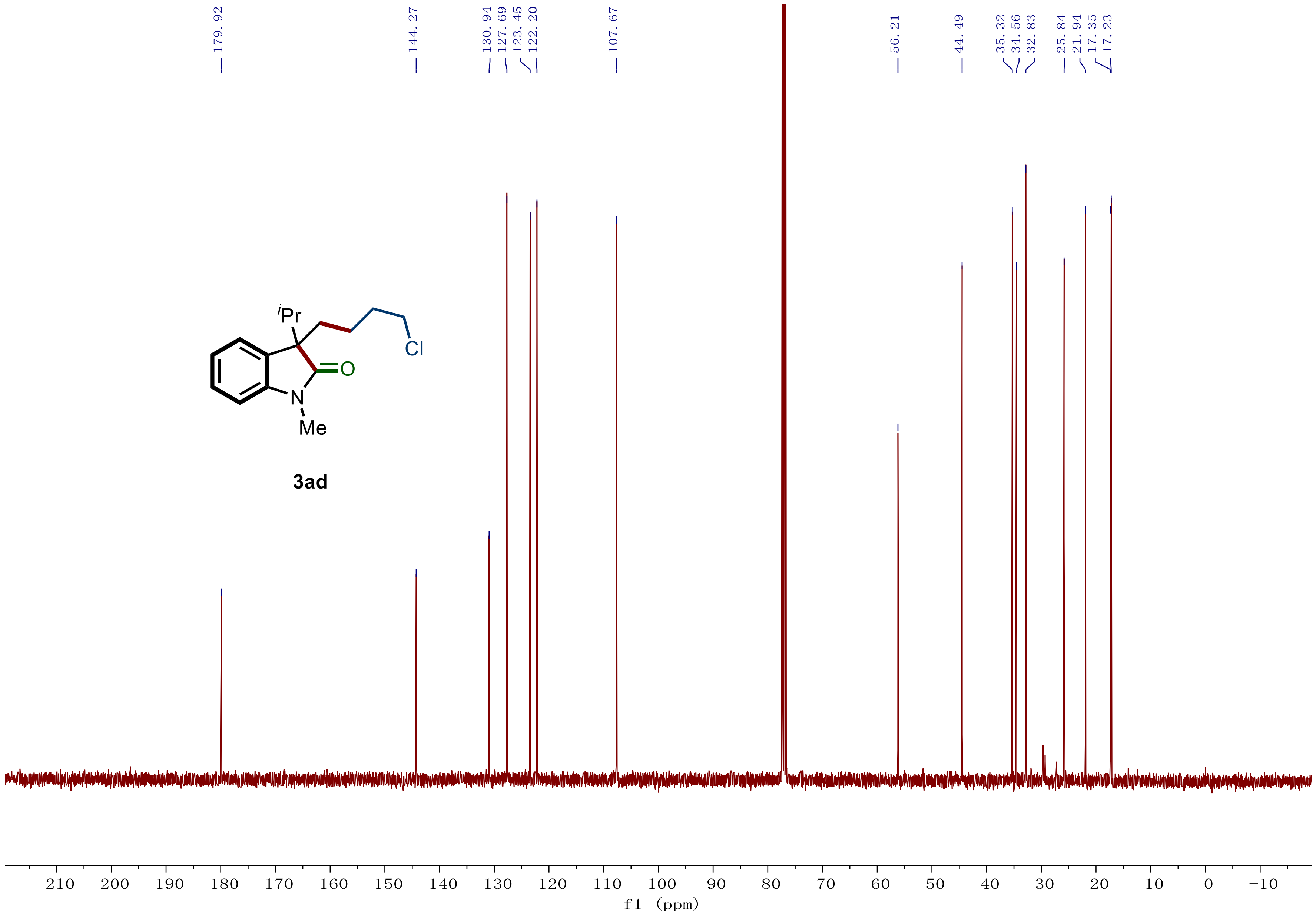

Supplementary Figure 140

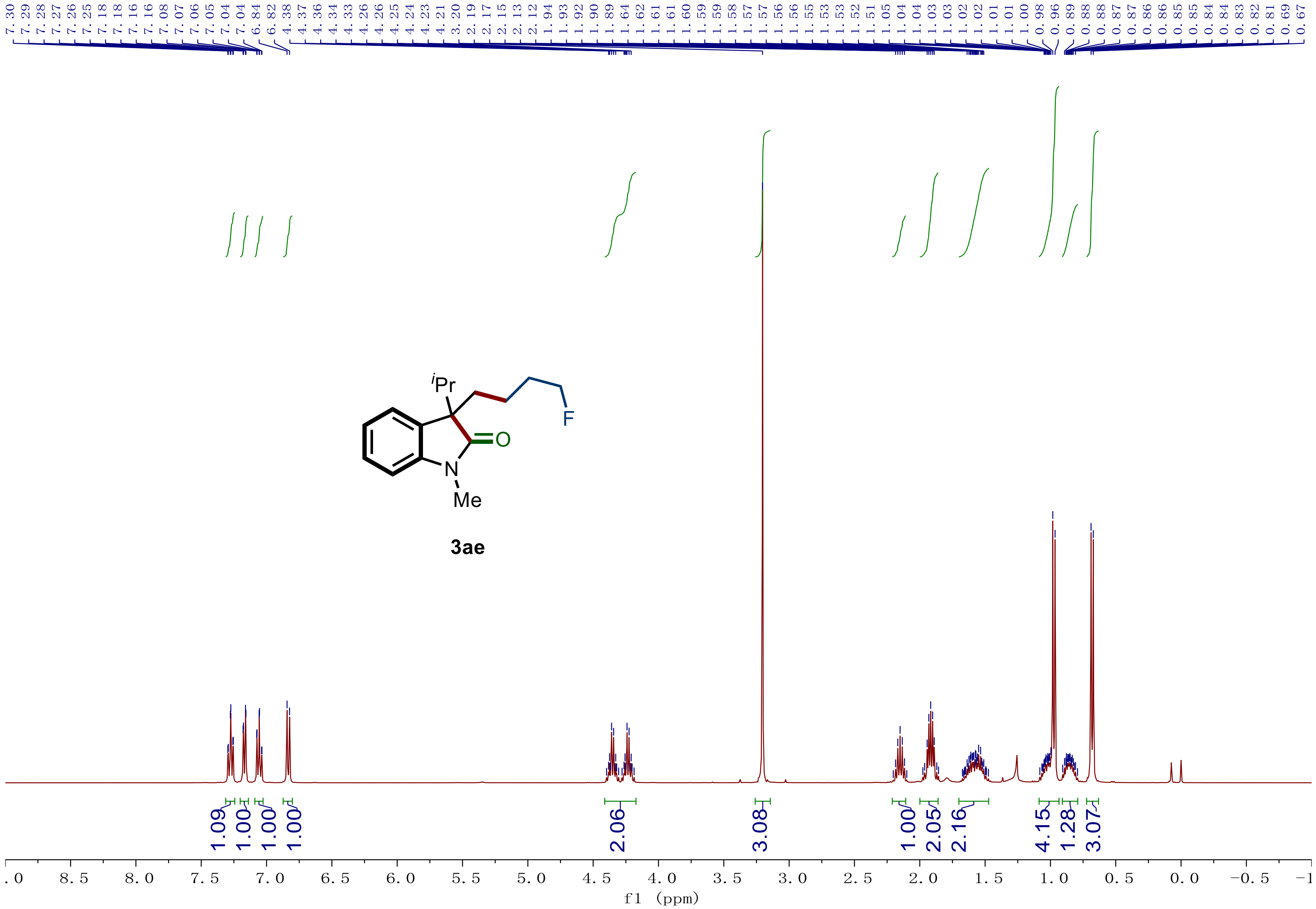

Supplementary Figure 141

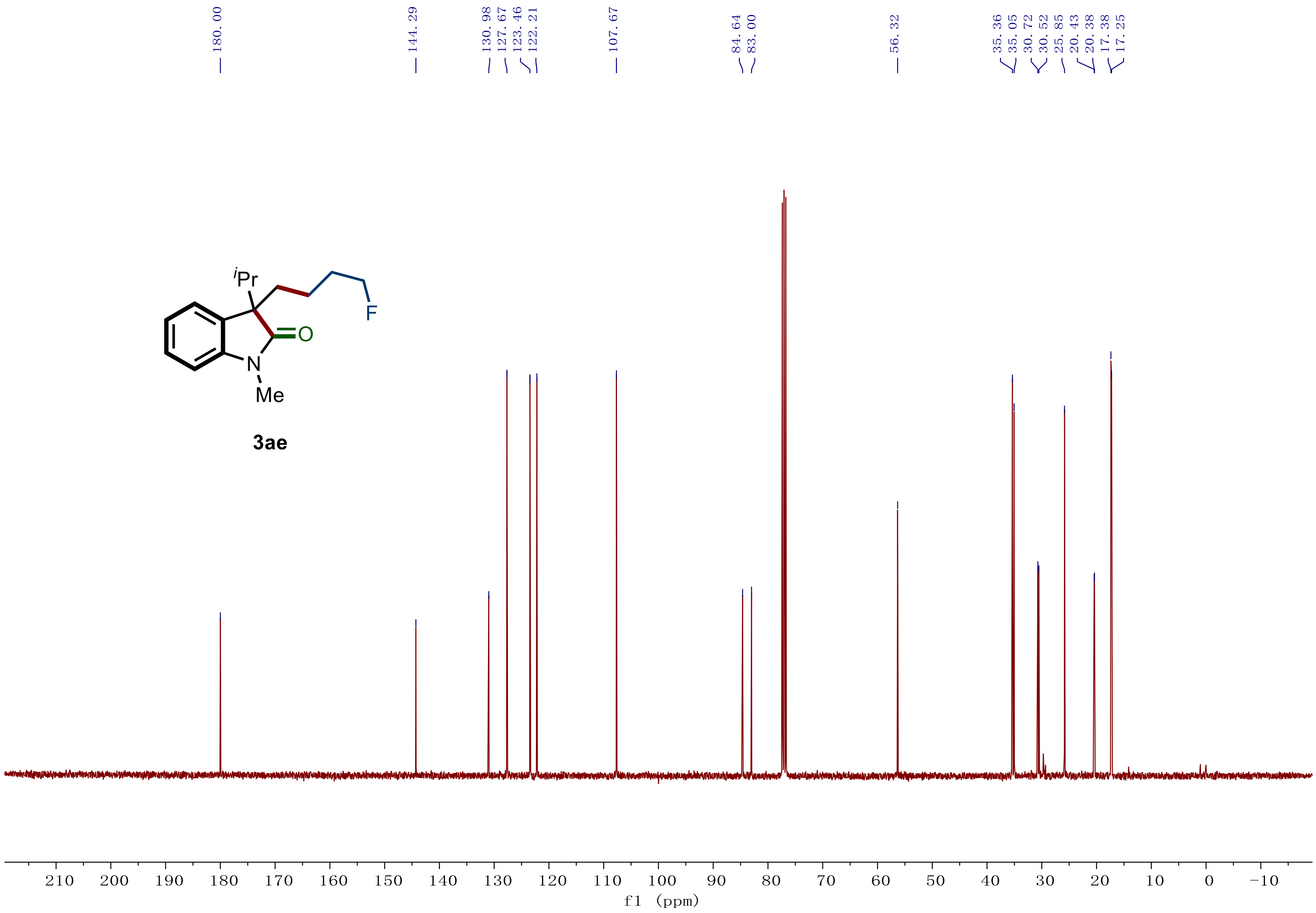

Supplementary Figure 142

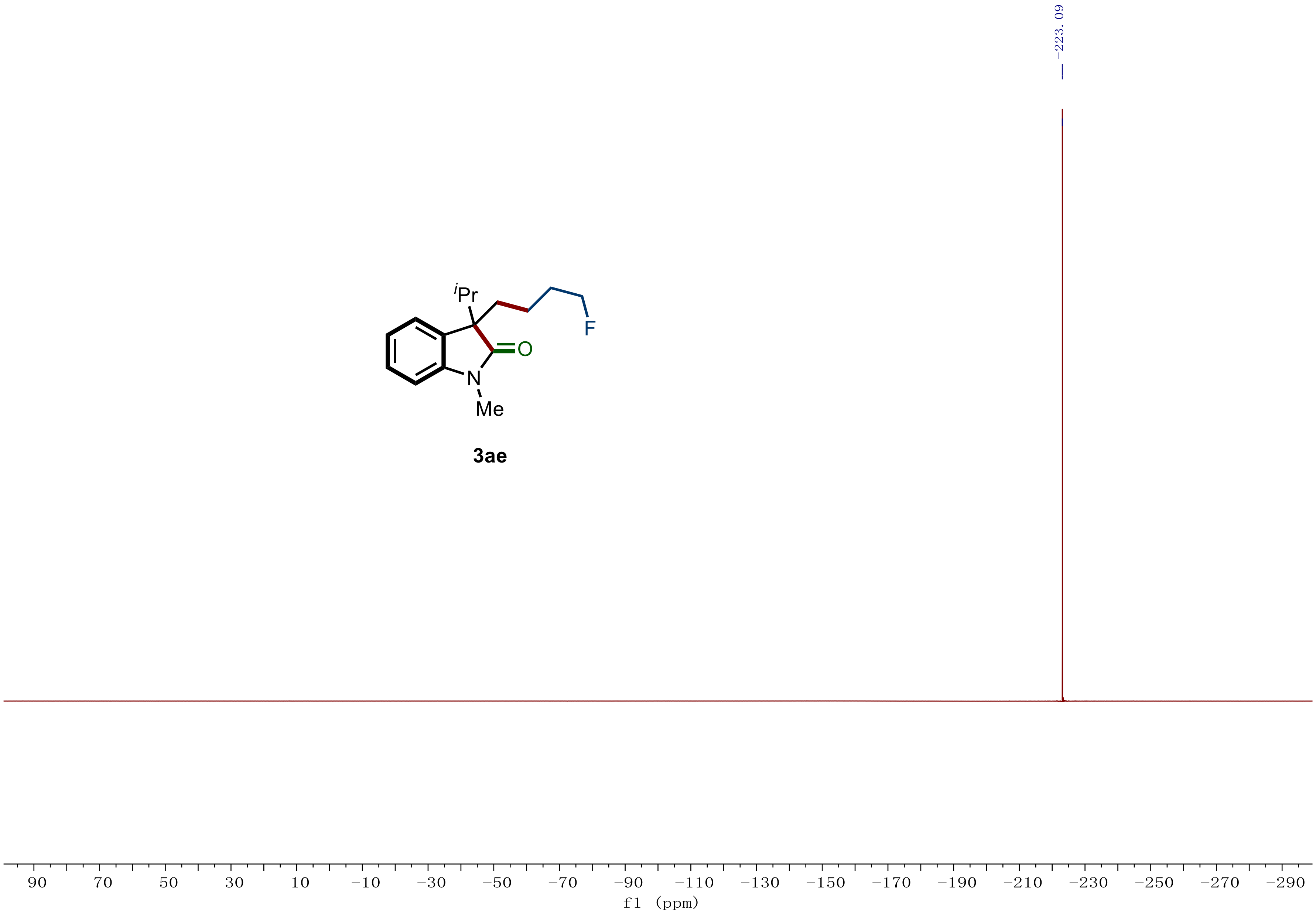

Supplementary Figure 143

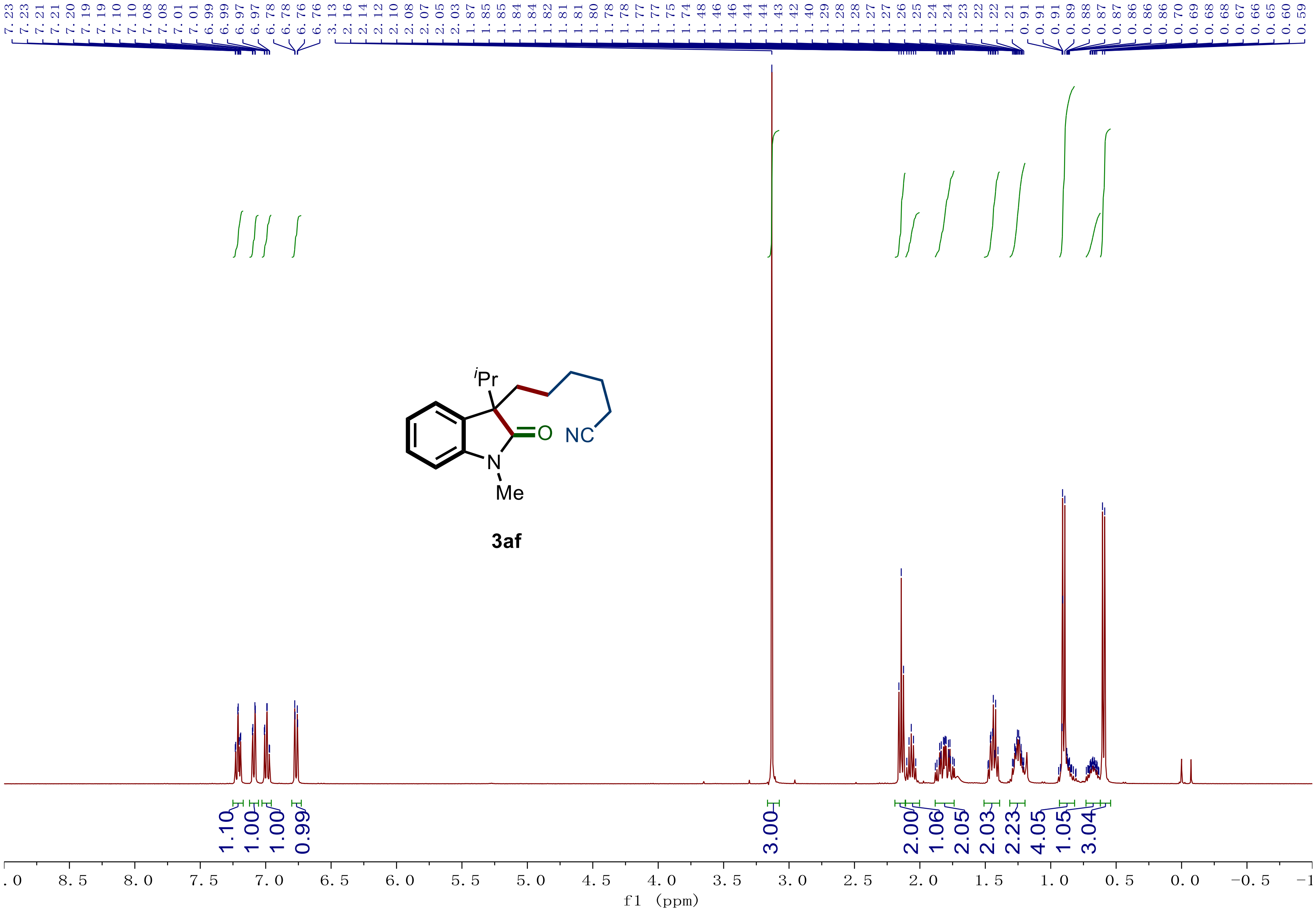

Supplementary Figure 144

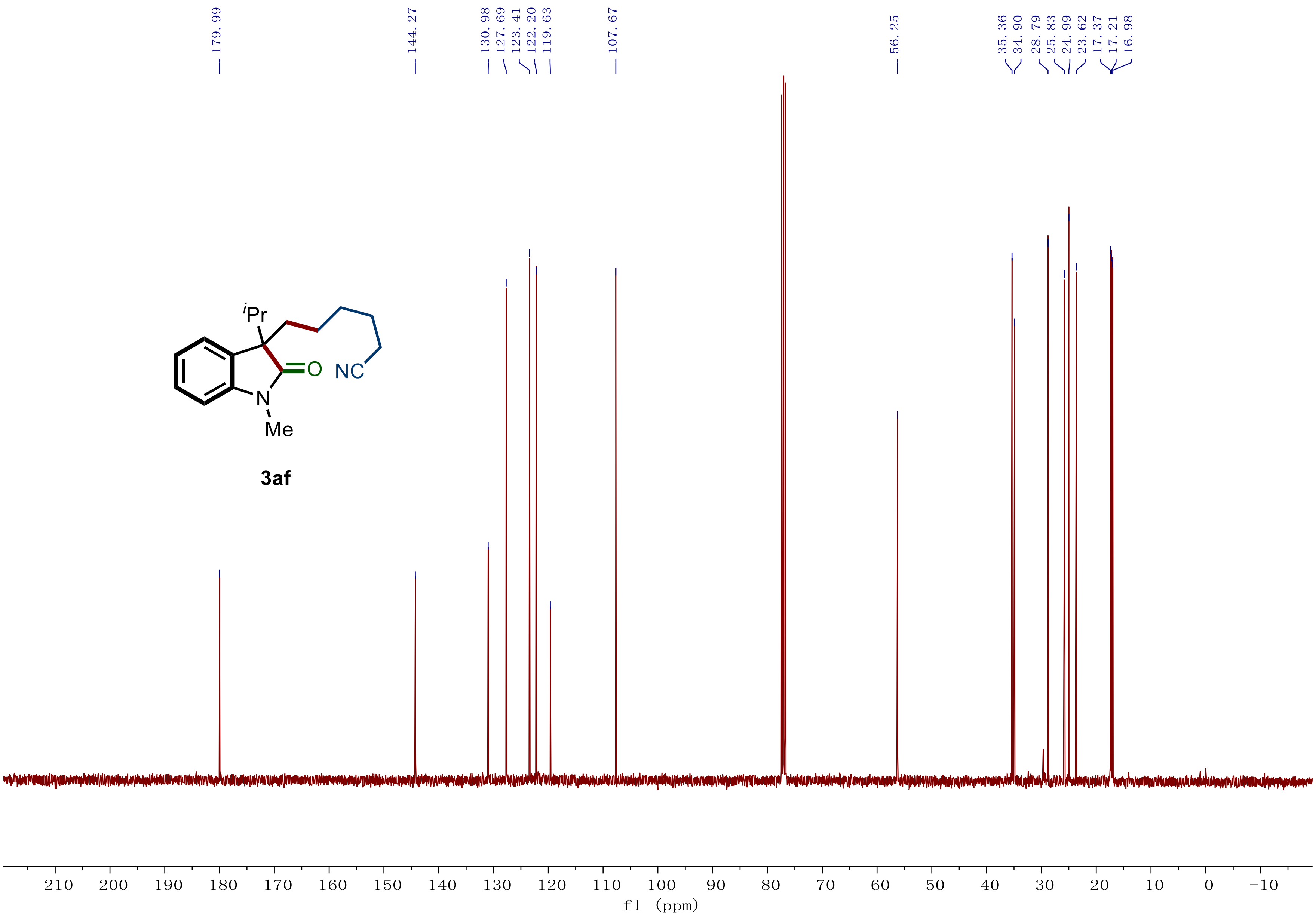

Supplementary Figure 145

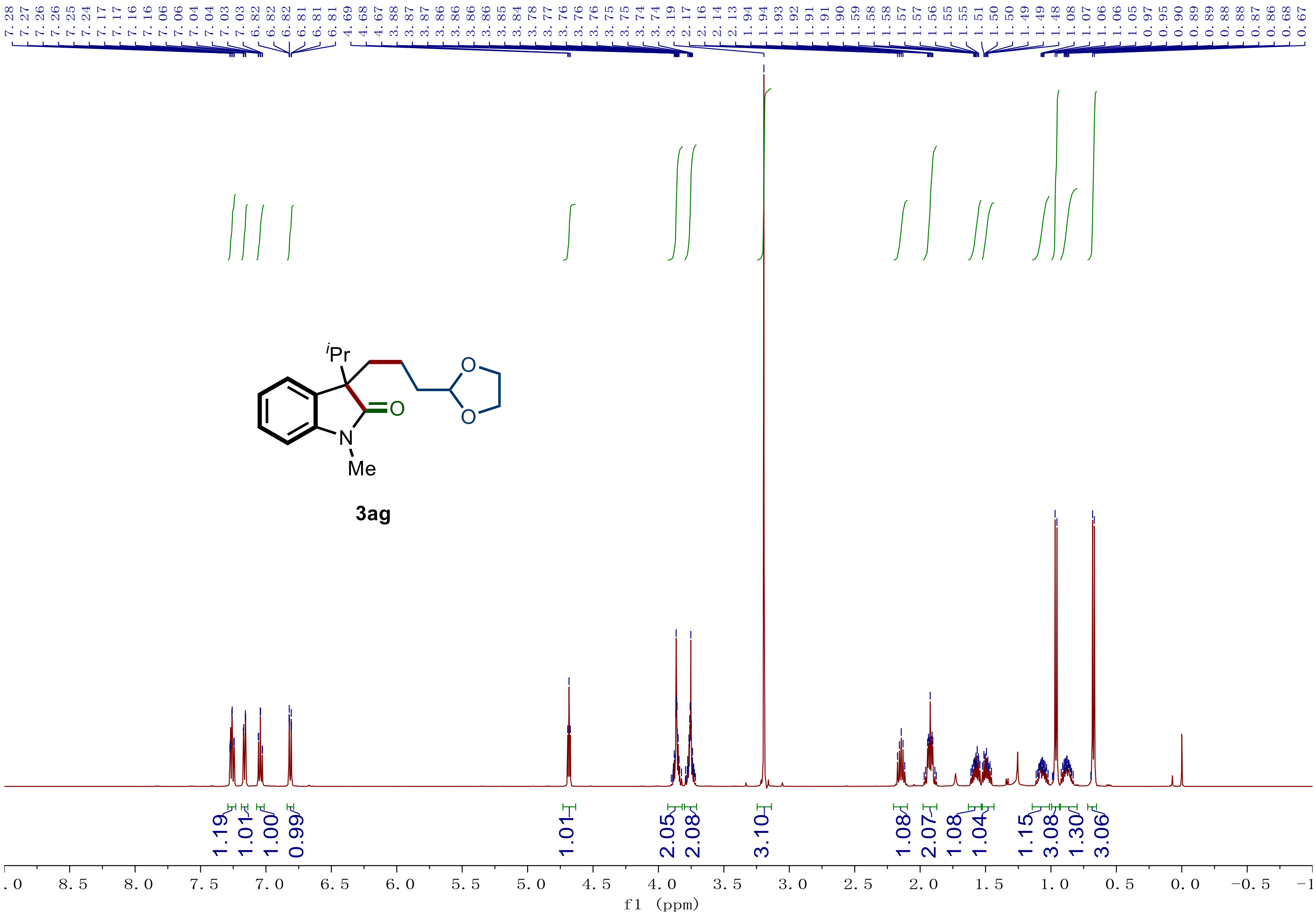

Supplementary Figure 146

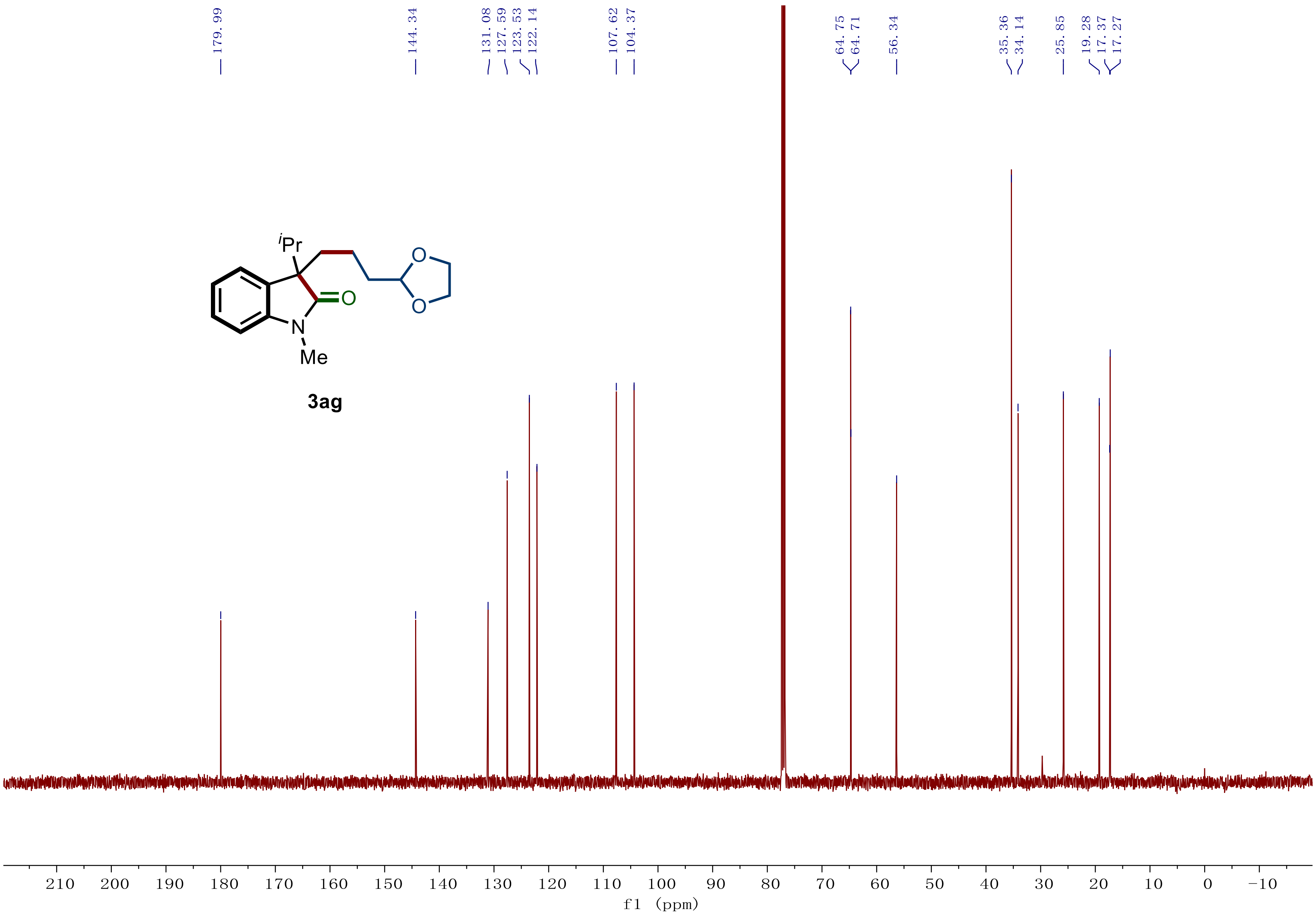

Supplementary Figure 147

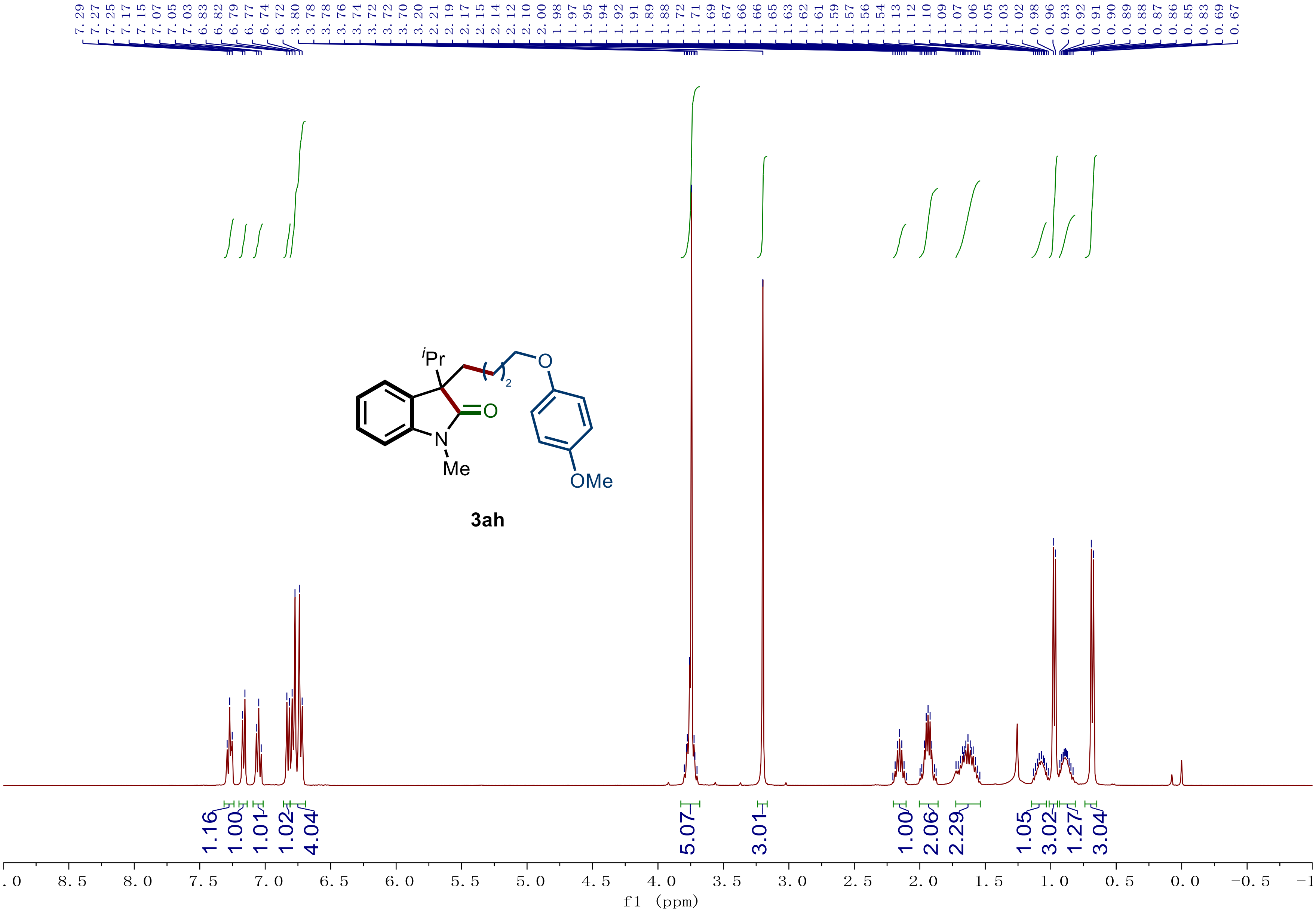

Supplementary Figure 148

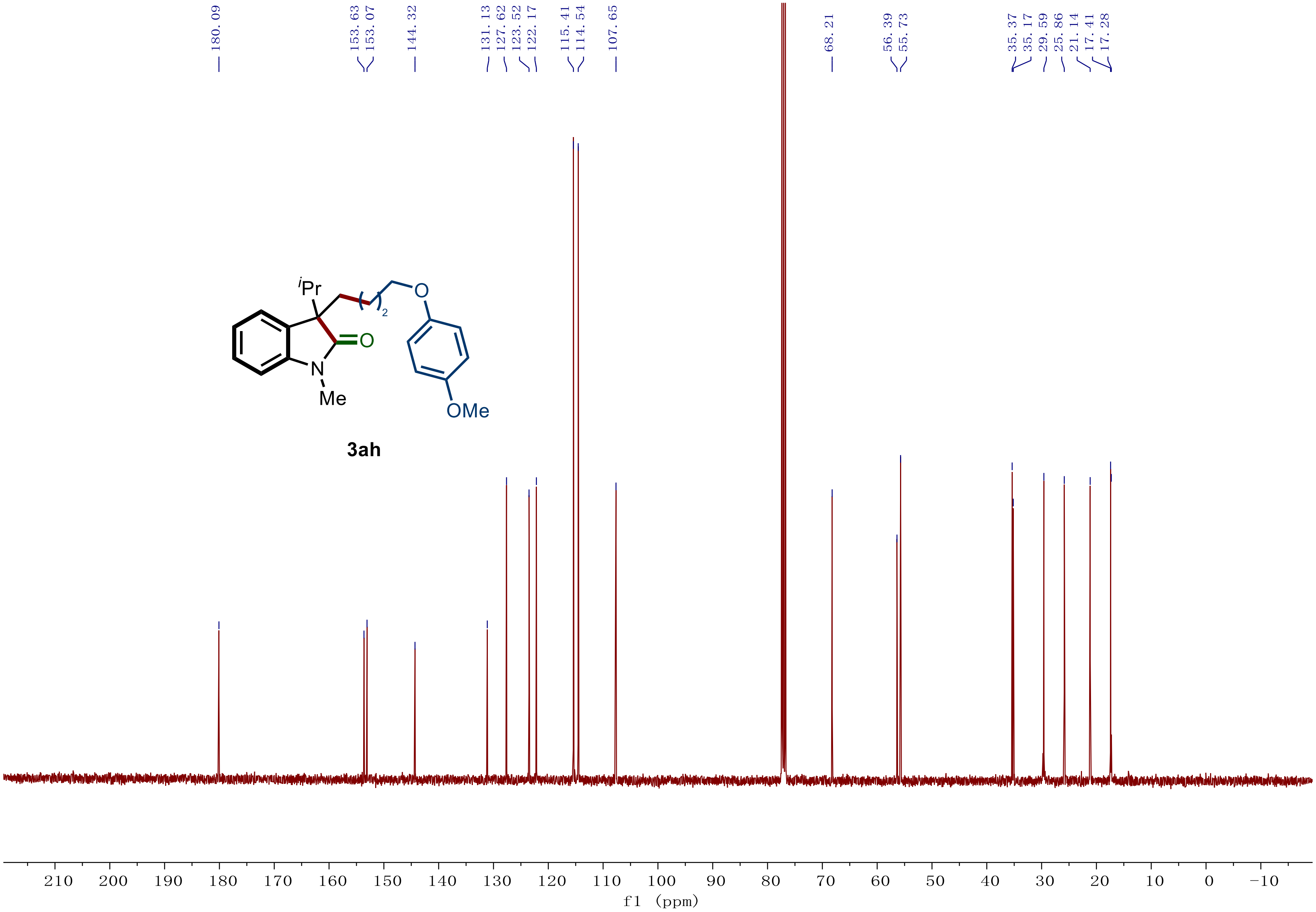

Supplementary Figure 149

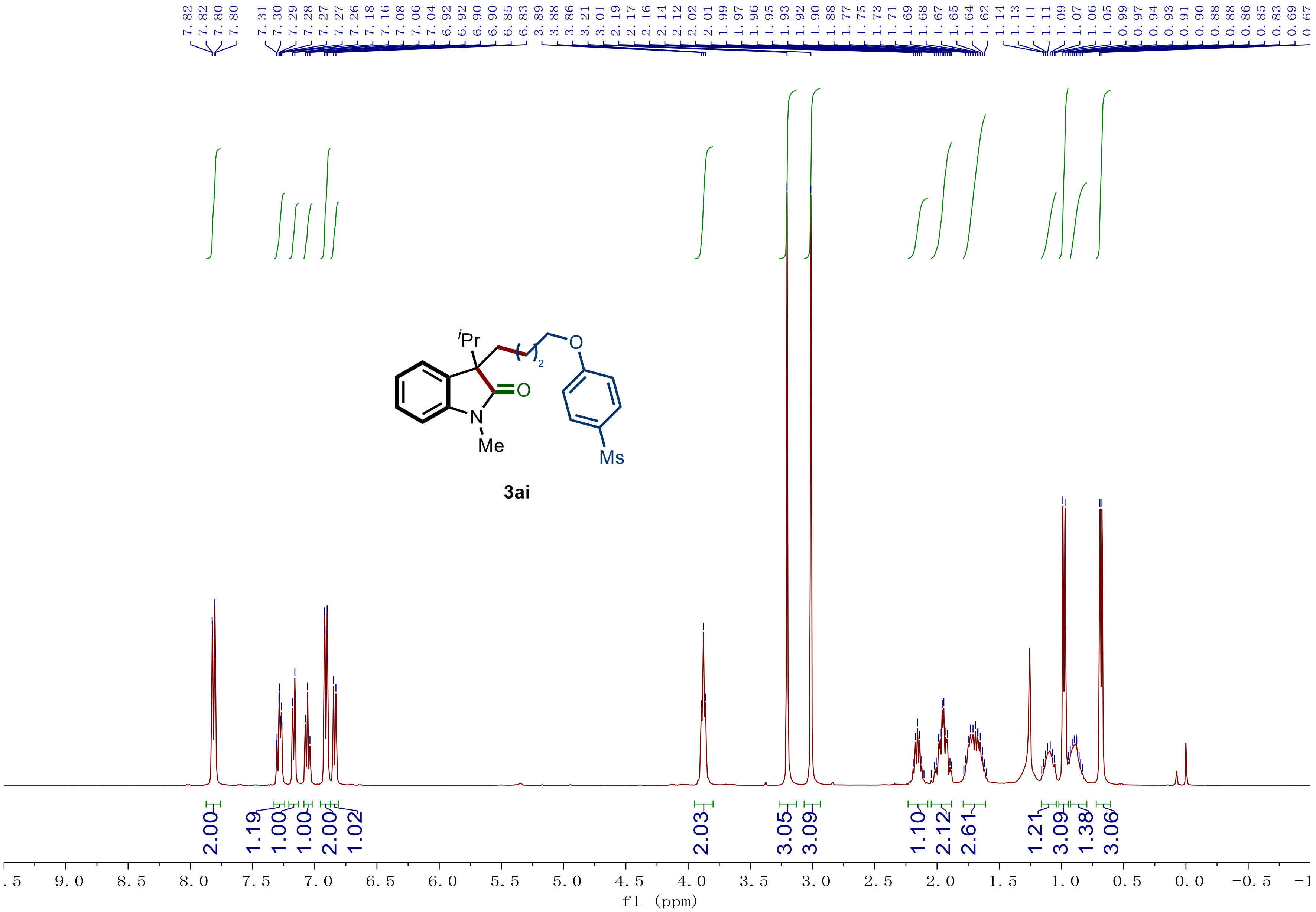

Supplementary Figure 150

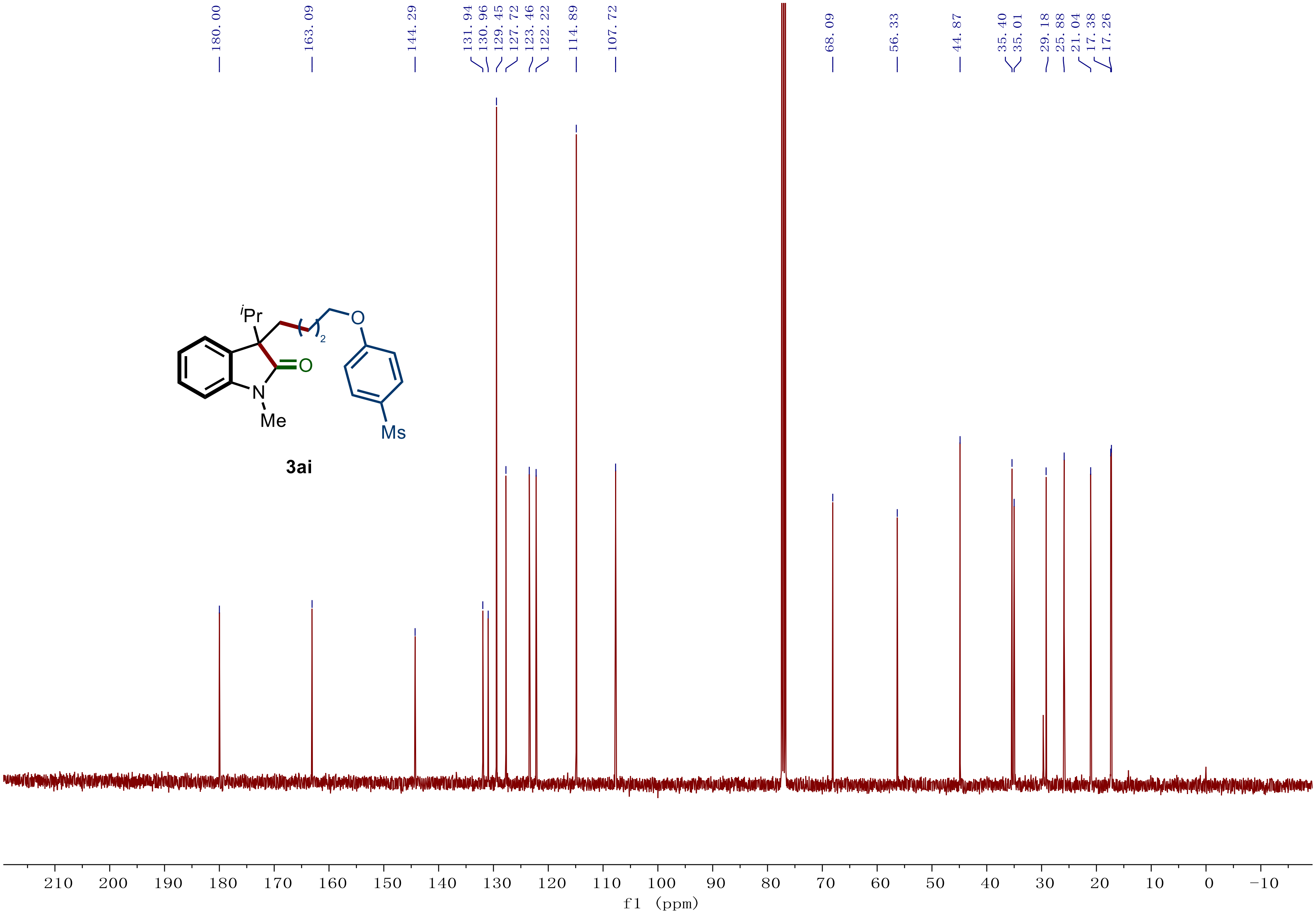

Supplementary Figure 151

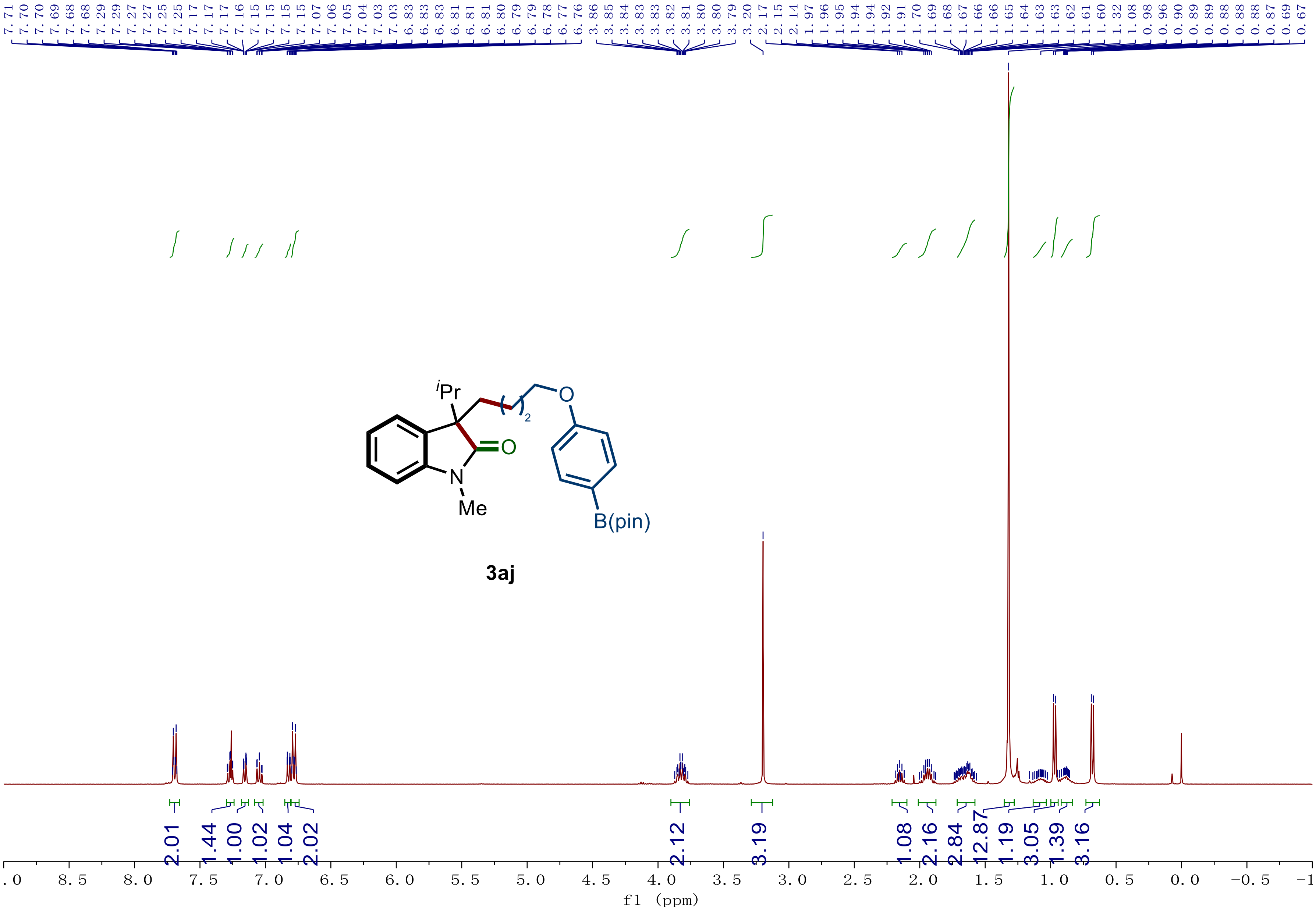

Supplementary Figure 152

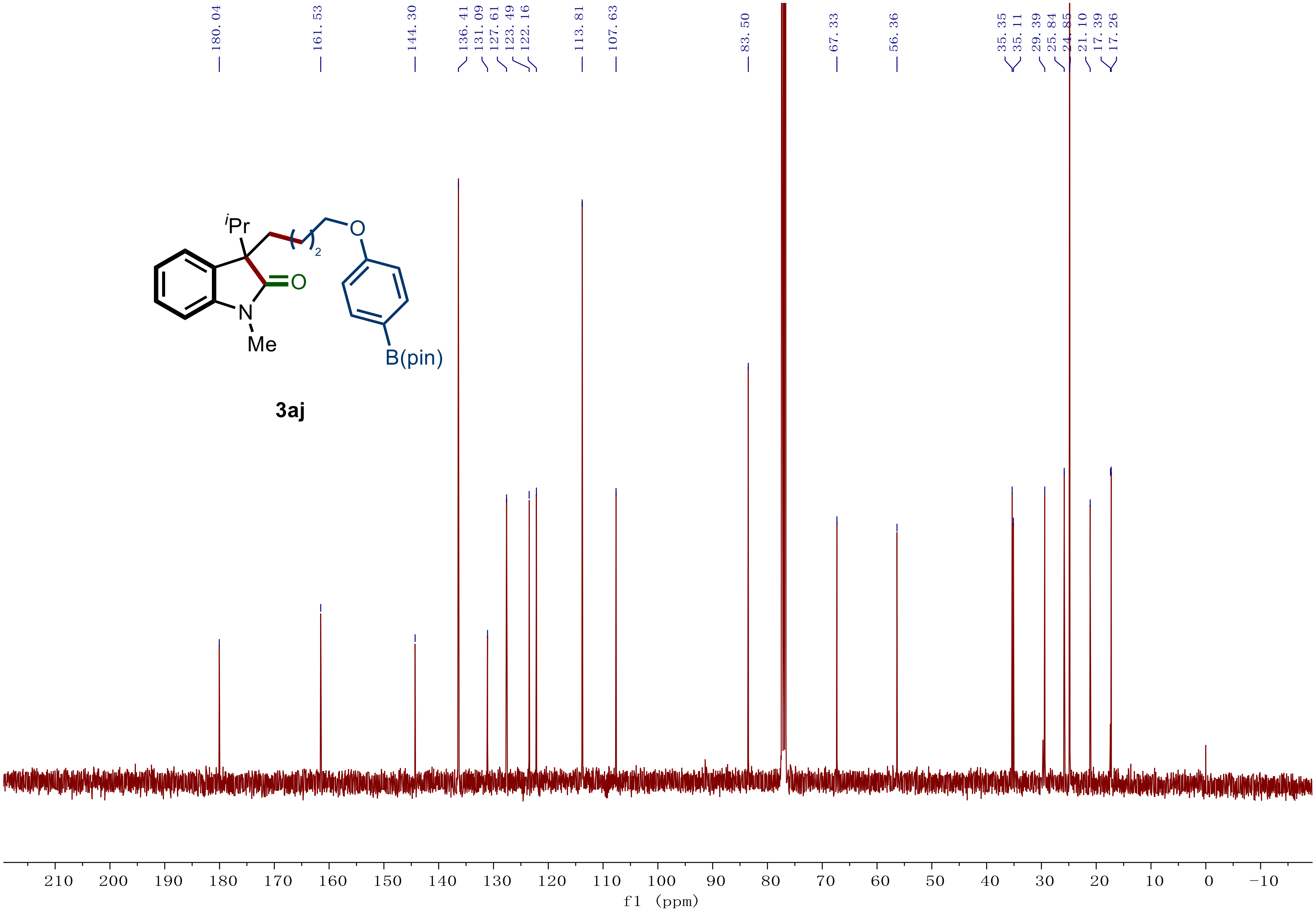

Supplementary Figure 153

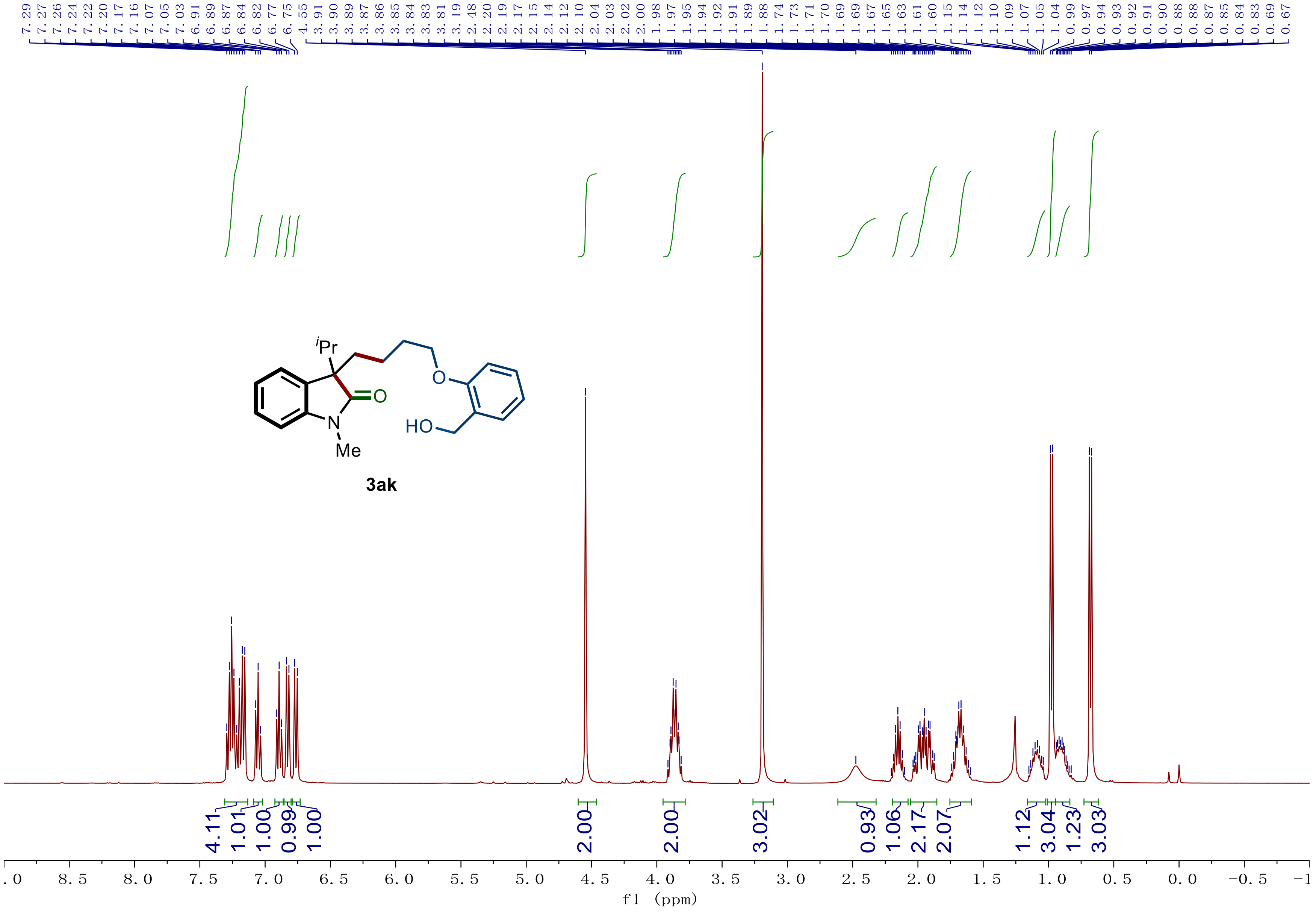

Supplementary Figure 154

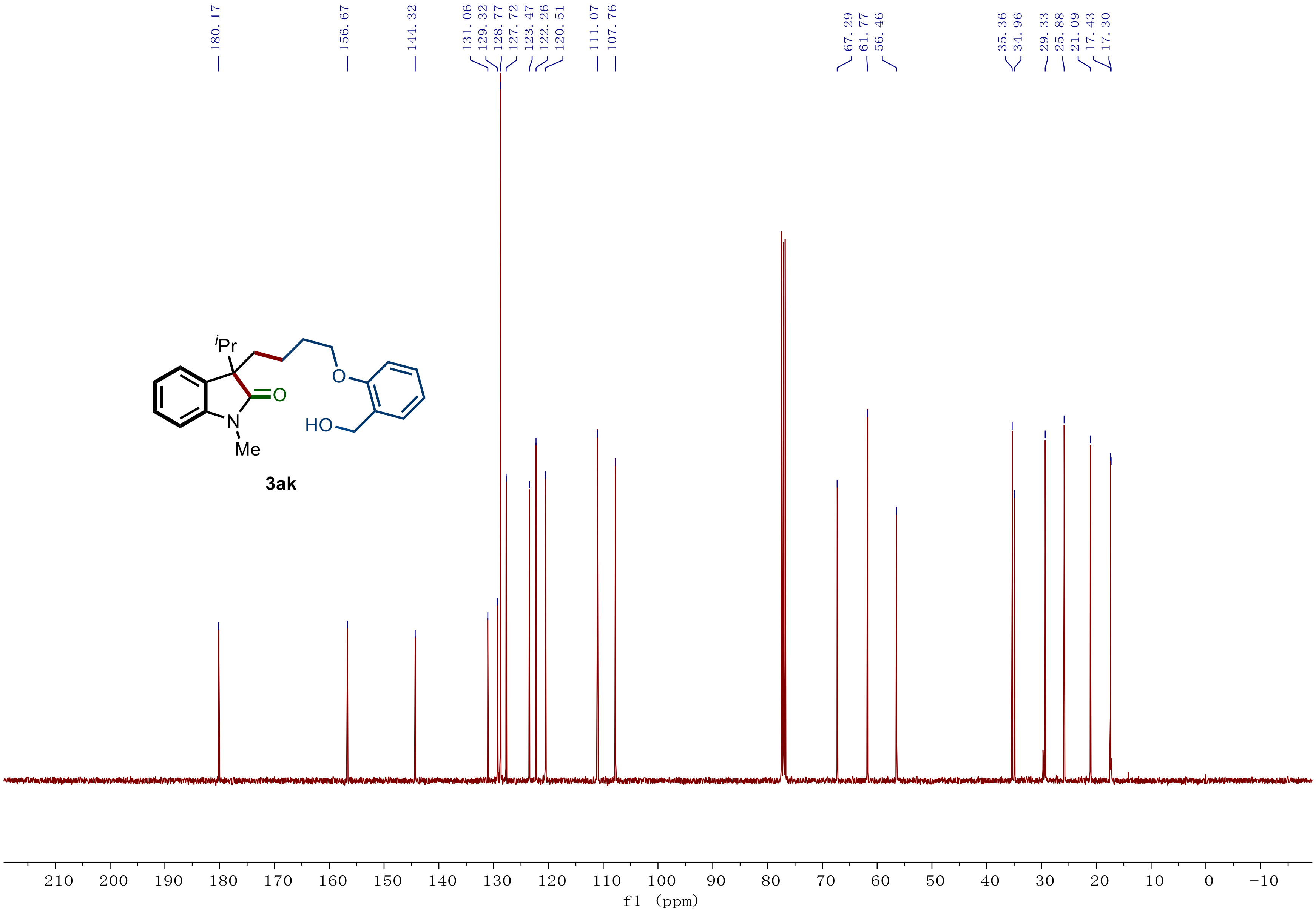

Supplementary Figure 155

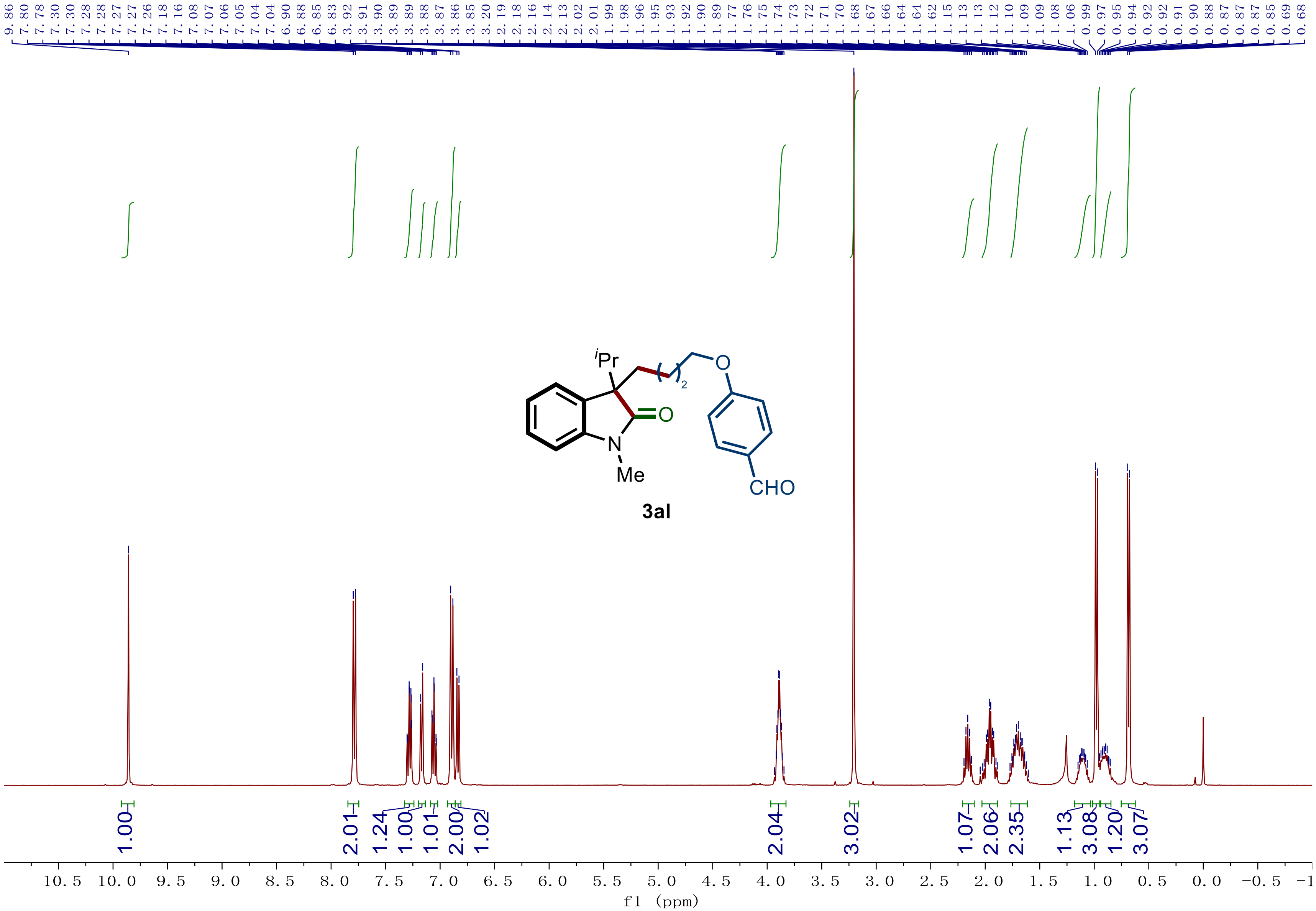

Supplementary Figure 156

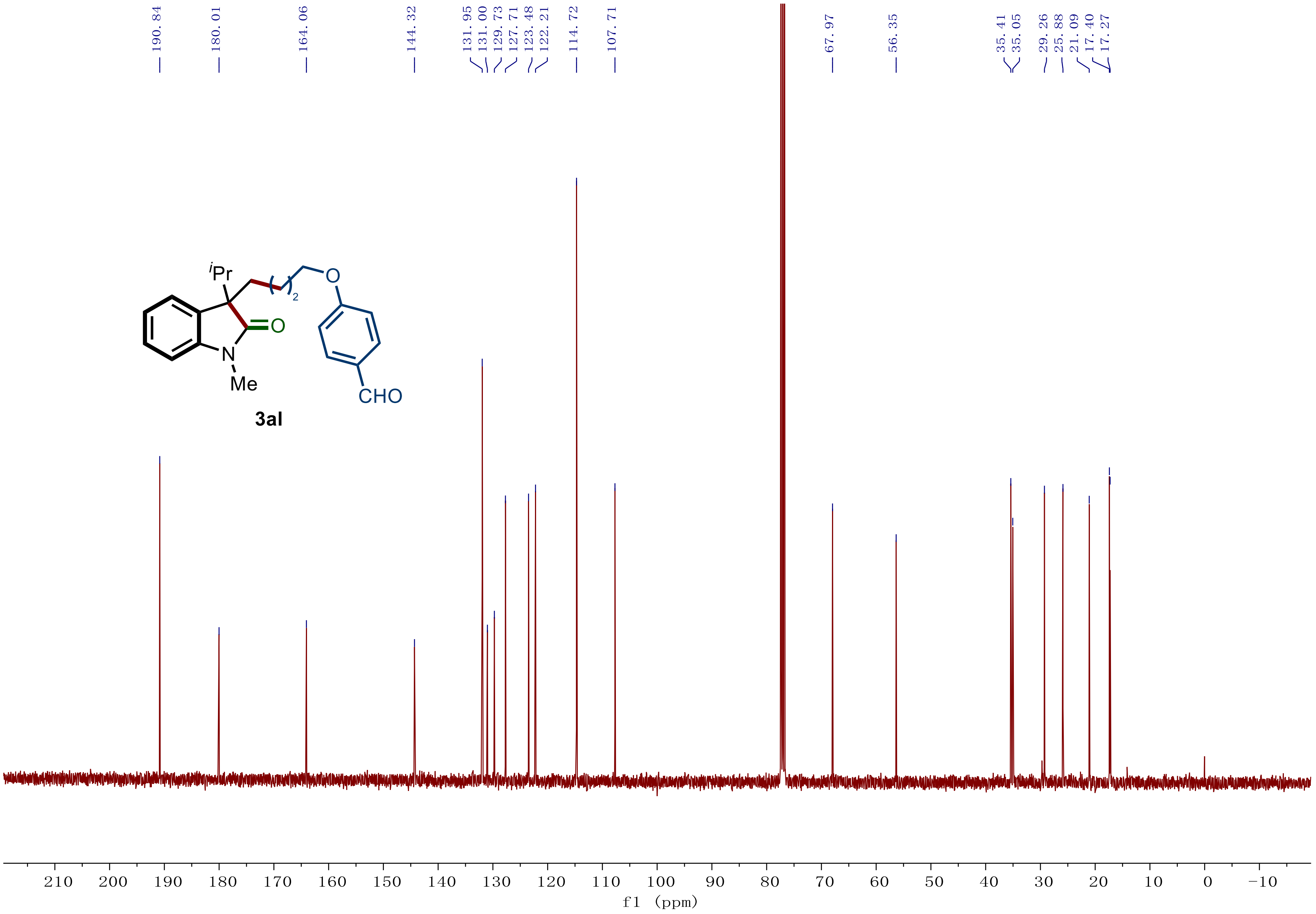

Supplementary Figure 157

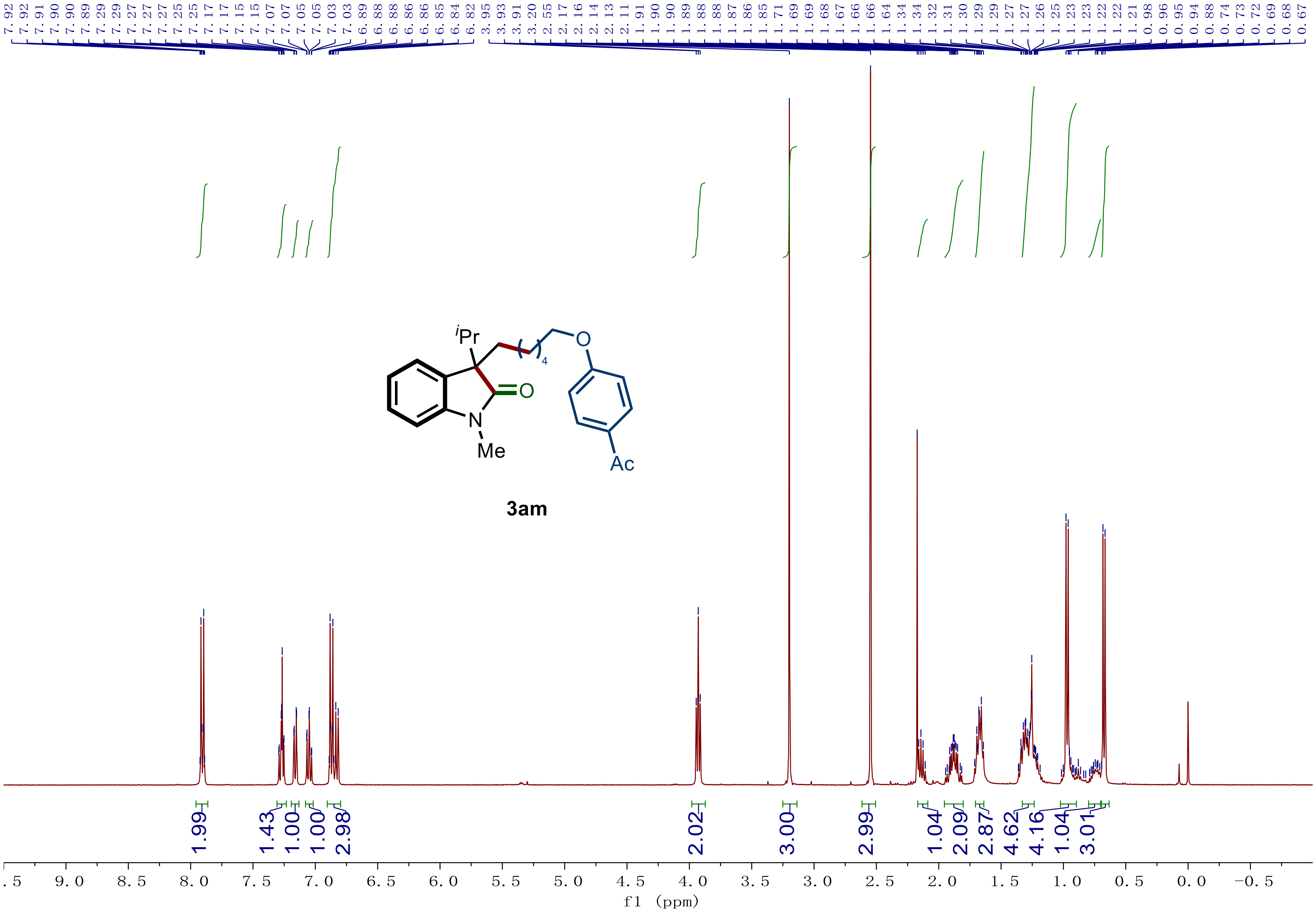

Supplementary Figure 158

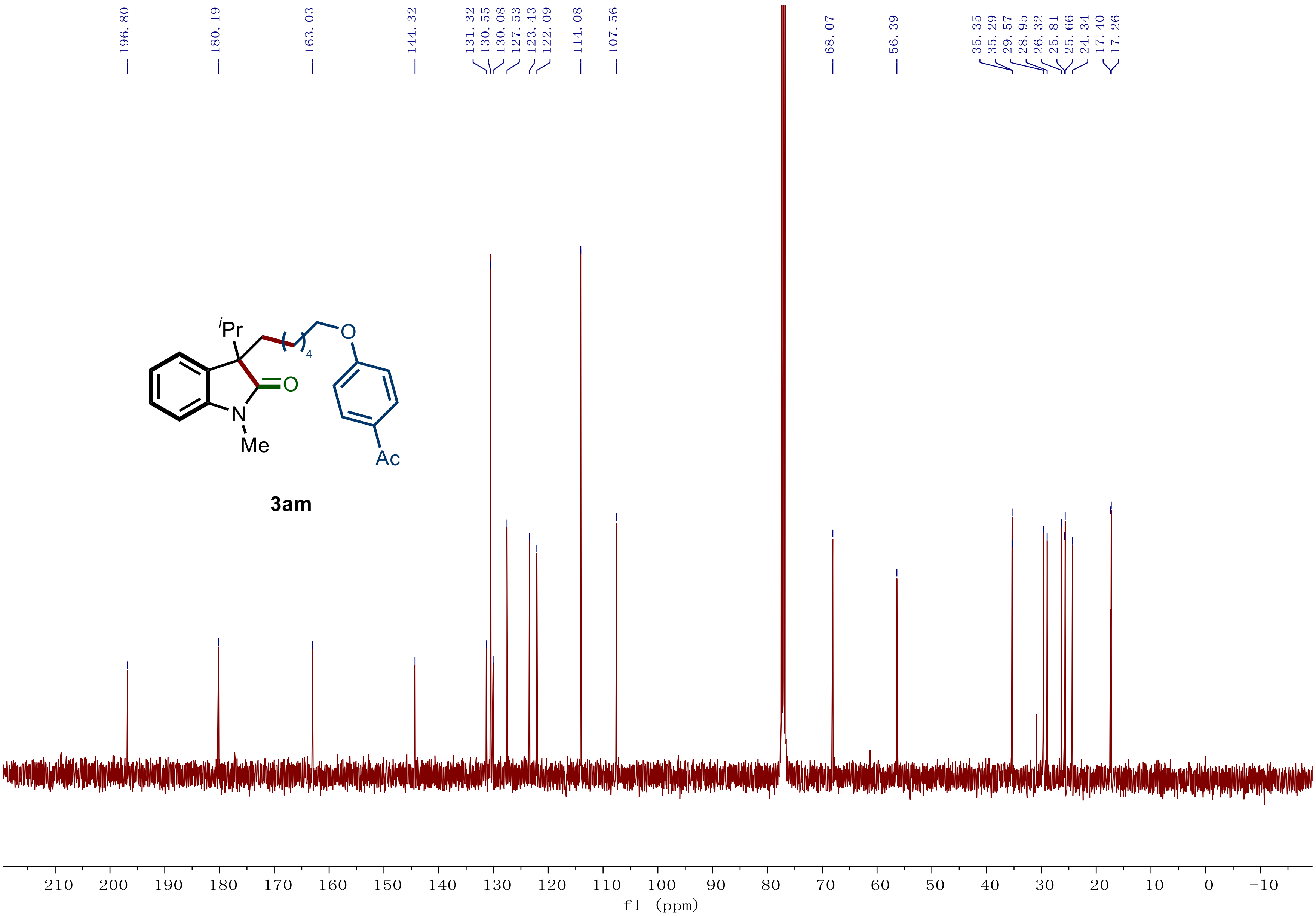

Supplementary Figure 159

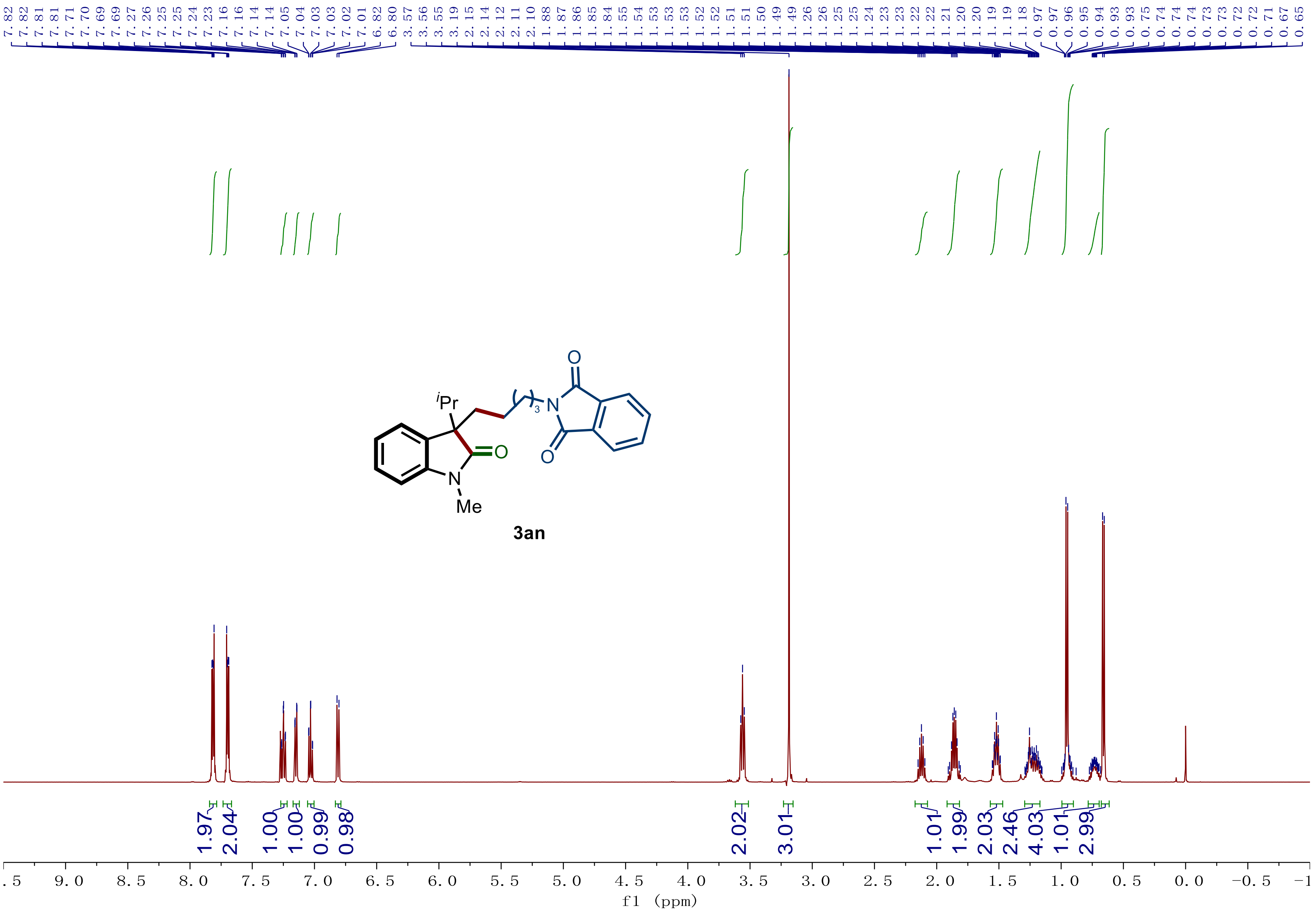

Supplementary Figure 160

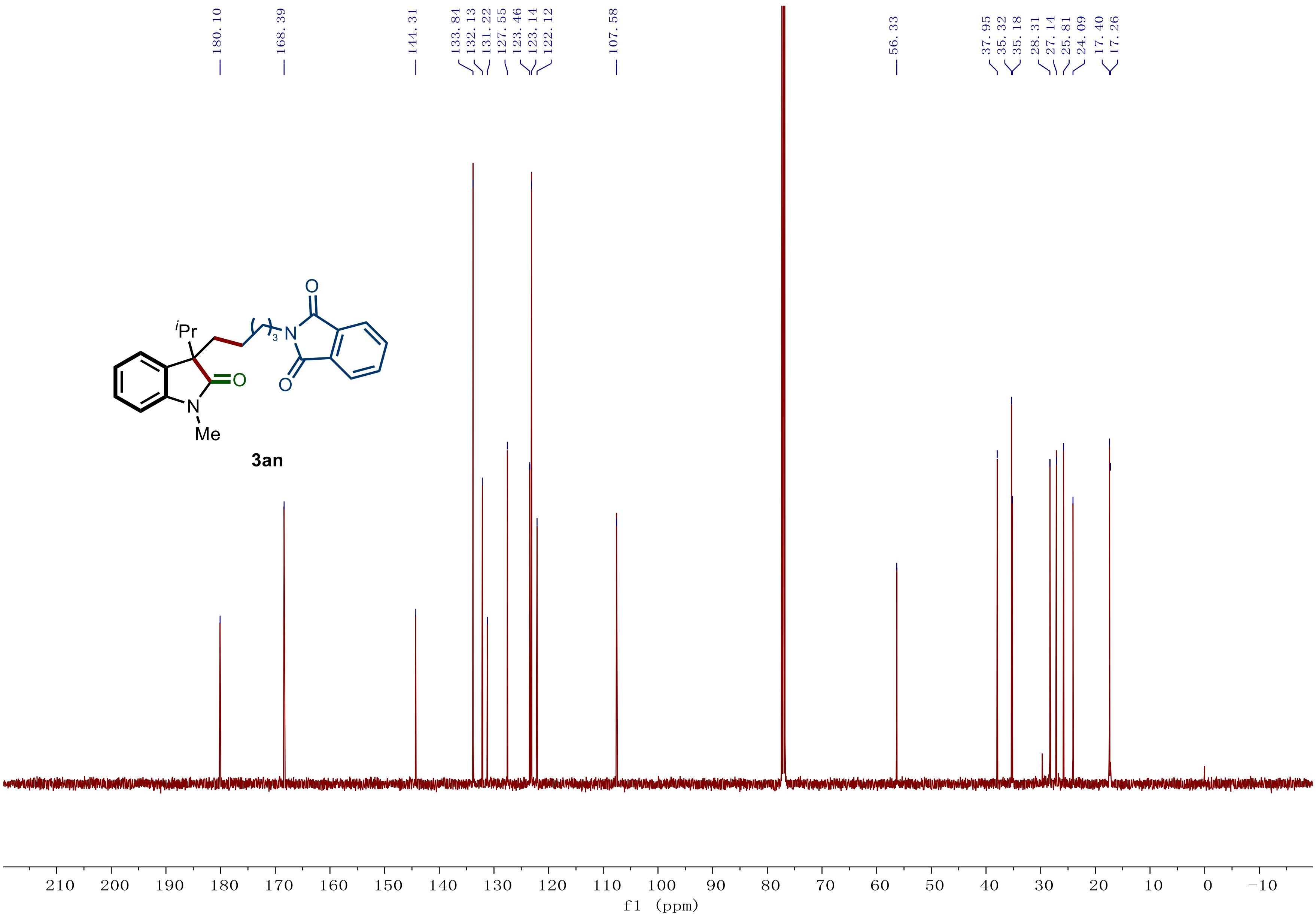

3an

Supplementary Figure 161

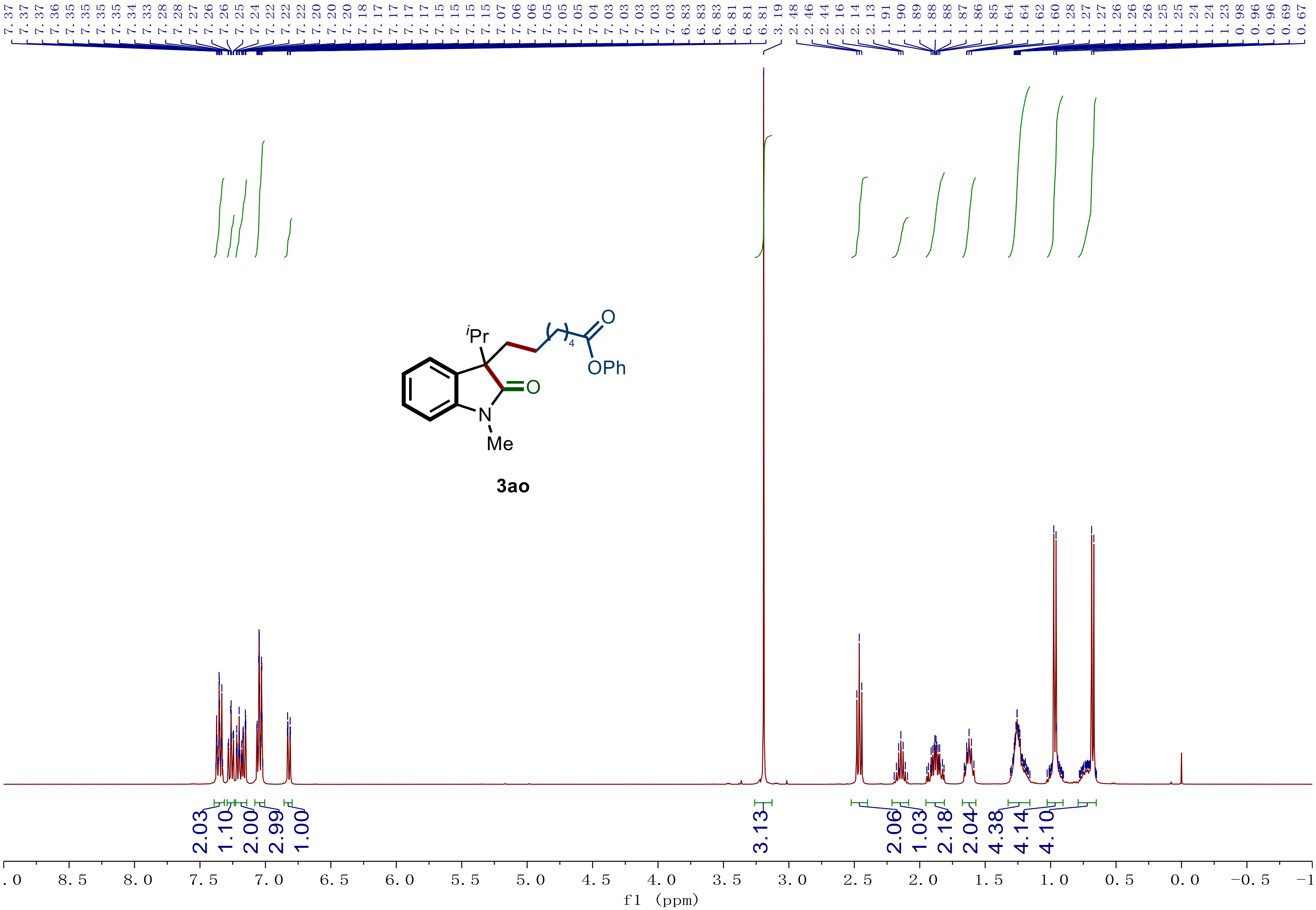

Supplementary Figure 162

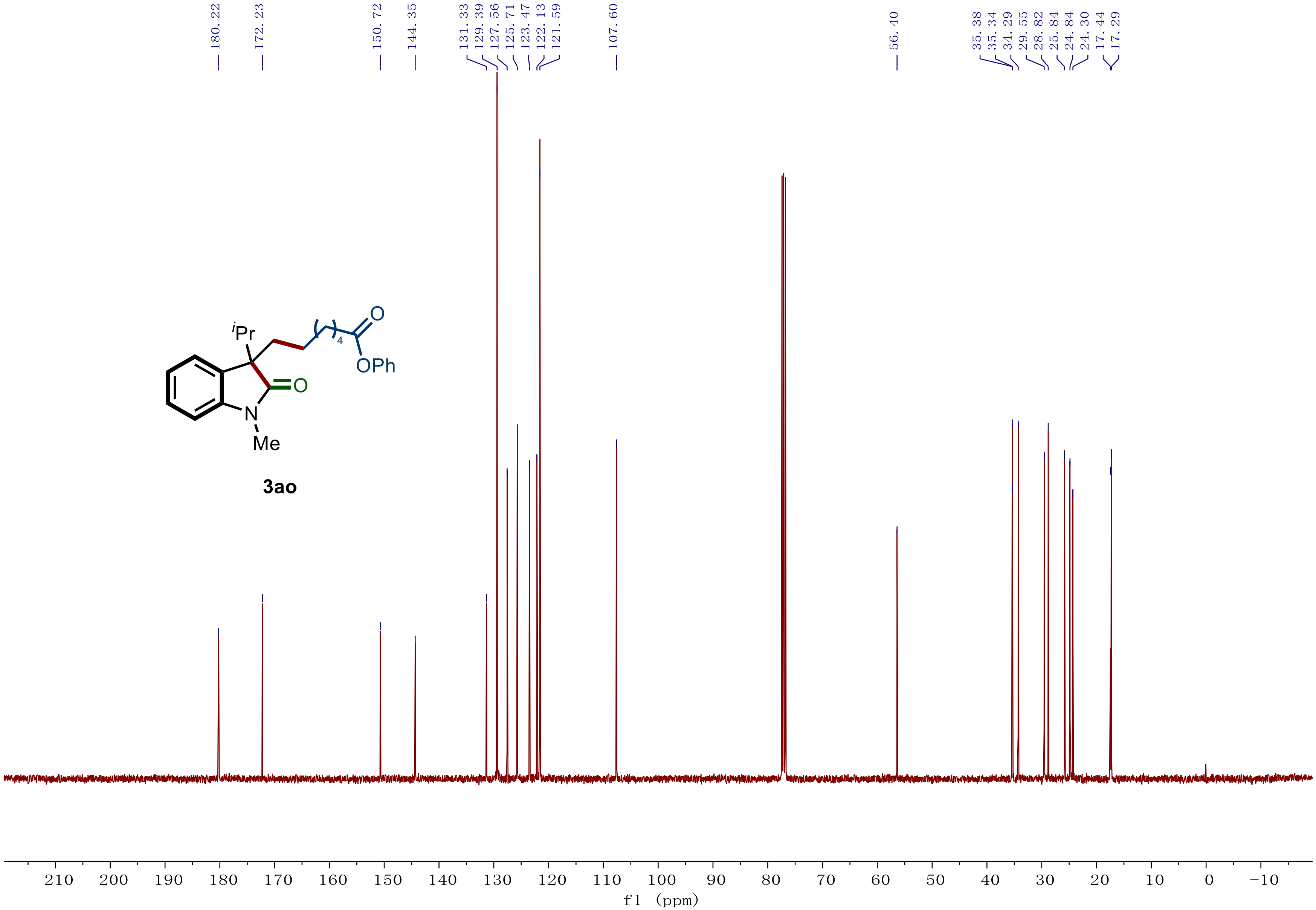

Supplementary Figure 163

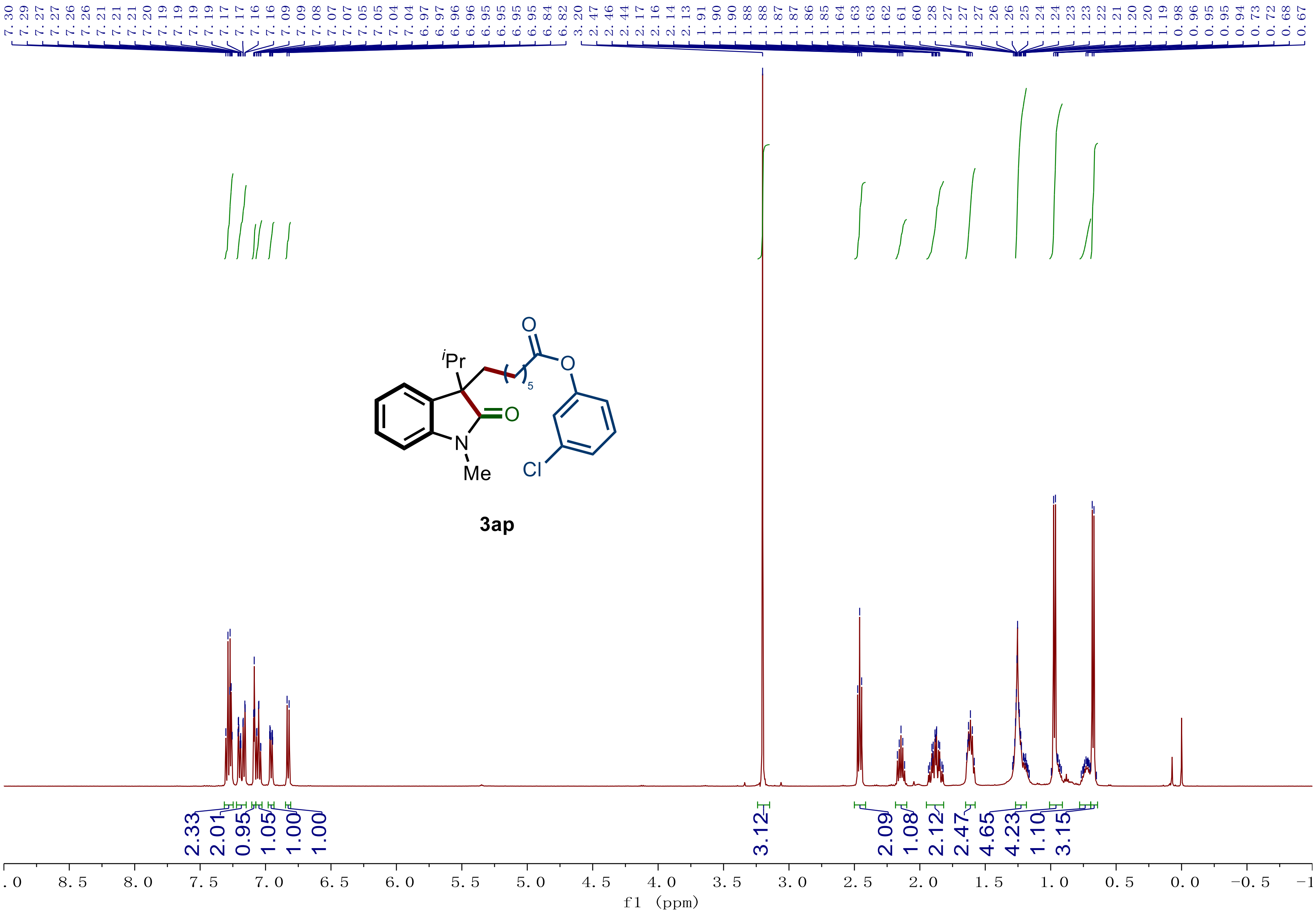

Supplementary Figure 164

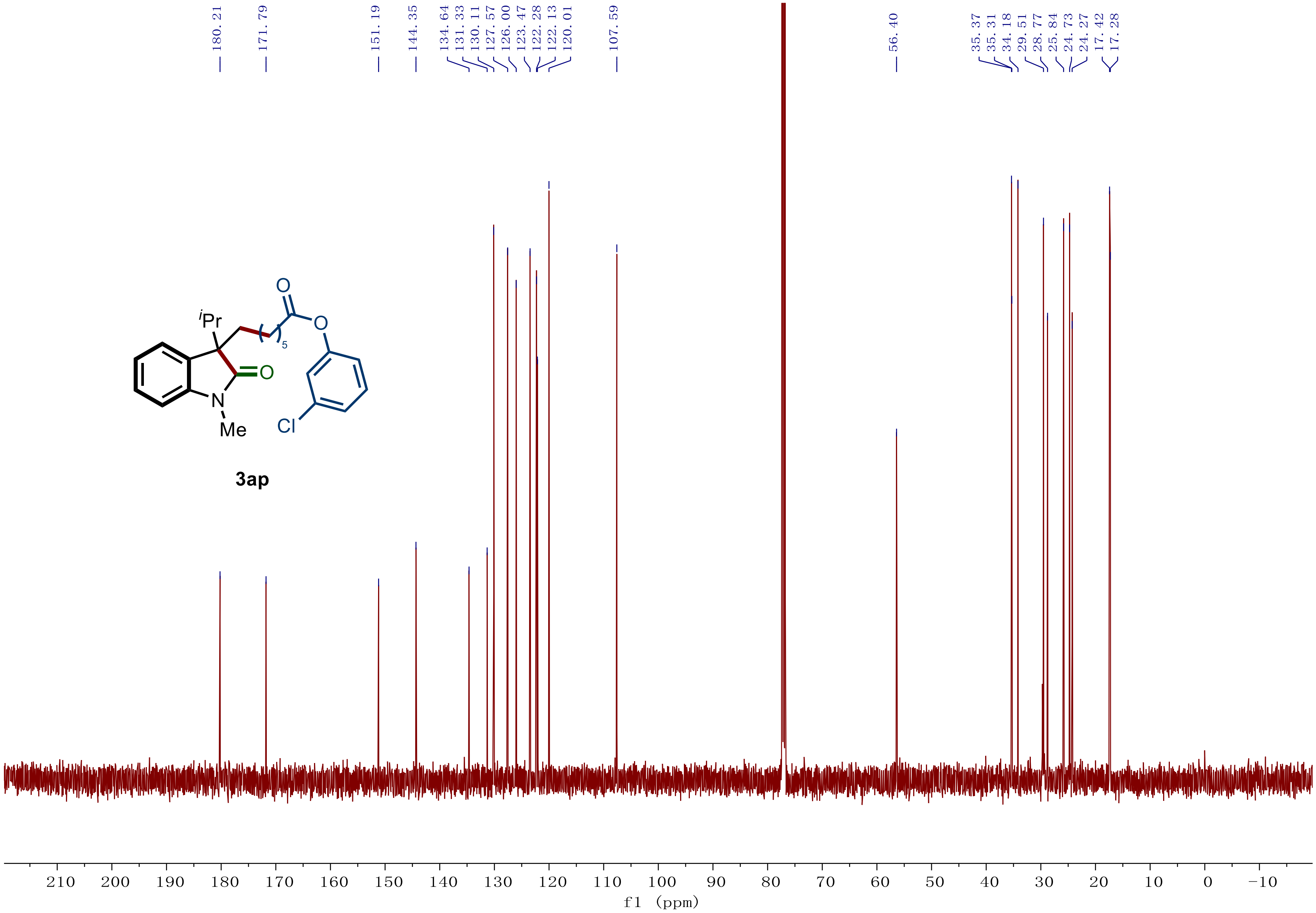

Supplementary Figure 165

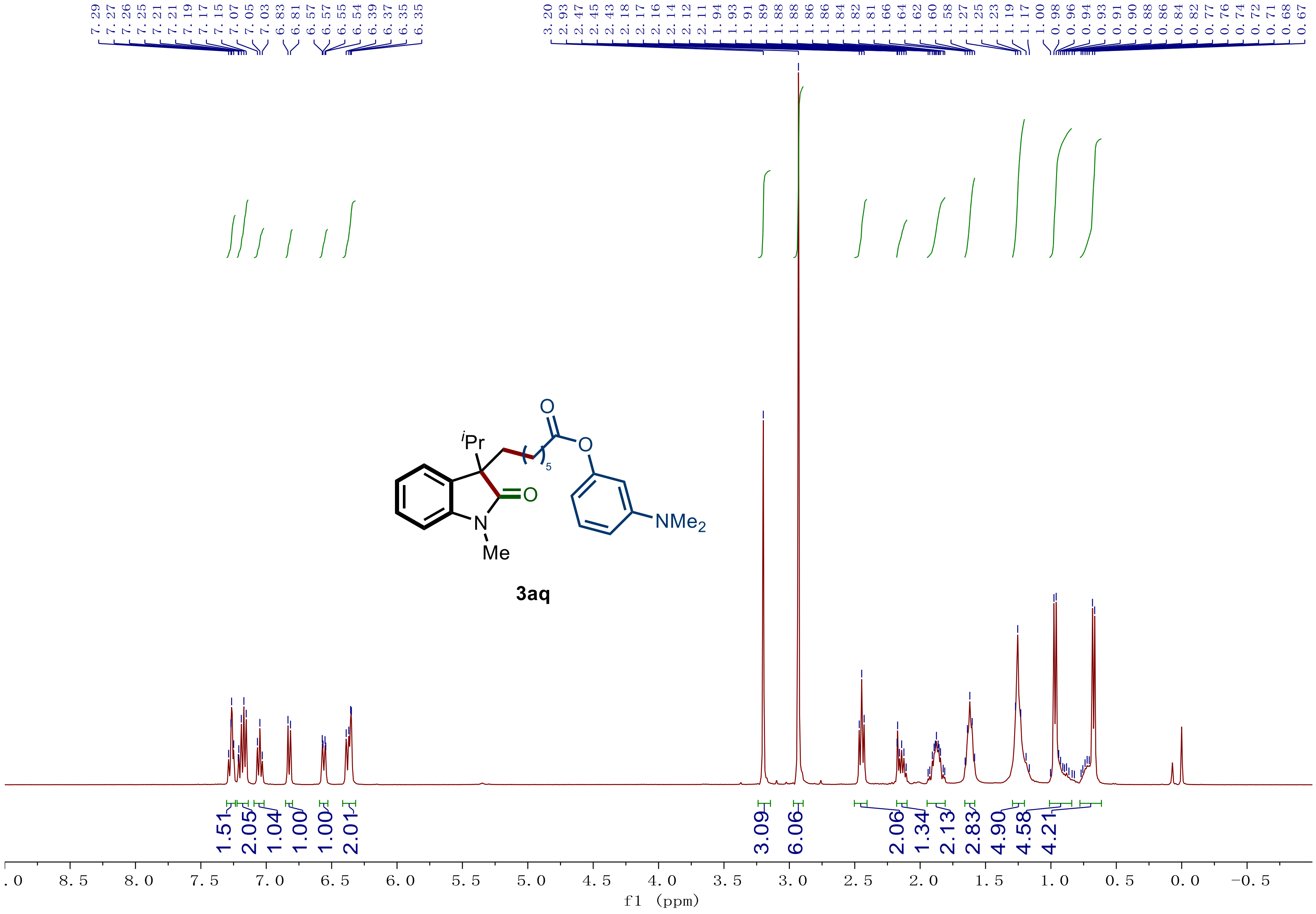

Supplementary Figure 166

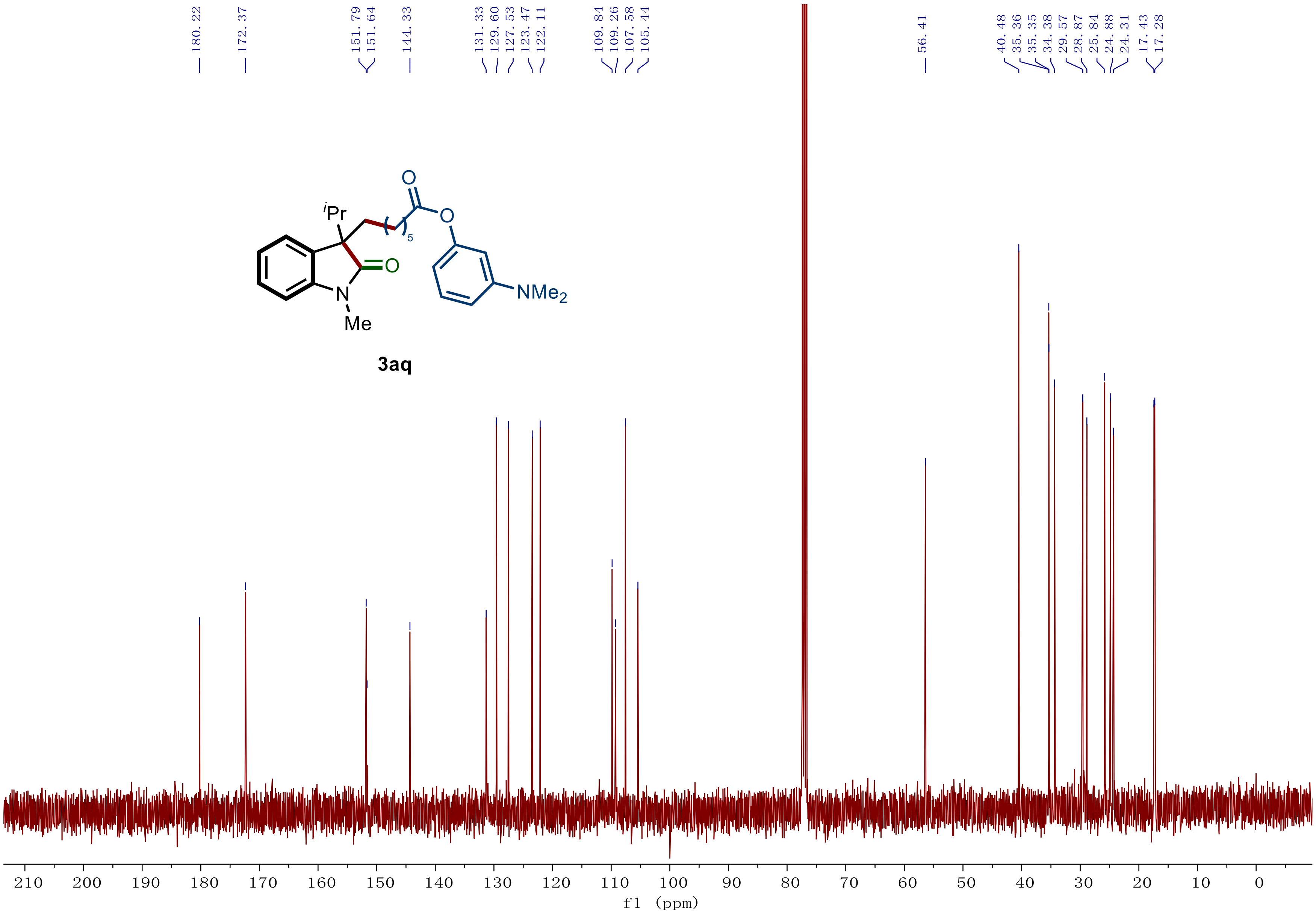

Supplementary Figure 167

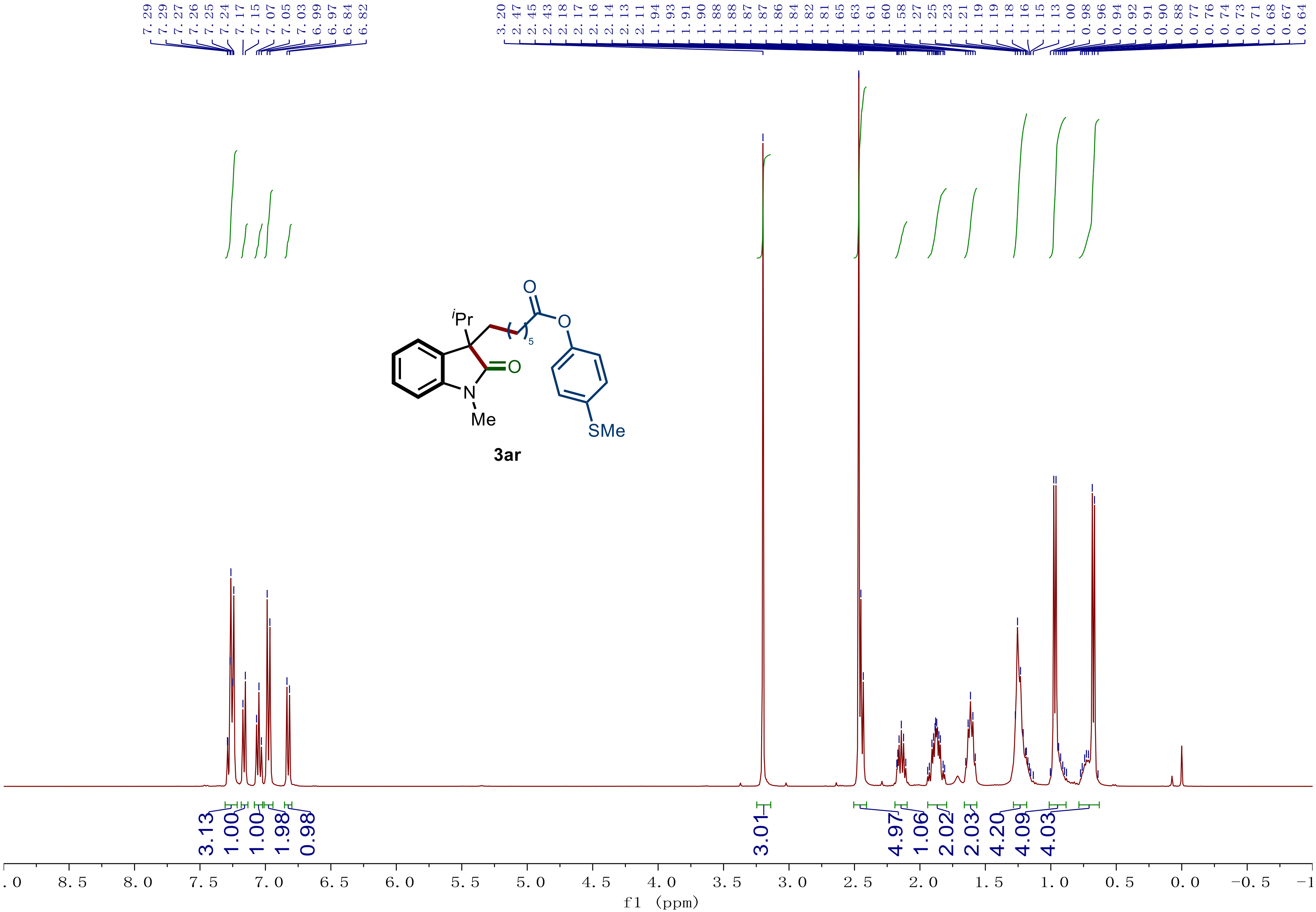

Supplementary Figure 168

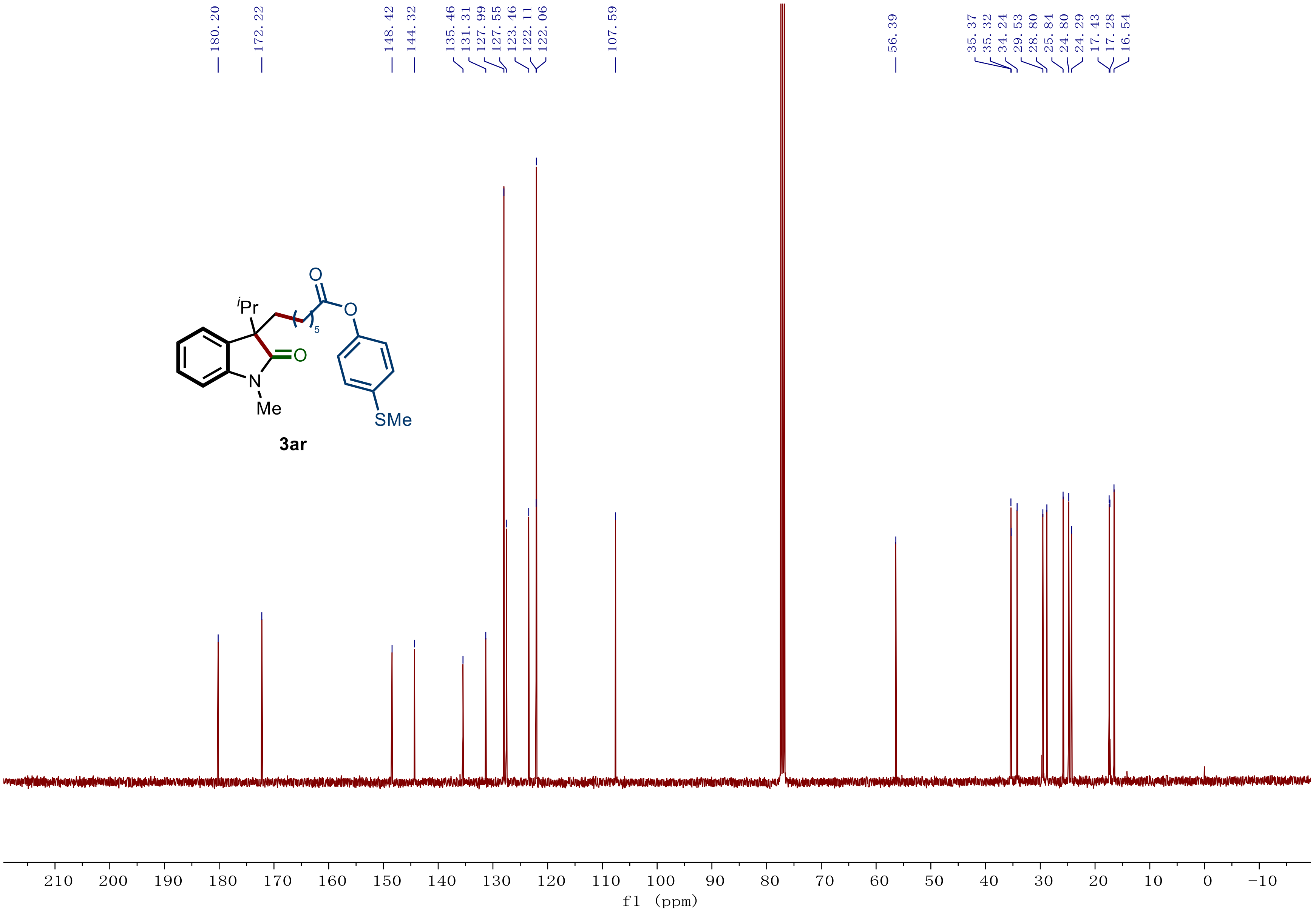

Supplementary Figure 169

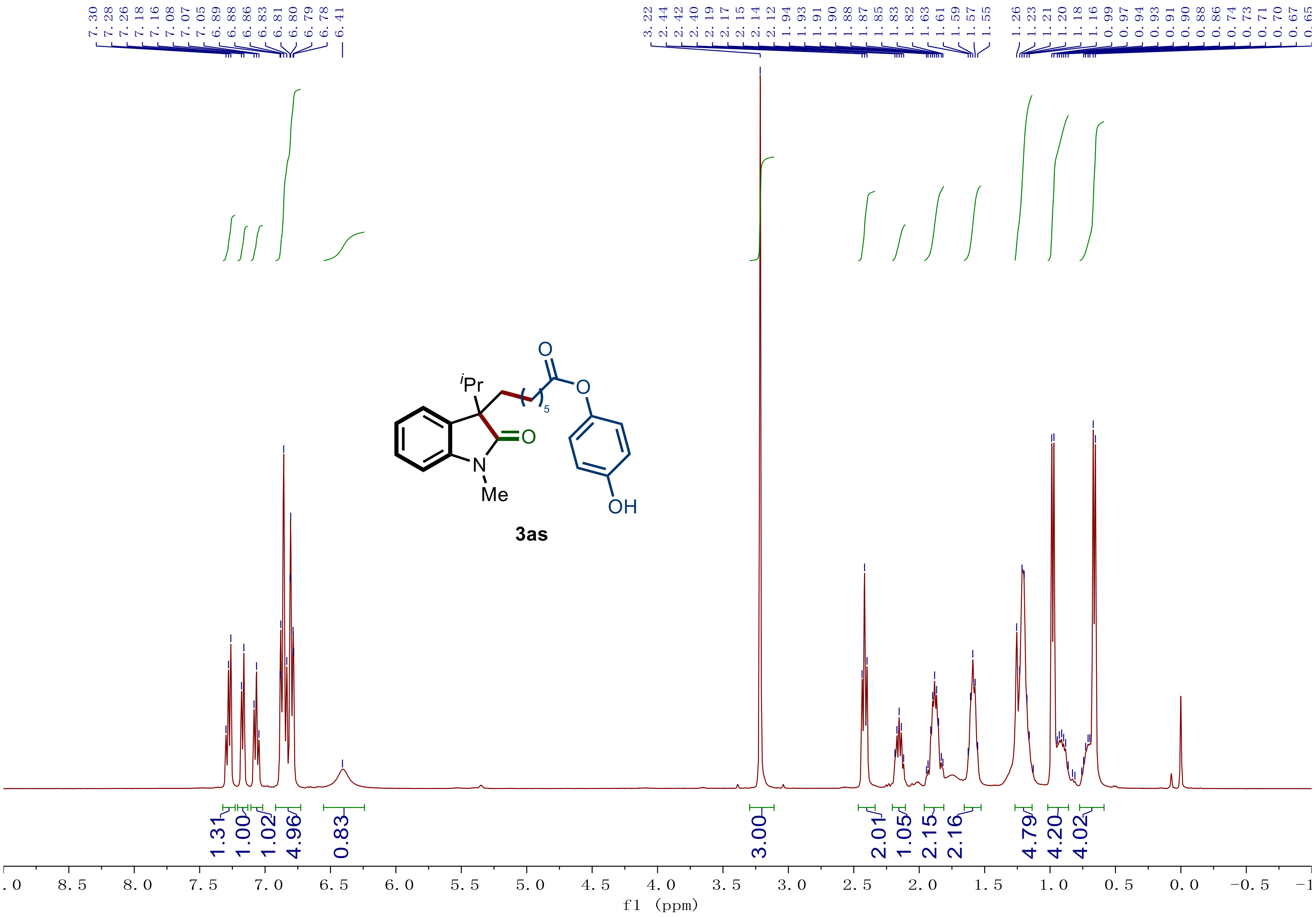

Supplementary Figure 170

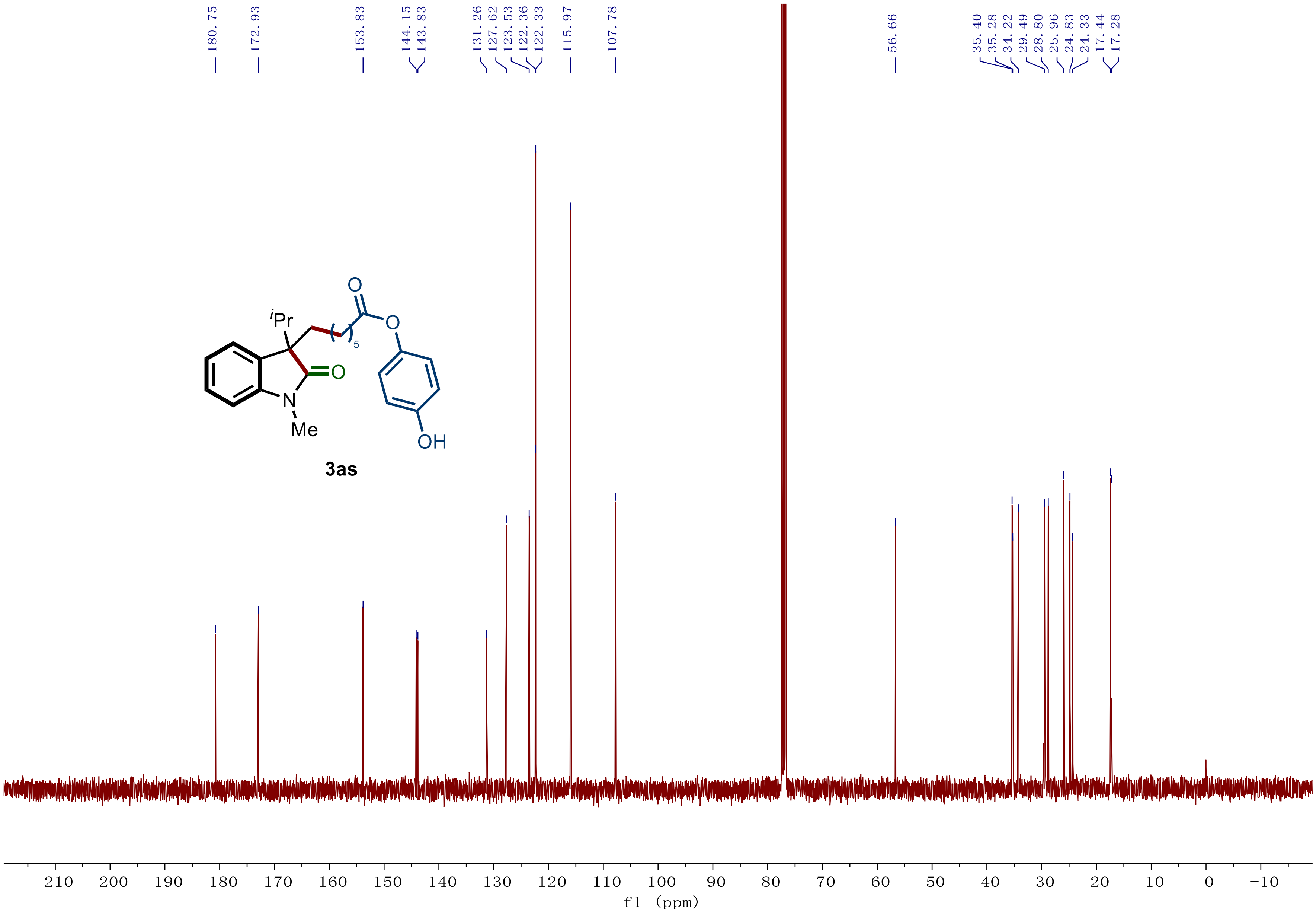

Supplementary Figure 171

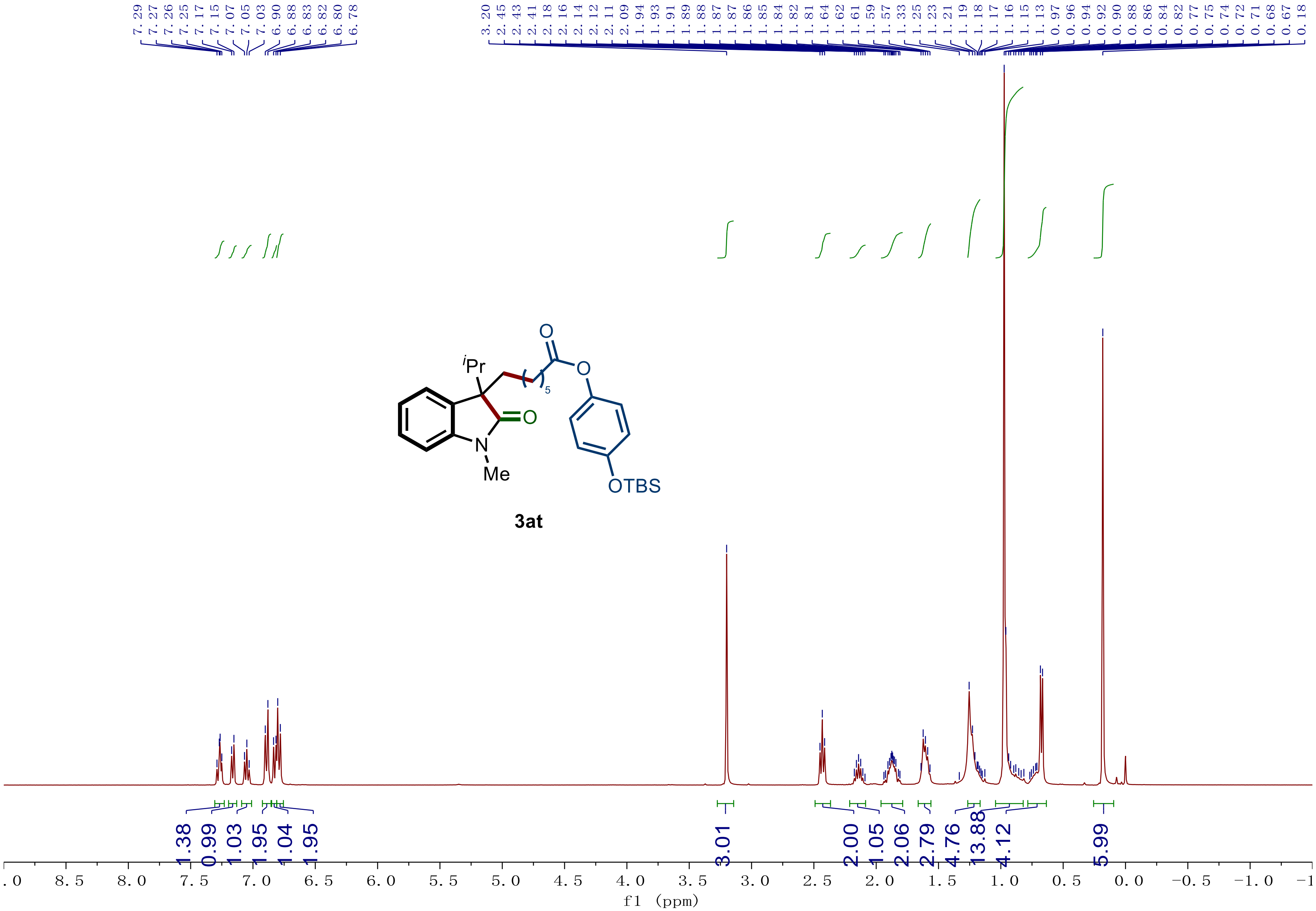

Supplementary Figure 172

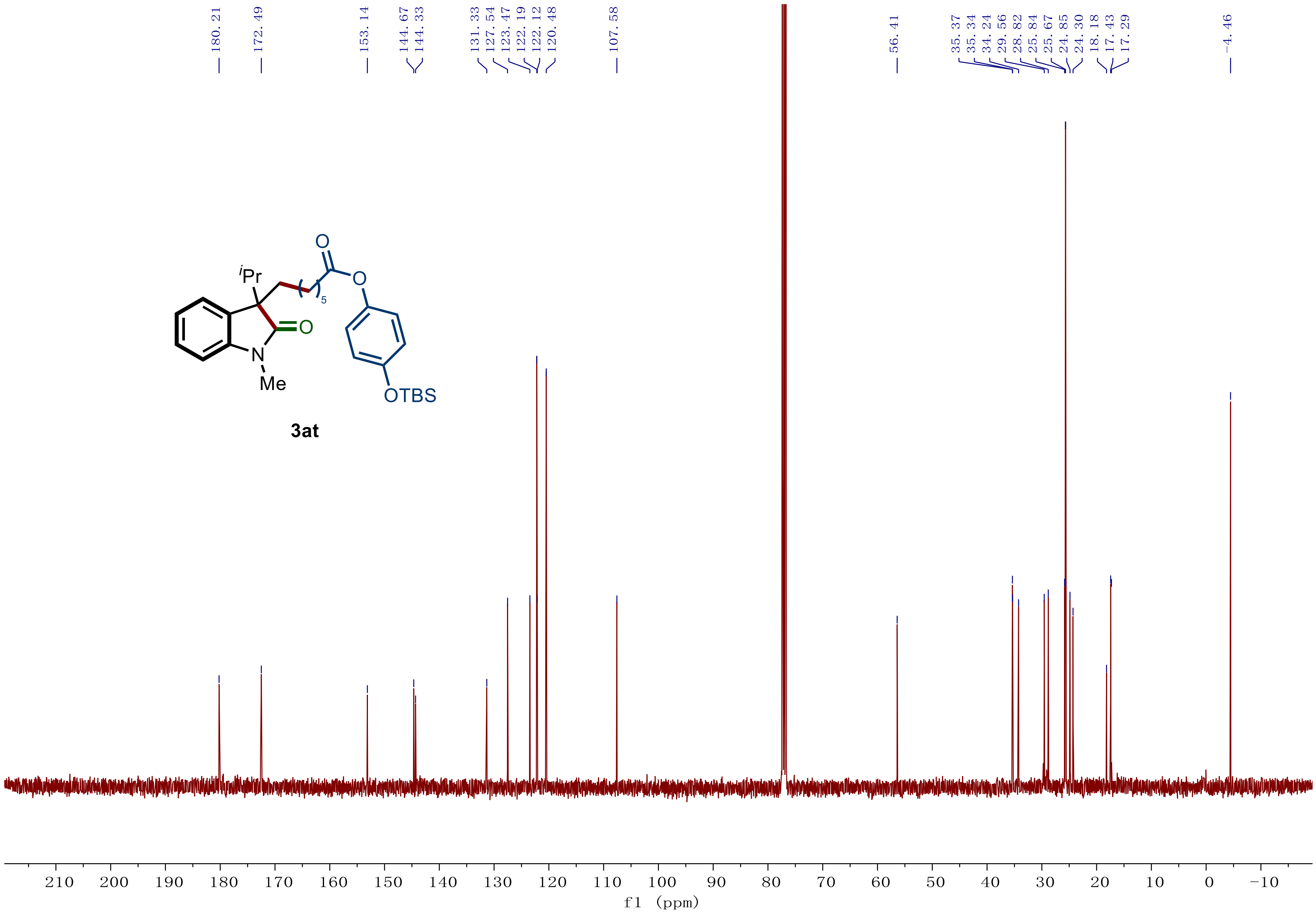

Supplementary Figure 173

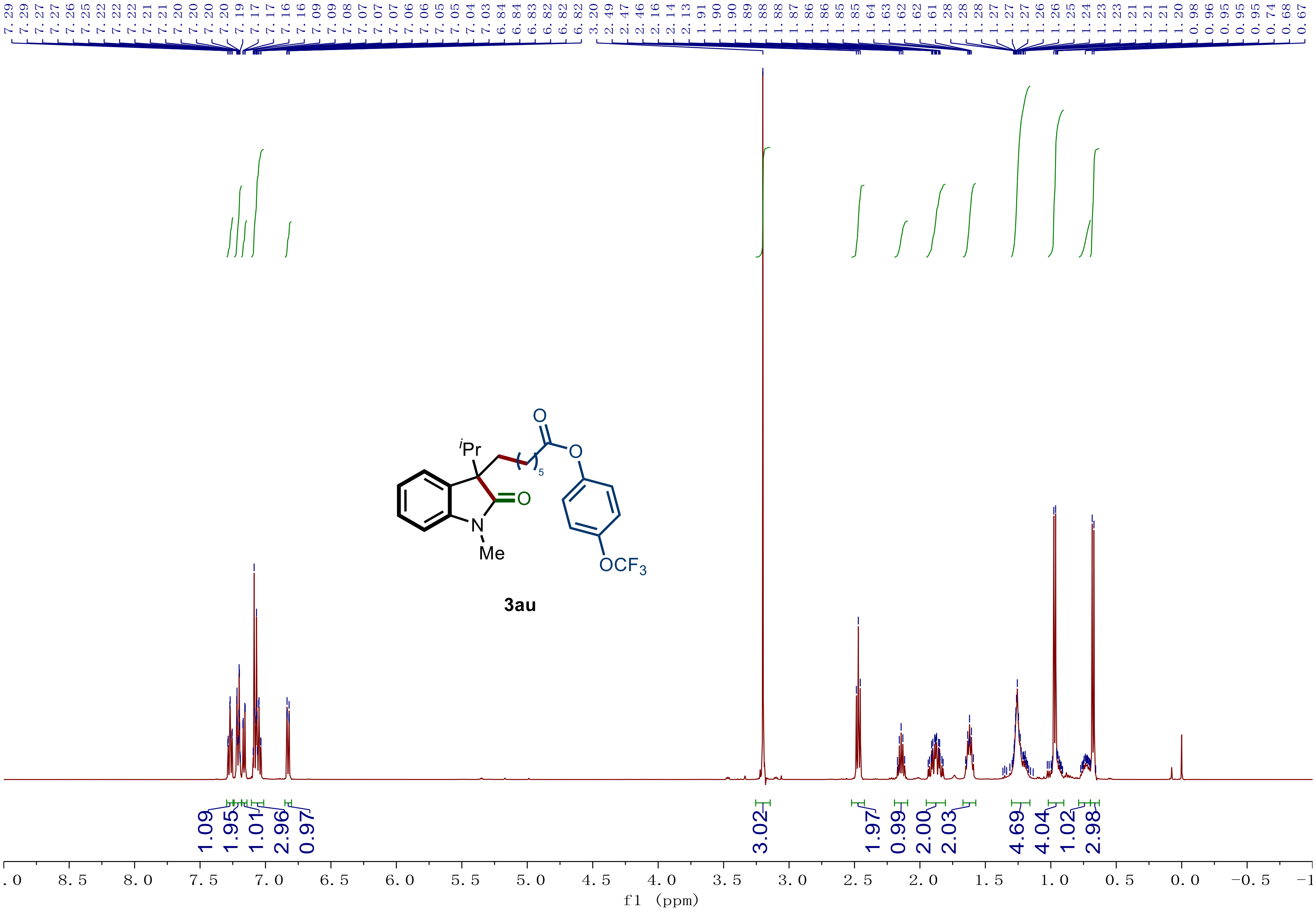

Supplementary Figure 174

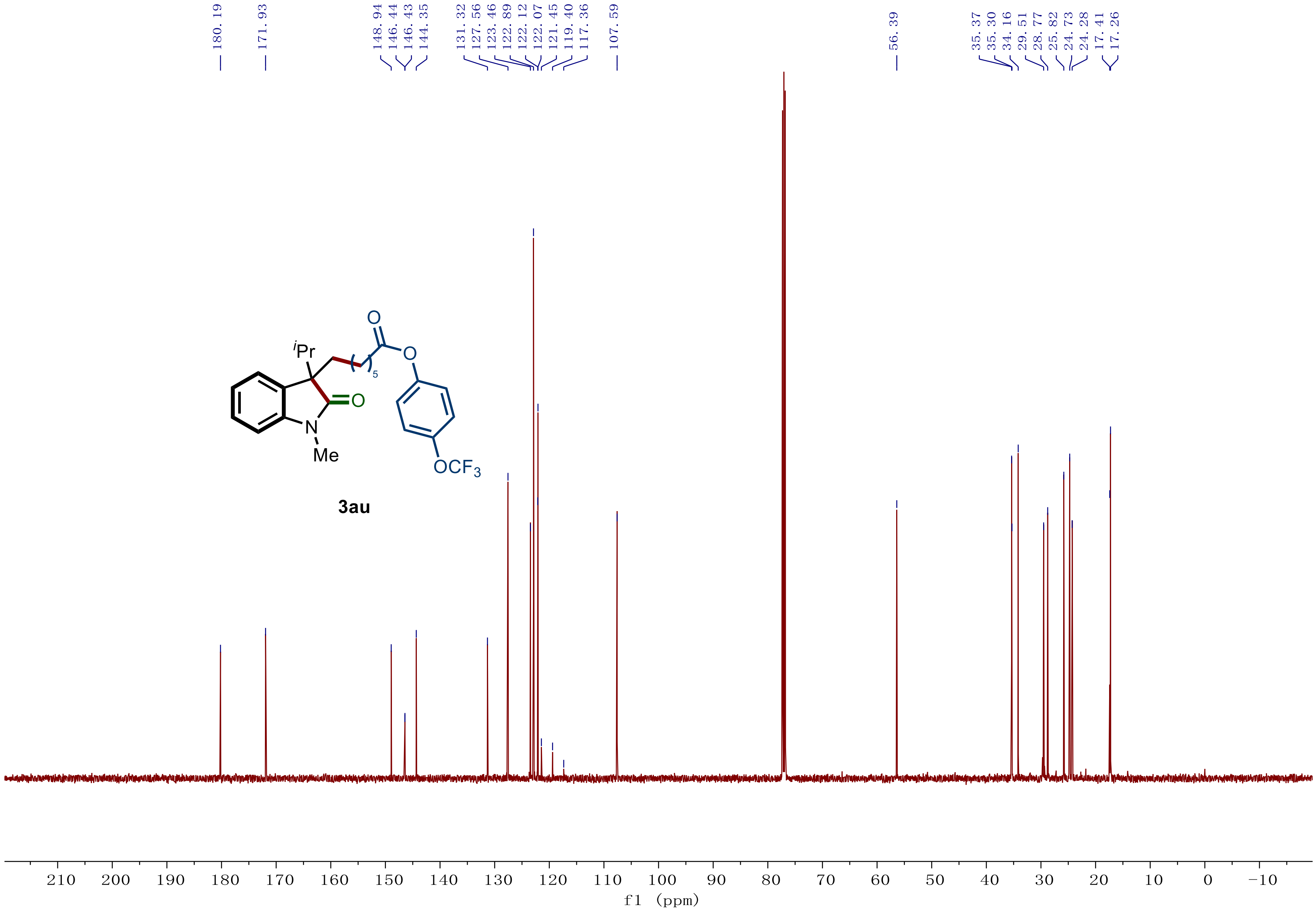

Supplementary Figure 175

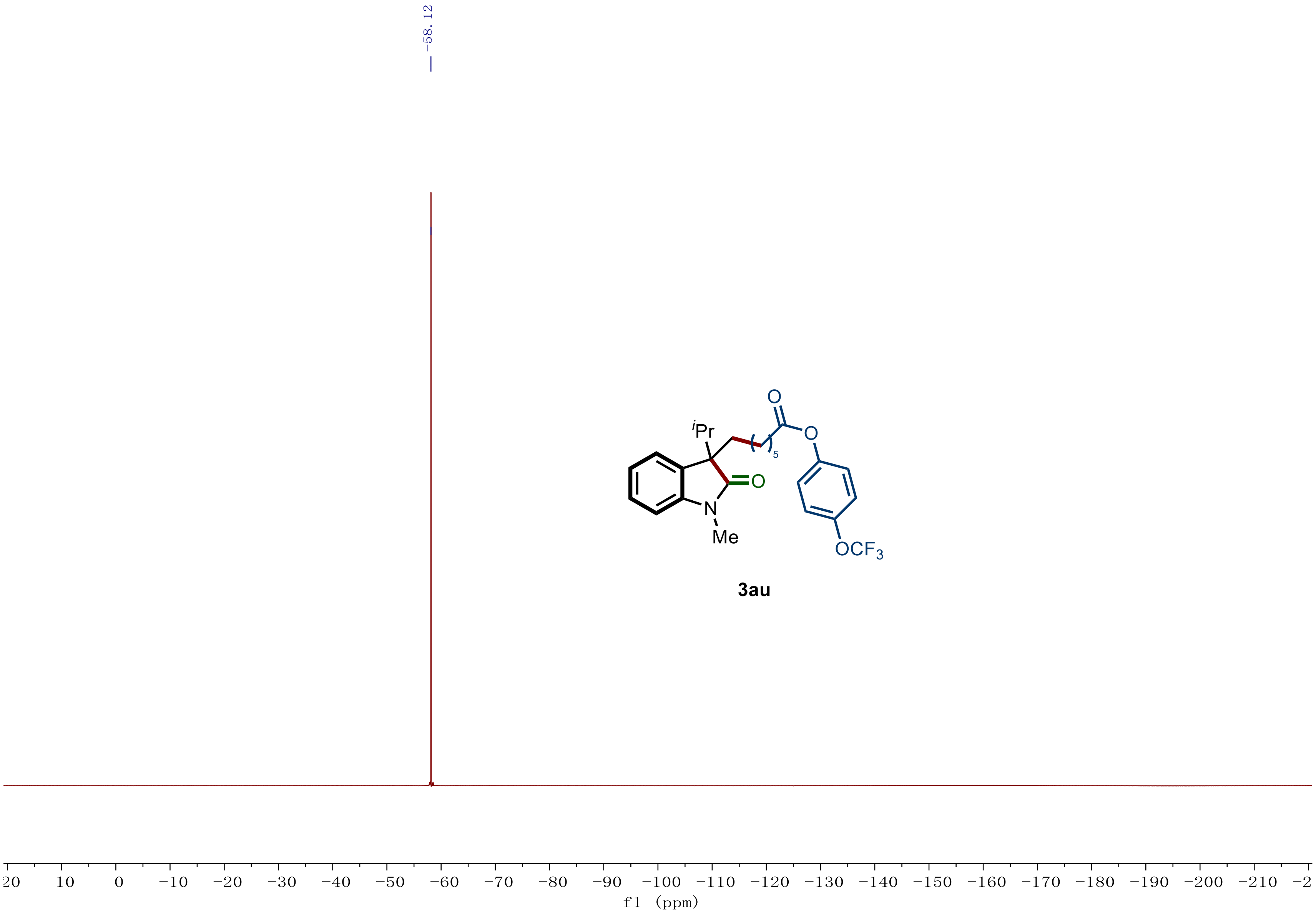

3au

Supplementary Figure 176

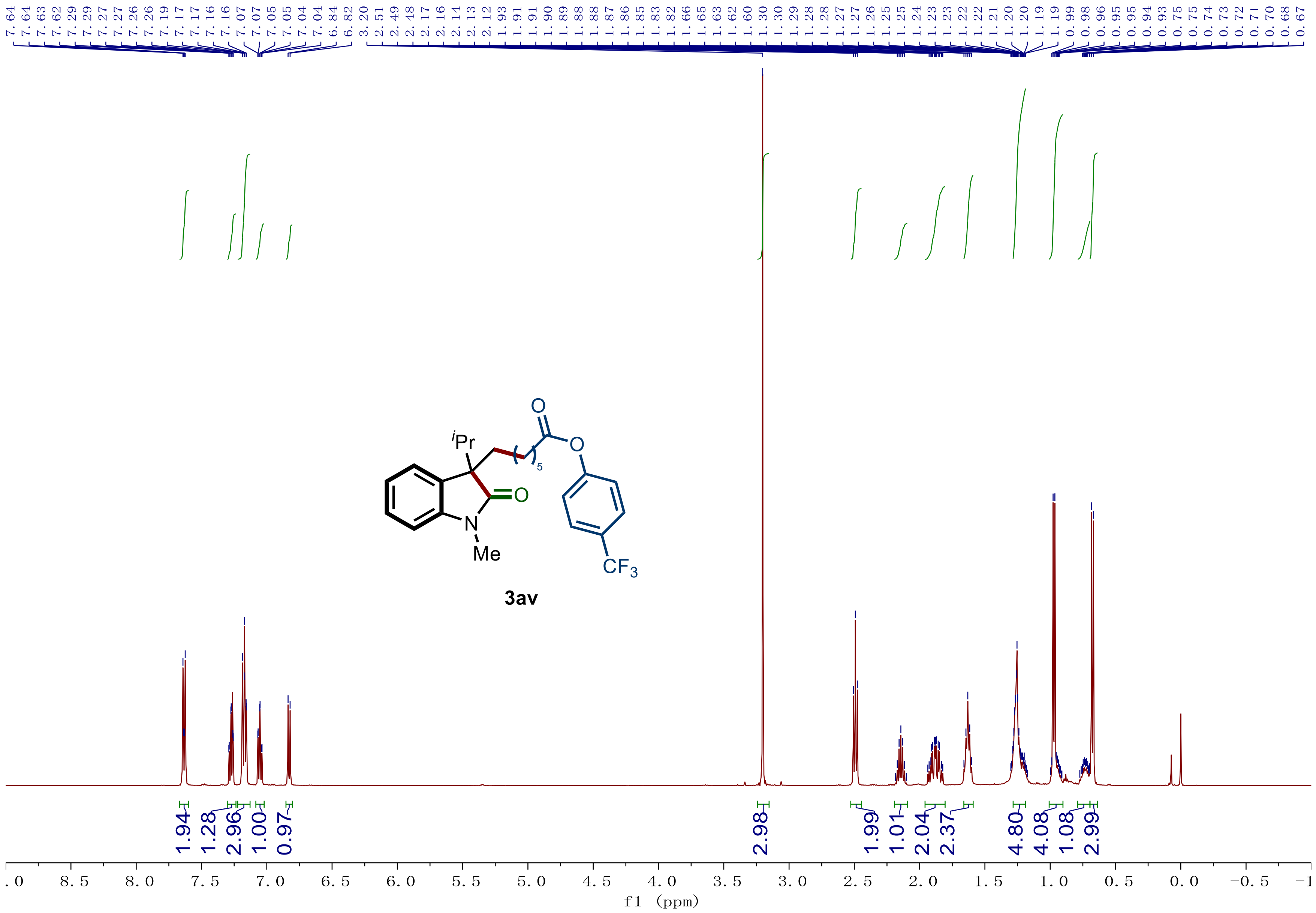

Supplementary Figure 177

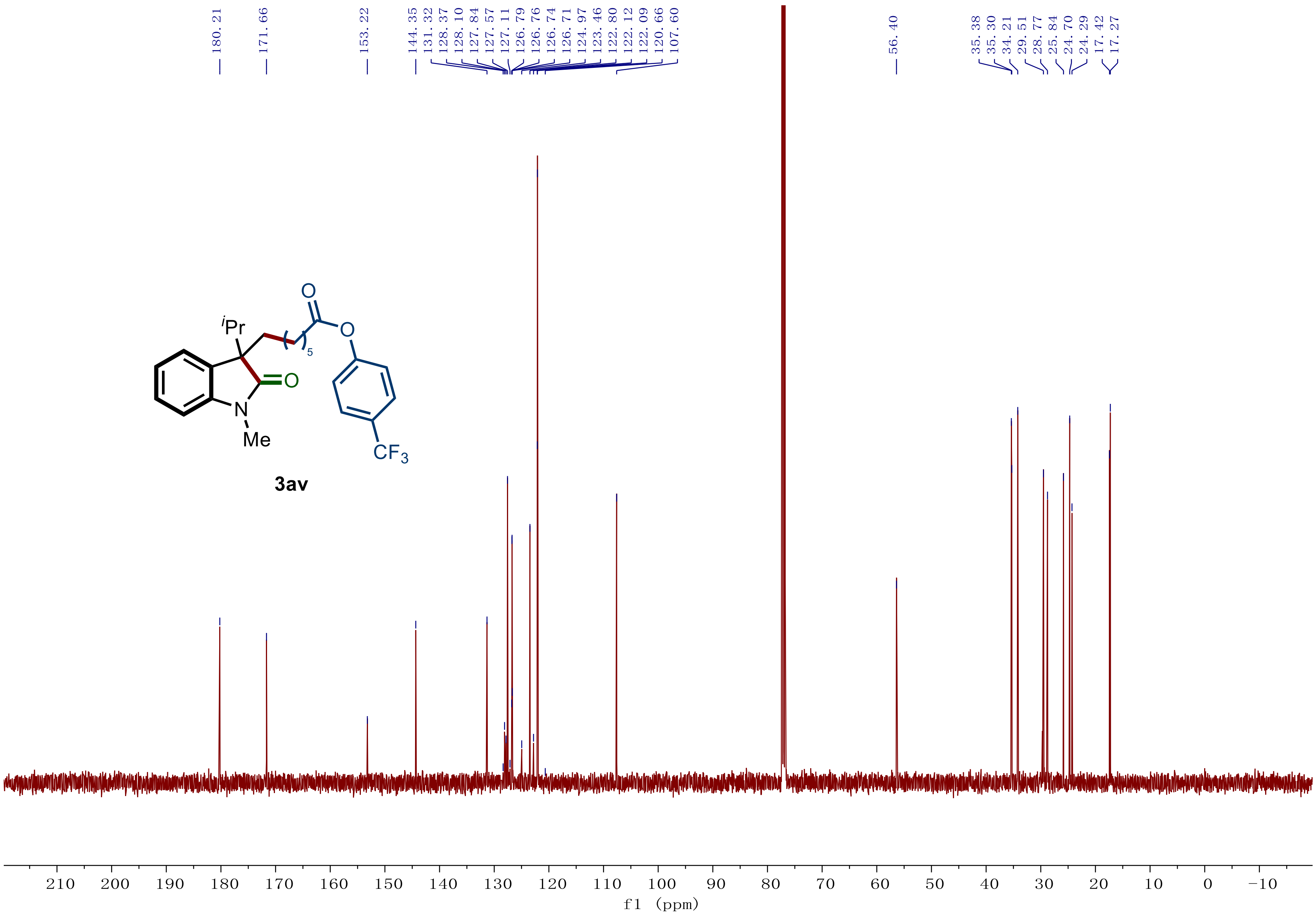

3av

Supplementary Figure 178

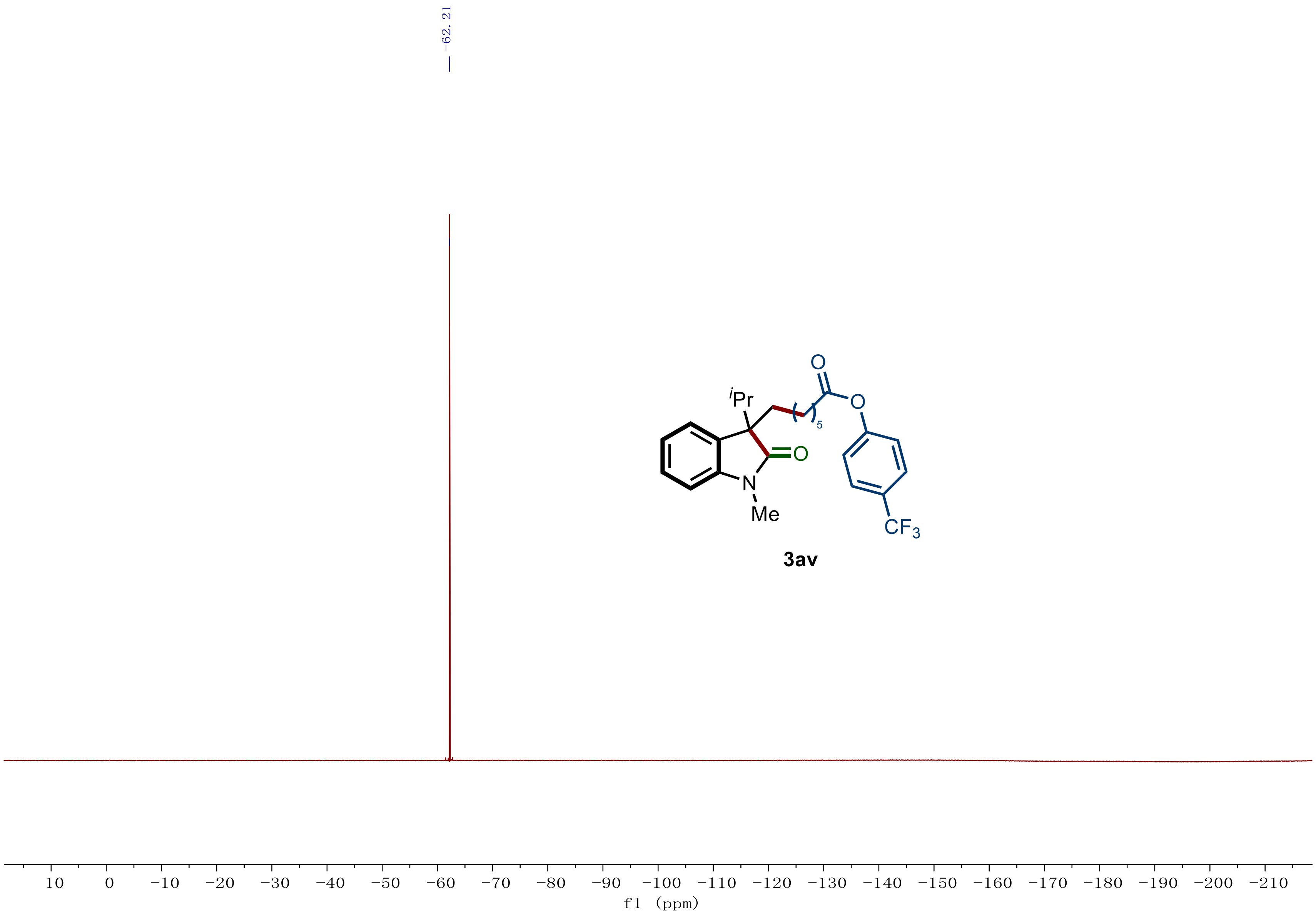

3av

Supplementary Figure 179

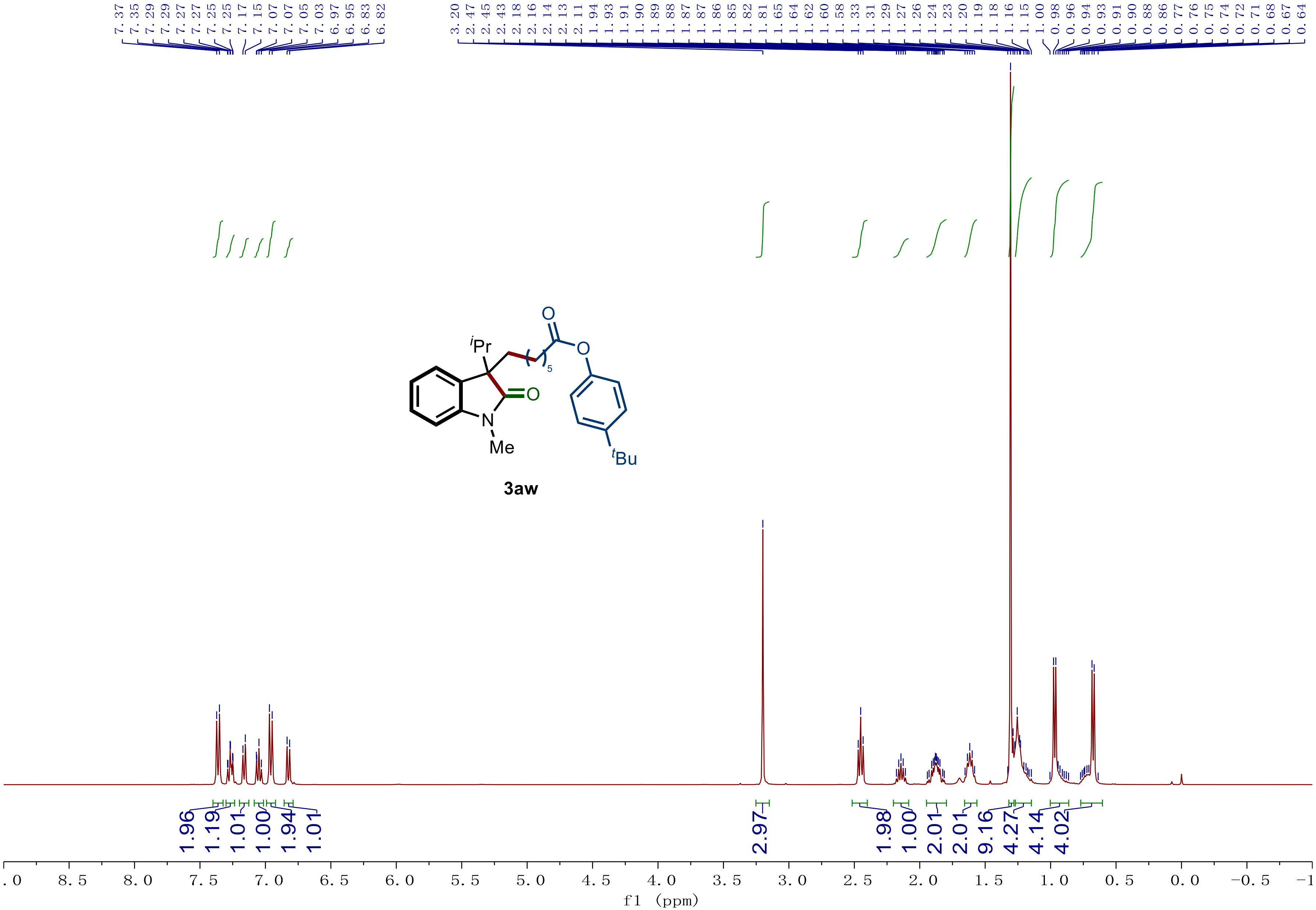

Supplementary Figure 180

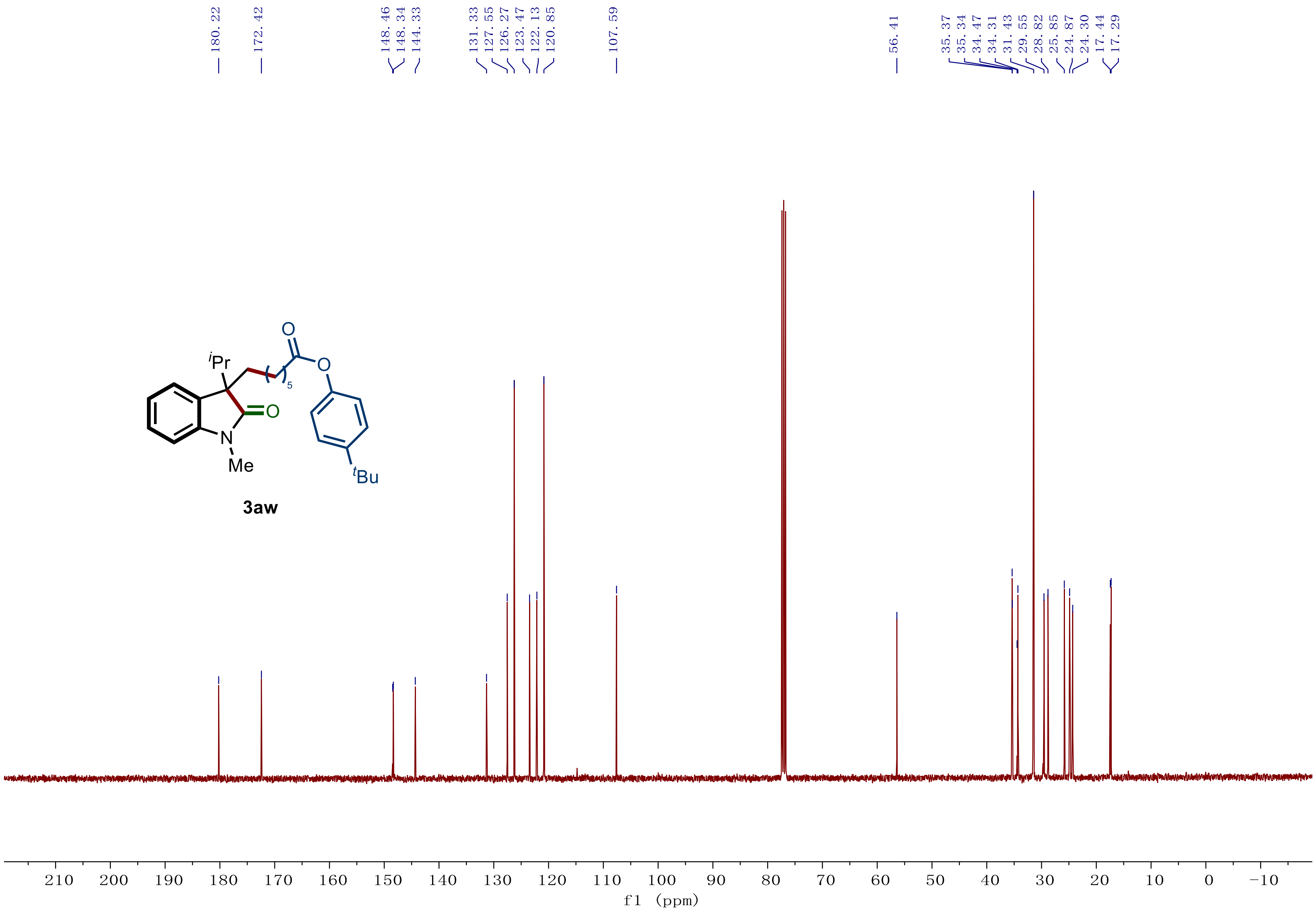

Supplementary Figure 181

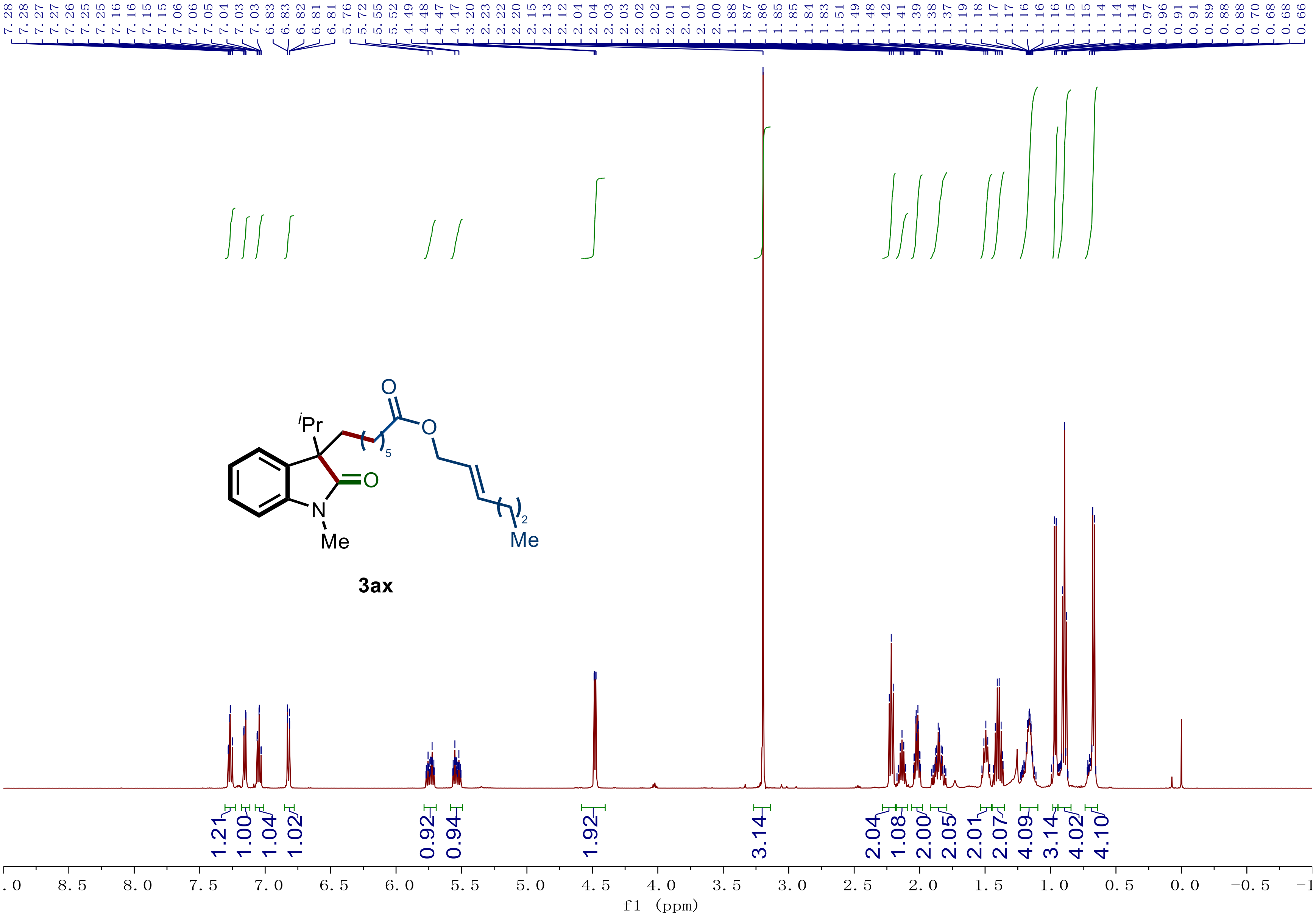

Supplementary Figure 182

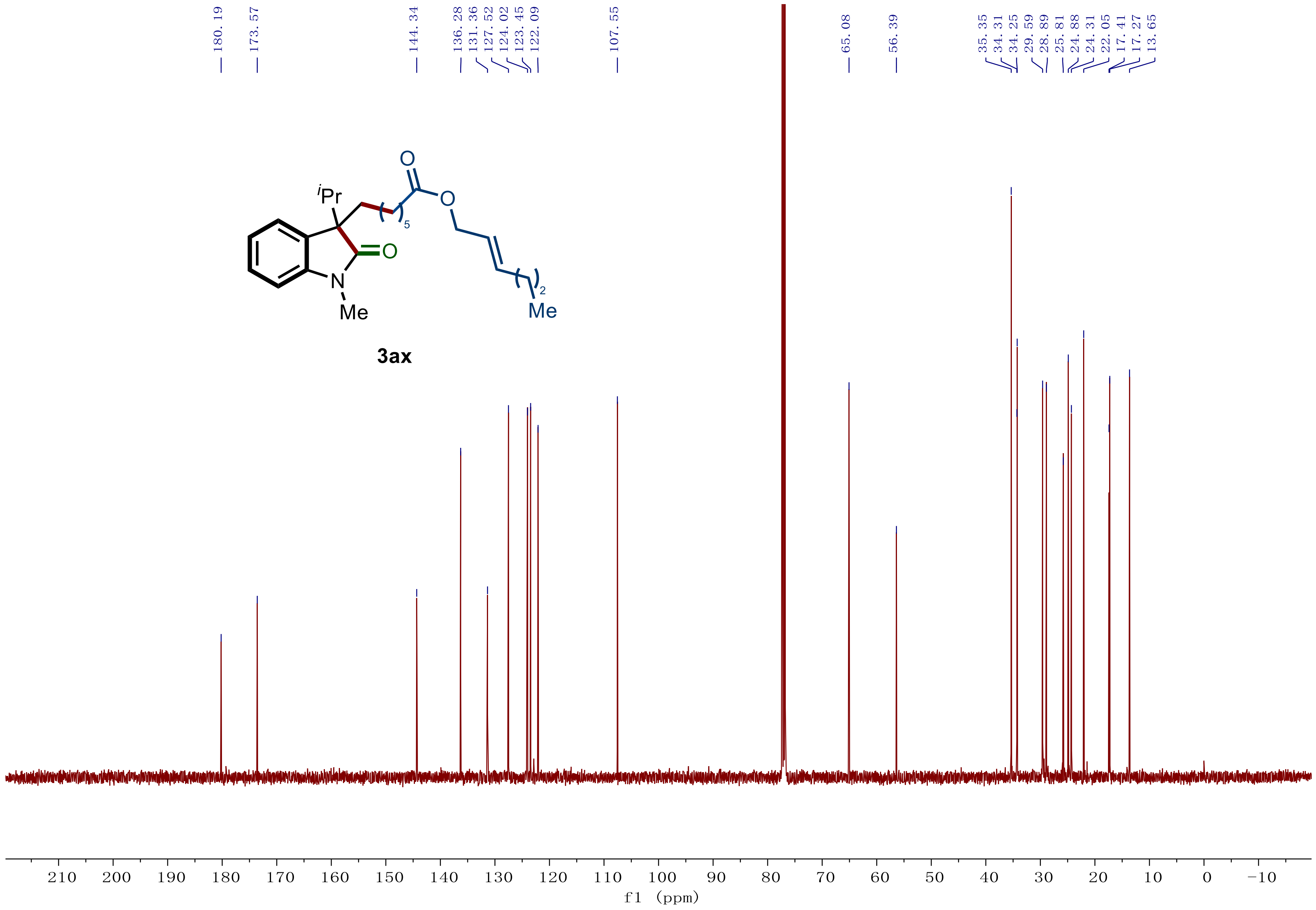

Supplementary Figure 183

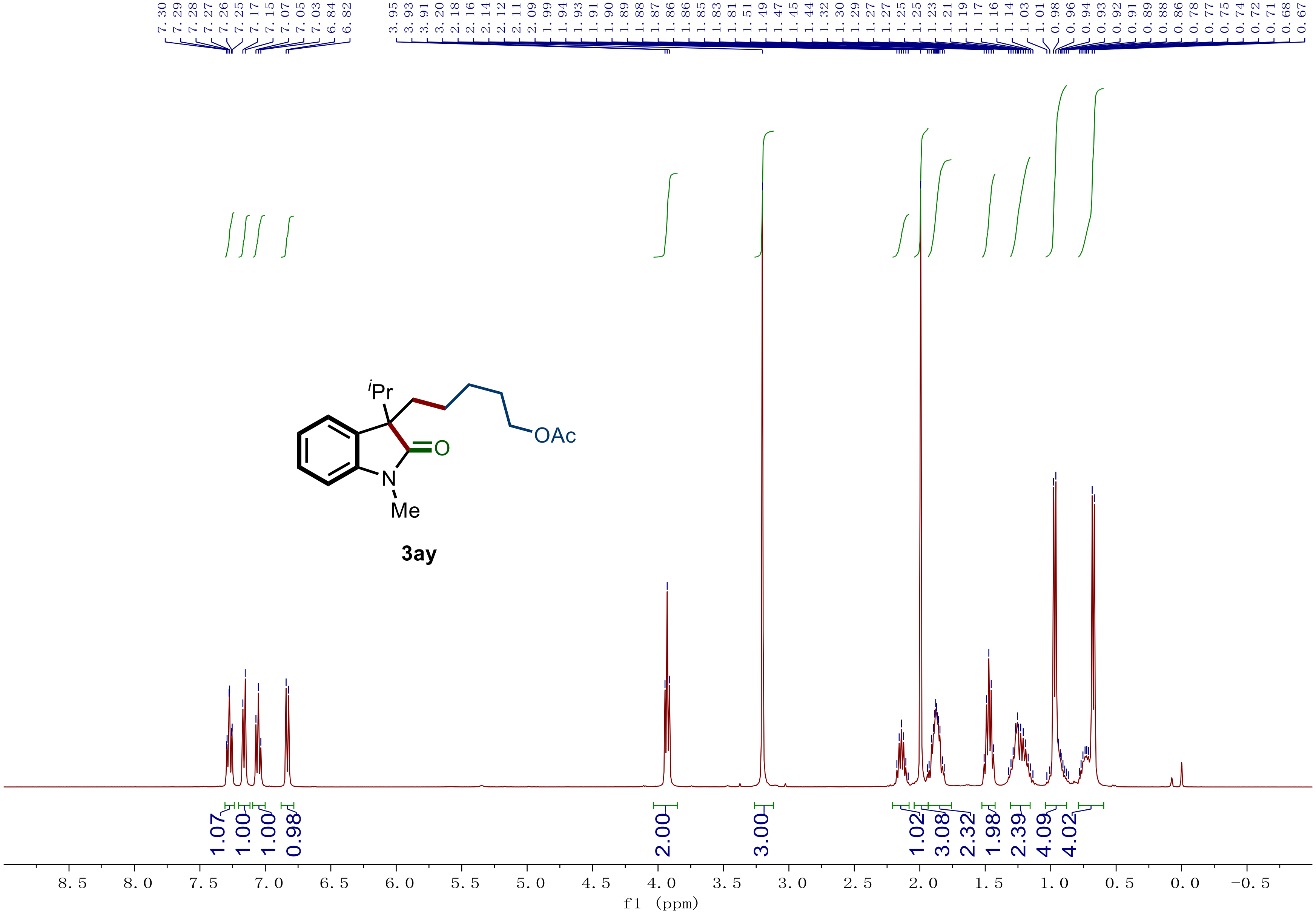

Supplementary Figure 184

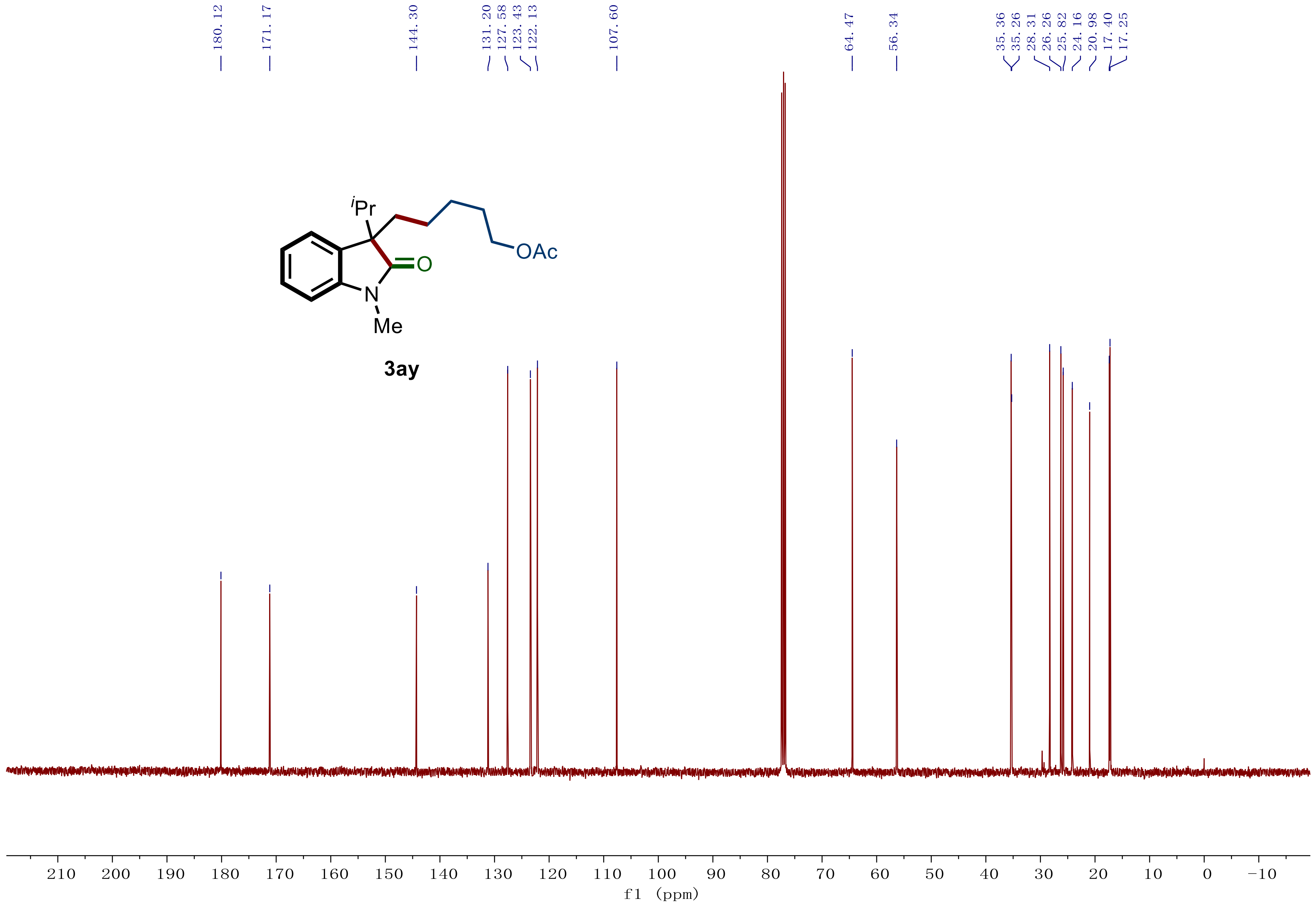

Supplementary Figure 185

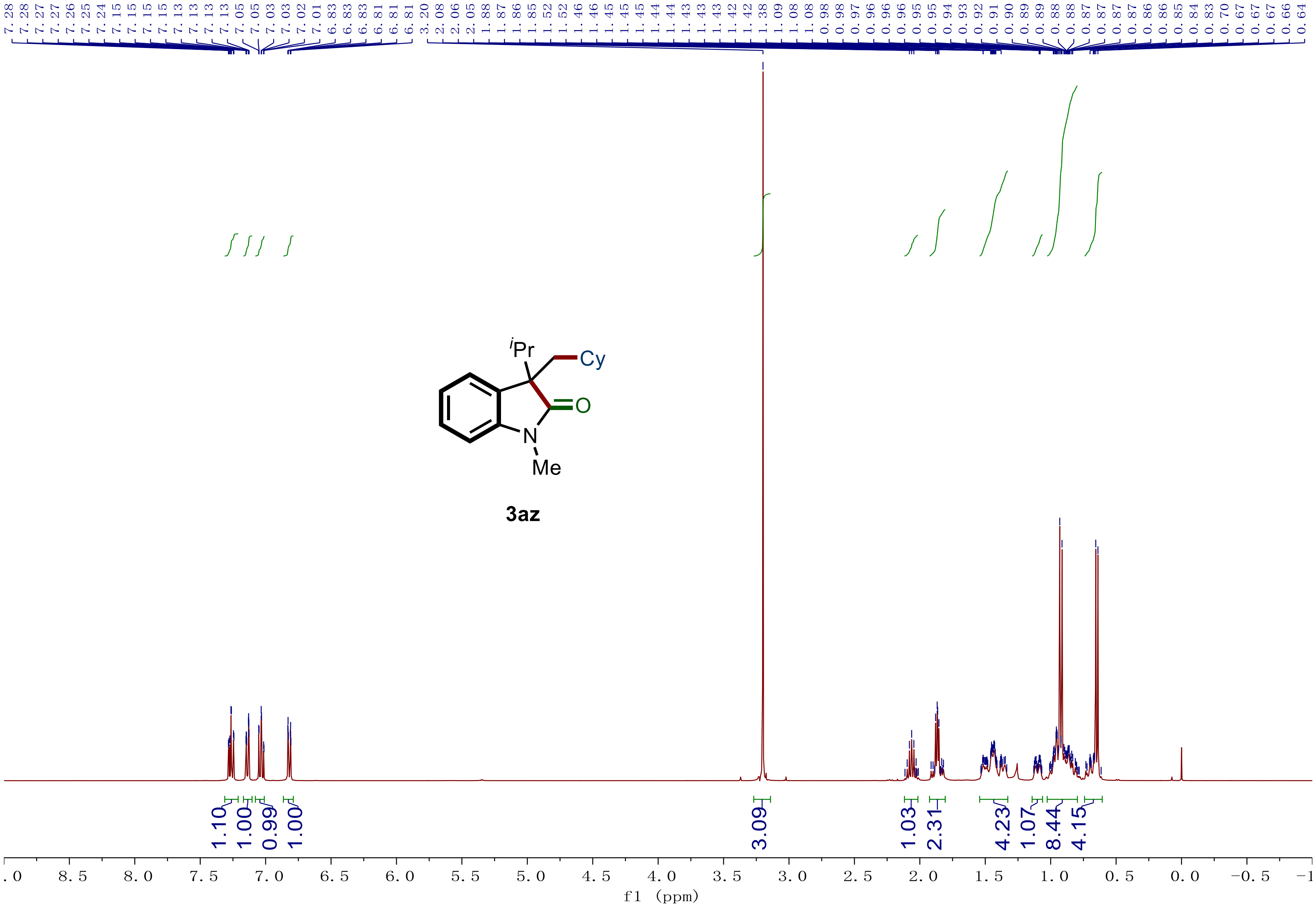

Supplementary Figure 186

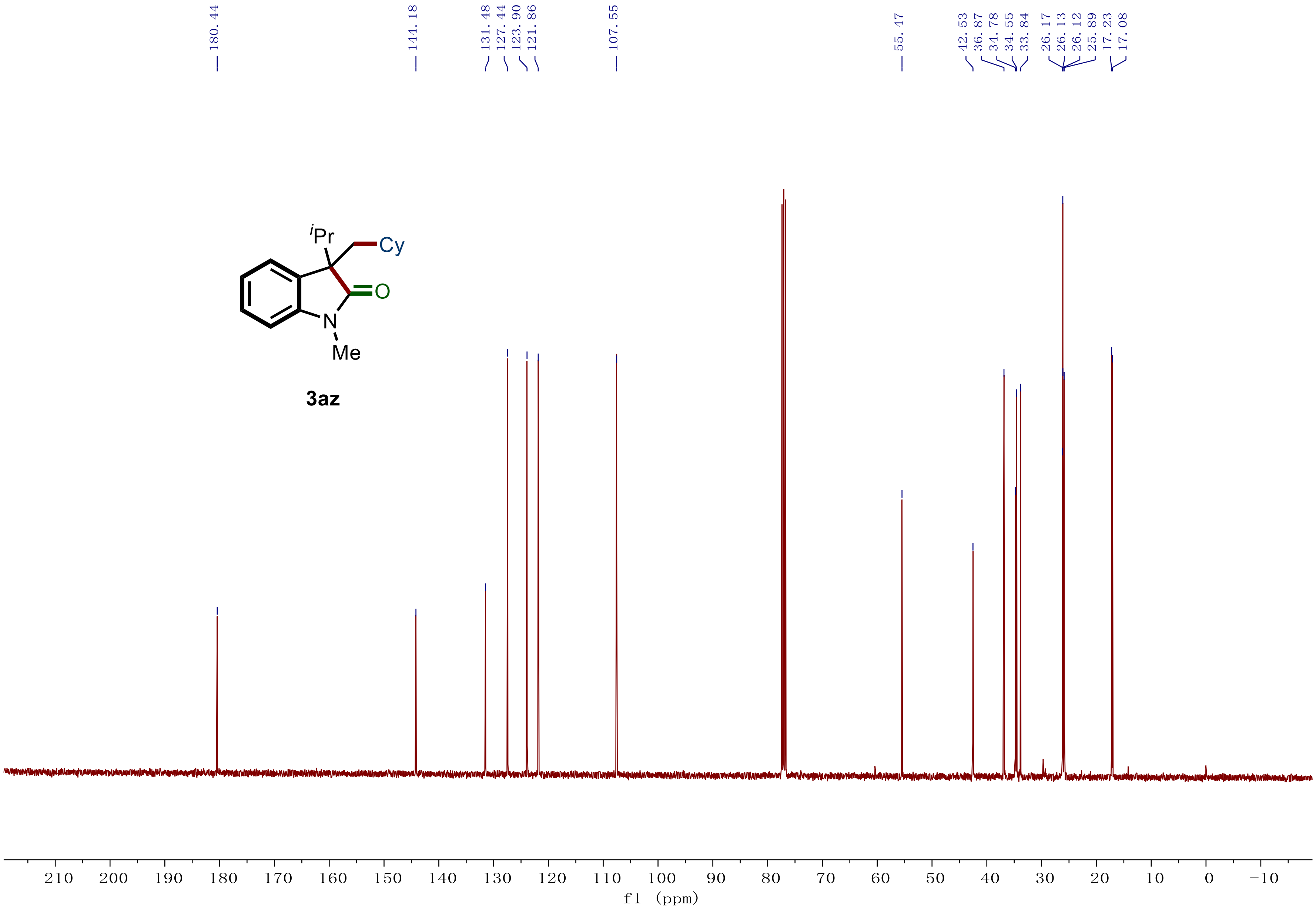

3az

Supplementary Figure 187

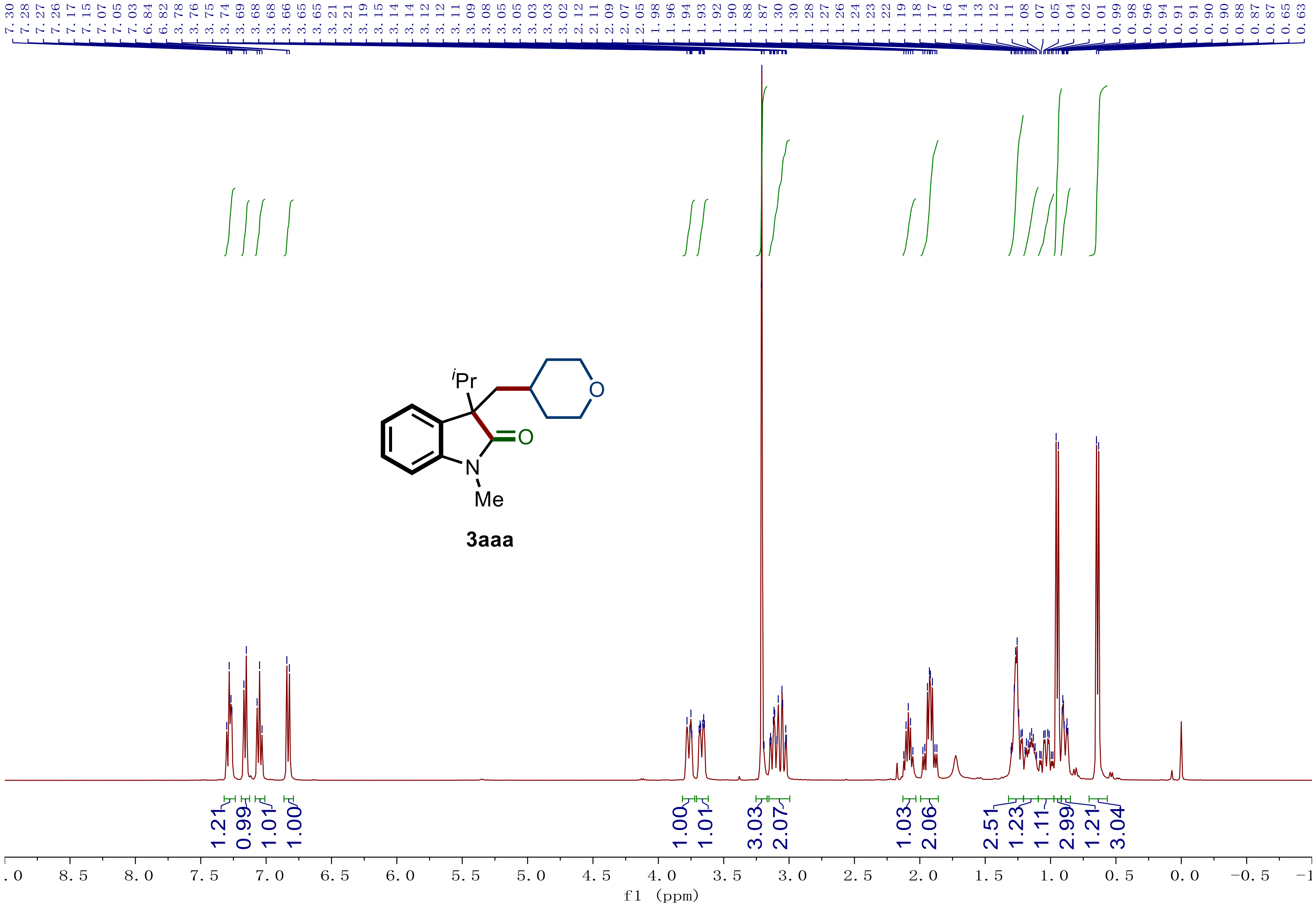

Supplementary Figure 188

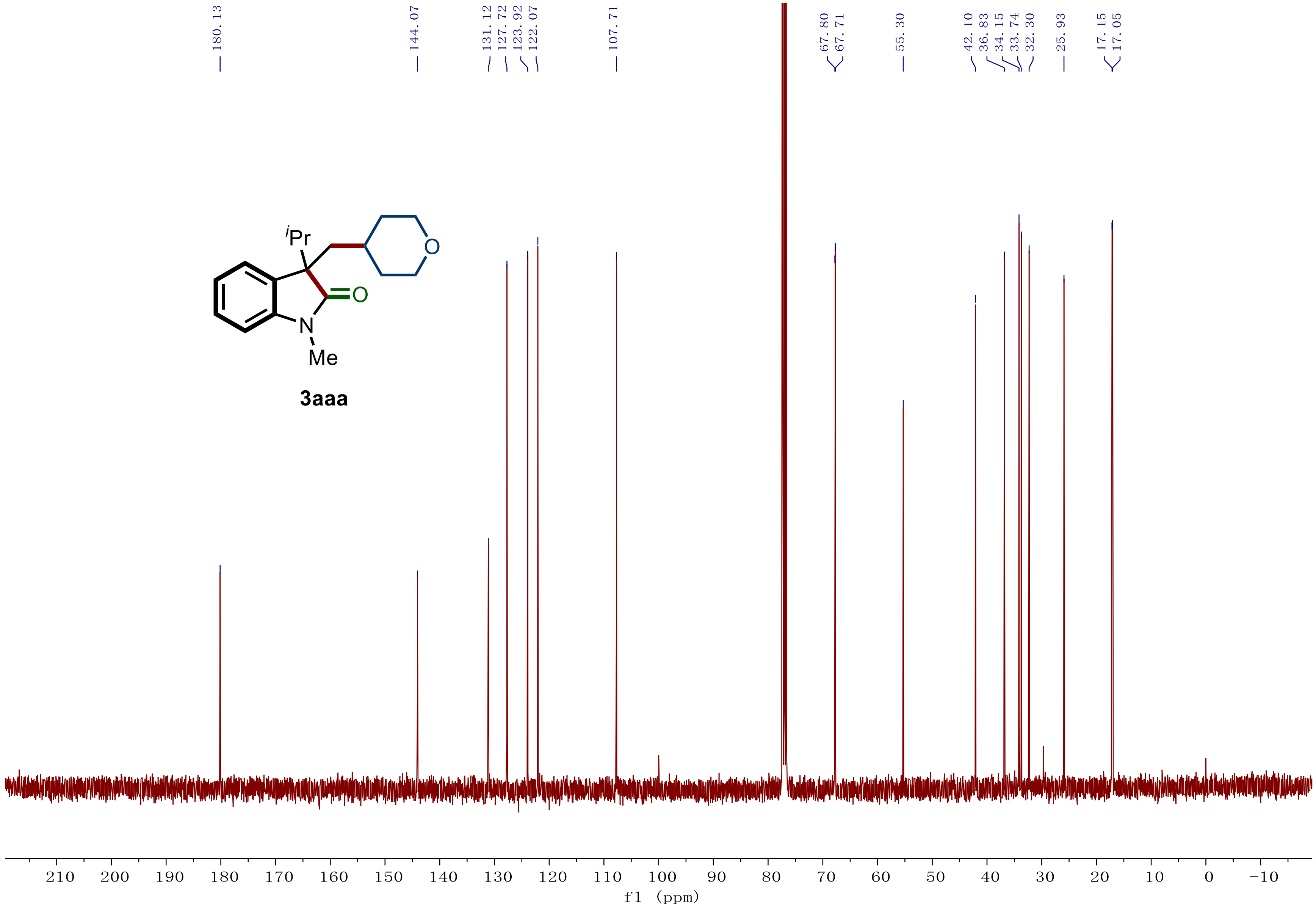

3aaa

Supplementary Figure 189

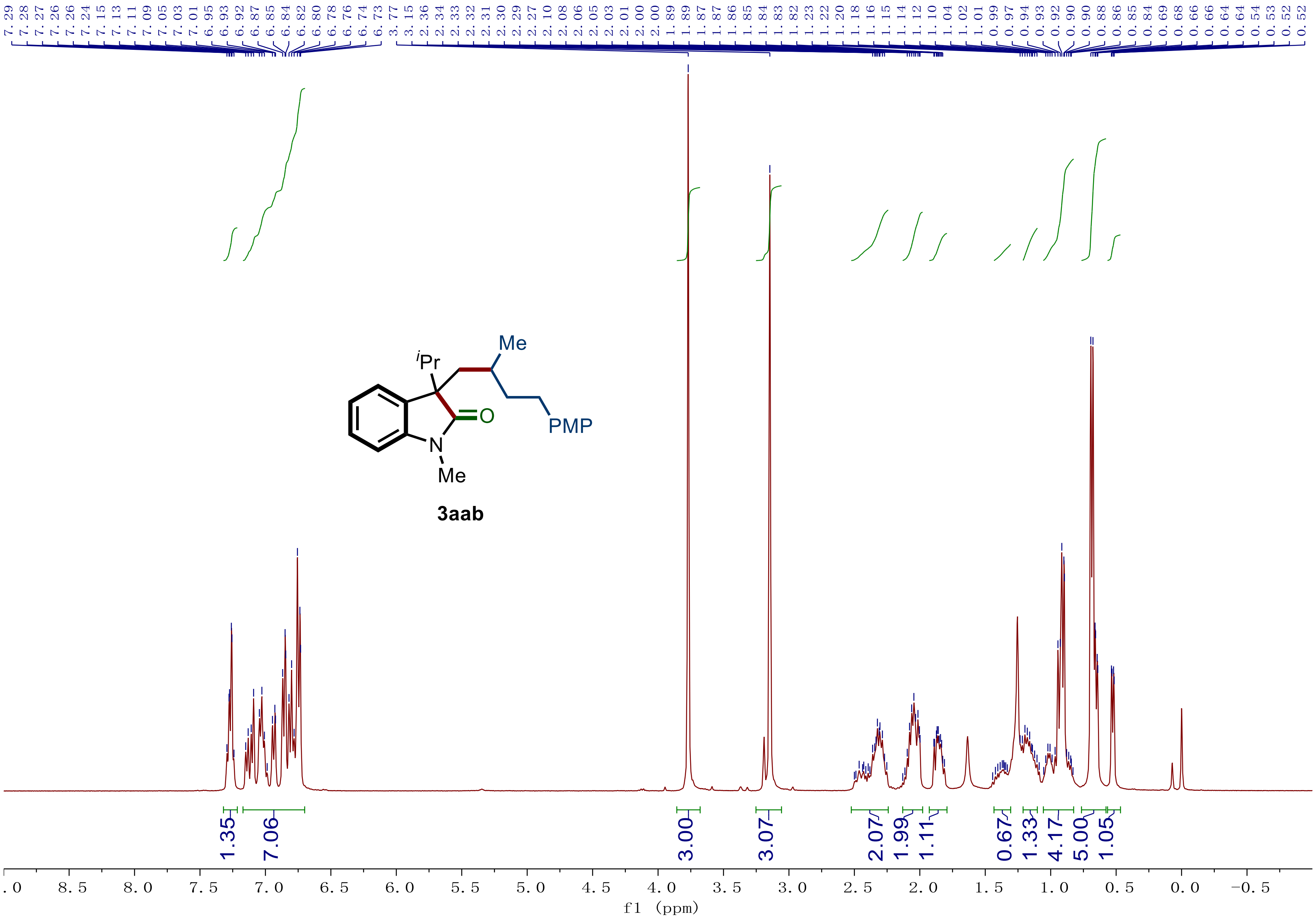

Supplementary Figure 190

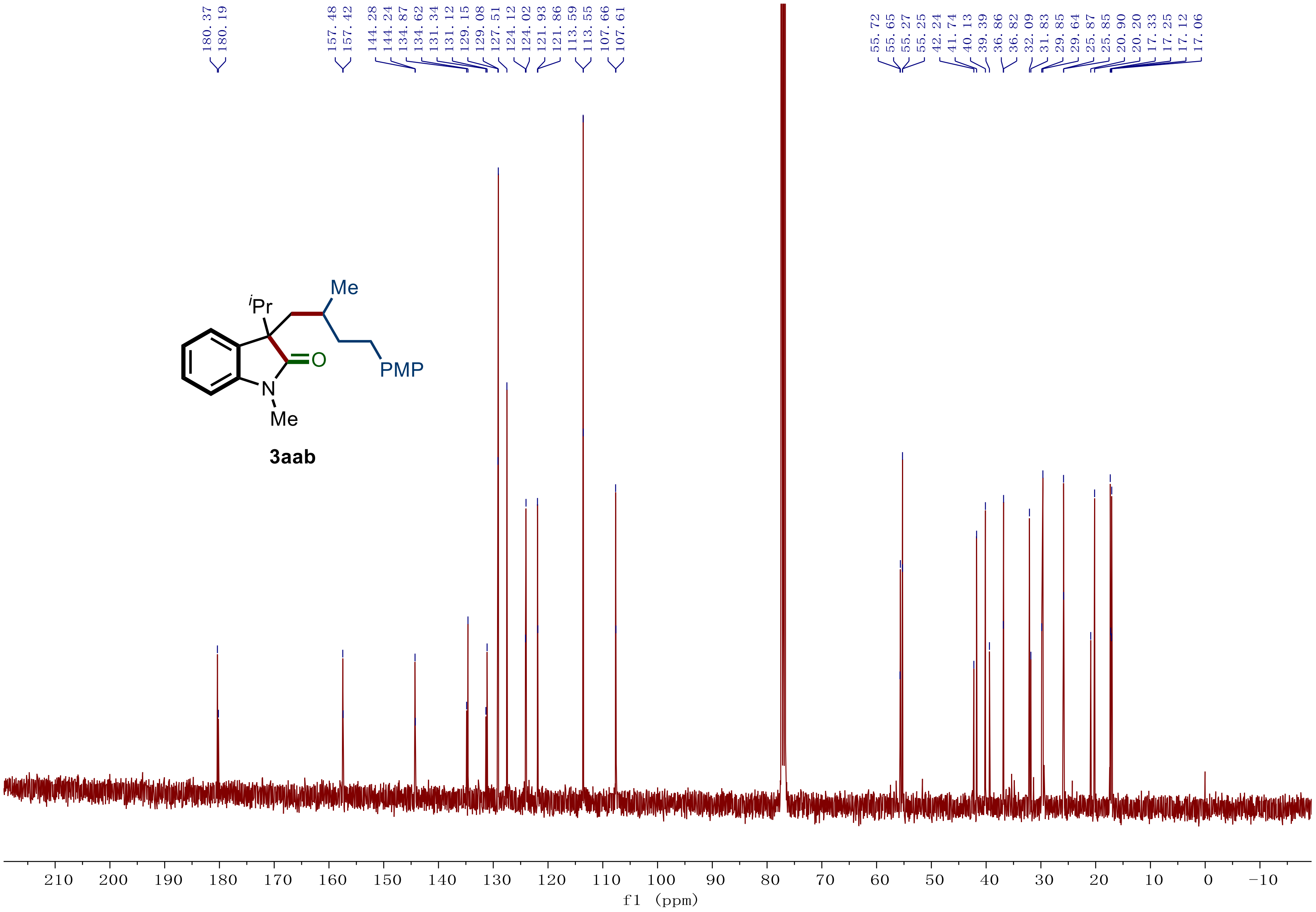

Supplementary Figure 191

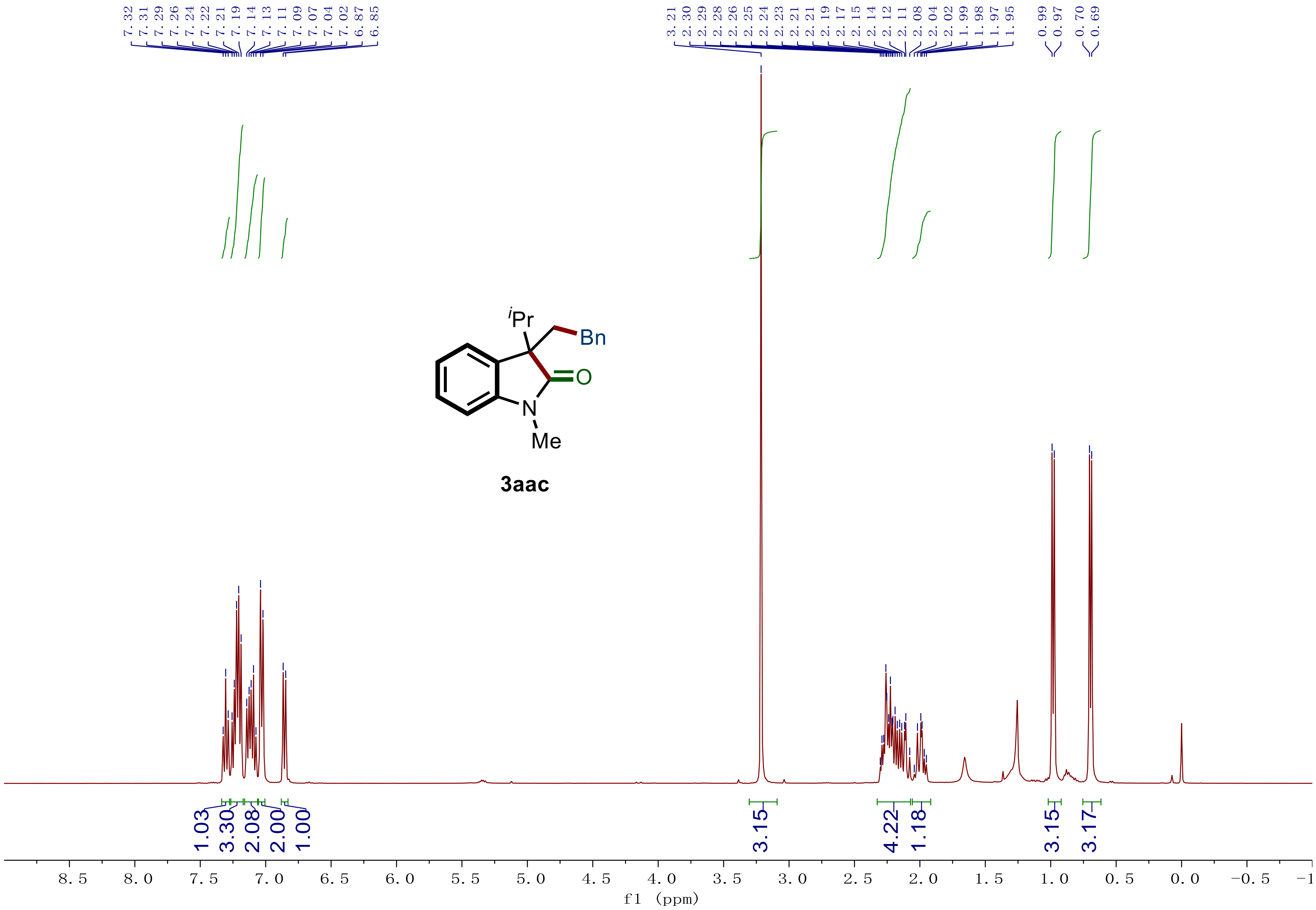

Supplementary Figure 192

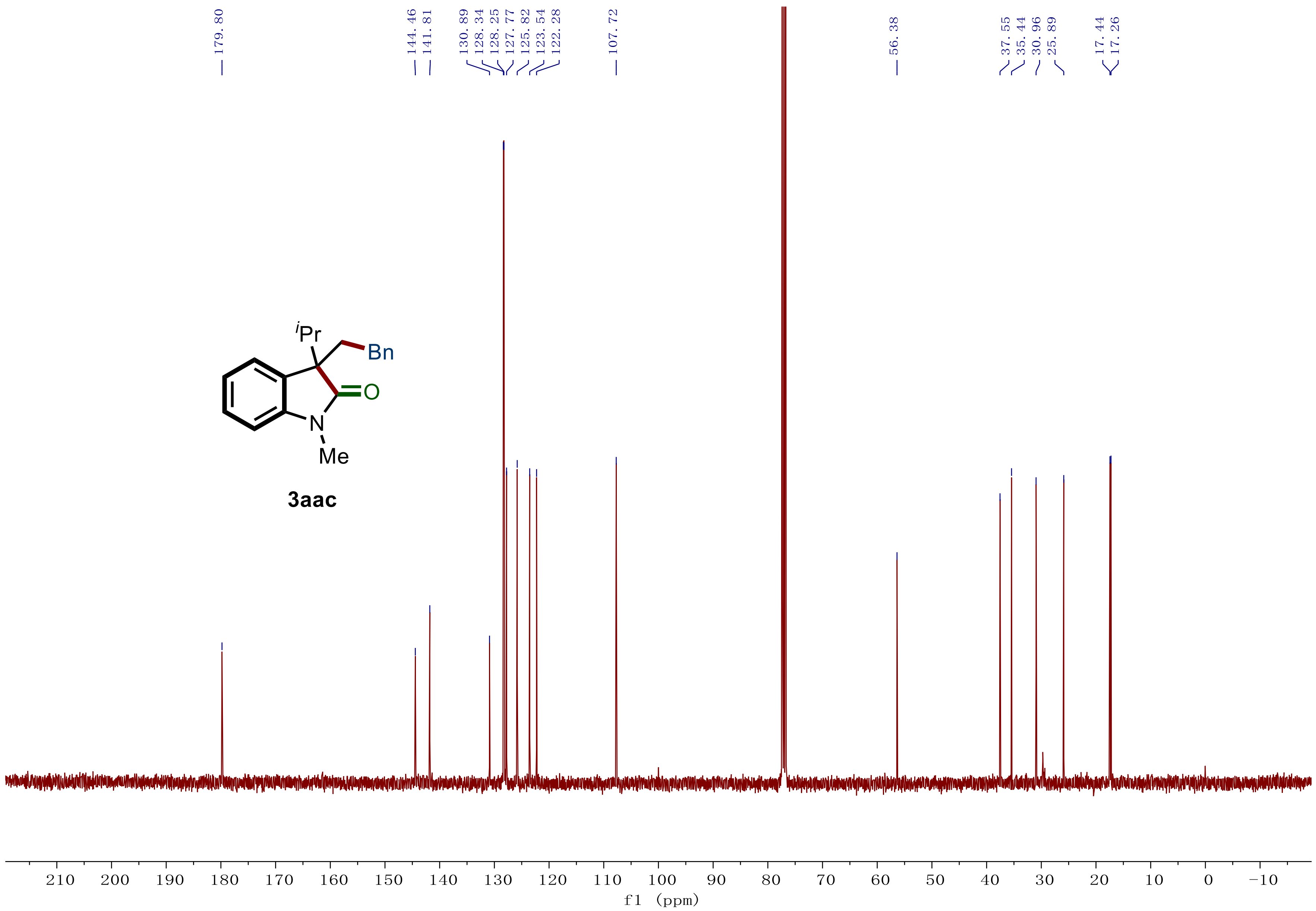

Supplementary Figure 193

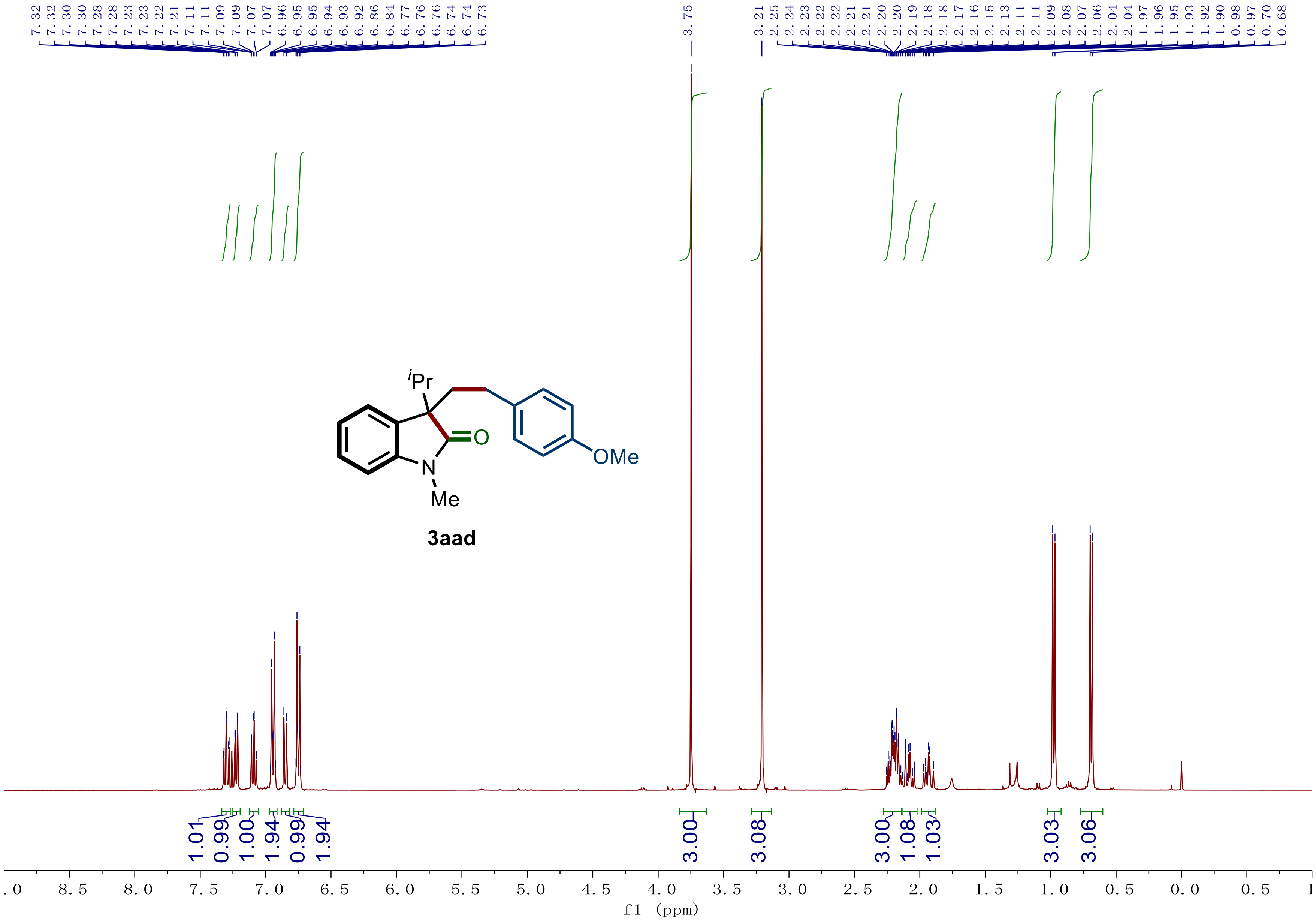

3aad

Supplementary Figure 194

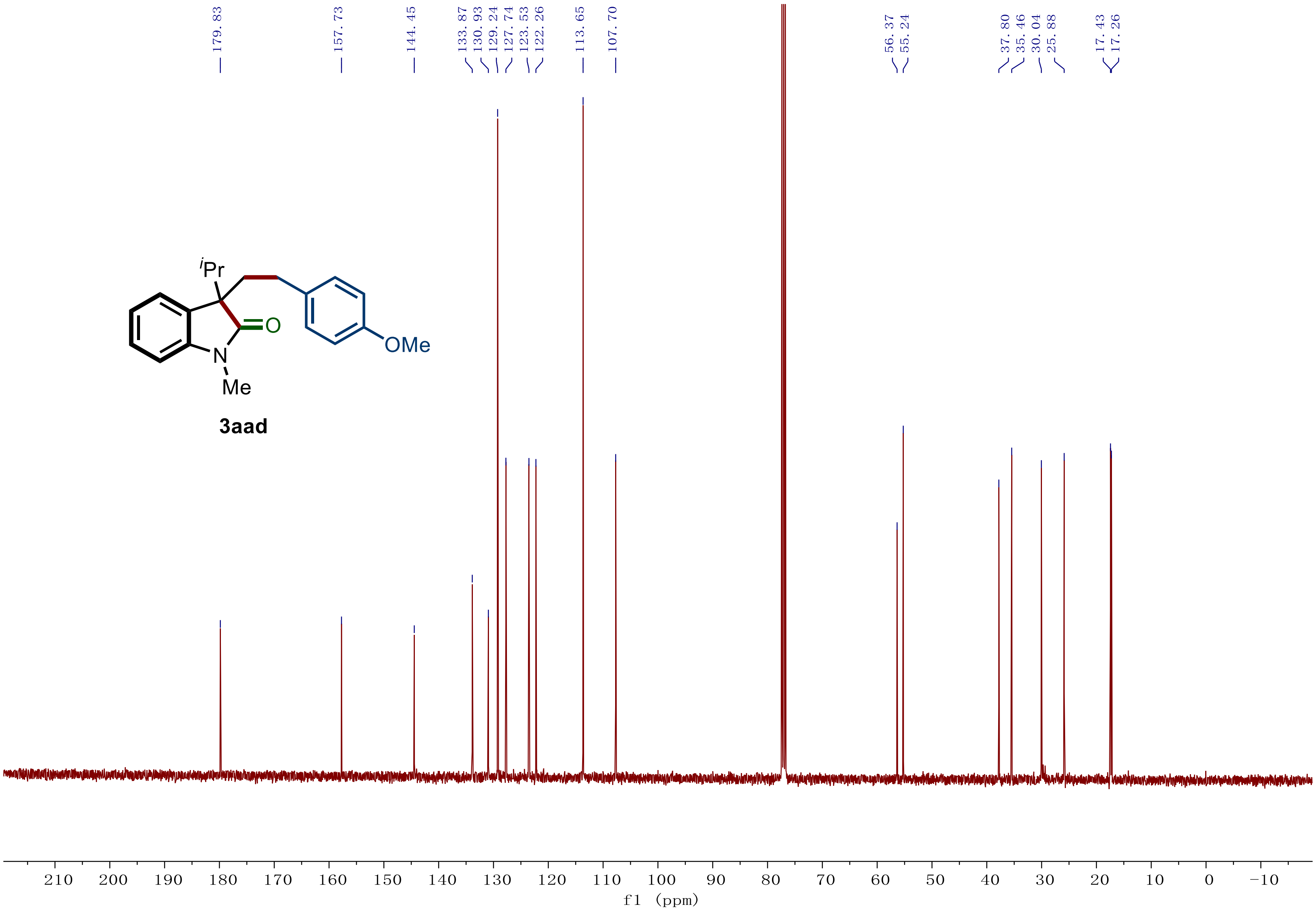

Supplementary Figure 195

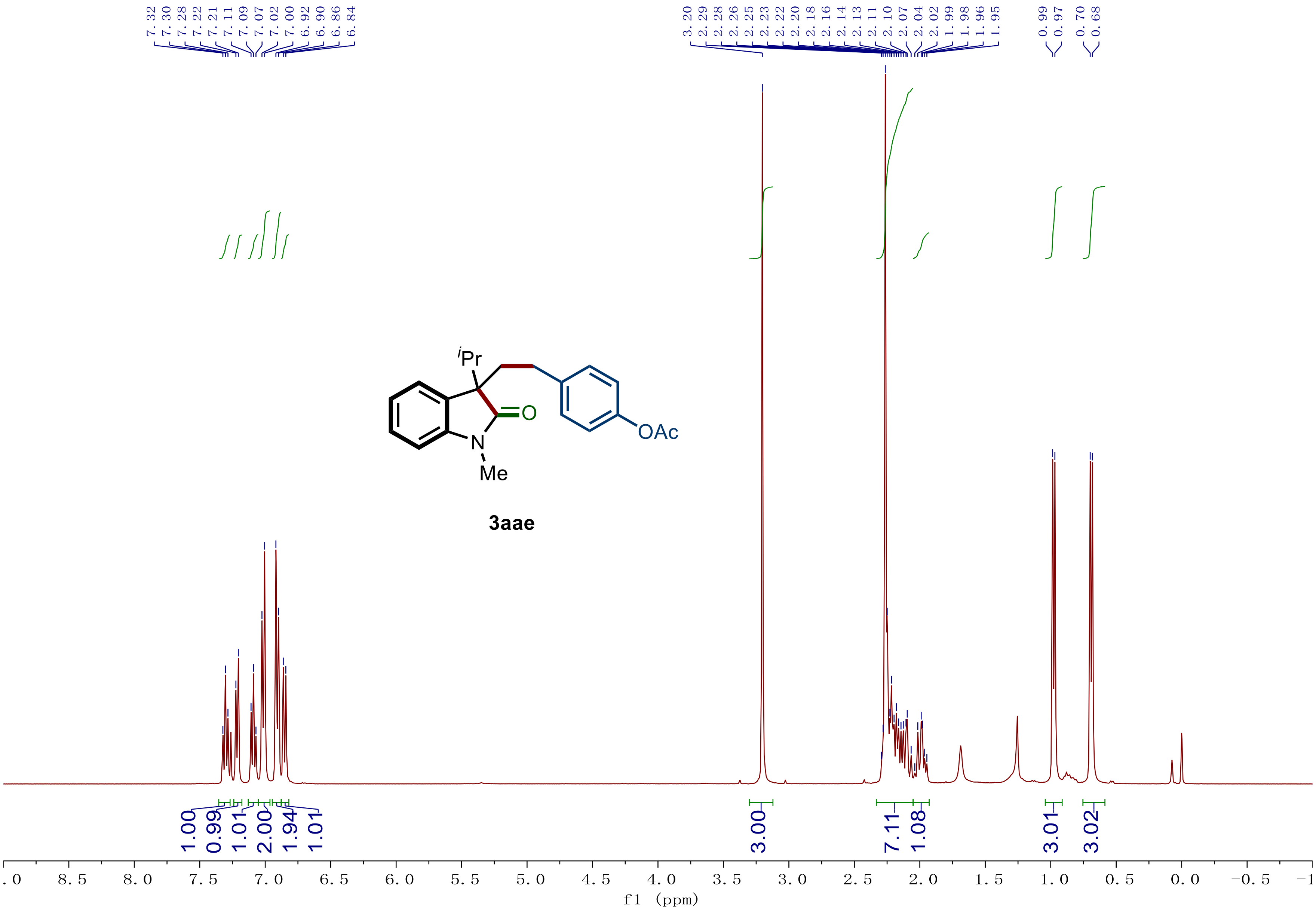

Supplementary Figure 196

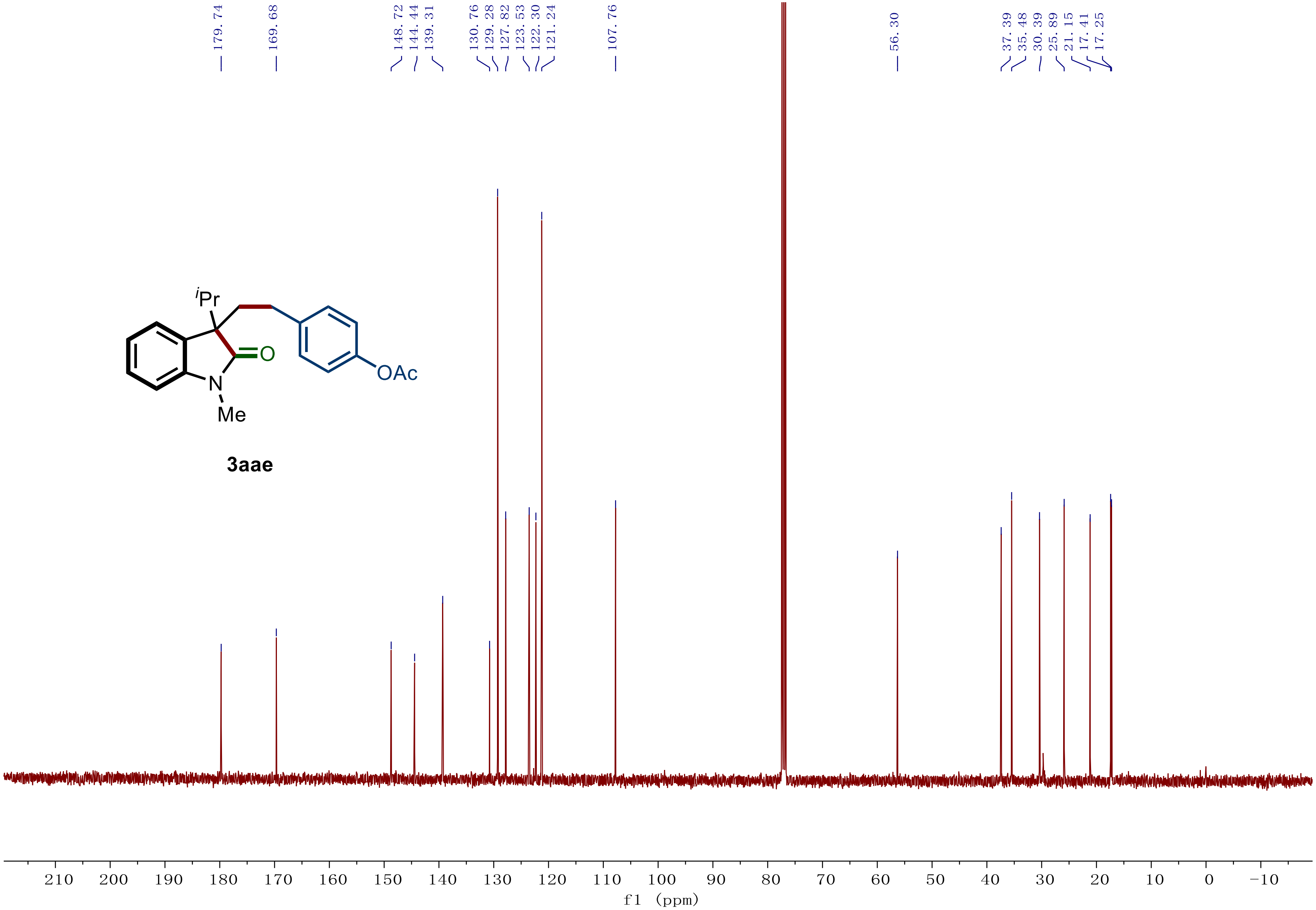

Supplementary Figure 197

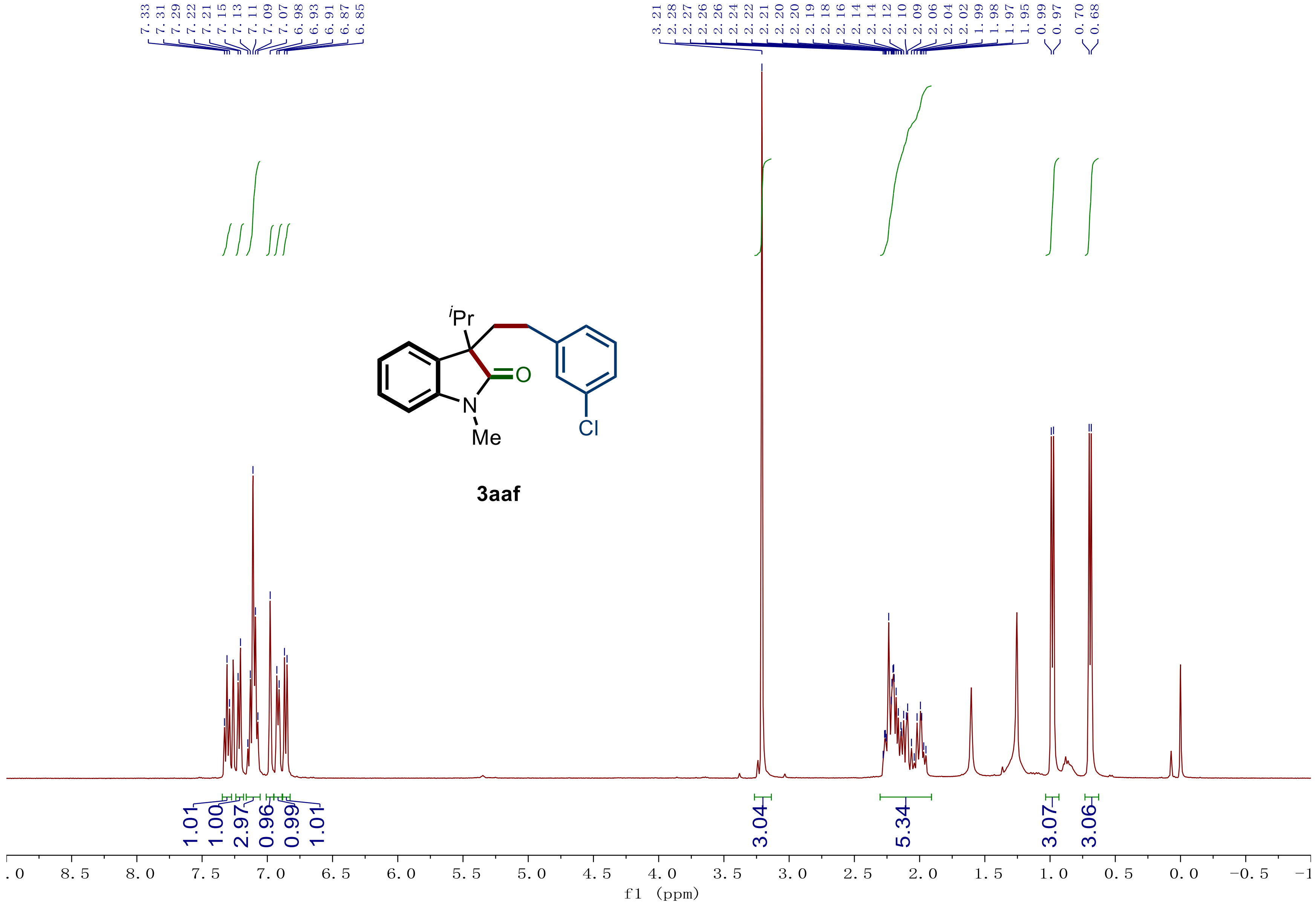

Supplementary Figure 198

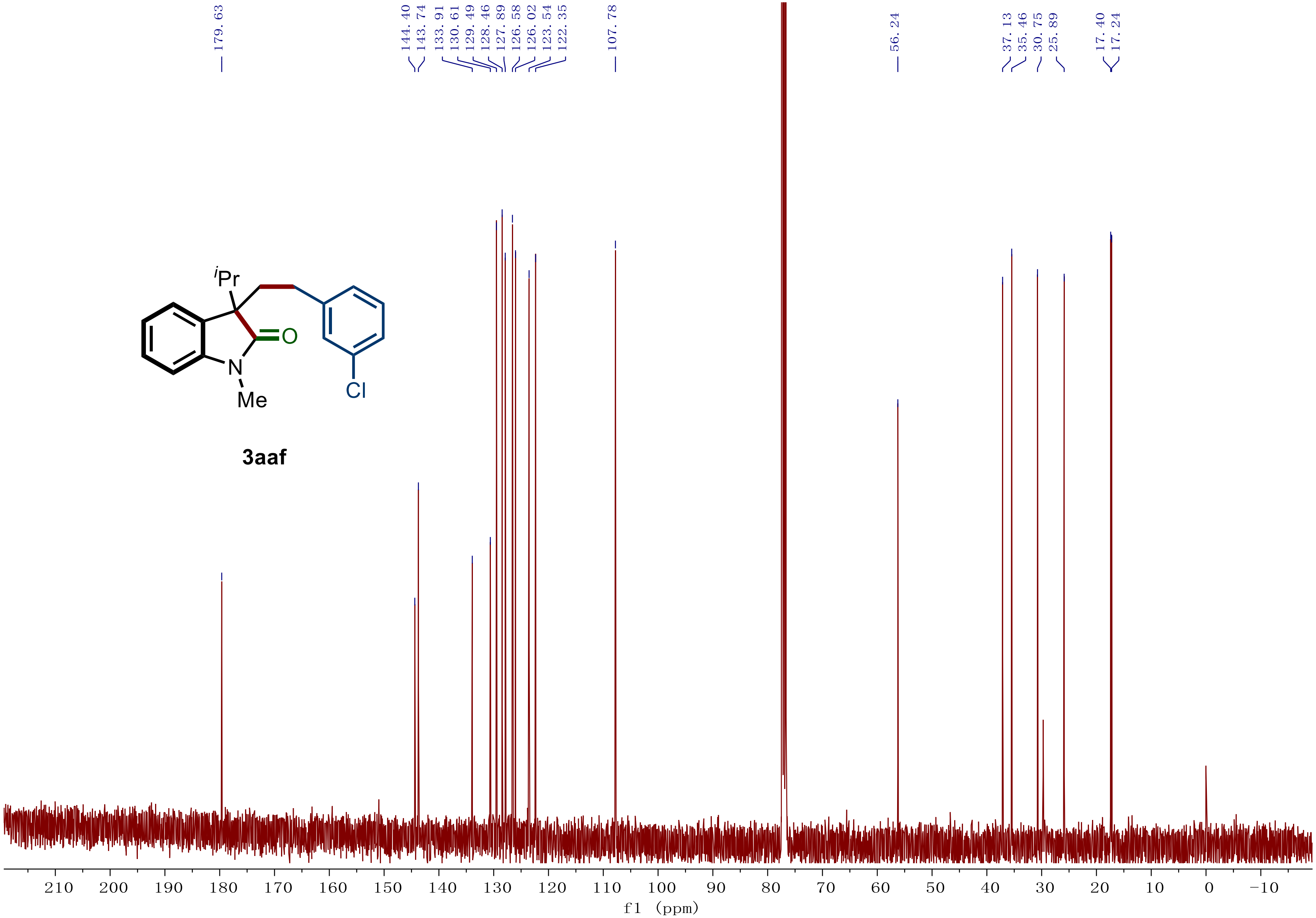

Supplementary Figure 199

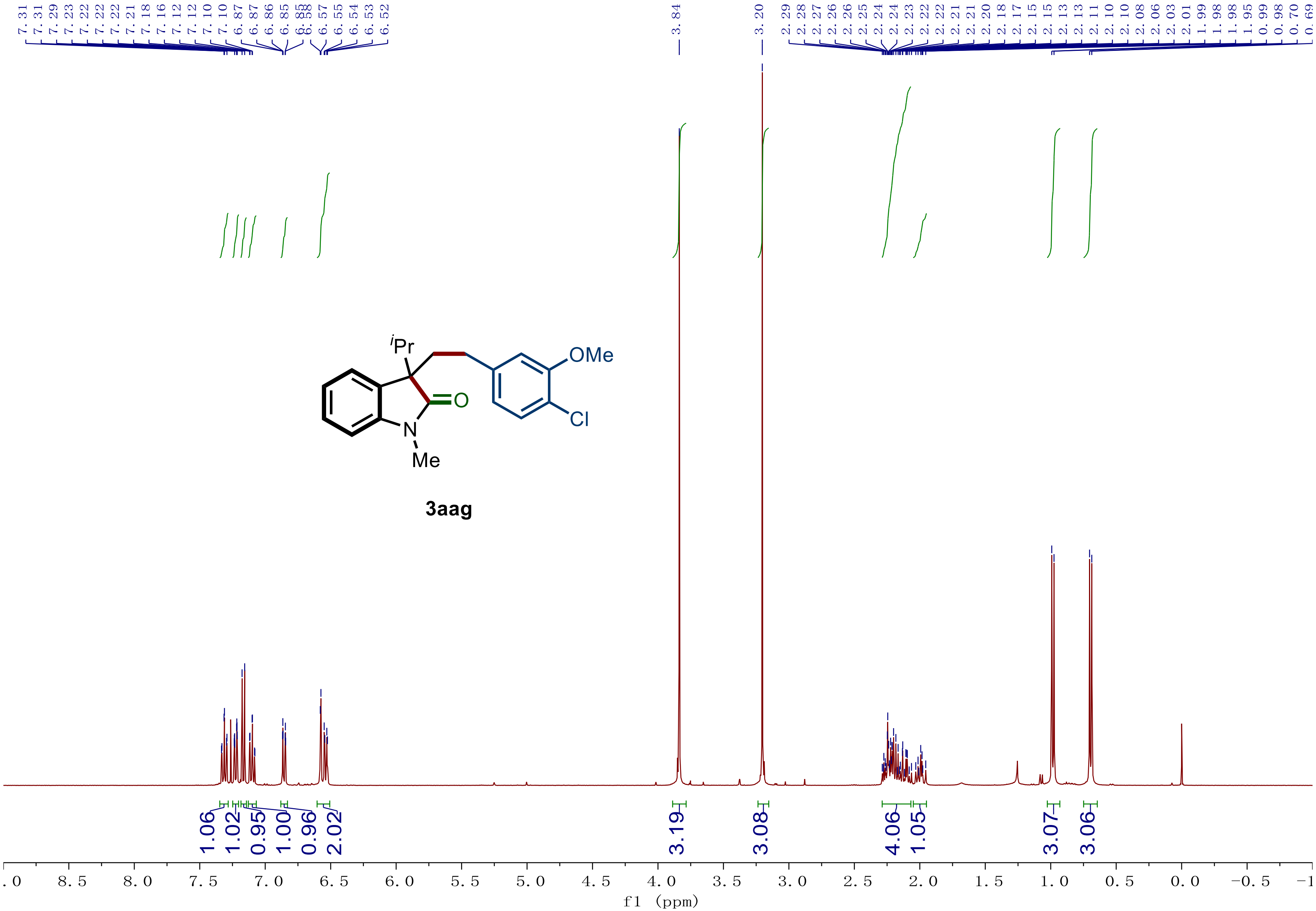

Supplementary Figure 200

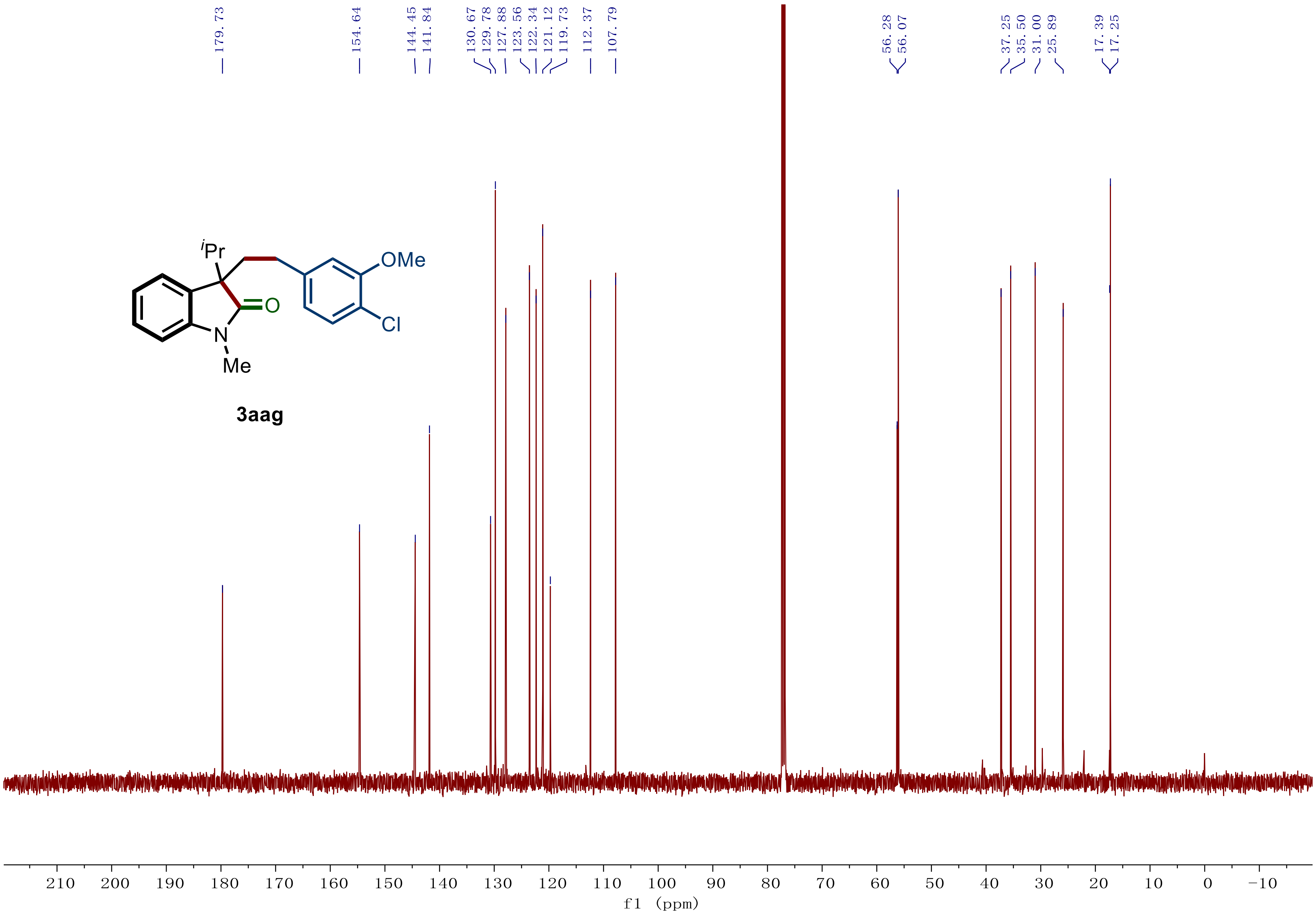

Supplementary Figure 201

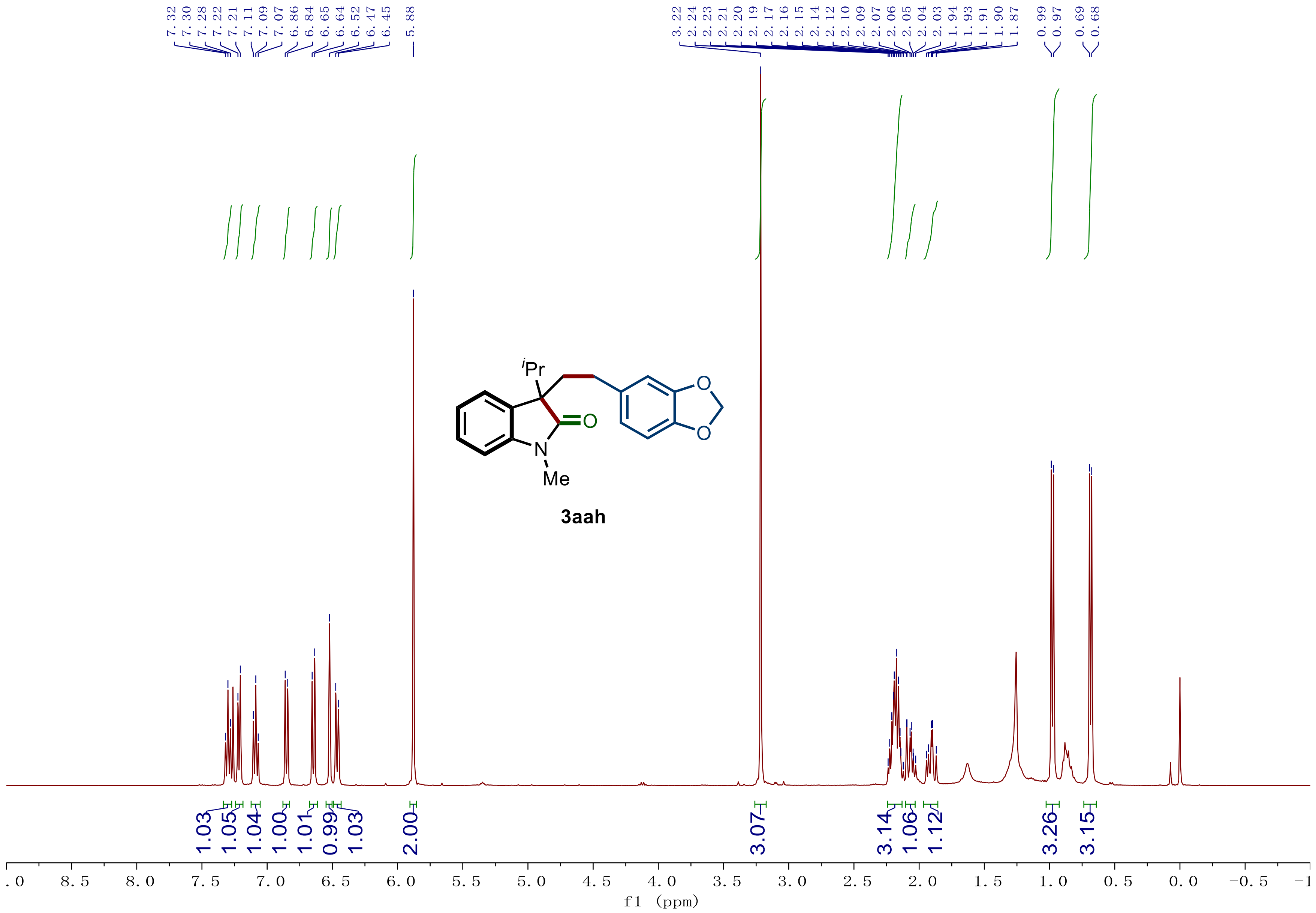

Supplementary Figure 202

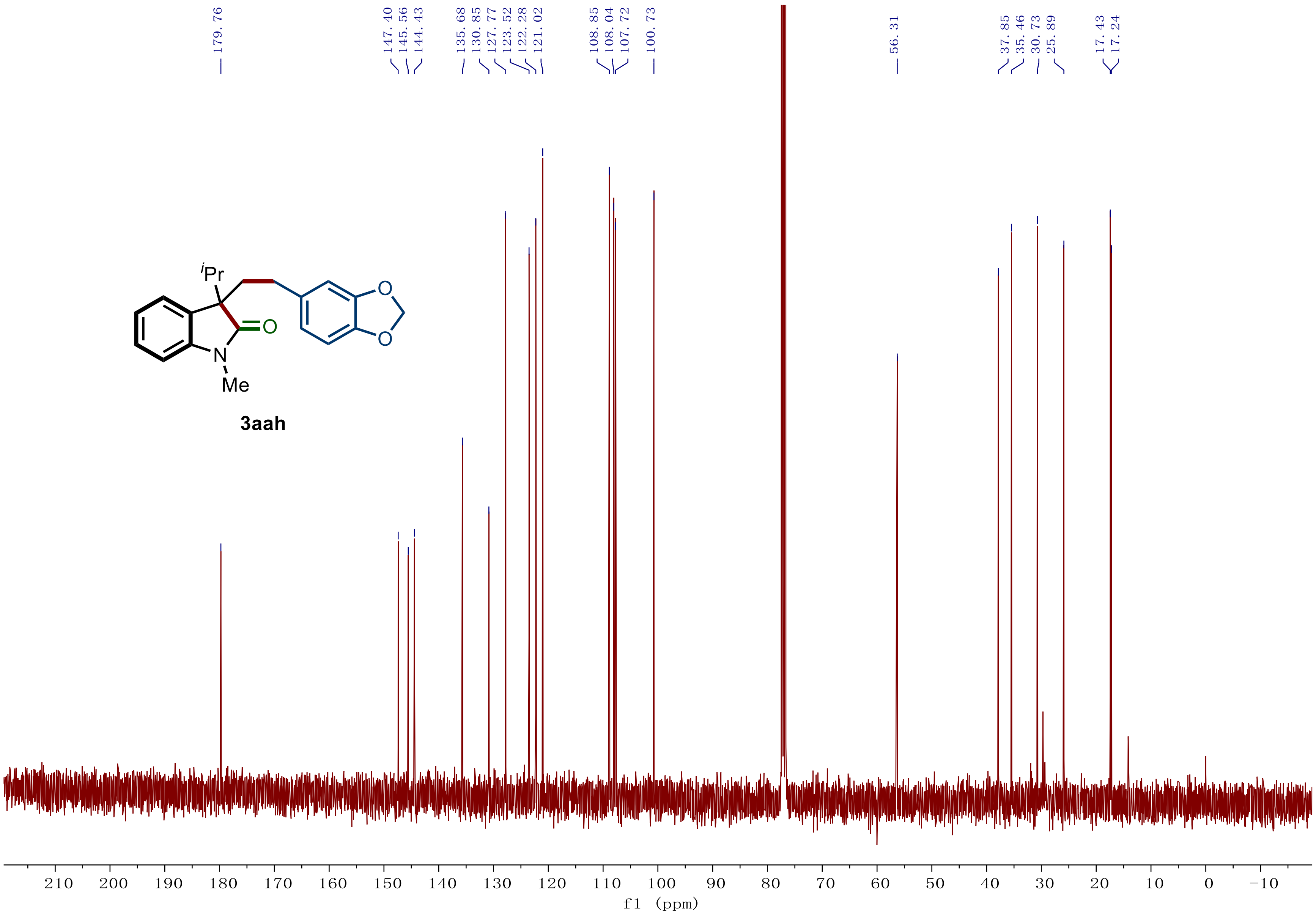

Supplementary Figure 203

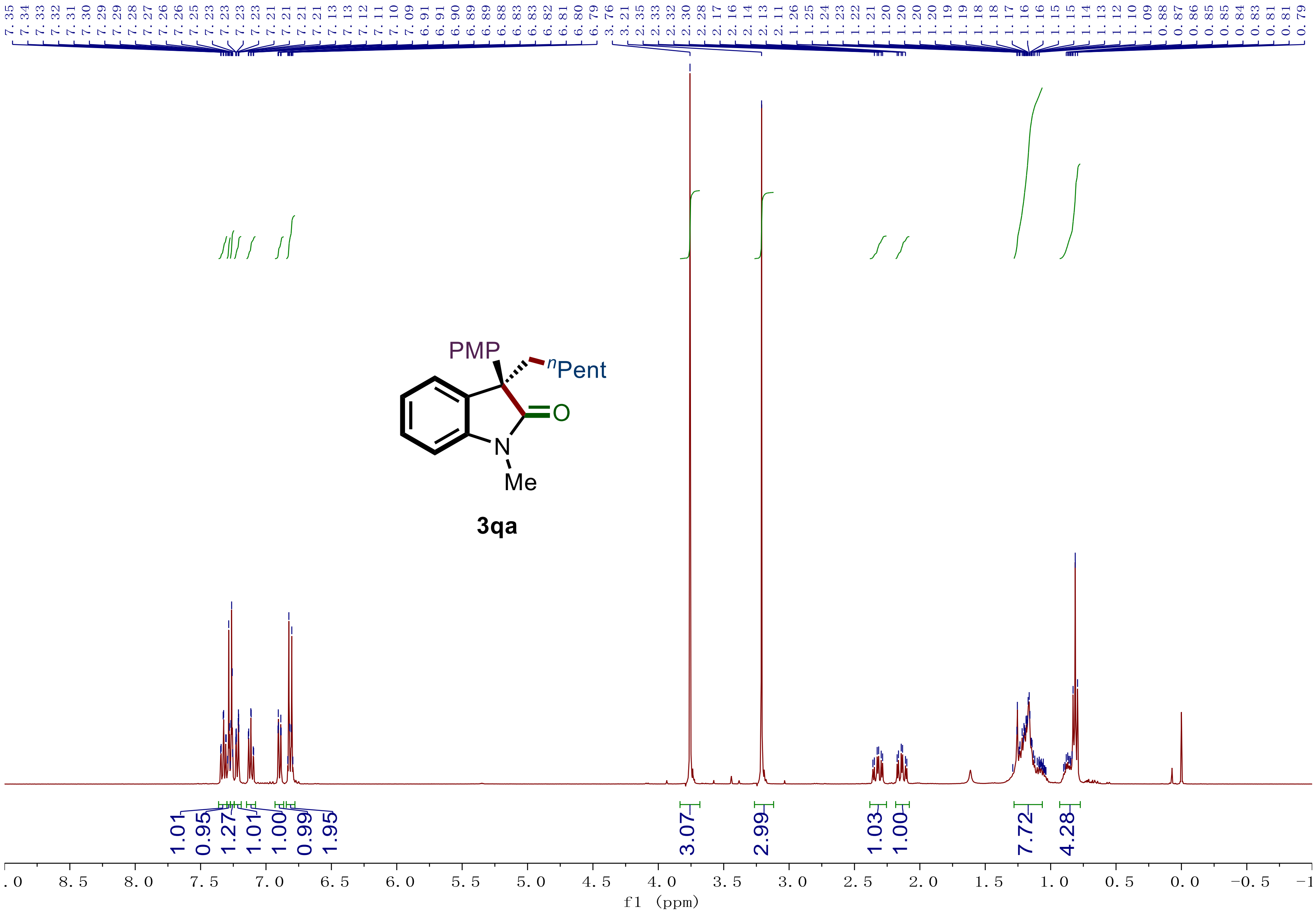

Supplementary Figure 204

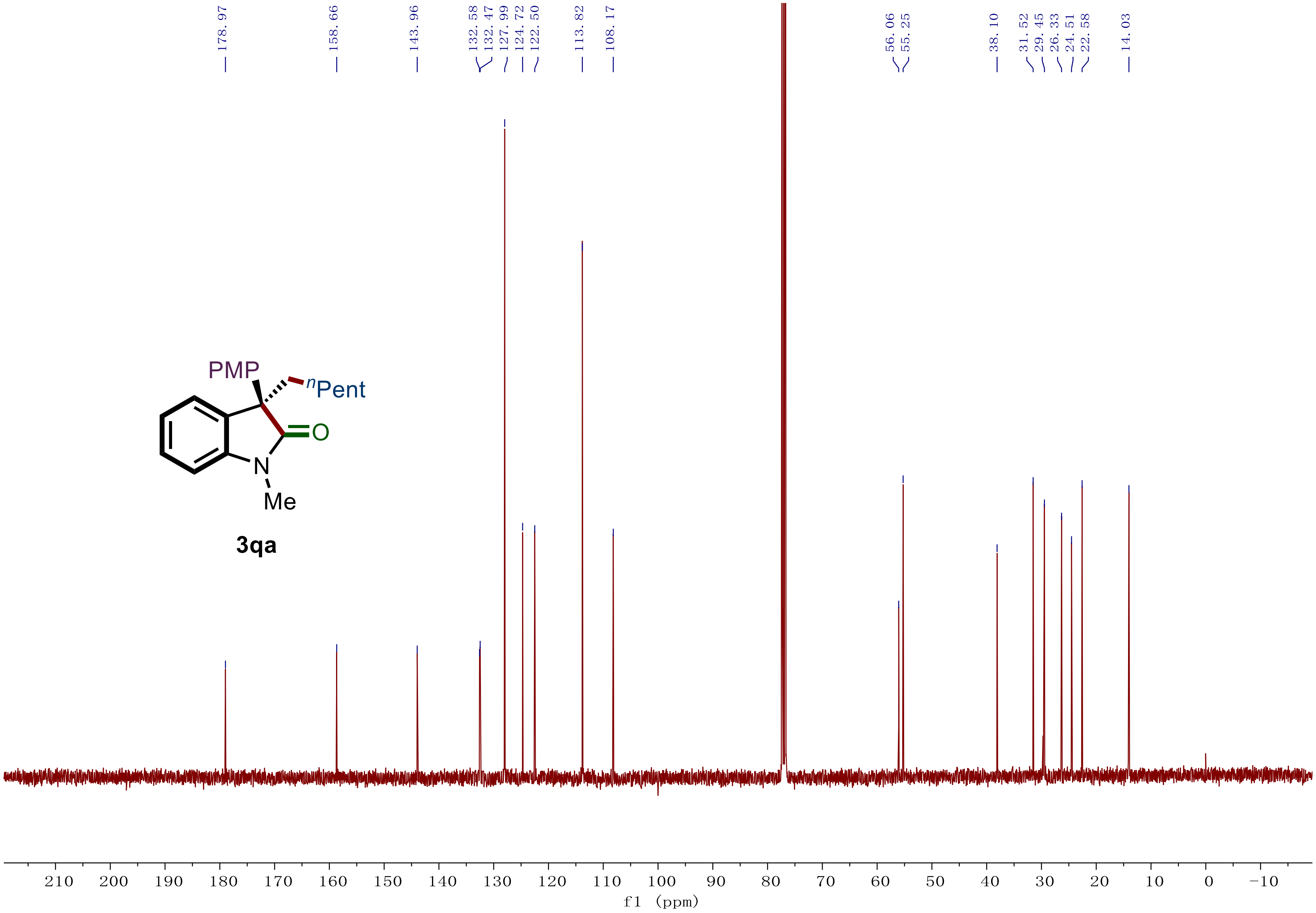

Supplementary Figure 205

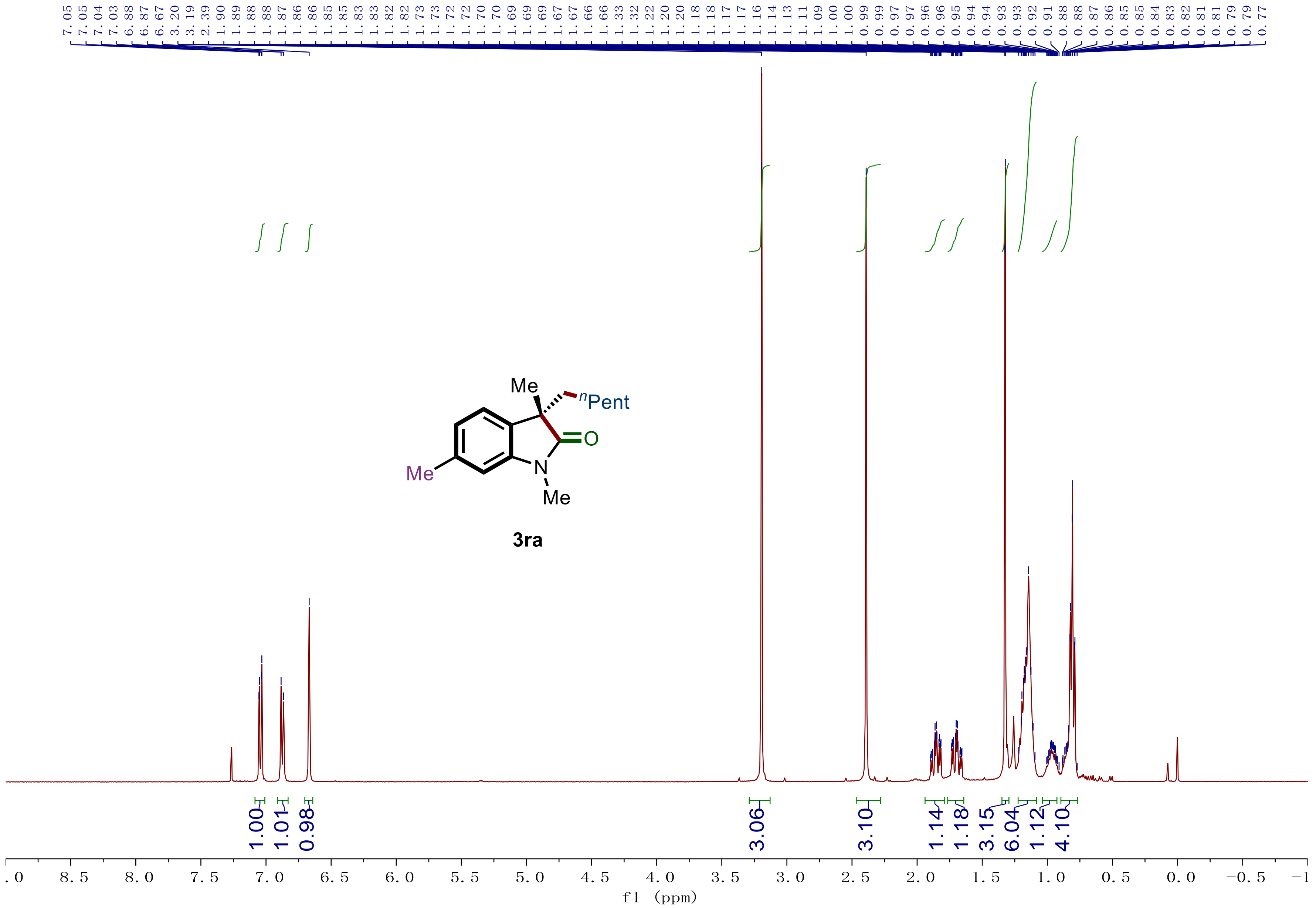

Supplementary Figure 206

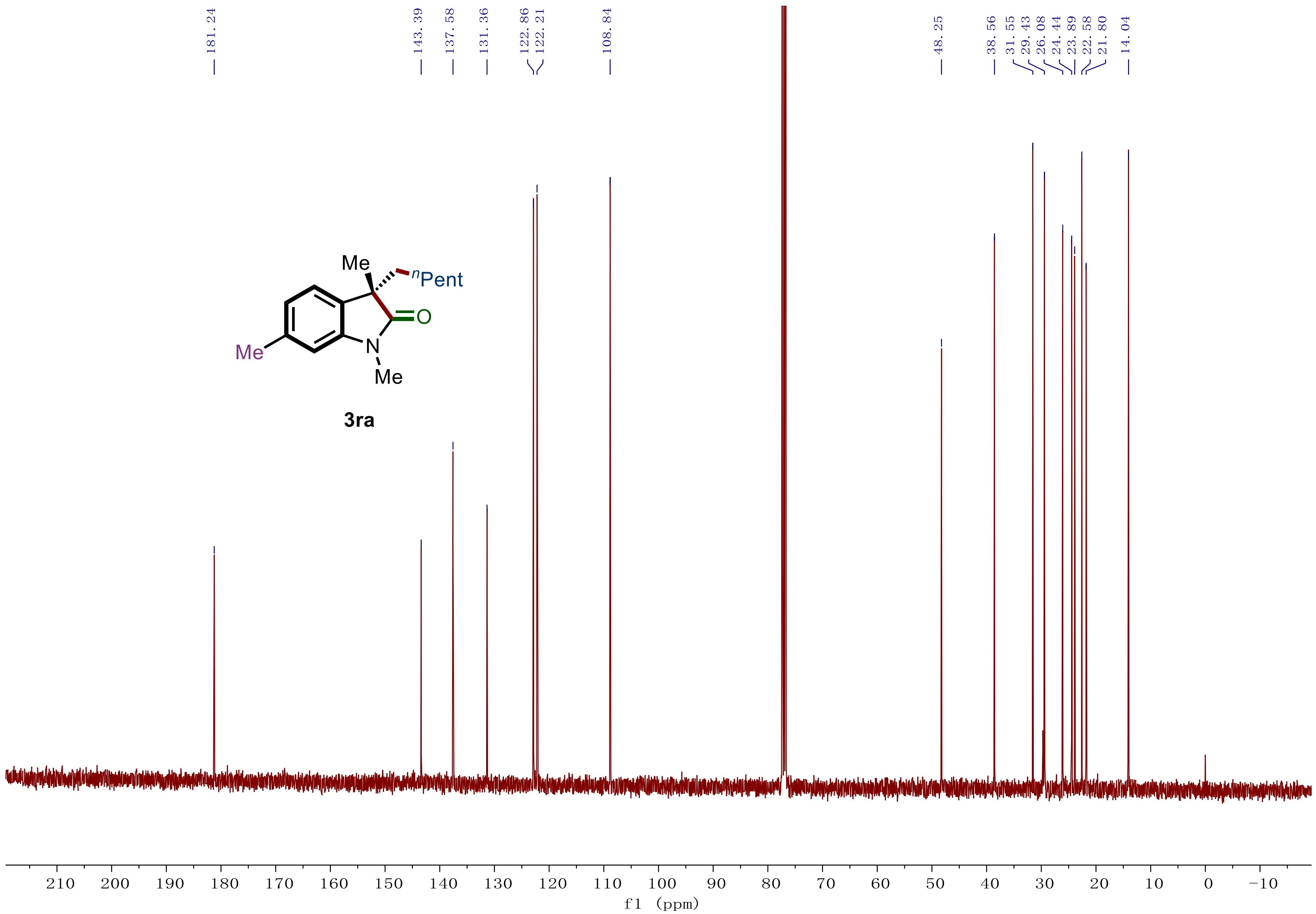

Supplementary Figure 207

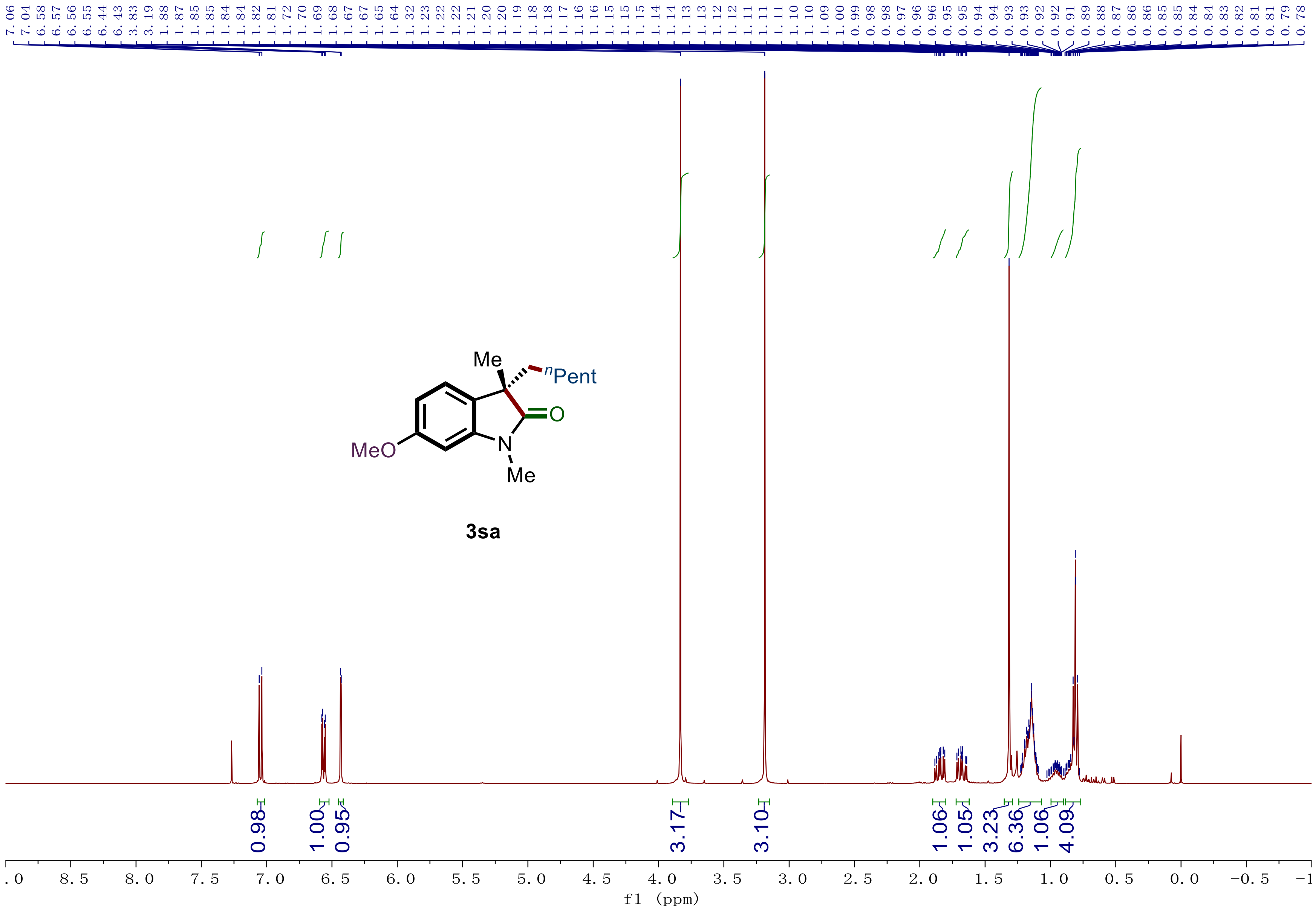

Supplementary Figure 208

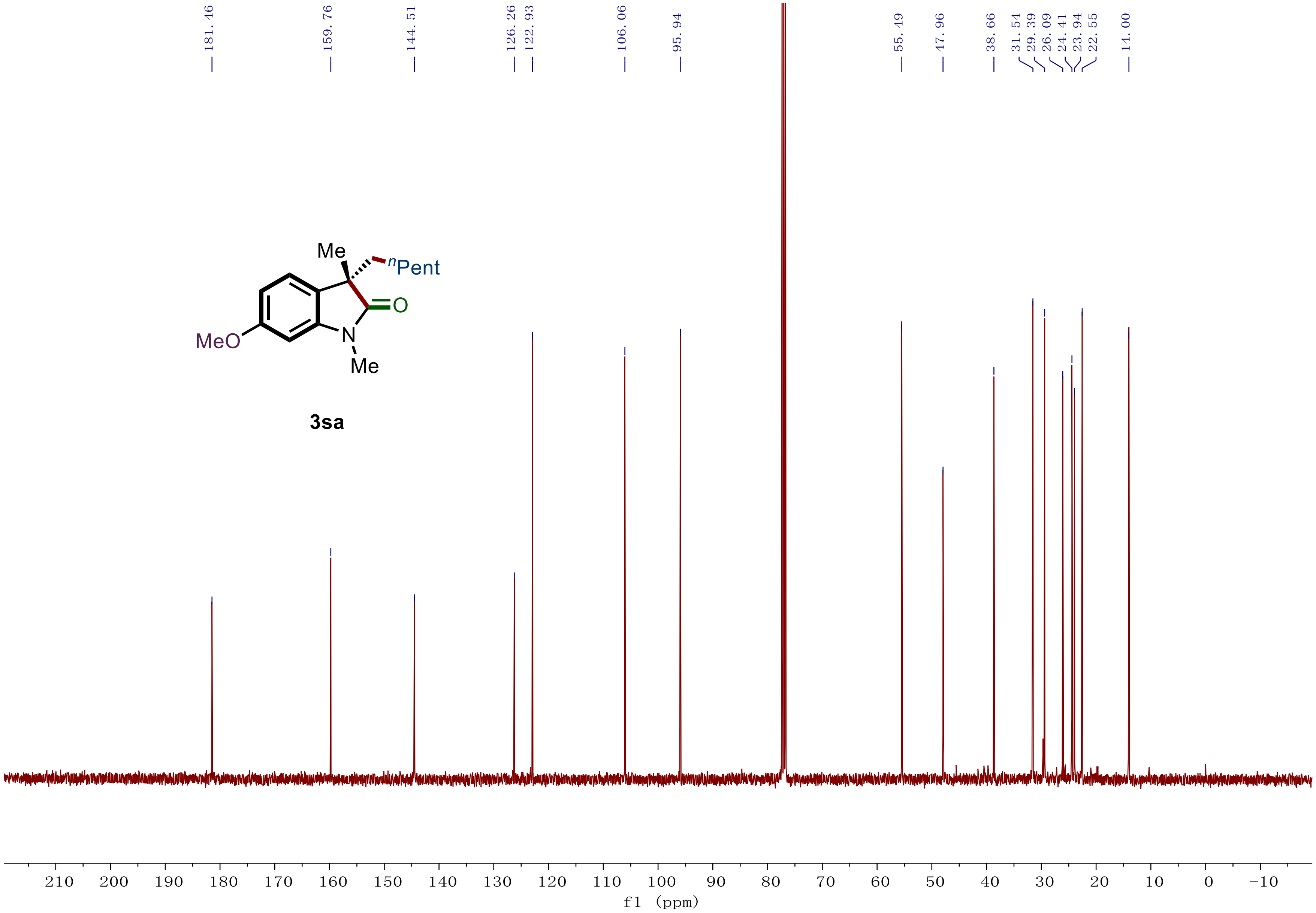

3sa

Supplementary Figure 209

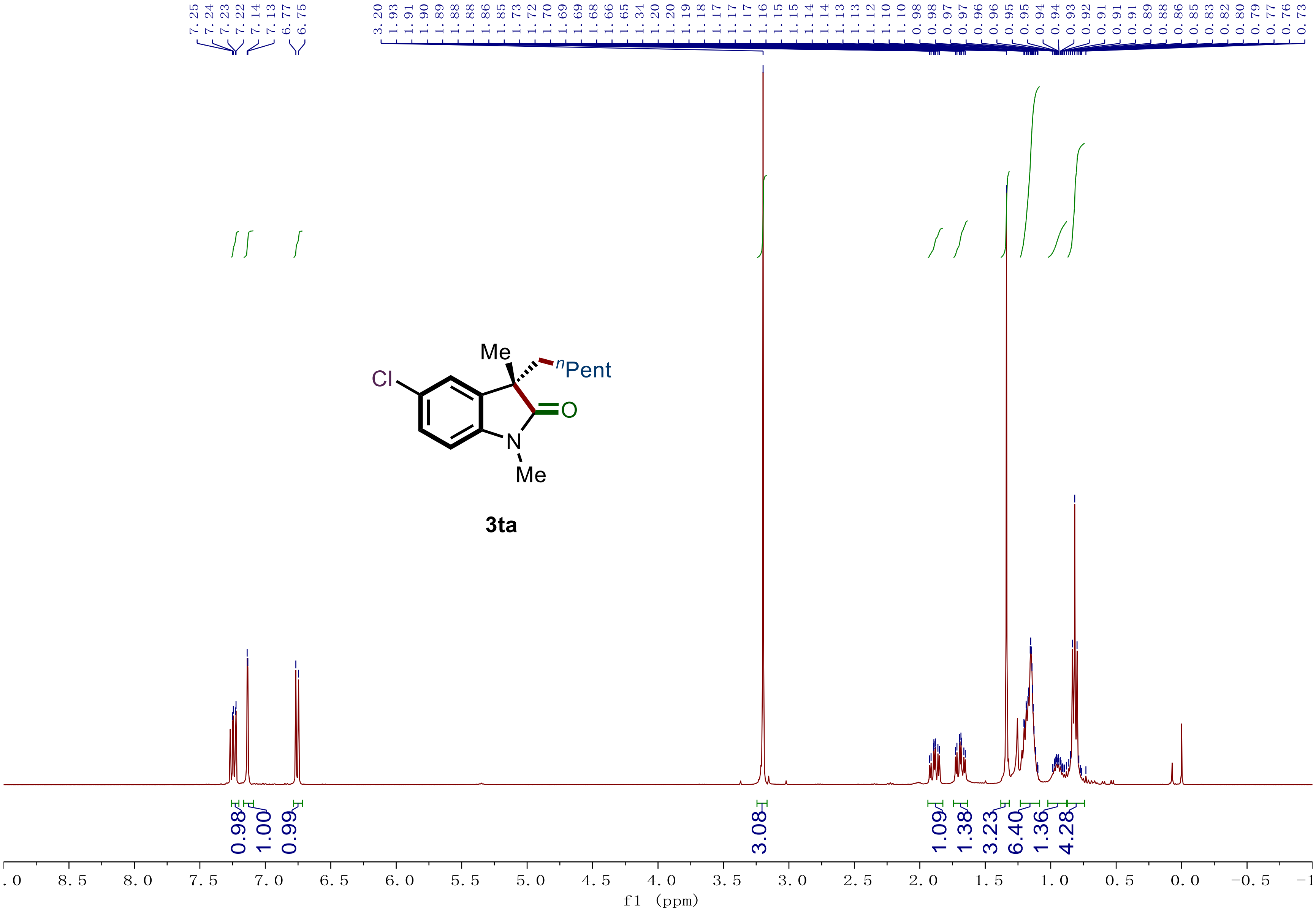

3ta

Supplementary Figure 210

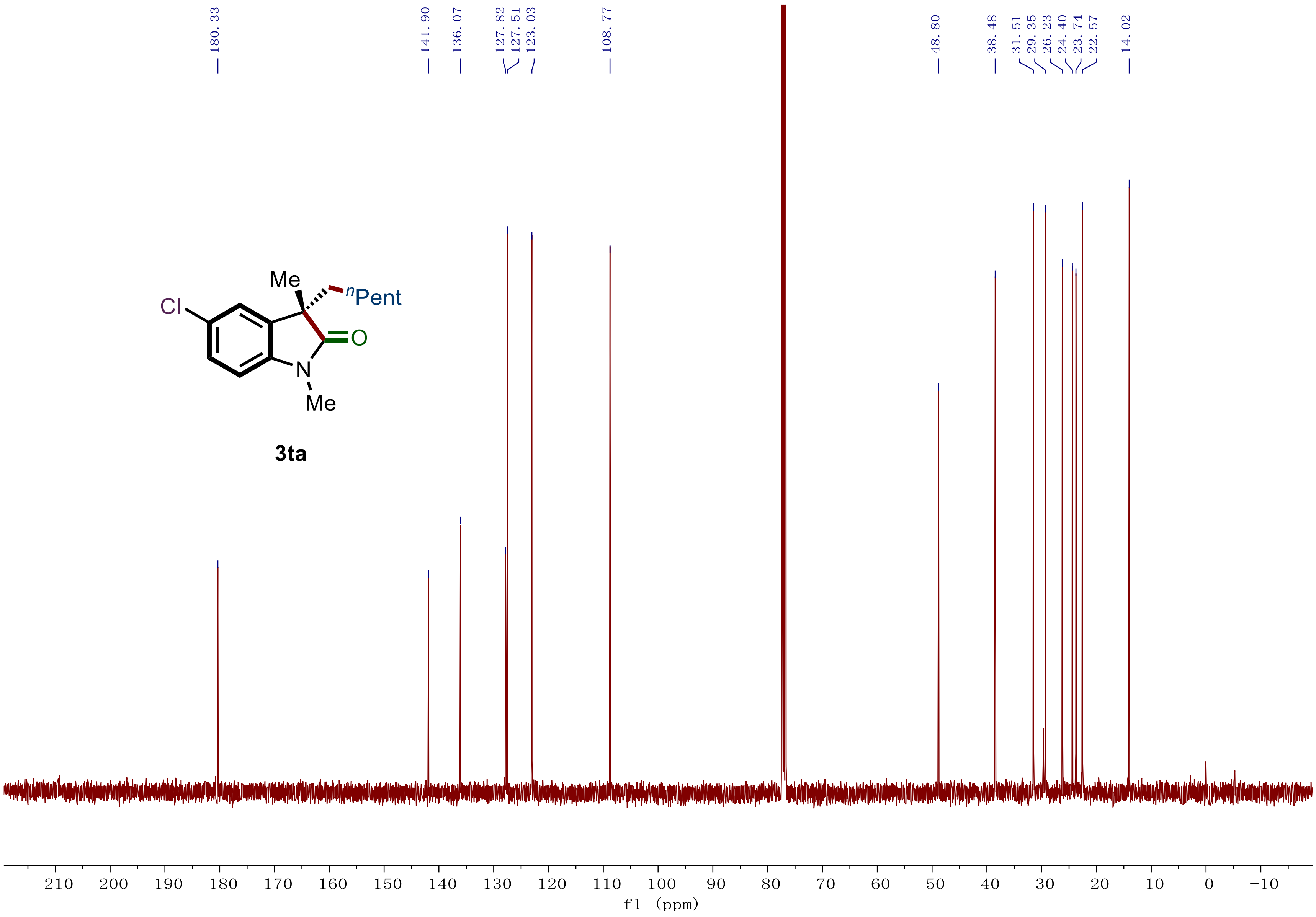

Supplementary Figure 211

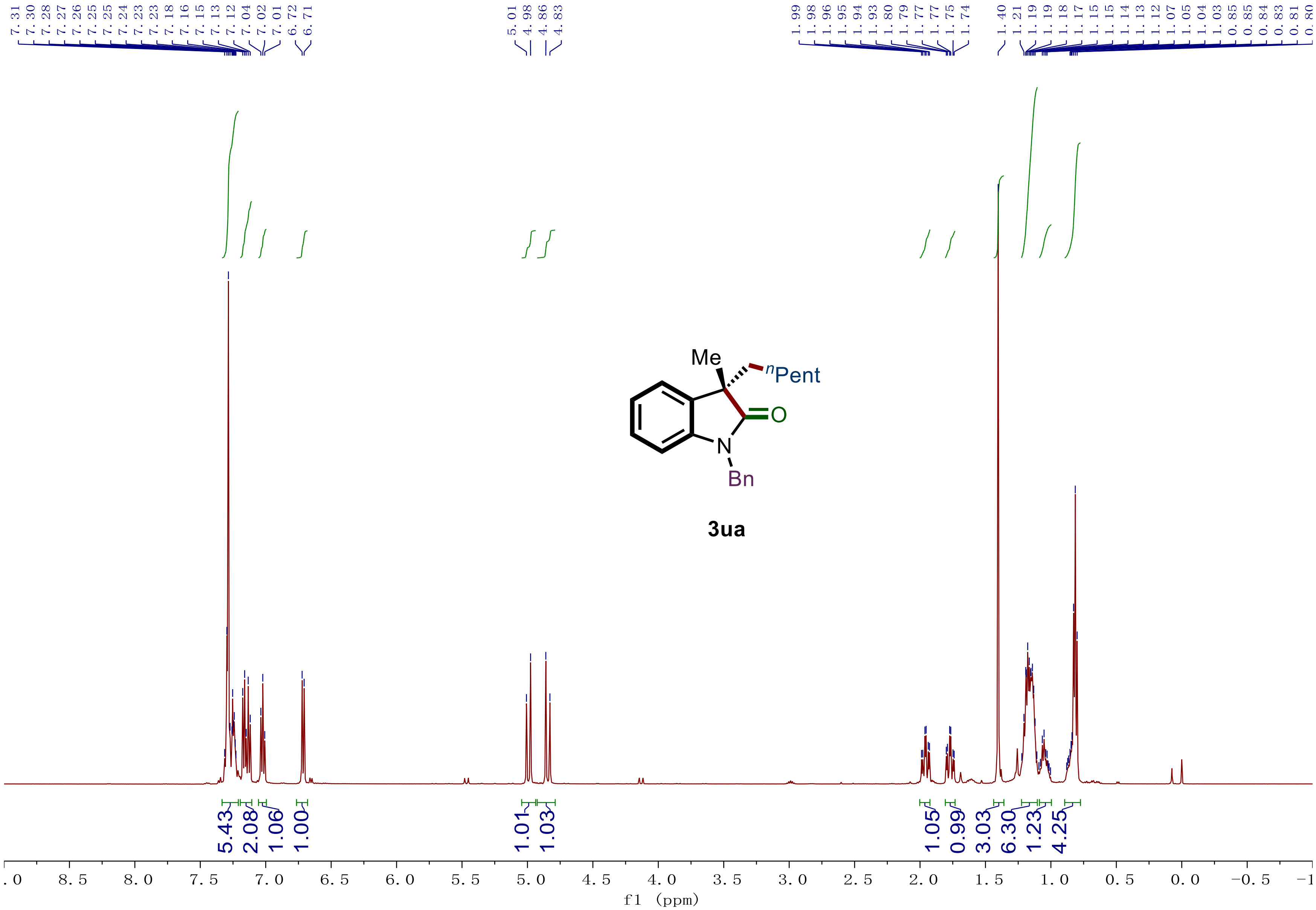

Supplementary Figure 212

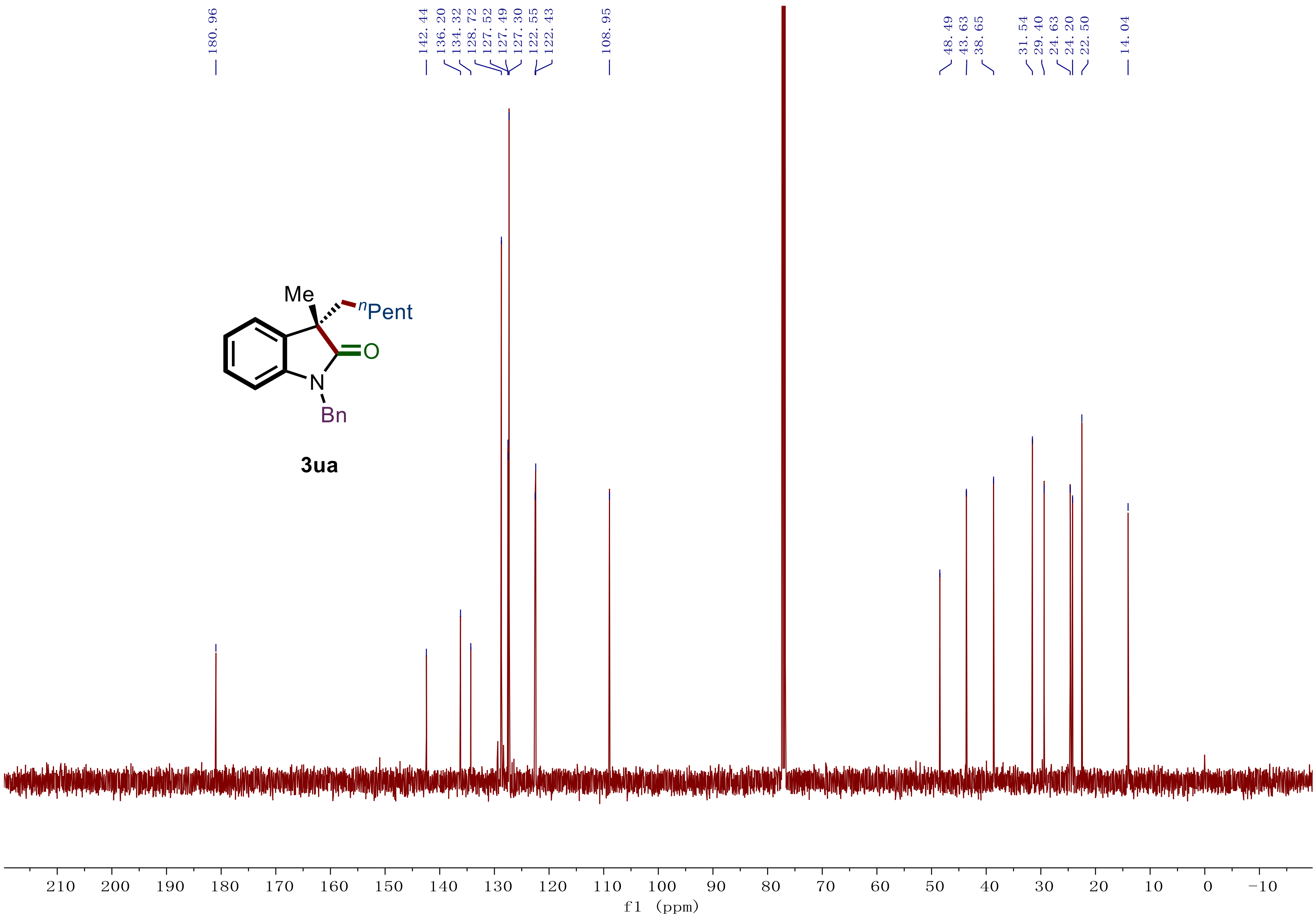

Supplementary Figure 213

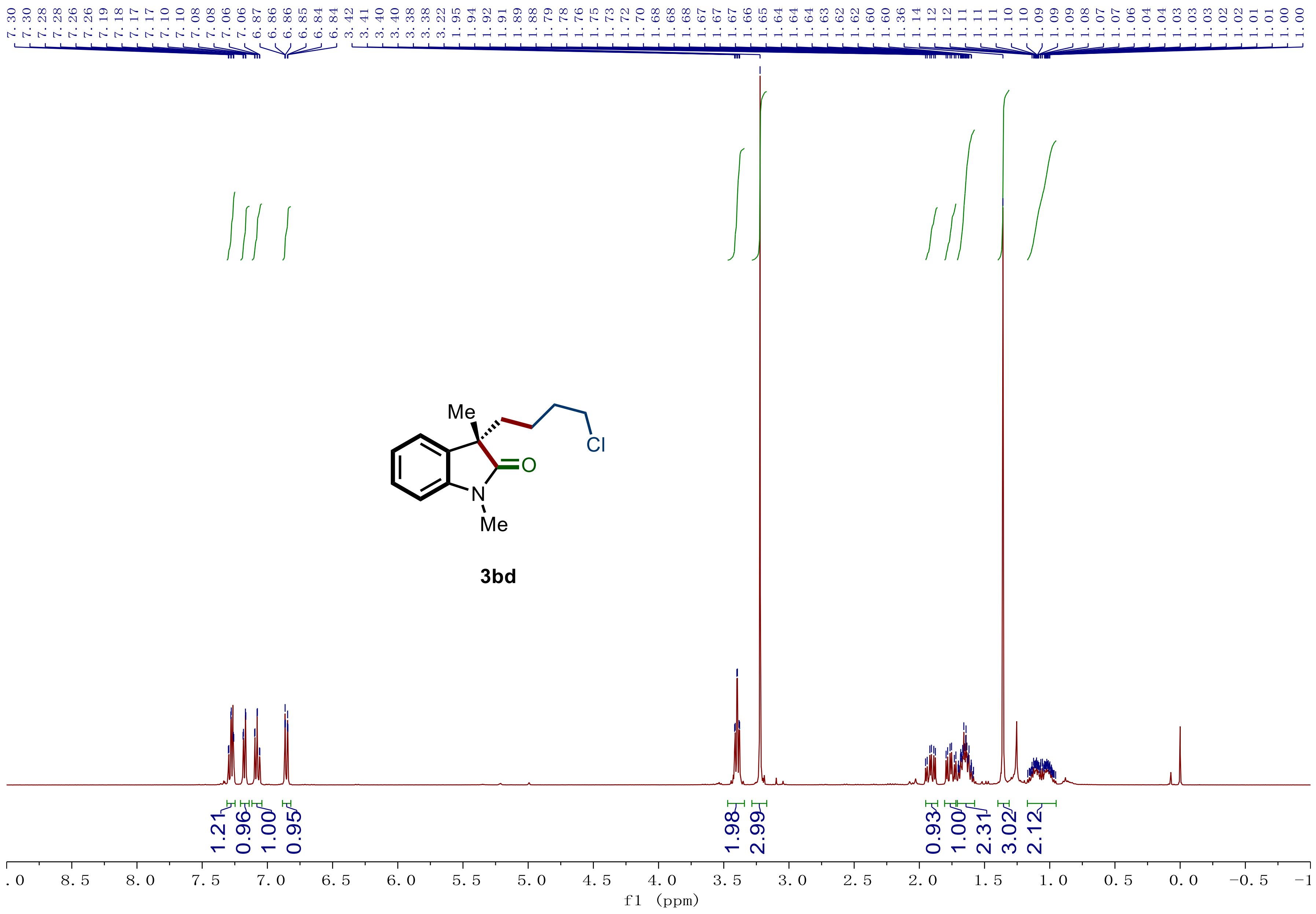

Supplementary Figure 214

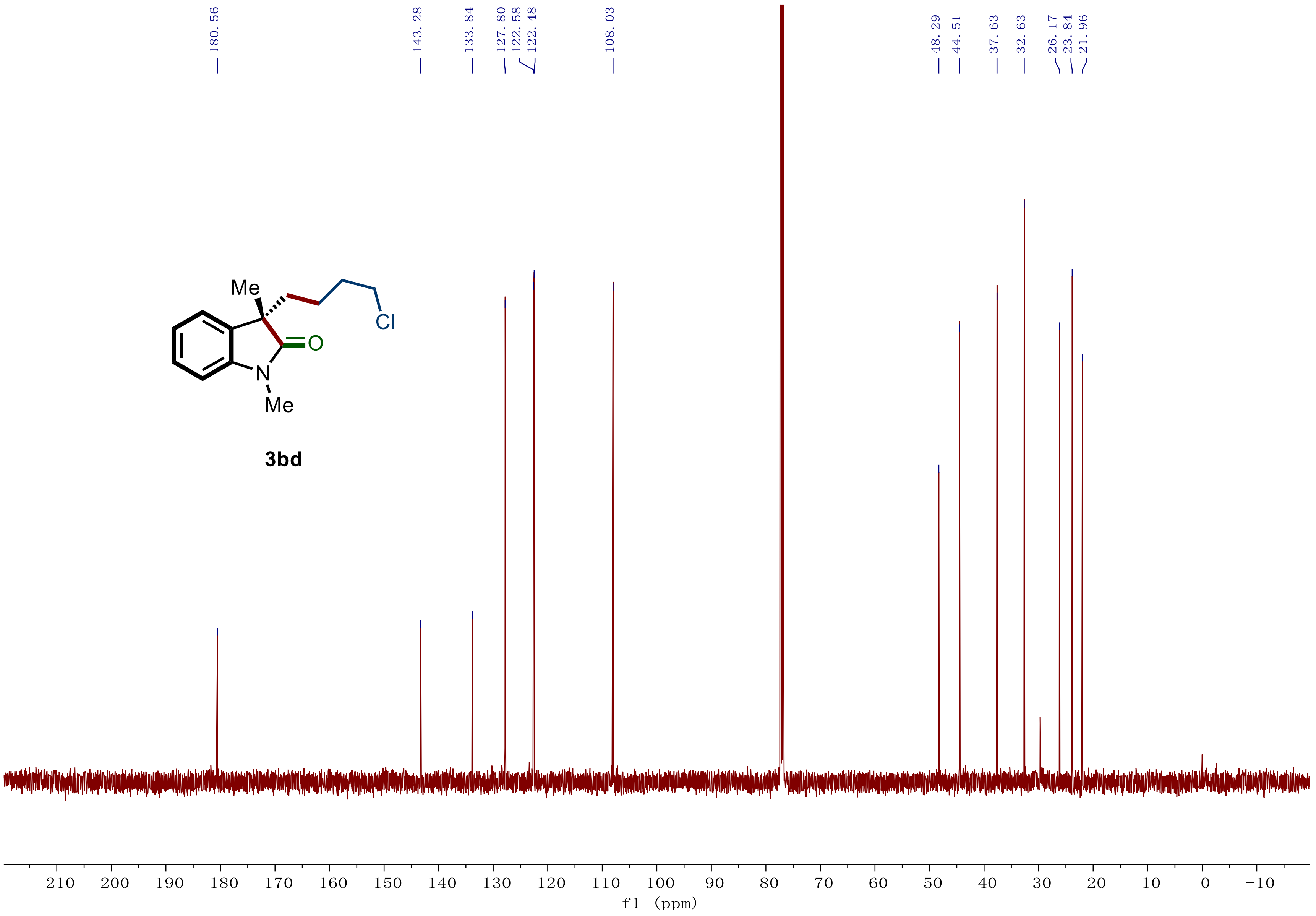

3bd

Supplementary Figure 215

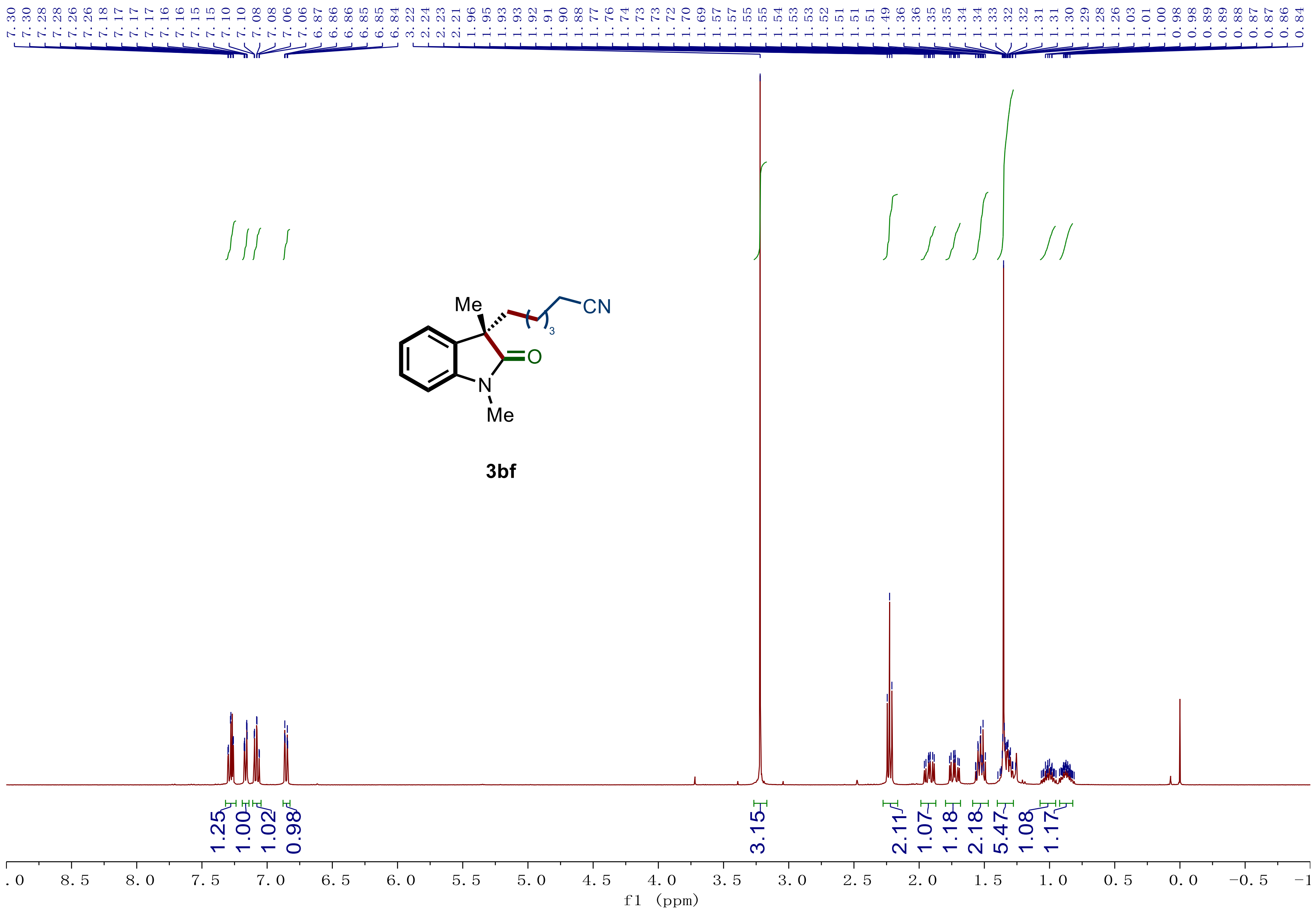

3bf

Supplementary Figure 216

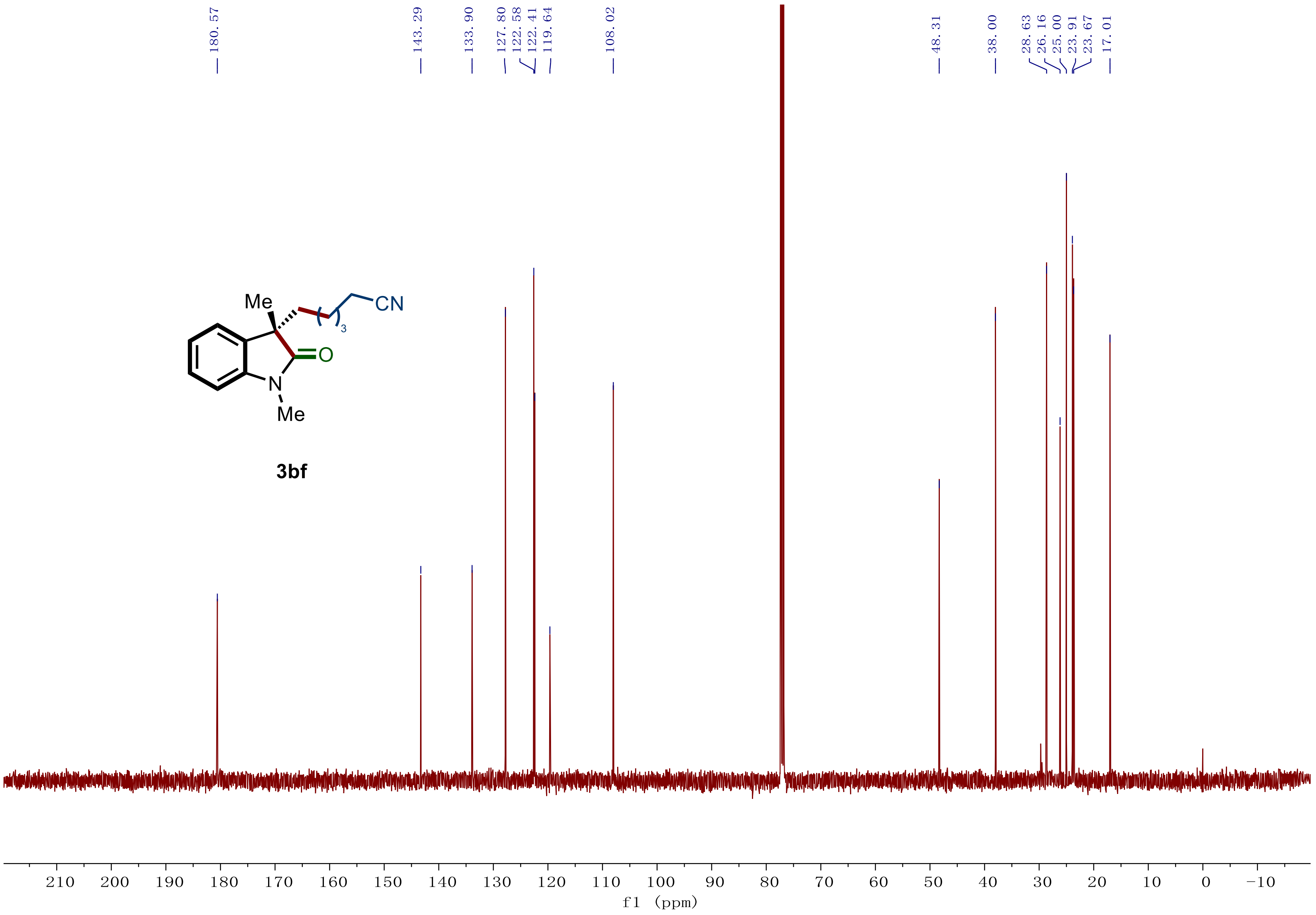

Supplementary Figure 217

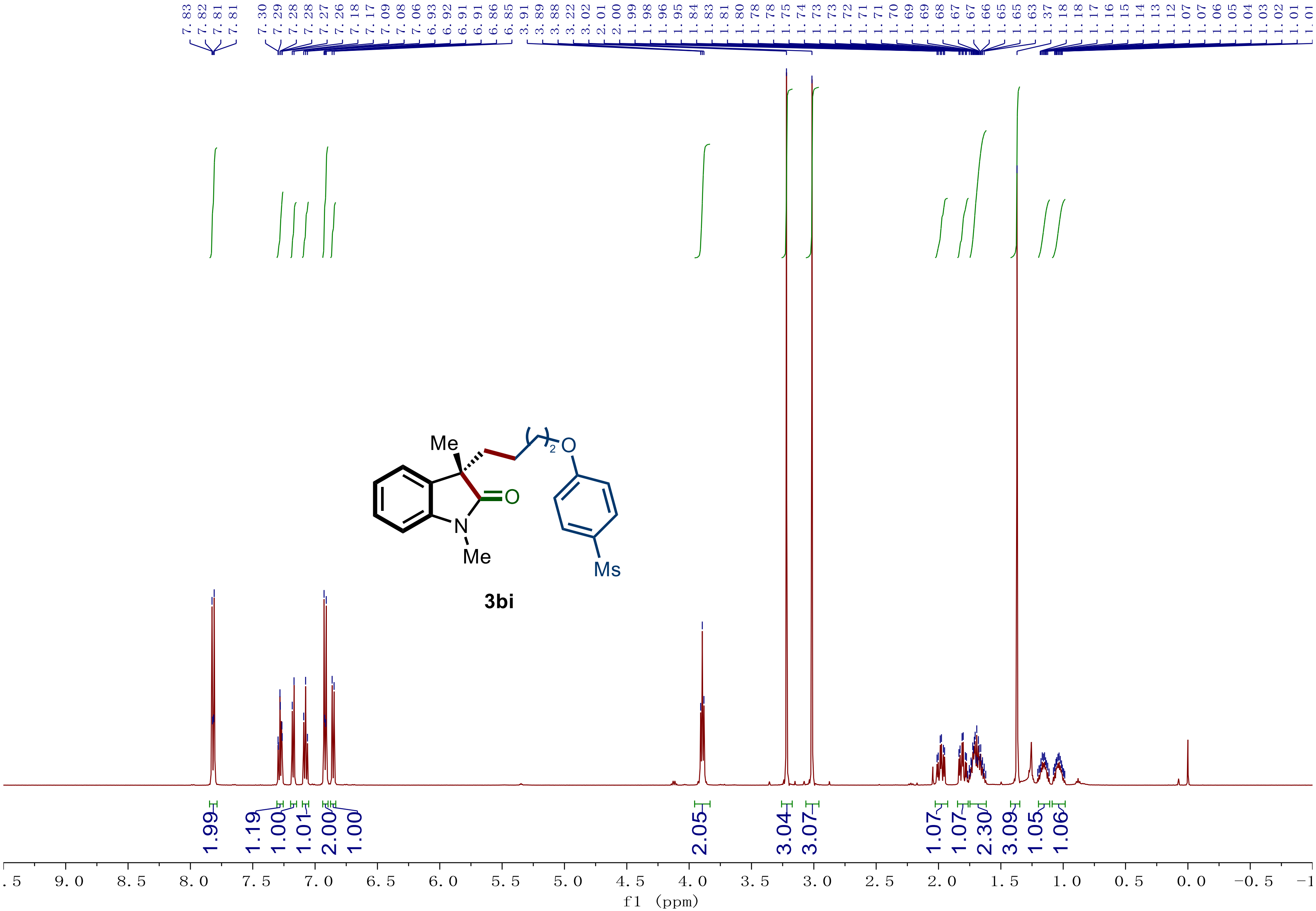

Supplementary Figure 218

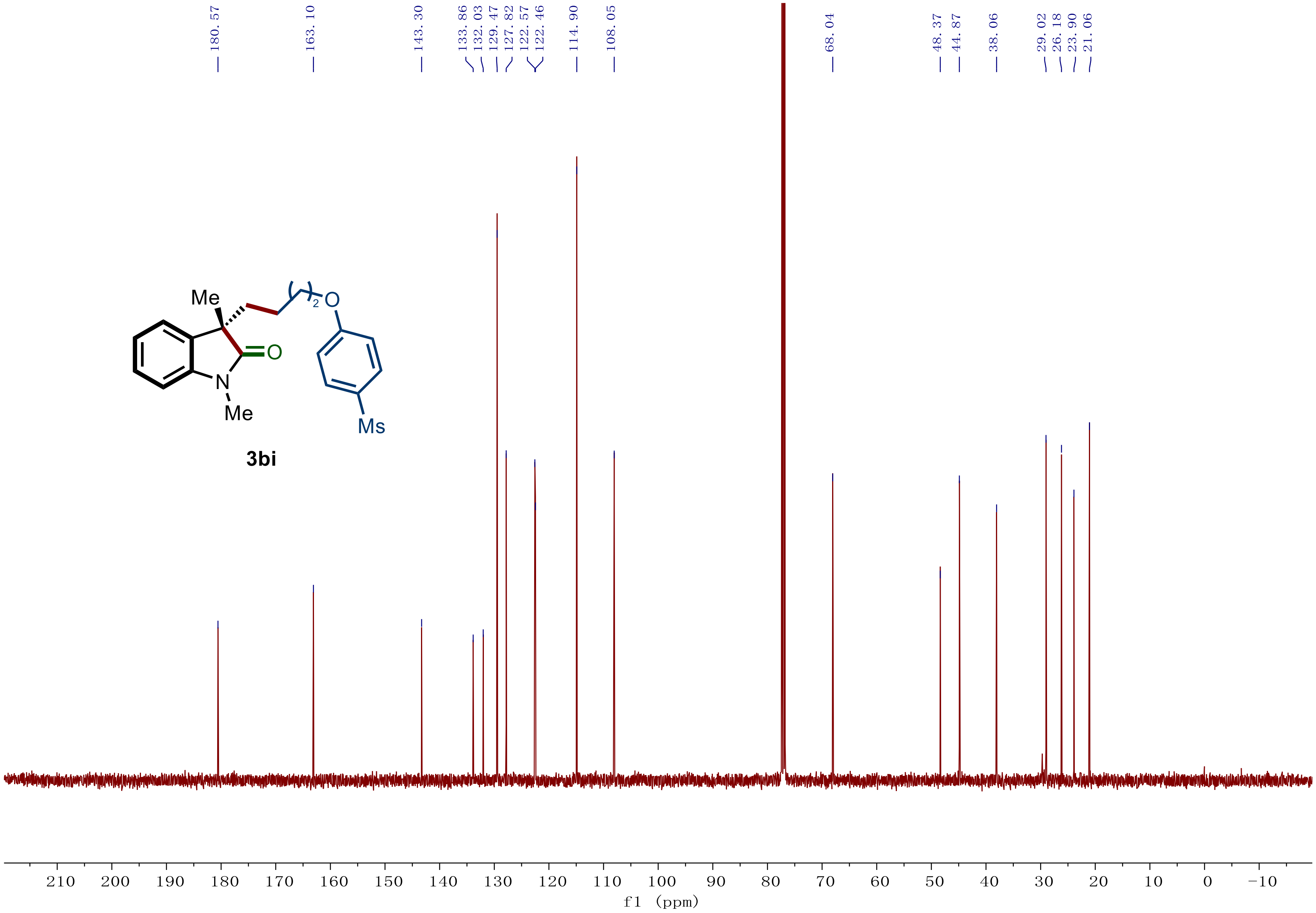

Supplementary Figure 219

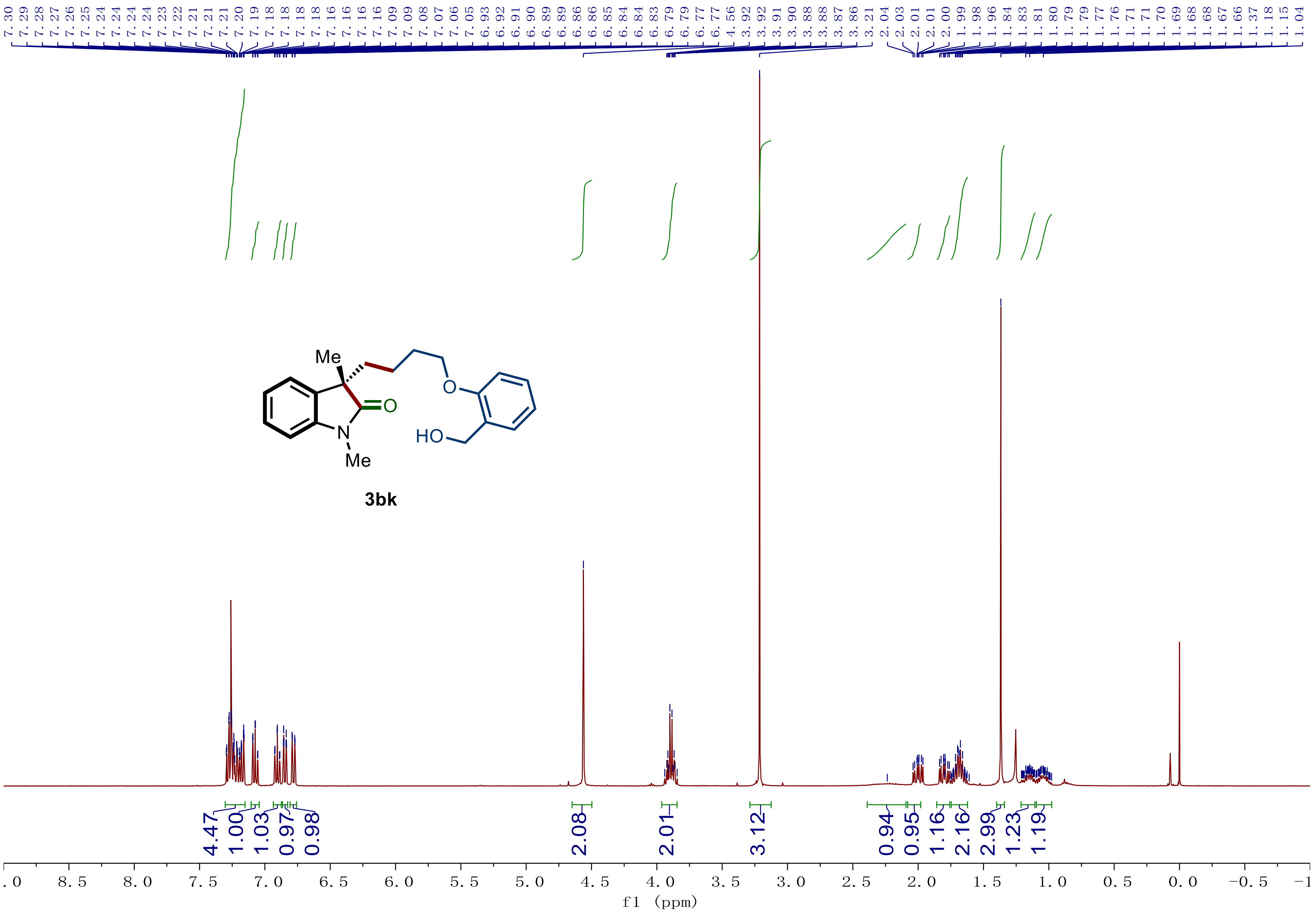

Supplementary Figure 220

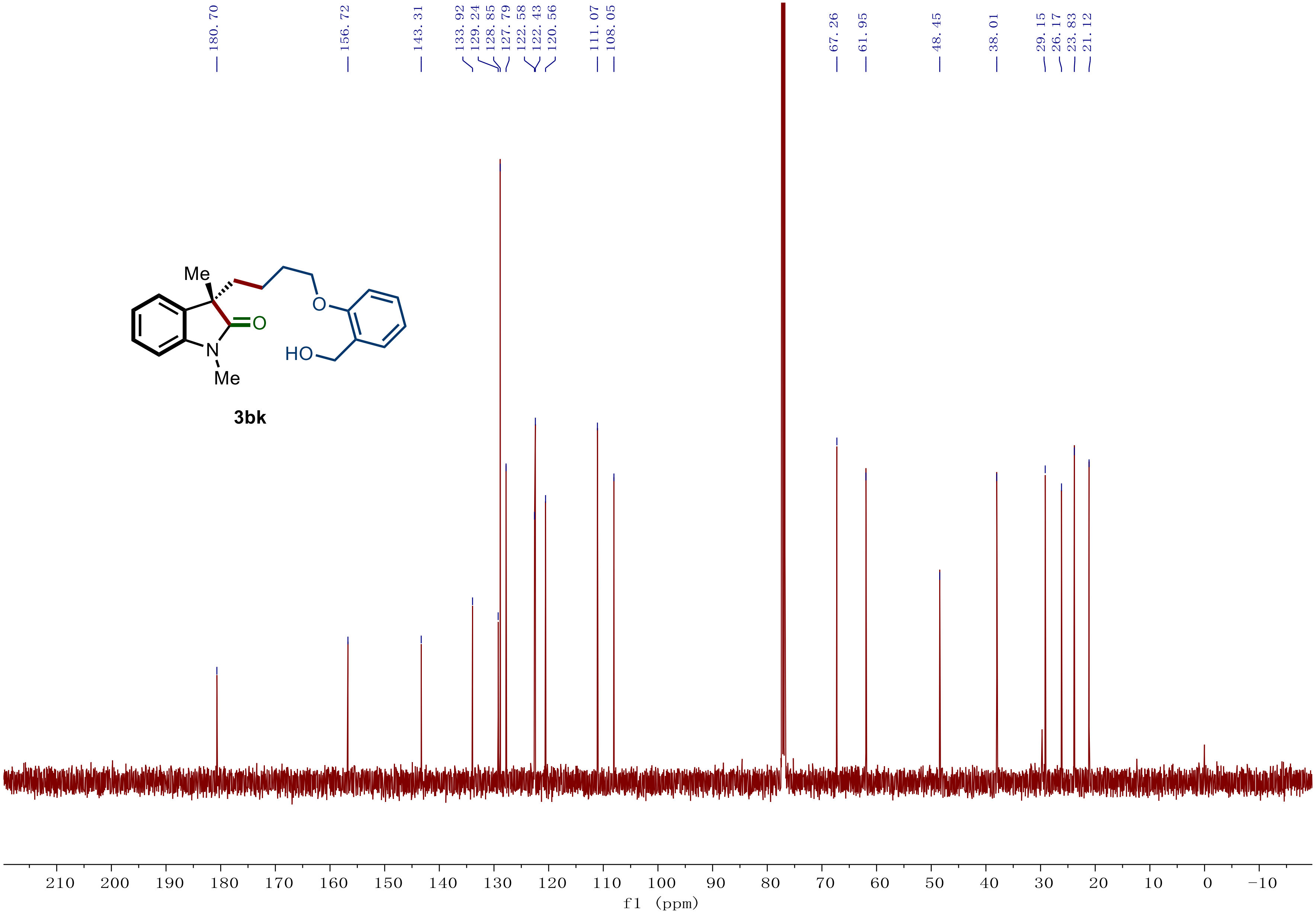

Supplementary Figure 221

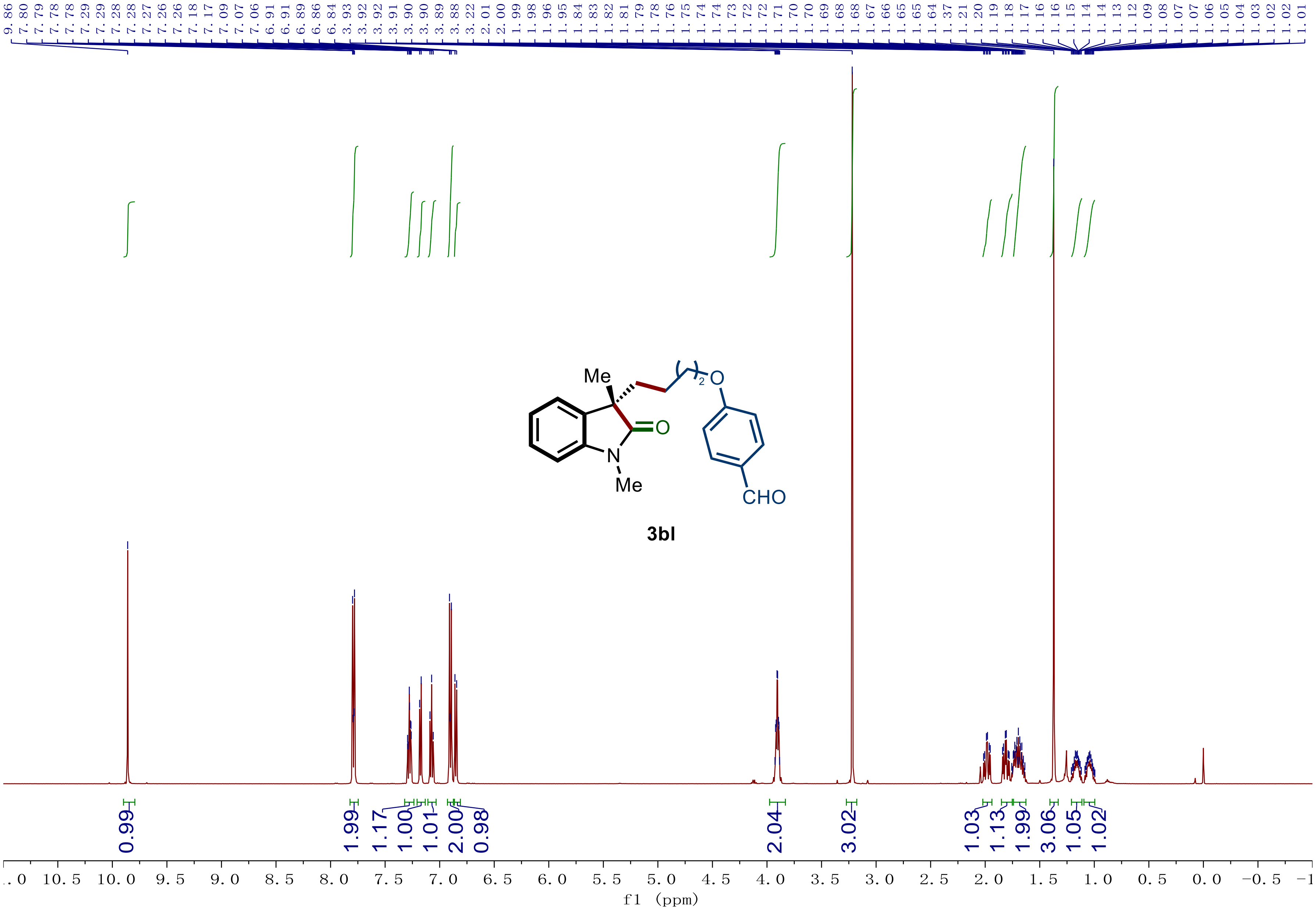

Supplementary Figure 222

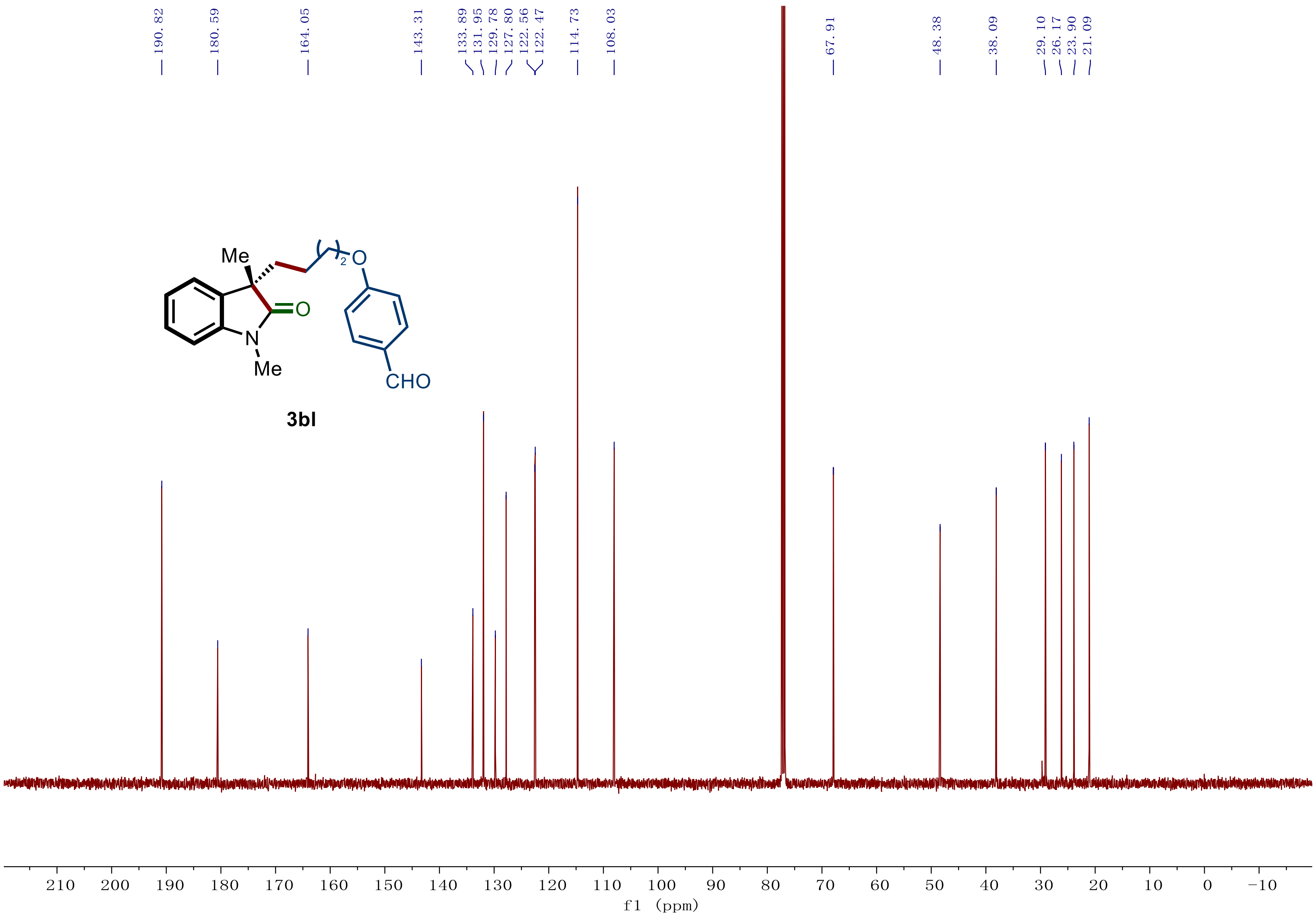

Supplementary Figure 223

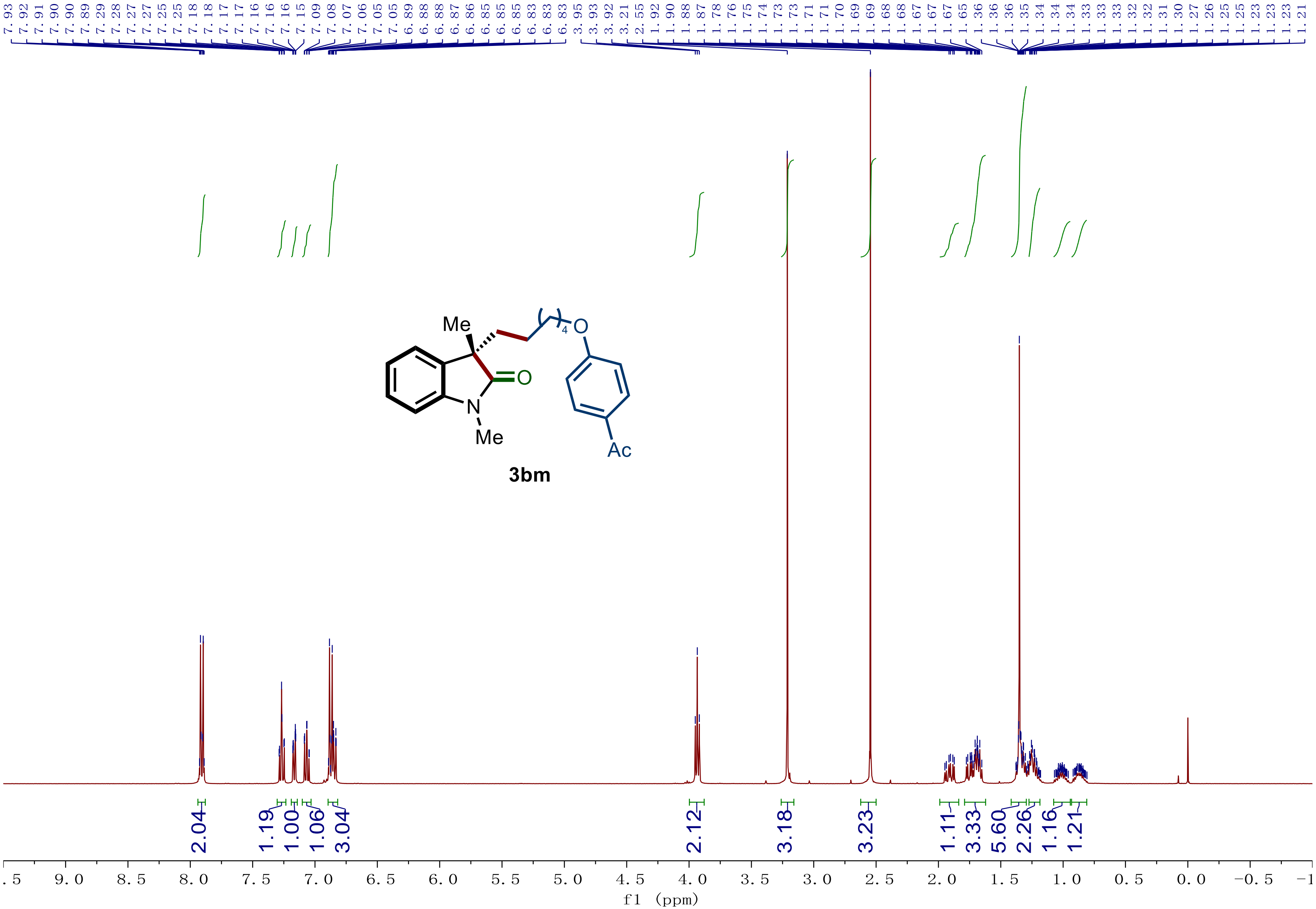

Supplementary Figure 224

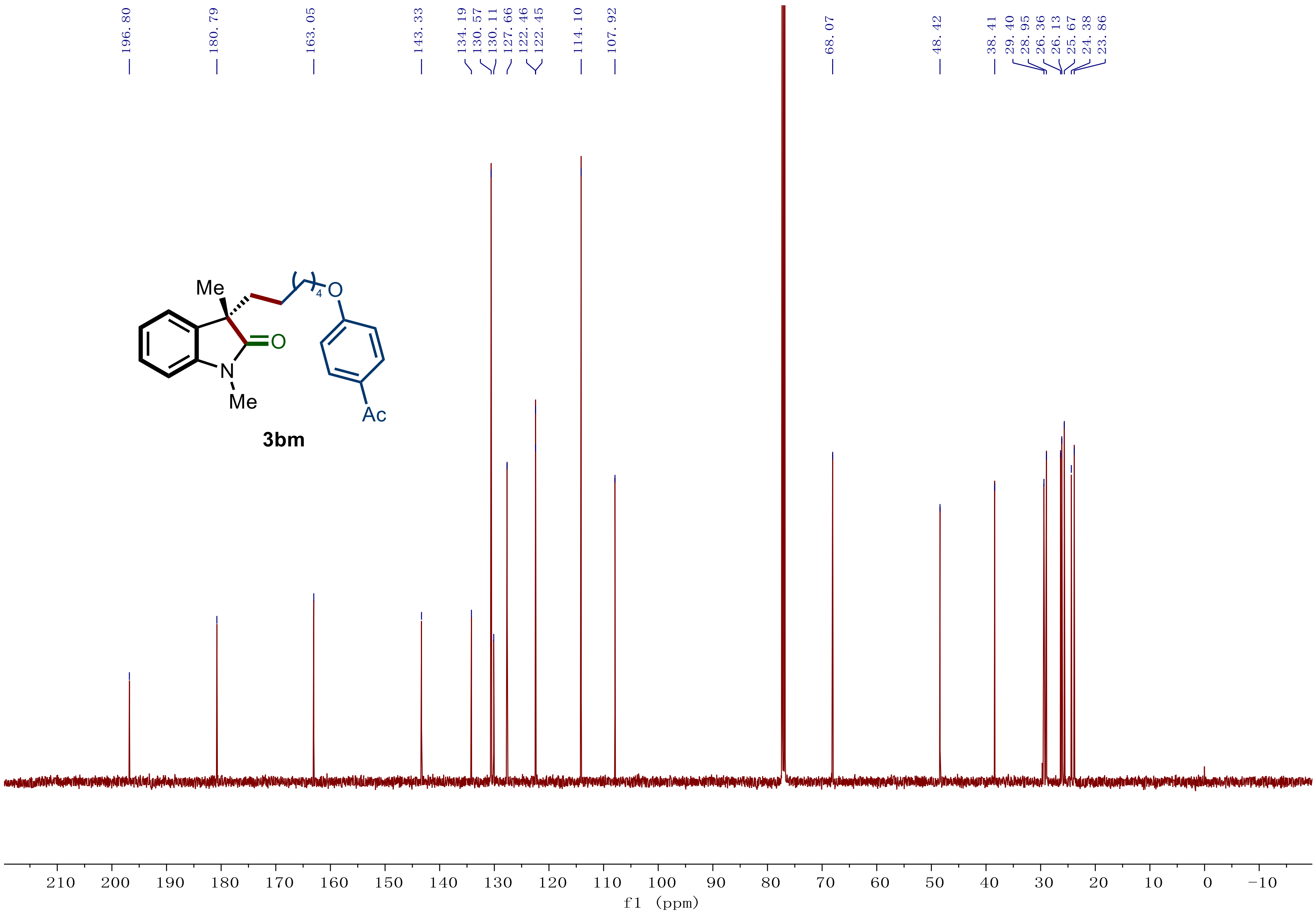

Supplementary Figure 225

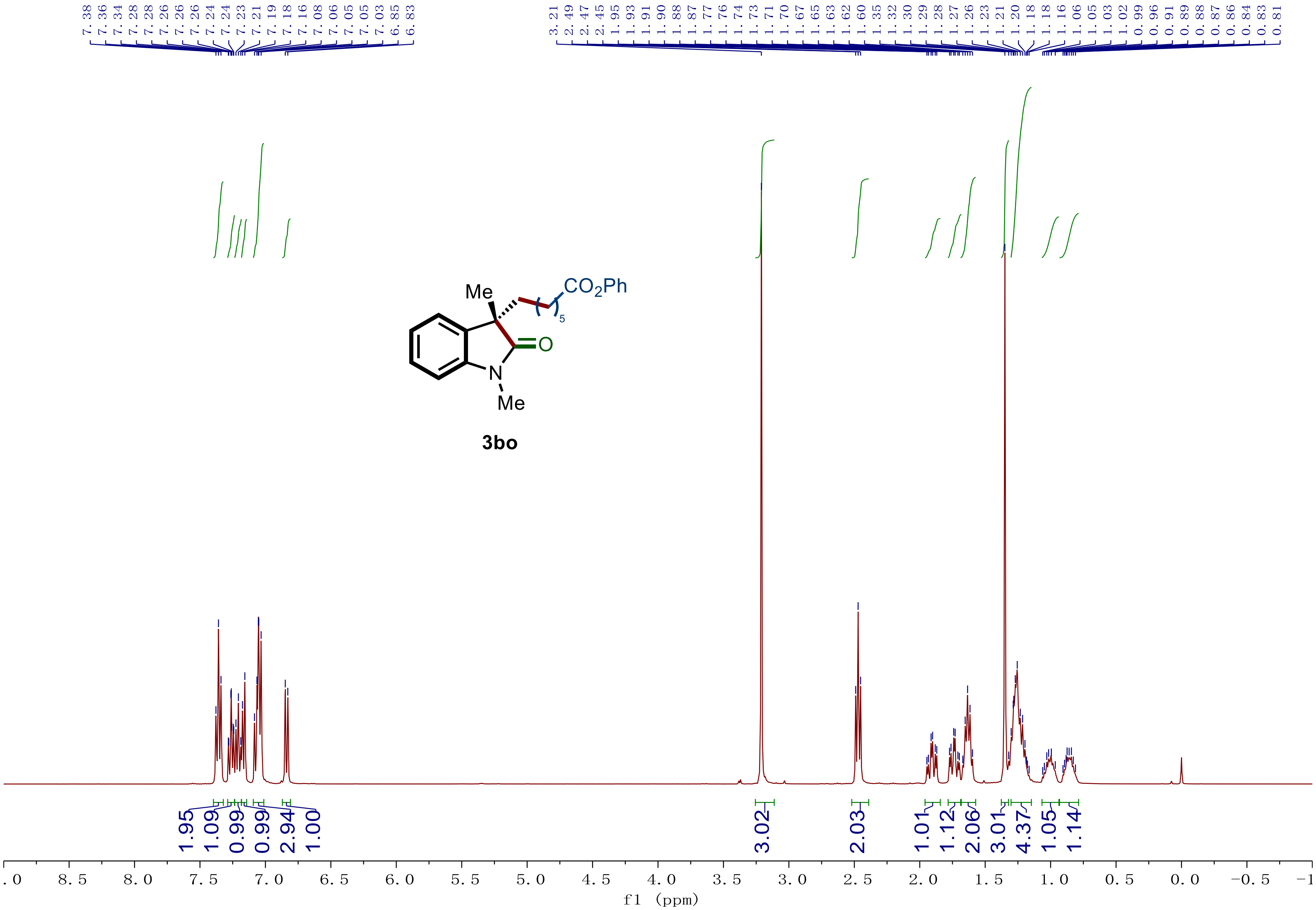

Supplementary Figure 226

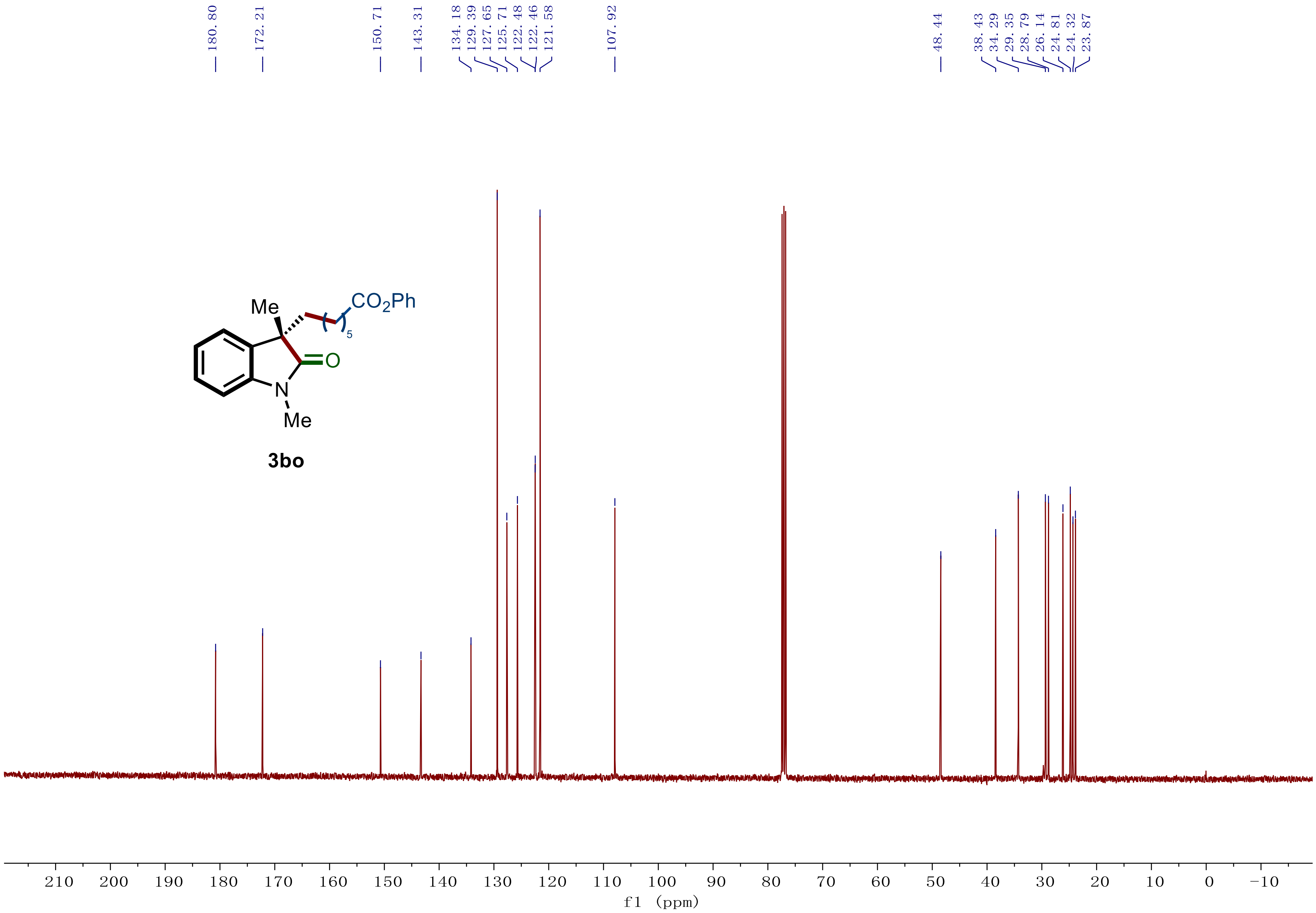

Supplementary Figure 227

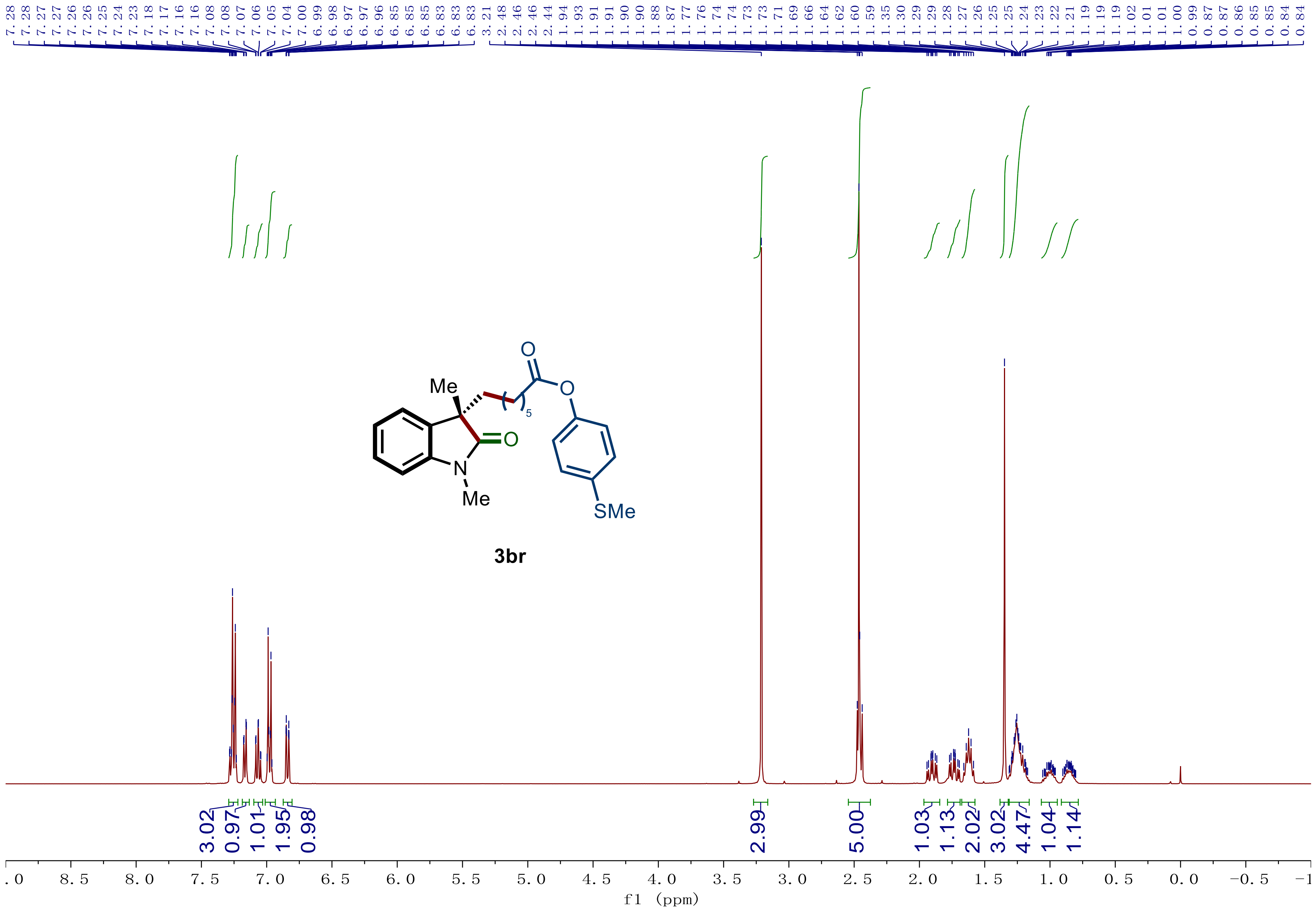

Supplementary Figure 228

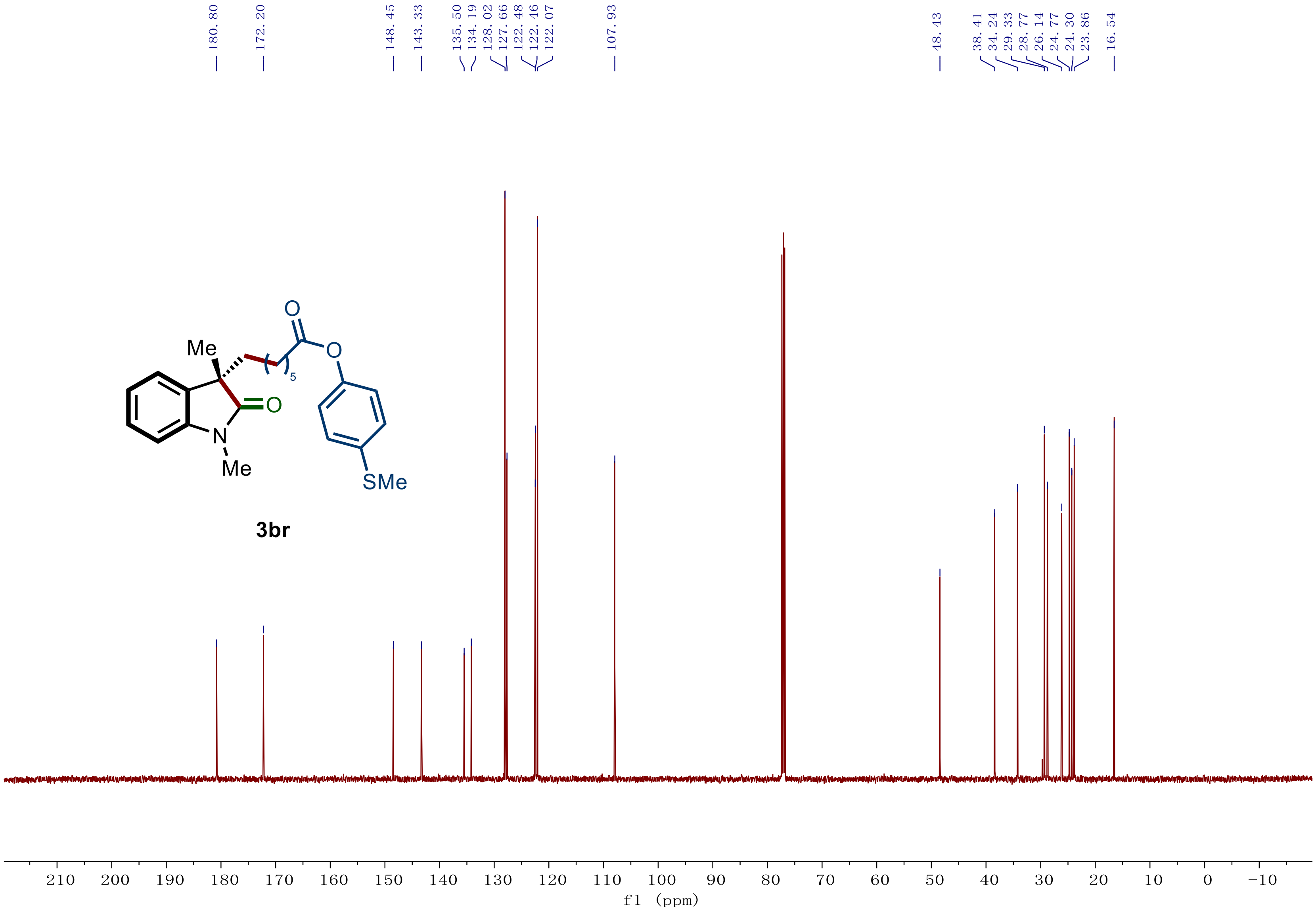

Supplementary Figure 229

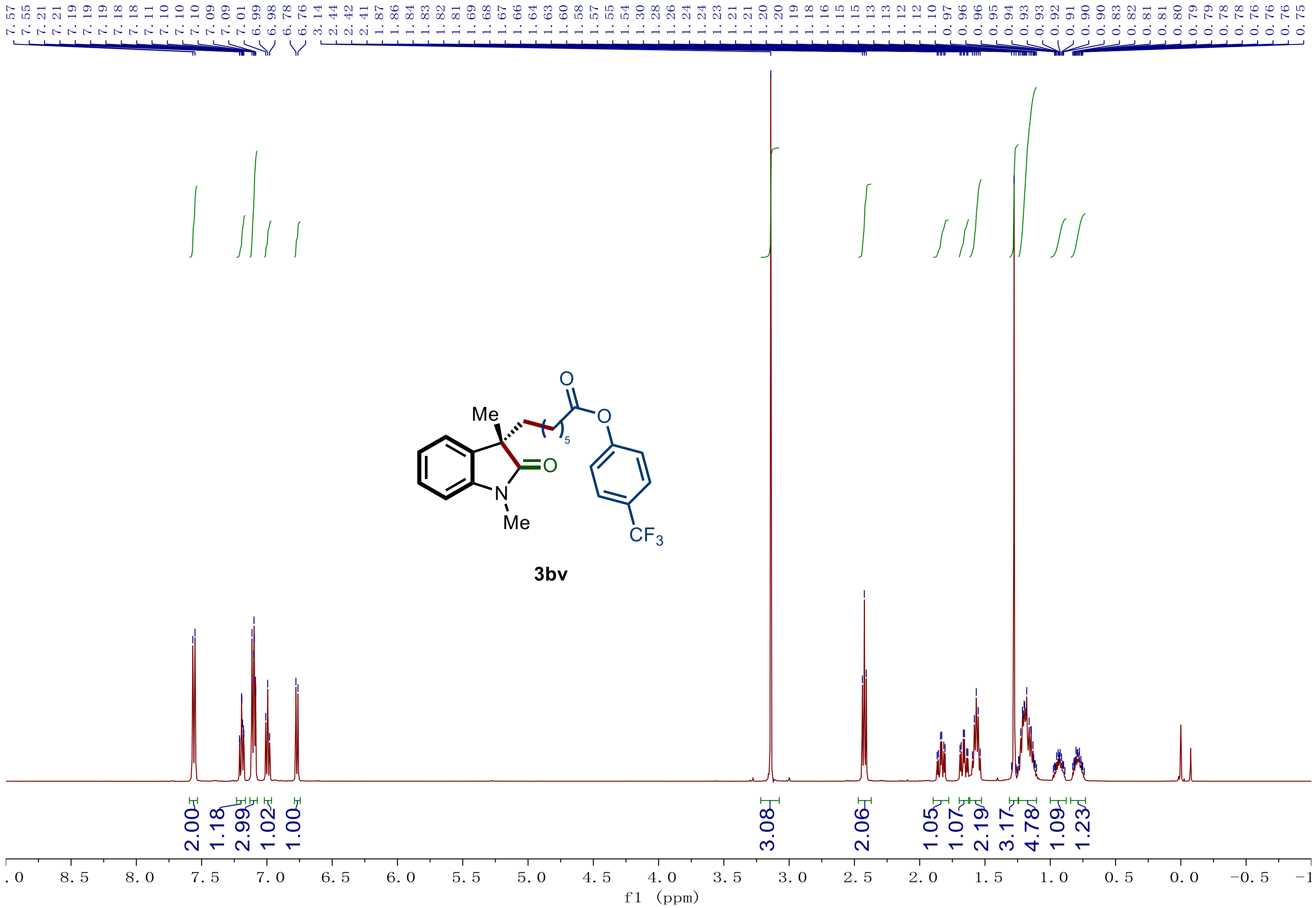

Supplementary Figure 230

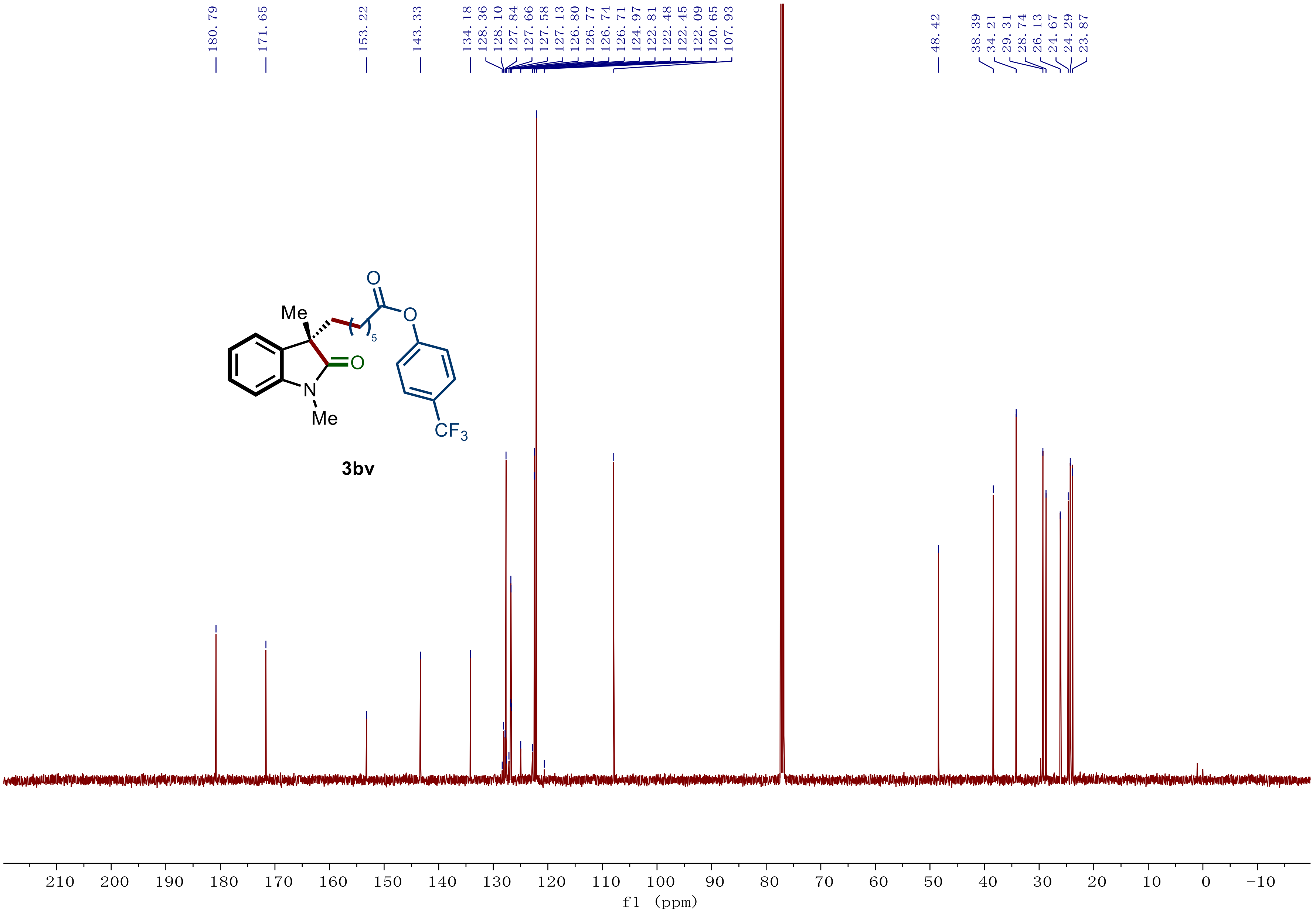

Supplementary Figure 231

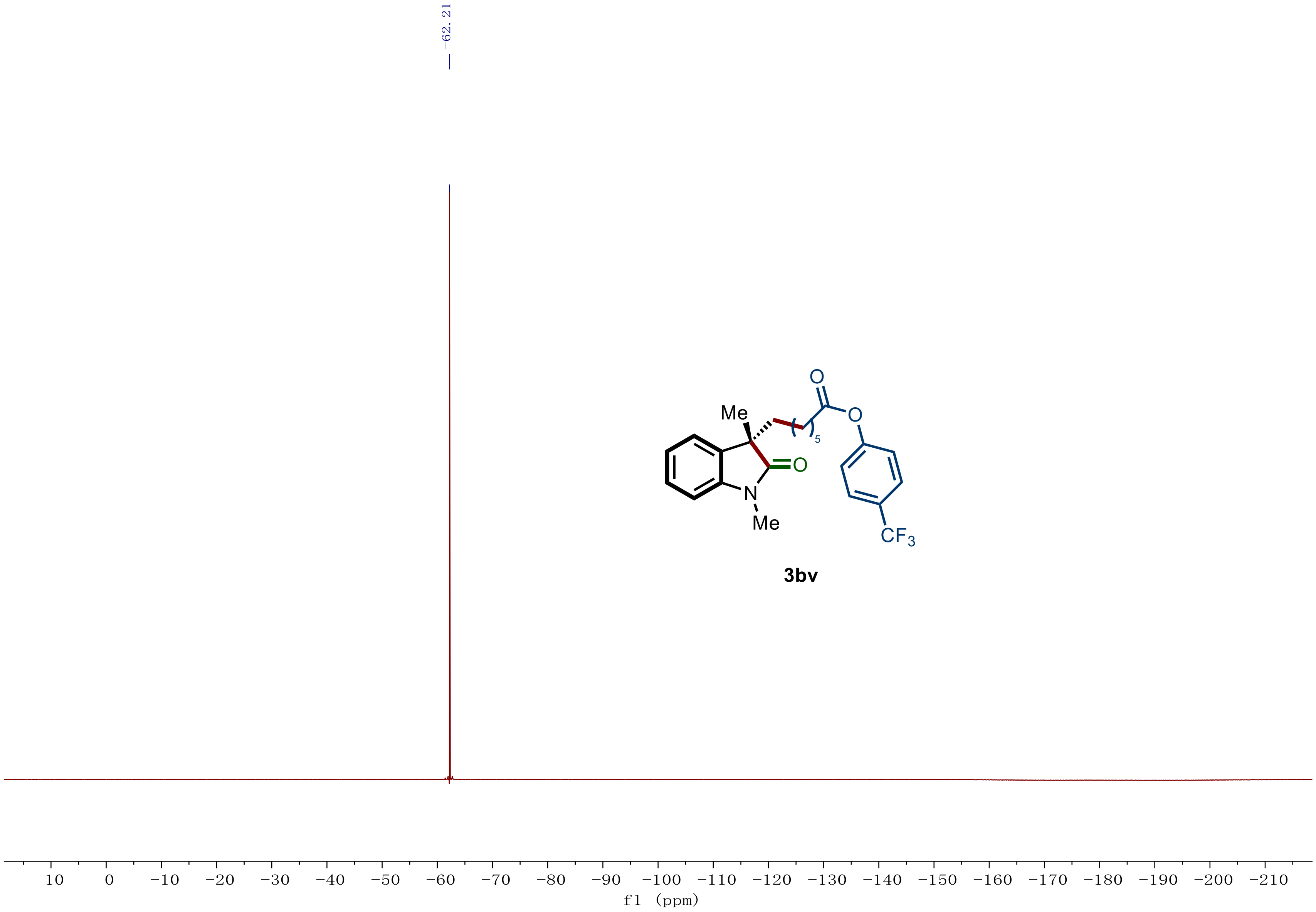

3bv

Supplementary Figure 232

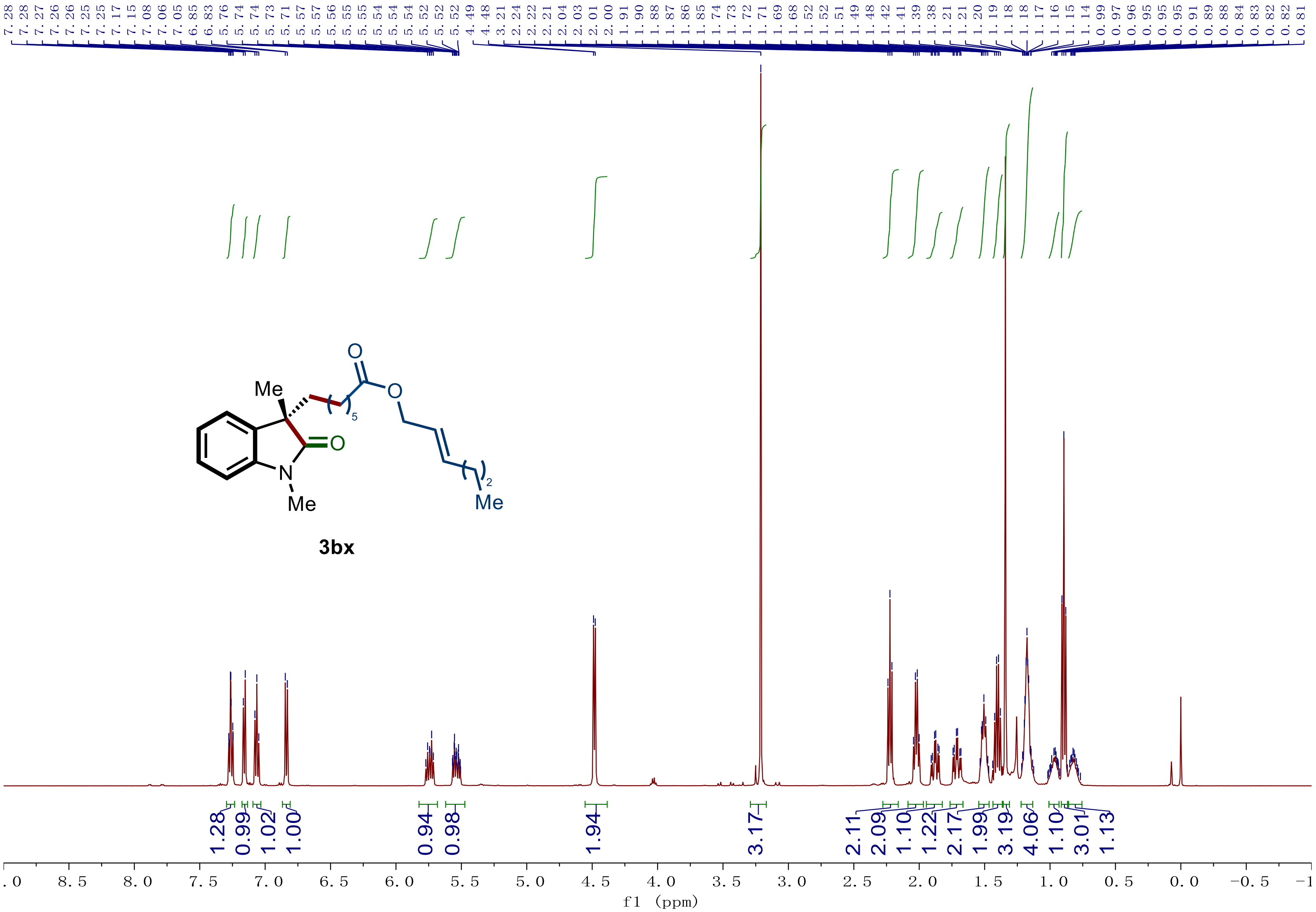

Supplementary Figure 233

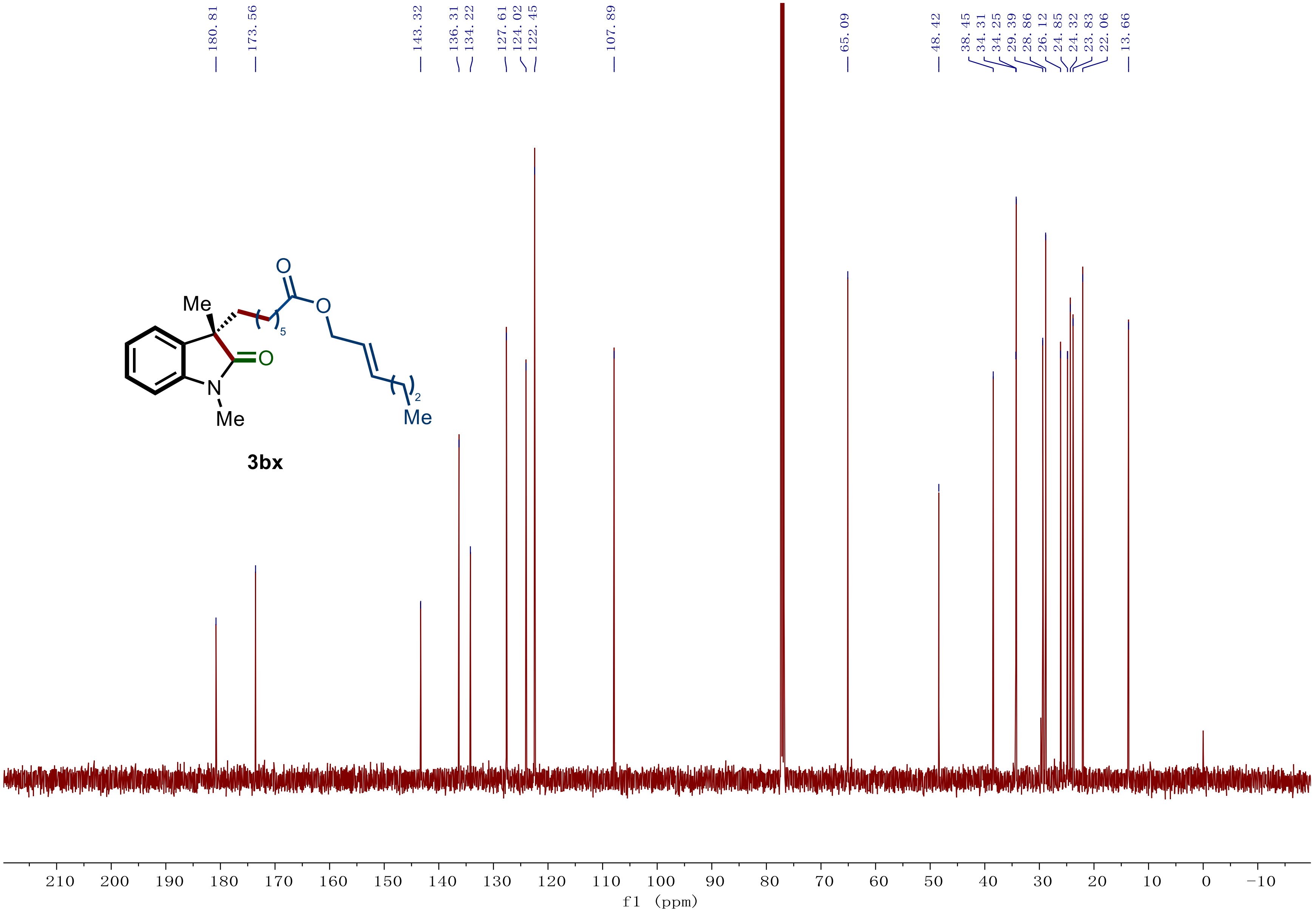

Supplementary Figure 234

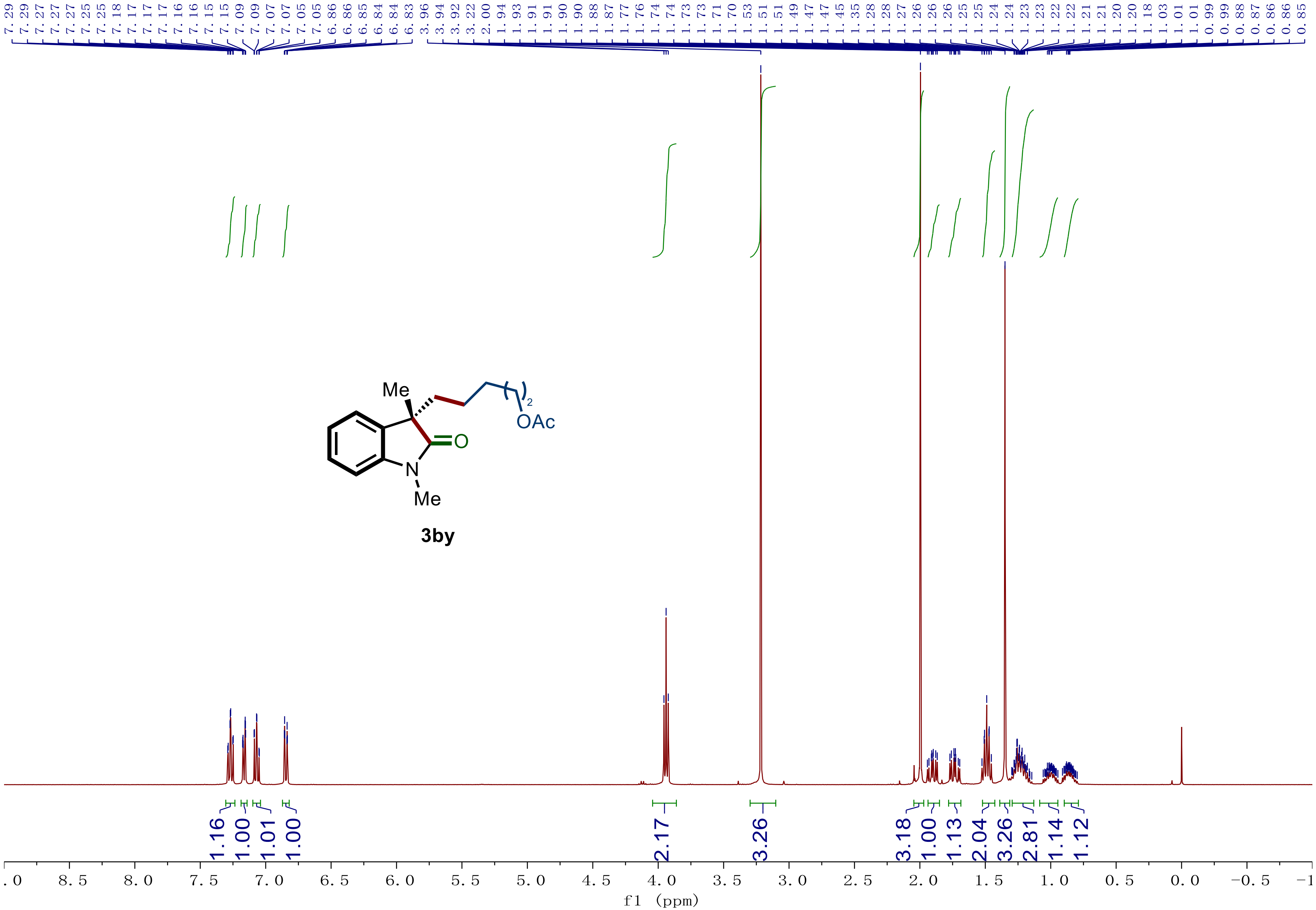

Supplementary Figure 235

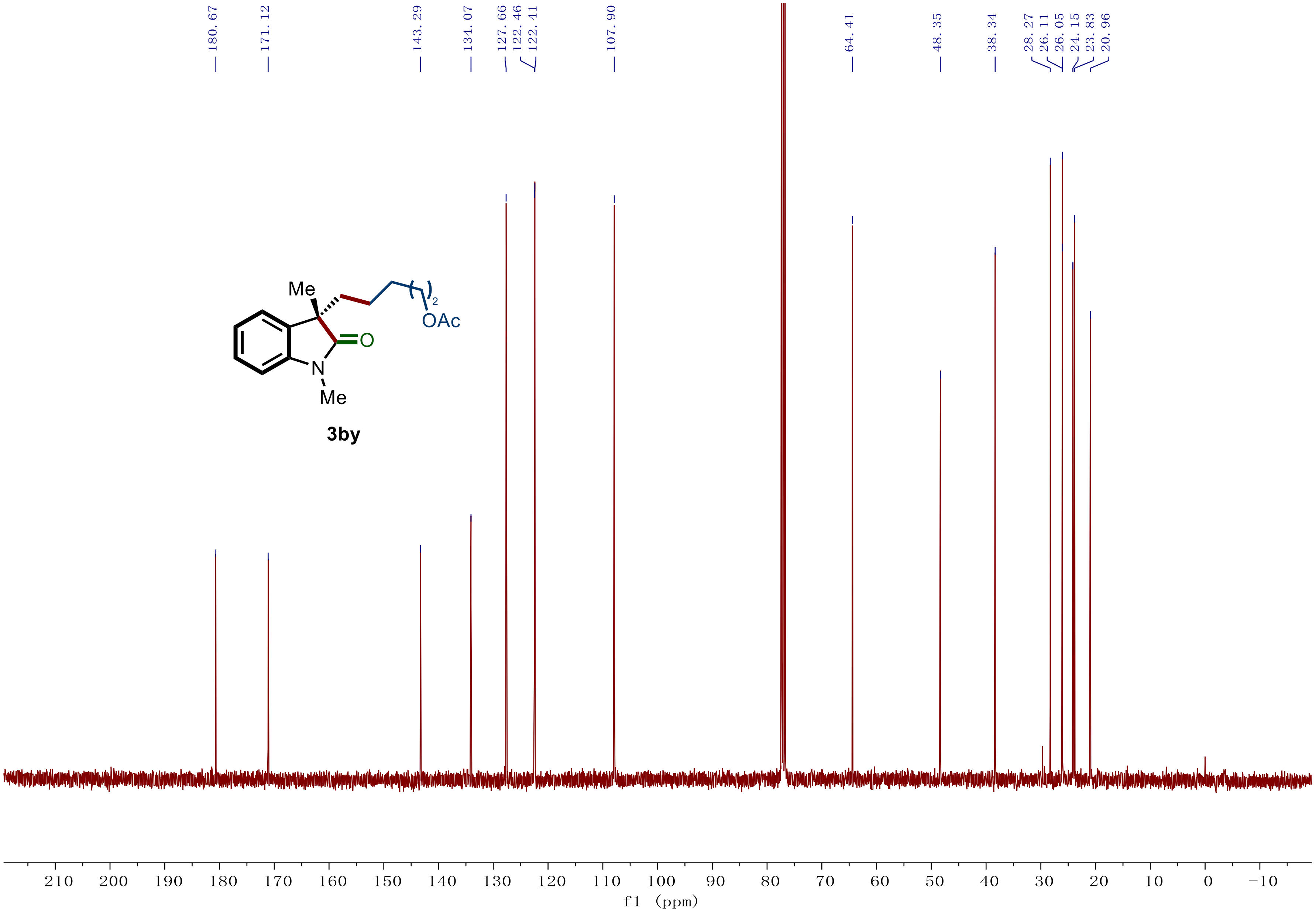

Supplementary Figure 236

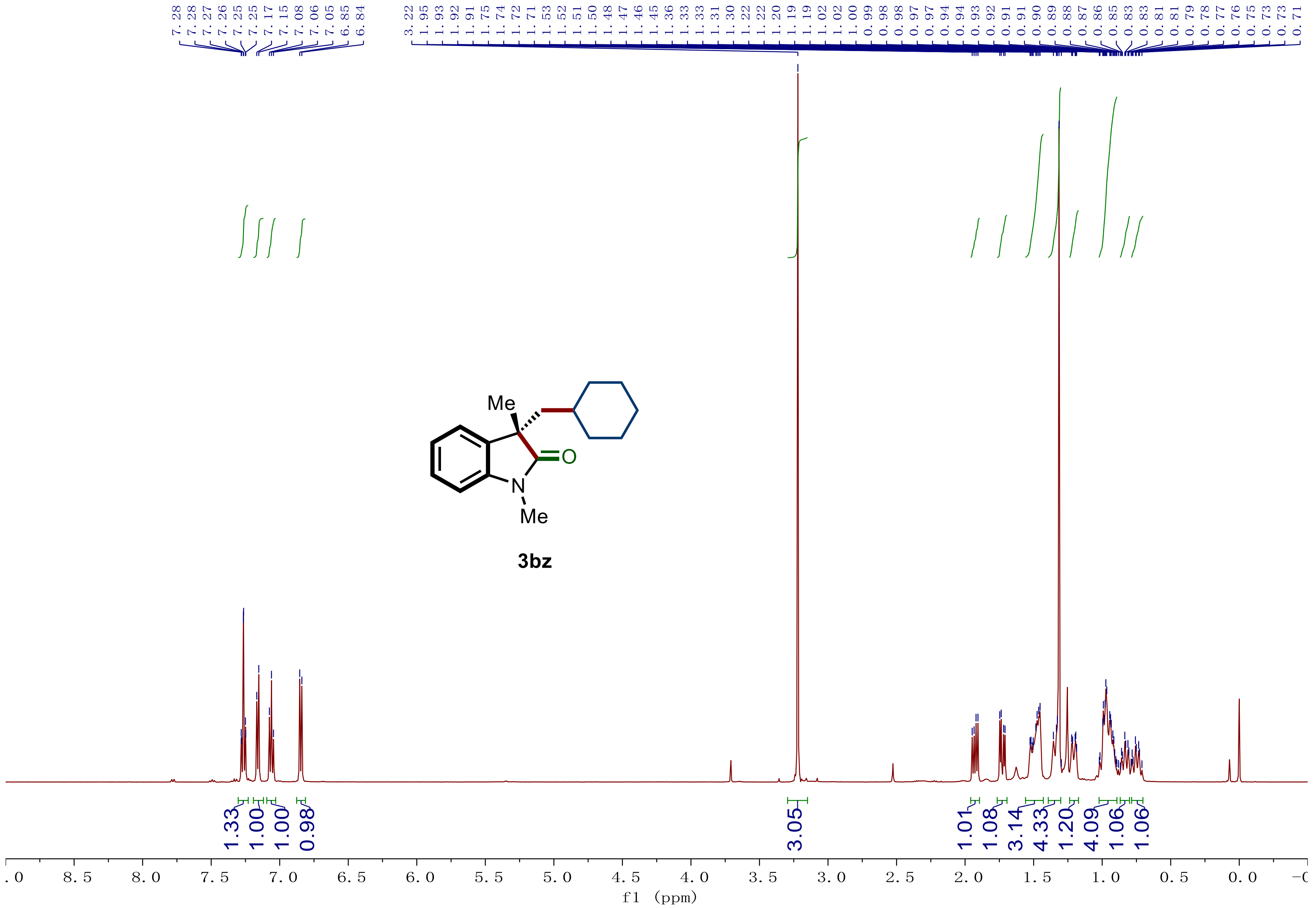

Supplementary Figure 237

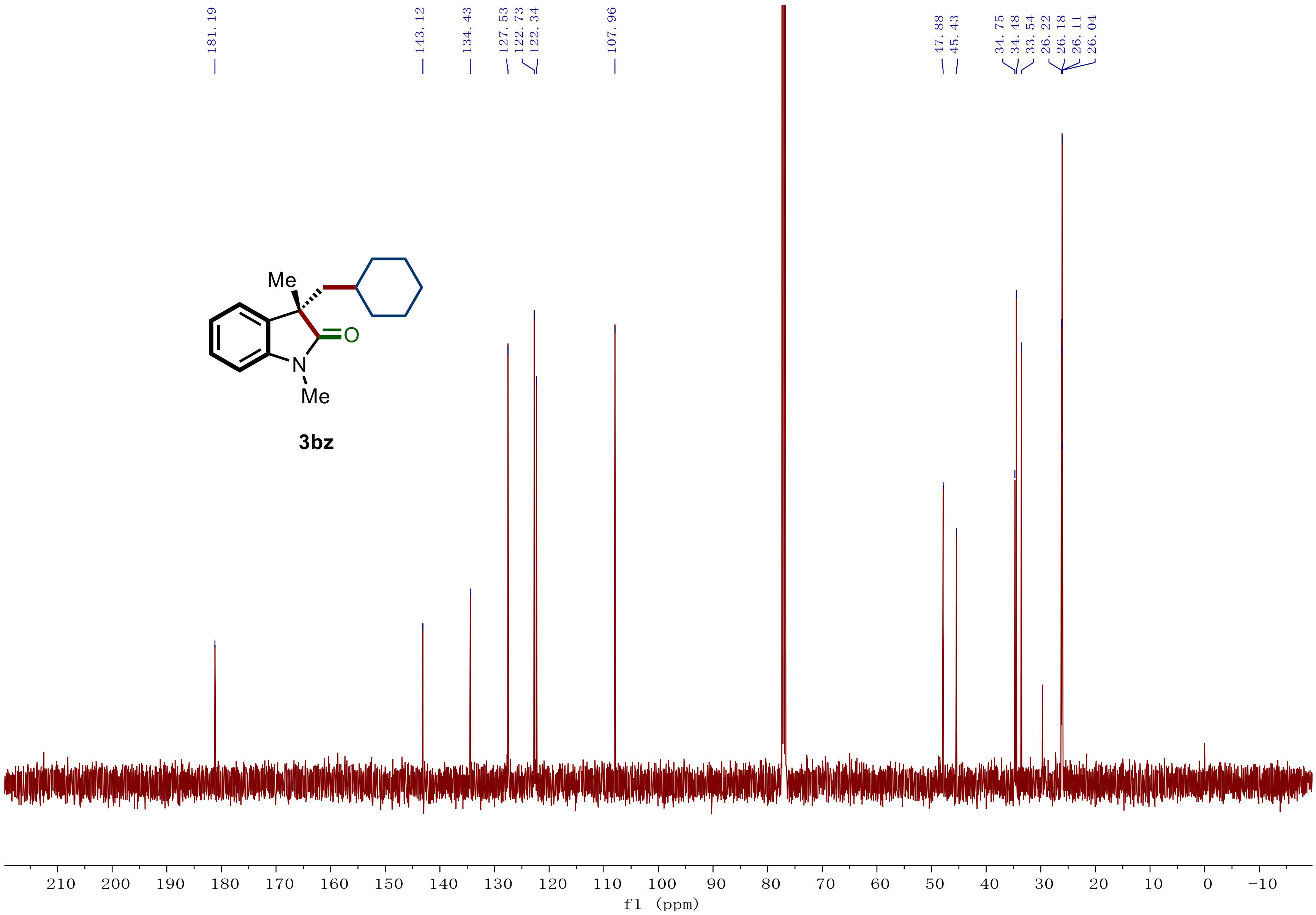

Supplementary Figure 238

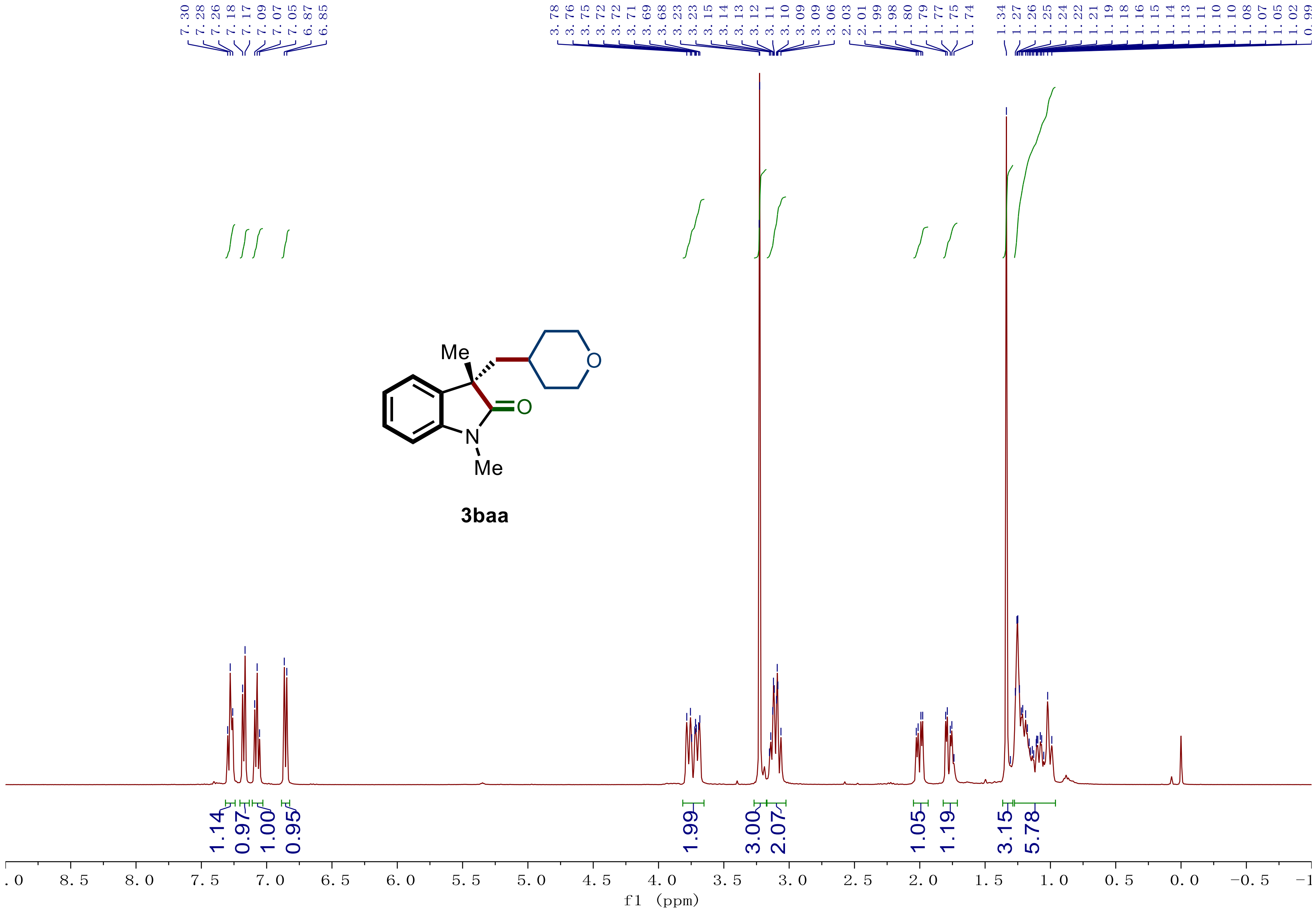

Supplementary Figure 239

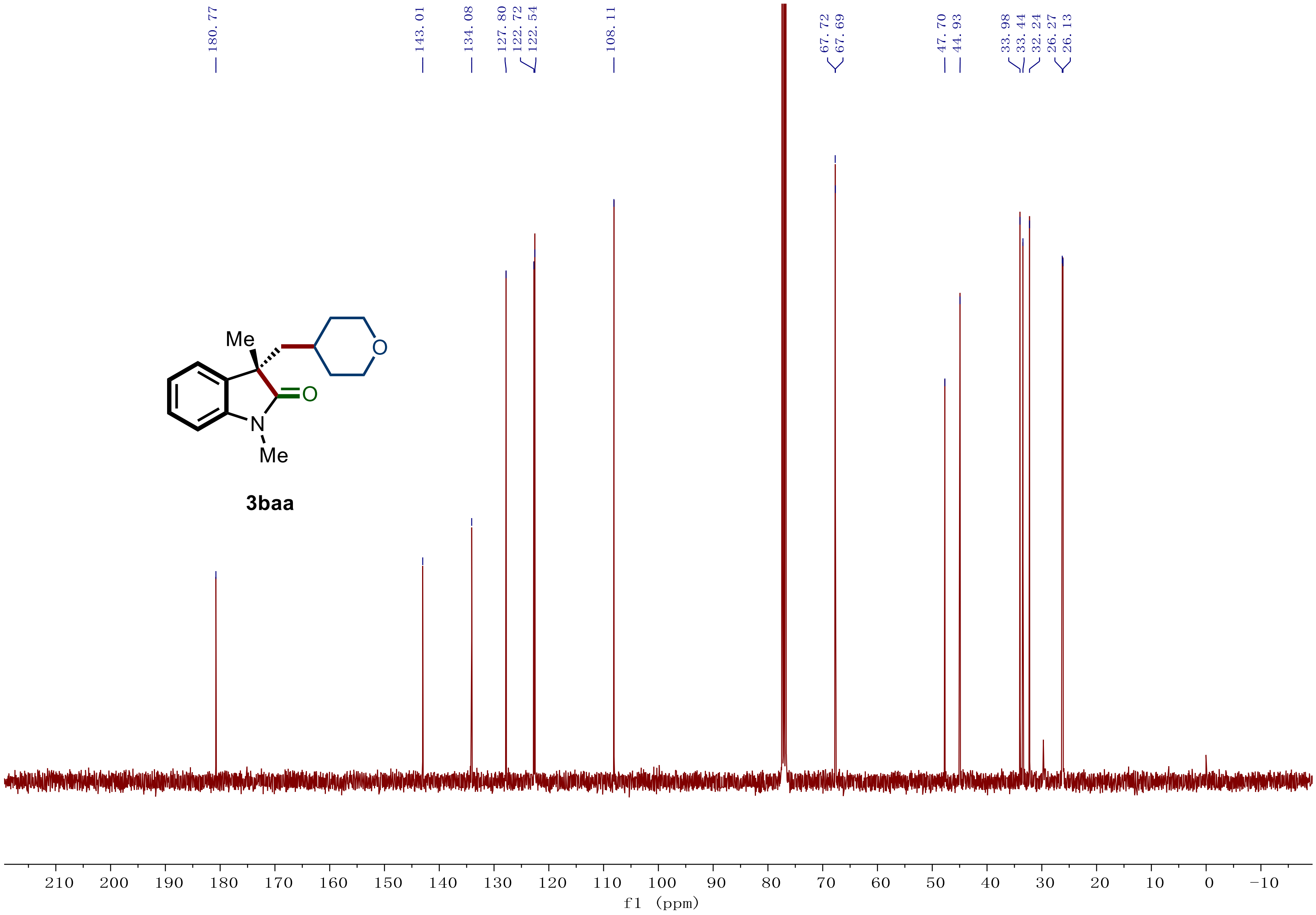

3baa

Supplementary Figure 240

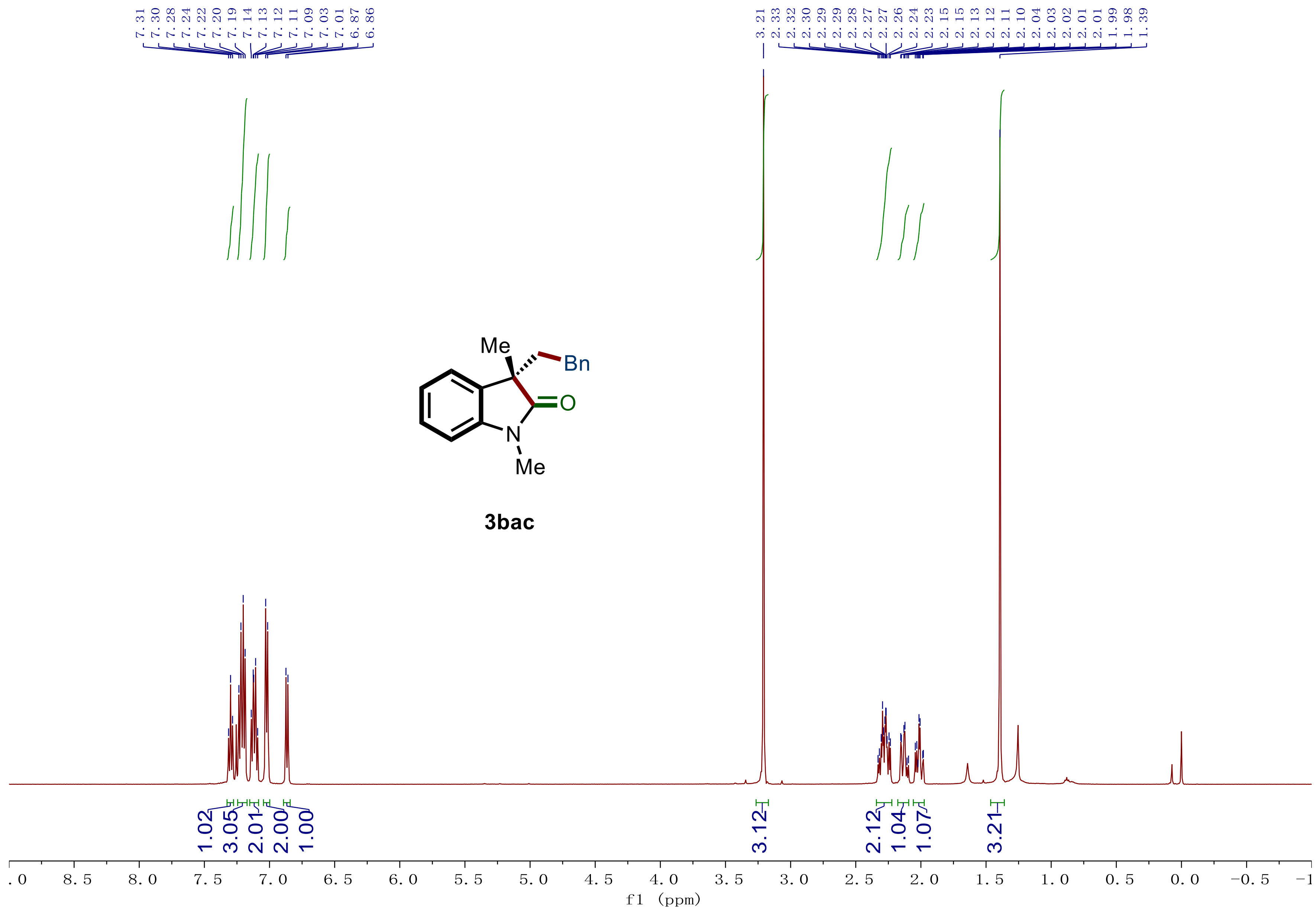

3bac

Supplementary Figure 241

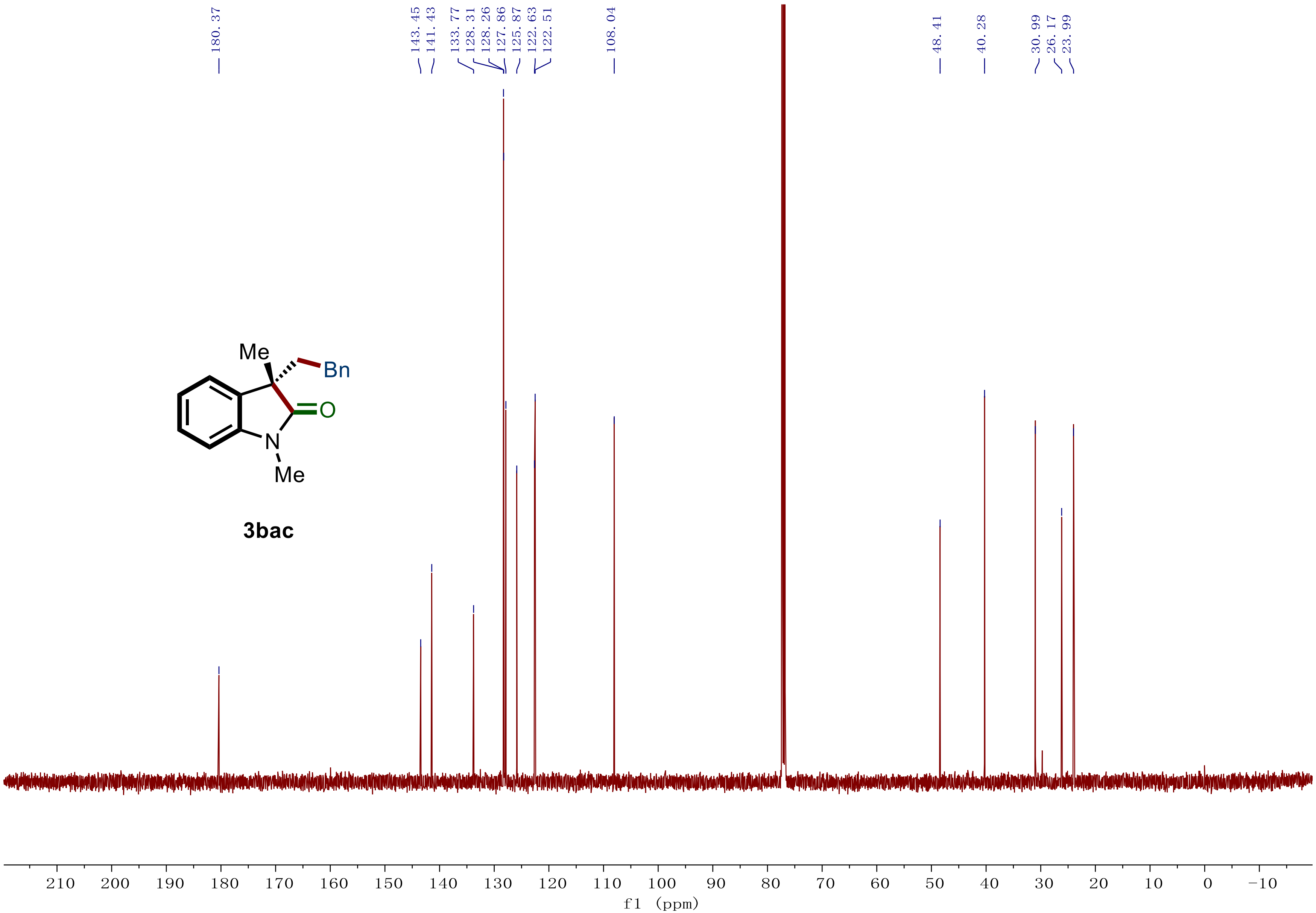

Supplementary Figure 242

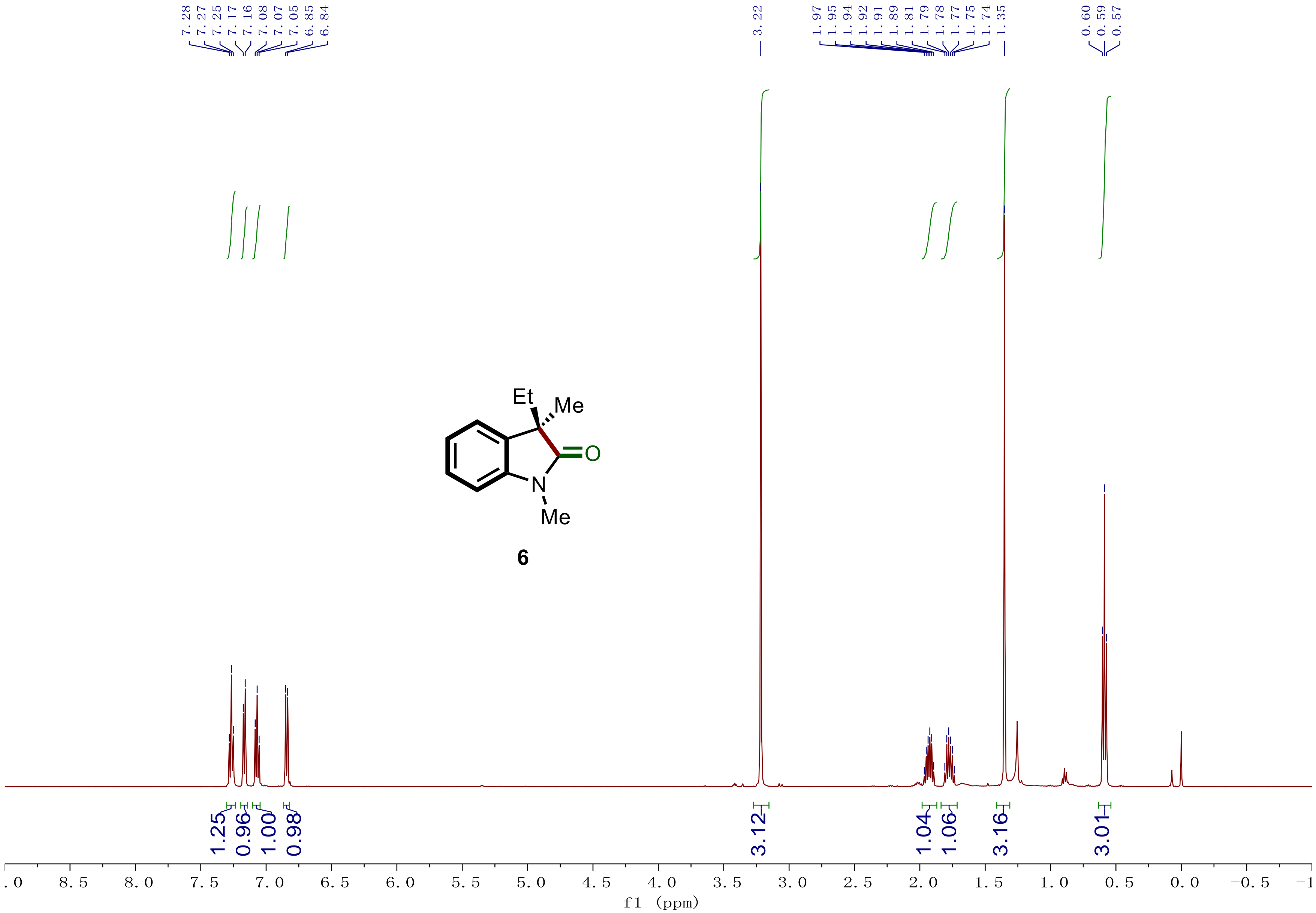

Supplementary Figure 243

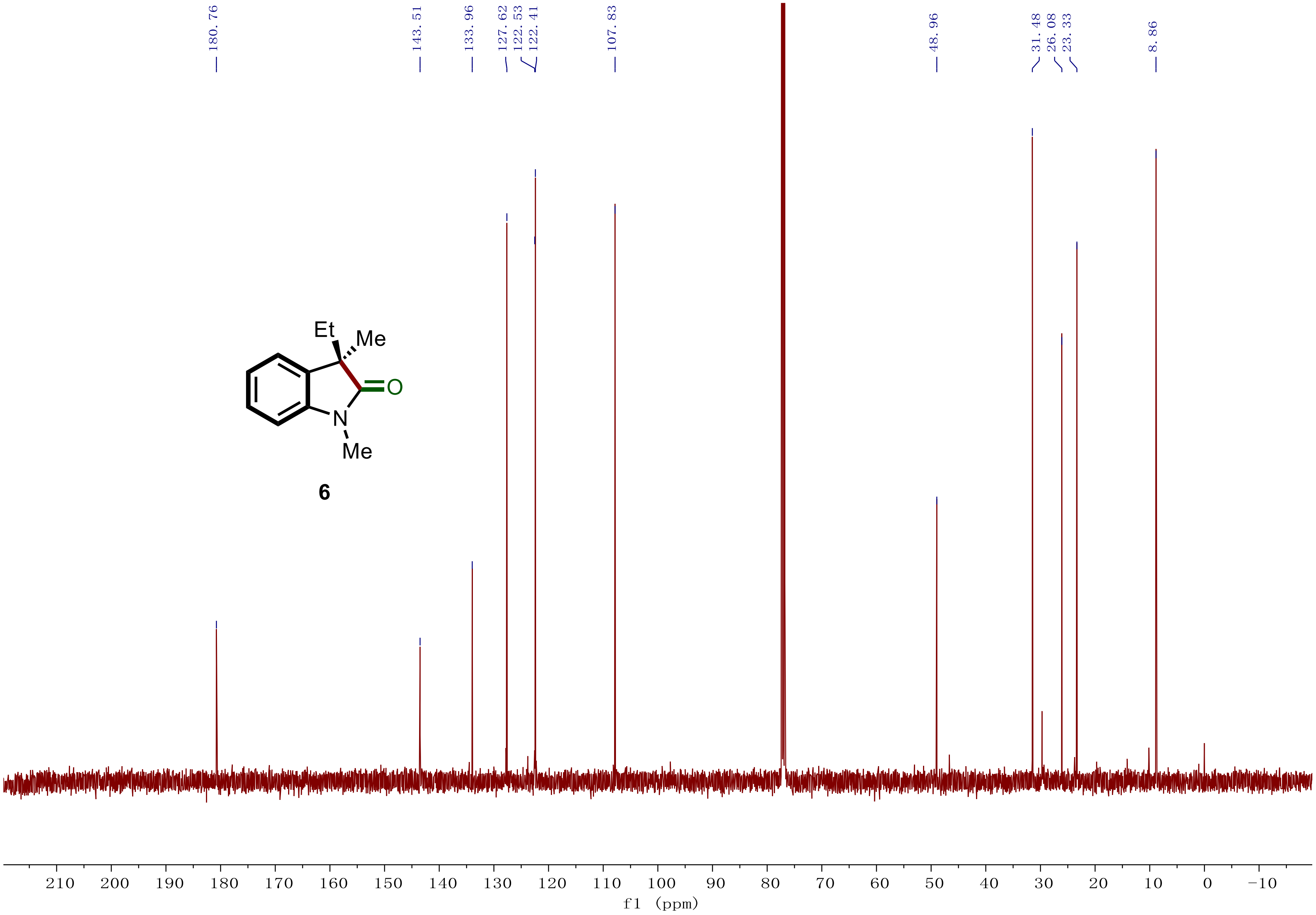

Supplementary Figure 244

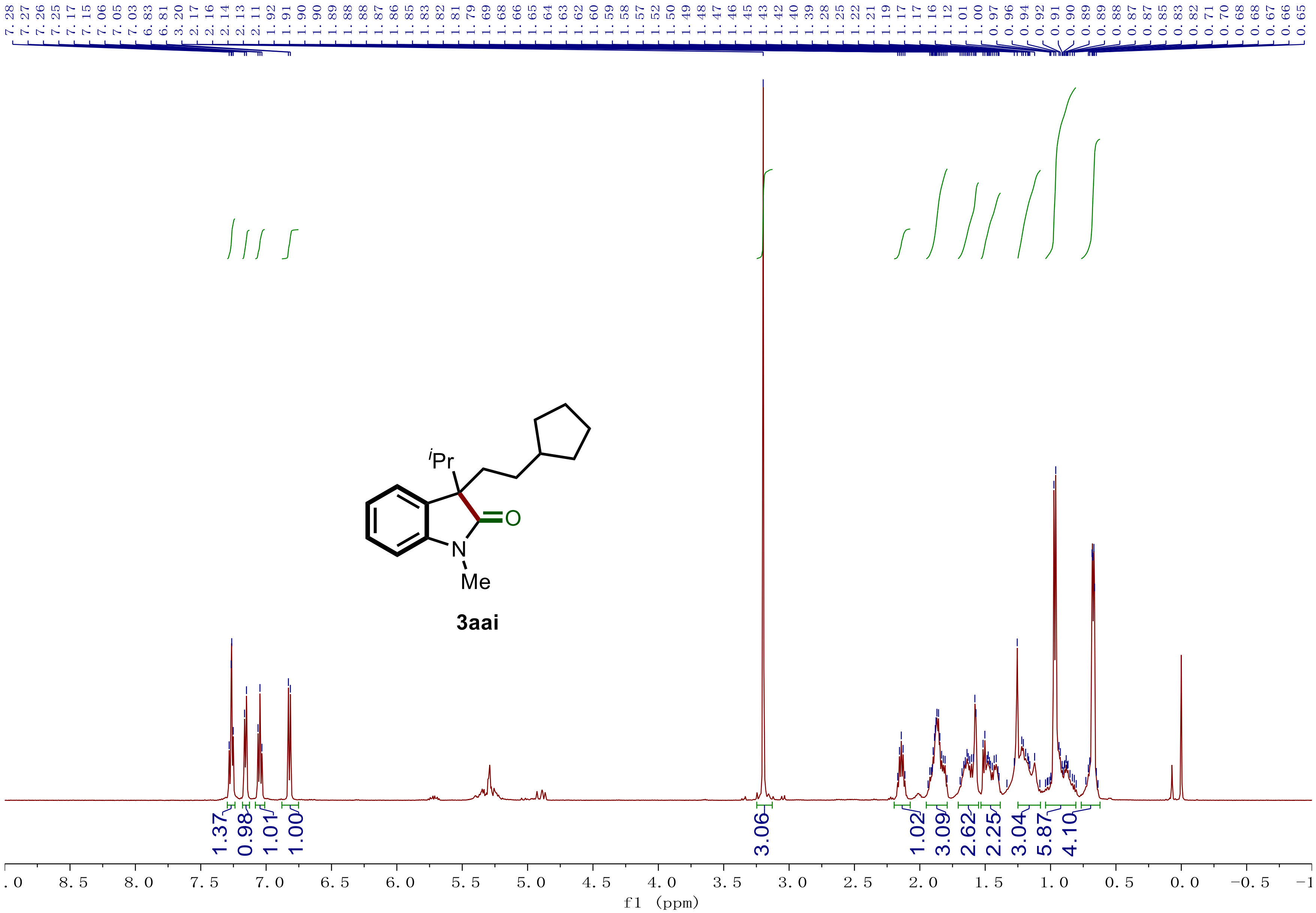

3aai

Supplementary References

1. Gomes, C. S. B., Costa, S. I., Silva, L. C., Jiménez-Tenorio, M., Valerga, P., Puerta, M. & Gomes, P. T. Cationic R-Substituted-Indenyl Nickel(II) Complexes of Arsine and Stibine Ligands: Synthesis, Characterization, and Catalytic Behavior in the Oligomerization of Styrene. *Eur. J. Inorg. Chem*. **2018**, 597−607 (2018).
2. Whyte, A., Burton, K. I., Zhang, J. & Lautens, M. Enantioselective Intramolecular Copper-Catalyzed Borylacylatio.n *Angew. Chem., Int. Ed*. **57**, 13927−13930 (2018).
3. Wang, X., Ji, X., Shao, C., Zhang, Y. & Zhang, Y. Palladium-Catalyzed C–H Alkylation of 2-Phenylpyridines with Alkyl Iodides. *Org. Biomol. Chem*. **15**, 5616−5624 (2017).
4. Louafi, F., Moreau, J., Shahane, S., Golhen, S., Roisnel, T., Sinbandhit, S. & Hurvois, J.-P. Electrochemical Synthesis and Chemistry of Chiral 1-Cyanotetrahydroisoquinolines. An Approach to the Asymmetric Syntheses of the Alkaloid (−)-Crispine A and Its Natural (+)-Antipode. *J. Org. Chem*. **76**, 9720−9732 (2011).
5. Szeja, W., Grynkiewicz, G., Bieg, T., Swierk, P., Byczek, A., Papaj, K., Kitel, R. & Rusin, A. Synthesis and Cytotoxicity of 2,3-Enopyranosyl C-Linked Conjugates of Genistein. *Molecules* **19**, 7072−7093 (2014).
6. Liu, Q., Hong, J., Sun, B., Bai, G., Li, F., Liu, G., Yang, Y. & Mo, F. Transition-Metal-Free Borylation of Alkyl Iodides via a Radical Mechanism. *Org. Lett*. **21**, 6597−6602 (2019).
7. Hazra, A., Lee, M. T., Chiu, J. F. & Lalic, G. Photoinduced Copper-Catalyzed Coupling of Terminal Alkynes and Alkyl Iodides. *Angew. Chem. Int. Ed*. **57**, 5492−5496 (2018).
8. Rezazadeh, S., Devannah, V. & Watson, D. A. Nickel-Catalyzed *C*-Alkylation of Nitroalkanes with Unactivated Alkyl Iodides. *J. Am. Chem. Soc*. **139**, 8110−8113 (2017).
9. Sun, L., Peng, G., Niu, H., Wang, Q. & Li, C. A Highly Chemoselective and Rapid Chlorination of Benzyl Alcohols under Neutral Conditions. *Synthesis* **40**, 3919−3924 (2008).

10. Zhou, F., Zhang, Y., Xu, X. & Zhu, S. NiH-Catalyzed Remote Asymmetric Hydroalkylation of Alkenes with Racemic α-Bromo Amides. *Angew. Chem., Int. Ed*. **58** 1754−1758 (2019).

11. Taylor, A. M., Altman, R. A. & Buchwald, S. L. Palladium-Catalyzed Enantioselective α-Arylation and α-Vinylation of Oxindoles Facilitated by an Axially Chiral P-Stereogenic Ligand. *J. Am. Chem. Soc*. **131**, 9900−9901 (2009).
